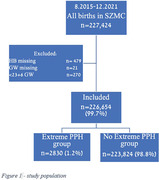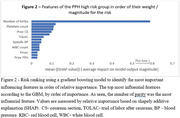# Poster Session 4: Friday, January 31, 2025 4:00 PM – 6:00 PM

**DOI:** 10.1002/pmf2.12030

**Published:** 2025-01-05

## 948 | Neighborhood Deprivation Associated with Extremely Preterm Birth during COVID‐19 Pandemic

Abigail Bell^1^; Megan Foeller^1^; Candice Woolfolk^1^; Emily Dively, BSN^2^; Fan Zhang^1^; Daniel Jackson^3^; Nandini Raghuraman^4^; Indira U. Mysorekar^5^; Jeannie C. Kelly^4^

*^1^Washington University School of Medicine in Saint Louis, St. Louis, MO; ^2^Washington University School of Medicine, St. Louis, MO; ^3^Mercy Hospital, St Louis, MO; ^4^Washington University School of Medicine in St. Louis, St. Louis, MO; ^5^Baylor College of Medicine, Houston, TX*

4:00 PM ‐ 6:00 PM

**Objective**: Area Deprivation Index (ADI) is strongly associated with obstetric outcomes but understudied during the COVID‐19 pandemic. We sought to evaluate the relationship between ADI and preterm birth during the early pandemic.

**Study Design**: We performed a secondary analysis of a prospective longitudinal cohort study investigating the impact of COVID‐19 exposure in pregnancy on perinatal outcomes. Pregnant patients were recruited 12/23/20‐7/18/22 and serially tested with serum antibody testing for COVID‐19 exposure during pregnancy. Delivery residence address was used to categorize patients into low ADI (≤ 50^th^ percentile) and high ADI (≥ 75^th^ percentile, indicating high level of deprivation) groups. The primary outcome was preterm birth, using multivariable regression and stratified by gestational age at birth; secondary outcomes included other perinatal complications.

**Results**: 306 patients were included. Overall, preterm birth rates did not differ between high and low ADI groups; however, low ADI was significantly associated with lower rates of extreme preterm birth < 28 weeks and < 34 weeks (Table 1). After adjusting for obesity and tobacco use, the association persisted at < 28 weeks (aOR 0.26, 95% CI 0.07‐0.73) and < 34 weeks (aOR 0.35 95% CI 95% 0.14‐0.87; Table 2). There were no other differences found in perinatal outcomes except for a higher rate of neonatal intracranial hemorrhage in the low ADI group (3.2% vs 0%, p = 0.03).

**Conclusion**: High ADI was associated with increased risk of extremely preterm birth during the early COVID‐19 pandemic even when controlling for obesity and tobacco use. The interplay of social determinants of health and preterm birth must be better studied to target interventions to modifiable risk factors.

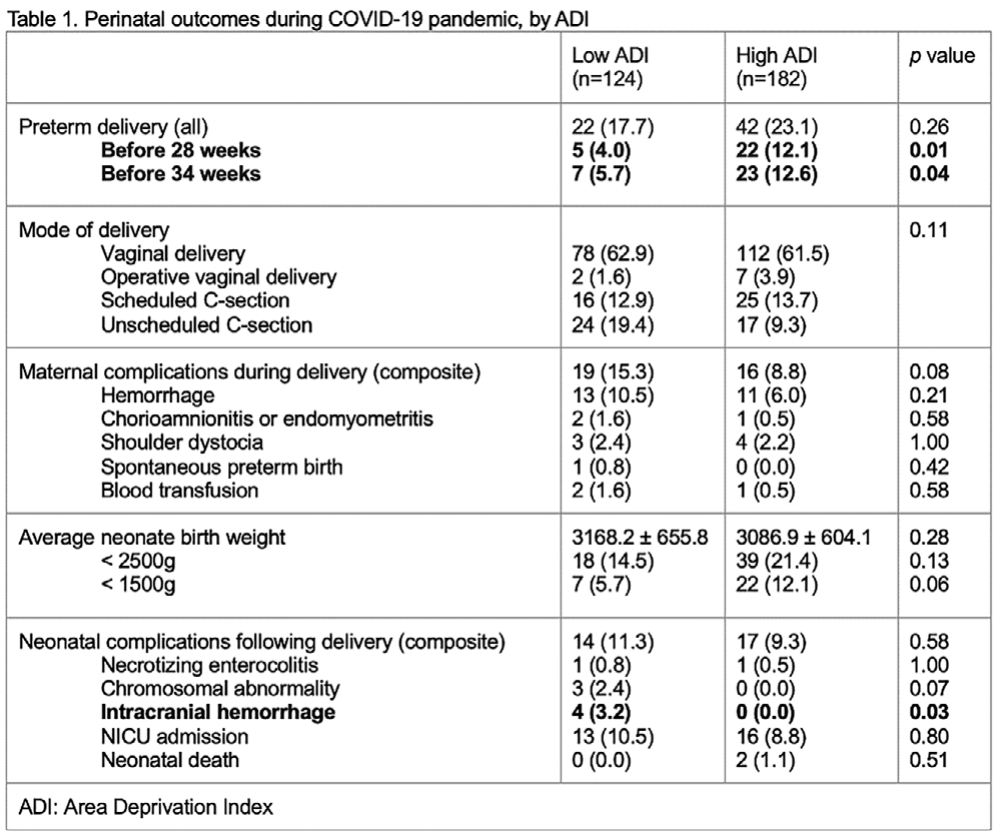

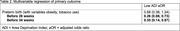

## 949 | Real‐World Accuracy of Angiogenic Factors for Predicting Severe Preeclampsia in Singleton and Twin Gestations

Abraham Tsur^1^; David Nadav Sabag^2^; Ronen Fluss^3^; Amit Huppert^3^; Michal Fishel Bartal^4^; Dan Dominissini^5^; Yoav Yinon^6^; Shali Mazaki Tovi^6^; Rakefet Yoeli‐Ullman^7^

*^1^Sheba Medical Center, Ramat Gan, Tel Hashomer, Israel, Ramat Gan, HaMerkaz; ^2^Sheba Medical Center, Ramat Gan, Tel Hashomer, Israel, Givatayim, HaMerkaz; ^3^Gertner Institute, Ramat Gan, HaMerkaz; ^4^UTH Houston & Sheba Medical Center Israel, Houston, TX; ^5^The sheba Medical Center, Ramat Gan, HaMerkaz; ^6^The Sheba Medical Center, Ramat Gan, HaMerkaz; ^7^Sheba Medical Center, Ramat Gan, HaMerkaz*

4:00 PM ‐ 6:00 PM

**Objective**: To determine real‐world accuracy of circulating angiogenic factors in predicting preeclampsia with severe features (sPE) in singleton and twin gestations.

**Study Design**: This retrospective cohort study consisted of individuals admitted to the antepartum unit of a tertiary academic center from 2023 to 2024. We included individuals with hypertensive disorders of pregnancy (HDP) at 23+0 to 34+6 weeks of gestation with available sFlt‐1/PlGF measurements obtained as part of the clinical flow. Exclusion criteria included developing sPE on the day of measurement. The area under the curve (AUC) was used to assess accuracy of the prediction of sPE within two weeks. The discriminatory ratio of ≥40 was evaluated for clinical performance. Similar to the PRAECIS study, measurements were re‐included if delivery did not occur within two weeks. In addition, we developed a logistic regression model integrating sFlt‐1/PlGF with blood pressure (BP). Prediction of sPE by the integrated model was compared to prediction based on sFlt‐1/PlGF alone using AUC and likelihood ratio test (LRT).

**Results**: The study group included 59 individuals (71 sflt‐1/PlGF measurements). In 35% of measurements, the individuals developed sPE within two weeks. Table 1 reports the discriminatory clinical performance of the ≥40 ratio stratified by singleton and twin pregnancies. The ≥40 ratio showed better sensitivity and NPV in twins than in singletons. Moreover, although specificity in twins was lower than in singletons, the PPV was higher in twins due to the increased prevalence of sPE. Integrating sFlt‐1/PlGF with BP measurement increased the model accuracy (AUC 0.83 VS 0.88, figure 1) and enhanced the model fit (LRT *p* = 0.005).

**Conclusion**: Real‐world evidence supports extending the clinical use of the sFlt‐1/PlGF ratio to assess the risk of developing sPE in twin pregnancies as well. Integrating sFlt‐1/PlGF with clinical risk factors like BP can enhance predictive performance and clinical decision‐making.

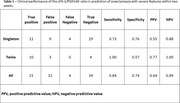

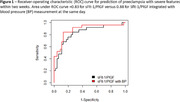

## 950 | What to Expect of Expectant Management in Preterm Preeclampsia with Severe Features

Ahmed Zaki Moustafa^1^; Beatrice Valentini^2^; Joe Haydamous^3^; Cabrina Becker^4^; Jasmin Abdeldayem^5^; Khalil M. Chahine^6^; Sean C. Blackwell^7^; Baha M. Sibai^7^

*^1^Division of Maternal‐Fetal Medicine, Department of Obstetrics, Gynecology and Reproductive Sciences, McGovern Medical School at UTHealth Houston, University of Texas ‐ Houston, TX; ^2^University of Parma, University of Parma, Emilia‐Romagna; ^3^Division of Maternal‐Fetal Medicine, Department of Obstetrics, Gynecology and Reproductive Sciences, McGovern Medical School at UTHealth Houston, Houston, TX; ^4^UT Health Science Center at Houston, McGovern Medical School, Houston, TX; ^5^Texas Tech Health Sciences Center of El Paso, El Paso, TX; ^6^McGovern Medical School at UTHealth, Houston, TX; ^7^McGovern Medical School at UTHealth Houston, Houston, TX*

4:00 PM ‐ 6:00 PM

**Objective**: Despite common practice in the US, Level I data from RCT's of expectant management of preterm preeclampsia with severe features (PSF) is limited. Of the three RCT's conducted, only one was performed in the US 30 years ago. There is also limited evidence guiding management in the setting of fetal growth restriction (FGR). Our objective was to describe maternal and perinatal outcomes with expectant management of early onset PSF by gestational age (GA) at diagnosis and by presence of FGR.

**Study Design**: This is a retrospective cohort study of pregnant individuals with a diagnosis of PSF between 24‐34 weeks at a single Level 4 center between January 2022 to April 2024. Exclusion criteria was delivery < 24 hours of admission. We compared the latency period (time interval from diagnosis to delivery) by GA at diagnosis and presence or absence of FGR. Indications for delivery, maternal and perinatal outcomes were evaluated.

**Results**: Over the study period, 241 patients were admitted with PSF < 34 weeks and 73% (n = 175) met criteria for analysis. Patients were divided by GA: 24w0d–27w6d (n = 43), 28w0d–31w6d (n = 90), and 32w0d–33w‐6d (n = 42). There were no significant differences in maternal characteristics.

Latency was longer with earlier GA at PSF diagnosis. The median (IQR) latency in days was 6.4 (2.7–14.2) in group 1, 4.2 (2.5‐11.0) in group 2, and 3.0 (1.7‐6.4) in group 3 (p = 0.024).

Rate of FGR was significantly higher with earlier onset at 51%, 46%, and 21% in groups 1, 2, and 3 (p = 0.01) and was associated with decreased latency though it did not reach statistical significance (Figure 1). Latency when PSF is diagnosed beyond 32 weeks in the presence of FGR was only 1.7 days (1.5–7.6). Fetal indications for delivery were higher in group 1 (65 vs 47, and 24%, p = 0.002) (Table 1).

**Conclusion**: In pregnancies complicated by preterm PSF, latency decreased with advancing gestational age at diagnosis especially in the setting of FGR. Further studies are needed to evaluate if there is a benefit in attempting expectant management beyond administration of corticosteroids when PSF is diagnosed beyond 32 weeks with FGR.

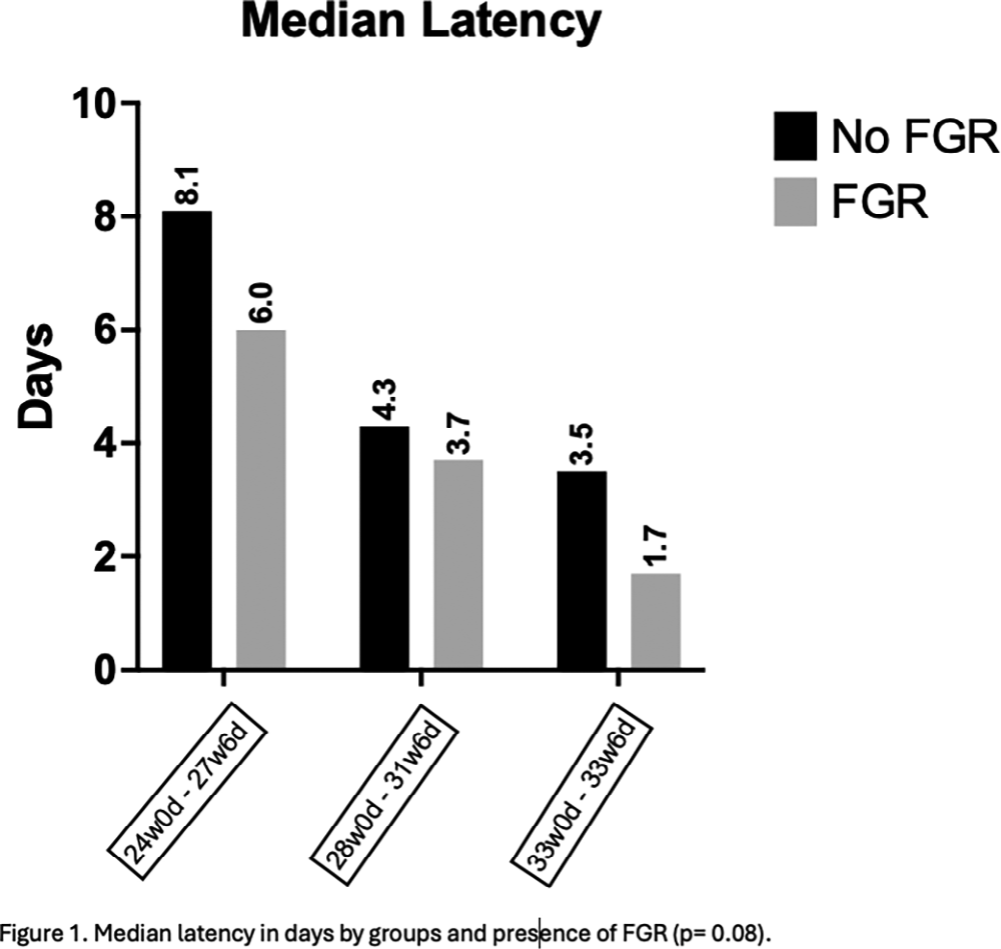

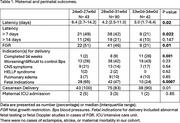

## 951 | Early Anatomic Ultrasound to Detect Fetal Anomalies in High Risk Patients at 12‐14 Weeks Gestation

Akos Herzeg^1^; Yili Zhao^1^; Mary E. Norton^2^

*^1^UCSF, San Francisco, CA; ^2^Center for Maternal‐Fetal Precision Medicine, University of California San Francisco, San Francisco, CA*

4:00 PM ‐ 6:00 PM

**Objective**: Detailed evaluation of the fetus in the late first trimester is now possible. We report on the outcomes of a cohort of high‐risk patients who underwent detailed fetal evaluation at 12‐14 weeks’ gestation (WG).

**Study Design**: Patients referred for early anatomy scans underwent detailed evaluation using a protocol based on AIUM practice parameters. Outcomes of all referred patients from 01/2021 through 07/2024 were collected. Variables included referral indications, maternal age, gestational age, need for endovaginal scan, detection of minor and major anomalies, genetic testing results, and outcomes based on second‐trimester anatomy scan, fetal echo, or at birth.

**Results**: In all, 217 patients were included. Indications included prior pregnancy with genetic or structural anomalies (34%), maternal age or patient request (14%), abnormal screening (10%), suspected abnormalities on a routine scan (8%), teratogen exposure (7%), perinatal risk factors (5%), and complicated twins (1%). Median gestational age was 13+2 weeks’ (IQR 1+1), 1.4% were twins and 2.8% required an endovaginal scan. In all, 55 (25.3%) had abnormal results: 36 of these were confirmed, 1 minor finding was not confirmed, 3 had fetal demise, 14 terminated, and 1 pregnancy is ongoing. Terminations without confirmation were for severe limb anomalies (2), acrania (1), abnormal CVS (8), sirenomelia (1), and bladder outlet obstruction (2). In the 162 (74.6%) cases with normal findings, 139 were confirmed normal at 20 WG or birth, 2 had mild pelviectasis at 20 WG, 4 terminated for CVS abnormalities*, 12 had follow‐up elsewhere, and 5 have ongoing pregnancies. No major abnormalities were detected, and the only false positive result was a suspected persistent left superior vena cava that was not confirmed on fetal echo.

**Conclusion**: Dedicated anatomic evaluation at 12‐14 weeks’ gestation in high‐risk patients has a high yield of abnormal findings and a high accuracy. For patients at high‐risk, this option can provide earlier detection of significant anomalies as well as earlier reassurance/counseling.

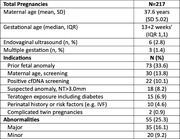

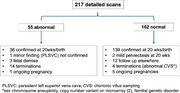

## 952 | Establishing Blood Loss Thresholds Predictive of Transfusion at the Time of Delivery

Alesha M. White^1^; Kristina Fin^1^; Jessica E. Pruszynski^2^; R. Nicholas Burns^2^; Anne M. Ambia^2^; Brenda Anyaehie^1^; Elaine L. Duryea^1^

*^1^University of Texas Southwestern Medical Center, Dallas, TX; ^2^University of Texas Southwestern, Dallas, TX*

4:00 PM ‐ 6:00 PM

**Objective**: In 2014, the American College of Obstetricians and Gynecologists established a standardized definition of postpartum hemorrhage (PPH) to be blood loss greater than 1000mL from any form of delivery. Prior to this in 1965, Pritchard established the definition of normal blood loss for spontaneous vaginal deliveries (SVD) to be 500mL and for cesarean deliveries (CD) to be 1000mL. Our objective was to establish blood loss thresholds at the time of delivery as they relate to transfusion using quantitative blood loss (QBL).

**Study Design**: This is a prospective observational study conducted from November 2023 until March 2024 at a single institution that included all SVDs and CDs at which QBL was collected. QBL was calculated and recorded in the electronic medical record by the nursing team, but not reported to providers, who used estimated blood loss (EBL) and clinical indices to guide clinical care, per existing practices. Receiver operating characteristic (ROC) curves were generated based on the probability of transfusion.

**Results**: QBL data was available for 4,543 deliveries. 3,236 (71%) of total deliveries were via SVD and 1,234 (27%) of deliveries were via CD. Based on an ROC curve for SVDs, the optimal cut point for transfusion was determined to be 449.5mL. This represented a sensitivity of 71% and a specificity of 79% with an AUC of 0.81. Based on an ROC curve for CDs, the optimal cut point for transfusion was determined to be 1012.5mL. This represented a sensitivity of 61% with a specificity of 72% and an AUC of 0.7. The prevalence of transfusion at each respective cut point for type of delivery represented a 10.90% prevalence of transfusion for CDs and a 4.60% prevalence of transfusion for SVDs.

**Conclusion**: The QBL cut points associated with transfusion in this study are very similar to prior cut points determined to represent normal blood loss at delivery, which is known to differ by mode of delivery. Such that, not all deliveries should be classified as normal based on a 1000mL cut point alone and a heightened awareness should be present when QBL exceeds 500cc for SVD.

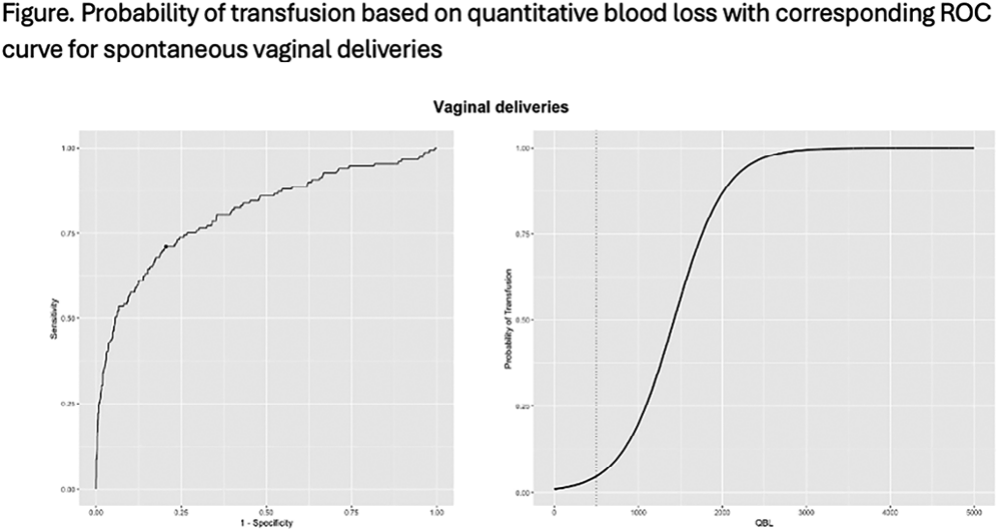

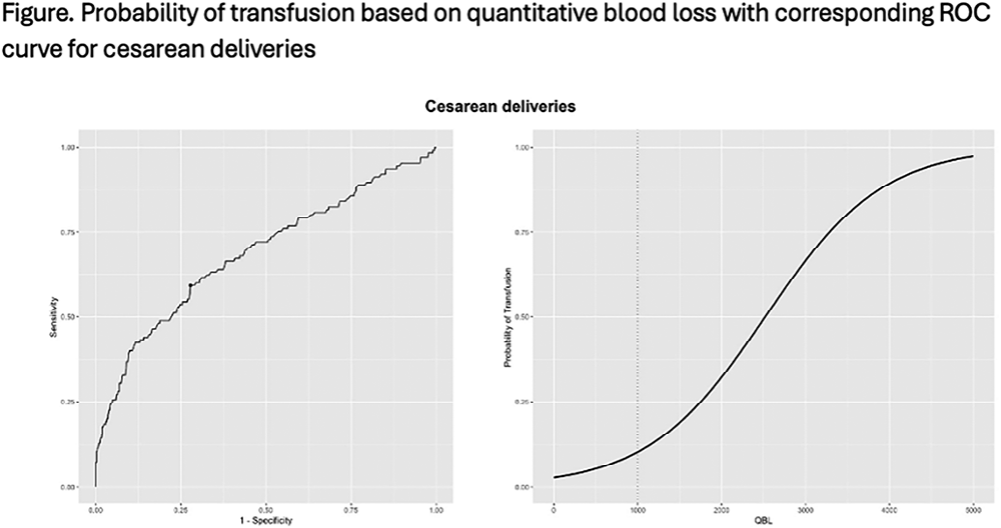

## 953 | Defining Postpartum Hemorrhage in Vaginal Deliveries Based on Quantitative Blood Loss

Alesha M. White^1^; Kristina Fin^1^; Jessica E. Pruszynski^2^; R. Nicholas Burns^2^; Anne M. Ambia^2^; Brenda Anyaehie^1^; Elaine L. Duryea^1^

*^1^University of Texas Southwestern Medical Center, Dallas, TX; ^2^University of Texas Southwestern, Dallas, TX*

4:00 PM ‐ 6:00 PM

**Objective**: Postpartum hemorrhage (PPH) is a leading cause of maternal mortality across the world. In 2014, the American College of Obstetricians and Gynecologists (ACOG) defined PPH as blood loss ≥ 1000cc regardless of delivery mode. The objective of this study was to compare the sensitivity of various quantitative blood loss (QBL) thresholds for detection of need for transfusion for spontaneous vaginal deliveries (SVD), including maternal anemia status.

**Study Design**: This is a prospective observational study conducted at a single institution including all SVDs at which QBL was collected. QBL was calculated and recorded in the electronic medical record by the nursing team, but not reported to providers, who used estimated blood loss (EBL) and clinical indices to guide clinical care, per existing practices. Performance characteristics of QBL cut points of 500cc, 750cc, and 1000cc were examined, including examination according to maternal anemia at presentation for delivery antepartum, defined as a hematocrit < 33 based on current recommendations from ACOG.

**Results**: QBL was available for 3,250 SVDs between November 1st 2023 until March 31st 2024. The prevalence of transfusion for a QBL ≥1,000cc was 4.5%. A universal cut‐point of 1,000cc demonstrated a 0.34 sensitivity for transfusion with a positive likelihood ratio (PLR) of 16.35. Maternal anemia was present at admission in 444 (14%) patients. The prevalence of transfusion for patients with and without anemia was 14.20% and 2.9% respectively. PLR reached 10 (a common threshold) at the 750cc cut point for patients with and without anemia, however the sensitivity of this cut point was lower for patients with anemia (0.41) compared to those without (0.6) (Table). A cut point of 500cc in patients with anemia achieved a sensitivity of 0.52.

**Conclusion**: Use of 1000cc as a definition of PPH for SVD results in a remarkably high PLR for transfusion while failing to detect the majority of patients requiring transfusion. Use of a cut point of 750cc for patients without anemia, and 500cc for patients with anemia is recommended.

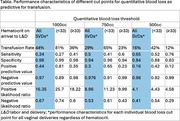

## 954 | Oxytocin Requirements Among Patients with a BMI Greater Than 30

Alexander M. Saucedo^1^; Miriam Alvarez^2^; Alison G. Cahill^3^; Lorie M. Harper^2^

*^1^Baylor College of Medicine, Houston, TX; ^2^Dell Medical School, Austin, TX; ^3^Dell Medical School Health Transformation Research Institute, Dell Medical School, The University of Texas at Austin, Austin, TX*

4:00 PM ‐ 6:00 PM

**Objective**: Patients with obesity undergoing induction of labor (IOL) may require increased oxytocin (OT) administration when compared to normal weight patients. The OT product (mU/min x hours), defined by multiplying the maximum OT rate (mU/min) by the total OT duration (hours), is a labor variable that highly correlates with total OT dose received in labor. We aimed to determine the effect of increasing obesity on the OT product in obese patients undergoing IOL.

**Study Design**: Secondary analysis of a single center, double‐blinded RCT between 6/2022 and 7/2023, which randomized near‐term, singleton gestations with a BMI ≥ 30 kg/m^2^ undergoing IOL, with a cervical dilation ≤ 3cm, to either 25mcg or 50mcg of vaginal misoprostol. For this analysis, patients were excluded if they did not receive OT during labor. We compared patients with a BMI of 30‐39 kg/m^2^ to those with a BMI ≥ 40 kg/m^2^. The primary outcome was the overall OT product. Multivariable linear regression was used to adjust for randomization group, nulliparity, cervical ripening balloon use, and cervical dilation on admission.

**Results**: A total of 154 patients received OT with 109 (70.8%) in the BMI 30‐39 group versus 45 (29.2%) in the BMI 40+ group. Baseline characteristics were similar. Following adjustment, an increased but non‐significant OT product was seen in the BMI 40+ group (143.09 vs. 191.78 mU/min‐hours; p = 0.19). Among only those who underwent vaginal delivery, a similar OT product was seen between groups (128.87 vs. 130.17 mU/min‐hours; p = 0.96). Patients in the BMI 40+ group did have an increased overall time to delivery (20.38 hrs vs. 24.89 hrs; p = 0.03), increased risk for failed IOL (3.7% vs. 24.4%; p = 0.01), and an increased quantitative blood loss at delivery (345 mL vs. 450 mL; p = 0.04).

**Conclusion**: Patients in the BMI 40+ group had an increased but non‐significant OT requirement when compared to the BMI 30‐39 group. This OT difference was reduced when excluding patients who underwent cesarean. Increasing obesity was associated with an increased duration of labor, cesarean due to failed IOL, and increased blood loss at delivery.

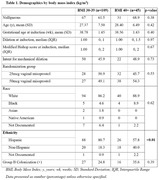

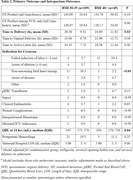

## 955 | Misoprostol Dose Requirements Among Patients with a BMI Greater Than 30

Alexander M. Saucedo^1^; Miriam Alvarez^2^; Alison G. Cahill^3^; Lorie M. Harper^2^

*^1^Baylor College of Medicine, Houston, TX; ^2^Dell Medical School, Austin, TX; ^3^Dell Medical School Health Transformation Research Institute, Dell Medical School, The University of Texas at Austin, Austin, TX*

4:00 PM ‐ 6:00 PM

**Objective**: Compared to normal weight persons, patients with obesity experience fewer uterine contractions after administration of vaginal misoprostol (VM). Therefore, patients with obesity may require higher doses of VM for induction of labor (IOL). We aimed to determine the effect of increasing obesity on the total VM dose requirement in obese patients undergoing IOL.

**Study Design**: Secondary analysis of a single center, double‐blinded RCT between 6/2022 and 7/2023, which randomized near‐term, singleton gestations with a BMI ≥ 30 kg/m^2^ undergoing IOL, with a cervical dilation ≤ 3cm, to either 25mcg or 50mcg of VM. For this analysis, we included all patients from the primary study. We compared patients with a BMI of 30‐34.9 kg/m^2^ to those with a BMI ≥ 35 kg/m^2^. The primary outcome was the overall misoprostol dose required during IOL. Multivariable linear regression was used to adjust for randomization group, nulliparity, cervical ripening balloon use, and cervical dilation on admission.

**Results**: A total of 179 patients were enrolled with 74 (41.3%) in the BMI 30‐34.9 group versus 105 (58.7%) in the BMI 35+ group. Baseline characteristics were similar between groups with the majority of patients having an unfavorable cervical exam upon initiation of IOL. Following adjustment, patients in the BMI 35+ group required an increased overall misoprostol dose requirement during IOL (55.0 mcg vs. 66.2 mcg; P = 0.02). While cesarean delivery did not differ between BMI groups, the indication for cesarean was significantly different between groups, with more patients in the BMI 35+ group being diagnosed with failed IOL (4.1% vs. 12.4%; P = 0.04). No other differences in maternal/neonatal outcomes were seen.

**Conclusion**: In patients undergoing IOL, increasing obesity (BMI ≥ 35 kg/m^2^) was associated with an increased overall misoprostol dose requirement as well as an increased risk for cesarean due to failed IOL. These findings highlight the potential pharmacokinetic differences with increasing obesity and vaginal misoprostol which may impact overall IOL efficacy.

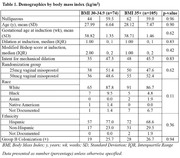

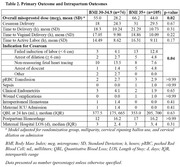

## 956 | Does Increased Antenatal Depression Screening Impact Postpartum Depression Scale Scores?

Alexandra L. Berra^1^; Kelly S. Gibson^2^; Anna Rybinska‐Campbell^3^; Katlyn Sullivan^2^

*^1^Case Western Reserve University Metrohealth Medical Center, Cleveland, OH; ^2^MetroHealth Medical Center / Case Western Reserve University, Cleveland, OH; ^3^Metrohealth, Cleveland, OH*

4:00 PM ‐ 6:00 PM

**Objective**: With mental health disorders a leading cause of maternal mortality, perinatal mental health screening and care is a strategy for prevention. The use of a validated screening tool, including the Edinburgh Postnatal Depression Scale (EPDS) has been recommended, but the optimal timing for screening and the impact of repeated screening has yet to be established. This study aimed to assess whether prenatal screening and identification of depression impacted postpartum EPDS scores.

**Study Design**: In this retrospective single site study, pregnant persons who enrolled in prenatal care prior to 20w gestation and delivered at the same institution from 2018‐2024 were included (N = 6,735). EPDS screening was performed at postpartum visits only until 2023 when EPDS was given at entry to care, during the third trimester, while inpatient postpartum and at postpartum visits. A score of >12 was considered positive for depression. Prenatal screening, socio‐demographic profiles, and PPD prevalence were analyzed pre/post implementation using t‐tests, chi‐square tests, and multiple variable logistic regression.

**Results**: Overall, 57.8% of patients delivered prior to the expanded EPDS implementation. The mean age at delivery was 28.5±6.0 years, 46.6% identified as White, 39.6% as Black, and 18.2% were Hispanic. Prior to implementation, 12.7% of all patients had a high postpartum EPDS score compared to 7.4% after (p< 0.001). Adjusting for demographic factors, patients who delivered after the implementation were less likely to have a high postpartum EPDS score (OR: 0.21, 95%CI: 0.19‐0.24, p‐value< 0.001). This association remained after accounting for additional screening (OR: 0.29, 95%CI: 0.26‐0.33, p‐value< 0.001).

**Conclusion**: Serial antenatal depression screening was associated with lower postpartum EPDS scores. Future investigation will examine the relationship between pre‐existing mental health disease, prenatal identification, and referral on postpartum depression screening patterns.

## 957 | The Impact of the SCOTUS Dobbs Decision on Adolescent Postpartum Contraceptive Method Selection

Zuha Khan^1^; Alicia Mastronardi^2^; Megan L. Young^3^; Zachary J. Shelton^2^; William S. Havron, IV^4^; Jill M. Maples^5^; Nikki B. Zite^4^

*^1^University of Tennessee, Knoxville, TN; ^2^University of Tennessee Graduate School of Medicine, Department of OB/Gyn, Knoxville, TN; ^3^University of Tennessee Medical Center, Center for Women and Infants, Knoxville, TN; ^4^University of Tennessee Graduate School of Medicine, Knoxville, TN; ^5^University of Tennessee Health Science Center, College of Medicine, Knoxville, TN*

4:00 PM ‐ 6:00 PM

**Objective**: To investigate the potential impact of the SCOTUS Dobbs Decision on postpartum adolescent contraceptive method selection immediately postpartum in a state with high teen birth rates.

**Study Design**: This retrospective cohort study used electronic health delivery records two years before (May 2020‐April 2022) and after (May 2022‐April 2024) the Dobbs Decision leak. Patients < 20 years old were analyzed and those with no documented contraceptive preference were excluded. Contraceptive methods were categorized based on the American College of Obstetricians and Gynecologists efficacy categories: highly effective (implant, IUD, sterilization), medium effective (injection, pill, patch, ring), least effective (condom, fertility awareness‐based methods), and no plan/abstinence. Pearson Chi‐square tests were performed in SPSS to compare contraceptive methods, recall of prenatal contraceptive counseling, and other demographic characteristics including residence categorized by March of Dimes maternity care access level, race and ethnicity, and insurance status.

**Results**: Analysis included 786 adolescent births (47.7% pre‐Dobbs; 52.3% post‐Dobbs). After the Dobbs decision, a higher percentage of postpartum adolescents opted for no contraceptive method (1.9%, 9.2%), while fewer adolescents opted for the highly effective contraception (44.0%, 38.7%), (p< 0.001). Additionally, recall of contraceptive counseling decreased post‐Dobbs (59.7% to 48.7%, p =  .027). Demographics considered, including maternity care access level, were not significantly different between contraceptive method efficacy categories.

**Conclusion**: Postpartum adolescents showed a greater tendency to select no contraception post‐Dobbs. The decreased recollection of prenatal contraceptive counseling may contribute to that finding. Greater effort is needed to ensure prenatal contraceptive counseling and access to all methods immediately postpartum as this may decrease repeat teen pregnancy.

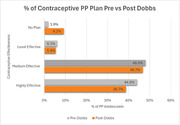

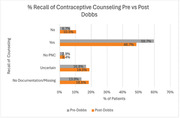

## 958 | Maternal Anxiety Moderates the Effect of Exogenous Oxytocin on the HPA axis

Alison M. Stuebe^1^; Katharine Bruce^1^; Anna E. Bauer^1^; Samantha Meltzer‐Brody^1^; Nisha O'Shea^2^

*^1^UNC School of Medicine, Chapel Hill, NC; ^2^RTI International, Research Triangle Park, NC*

4:00 PM ‐ 6:00 PM

**Objective**: Oxytocin (OT) has multiple behavioral effects. We sought to quantify the effect of inhaled OT on the HPA axis response to stress among mothers of school‐age children.

**Study Design**: We conducted a double‐blind randomized controlled trial of nasal OT embedded within the Mood, Mother, and Infant Study, a longitudinal observational cohort recruited in the 3rd trimester of pregnancy. In the first postpartum year, participants completed periodic assessments, including the Edinburgh Postnatal Depression Scale (EPDS), Spielberger State‐Trait Anxiety Inventory (STAI), and Beck Depression Inventory (BDI‐II). At about 6 years postpartum, participants were randomized to 24 IU of nasal OT or placebo, followed by the Trier Social Stress Test (TSST), which is comprised of a speech task and a math task. The TSST reliably induces large and consistent HPA responses. We quantified the extent to which postpartum depression and anxiety symptoms above vs. below the median modified the effect of OT on the lagged association between ACTH and CRT. Mixed effects models were used for repeated measures analysis with α = .05.

**Results**: Of 222 individuals in the parent study, 105 completed the RCT, and measures of ACTH and lagged CRT were available for 85. COVID interrupted data collection, and there were no significant differences between completers and non‐completers. In unstratified analysis, we found that OT vs. placebo did not modify the association between ACTH and lagged CRT (*p* = 0.21). We found an interaction between mean STAI (baseline‐ 6 months postpartum) and ACTH (*p* = 0.03, Figure 1), and between trait anxiety at 12 months and ACTH (*p* = 0.04). The EPDS‐ACTH interaction was not significant (*p* = 0.06). We found no effect modification by BDI‐II (*p* = 0.79).

**Conclusion**: Symptoms of postpartum anxiety, but not depression, were associated with modification of the effect of oxytocin on the HPA axis during a standardized social stressor. These findings suggest that dysregulation of oxytocin effects on the HPA axis may contribute to perinatal anxiety symptoms.

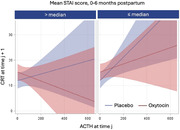

## 959 | Optimizing Postpartum Care in Pregestational Diabetes: Impact of a Comorbidity Index on Follow‐Up and Outcomes

ALISSA PRIOR^1^; Lea Almahameed^2^; Megan Weatherborn^3^; Christopher Nau^4^

*^1^The Metrohealth System/Case Western Reserve University Program, Cleveland, OH; ^2^The Metrohealth System, OH; ^3^UMass Chan Medical School ‐ Baystate, Springfield, MA; ^4^University Hospitals Cleveland Medical Center, Cleveland, OH*

4:00 PM ‐ 6:00 PM

**Objective**: Coordination of postpartum care for patients with pregestational diabetes mellitus (PDM), including timely diabetes follow‐up, is imperative for their long‐term health. At our institution, only 21% of patients have a postpartum diabetic care visit in the first 6 months postpartum. A maternal comorbidity index (CMI), a published score to summarize the severity of maternal illness, is a tool to identify high risk pregnant parturients (Table 1). In a cohort of patients with PDM, our objective was to compare a patient's CMI and postpartum outcomes. We predict a higher CMI would be associated with suboptimal glycemic control and follow up.

**Study Design**: This is a retrospective cohort study at a single urban hospital of patients with PDM who attended the center's diabetes in pregnancy clinic from 2009‐2018. Patients who delivered at an outside institution were excluded. Our independent variable was the CMI, dichotomized into low (score 1‐4) vs high (score ≥5) groups. The primary outcome was attendance of a diabetes care visit, with Primary Care or Endocrinology, in the 1st year postpartum. Secondary outcomes included postpartum HbA1c, contraception, breastfeeding, and a short‐interval pregnancy. Outcome data was analyzed with Fisher's exact, Wilcoxon rank sum, and Pearson's Chi‐squared tests.

**Results**: There were 469 patients included; CMI scores ranged from 1‐12 with 346 and 123 patients in the low and high groups, respectively. Baseline demographics, diabetes severity and type, and pregnancy outcomes were similar between groups. Attendance rates of a diabetes care visit were similar, with 51% in the low and 52% in the high CMI group (p >0.9). HbA1c median values were 7.30 in the low vs 7.85 in the high CMI group (p = 0.2). All other study outcomes were similar between groups (Table 2).

**Conclusion**: Maternal CMI was not associated with differing rates of HbA1c or follow up in patients with PDM up to 12 months postpartum. Only 50% of patients had a diabetes visit in the 1^st^ year postpartum. Further research is needed to identify patients at highest risk of poor follow up.

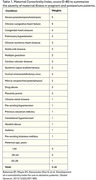

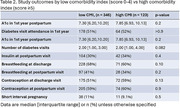

## 960 | How Pregnancy Impacts health‐Related Quality of Life: Qualitative Study in Five African and Asian Countries

Allison Sylvetsky^1^; Natalie Vallone^2^; Sasha Baumann^3^; M. Bridget Spelke^4^; Bitanya Berhane^2^; Anne G. Cherian^5^; Gregory Ouma^6^; Mahya Mehrihajmir^2^; Ellen Boamah‐Kaali^7^; Gabriela Diaz‐Guzman^2^; Muslima Ejaz^8^; Peter Otieno^6^; Winifreda M. Phiri^9^; Martha Abdulai^10^; Janae Kuttamperoor^2^; Shruti Bisht^11^; Dorothy Lall^12^; Emily Smith^1^; Piya Patel^2^; Jenifer Priya^12^; Neeraj Sharma^11^; Amna Khan^8^; Priyanka Adhikary^11^; Zahra Hoodbhoy^8^; Margaret P. Kasaro^13^

*^1^The George Washington University, The George Washington University, DC; ^2^George Washington University, Washington, DC; ^3^The George Washington University, Washington, DC; ^4^University of North Carolina at Chapel Hill; UNC ‐Global Projects Zambia, Lusaka, Lusaka; ^5^Christian Medical College, Vellore, Christian Medical College, Tamil Nadu; ^6^KEMRI, Kisumu, Nyanza; ^7^Kintampo Health Research Center, Kitampo, Brong‐Ahafo; ^8^Aga Khan University, Karachi, Sindh; ^9^UNC Lusaka, UNC Lusaka, Lusaka; ^10^Kintampo Health Research Center, Kintampo, Brong‐Ahafo; ^11^Society for Applied Studies, Delhi, Delhi; ^12^Christian Medical College, Vellore, Tamil Nadu; ^13^UNC Lusaka, UNC Zambia, Lusaka*

4:00 PM ‐ 6:00 PM

**Objective**: To examine how pregnancy‐related physical, emotional, social, and financial challenges are conceptualized, discussed, and experienced by pregnant women in six demographic and health surveillance study areas in Africa and Asia and how these challenges impact health and wellbeing during pregnancy.

**Study Design**: Thirteen focus groups discussions (FGDs) were conducted, with a total of 120 pregnant women in Ghana (2 FGDs, n = 22), Kenya (2 FGDs, n = 20), Zambia (3 FGDs, n = 23), India (4 FGDs, n = 34), and Pakistan (2 FGDs, n = 21) using a semi‐structured guide. FGDs were transcribed verbatim in the local language and translated into English. A subset of translated transcripts from each site was coded independently by two coders (one from the country of collection) to develop six preliminary site‐specific codebooks, which informed the development of two regional codebooks. All translated transcripts were coded independently by two coordinating site coders using Dedoose; themes and subthemes were identified using thematic analysis and discussed with local site teams, after which refinements were made and representative quotations were selected.

**Results**: Three overarching themes were identified: 1) Pregnancy symptoms pose a barrier to daily life; 2) Lack of social support exacerbates physical and emotional challenges during pregnancy; and 3) Social support alleviates physical, emotional, and financial challenges of pregnancy. One additional minor theme was that support from the healthcare system impacts health and wellbeing.

**Conclusion**: These findings demonstrate that pregnant women experience a variety of health and wellbeing challenges during pregnancy, which are exacerbated when social support is not available and alleviated in the context of help from family and community. Additional physical, emotional, and family and community support during pregnancy is needed to alleviate the plethora of complex and multifaceted challenges women face during pregnancy and improve their physical and mental health and overall quality of life.

## 961 | Risk of Severe Maternal Morbidity and Mortality in Relation to ARRIVE Trial

Amanda M. Baucom^1^; Heather N. Czarny^2^; Robert M. Rossi^2^; Isabella Toledo^3^

*^1^University of Cincinnati, Cincinnati, OH; ^2^University of Cincinnati College of Medicine, Cincinnati, OH; ^3^Indiana University School of Medicine, Indianapolis, IN*

4:00 PM ‐ 6:00 PM

**Objective**: Since the 2018 ARRIVE (A Randomized Trial of Induction versus Expectant Management) trial, the rate of elective 39‐week induction has increased. However, the population impact of this trial on obstetric and maternal outcomes is unknown. This study aims to evaluate trends in severe maternal morbidity (SMM) and obstetrical outcomes among low‐risk term patients following the ARRIVE trial.

**Study Design**: This study is a population‐based, retrospective cohort analysis of U.S. delivery hospitalizations, utilizing data from the National Inpatient Sample database. Deliveries were identified using ICD‐10 codes and categorized into two epochs: Epoch 1, Pre‐ARRIVE (2015 Q4 to 2018 Q2), and Epoch 2, Post‐ARRIVE (2019 Q2 to 2021 Q4), with the dissemination phase 2018 Q3 to 2019 Q1. Patients with medical indications for induction, outlined by the ARRIVE trial, were excluded. SMM was defined according to the CDC with related subgroups. The primary outcome was the difference in risks (aRR, 95% CI) of SMM and obstetrical outcomes as estimated by multivariate logistic regression analyses.

**Results**: Among 8,404,920 deliveries, the rate of 39‐week delivery increased from 52.4% to 57.7% (P< 0.001) among low‐risk patients. The rate of SMM increased (0.31 vs 0.38%, p< 0.001), while cesarean delivery decreased (14.0 vs 13.8%, p = 0.0422) between Epoch 1 and 2. Risk of SMM increased (aRR 1.20, CI 1.12‐1.28), as did SMM subgroups including acute renal failure, respiratory SMM, sepsis, shock, and hemorrhagic SMM (Fig. 1). Eclampsia rates decreased (aRR 0.59, CI 0.49‐0.72), despite an increase in pregnancy‐associated hypertension. There were also reductions in cesarean delivery (aRR 0.97, CI 0.95‐0.98), obstetric anal sphincter injury (aRR 0.96, CI 0.94‐0.98), and hospital length of stay exceeding the 95th% (aRR 0.91, CI 0.87‐0.95) (Fig 2).

**Conclusion**: Following the ARRIVE trial, there has been an increase in SMM and pregnancy‐related hypertension. However, there has been a decrease in the rates of eclampsia, OASIS, and cesarean sections, suggesting a complex potential impact of the ARRIVE trial on outcomes in low‐risk patients.

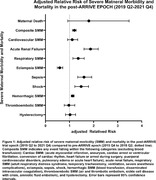

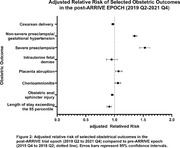

## 962 | Impact of Emergency Room Abortion Bans on Pregnancies with Previable PPROM: A Cost‐Effectiveness Analysis

Amelia H. Gagliuso^1^; Meghana Narahari^2^; Megha Arora^3^; David M. Monroe^3^; Aaron B. Caughey^3^

*^1^Oregon Health & Sciences University, Portland, OR; ^2^Oregon Health and Science University, Portland, OR; ^3^Oregon Health & Science University, Portland, OR*

4:00 PM ‐ 6:00 PM

**Objective**: Preterm premature rupture of membranes (PPROM) is a serious condition that affects 0.4‐0.7% of all pregnancies. Pregnancies with PPROM are associated with increased neonatal morbidity and mortality. Additionally, children surviving PPROM have higher rates of physical disability and neurodevelopmental delay. Under the Emergency Medical Treatment and Labor Act (EMTALA), any patient who presents with an emergency medical condition must be appropriately screened and stabilized, including instances in which treatment entails stabilization through emergency abortion. This analysis evaluated the cost‐effectiveness of emergency abortions for previable PPROM between 18‐22 weeks.

**Study Design**: In this analysis, a TreeAge model was created to compare outcomes in previable PPROM patients with emergency abortion available to those who do not have access to emergency abortion under EMTALA. The cohort consisted of 1,183 individuals, the estimated number of pregnant people with previable PPROM seeking abortion. Clinical outcomes measured in the model included chorioamnionitis, maternal sepsis, maternal death, stillbirth, neonatal death, and neurodevelopmental delay. Probabilities, utilities and costs were derived from literature. QALYs were discounted at a rate of 3%.

**Results**: In this theoretical cohort, abortion access under EMTALA was associated with a decrease of 12 cases of maternal sepsis, 1 case of maternal death, 621 cases of stillbirth, 125 cases of neonatal death, and 95 cases of neurodevelopmental delay. Emergency abortion availability was the dominant strategy as it saved $97,131,562 and led to 1,952 additional QALYs.

**Conclusion**: Restrictive abortion bans that bar emergency abortion as care to treat previable PPROM results in increased maternal morbidity and mortality, as well as increased costs and decreased quality of life.

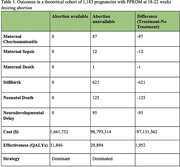

## 963 | Risk Factors Associated with Antiseizure Medication Discontinuation During Pregnancy

Amelie Pham^1^; Andrew D. Wiese^1^; Andrew J. Spieker^1^; Chad You^2^; Ashley A. Leech^1^; Margaret A. Adgent^1^; Carlos G. Grijalva^1^; Sarah S. Osmundson^1^

*^1^Vanderbilt University Medical Center, Nashville, TN; ^2^Vanderbilt University Medical Center, Nasvhille, TN*

4:00 PM ‐ 6:00 PM

**Objective**: Evaluate factors associated with discontinuation of antiseizure medication (ASM) by the first trimester of pregnancy.

**Study Design**: We constructed a retrospective cohort of births (2007‐2019) among Tennessee Medicaid pregnant patients with enrollment from 90 days before conception through delivery. Data included healthcare claims, prescription fills, hospital discharge and birth certificates. We included births ≥ 20 weeks’ gestation among patients aged 15‐44 years on ASM monotherapy. Among patients with filled prescriptions for ASMs before conception or in the first trimester, we defined discontinuers as those with no ASM prescription filled after the first trimester, and continuous users as those who filled prescriptions after the first trimester. We used logistic regression to model the association between clinical factors and ASM discontinuation overall and among patients with an epilepsy diagnosis, adjusting for relevant confounders.

**Results**: Of 5279 pregnant patients with ASM exposure before conception or in the first trimester, 54% discontinued by the first trimester (Table 1). Factors associated with ASM discontinuation included the absence of an epilepsy diagnosis, the presence of a psychiatric or other diagnosis, younger age, nulliparity, earlier study delivery year, living in a rural area, and use of older ASM class (Table 2). While 26% of pregnant patients with ASM use had an epilepsy diagnosis, 35% had no diagnosis traditionally associated with ASM use. Among patients with epilepsy, 29% discontinued ASMs by the first trimester. In this group, older ASM class and earlier study delivery year were associated with higher odds of discontinuation. Non‐Hispanic Black individuals had lower odds of discontinuation than non‐Hispanic White individuals (Table 2).

**Conclusion**: For patients with indications for ASM use, national guidelines recommend continuing most ASMs during pregnancy or discontinuing with clinician guidance. The majority of pregnant patients in our cohort, including those with epilepsy, discontinued use around pregnancy diagnosis.

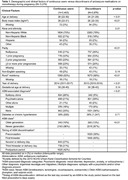

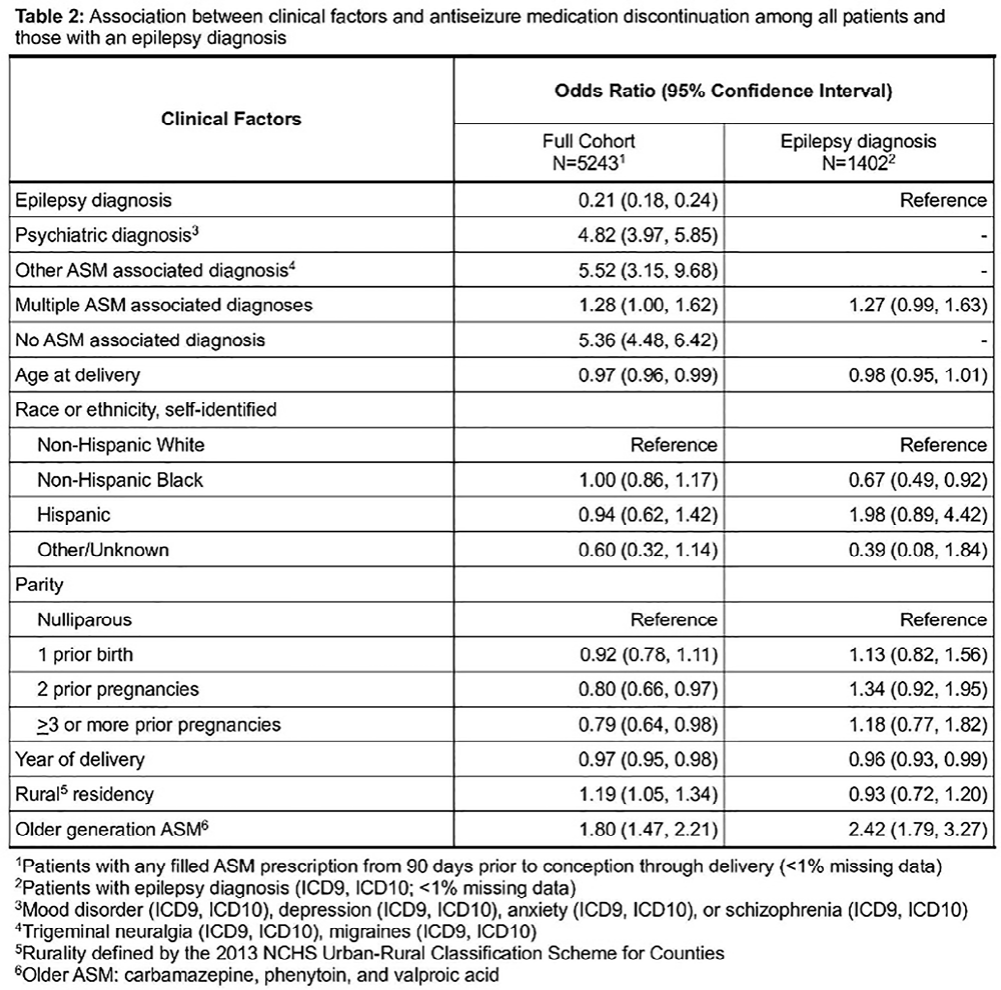

## 964 | Changes in Antiseizure Medication use and Discontinuation During Pregnancy

Amelie Pham^1^; Andrew D. Wiese^1^; Andrew J. Spieker^1^; Chad You^2^; Ashley A. Leech^1^; Margaret A. Adgent^1^; Carlos G. Grijalva^1^; Sarah S. Osmundson^1^

*^1^Vanderbilt University Medical Center, Nashville, TN; ^2^Vanderbilt University Medical Center, Nasvhille, TN*

4:00 PM ‐ 6:00 PM

**Objective**: To evaluate trends in antiseizure medication (ASM) use and discontinuation in pregnancy, by delivery year and medication.

**Study Design**: We constructed a retrospective cohort of births from 2007 to 2019 among pregnant patients enrolled in Tennessee Medicaid with continuous enrollment from 90 days before pregnancy through delivery. Enrollment files were linked to healthcare encounters, prescription fills, hospital discharges, and birth certificate data. We included births ≥ 20 weeks gestation among patients aged 15 to 44 years with any ASM exposure, defined as ≥ 1 prescription fill for a single ASM (monotherapy) between the 90 days before conception through delivery. We evaluate trends in ASM use, including newer (levetiracetam, lamotrigine, oxcarbazepine, zonisamide) and older (carbamazepine, phenytoin, and valproate) ASM class. We further classified our cohort into two subgroups: 1) ASM exposure only from 90 days before conception through the first trimester (discontinued use) and 2) ASM exposure initiated by the first trimester with continued use through the third trimester or delivery (continued use during pregnancy).

**Results**: Among 5,278 pregnant patients exposed to ASM monotherapy, lamotrigine was consistently the most used ASM overall, while levetiracetam use increased over the study period (all users, Figure 1A). Lamotrigine was also the most commonly used medication among subgroup 1 (Figure 1B). By 2013, levetiracetam use increased and remained similar to lamotrigine use among subgroup 2 (Figure 1C). Use of all older ASMs decreased over the study period. Valproate exposure was rare (< 1%) and no valproate use was found in subgroup 2.

**Conclusion**: Starting in 2009, national guidelines recommended continuing most ASMs during pregnancy or discontinuing with clinician guidance based on ASM class, specifically use of valproate, phenytoin, and polytherapy with valproate in pregnancy. This study highlights how practice patterns are slowly changing to reflect the predominance of newer ASM use, but that early pregnancy discontinuation remains common.

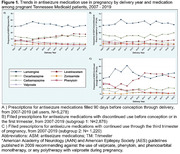

## 965 | The Effect of Antibiotic Treatment in Late Preterm Premature Rupture of Membranes on pregnancy Outcomes

Anat Pardo^1^; Raud Qassim^2^; Michal Axelrod^3^; Shiri Barabash Hazan^4^; Noam Pardo^5^; Ohad Houri^6^; Anat Shmueli^4^; Eran Hadar^6^

*^1^The Helen Schneider Hospital for Women at Rabin Medical Center, Petach Tikwa, HaMerkaz; ^2^Helen Schneider Hospital for Women, Rabin Medical Center, Petach‐Tikva, Petach‐Tikva, HaMerkaz; ^3^Department of Obstetrics and Gynecology, Sheba Medical Center, Tel HaShomer, Ramat Gan, HaMerkaz; ^4^Helen Schneider Hospital for Women, Rabin Medical Center, petach tikwa, HaMerkaz; ^5^Maternal Fetal Medicine Division, Sheba Medical Center, Tel Hashomer, Sheba Medical Center/Ramat Gan, HaMerkaz; ^6^Rabin Medical Center, Petach Tikva, HaMerkaz*

4:00 PM ‐ 6:00 PM

**Objective**: To Compare pregnancy outcomes in women with late preterm premature rupture of membranes (PPROM) between 34+0 and 36+6 weeks, treated with antibiotics versus those not receiving antibiotics, to assess its efficacy in the late preterm period

**Study Design**: Retrospective cohort study at a single tertiary center between 2020 and 2023, included 228 women who presented with late PPROM. The outcomes of 95 treated with antibiotics were compared to 133 untreated with antibiotics. Primary outcomes were duration of latency period, chorioamnionitis and neonatal sepsis. Secondary outcomes included maternal complications (Post partum hemorrhage, Fever, Endometritis, Operative deliveries) and neonatal complications (5‐min Apgar< 7, umbilical cord pH< 7.1, and neonatal intensive care unit admission). The groups were compared using demographic and clinical independent variables in a univariate and multivariate analysis

**Results**: Baseline characteristics did not significantly differ between the groups. In a univariate analysis the group treated with antibiotics demonstrated longer latency period in comparison to the non‐treated group (2 vs.1 day, p< 0.001). Despite an earlier gestational age (GA) at PPROM (p = 0.01) in the treated group, no difference was observed at gestational age at delivery. No difference in chorioamnionitis or neonatal sepsis rate was seen between the groups, however the group treated with antibiotic demonstrated less positive placental cultures compared to untreated women (p = 0.04). No other significant adverse maternal or neonatal outcome was significantly different. Same pattern of longer latency but without significant difference in delivery GA was observed in a sub analysis of women who progressed to active labor spontaneously (p = 0.005). A multivariate analysis of the latter which included parity, GBS status, GA and cervical dilatation at diagnosis failed to show that antibiotic treatment prolonged latency (aOR = 0.70, 95% CI 0.27‐1.79, p = 0.46)

**Conclusion**: Antibiotic treatment in the management of late PPROM did not prolong latency period nor influenced maternal and neonatal outcomes

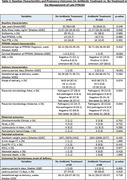

## 966 | Equitable Implementation of Obstetric Life Support in Maternity Care Deserts and Low Access Areas

Andrea D. Shields^1^; Rogie Royce Carandang^2^; Andy Lowe^3^; Ann Marie Day^4^; Thomas Trimarco^5^; Les R. Becker^6^; Vincent N. Mosesso, Jr.^7^; Shayna Cunningham^8^

*^1^University of Connecticut Health, Avon, CT; ^2^University of Connecticut Health, University of Connecticut Health, CT; ^3^New England Rural Health Association, New England Rural Health Association, VT; ^4^New England Rural Health Association, Barre, VT; ^5^Dartmouth Health, Dartmouth CREST, NH; ^6^MedStar Health, Rockville, MD; ^7^University of Pittsburgh, Pittsburgh, PA; ^8^University of Connecticut School of Medicine, Farmington, CT*

4:00 PM ‐ 6:00 PM

**Objective**: Obstetric Life Support (OBLS) is an evidence‐based, interdisciplinary simulation‐based curriculum to train prehospital and hospital‐based healthcare workers (HCWs) on prevention, recognition, and management of maternal medical emergencies. We aimed to identify barriers and facilitators to implementing OBLS in rural and low‐resource settings to inform adaptations for optimizing medical emergency education in these contexts.

**Study Design**: We conducted a nationwide survey (n = 122) of administrators and HCWs in rural settings, exploring Consolidated Framework for Implementation Research (CFIR) elements that may influence implementation outcomes across multiple levels. Focus groups were conducted with a subset of 15 participants to discuss strategies for promoting OBLS adoption, implementation, and sustainability.

**Results**: Both prehospital and hospital‐based participants found the OBLS learning objectives and content highly appropriate and acceptable but expressed concerns regarding the feasibility of implementation in their work contexts. Salient CFIR domains identified included: 1) Intervention Characteristics: Evidence strength and quality, relative advantage, design quality and packaging, and cost; 2) Inner Setting: Implementation climate and readiness for implementation; 3) Outer Setting: Patient needs and resources, and external policy and incentives.

Resource constraints were perceived to be a significant barrier, especially time, staffing, space, and equipment. Suggested adaptations included the development of eLearning course modules, a shortened in‐person component, a web‐based megacode evaluation tool, and an implementation toolkit to facilitate planning, progress monitoring, and change management. Participants also highlighted the importance of local champions to drive the implementation process and endorsed a train‐the‐trainer approach to build local capacity and ensure sustainability.

**Conclusion**: Equitable scale‐up of OBLS requires an implementation plan that considers the training program's context, delivery mechanisms, and resource requirements.

## 967 | Race and Insurance Disparities in Partner Screening for Autosomal Recessive Conditions During Pregnancy

Ajitha Chivukula^1^; Makayla Murphy^2^; Brittany Gancarz^2^; Andrea D. Shields^3^

*^1^University of Connecticut School of Medicine, University of Connecticut School of Medicine, CT; ^2^University of Connecticut Health, University of Connecticut Health, CT; ^3^University of Connecticut Health, Avon, CT*

4:00 PM ‐ 6:00 PM

**Objective**: We aim to identify if race and insurance statuses of pregnant people and their partners impact decisions to undergo genetic carrier screening.

**Study Design**: Retrospective chart review was conducted on pregnant people receiving care at tertiary academic medical center from 6/1/2021‐5/31/2024. Inclusion criteria were pregnant people (18+) with singleton pregnancies who tested positive for autosomal recessive conditions in 1^st^ or 2^nd ^trimesters and had genetic counseling. The primary outcome was comparison of uptake rates for genetic screening in partners of pregnant people with abnormal carrier screens by race and insurance status. Continuous variables were summarized with mean and standard deviations. Categorical variables were analyzed by Pearson's Chi square test, summarized with % and frequencies.

**Results**: Out of 62 pregnant people with abnormal genetic screens, 48% of partners opted for a genetic screen. Table 1 shows race and insurance statuses for pregnant people and partners. There was significant difference in racial makeup of patients (p< 0.0001) and partners (p< 0.0001) comparing tested and declined groups. There was no significant difference between insurance types of partners who tested vs. did not (p = 0.422). More pregnant people identified as Hispanic/Latino (31%) and had Medicaid (63%), while more partners identified as White (31%) and had private insurance (42%). Couples with matching insurance were more likely to have both partners test vs. those with nonmatching insurance (p = 0.0001). Couples with Medicaid had highest proportion of tested partners (77%). While there was no association between the insurance type of pregnant patients and whether their partners tested (p = 0.306), there was an association between whether their partners tested and whether they were insured (p< 0.0001).

**Conclusion**: Our results suggest a correlation between partner demographics and insurance and uptake rates of partner carrier testing. This shows disparities in uptake rates of genetic carrier screening, suggesting that SES factors may impact the decision for partner to test even with genetic testing access.

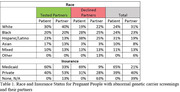

## 968 | Simulation Training to Improve Dilation and Evacuation Counseling in a Restrictive State

Anjali Nambiar^1^; Courtney C. Baker^1^; R. Nicholas Burns^2^

*^1^UT Southwestern Medical Center, Dallas, TX; ^2^University of Texas Southwestern, Dallas, TX*

4:00 PM ‐ 6:00 PM

**Objective**: Following restrictive abortion legislation in Texas, OB/GYN residents have reduced exposure to Dilation and Evacuation (D&E). To address this training gap, a simulation and quality improvement initiative was implemented to increase resident familiarity with D&E and improve options counseling for patients with intrauterine fetal demise (IUFD).

**Study Design**: Residents at a single teaching institution participated in a monthly simulation activity, that included a pre‐assessment of perceived barriers to D&E, a didactic video reviewing perioperative considerations and D&E steps, and a case‐guided D&E simulation on a low‐fidelity uterine model. The rate of complete options counseling (D&E vs medical induction) documented by residents in the electronic medical record and rate of D&E performed for patients with 2nd trimester IUFD (< 22 weeks gestation) was assessed before (9/2023‐12/2023) and after (3/2024‐6/2024) simulation implementation. Statistical significance was assessed via chi‐squared test.

**Results**: 42 residents participated in the activity. On pre‐assessment, most residents (76%) reported “rarely” or “never” discussing D&E with patients. “I do not know how to perform a D&E,” “I do not have available staff to supervise me during D&E,” and “I do not know how to counsel patients regarding D&E” were the most perceived barriers to D&E, identified by 71%, 67%, and 50% of residents respectively. In the pre‐simulation period, 9/19 (47%) patients with IUFD had complete options counseling, and 3/9 (33%) elected for D&E. In the post‐implementation period, 24/26 (92%, p = 0.001) had complete options counseling and 8/24 (33%) chose D&E.

**Conclusion**: Documentation of complete options counseling was significantly improved after initiative implementation. Despite perceived barriers to D&E, residents counseled patients more consistently and performed more D&Es after simulation training. Rate of patient desire for D&E was unchanged. Low‐fidelity D&E simulation may be an effective quality improvement initiative to address resident education gaps and improve options counseling for patients that are candidates for D&E.

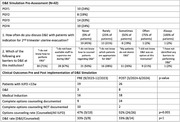

## 969 | Black Birthing persons’ Perspectives on Factors Leading to Medical Mistrust in Obstetric Settings

Anna Palatnik^1^; Erica Marion^1^; Jessica Olson^1^; Dalvery Blackwell^2^; Joni Williams^1^; Katherine Quinn^1^

*^1^Medical College of Wisconsin, Milwaukee, WI; ^2^African American Breastfeeding Network Inc., Milwaukee, WI*

4:00 PM ‐ 6:00 PM

**Objective**: To examine factors leading to medical mistrust among Black birthing persons.

**Study Design**: In this prospective qualitative study, 30 Black birthing people participated in semi‐structured interviews to elicit their perspectives on factors leading to medical mistrust during their obstetric care. Purposive sampling was used to ensure a cohort diverse in terms of obstetric experiences such as parity, current mode of delivery, and with or without doula support in current pregnancy. Interviews were recorded, transcribed, and coded twice by two different team members in MAXQDA2022. Data were analyzed using thematic analysis to identify patterns and develop and refine themes.

**Results**: The average age of participants was 28 (range 19‐41 years of age); 13% were married, 27% had a high school diploma, 40% were unemployed, 40% had an income of $20‐000‐$40,000, 77% had public insurance, and 27% had used doula services for the most recent pregnancy. Majority of participants were multiparous (70%), delivered vaginally (53%) and interviewed within 6 weeks postpartum. Five themes contributing to medical mistrust were identified: 1) lack of sufficient education provided to birthing people around possible obstetric  complications 2) not feeling heard by the medical team due to lack of time and feeling rushed at prenatal appointments, 3) overuse of the medical terminology and jargon by the medical team, 4) discrepancies in expectations regarding birthing experiences in the hospital, and 5) inadequate support and reassurance after traumatic pregnancy or birth experience.

**Conclusion**: Perspectives gathered in this analysis identify inadequate communication, and insufficient time and knowledge for shared decision making as key factors leading to medical mistrust among Black birthing persons.

## 970 | Detection of Individual Bile Acids by Sebum Sampling in Cholestasis of Pregnancy

Anne M. Ambia^1^; Jeffrey McDonald^2^; Yevgenia Y. Fomina^1^; Andrea Rizkallah^2^; C. Edward Wells^3^; Catherine Y. Spong^3^; Donald D. McIntire^3^; David B. Nelson^3^

*^1^University of Texas Southwestern, Dallas, TX; ^2^UT Southwestern, Dallas, TX; ^3^University of Texas Southwestern Medical Center, Dallas, TX*

4:00 PM ‐ 6:00 PM

**Objective**: Intrahepatic cholestasis of pregnancy (ICP) is characterized by elevated bile acids and pruritis of palms as a disease unique to pregnancy. There is a paucity of information on the individual bile acids that contribute to palmar pruritis in ICP. We aimed to perform a feasibility study to utilize a novel methodology of extraction of steroids through sebum sampling in those undergoing evaluation for ICP.

**Study Design**: This was a prospective, observational cohort study of pregnant individuals evaluated for ICP from December 2023 to July 2024 a single academic center. Patients were characterized into pruritis of pregnancy (total BA < 10 mmol/L, referent), ICP (BA ≥10 mmol/L), using a quantitative enzymatic assay. At the time of initial evaluation for ICP, sebum sampling was performed after preparation of the skin to be sampled with an alcohol wipe cleanse. Three‐five strips of sebutape were placed for 15 minutes and removed. Extraction of bile acids were performed through liquid chromatography mass spectrometry methods.

**Results**: There were 5 patients with cholestasis of pregnancy and 2 patients with pruritis of pregnancy whose samples were analyzed. There were no significant demographic differences between those with and without cholestasis of pregnancy. Gestational age at sebum sampling did not differ between the groups 35.9±0.9 vs 36.0±2.4, P = 0.97. The mean total bile acids of those with cholestasis were 52.6±19.1. Corresponding analysis of serum samples from these cholestasis patients detected taurocholic acid as the main analyte. Levels of taurocholic acid, glycolcholic acid, taurochenodeoxycholic acid, cholic acid, glycol‐chenodeoxycholic acid, and glycol‐deoxycholic acid were detected in both upper back and palmar samples.

**Conclusion**: Beyond serum sampling of total bile acids, we have demonstrated a novel methodology of detection of bile acid levels in sebum samples in patients with cholestasis of pregnancy. This may offer further insight into disease characteristics and symptomatology.

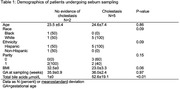

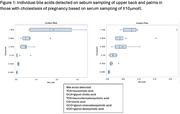

## 971 | Rates of Cesarean‐Delivery and Perinatal Outcomes in OA Verses OP Position by Birth‐Weight in Nulliparous

Annika Willy^1^; Bharti Garg^2^; Aaron B. Caughey^2^

*^1^Oregon Health & Science University, Bend, OR; ^2^Oregon Health & Science University, Portland, OR*

4:00 PM ‐ 6:00 PM

**Objective**: We aimed to evaluate how birth weight affects cesarean delivery rates and delivery outcomes for fetuses in occiput‐anterior (OA) versus occiput‐posterior (OP) positions. Specifically, we investigated whether the impact of fetal position on cesarean delivery varies with different birth weight categories and how it influences neonatal outcomes in nulliparous patients.

**Study Design**: This was a retrospective cohort study of singleton, non‐anomalous, term deliveries with vertex presentation in nulliparous individuals of California (2008‐2020). Birth weight into eight categories: < 3500g, 3500‐3749g, 3750g‐3999g, 4000‐4249g, 4250‐4499g, 4500‐4749g, 4750‐4999g, ≥5000g. Multivariable Poisson regression model was used to examine the association of OP position and cesarean delivery along with outcomes (shoulder dystocia and scalp injury) for each weight category. Adjusted risk ratios (aRR) with 95% CI were estimated.

**Results**: In this study, we included 1,781,036 births of which 1.5% (n = 26,943) were in OP position. Cesarean delivery rates were higher in OP  presented neonates in all birthweight categories, such as in < 3500 grams (77.6% vs 21.1%; aRR = 3.50; 95% CI: 3.46‐3.54), and ≥5000g (100% vs. 76.2%; aRR = 1.29 (1.22‐1.38)). Risk of shoulder dystocia was found to be increased in all most all birth weight categories in OP neonates, such as< 3500 g (0.92% vs 0.59%; aRR = 1.51 (1.05‐2.19)) and 3750‐3999 g (6.25% vs 3.69%;aRR = 1.72(1.23‐2.39)). Risk of scalp injuries were found to be higher in OP neonates in birth weight categories such as < 3500 g (12.06 vs 4.69 aRR = 2.53 (2.30‐2.78)).

**Conclusion**: We found that OP fetuses had a higher rate of cesarean delivery in all weight categories compared to OA. The difference between OA and OP cesarean delivery rates decreased as birth weight increased. In regards to perinatal outcomes we found OP neonates in most weight categories had higher rates of shoulder dystocia. Additionally scalp injury was increased in OP neonates compared to OA neonates.

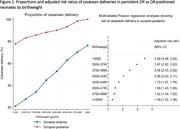

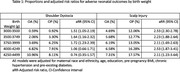

## 972 | Preterm Birth in Patients with Fontan Circulation: Looking to the Placenta for Answers

Ashley M. Hesson; Caroline Simon; Angela Quain; Michael R. Joynt; Elizabeth S. Langen

*University of Michigan, Ann Arbor, MI*

4:00 PM ‐ 6:00 PM

**Objective**: Pregnant patients with Fontan circulation have relative hypoxemia with limited ability to augment cardiac output. Preterm birth (PTB) and fetal growth restriction (FGR) are common complications. We integrate clinical, histopathologic, and spatial transcriptomic data to characterize PTB with and without FGR in this population.

**Study Design**: 17 live births in 15 patients with Fontan circulation were identified and Cardio‐obstetric variables were abstracted from the medical record. Placentas were analyzed by a pathologist. RNA was extracted from 6 preterm (3 normally grown, 3 FGR), formalin fixed, paraffin embedded samples with representative findings for spatial transcriptomics. Samples were sequenced with Visium CytAssist and analyzed in Loupe Browser/R.

**Results**: Mean pre‐pregnancy SpO2 was 93.3±3.5%, where SpO2 at delivery decreased with increasing gestational age (GA, R = ‐0.48, P = 0.046). Over 1/3 (41.2%, N = 7/17) had FGR and most (82.4%, N = 14/17) had PTBs (mean GA 233.1±24.9 days [33 weeks]). Of PTBs, 64.2% (N = 9/14) labored spontaneously. 64.7% of the placentas (N = 11/17) showed accelerated maturation for GA (Fig 1). Spatial capture spots clustered based on clinical and histopathologic features, contrasting samples from FGR vs normally grown placentas and regions of frank vs minimal pathology (Fig 2). Spots with frank pathology showed upregulation of hypoxia pathways with peri‐lesion upregulation of pro‐fibrotic transcripts; structural integrity transcripts (PLEC, SPTAN1, SRGN) were downregulated. Regions without frank pathology in FGR placentas also overexpressed pro‐fibrotic markers and markers of placental dysfunction (PSG11, ALPP). Adverse pregnancy outcome markers (PAEP, PAPPA2) were upregulated in early PTB (< 34 weeks) compared to late PTB; PLEC, SPTAN1, SRGN were downregulated.

**Conclusion**: We identify a transcriptomic profile of consistently downregulated structural genes that unify placentas from early PTB with histopathologic placental findings in patients with Fontan circulation. These markers should be explored as potential mediators of PTB in cases of significant maternal cardiac disease.

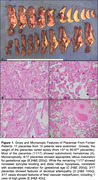

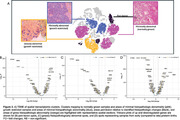

## 973 | Uterine Inflammatory Characteristics Following Cesarean Delivery

Aya Mohr‐Sasson^1^; Tal Dadon^2^; David Stockheim^3^; Jigal Haas^3^; Adva Aizer^3^; Roy Mashiach^3^; Raoul Orvieto^3^

*^1^Division of Minimally Invasive Gynecological Surgery, Department of Obstetrics, Gynecology and Reproductive Sciences, UTHealth McGovern Medical School, Houston, TX; ^2^Sheba Medical Center, Ramat Gan, Tel Aviv; ^3^Sheba Medical Center, Ramat‐Gan, Tel Aviv*

4:00 PM ‐ 6:00 PM

**Objective**: An incomplete healed scar (niche) is a long‐term complication of cesarean delivery (CD), and is associated with symptoms including subfertility. One theory refers to inflammatory process at the area of the niche that harms the endometrial environment, however, data is limited. The aim of this study is to compare the inflammatory characteristics of women with cesarean uterine scar to those without.

**Study Design**: This is a prospective study including all women visiting hysteroscopy ambulatory clinics for diagnostic hysteroscopy. Study population included women with cesarean uterine scar (study group that were compared to those without (controls). A syringe was attached to the outlet of the diagnostic hysteroscope and the first 5 cc of 0.9% normal saline fluid used for hydro dissection were collected. The samples were analyzed for inflammatory factors levels, including: GM‐CSF, IFN‐gamma, IL‐1, IL‐2, IL‐5, IL‐6, IL‐7. IL‐13, IL‐15, IL‐17, IL‐17F, IL‐22, IL‐23, IL‐31, IL‐36 and TNF‐alpha. Primary outcome was defined as the difference in inflammatory factors level.

**Results**: 80 women met inclusion criteria, of them 29(36%) had history of  CD and 51(64%) had no uterine scar. No difference was found in the level of any of the 17 factors collected. Analysis by indication to perform the procedure, comparing infertility to all other indications, revealed significantly higher IL33 levels in the infertility group [25.60(2.80‐185.83) vs. 5.98((0‐43.84pg/ml; p = 0.02]. While comparing the 9 women with infertility to the 20 women without infertility in the CD group, no difference was found in uterine scar characteristics, however, IL 33 was found to be 25 times higher in the infertility group [61.23(19.84‐169.44) vs. 4.61(0‐33.72) pg/ml;p = 0.004]. This difference was not demonstrated in women without CD (p = 0.29).

**Conclusion**: Women undergoing evaluation due to infertility with history of uterine scar have significantly higher level of inflammatory marker IL33. This novel finding might be grounds for future treatment for this population.

## 974 | Robotic‐Assisted Abdominal Cerclage: A Single‐Center Journey

Asha Bhalwal; Elio Tahan; Aya Mohr‐Sasson

*Division of Minimally Invasive Gynecological Surgery, Department of Obstetrics, Gynecology and Reproductive Sciences, UTHealth McGovern Medical School, Houston, TX*

4:00 PM ‐ 6:00 PM

**Objective**: Transabdominal cerclage (TAC) is currently considered following unsuccessful transvaginal cerclage. The placement of the cerclage can serve for all future pregnancies as it is not removed once it was placed, and following its placement, women are obligated to deliver by Cesarean delivery. Increasing evidence suggests improved neonatal survival rates following abdominal cerclage compared with repeat vaginal cerclage. The aim of this study is to present patient's characteristics and clinical outcomes of women following robotic assisted abdominal cerclage placed for history indicated cervical insufficiency

**Study Design**: This is a retrospective study conducted at a single tertiary medical center. All women following robotic assisted abdominal cerclage placement between November 2020 to February 2024 were included in the study. Data were collected from women's' medical files. Primary outcome was defined as the rate of delivery ≥32 weeks of gestation.

**Results**: 16 women underwent robotic assisted abdominal cerclage during the study period, of them 6 (37.5%) were pregnant during the procedure. Women's mean age and body mass index were 34.4(±4.4SD) years‐old and 35.7(±6.9SD) kg/m^2^, respectively. The mean gravida was 4(±1.8SD) with parity of 1(±1.0SD). 60% of the women had history of previous vaginal cerclage (n = 9). The mean gestational age of previous miscarriages was 21.5 (±4.6SD). Mean surgical duration was 136 (±40) minutes with minimal blood loss [mean: 50(±40SD) cc]. Two of the procedures performed during pregnancy were converted, one to open approach as the patient did not tolerate Trendelenburg and one to vaginal approach due substantial pelvic adhesions. Four of the seven women (70% of the non‐pregnant) attempted to conceive, got pregnant with an average time of 3.1(±3.3SD) months. Six of the women included in the study (36%) had already given birth with an average gestational age of 34.7(±3.4SD).

**Conclusion**: Robotic assisted abdominal cerclage is a feasible option for the treatment of cervical insufficiency with low complication rate.

## 975 | Epigenetic Age Acceleration and Blood Pressure in Pregnancy: Findings from the CHAP Study

Bertha A. Hidalgo; Amit Patki; Hemant K Tiwari; Marguerite Ryan Irvin; On behalf of the CHAP Consortium

*University of Alabama at Birmingham, Birmingham, AL*

4:00 PM ‐ 6:00 PM

**Objective**: Elevated blood pressure (BP) during pregnancy can have cardiovascular health consequences for the mother in the short and long term. Measures of biological age including DNA methylation age (DNAmAge) and derived epigenetic age acceleration (EAA) have been associated with cardiovascular traits. However, few studies have examined if epigenetic age measures are associated with BP during pregnancy and PreE.

**Study Design**: We performed a secondary analysis of the Chronic Hypertension and Pregnancy (CHAP) study of n = 2408 individuals with mild hypertension randomized to standard care versus treatment. We measured DNAmAge from blood samples drawn between 6‐23 weeks gestation in n = 1310 from the trial. DNAmAge was measured using 4 calculators: 1) Horvath, 2) Hannum, 3) skin and blood, and 4) PhenoAge. We analyzed correlations between the DNAmAge clocks and chronological age, as well as associations between EAA and five BP variables as outcomes 1) PreE; 2) systolic and diastolic BP (SBP/DBP) at baseline (6‐23 weeks), 3) visit 2 SBP/DBP (23‐34 weeks), 4) delivery SBP/DBP, and 5) 6‐weeks post‐partum (PP) SBP/DBP). Regression models (linear for BP and logistic for PreE) were adjusted for ancestry and smoking during pregnancy.

**Results**: The study population was 50% Black, 38% White and 12% other, and the average age was 32.3 ± 5.7 years. DNAmAge was highly correlated with chronological age for each of the 4 calculators. EAA from the skin and blood clock was associated with baseline SBP (p = 0.006), baseline DBP (p = 0.029), and DBP PP (p = 0.029). No EAA estimate was statistically significantly associated with PreE or BP at delivery. See Table 1 for the association of EAA with baseline SBP, DBP and PreE.

**Conclusion**: There was a cross‐sectional association between two EAA measures with early pregnancy BP, but not BP later in pregnancy or PreE. Other epigenetic variables and/or estimates from assays measured at additional time points may give additional insight into the relationship between biological aging and BP during pregnancy in women with chronic hypertension.

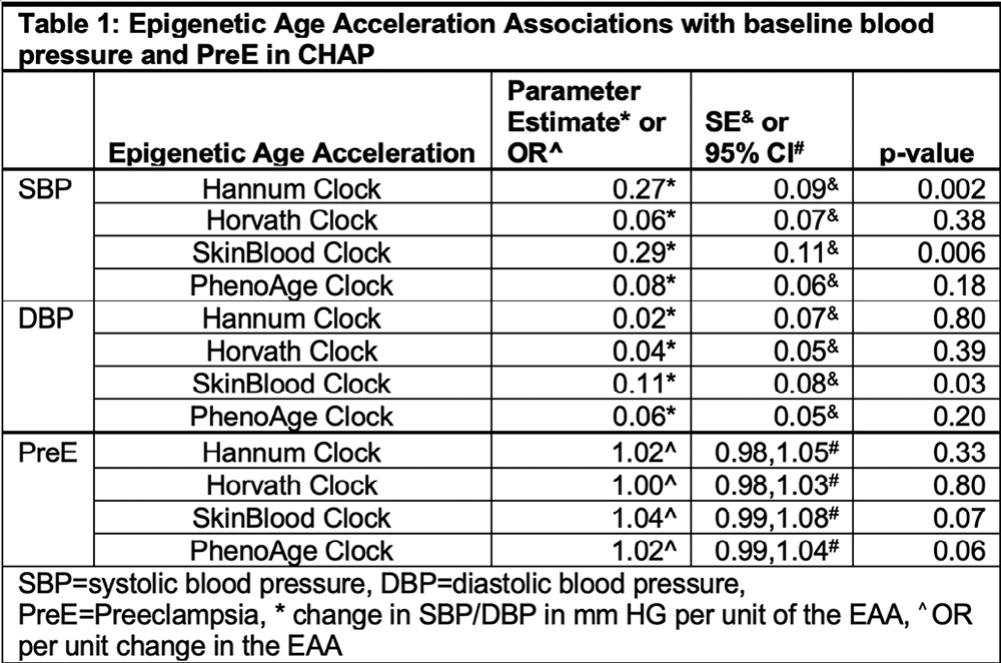

## 976 | National Prevalence of Healthcare Provider‐Recommended Bedrest and Activity Restriction by Pregnancy Condition

Maria A. Fernandez^1^; Ellie C. Mayers^2^; Gladys E. Smith^2^; Ellen C. Caniglia^2^; Stefanie N. Hinkle^3^; Sunni Mumford^3^; Enrique Schisterman^2^; Lisa D. Levine^3^; Beth L. Pineles^2^

*^1^Perelman School of Medicine, University of Pennsylvania, Boston, MA; ^2^Perelman School of Medicine, University of Pennsylvania, Philadelphia, PA; ^3^University of Pennsylvania Perelman School of Medicine, Philadelphia, PA*

4:00 PM ‐ 6:00 PM

**Objective**: Bedrest and activity restriction (BAR) are often recommended in pregnancy despite an association with worse pregnancy outcomes. With guidelines recommending against BAR, some consider it an issue of the past, yet the contemporary national prevalence remains unknown. We aimed to determine the contemporary national prevalence of healthcare provider‐recommended BAR among pregnant individuals, the pregnancy conditions associated with these recommendations, and adherence to BAR.

**Study Design**: An internet‐based survey was distributed to females aged 18‐54 who were pregnant or had been pregnant in the past year. The survey was tested and validated. Participants were asked, “Has anyone recommended bedrest or advised you to do less physical activity?,” in addition to details about these recommendations, whether their pregnancy was considered, “high‐risk,” and their pregnancy conditions. The primary outcome was a recommendation for BAR by an obstetrician‐gynecologist or midwife. To account for differential response rates, weighting for age and race/ethnicity was performed based on the target population of all births in the 2022 U.S. Natality Database, and all results presented are weighted. Two‐sided p< 0.05 was considered statistically significant.

**Results**: 1500 survey responses were obtained. After weighting, 539 (38%) participants reported high‐risk pregnancies. The frequency of BAR was 40% (95% confidence interval [CI] 37‐43%); greater in high‐risk pregnancies [61% (95% CI 57‐65%)] than low‐risk pregnancies [27% (95% CI 24‐30%)], p‐value< 0.01. Frequency of recommendations and recommendation type varied by pregnancy condition (chi‐squared p < 0.01, Table). Adherence to BAR varied by both pregnancy condition and restriction type, with most participants across groups adherent (p values < 0.01, Figure).

**Conclusion**: Despite clear national guidelines recommending against BAR, recommendations for BAR practices remain prevalent, especially among high‐risk pregnancies but common even among low‐risk pregnancies. Future studies should focus on the deimplementation of this prevalent and harmful practice.

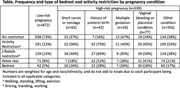

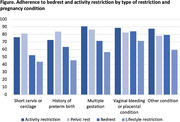

## 977 | Risk Factors for Failing Aspirin Prophylaxis for Preeclampsia Prevention: A Prospective Cohort Secondary Analysis

Bhaskari Burra^1^; Danetta Green^1^; Traci Carson^2^; Lisa M. Foglia^2^

*^1^Mountain Area Health Education Center, Asheville, NC; ^2^Mountain Area Health Education Center (MAHEC), Asheville, NC*

4:00 PM ‐ 6:00 PM

**Objective**: We aimed to identify demographic characteristics and risk factors associated with failure of aspirin (ASA) prophylaxis in a nulliparous population with singleton pregnancies.

**Study Design**: We conducted a secondary analysis of the prospective NuMom2B cohort of 9289 nulliparous women. We included participants with a gestational age of 20 weeks’ gestation or greater. Our sample included 8741 women. The exposure of interest was ASA. The primary outcome was the development of any hypertensive disorder of pregnancy. We compared demographic factors among women who did and did not use aspirin during their pregnancy and outcomes by group.

**Results**: Patients who used aspirin during pregnancy (n = 117) were older (31 vs. 27, p< 0.001), more likely to be White (87% vs. 65%, p< 0.001), non‐Hispanic (87% vs. 73%, p< 0.001) and more likely to have chronic hypertension (HTN) (9% vs 3%, p< 0.001), compared to non‐aspirin users (n = 8624). ASA users were more likely to develop preeclampsia (11% vs 6%, p< 0.05), versus the non‐aspirin group. There were no notable differences in risk factors between ASA users and non‐ASA users for development of hypertensive disorders. There was no significant difference in the gestational age at delivery between the two groups.

Within the ASA group, women who developed hypertensive disorders had a lower mean BMI (24.4 vs 27.4, p< 0.05) and were slightly older (33 vs 31, p = 0.15) compared to those who did not develop hypertensive disorders.

**Conclusion**: In this study, we found that there is no difference in risk factors for the development of hypertensive disorders between ASA and non‐ASA users. We did find a higher incidence of hypertensive disorders in the ASA group. This is possibly due to our relatively small sample size of patients taking ASA, but also could represent a higher apriori risk in this group. The similar gestational ages at delivery amongst ASA and non‐ASA also supports the theory that ASA use prevents the onset of hypertensive disorders of pregnancy by delaying the “metabolic clock” of placental maturation during pregnancy.

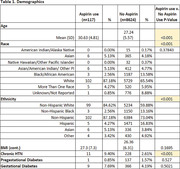

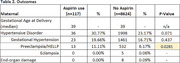

## 979 | Differential Protein Expression Associated with Persistent Hypertension Following a Hypertensive Disorder of Pregnancy

Brian Kleiboeker^1^; Janet Catov^2^; Koen Raedschelders^3^; Aleksandra Binek^3^; Simion Kreimer^3^; C. Noel Bairey Merz^4^; Jennifer Van Eyk^3^; Arun Jeyabalan^1^; Adam C. Straub^5^; Agnes Koczo^1^; Alisse Hauspurg^6^

*^1^University of Pittsburgh School of Medicine, Pittsburgh, PA; ^2^Magee‐Womens Hospital, Pittsburgh, PA; ^3^Cedars Sinai Medical Center, Cedars‐Sinai, CA; ^4^Cedars‐Sinai Medical Center, Los Angeles, CA; ^5^University of Pittsburgh, Pittsburgh, PA; ^6^Alpert Medical School of Brown University, Providence, RI*

4:00 PM ‐ 6:00 PM

**Objective**: Hypertensive disorders of pregnancy (HDP) are associated with future cardiovascular risk, however, the underlying mechanisms are unclear. We sought to identify differentially expressed proteins in individuals who developed chronic hypertension (HTN) in the first year postpartum following a HDP compared to those who remained normotensive.

**Study Design**: We used data from a randomized clinical trial evaluating lifestyle intervention and home blood pressure (BP) monitoring of individuals with pre‐pregnancy body mass index (BMI) ≥25 kg/m^2^ and a new‐onset HDP. The primary outcome was feasibility and no differences were seen in BP between arms, thus randomization groups were combined. BP was measured at remote research visits at 6 weeks and 1 year postpartum and blood microsamples were collected at the second visit. Mass‐spectrometry was used to quantify 381 proteins, from which differential expression and pathway enrichment analyses were performed to detect alterations with persistent HTN (BP ≥140/90 mmHg). All analyses controlled for BMI, tobacco use, and the social construct of race.

**Results**: Of the 100 randomized individuals, 88 (88%) completed sample collection with 85 yielding usable proteomics data. 4 proteins were differentially expressed in individuals with persistent HTN. Stage 2 HTN was associated with increased levels of Complement C4‐B (C4B) and inter‐alpha‐trypsin inhibitor heavy chain 4 (ITIH4) and decreased levels of Afamin (AFM) and the smooth muscle protein myosin light chain 6 (MYL6) compared with Stage 1 HTN or normalization of BP. Pathway enrichment analysis suggests a shared function of endopeptidase inhibitor activity (such as Neprilysin, which degrades natriuretic peptides) in upregulated proteins and ubiquitin‐proteasome mediated proteolysis activity in downregulated proteins.

**Conclusion**: Home microsampling is a promising methodology for postpartum sample collection. Serum proteomics using this approach suggest that protein homeostasis may be dysregulated in persistent HTN following HDP and provides novel candidates for future interventions to reduce progression to HTN following HDP.

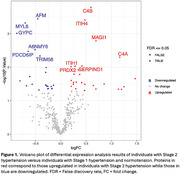

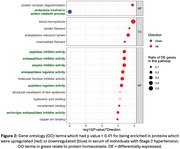

## 980 | Comparison of Various Community Level Social Indices and their Associations with Adverse Pregnancy Outcomes

Bridget C. Huysman^1^; Loren Adler^2^; Sarah Cohen^1^; Candice Woolfolk^3^; Sarah K. England^4^; Antonina I. Frolova^5^; Jeannie C. Kelly^6^; Nandini Raghuraman^6^; Peinan Zhao^4^

*^1^Washington University in St. Louis, St. Louis, MO; ^2^Washington University in St. Louis/Barnes‐Jewish Hospital, St. Louis, MO; ^3^Washington University School of Medicine in Saint Louis, St. Louis, MO; ^4^Washington University, St. Louis, MO; ^5^Washington University School of Medicine, St. Louis, MO; ^6^Washington University School of Medicine in St. Louis, St. Louis, MO*

4:00 PM ‐ 6:00 PM

**Objective**: The social vulnerability (SVI), maternal vulnerability (MVI), and area deprivation (ADI) indices measure community‐level resilience to stressors, vulnerability to adverse maternal health outcomes, and socioeconomic disadvantage, respectively. Prior work has shown that ADI, SVI, and MVI can identify those at risk for preterm birth. The objective of this study was to compare the three indices and their associations with adverse pregnancy outcomes.

**Study Design**: This is a nested case control study within a prospective cohort study of singleton pregnancies from 2017 to 2020. The parent study excluded non‐English speaking patients and IVF pregnancies. The primary outcome for this analysis was composite adverse obstetric outcomes (Table 1). These outcomes were compared between patients with low SVI, MVI, and ADI (< 75^th^ percentile, representing higher resilience/less vulnerable/prosperity) versus high SVI, MVI, and ADI (≥75^th^ percentile, representing lower resilience/more vulnerable/deprivation) and stratified by race.

**Results**: Among 1260 birthing people, patients with high SVI and ADI were more likely to have composite obstetric adverse outcomes compared to those with lower SVI and ADI, respectively (aOR 1.86, 95% CI [1.43, 2.42] and aOR 1.36, 95%CI [1.04, 1.78]). High MVI was not associated with adverse outcomes. In the stratified analysis, race did not modify the association between SVI, MVI, ADI and adverse outcomes (P for interaction > 0.05 for all three).

**Conclusion**: Among the three indices, SVI and ADI might be the most reliable indices to capture community‐level determinants of obstetric outcomes. Social vulnerability and community‐level deprivation, independent of race, are associated with adverse pregnancy outcomes.

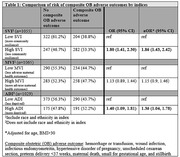

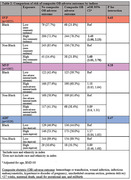

## 981 | The Effect of Maternal Blood Transfusion History on Alloimmunized Pregnancies

Brittany J. Arkerson^1^; Faezeh Aghajani^2^; Evelyn McGuire^3^; Katherine Modrall^3^; Lucy Salter^3^; Anthony L. Shanks^3^; Hiba J. Mustafa^4^

*^1^Indiana University, Indianapolis, IN; ^2^Maternal Fetal Care Center, Division of Fetal Medicine and Surgery, Boston Children's Hospital and Harvard School of Medicine, Boston, MA; ^3^Indiana University School of Medicine, Indianapolis, IN; ^4^Indiana University and Riley Children's Hospital, Indianapolis, IN*

4:00 PM ‐ 6:00 PM

**Objective**: To evaluate the impact of prior blood transfusion on diagnosis and severity of maternal alloimmunization.

**Study Design**: A single‐center retrospective cohort study of alloimmunized singleton gestations with antibodies known to be associated with hemolytic disease of the fetus and newborn (HDFN) from 2018 to 2023. We excluded pregnancies if the fetus was determined to be not at risk for HDFN either by negative paternal genotyping or negative invasive diagnostic testing. Multiple gestations were excluded as well. Univariate and multivariate analyses were performed. P‐value < 0.05 was considered statistically significant.

**Results**: 76 patients with alloimmunization were identified who had a documented history of blood transfusion. 40 patients had no prior history of blood transfusion while 36 did report a prior transfusion. The type of antibody present differed significantly between the two groups. Patients with a history of blood transfusion presented with a wider variety of antibodies than those who did not. Patients with a history of blood transfusion predominantly presented with anti‐K, whereas those without prior blood transfusion history presented with primarily anti‐D. Both groups were equally likely to have more than one antibody present. Patients with a history of blood transfusion had their first titer drawn at a later gestational age (11.02 ± 2.2 vs. 9.6 ± 2.3). However, there was no significant difference between the maximum antibody titer reached during pregnancy (101.5 ± 251.3 vs. 220.2 ± 475.5). Moreover, both groups were equally likely to have MCA Dopplers initiated, reach maximum MCA PSV MoM ≥1.5, and require IUT.

**Conclusion**: Patients who develop alloimmunization due to blood transfusion are more likely to present with a wider variety of antibodies but this does not impact the need for MCA Doppler monitoring or IUT in a clinically significant way.

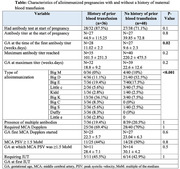

## 982 | Association of Breastfeeding and Early Childhood Preventative Dental Care

Rodolfo Fernandez‐Criado^1^; Ntami P. Echeng^2^; Laurie B. Griffin^3^; Margaret M. Thorsen^3^; Anna R. Whelan^4^; Brock E. Polnaszek^5^

*^1^Women & Infants Hospital of Rhode Island / Alpert Medical School of Brown University., Providence, RI; ^2^Women & Infants Hospital of Rhode Island / Alpert Medical School of Brown University, Providence, RI; ^3^Warren Alpert Medical School of Brown University / Women and Infants Hospital of Rhode Island, Providence, RI; ^4^University of Massachusetts Chan School of Medicine, Worcester, MA; ^5^Medical College of Wisconsin, Milwaukee, WI*

4:00 PM ‐ 6:00 PM

**Objective**: Breastfeeding has established benefits for maternal and childhood health. However, mixed evidence exists on the impact of breastfeeding and childhood dental caries. Preventative pediatric dental care can mitigate the risk of dental caries in this population. Thus, we examined the association of breastfeeding and preventative dental care visits among children age 0‐5 years in the United States.

**Study Design**: We analyzed data from the 2021 National Survey of Children's Health, an annual cross‐sectional survey across multiple domains of a family unit's social determinants of health. The primary outcome was preventative dental care visit, defined by presence or absence of a dentist or oral health provider visit in past 12 months (check‐ups, education, cleanings, sealants, fluoride, or x‐ray). The main exposures were breastfeeding and breastfeeding exclusivity. Established parental and household unit characteristics were explored. A weighted and multivariable logistic regression analysis calculated adjusted odds ratios of preventative dental care visits.

**Results**: The majority 24,668 (81%) of children age 0‐5 years had received breastmilk via direct or expressed breast milk. More than half 14,929 (56%) of these children had a preventative dental care visit. Dental check‐up, cleaning, education, x‐ray, fluoride, and sealant visits occurred more frequently in those children who were breastfed compared to those who were not (Table 1). After adjusting for differences between groups, breastfeeding or exclusive breastfeeding was not associated with odds of preventative dental visits, while having unmarried parents and less severe household poverty was associated with reduced odds (Table 2).

**Conclusion**: Social determinants such as family and economic instability–not breastfeeding status–were key drivers of preventative dental care. Dental health care education should be integrated into prenatal care conversations around breastfeeding to dispel myths and promote optimal parental and early childhood health.

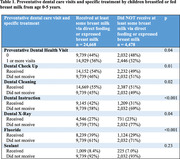

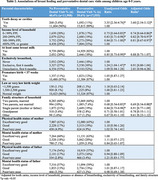

## 983 | Longitudinal Change in Short‐Term Variation with Intraamniotic Infection After PPROM: A Historical Cohort Study

Brynhildur T. Birgisdottir^1^; Tomas Andersson^2^; Ingela Hulthén Varli^3^; Sissel Saltvedt^3^; Ke Lu^4^; Farhad Abtahi^5^; Ulrika Åden^6^; Malin Holzmann^6^

*^1^Karolinska University Hospital, Stockholm, Stockholms Lan; ^2^Institute of Environmental Medicine, Karolinska Institutet, Stockholm, Stockholms Lan; ^3^Department of Women's and Children's Health, Karolinska Institute, Stockholm, Stockholms Lan; ^4^Department of Electrical Engineering, Chalmers University of Technology, Gothenburg, Vastra Gotaland; ^5^Department of Clinical Science, Intervention and Technology, Karolinska Institutet, Stockholm, Stockholms Lan; ^6^Department of Women's and Children's Health, Karolinska Institutet, Stockholm, Stockholms Lan*

4:00 PM ‐ 6:00 PM

**Objective**: Currently used diagnostic tools have been shown to have poor diagnostic performance for intraamniotic infection (IAI). In search of better diagnostic tools we have studied short‐term variation (STV) in fetal heart rate in pregnancies with preterm prelabor rupture of membranes (PPROM). We have previously shown that in IAI exposed pregnancies, STV was > 20% lower in the last cardiotocography trace before start of labor, as compared to those not exposed. The current study was aimed at further examining this association by examining the longitudinal change in STV in association with IAI after PPROM.

**Study Design**: We performed a historical cohort study on 628 singleton pregnancies with PPROM, delivering between 24 + 0 to 33 + 6 gestational weeks. The studied exposure was IAI, using early onset neonatal sepsis as a proxy as no easily available method exists for confirming IAI antepartum, and IAI and early onset neonatal sepsis are strongly associated. The main outcome was STV in fetal heart rate. At least two available cardiotocography traces per fetus were required. A total of 9 690 cardiotocography traces were analyzed.

**Results**: Fetuses exposed to IAI had a 26.5% steeper decline in their STV during the last 24 hours before the start of labor when compared to fetuses not exposed (95% CI ‐32.9% to ‐19.4%; *P* < 0.001). After adjustment for antenatal corticosteroids, the decline remained significant. The decline became less prominent but remained significant when also adjusting for the baseline frequency (‐12.7% [95% CI ‐19.3% to ‐5.5%], *P* < 0.001). In the IAI‐exposed group, the baseline frequency increased by 11.1 bpm during the last 12 hours before the start of labor, beyond those who were not exposed (95% CI 8.3 bpm to 13.8 bpm; *P* < 0.001).

**Conclusion**: In pregnancies affected by IAI the STV has a steeper decline in the last 24 hours before start of labor as compared to pregnancies not affected by IAI, even after adjustment for increasing baseline frequency. The association of STV in relation to IAI needs to be further studied in order to evaluate and establish STVs usefulness in monitoring patients for IAI.

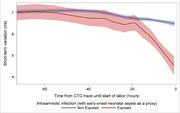

## 984 | Racial and Ethnic Differences in Delivery Characteristics in Patients with a Cesarean Wound Infection

Carmen Rauh Garrido^1^; Rachel L. Wood^2^; Sarah K. Dotters‐Katz^3^; Janice Wong^3^

*^1^Duke School of Medicine, Durham, NC; ^2^Duke University, Durham, NC; ^3^Duke University School of Medicine, Durham, NC*

4:00 PM ‐ 6:00 PM

**Objective**: Black race and Hispanic ethnicity have been identified as risk factors for cesarean wound infection. This likely represents systemic bias, as there is no biological explanation for these differences. This study sought to identify differences among self‐reported racial and ethnic groups in delivery or postpartum factors that could be targeted to lessen disparities.

**Study Design**: This was an IRB‐approved, retrospective cohort study of patients with post‐cesarean wound infections requiring an emergency department or triage wound‐related visit from a single healthcare system between 6/1/2013‐7/31/2022, excluding patients with placenta accreta or severe preeclampsia. Primary exposure was self‐reported race and ethnicity. Outcomes of interest were delivery characteristics predisposing to wound infection (GBS status, cesarean operative time, antibiotics received, estimated blood loss, skin closure with staples, peripartum infection). Bivariate statistics were used to analyze data. Regression models developed to control for confounders.

**Results**: Of 533 patients with post‐cesarean wound infections, 180(33.7%) identified as non‐Hispanic Black(NHB), 233(43.7%) non‐Hispanic White(NHW), and 80(15.0%) as Hispanic. NHB and Hispanic patients had a significantly higher BMI and were more likely to have public insurance than NHW patients. NHB patients were more likely to use tobacco than NHW patients(Table 1). Hispanic patients had a higher rate of PP endometritis treatment(n = 16,8.7%), compared to NHW(n = 7,3.0%), and NHB(n = 3,3.8%). In regression models, NHB patients were significantly more likely to be GBS positive(OR 1.70 (1.01,2.85)) and were more likely to be diagnosed in triage(OR 1.51 (0.98, 2.33)), though this finding was not statistically significant(Table 2).

**Conclusion**: Among patients with wound infections after cesarean, PP endometritis, GBS, and diagnosis location differed, yet no modifiable delivery characteristics were noted among racial and ethnic groups. Future research may investigate disparities in peripartum infection as a risk factor for wound infection and differences in postpartum healthcare access.

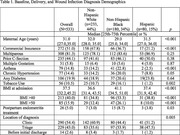

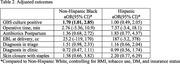

## 985 | Tight Versus Standard Postpartum Control Following Hypertensive Disorders of Pregnancy: A Randomized Controlled Feasibility Trial

Carrie Bennett^1^; Rosemary Shay^1^; Sanjana Ghosh^1^; Sila Yavan^1^; Jennifer Hernandez^1^; Hyagriv Simhan^1^; Malamo Countouris^1^; Janet Catov^2^; Alisse Hauspurg^3^

*^1^University of Pittsburgh Medical Center, Pittsburgh, PA; ^2^Magee‐Womens Hospital, Pittsburgh, PA; ^3^Alpert Medical School of Brown University, Providence, RI*

4:00 PM ‐ 6:00 PM

**Objective**: To assess the feasibility of performing a randomized controlled trial (RCT) investigating tight versus standard postpartum blood pressure (BP) control following hypertensive disorder of pregnancy (HDP) through a postpartum remote monitoring program.

**Study Design**: We performed a single‐blinded feasibility RCT from 11/2023‐4/2024. Tight BP control was defined as medication initiation consistent with American Heart Association guidelines: inpatient BP (mmHg) ≥140/90 and/or outpatient ≥135/85 for two values at least 4 hours apart. Standard BP control was defined as medication initiation for BP ≥150/100 at least 4 hours apart. Eligible individuals were ≥18 years of age, diagnosed with HDP, and enrolled in our institution's remote BP management program. Individuals were excluded if they were diagnosed with chronic hypertension, pre‐gestational diabetes, renal or cardiac disease, a fetal anomaly, or currently used anti‐hypertensives. We compared demographics, inpatient medication initiation, and outpatient medication initiation among groups.

**Results**: We enrolled 60 individuals over a 5‐month time course out of 143 who were approached (43%); 29 were randomized to tight control and 31 to standard control. Average age, body mass index, race/ethnicity, health insurance status, and household incomes were similar among groups (Table). Those in the standard control arm were more likely to be diagnosed with preeclampsia, including with severe features (p = 0.04). Anti‐hypertensive medications were initiated at similar rates among groups both inpatient (31% tight vs 25.8% standard, p = 0.65) and outpatient (20.7% tight vs 22.6% standard, p = 0.86).

**Conclusion**: While a single center trial of tight versus standard postpartum BP control is feasible, there were similar rates of oral anti‐hypertensive initiation among groups. This may be due to a differential representation of HDP severity among groups with a trend toward higher enrollment BP in patients randomized to standard control.

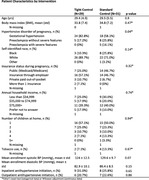

## 986 | Prenatal Characteristics Predictive of Xylazine Positivity: Who is being Exposed?

Cassandra Trammel^1^; Vahid Azimi^2^; Bridgit Crews^2^; Stephen Roper^2^; Nandini Raghuraman^3^; Antonina I. Frolova^4^; Megan L. Lawlor^2^; Hayley Friedman^2^; Jeannie C. Kelly^3^

*^1^Washington University in St Louis, St. Louis, MO; ^2^Washington University in St. Louis, St. Louis, MO; ^3^Washington University School of Medicine in St. Louis, St. Louis, MO; ^4^Washington University School of Medicine, St. Louis, MO*

4:00 PM ‐ 6:00 PM

**Objective**: Xylazine, a non‐opioid veterinary sedative, is increasingly present as a contaminant in nonprescribed fentanyl. We sought to assess demographic and prenatal characteristics’ association with odds of xylazine positivity in pregnant patients who use fentanyl.

**Study Design**: We performed a case control study of all patients positive for nonprescribed fentanyl on urine drug screening (UDS) at any point during pregnancy on an urban Midwestern tertiary care center labor unit in 2023. Patients with xylazine on any pregnancy or neonatal UDS were classified as xylazine cases, while fentanyl only patients were controls. Data was collected via chart review and odds ratios calculated. For cells with no occurrences, a Haldane correction was used to approximate an odds ratio.

**Results**:  A total of 65 patients with nonprescribed fentanyl use were identified, with 40 xylazine exposure cases based on testing of the pregnant person or neonate. There were no factors identified that were associated with an increased or decreased odds of xylazine positivity. The majority of patients in both groups reported public insurance (xylazine positive 97.3, negative 100%) concurrent use of a non‐opioid substance (100%, 91.3%), and tobacco (100%, 93.3%), and having an antepartum admission (50%, 60%). A large proportion reported intravenous use (41.5%, 16.7%), had evidence of stimulant use (100%, 75%), a comorbid psychiatric condition (61%, 79%) and infection with hepatitis C (53.7%, 29.2%). The majority presented to care after the first trimester (80.5%, 62.5%), and approximately half in both groups were on medication treatment for opioid dependence.

**Conclusion**: Fentanyl exposure continues to be associated with a high degree of social vulnerability, concomitant use of other substances, comorbid conditions, and limited prenatal care, however none of the background variables evaluated were associated with an increased or decreased odds of xylazine exposure. As xylazine may increase perinatal morbidity, all patients with fentanyl use should be counseled regarding potential risks of xylazine exposure.

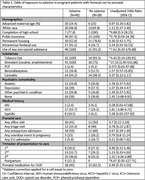

## 987 | Fentanyl Exposure in the Era of Xylazine: an Update on Perinatal Outcomes

Cassandra Trammel^1^; Vahid Azimi^2^; Bridgit Crews^2^; Stephen Roper^2^; Nandini Raghuraman^3^; Antonina I. Frolova^4^; Hayley Friedman^2^; Megan L. Lawlor^2^; Jeannie C. Kelly^3^

*^1^Washington University in St Louis, St. Louis, MO; ^2^Washington University in St. Louis, St. Louis, MO; ^3^Washington University School of Medicine in St. Louis, St. Louis, MO; ^4^Washington University School of Medicine, St. Louis, MO*

4:00 PM ‐ 6:00 PM

**Objective**: Xylazine, a non‐opioid veterinary sedative, is increasingly contaminating nonprescribed fentanyl. We sought to explore outcomes in fentanyl‐exposed obstetric patients in an urban labor unit with high prevalence of xylazine in the local fentanyl supply.

**Study Design**: We performed a retrospective cohort study of all pregnant patients with nonprescribed fentanyl on urine drug screening (UDS) at a Midwestern tertiary care center in 2023. UDS via mass spectrometry is performed after a positive result on universal verbal screening. The primary outcome was preterm birth (PTB). Secondary outcomes included out of hospital birth (OOHB), adult intensive care unit (ICU) admission, neonatal ICU (NICU) admission, and other perinatal metrics. A secondary analysis comparing xylazine positive and negative dyads was performed with Χ^2^ or Mann Whitney U tests as appropriate.

**Results**: 65 patients were identified. 57 had peripartum care in our facility, and 56 had neonatal data available with 59 neonates after accounting for twins. 62% of pregnant patients had xylazine exposure. Groups were similar aside from higher rates of intravenous drug use in the xylazine group (41.5 vs 16.7%, p = 0.04). 41.7% of xylazine exposed patients experienced PTB, compared to 28.6% of unexposed patients (RR 1.46, 95% CI 0.67‐3.18) with a median gestational age of 37 weeks. Unplanned OOHB occurred in 19.4% of xylazine positive dyads and 9.5% of negative dyads, maternal ICU admission in 5.9% and 16.7%, and NICU admission in 76.5% and 70%, though differences were not significant. Xylazine dyads had lower rates of antepartum bleeding (2.4 vs 25%, RR = 0.09, [0.01‐0.76]) and higher fentanyl positivity at delivery (100 vs 81%, RR = 1.23, [1.003‐1.52]). Xylazine exposed neonates were less likely to be discharged in parental custody (5.6 vs 26.1%, RR 0.21, [0.58‐2.3]).

**Conclusion**: Perinatal fentanyl use is associated with significant obstetric and neonatal morbidity regardless of xylazine exposure. Preliminary data suggests that xylazine may be a marker of severe disease and should be targeted for future study.

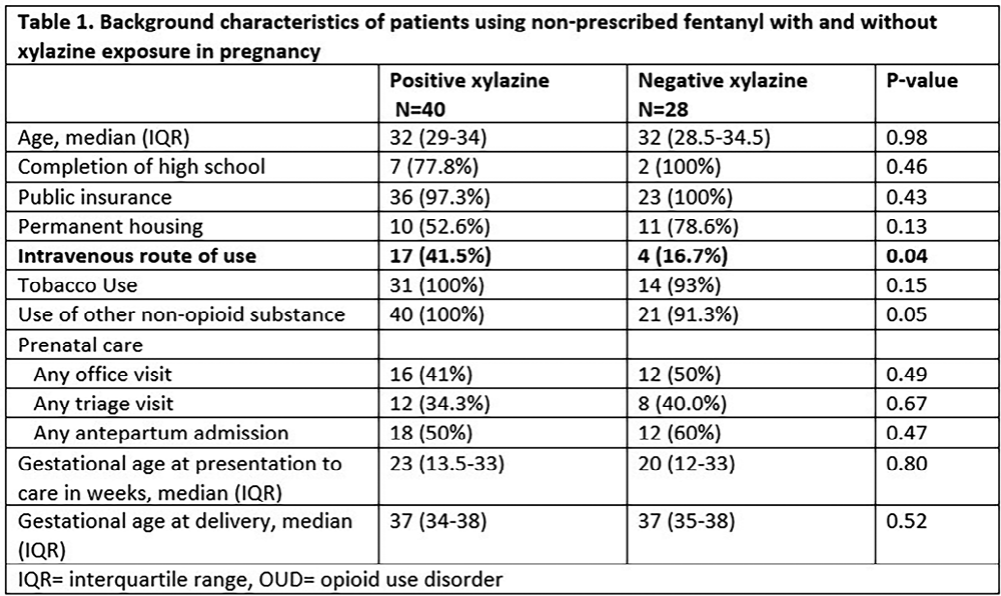

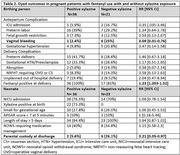

## 988 | Safety of Cervical Cerclage Between 24 and 27 Weeks: Analysis from the IC‐CLEAR Cohort Study

Catalina M. Valencia^1^; Arturo Cardona^1^; Jose Bareño Silva^2^; Lina Herrera‐ Agudelo^3^; Susana Villegas‐Sanchez^4^; Santiago Galeano‐Herrera^3^; Silvia M. Villegas^5^; Nicole D. Rojas^6^; Rupsa C. Boelig^7^; Vincenzo Berghella^8^; Joseph Bell^9^; Leonardo Pereira^10^; Luisa López‐Torres^11^; Richard M. Burwick^12^; Julio Mateus Nino^13^; Joanne N. Quiñones^14^; Jorge E. Tolosa^15^

*^1^Clinica del Prado, Unidad de Medicina Materno Fetal, Medellín, Colombia, Medellin, Antioquia; ^2^Universidad CES, Medellin, Colombia, Medellín, Antioquia; ^3^Departamento de Obstetricia y Ginecologia, Corporación Universitaria Remington, Medellín, Colombia, Medellin, Antioquia; ^4^Department of Obstetrics & Gynecology, Universidad CES, Medellín, Colombia, Medellin, Antioquia; ^5^Clinica del Prado, Departamento de Obstetricia y Ginecología, Medellín, Colombia, Medellin, Antioquia; ^6^Departamento de Obstetricia y Ginecología, Corporación Universitaria Remington, Medellín, Colombia, Medellin, Antioquia; ^7^Sidney Kimmel Medical College, Thomas Jefferson University, Philadelphia, PA; ^8^Sidney Kimmel Medical College of Thomas Jefferson University, Philadelphia, PA; ^9^St. Luke's University Health Network, Department of Obstetrics & Gynecology, Maternal Fetal Medicine, Bethlehem, PA, 18015, Bethlehem, PA; ^10^Oregon Health & Science University, Portland, OR; ^11^Universidad Pontificia Bolivariana, Medicina Materno Fetal, Medellín, Colombia, Medellin, Antioquia; ^12^San Gabriel Valley Perinatal Medical Group; Pomona Valley Hospital Medical Center, Pomona, CA; ^13^Atrium Health, Wake Forest University School of Medicine, Charlotte, NC; ^14^Lehigh Valley Health Network, Allentown, PA; ^15^St. Luke's University Health Network, Bethlehem, PA*

4:00 PM ‐ 6:00 PM

**Objective**: Cervical cerclage before 24 weeks gestation prolongs pregnancy in patients at risk for preterm birth. Its benefits after 24 weeks are unclear due to limited safety data. We sought to describe maternal and perinatal outcomes with cerclage placement at 24‐27 weeks.

**Study Design**: This is a secondary analysis from the International Collaborative for Cerclage Longitudinal Evaluation and Research (IC‐CLEAR), a cohort study of women with singleton pregnancies and cerclage placement. Participants were enrolled at 6 institutions in the U.S. and 2 in Colombia from June 2016 to June 2020. For this study, the primary exposure was cerclage placement between 24 0/7 and 27 5/7 weeks; the comparator group was cerclage between 20 0/7 and 23 6/7 weeks. Maternal and neonatal outcomes were evaluated using a test of medians and chi‐squared test.

**Results**: 35 out of 839 pregnancies underwent cerclage placement between 24 0/7 and 27 5/7 weeks; 88% (n = 31) were from the centers in Colombia and 12% (4) in the U.S. Cerclage was performed at a median gestational age of 25.4 weeks (IQR 24.8‐26.3) and the median latency from placement to delivery was 10.7 weeks (IQR 7.4‐11.35). Premature rupture of membranes occurred in 18% (n = 6), and clinical chorioamnionitis in 3% (n = 1). Three patients required ICU admission, two due to preeclampsia with severe features. NICU admission was required for 10 neonates (32%), with two cases of sepsis and two neonatal deaths. When comparing maternal and perinatal safety outcomes in those with cerclage at 24‐27 weeks to those with cerclage at 20‐24 weeks (n = 328), there was a higher rate of maternal ICU admission, not associated with cerclage use; no significant differences were observed in maternal or neonatal infectious morbidity, or in other neonatal morbidity or mortality (Table 1).

**Conclusion**: Cervical cerclage performed between 24 and 27 weeks is not associated with a significant increase in maternal morbidity or adverse neonatal outcomes. Randomized controlled studies to evaluate the effectiveness and safety of cerclage in patients at 24 or more weeks of gestation are needed.

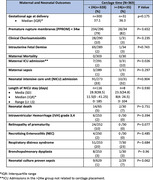

## 989 | Sonographic Indicators of Intralesional Haemorrhage in Fetal Lymphatic Malformation: a Retrospective Cohort Study

Charles Litwin^1^; Yada Kunpalin^1^; Lennart Van der Veeken^1^; Paolo Campisi^2^; Manuel Carcao^3^; Johannes Keunen^1^; Nimrah Abbasi^1^; Greg Ryan^4^; Tim Van Mieghem^5^; Shiri Shinar^1^

*^1^Fetal Medicine Unit, Department of Obstetrics and Gynaecology, Mount Sinai Hospital and University of Toronto, Toronto, ON; ^2^Department of Otolaryngology–Head & Neck Surgery, Hospital for Sick Children and University of Toronto, Toronto, ON; ^3^Department of Haematology/Oncology, Hospital for Sick Children and University of Toronto, Toronto, ON; ^4^University of Toronto, Toronto, ON; ^5^Mount Sinai Hospital and University of Toronto, Toronto, ON*

4:00 PM ‐ 6:00 PM

**Objective**: To review the rate of intralesional hemorrhage (IH), sonographic indicators of IH and outcomes in a cohort of fetuses with lymphatic malformations (LMs).

**Study Design**: We performed a single‐center retrospective cohort study of fetal LMs over a 13‐year period. IH was defined as ultrasound findings demonstrating reticular echogenicity, fluid‐fluid levels or clot without flow on Doppler. Fetal transfusion was considered if the middle cerebral artery peak systolic velocity (MCA‐PSV) was elevated >1.5 MoM. Lymphangioma volume ratio (LVR) was calculated using the formula: 0.532 x (tumour width x length x height in cm)/head circumference (cm). Predictive strength of LVR on IH was determined by ROC analysis.

**Results**: Twenty‐six isolated LMs were identified antenatally. Three pregnancies were terminated and 5 were lost to follow‐up. Of the remaining, 10 involved the head and neck (H&N) and 8 involved the axilla. H&N LMs were diagnosed at 26.1 weeks gestation (IQR 21.6‐28.8 weeks) with an LVR at presentation of 2.24 (IQR 0.68‐17.29). Axillary LMs were diagnosed at 28.1 weeks gestation (IQR 23.6‐32.3 weeks) with an LVR at presentation of 4.17 (IQR 1.26‐7.02). Lesion growth was observed over the course of pregnancy (Fig 1). Five cases of IH were detected at 28.7 weeks gestation (IQR 24.4‐35.2) and were found to have significantly larger tumour volume compared to those without bleeding (LVR: 11.09±5.25 vs 5.33±4.11, p = 0.04, table 1). LVR greater than 6.86 had a detection rate of 80% (95%CI 37.6‐99.0%) for IH with a specificity of 66.7% (95% CI 39.1‐86.2%). Polyhydramnios was present in all cases of IH but only in 2 out of 13 without IH. Of the 5 cases of IH, 2 died in utero, 2 required fetal transfusions and all 3 survivors were treated with sclerotherapy and rapamycin postnatally.

**Conclusion**: Intralesional hemorrhage is a recognized complication of fetal LMs with polyhydramnios. LVR may serve as a good predictor for IH prompting serial follow‐up with MCA‐PSV. Surviving fetuses typically require further treatment postnatally.

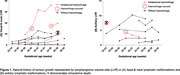

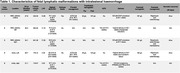

## 990 | Diuretics for Preventing Postpartum Readmission: A Systematic Review and Meta‐Analysis

Chase Calvert^1^; Rajesh Reddy^1^; Molly Singer^2^; Jeny Ghartey^3^; Lorie M. Harper^1^

*^1^Dell Medical School, Austin, TX; ^2^Dell Medical School, University of Texas at Austin, Austin, TX; ^3^University of Texas at Austin, Austin, TX*

4:00 PM ‐ 6:00 PM

**Objective**: Hypertensive disease of pregnancy (HDP) is a leading cause of maternal postpartum readmission. While diuretics have been shown to reduce postpartum hypertension in women with HDP, their effectiveness in preventing postpartum readmission remains unclear. We performed a meta‐analysis and systematic review to evaluate the impact of diuretic use on postpartum readmission rates in patients with HDP.

**Study Design**: We conducted a comprehensive search on PubMed, MEDLINE, Web of Science, PROSPERO, Cochrane Library, and ClinicalTrials.gov using a combination of key terms. Studies were included if they were published after 1995 and comprised clinical trials or observational cohorts. Eligible studies compared diuretic treatment to non‐diuretic‐based or no treatment in the postpartum period for patients with HDP. The primary outcome measured was readmission rates.

**Results**: Six studies, encompassing a total of 2,893 patients, were included. Five studies utilized loop diuretics (furosemide [4] and torsemide [1]), and one utilized a thiazide. The pooled odds ratio (OR) for readmission with any diuretic use was 1.35 (95% CI: 0.66 to 2.75). Visual inspection of the funnel plot revealed some asymmetry, suggesting that smaller studies showing positive results (i.e., those indicating a beneficial effect of diuretics on reducing readmission rates) might be missing. This asymmetry indicates potential publication bias. The variability among the studies was moderate (I^2^ = 44.8%, P = 0.107). Metaregression analysis highlighted that the type of diuretic used had a significant impact on the results. Specifically, secondary analyses focusing only on studies that used loop diuretics (furosemide and torsemide) consistently showed a trend towards increased readmission rates.

**Conclusion**: Diuretic use in postpartum patients with HDP is not associated with decreases in readmission. Potential publication bias and the significant impact of diuretic type, particularly loop diuretics, on readmission rates highlight the need for targeted, large‐scale prospective studies to accurately assess their role in managing postpartum hypertension.

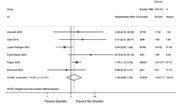

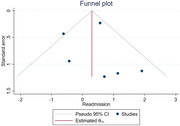

## 991 | Poor Periconceptional Diet and Sleep Quality are Associated with Hypertensive Disorders of Pregnancy

Chelsea A. DeBolt^1^; Zhan Zhao^1^; Katharine McCarthey^1^; Kimberly B. Glazer^2^

*^1^Icahn School of Medicine at Mount Sinai, New York, NY; ^2^Department of Population Health Science and Policy; Department of Obstetrics, Gynecology and Reproductive Science, Blavatnik Family Women's Health Research Institute, Icahn School of Medicine at Mount Sinai, New York, NY*

4:00 PM ‐ 6:00 PM

**Objective**: Hypertensive disorders of pregnancy (HDP) remain a leading problem in the US and risk factor for future adverse cardiovascular health. While recommendations to support long‐term cardiovascular health exist for adults, specific lifestyle and wellness guidelines intended to improve pregnancy health are limited. We sought to examine the relationship between periconceptional lifestyle‐related factors, including nutrition, sleep, and physical activity and development of HDP.

**Study Design**: We conducted a secondary analysis of singleton pregnancies to nulliparous individuals in the Nulliparous Pregnancy Outcomes Study: Monitoring Mothers‐to‐be (nuMoM2b) prospective cohort. We studied 7,942 participants without chronic hypertension or pregestational diabetes. We assessed periconceptional dietary quality using the Alternative Healthy Eating Index‐2010 (AHEI‐2010) score, sleep quality based on duration and snoring, and physical activity based on duration and adequacy as reported at study visit 1 (6 to 13 weeks’ gestation). Multivariable logistic regression estimated odds ratios for HDP adjusted for markers of socioeconomic status, depression, perceived social support, maternal age, tobacco use, and medical comorbidities.

**Results**: Individuals who developed HDP were more likely to be older, non‐Hispanic Black, unmarried, and use tobacco, with higher BMI compared to those who did not (Table 1). In adjusted models, AHEI‐2010 score in the lowest quartile was associated with 1.3 times (95% confidence interval 1.04‐1.58) higher odds of HDP compared to the highest quartile (Table 2). Early pregnancy snoring was associated with 1.4 times (95% confidence interval 1.18‐1.63) higher odds of HDP (Table 2).

**Conclusion**: Poor diet quality and early pregnancy snoring were associated with HDP in nulliparas without preexisting hypertension or diabetes. Findings support developing low‐cost lifestyle interventions targeting diet and snoring to reduce HDP risk, emphasizing the importance of preconception and early pregnancy care to improve maternal and infant health.

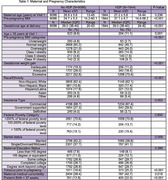

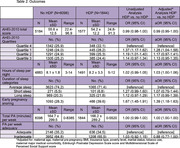

## 992 | Is the Timing of Gestational Diabetes Screening Associated with Adverse Perinatal Outcomes?

Chloe Getrajdman^1^; Isabelle C. Band^2^; Sara Wetzler^2^; Alexandra N. Mills^3^; Guillaume Stoffels^2^; Chelsea A. DeBolt^2^; Calvin E. Lambert, Jr.^2^

*^1^Icahn School of Medicine at Mount Sinai, Icahn School of Medicine at Mount Sinai, NY; ^2^Icahn School of Medicine at Mount Sinai, New York, NY; ^3^Division of Maternal‐Fetal Medicine, Icahn School of Medicine at Mount Sinai, New York, NY*

4:00 PM ‐ 6:00 PM

**Objective**: Though there is a universal recommendation to screen for gestational diabetes (GDM) from 24‐28 weeks, the impact of the timing of screening within this 5‐week interval has yet to be studied. The study objective was to determine whether the gestational age of 2‐step GDM screening among those diagnosed with GDM is associated with adverse outcomes

**Study Design**: A retrospective cohort study of singleton, non‐anomalous pregnancies at one institution from 2019‐2022 and diagnosed with GDM via the 2‐step test. The early cohort included patients screened from 24‐26.6 and the late cohort included those screened from 27‐28.6. The primary outcome was a composite of perinatal morbidity: macrosomia, shoulder dystocia, hypertensive disorders of pregnancy, cesarean delivery for presumed macrosomia, neonatal intensive care unit (NICU) admission, among other adverse neonatal outcomes. Additional secondary maternal and neonatal outcomes were collected. Bivariate analyses, univariate and multivariate regressions were performed

**Results**: Of 433 patients included, 213 (49.2%) screened early and 220 (50.8%) screened late. More patients self‐identified as Asian in the early group (p = 0.0002) and a larger proportion of patients had 1^st^ trimester GDM screening (p = 0.001) in the late group (Table 1). In univariate and multivariate analyses, there were no significant differences in the composite outcome between the groups (late vs early screening: aOR 0.82, 95% CI 0.54‐1.26, p = 0.36). A significantly higher incidence of macrosomia (7 vs 2.7%, p = 0.04) and trend toward increased NICU admission (8.9 vs 4.5%, p = 0.07) was seen in the early group (Table 2)

**Conclusion**: Among patients diagnosed with GDM using the 2‐step test, there was no difference in odds of composite perinatal morbidity among patients screened at the earlier or later end of the 24‐28 week screening interval, though there were differences in race and prevalence of early glucose screening between the groups. Additional research is needed to further elucidate these findings and understand the implications of the timing of GDM screening on maternal and neonatal outcomes

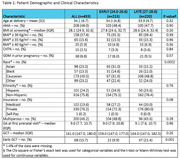

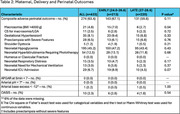

## 993 | Is Time Between Screening and Diagnosis of Gestational Diabetes Associated with Adverse Outcomes?

Chloe Getrajdman^1^; Sara Wetzler^2^; Isabelle C. Band^2^; Alexandra N. Mills^3^; Guillaume Stoffels^2^; Calvin E. Lambert, Jr.^2^; Chelsea A. DeBolt^2^

*^1^Icahn School of Medicine at Mount Sinai, Icahn School of Medicine at Mount Sinai, NY; ^2^Icahn School of Medicine at Mount Sinai, New York, NY; ^3^Division of Maternal‐Fetal Medicine, Icahn School of Medicine at Mount Sinai, New York, NY*

4:00 PM ‐ 6:00 PM

**Objective**: To determine whether the interval between gestational diabetes (GDM) screening glucose challenge test (GCT) and diagnostic glucose tolerance test (GTT) is associated with adverse perinatal outcomes

**Study Design**: A retrospective cohort study of singleton, non‐anomalous pregnancies at one institution from 2019‐2022 and diagnosed with GDM via the 2‐step test. The primary outcome was a composite of perinatal morbidity: macrosomia, shoulder dystocia, hypertensive disorders of pregnancy, cesarean delivery for presumed macrosomia, neonatal intensive care unit (NICU) admission, among other adverse neonatal outcomes. The comparison groups included patients with an interval between GCT and GTT of less than 10 days or greater than or equal to 10 days. Bivariate analyses, univariable and multivariable regressions were performed

**Results**: Of 389 patients included, 258 (66.3%) had an interval of less than 10 days between GCT and GTT and 131 (33.7%) had an interval of greater than or equal to 10 days. Patients in the ^3^10 days group were more likely to be obese at time of GCT (30 vs 43.7%, p = 0.009) and more likely to be of Black race (9.4 vs 19.2%, p = 0.001) and have public insurance (16.3 vs 35.1%, p< 0.0001) (Table 1). There was a trend toward increased need for medication initiation in the ^3^10 day group (32.9 vs 42%, p = 0.08) and a significantly later gestational age at initiation of medication (31.0 vs 32.9 weeks, p = 0.003) (Table 2). In the univariate and multivariate analyses there was no significant differences in the composite outcome between the groups (< 10 vs ^3^10 days: aOR 1.03, 95% CI 0.64‐1.67, p = 0.9)

**Conclusion**: While patients who had GTT ^3^10 days after GCT were more likely to be Black, obese at time of GCT, have public insurance and initiated antihyperglycemic medication on average 2 weeks later than patients who had GTT within 10 days of GCT, no difference was seen in odds of adverse perinatal outcomes.

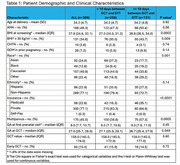

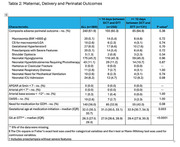

## 994 | Prevalence and Screening Methods for Asymptomatic Bacteriuria in Pregnancy in Low Resource Settings

Md Shahjahan Siraj^1^; Masum Billah^1^; Rashidul Haque^1^; Elizabeth M. McClure^2^; William A. Petri, Jr.^3^; Christian A. Chisholm^3^

*^1^International Centre for Diarrheal Disease Research, Bangladesh, Dhaka, Dhaka; ^2^RTI International, Research Triangle Park, NC; ^3^University of Virginia, Charlottesville, VA*

4:00 PM ‐ 6:00 PM

**Objective**: Screening for asymptomatic bacteriuria (AB) has been recommended to reduce the risk of pregnancy complications. The evidence for this practice is outdated and of low quality, and global implementation is inconsistent. We conducted a prospective study in a rural Bangladeshi population to ascertain the prevalence of AB, assess the utility of point of care screening methods, determine antimicrobial sensitivity patterns, and report the prevalence of low birth weight (LBW) in pregnancies with AB.

**Study Design**: We obtained urine samples from 600 asymptomatic pregnant women (1150 samples) being enrolled in a prospective registry of women in antenatal care. Samples were cultured on blood and MacConkey agar plates. AB was defined as presence of a single organism ≥105 CFU/mL. Antimicrobial sensitivity testing was performed. Samples were inoculated on urine dipslides and analyzed for nitrite and leukocyte esterase (LE) using urine dipsticks (positive dipstick = presence of both nitrite and LE). The performance of dipslide and dipstick were calculated using culture as the reference.

**Results**: A positive culture was found in 37/1150 samples (3.2%); the overall prevalence of AB during pregnancy was 5.2% (31/600 subjects positive on at least one sample). E. coli was most common (65% of total) followed by Enterococcus and Enterobacter. 90% of all isolates were sensitive to nitrofurantoin, including 100% of E. coli (Table). When compared to culture, the sensitivity of dipslide and dipstick for AB were 65% and 14%. 26% of pregnancies with AB resulted in a LBW newborn vs 21% of pregnancies with negative culture (p = 0.56).

**Conclusion**: AB was present in 1 in 20 pregnant subjects in this cohort. The sensitivity of dipstick and dipslide were poor, indicating lack of suitability to serve as a primary method of screening for AB in this population. Future trials assessing the impact of screening and treating AB in pregnancy on the incidence of adverse outcomes in low resource settings must address logistics and cost of widespread implementation of urine culture capacity given the low sensitivity of point of care screening tests.

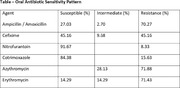

## 995 | Impact of an Addiction in Pregnancy Program on Breastfeeding in Patients with Substance Use Disorder

Christianna L. Tu^1^; Yumo Xue^2^; Charlotte B. McCarley^1^; Victoria C. Jauk^1^; Ayodeji Sanusi^2^; Rachel G. Sinkey^1^

*^1^University of Alabama at Birmingham, Birmingham, AL; ^2^Center for Women's Reproductive Health, University of Alabama at Birmingham, Birmingham, AL*

4:00 PM ‐ 6:00 PM

**Objective**: Breastfeeding (BF) plays a crucial role in promoting maternal and neonatal health. However, BF disparities exist among patients with substance use disorder (SUD). We evaluated the association between participation in a comprehensive addiction in pregnancy program (CAPP) and BF in patients with SUD.

**Study Design**: Retrospective cohort of pregnant patients with SUD who delivered between 4/2028—8/2022 at a tertiary care center in the southeast US. Exclusion criteria were: HIV, fetal demise, or infant adoption. CAPP provided multi‐disciplinary group‐based prenatal care to pregnant patients with SUD; patients were CAPP eligible if < 32 weeks, used ≥1 illegal substance, and scored ≥4 on the National Institute on Drug Abuse—Modified Assist Tool. The primary outcome was BF rate at discharge from the delivery‐associated hospitalization in those who participated in CAPP compared to patients with SUD who were CAPP eligible but did not enroll (non‐CAPP). Secondary outcomes included rate of BF at 6 weeks postpartum, BF intention, rate of neonatal opioid withdrawal syndrome (NOWS), highest modified Finnegan score, and neonatal length of stay (LOS). Outcomes were compared between groups.

**Results**: A total of 421 patients were included: 147 CAPP, 274 non‐CAPP. Of the patients who participated in CAPP, 69.4% BF their infant at discharge compared to 42.9% of non‐CAPP patients (p< 0.001, OR 2.89 (95% CI 1.85, 4.52)). Of CAPP patients who BF, 44.8% exclusively BF compared to 14.5% non‐CAPP (OR 4.63, 95% CI 2.84, 7.55); and 24.6% of CAPP patients provided both breastmilk and formula compared to 28.4% non‐CAPP (OR 0.80, 95% CI 0.50–1.30). At the postpartum visit, 32.1% of CAPP patients and 39.5% of non‐CAPP patients were still BF, with no significant difference between cohorts. There was no difference in NOWS, highest Finnegan score, or NICU LOS between groups (Table).

**Conclusion**: Participation in a comprehensive addiction in pregnancy program is positively associated with BF at discharge from the delivery‐associated hospitalization. Additional postpartum interventions are warranted to support BF after hospital discharge.

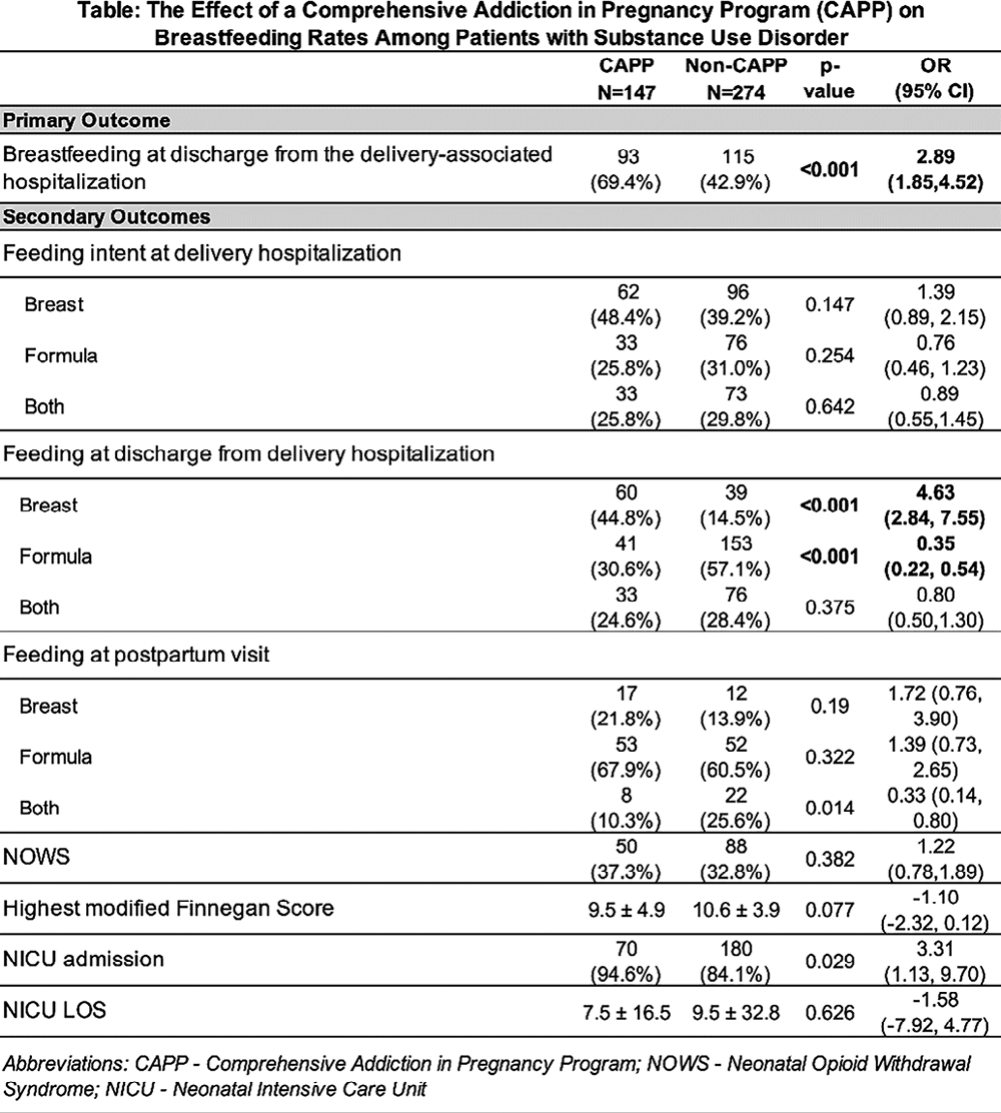

## 996 | Association of Fetal Heart Rate Tracing with Neonatal Adverse Outcomes at 32.0 to 36.6 weeks

Christina Cortes^1^; Kristen A. Cagino^2^; Aaron W. Roberts^3^; Rachel L. Wiley^4^; Claudia J. Ibarra^1^; Shareen Patel^1^; Khalil M. Chahine^1^; Natalie L. Neff^5^; Kimen S. Balhotra^1^; Tala Ghorayeb^1^; Holly Flores^6^; Fabrizio Zullo^7^; Hector M. Mendez‐Figueroa^1^; Suneet P. Chauhan^8^

*^1^McGovern Medical School at UTHealth, Houston, TX; ^2^UT Houston, Houston, TX; ^3^McGovern Medical School at UTHealth Houston, Houston, TX; ^4^University of California, San Diego, San Diego, CA; ^5^McGovern Medical School at UT Health, Houston, TX; ^6^University of Texas Health Science Center, Houston, TX; ^7^University of Rome La Sapienza, Rome, Lazio; ^8^Delaware Center of Maternal‐Fetal Medicine at Christiana Care, Delaware, DE*

4:00 PM ‐ 6:00 PM

**Objective**: The objective was to determine if features of fetal heart rate tracings (FHRT) were associated with an increased rate of neonatal adverse outcomes among preterm deliveries at 32.0–36.6 weeks.

**Study Design**: A retrospective review of all available FHRT among non‐anomalous singletons attempting labor at 32.0 to 36.6 weeks. The study was conducted at a Level IV maternal center during a consecutive 15‐month period. Obstetricians reviewing FHRT were blinded to the maternal characteristics, gestational age, and peripartum outcomes. Features of FHRT were dichotomized by time spent during the last hour before delivery (<50% vs. ≥50%). The primary outcome was the rate of composite neonatal adverse outcomes (CNAO). Chi‐square test was used to compare groups, and likelihood ratios (LR) were calculated for FHRT features that differed significantly for those with and without CNAO. A priori, LR greater than 10 was considered a useful diagnostic test (Jeschker R et al. JAMA 1994).

**Results**: Of 5,160 patients, 672 (13%) met the inclusion criteria. CNAO occurred in 57 (8.5%) newborns. Maternal characteristics (age, nulliparity, race/ethnicity, tobacco use, and hypertensive disorders) were similar between groups (Table 1). Compared to those without, minimal variability was significantly more likely to occur with CNAO (11.2% vs. 24.6% p< 0.01). Two features of FHRT occurred significantly less often with CNAO: moderate variability (85.4% vs. 66.7%, p < 0.01) and presence of accelerations (66.8% vs. 50.9% p < 0.02). The LR of the three features which differed significantly among those with and without CANO ranged from 1 to 2. Notably, there was no significant difference between CNAO with any of the following: prolonged decelerations, combination of decelerations, and recurrent decelerations (Table 2).

**Conclusion**: Though certain features of fetal heart rate tracings differed significantly in those with and without composite neonatal adverse outcomes at 32.0 to 36.6 weeks, the likelihood ratios of 1 to 2 suggest these characteristics are poor predictors of adverse neonatal outcomes in this cohort.

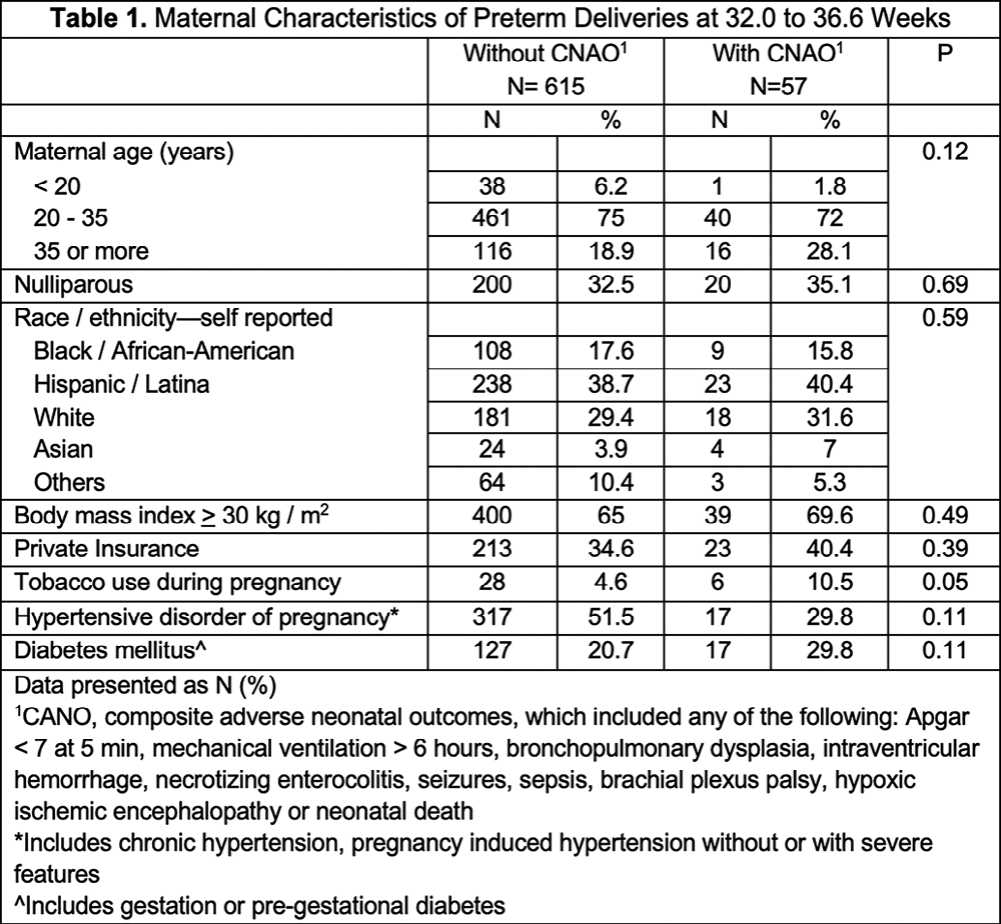

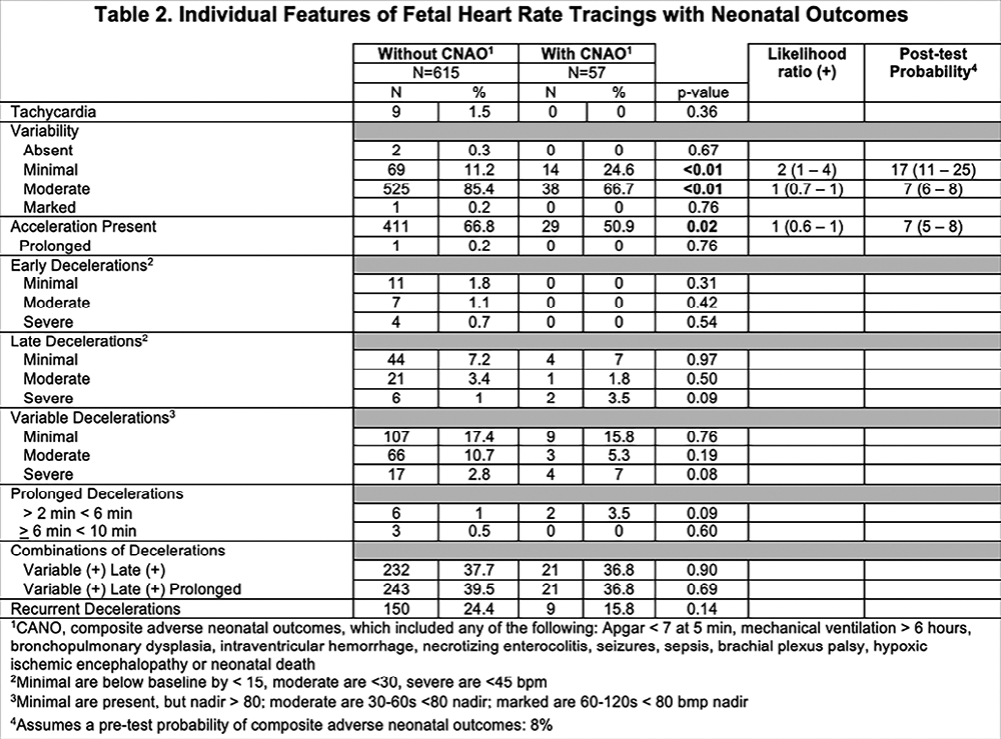

## 997 | Using Human‐Centered Design to Map the Perinatal Ecosystem for Pregnant People with Substance use Disorders

Christina N. Schmidt^1^; Benjamin S. Alpers^2^; Devika Patel^3^; Lara Chehab^2^; Melanie Thomas^4^; Neeti Doshi^4^; Heather Briscoe^4^; Marcy Spaulding^5^; Liliana Ocegueda^6^; Amanda Sammann^4^

*^1^Massachusetts General Hospital and Brigham and Women's Hospital, Cambridge, MA; ^2^The Better Lab, San Francisco, CA; ^3^Pennsylvania State University College of Medicine, Hershey, PA; ^4^University of California San Francisco, San Francisco, CA; ^5^Department of Maternal, Child and Adolescent Health, San Francisco Department of Public Health, San Francisco, CA; ^6^Department of Maternal, Child and Adolescent Health, Department of Public Health, San Francisco, San Francisco, CA*

4:00 PM ‐ 6:00 PM

**Objective**: Given the profound impacts of substance use disorders (SUDs) in pregnancy on the health and wellbeing of parents and newborns, improving access to perinatal services for this population is a priority. The objective of this study was to use human‐centered design methodologies to map the steps through which families affected by parental substance use progress through prenatal and postpartum care, with the goal of identifying opportunities to improve systems of care for substance‐exposed families.

**Study Design**: We used process mapping–a human‐centered design methodology–to characterize the steps associated with engaging in perinatal care systems as a parent with a SUD. Clinical and community‐based staff working within a safety‐net healthcare system participated in mapping workshops in which they responded 39 questions corresponding to the steps taken throughout a patient's journey from prenatal to postpartum care. Using a human‐centered design approach to inductive thematic analysis, we identified themes, constructed insight statements, and generated corresponding design opportunities.

**Results**: Forty‐seven stakeholders participated in process mapping. Eight insights emerged which clustered into 3 overarching themes: (1) provider variability in understanding policies and procedures, (2) care coordination challenges between team members, and (3) lack of standardization in engaging with child welfare. These findings generated 13 actionable design opportunities to strengthen perinatal care delivery.

**Conclusion**: Process mapping is a unique methodology to understand complex service delivery challenges and to improve systems of care for pregnant people with SUDs. In our ecosystem, process mapping revealed that unstandardized policies and care coordination challenges complicated perinatal care for substance‐exposed families. Strengthening healthcare systems to better serve the needs of this population is central to improving family outcomes.

## 998 | Improving Care Coordination for Socially Complex Perinatal Patients: A Human‐Centered Design Study

Christina N. Schmidt^1^; Benjamin S. Alpers^2^; Devika Patel^3^; Neeti Doshi^4^; Heather Briscoe^4^; Amanda Sammann^4^

*^1^Massachusetts General Hospital and Brigham and Women's Hospital, Cambridge, MA; ^2^The Better Lab, San Francisco, CA; ^3^Pennsylvania State University College of Medicine, Hershey, PA; ^4^University of California San Francisco, San Francisco, CA*

4:00 PM ‐ 6:00 PM

**Objective**: Designing novel solutions to address care fragmentation in the perinatal ecosystem requires rigorous evaluation of current workflows and engagement with the diverse network of stakeholders supporting patient care. Human‐centered design (HCD) is an established methodology for identifying challenges in clinical workflows, with the goal of producing actionable insights to inform novel interventions. The objectives of this study were to use HCD methodologies to 1) understand the processes of transitioning perinatal care within a safety‐net system, and 2) identify opportunities to improve care coordination for socially complex pregnant patients.

**Study Design**: We conducted semi‐structured interviews with patients, clinical providers, and staff at community‐based organizations (CBOs) working in a safety‐net healthcare system. Interviews were analyzed using an HCD approach to inductive thematic analysis. We also completed an inventory of all questionnaires administered to patients by their perinatal care teams. Items were coded according to inductively generated themes for a descriptive analysis of item overlap.

**Results**: Fifteen patients, 21 clinical providers and 6 CBO staff participated in interviews. Six insights statements emerged: 1) the perinatal care system is resource rich but coordination is poor, 2) safety‐net hospitals rely on CBOs to support patients but lack reliable communication systems with them 3) patient data protections complicate collaborative care planning, 4) poor communication around substance‐use policies introduces bias in decision‐making, 5) patients expect continuity of care and seeing new providers may engender mistrust. Eighteen perinatal questionnaires containing 833 items were coded into 21 unique themes, with 17 themes (81%) being duplicated between questionnaires.

**Conclusion**: Using an HCD approach, we identified key challenges in care coordination for socially complex perinatal patients. Interventions strengthening informational continuity between clinical and community‐based providers offer promise for improved care delivery in safety‐net systems.

## 1000 | Emergency Cervical Cerclage Versus Expectant Management in Women with Cervical Insufficiency Beyond 24 weeks

Christopher A. ENAKPENE^1^; Serin THOMAS^2^; Eric K. BRONI^3^; Brinkley COVER^4^; Erin ADAMS^4^; Omosiuwa ENAKPENE^5^; Sandra O. Anazor^6^; Michael GALLOWAY^7^

*^1^Texas Tech University Health Sciences Center, Permian Basin, Midland‐Odessa, TX; ^2^Texas Tech University Health Sciences Center, Midland‐Odessa, TX; ^3^University of Pennsylvania Perelman School of Medicine, Philadelphia, PA; ^4^Texas Tech University Health Sciences Center, School of Medicine, Midland‐Odessa, TX; ^5^University of Michigan, Ann Arbor, MI; ^6^Corewell Health West/Michigan State University, Grand Rapids, Michigan., Grand Rapids, MI; ^7^Texas Tech University Health Sciences Center, Odessa, TX*

4:00 PM ‐ 6:00 PM

**Objective**: To determine whether cervical cerclage (CC) reduced the rate of preterm birth (sPTB), prolonged pregnancy (PP) latency and improved perinatal outcomes (PO) in women with cervical insufficiency (CI) > 24 weeks compared to expectant management (EM).

**Study Design**: This was a prospective quasi‐experimental study that compared CC vs EM in patients with CI > 24 weeks (cervical dilatation + exposed fetal membranes). Prospective data of the CC group was compared with historic data of similar characteristics of EM between 2022 to 2024 at an urban hospital in a 3:1 ratio. Both groups received Vaginal Progesterone, Betamethasone, Antibiotics and Indomethacin. Patient in the CC group were discharged home within 24 hours of surgery while EM group remained on admission until delivery. The outcomes were overall sPTB < 37 weeks, early and late sPTB (< 34 and 34 ‐ 37 weeks), PO, NICU admission and duration. Baseline characteristic and PO were stratified and reported by intervention groups using Chi square and Kruskal Wallis. Multivariable and adjusted binomial and multinomial logistic regression models were used to assess rate of PTB between the two study groups. **(Table 2)**.

**Results**: Of the 91 patients recruited, 67 had CC while 24 had EM. Baseline characteristics were similar. Neonates of women with CC were delivered at a later gestational age [38.2 wks vs 29.9 wks, p < 0.00 1], had higher birth weight [3170 g vs 2295 g, p < 0.001], lower NICU admission rate [18 (27.3%) vs 16 (66.7%), p = 0.001] and lower rate of severe neonatal complications [1 (6%) vs 6 (46.2%), p < 0.010] Table 1. Compared to women with EM, those with CC had 95% lower odds of PTB < 37 weeks [aOR; 0.05, (0.0.14 ‐ 0.19), p < 0.001] and 97% lower risk of early PTB < 34 weeks [aRR; 0.03, (0.01 ‐ 0.15), p < 0.001]. There was no difference in late PTB (34 ‐ 37 weeks) between the groups [aRRR; 0.18, (0.25 ‐ 1.25), p = 0.08] **Table 2**.

**Conclusion**: This study showed that compared to EM, CC in women with CI > 24 weeks reduced the risks of sPTB, prolonged pregnancy latency and reduced NICU admission and severe neonatal complications rates.

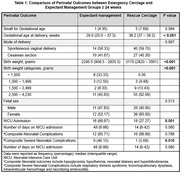

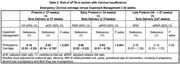

## 1001 | Detecting Maternal Mosaicism in Fetal Sex Chromosome Aneuploidy Screening

C.J. Battey^1^; J. Sherry Wang^1^; Kyle Trettin^1^; Ravi M. Patel^1^; Anu Srinivasan^1^; Janani Saikumar^1^; Divya Kushnoor^1^; Heather Labreche^1^; Carly Hammer^1^; Summer Pierson^1^; Manesha Putra^2^; Genevieve Gould^1^; Dale Muzzey^1^

*^1^Myriad Genetics, Inc., Salt Lake City, UT; ^2^University of Colorado Anschutz Medical Campus, Aurora, CO*

4:00 PM ‐ 6:00 PM

**Objective**: Sex chromosome anomalies (SCA) are a common class of fetal aneuploidy, but cell‐free DNA (cfDNA) screening assays have relatively low positive predictive value (PPV) for SCA calls. False positives are caused by factors including statistical error, confined placental mosaicism, and maternal mosaicism. To compare the impact of these factors and improve SCA screening PPV, we developed a new “depth trajectory” method that identifies mosaic maternal aneuploidy by analyzing covariation in read depth and DNA fragment size.

**Study Design**: We screened 728 prenatal cfDNA samples for fetal SCA with a whole‐genome sequencing (WGS) assay and a new targeted sequencing assay. The targeted assay uses *in silico* size selection to measure how chromosome dosage varies with fetal fraction within each sample's sequencing reads. Maternal mosaic loss of X is identified when X chromosome dosage is depressed but increases along with fetal fraction. The cohort included 25 patients screening positive for monosomy X and 36 patients screening positive for other SCAs on the WGS assay. Fetal diagnosis via amniocentesis or postnatal karyotype was obtained for 12 monosomy X screen‐positive patients.

**Results**: 59 of 61 patients screening positive for fetal SCA in the WGS assay were also positive in the targeted assay prior to maternal anomaly calling. PPV of monosomy X calls in the WGS assay was 42% (5/12). One fetal mosaic true positive was not detected and 3 of 7 false positives were identified as maternal anomalies in the targeted assay, improving PPV to 50% (4/8). Remaining false positives had an average 61% degree of mosaicism in the targeted assay, consistent with placental mosaicism.

**Conclusion**: Depth trajectory analysis successfully identifies maternal X chromosome mosaicism and improves PPV in fetal sex chromosome anomaly screening. We observed high confirmation rates in re‐tested samples, suggesting that statistical error is a relatively minor contributor to SCA false positives, which are primarily explained by confined placental mosaicism and maternal mosaicism.

## 1002 | Incidence of Recurrent Preterm Birth in the US after FDA Withdrawal of 17‐OH‐Progesterone

Claire H. Packer^1^; Taylor S. Freret^2^; Kaitlyn E. James^3^; Sarah E. Little^2^; Alexander Melamed^3^; Mark A. Clapp^3^

*^1^Mass General Brigham, Boston, MA; ^2^Beth Israel Deaconess Medical Center, Boston, MA; ^3^Massachusetts General Hospital, Boston, MA*

4:00 PM ‐ 6:00 PM

**Objective**: For >10 years, 17‐OH‐progesterone (17‐OH‐P) was prescribed to reduce the risk of recurrent spontaneous preterm birth. In 2023, its FDA approval was withdrawn due to a lack of demonstrated efficacy. The objective of this study was to examine population changes in the incidence of recurrent preterm birth after the FDA withdrawal.

**Study Design**: We performed a pre‐post study using cross‐sectional US natality data. Patients with a history of preterm birth and who delivered a singleton, live birth after 20 weeks of gestation were included. FDA withdrawal of approval for 17‐OH‐P was in April 2023. The pre‐period was from 09/2022‐03/2023, and the post‐period from 09/2023‐03/2024; both periods were the same length and comprised the same calendar months. Births from 04/2023‐08/2023 were excluded to avoid patients who potentially discontinued 17‐OH‐P mid‐pregnancy. We examined the incidence of recurrent preterm birth < 37 and< 34 weeks of gestation; results were stratified by race and ethnicity and reported for groups with ≥100 individuals in the denominator. Chi‐square tests were used for comparison.

**Results**: There were 16,088 deliveries in the pre‐period and 16,534 in the post‐period. There were no significant differences in the incidence of preterm birth < 37 weeks (26.7 vs. 26.4%, p = 0.54) or < 34 weeks (6.7 vs 6.7%, p = 1.00) between the pre‐ and post‐periods. When stratified by race, there was no significant increase in preterm births in the post‐period among any of the racial and ethnic groups (Figure).

**Conclusion**: When comparing the periods immediately before and after the FDA withdrawal of its approval for 17‐OH‐P, there were no apparent national differences in the incidences of recurrent preterm birth overall or among racial and ethnic groups. Future studies using individual patient‐level data with more granular details on the etiologies for prior preterm births and accounting for other changes related to recurrent preterm birth management (e.g., vaginal progesterone use) will be important in understanding the population impacts of this major practice change.

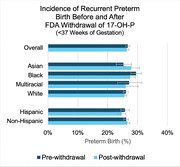

## 1003 | Racial Disparities in Cesarean Deliveries without Trial of Labor: A Population‐Based Analysis of Birth Timing

Clarice G. Zhou^1^; Christopher X. Hong^2^; Mariana Masteling^2^; Matthew K. Janssen^3^; Rebecca F. Hamm^4^

*^1^New York University Langone Health, New York, NY; ^2^University of Michigan, Ann Arbor, MI; ^3^University of Pennsylvania, Pittsburgh, PA; ^4^University of Pennsylvania Perelman School of Medicine, Philadelphia, PA*

4:00 PM ‐ 6:00 PM

**Objective**: While racial disparities in obstetrics are multifactorial, differences in resource allocation may contribute to the disparity. Here, we assess racial differences in delivery timing for cesarean deliveries (CD) without a trial of labor (TOL).

**Study Design**: This retrospective population‐based cohort used birth certificate data from the National Vital Statistics System from 2016‐2021, which includes birth timing for CD in the US. Since the timing of planned CD may be more susceptible to external systemic influences, this analysis focuses on CD without TOL. We excluded CD with TOL and unknown delivery time. Maternal race was self‐identified (White, Black, Asian, American Indian or Alaskan Native, Native Hawaiian or Pacific Islander, or mixed or other race) and categorized as White or Black, Indigenous, and People of Color (BIPOC). The number of CD without TOL at each minute was normalized relative to totals in each racial group to allow for balanced comparison. The cumulative sum in the proportion of CD was calculated starting from 6:00 a.m. Differences in cumulative sum of CD between races were analyzed with the Kolmogorov‐Smirnov test.

**Results**: Among 7,045,802 patients who underwent a CD without TOL, 72% were categorized as White and 26.3% as BIPOC; less than 3% had unknown racial data. For both groups, the highest proportion of CD occurred around 8:00 a.m. followed by a second peak around 12:30 p.m. Differences in the cumulative sum distribution exist between racial groups (*p* = 0.01). The largest difference in the cumulative sum distribution between groups is observed at 10:10 a.m. between White and BIPOC patients; by this time, CD without TOL occurred for 35% of White patients versus only 28% of BIPOC patients.

**Conclusion**: This population‐based study reveals differences in the timing of CD without TOL between White and BIPOC patients, with White patients more likely to undergo CD earlier in the clinical workday. Future research should assess root causes, and whether disparities in resource allocation contribute to differences in obstetrical outcomes.

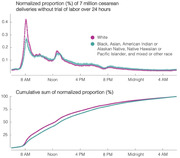

## 1004 | Early Placental Volume as a Biomarker for Maternal Vascular Malperfusion

Claudia J. Tawil^1^; Baris Oguz^2^; Gabriel A. Arenas^2^; Rebecca L. Linn^3^; Jasmine Steele^4^; Ipek Oguz^5^; Nadav Schwartz^1^

*^1^University of Pennsylvania, Perelman School of Medicine, Philadelphia, PA; ^2^University of Pennsylvania, Philadelphia, PA; ^3^The Children's Hospital of Pennsylvania, Philadelphia, PA; ^4^The Children's Hospital of Philadelphia, Philadelphia, PA; ^5^Vanderbilt University, Nashville, TN*

4:00 PM ‐ 6:00 PM

**Objective**: Early placental volume (PV) is associated with fetal growth outcomes, but manual segmentation makes it clinically infeasible. Novel automated techniques have made 3D placental biometry more clinically practical; yet, it is unclear whether PV can distinguish constitutionally small fetuses from affected by placental insufficiency. Such a distinction is critical for developing targeted interventions and improving pregnancy outcomes. We sought to explore whether early PV, measured by an automated segmentation tool, is associated with placental pathology.

**Study Design**: In this prospective, longitudinal cohort study, 3DUS volumes of the placenta were obtained at 11‐16 weeks (V1) and 18‐24 weeks (V2). Automated segmentation tools developed in our lab were utilized to measure PV, which were normalized to gestational age. Placentas were examined by a trained placental pathologist blinded to the PVs. Outcomes included birth weight (BW), birth weight centile (BW%), placental weight (PW), placental weight z‐score, and high grade maternal (MVM) or fetal (FVM) vascular malperfusion on the delivered placenta. Given the sample size, analyses were unadjusted.

**Results**: 72 subjects had a V1PV and available outcomes, 65 of which also had a V2PV. MVM and FVM was identified in 12.3% (n = 8) and 13.9% (n = 9) of placentas, respectively. V1PV (mean: 6.4±1.8 cc/wk) was significantly correlated with BW (mean: 3144.9±548g; p = 0.007) and BW %ile (mean: 42.8±25.6; p = 0.03). V1PV was significantly smaller in cases with MVM (mean: 5.3 vs 6.9cc, p = 0.039). V2PV (mean: 11.3±2.9 cc/wk) was significantly correlated with PW (mean: 463.7±98g; p = 0.02), PW z‐score (mean: –0.5±1.3; p = 0.02), and showed a trend towards association with BW (p = 0.06) and MVM (p = 0.06). **Table**

**Conclusion**: Novel segmentation tools can measure PV in an automated fashion to generate a biomarker of fetal growth outcomes. Our results demonstrate that early PV may be used to identify placental maldevelopment in pregnancy, where early identification can significantly improve patients’ counseling and clinical management.

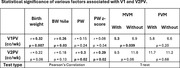

## 1005 | FGF2 Interferes with EGF/EGFR Pathway in Trophoblasts

Colleen Sinnott^1^; Olga Grechukhina^2^; Viktoriia Kolesnyk^2^; Yang Yang‐Hartwich^2^

*^1^Yale University, New Haven, CT; ^2^Yale School of Medicine, New Haven, CT*

4:00 PM ‐ 6:00 PM

**Objective**: Trophoblast migration and invasion are critical to establishment of the healthy placenta, and dysregulation of these processes can result in placental disorders including placenta accreta spectrum (PAS) and preeclampsia (PEC) with high morbidity and mortality. We previously identified fibroblast growth factor‐2 (FGF2) as a likely regulator of trophoblast migration. Further, we suggested that FGF2 may interact with epidermal growth factor (EGF) pathway, another signaling pathway that plays a key role in placenta biology. Here we aim to better characterize specific effects of FGF2 on EGF‐mediated trophoblast function.

**Study Design**: Using immortalized human trophoblast cells (Sw.71), we performed transwell migration assay with human serum from healthy pregnant patients with and without a selective FGF2 neutralizing antibody. Next, trophoblasts were treated with either EGF alone or a combination of both EGF and FGF after which flow cytometry for surface expression of EGF receptor (EGFR) was performed. Western blot (WB) was then conducted to evaluate total EGFR expression after treatment with FGF2 and EGF individually and in combination.

**Results**: Blocking FGF2 in serum collected from healthy pregnant patients resulted in suppressed trophoblast migration. (Figure 1). Trophoblast exposure to EGF resulted in decrease in surface EGFR; however, combination treatment with FGF2 and EGF further decreased surface EGFR compared to treatment with EGF alone (Figure 2A). Similarly, EGF, but not FGF2, solo treatment resulted in decreased total levels of EGFR protein demonstrated by WB (Figure 2B). Combined treatment with FGF2 and EGF resulted in total EGFR expression most similar to EGF treatment alone.

**Conclusion**: FGF2 is not only critical to trophoblast migration but may also play a role in regulating the EGF/EGFR pathway in trophoblasts. The decreased surface expression of EGFR after co‐treatment with both EGF and FGF2 compared to EGF alone suggests that FGF2 may control cell‐surface recycling of EGFR, leaving cells less able to respond to EGF signaling.

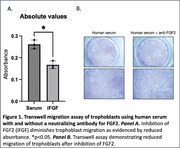

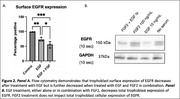

## 1006 | Equity and Perinatal Urine Drug Toxicology Testing by Hospital Location

Cresta W. Jones^1^; Savannah Maynard^1^; Caroline Brumley^1^; Samantha Considine^2^; Emily Biscaye^1^; Katelyn M. Tessier^3^

*^1^University of Minnesota Medical School, Minneapolis, MN; ^2^University of Minnesota Medical Center, Minneapolis, MN; ^3^University of Minnesota, Minneapolis, MN*

4:00 PM ‐ 6:00 PM

**Objective**: To evaluate racial inequities in perinatal urine toxicology via urine drug screen (UDS) by hospital location.

**Study Design**: A retrospective cohort study was conducted examining data from pregnancy episodes for all live births occurring at three hospitals (urban vs. suburban vs. rural) within a single health system between January 1, 2019, and March 31, 2019. We assessed racial differences in urine toxicology testing via a UDS, as well as testing results. Data were compared by hospital location.

**Results**: Among 1368 patients across all hospital locations, 7.3% (n = 99) underwent a UDS before delivery, and 15.2% (n = 208) were tested at the time of delivery. There were significant associations between hospital location and testing rates, with the suburban hospital demonstrating the lowest rate compared to the urban and rural hospitals for testing both before delivery admission (1.9% vs 12.8% vs 14.7%, respectively; p < 0.001), and at delivery admission (11.9% vs 19.1% vs 18.4%, respectively; p = 0.001). There were no significant differences in the rates of UDS with substances detected between the hospital sites regardless of the timing of testing (p = 0.305 and p = 0.386). There were significant differences between UDS rates by identified patient race, and Black birthing patients were three times as likely as White birthing patients to undergo toxicology testing before delivery (15.4% vs 5.6%, p < 0.001) and twice as likely at delivery (25.8% vs 12.3%, p < 0.001).

**Conclusion**: While rates of perinatal substance use across racial groups are similar, Black birthing patients are more likely to undergo urine toxicology evaluation compared to their White birthing counterparts, and this appears to differ by hospital location. Policies and practices of perinatal urine drug testing warrant critical review to limit racial inequities in toxicology testing and to better support patients with perinatal substance use.

## 1007 | Practice Attitudes and Behaviors for Peripartum Management of Attention Deficit Hyperactivity Disorder (ADHD)

Cresta W. Jones^1^; Bukky Akingbola^2^; Jessica Makori^2^; Anwei Gwan^2^; Connor Demorest^3^; Katelyn M. Tessier^2^

*^1^University of Minnesota Medical School, Minneapolis, MN; ^2^University of Minnesota, Minneapolis, MN; ^3^Masonic Cancer Center, University of Minesota, University of Minnesota/Minneapolis, MN*

4:00 PM ‐ 6:00 PM

**Objective**: There is no current standard of care for managing attention deficit hyperactivity disorder (ADHD) in pregnancy. This study explores current practice in peripartum ADHD management among healthcare providers within a single academic health system.

**Study Design**: Prospective Observational ‐ Over a 6 month period (December of 2023 to June of 2024), a 17‐item electronic survey was administered to 198 active women's healthcare professionals within a single academic health system.

**Results**: A total of 75 respondents completed the survey (37.8% response rate). The average years in practice was 13.4 years, and most respondents (95%) had directly cared for a peripartum patient with ADHD. The majority of respondents (78.7%) were unsure if ADHD medications could be safely used during pregnancy/peripartum period. With regards to medication dose prior to attempting pregnancy, most (60%) recommend decreasing to the lowest effective dose, 14.7% recommend no change in medication and 5.3% recommend stopping ADHD medications prior to conception. During pregnancy, 62.7% advised patients to decrease to the lowest effective dose, 17.3% advised patients to discontinue ADHD medications, and only 12% advised patients to stay on their pre‐pregnancy medication dose. For patients continuing ADHD medications during pregnancy, 89.3% of providers recommend changes in obstetric care, including a detailed anatomic survey (62.7%), fetal growth ultrasound surveillance (57.5%), and/or third trimester antepartum surveillance (8%). In the postpartum period, there were significant variations in recommendations for medication treatment while breast/chest feeding.

**Conclusion**: There is significant variability among healthcare providers’ recommendations in the management of perinatal ADHD. We call for a need to align provider behaviors through the development of evidence‐based perinatal ADHD guidelines.

## 1008 | The Impact of Discrimination on Perceived Control Over Birth

Dana R. Canfield^1^; Marni B. Jacobs^2^; Emmanuel Elijah^3^; Karoline Gutierrez^4^; Minhazur R. Sarker^3^; Rachel L. Wiley^3^; Elizabeth Nicole Teal^3^; Cynthia Gyamfi‐Bannerman^3^

*^1^UC San Diego Health, San Diego, CA; ^2^University of California, San Diego Health, San Diego, CA; ^3^University of California, San Diego, San Diego, CA; ^4^University of Miami, Miami, FL*

4:00 PM ‐ 6:00 PM

**Objective**: While studies have linked patient factors with perceived control over delivery, known as labor agentry, and discrimination to poor maternal outcomes, the impact of discrimination on labor agentry is unknown. We examined the association between discrimination, measured by the Everyday Discrimination Scale (EDS), and labor agentry, measured by the Labor Agentry Scale (LAS) a 29‐item Likert‐scale response survey in which a higher score corresponds with greater agentry.

**Study Design**: This a prospective cohort study in which patients were recruited at 27‐34 weeks GA from high‐risk obstetric clinics and completed surveys on health and lived experience, including the EDS. Following birth, they completed the LAS. The cohort was divided into two groups: those who reported experiencing discrimination (EDS≥1, n = 28, 47.5%) and those who did not (EDS = 0, n = 31, 52.5%). Plausible confounders were assessed (Table 1), and linear regression was used to estimate differences in LAS score based on reported discrimination, controlling for significant variables. Correlation analysis was also performed with EDS as a continuous measure calculated as frequency of reported discrimination.

**Results**: 59 patients were included. Patients experiencing discrimination were younger and more frequently preferred a non‐English language. Mean unadjusted LAS was 166.8 (SD 32.9) for patients who did not experience discrimination and 161.1 (SD 23.2) for those who did. Age and preferred non‐English language were included in the final model of discrimination and LAS. None of the factors in our final model were significantly associated with LAS (Table 2). There was a weak but significant negative correlation between EDS score and labor agentry, suggesting as frequency of discrimination increased, labor agentry decreased (ρ = ‐0.28, p 0.03).

**Conclusion**: Patients experiencing discrimination had slightly lower labor agentry when controlling for age and preferred language, with a weak but significant relationship noted between discrimination and agentry. Further studies with larger cohorts are needed to further investigate this relationship.

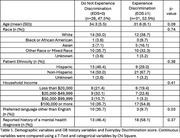

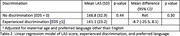

## 1009 | Piperacillin‐Tazobactam versus Ceftriaxone as Initial Antibiotic Treatment in 2nd Trimester Pregnancies Complicated by Acute Pyelonephritis

Daniel J. Martingano^1^; Amanda F. Francis Oladipo^2^; Marwah Al‐Dulaimi^3^; Sandra Kumwong^4^; Andrea Ouyang^5^; Lauren Cue^6^; Ashley Nguyen^3^; Francis X. Martingano^7^; Shailini Singh^8^; Alexander Ulfers^9^; Mark Rebolos^3^; Kristin Cohen^10^; Donald Morrish^3^; Iffath A. Hoskins^11^

*^1^St. John's Episcopal Hospital‐South Shore and William Carey University College of Osteopathic Medicine, Far Rockaway, NY; ^2^Hackensack University Medical Center, Hackensack, NJ; ^3^St. John's Episcopal Hospital‐South Shore, Far Rockaway, NY; ^4^Touro College of Osteopathic Medicine‐Harlem Campus, New York, NY; ^5^William Carey University, Hattiesburg, MS; ^6^Rutgers University and the Jersey City Medical Center, Jersey City, NJ; ^7^NYU Grossman School of Medicine ‐ NYU Brooklyn, New York, NY; ^8^AtlantiCare Regional Medical Center, Pamona, NJ; ^9^Walter Reed National Military Medical Center, Bethesda, MD; ^10^RWJBarnabas Health ‐ Trinitas Regional Medical Center, Elizabeth, NJ; ^11^Albert Einstein College of Medicine ‐ Montefiore Medical Center, New York, NY*

4:00 PM ‐ 6:00 PM

**Objective**: Acute pyelonephritis is one of the most common indications for antepartum hospitalization, yet the optimal antibacterial regimen for the treatment of acute pyelonephritis in pregnancy remains undefined. This study sought compare the effectiveness of piperacillin‐tazobactam versus ceftriaxone as the initial antibiotic treatment in 2^nd^ trimester pregnancies complicated by acute pyelonephritis.

**Study Design**: We conducted a multi‐center, prospective observational study from 7/2022 to 7/2024 and included all pregnant women diagnosed with acute pyelonephritis in the 2^nd^ trimester receiving initial antibiotic treatment with either piperacillin‐tazobactam or ceftriaxone. Choice of regimen was determined by physician preference. Pyelonephritis diagnostic criteria included presence of fever of 38.0°C, costovertebral angle tenderness, and urinalysis suggestive of urinary tract infection. Primary outcomes included inpatient length of stay (LOS), adult respiratory distress syndrome (ARDS), urosepsis, intensive care unit (ICU) admission and preterm labor. Patients were excluded if they received additional antibiotics before admission, had preexisting renal disease, or had an allergy to either medication.

**Results**: The study included 404 patients, with 235 receiving piperacillin‐tazobactam and 169 receiving ceftriaxone. Baseline demographic factors were not significantly different. The causative uropathogen was not significantly different between treatment groups. Patients receiving piperacillin‐tazobactam had lower rates of ICU admissions (5.1% v. 10.7% p = 0.003) and a shorter LOS (2.6 v. 3.1 days, p = 0.041) with a 44% (RR = 0.56, 95% CI 0.31‐0.71, p = 0.004) and 17% (RR = 0.83, 95% CI 0.79‐0.95, p = 0.001) decreased risk in confounder adjusted models, respectively. Patients receiving ceftriaxone had higher rates of ARDS (2.96% v. 0.85%, p< 0.001) with an 18% increased risk in adjusted models (RR = 1.18, 95%CI 1.10‐1.32, p = 0.005).

**Conclusion**: Piperacillin‐tazobactam is a reasonable choice for initial antibiotic treatment for 2^nd^ trimester pregnancies complicated by acute pyelonephritis.

## 1010 | Adequacy of Chorionic Villus Samples in one‐ vs. Two‐Pass Procedures

Daniela A. Febres‐Cordero^1^; Rafaela Germano Toledo^1^; Julia Sroda Agudogo^1^; Annliz Macharia^1^; Anna M. Modest^1^; Yinka Oyelese^2^; Barbara O'Brien^1^

*^1^Beth Israel Deaconess Medical Center, Boston, MA; ^2^Beth Israel Deaconess Medical Center/Harvard Medical School, Boston, MA*

4:00 PM ‐ 6:00 PM

**Objective**: At our center, when chorionic villus sampling (CVS) is performed, the initial pass sample adequacy is assessed immediately by an on‐site cytogenetic lab. If the initial sample is inadequate, a second pass is done. Thus, a two‐pass CVS procedure is a surrogate for specimen adequacy. CVS adequacy may be affected by body mass index (BMI), gestational age (GA), procedure route (transvaginal vs transabdominal), and provider experience. This study assesses risk factors for inadequate samples by comparing one‐pass and two‐pass CVS procedures.

**Study Design**: Retrospective cohort study of patients who underwent CVS at a single tertiary center in 2019‐2023. Data was obtained via medical record review. Cases with an unknown number of placental entries were excluded. Comparisons were made between two groups: adequate villi (one‐pass) and initially inadequate villi (two‐pass). Factors compared were BMI, GA, genetic abnormality presence, placental location, route, needle size, and provider experience. Chi‐square, Fisher's test for categorical data, and Wilcoxon rank sum for continuous data were used for analysis.

**Results**: Of the 408 CVS procedures that met inclusion criteria, 84% were successful after one pass. Maternal BMI was significantly higher in the two‐pass than in the one‐pass group (27.9 and 25.4, respectively, *P =*0.02). There was no difference in GA, placental location, presence of genetic abnormalities, and number of fetuses (Table 1). Fellow years of experience was lower in the two‐pass group (median 2.0 years, IQR 1.0‐2.0) than the one‐pass one (IQR 2.0‐3.0). There was no difference across CVS route, needle size, or attending years of experience (Table 2).

**Conclusion**: Higher maternal BMI and fewer years of fellow experience were associated with a two‐pass procedure, indicating sample inadequacy. Route of procedure, placental location, and genetic abnormalities were not associated with requiring a second pass. Rate of complications was low in both groups. This suggests that when on‐site cytogenetics is unavailable, two‐pass procedures should be considered for high maternal BMI and inexperienced operators.

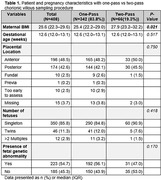

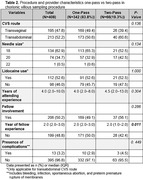

## 1011 | Low Dose Aspirin for Preeclampsia Risk Reduction: Do Patients Actually Take it?

Daniella Rogerson^1^; Marni B. Jacobs^2^; Omar Mesina^1^; Carolina Diaz Luévano^1^; Ayelet Ruppin‐Pham^1^; Maryam Tarsa^3^; Ukachi N. Emeruwa^4^; Cynthia Gyamfi‐Bannerman^4^; Elizabeth Nicole Teal^4^

*^1^University of California San Diego Health, San Diego, CA; ^2^University of California, San Diego Health, San Diego, CA; ^3^University of California San Diego, San Diego, CA; ^4^University of California, San Diego, San Diego, CA*

4:00 PM ‐ 6:00 PM

**Objective**: ACOG, SMFM, and USPSTF recommend low dose aspirin (LDA) in patients at high risk for preeclampsia (PreE). While there is increasing attention to LDA prescription, little is known about LDA uptake and adherence. This study examines LDA uptake and adherence among patients prescribed.

**Study Design**: This is a retrospective cohort study of patients who delivered at an academic medical center from 9/2022 to 7/2024 and were prescribed or recommended LDA for PreE risk reduction. LDA prescription/recommendation was ascertained through the electronic health record and chart review by a quality improvement nurse. Patients not prescribed LDA or for whom prescribing data was unavailable were excluded. The primary outcome was rate of LDA uptake. Secondary outcomes included LDA adherence and associations between sociodemographic characteristics and uptake. Chi‐square and Student's t‐tests were used to compare baseline characteristics and multivariable logistic regression determined strength of association.

**Results**: Among 1,715 patients prescribed LDA, 1,213 (70%) answered questions about LDA uptake and adherence at delivery admission: 953 (79%) reported taking LDA and 260 (21%) did not. Among those who took LDA, 307 (32%) had adherence data available: 246 (80%) reported taking LDA every day, 43 (14%) missed some doses, and 18 (6%) missed many doses. Patients who took LDA were older and had higher BMI than those who did not (p >0.05, Table 1). Compared to White patients, patients who identified as Black and/or African American had lower odds of LDA uptake in an unadjusted analysis and when adjusting for maternal age and BMI (OR 0.55, 95% CI 0.34–0.87 and aOR 0.54, 95% CI 0.30–0.96, Table 2). There was no association between language or insurance and uptake.

**Conclusion**: Most patients prescribed LDA for PreE risk reduction took the medication and reported good adherence. However, Black patients indicated lower LDA uptake than White patients. Further studies examining LDA uptake/adherence are needed to understand the root causes of these disparities and identify solutions.

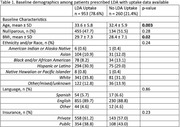

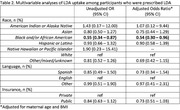

## 1012 | Induction Versus Expectant Management in Multiparous Low‐Risk Women: Single Institution Impact of the ARRIVE Trial

Kathleen M. Pulice^1^; Shekinah Dosunmu^2^; Joanne N. Quiñones^2^; Meredith Rochon^2^; Amanda B. Flicker^2^; Travis Dayon^2^; George Angelakakis^3^; Janae Cornwall^3^; Ahsan Usmani^3^; Danielle Durie^2^

*^1^Lehigh Valley Health Network, Macungie, PA; ^2^Lehigh Valley Health Network, Allentown, PA; ^3^University of South Florida Morsani College of Medicine, SELECT Program, Tampa, FL*

4:00 PM ‐ 6:00 PM

**Objective**: The ARRIVE trial found that elective induction of labor (eIOL) at 39 weeks in nulliparous persons compared to expectant management was associated with a lower rate of cesarean delivery (CD). The benefit of eIOL in a multiparous population is less certain. Our goal was to evaluate the performance of low‐risk multiparous women undergoing eIOL at 39 weeks.

**Study Design**: Retrospective cohort study of all multiparous pregnant persons admitted for delivery ≥ 39w0d at a single academic community institution between 3/1/20‐3/1/22, when we started offering eIOL. Those with singleton low risk pregnancies, without any clinical indication for delivery prior to 40w5d, were included. Maternal and neonatal outcomes of persons undergoing eIOL between 39w0d‐39w4d were compared to women expectantly managed. The primary outcome was rate of CD. Secondary outcomes included select maternal outcomes and composite neonatal outcome (neonatal death and/or serious morbidity).

**Results**: 1141 multiparous persons with low‐risk singleton gestations were identified, with 297 (26%) undergoing eIOL and 844 (74%) expectantly managed. Those undergoing eIOL delivered earlier (39.2 vs 40.0, p< 0.001) and were more likely to be obese (34.4 vs 23.7%, p< 0.001) and have diet controlled gestational diabetes (10.1 vs 6.4%, p = 0.04) (Table). The CD rate was similar between the eIOL and expectant management groups (2.7% vs 2.0%, p = 0.49). Elective IOL was associated with longer maternal but similar newborn lengths of stay compared to expectant management. The composite neonatal outcome occurred in significantly fewer neonates in the eIOL group compared to the expectant management group (4.0% vs 8.3%, p = 0.02), primarily due to decreased need for respiratory support in the eIOL group (1.7% vs. 4.5%, p = 0.03).

**Conclusion**: In our cohort of low‐risk multiparous pregnant persons, eIOL at 39w0d‐39w4d did not decrease CD rate but did decrease composite neonatal risk. Like the findings of ARRIVE trial in a nulliparous population, our findings suggest that eIOL at term for multiparous patients is safe and may have benefit.

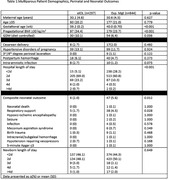

## 1013 | The Association Between First Trimester Serum Creatinine and Pregnancy Outcomes in Patients with Chronic Hypertension

Danielle N. Olson^1^; Alyssa Wolfinger^2^; Kelly S. Gibson^2^

*^1^MetroHealth Medical Center / Case Western Reserve University, Cleveland Heights, OH; ^2^MetroHealth Medical Center / Case Western Reserve University, Cleveland, OH*

4:00 PM ‐ 6:00 PM

**Objective**: Pre‐existing hypertension (HTN) and renal disease are known risk factors for adverse pregnancy outcomes, including pre‐eclampsia (PreE). This study aimed to assess whether an elevated first trimester serum creatinine (sCr) in patients with chronic HTN, in the absence of pre‐existing kidney disease, is associated with an increased risk of PreE.

**Study Design**: This was a retrospective cohort study at a single tertiary center from 1/2018 to 12/2022. Inclusion criteria were patients with HTN with a first trimester sCr result. Exclusion criteria were pre‐existing kidney disease, significant proteinuria in the first trimester, acute illness during sCr evaluation, and conditions known to increase the risk for adverse pregnancy outcomes including pregestational diabetes, autoimmune disease, multiple gestation, history of renal surgery, transplantation, donation, and isolated renal agenesis. An elevated sCr was defined as >0.7 mg/dL. The primary outcome was the diagnosis of PreE. Secondary outcomes included assessment of first trimester sCr in patients diagnosed with PreE, gestational age at PreE diagnosis and at delivery, small for gestational age (SGA), and gestational diabetes (GDM). Wilcoxon rank sum tests and Chi‐squared tests were used.

**Results**: 226 patients with normal sCr and 76 with elevated sCr were identified. 82/226 (36%) in the normal sCr group developed PreE, compared to 36/76 (47%) in the elevated sCr group (p = 0.11). First trimester sCr as a continuous variable was associated with an increased incidence of PreE diagnosis (p = 0.03). Gestational age at PreE diagnosis (35.2±3.4 v 35.1±3.6w, p = 0.845), gestational age at delivery (37.3±2.7 v 36.7±3.1w p = 0.045), SGA (27 v 32% p = 0.534), and GDM (15 v 20% p = 0.494) were not different between groups.

**Conclusion**: In the population studied, a first trimester sCr >0.7 mg/dL was not associated with an increased incidence of PreE, though this was associated with earlier gestational age at delivery. Patients who developed PreE had higher sCr in the first trimester on average than those who did not develop PreE.

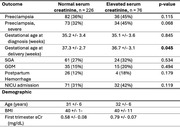

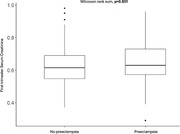

## 1014 | Adverse Perinatal Outcomes Based on Gestational Surrogacy Status in a Large Integrated Healthcare System

Fagen Xie^1^; Theresa M. Im^1^; Daniella Park^1^; Vicki Y. Chiu^1^; Michael J. Fassett^2^; Darios Getahun^1^

*^1^Kaiser Permanente Southern California, Department of Research & Evaluation, Kaiser Permanente Southern California/Pasadena, CA; ^2^Kaiser Permanente West Los Angeles Medical Center, Department of Obstetrics & Gynecology, Kaiser Permanente West Los Angeles Medical Center/Los Angeles, CA*

4:00 PM ‐ 6:00 PM

**Objective**: To examine adverse perinatal outcomes by gestational surrogacy status in a large integrated healthcare system in the United States.

**Study Design**: We performed a retrospective cohort study among pregnant individuals receiving obstetrical care at Kaiser Permanente (KP) Southern California (01/01/2008–12/31/2023). Unstructured clinical notes abstracted from electronic health records were used to determine surrogate pregnancy status via natural language processing (NLP) validated by manual chart reviews. Births at < 20 weeks gestation were excluded from all analyses. Adjusted risk ratios (aRR) and 95% confidence intervals (CI) derived from robust Poisson regression described the associations.

**Results**: Among 636,300 pregnancies during the study period, 1,109 (0.17%) were identified as surrogate pregnancies. Compared to those with non‐surrogate pregnancy, people with gestational surrogacy were more likely to be older (32.4% versus 19.4%), non‐Hispanic white (39.0% versus 23.8%), have privately funded KP membership (19.8% versus 7.6%), and have multiple gestations (15.8% versus 1.6%). They were also less likely to smoke (0.7% versus 2.4%) and consume alcohol (19.0% versus 27.1%) during pregnancy. Gestational surrogacy was associated with significantly increased risk of placenta previa (aRR: 1.74, 95% CI: 1.44‐2.10), placental abruption (aRR: 2.25, 95% CI: 1.45‐3.50) and preterm birth (aRR: 1.27, 95% CI: 1.13‐1.43) while with significantly decreased risk of small for gestational age/intrauterine growth restriction (aRR: 0.80, 95% CI: 0.69‐0.93), premature rupture of membranes (aRR: 0.72, 95% CI: 0.53‐0.98), gestational diabetes (aRR: 0.84, 95% CI: 0.72‐0.98) and chorioamnionitis (aRR: 0.58, 95% CI: 0.40‐0.86) (Table).

**Conclusion**: This study successfully leveraged unstructured data to identify surrogate pregnancies via NLP in a large integrated healthcare system. Placenta previa, placental abruption, and preterm birth were found to be associated with gestational surrogate pregnancy. Identifying these risk factors can provide empirical evidence for predicting and preventing adverse perinatal outcomes.

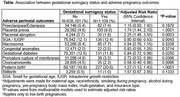

## 1016 | How to Efficiently Recruit Participants in a Perinatal Cohort Study; CAN‐B Cohort Strategies/Lessons Learned

Dhyana Kpegba^1^; Virginie Gillet^2^; Virginie Bouchard^2^; Claudia Lugo‐Candelas^3^; Jonathan Posner^4^; Annie Ouellet^2^

*^1^University of Sherbrooke, Sherbrooke, PQ; ^2^Université de Sherbrooke, Sherbrooke, PQ; ^3^Columbia University, New York, NY; ^4^Duke University, Durham, NC*

4:00 PM ‐ 6:00 PM

**Objective**: Recruitment processes in perinatal cohort remain challenging, particularly when a specific population such as cannabis consumers is involved. The CAN‐B study aims to determine the impact of prenatal cannabis exposure on fetal brain and infant neurodevelopment with longitudinal follow‐up of mother/infant from pregnancy until 2y post‐natal. Here we share our experience in finding the best recruitment strategy to meet our enrollment objective.

**Study Design**: From May 2022 to May 2024, at the routine 1^st^ trimester scan at the obstetrical ultrasound unit of Sherbrooke University Hospital Center (CHUS), 4 recruitment strategies were successively tried: 1) study flyer given by care providers (May‐September 2022), 2) by phone (October‐November 2022),  3) study flyer given by care providers or part time in‐person presentation of the study by research team (December 2022‐May 2023) and  4) full time in‐person presentation of the study by research team (June 2023 to May 2024). Interest and enrollment rate were compared.

**Results**: 112 pregnant women were enrolled (71 cannabis users/41 controls). Strategy 4 triggered the highest basic interest to participate in a clinical research compared to the other strategies (87% vs < 64%) and yielded the highest enrollment rate (80%) compared to strategies 1(67%), 2 (40%) and 3 (70%). Ultimately, the last strategy with full time direct personalized approach allowed us to double our monthly enrollment rate of cannabis users. Same strategies performances were observed for the recruitment of controls.

**Conclusion**: Recruitment of cannabis users in the CAN‐B cohort showed that low‐cost passive strategies (e.g. flyers) or relying only on care providers appeared to be much less efficient and not cost‐effective. Although costly, direct personal contact by research professional integrated into routine care is the best strategy to prioritize for recruiting pregnant women. This experience provides useful information for clinical researchers in designing perinatal cohorts.

## 1017 | Practice Patterns in the Administration of Late Preterm Antenatal Corticosteroids Among Twin Gestations

Diana S. Abbas^1^; Dana S. Berger^1^; Lindsay N. Marty^1^; Kate Tolleson^1^; Meghana A. Limaye^2^; Justin S. Brandt^2^

*^1^NYU Grossman School of Medicine, New York, NY; ^2^NYU Langone Health, New York, NY*

4:00 PM ‐ 6:00 PM

**Objective**: Predicting preterm delivery is challenging, especially among twin pregnancies. We sought to evaluate the odds of delivering twin pregnancies within 2‐7 days of antenatal corticosteroid (ACS) administration in the late preterm period (optimally timed), by delivery indication.

**Study Design**: This is a retrospective cohort study of twin pregnancies at risk for late preterm delivery (Jan 2013‐Dec 2022) performed at a large tertiary care health system. Patients were included if they had non‐anomalous twin gestations and received at least one dose of ACS in the late preterm period (34 0/7‐ 36 6/7 weeks). They were excluded if they received ACS previously or had pre‐gestational diabetes. The cohort was stratified into two exposure groups: patients at risk for spontaneous preterm birth (sPTB; preterm labor or preterm pre‐labor rupture of membranes) and patients at risk for clinician initiated late PTB (i.e., hypertensive disorders of pregnancy). The primary outcome was optimally timed ACS administration. Secondary outcomes included median time from ACS administration to delivery, delivery at < 2 days and > 7 days, and PTB. Multivariable logistic regression was used to calculate odds ratios (aORs) of the primary and secondary outcomes, adjusted for potential confounders.

**Results**: Of 166 eligible patients, 30 (18.1%) delivered within 2‐7 days. 86 (51.8%) were at risk for sPTB, and 80 (48.2%) were at risk for a clinician initiated late PTB. Group demographics are described in Table 1. Compared to patients at risk for sPTB, those with a clinician initiated late PTB were more likely to have optimally timed administration (4.6% vs 32.5%; aOR 9.2, 95% CI 3.2, 33. 9). Patients at risk for sPTB were more likely to deliver < 2 days (aOR 0.19, 95% CI 0.08, 0.42), with a shorter latency to delivery (7.0 vs 45.7 hours) (Table 2).

**Conclusion**: In this cohort of twin gestations at risk for late PTB, those at risk for clinician initiated late PTB were more likely to deliver within the optimal time period compared to those at risk for sPTB. Further study is warranted to evaluate factors associated with delivery timing.

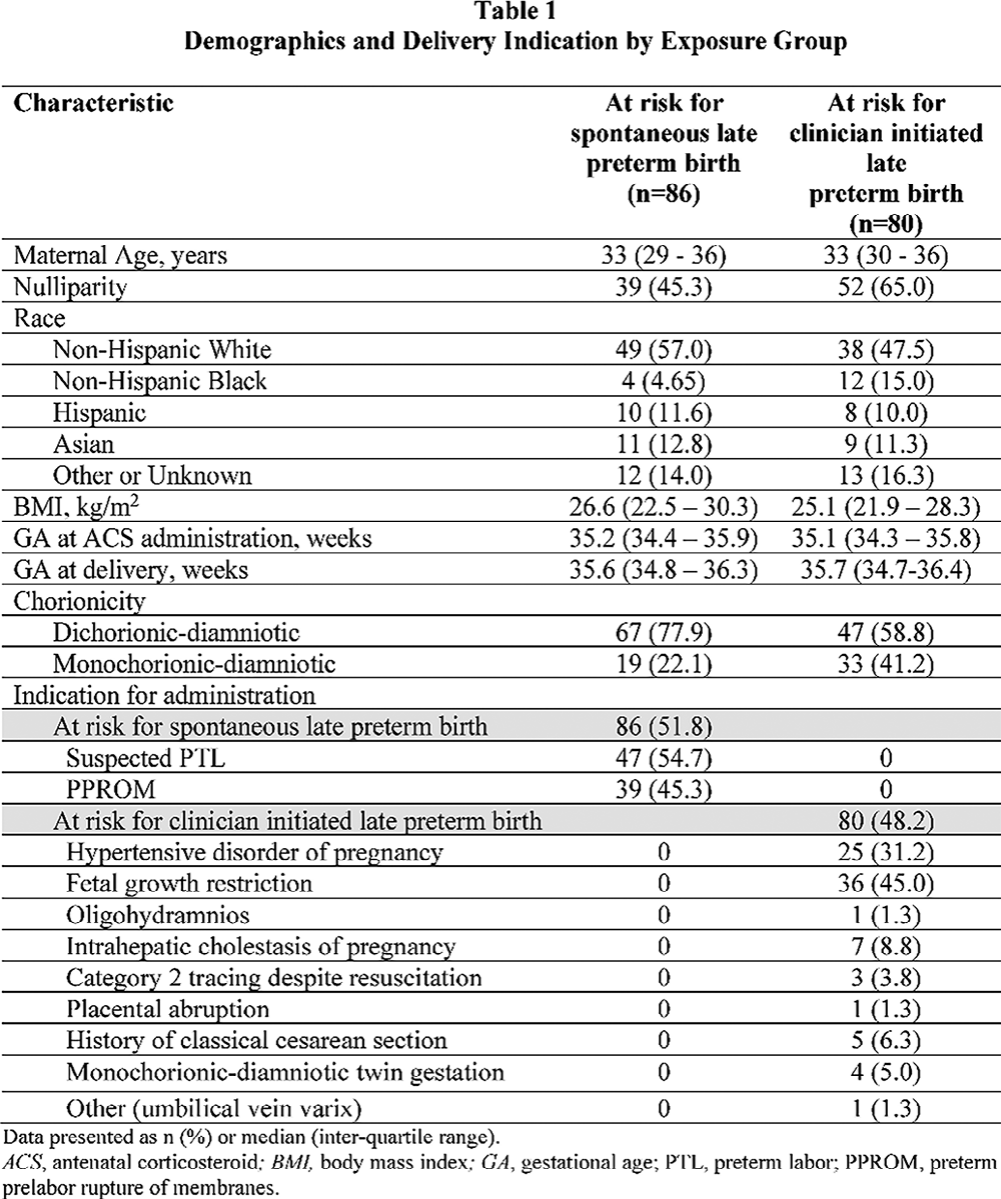

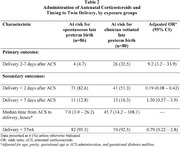

## 1018 | Risk for Adverse Perinatal Outcomes following Induction of Labor in Patients with Class III Obesity

Easha Patel^1^; Kirat Sandhu^1^; Cara D. Dolin^1^; Stacey Ehrenberg^1^; Maeve Hopkins^1^; Adina R. Kern‐Goldberger^2^

*^1^Cleveland Clinic Foundation, Cleveland, OH; ^2^Cleveland Clinic Lerner College of Medicine, Cleveland, OH*

4:00 PM ‐ 6:00 PM

**Objective**: Maternal obesity is a known risk factor for adverse maternal and perinatal outcomes. This study evaluates risk for these pregnancy complications among patients with class 3 obesity undergoing induction of labor (IOL).

**Study Design**: This is a retrospective cohort study of nulliparous, singleton deliveries with BMI > 40 following induction of labor > 20 weeks at a multi‐hospital academic health system from 1/1/2022‐6/30/2024. The primary outcome was unplanned cesarean and secondary outcomes included severe maternal morbidity (SMM) without transfusion, postpartum hemorrhage, postpartum infection, postpartum length of stay > 5 days, maternal readmission, and NICU admission. Maternal clinical characteristics and outcomes were extracted from the medical record and compared in univariable analyses. The association between class 3 obesity and primary and secondary outcomes was evaluated using a logistic regression model based on significance from univariable analyses.

**Results**: 6,533 nulliparous patients undergoing IOL were included, 3.4% of whom had class 3 obesity. Numerous comorbidities and outcomes were different based on class 3 obesity status, including significantly higher rates of pregestational and gestational diabetes and chronic and gestational hypertension, but not severe preeclampsia (Table 1). Unadjusted incidence of NICU admission was twofold higher for patients with class 3 obesity (16.8 v. 8.4%, p < 0.01). Adjusted analysis demonstrated significantly increased odds of cesarean in nulliparous patients undergoing IOL with class 3 obesity compared to those without (aOR 1.97, 95% CI 1.51–2.56, p < 0.01), but for none of the secondary outcomes other than hemorrhage, which had significantly lower odds in class 3 obesity (Table 2).

**Conclusion**: Nulliparous patients with class 3 obesity undergoing IOL have significantly higher risk of cesarean than those with lower BMI, but not for other adverse outcomes. Further study of predictors of successful vaginal delivery for patients with class 3 obesity is important.

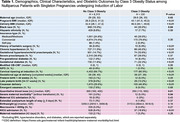

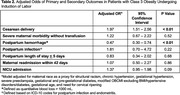

## 1019 | Mediastinal Shift Angle and its Association with Perinatal Mortality and Lung Volumes in CDH Fetuses

Edgar A. Hernandez‐Andrade^1^; Donatella Gerulewicz Vannini^2^; Katrina S. Hughes^3^; Sami Backley^4^; Jerrie Refuerzo^1^; Jimmy Espinoza^4^; Eric P. Bergh^5^; Percy N. Pacora‐Portella^6^; Gustavo Vilchez^7^; Ramesha Papanna^1^; Anthony Johnson^8^

*^1^McGovern Medical School at UTHealth Houston, Houston, TX; ^2^University of Texas Health Science Center in Houston, McGovern Medical School, Houston, TX; ^3^McGovern Medical School. UThealth Houston, Houston, TX; ^4^Division of Fetal Intervention, Department of Obstetrics, Gynecology and Reproductive Sciences, McGovern Medical School at UTHealth Houston, Houston, TX; ^5^UT Health, Houston, TX; ^6^Division of Fetal Intervention, Dpt Obstetrics,Gynecol and Reprod Sciences, McGovern Medical School at UTHealth Houston, Houston, TX; ^7^UTHealth Houston, Houston, TX; ^8^McGovern Medical School University of Texas Health Science Center at Houston (UThealth), Houston, TX*

4:00 PM ‐ 6:00 PM

**Objective**: The mediastinal shift angle (MSA) has been proposed as a potentially useful marker for the identification of CDH fetuses at risk of perinatal death. We aimed to evaluate the predictive performance of MSA for perinatal mortality in fetuses with isolated left CDH, particularly among those considered as mild and moderate with an O/E LHR (observed/expected lung‐to‐head ratio) >25%, and its correlation with other metrics of CDH severity.

**Study Design**: MSA was measured in a cross‐sectional image of the fetal thorax in 84 fetuses with left CDH. MSA was obtained from two lines starting from a common point in the skin at the midline of the back of the spine, the first line dividing the thorax into two halves, and a second line directed to the lateral border of the right atrium (Fig 1). The MSA becomes wider as the right atrium is displaced towards the right side of the thorax. Additionally, the O/E LHR and MRI assessment of O/E total fetal lung volume (TFLV), and percentage of liver herniation (%LH) were evaluated. ROC analysis, prediction, and association with perinatal mortality and correlations with the mentioned predictors were evaluated

**Results**: Perinatal mortality was 31.3% (26/83). The areas under the ROC curve for perinatal mortality for MSA and O/E LHR were 0.694 and 0.753, respectively (p = 0.2). MSA best cut‐off value was 35° with 77% sensitivity, and 38% 1‐specificity; OR 6.68 (95% CI 2.2‐21.3; p < 0.001), adjusted aOR 8.9 (95% CI 1.2‐59.7; p = 0.02). Among fetuses with O/E LHR > 25% (n = 60) mortality was 21% (n = 13), MSA ≥ 35° showed an AUROC = 0.691 with 75% sensitivity and 36% 1‐specificity, aOR 5.29 (1.47‐19.01; p = 0.01). There was a significant correlation between MSA and O/E LHR (‐0.48, p < 0.001), O/E TFLV (‐0.36, p = 0.005), and %LH (0.43, p = 0.001).

**Conclusion**: The MSA angle is a technically simple and feasible metric to evaluate severity and risks for  perinatal mortality in left‐sided CDH cases. An MSA ≥ 35° can identify 77% of cases at increased risk of perinatal mortality. The prediction performance is similar among fetuses with moderate or mild CDH

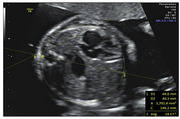

## 1020 | Risk of Spontaneous PTB < 34 weeks Among Women with Mid‐Trimester Cervical Length >25 mm

Edgar A. Hernandez‐Andrade^1^; Donatella Gerulewicz Vannini^2^; Eleazar E. Soto^1^; Sean C. Blackwell^1^

*^1^McGovern Medical School at UTHealth Houston, Houston, TX; ^2^University of Texas Health Science Center in Houston, McGovern Medical School, Houston, TX*

4:00 PM ‐ 6:00 PM

**Objective**: There is a general assumption that the longer the cervix the lower the rate of spontaneous preterm birth (SPTB). This study aimed to test this hypothesis in low‐risk and high‐risk pregnant women with normal cervical length.

**Study Design**: Transvaginal cervical length (TVCL) was measured in 3,376 singleton pregnancies with no fetal anomalies at 17‐24 weeks of gestation. Patients were grouped as low (LR) and high risk (HR) based on history of SPTB. TVCL measurements were analyzed as < 15 mm and < 25 mm, and then in groups of 5 mm from 25‐ 55 mm. SPTB was considered as ≤ 34 weeks of gestation not due to maternal or fetal indication such as placenta accreta, preeclampsia, or fetal growth restriction. Differences in the prevalence of SPTB among the CL groups and between LR and HR patients were evaluated.

**Results**: 2,863 (84.9%) patients were considered LR, and 513 (15.1%) HR. Short cervix (≤ 25 mm) was found in 3.0% of women (n = 103; LR n = 82 (2.8%); HR n = 21 (4.1%)). Prevalence of SPTB was 6.6% (n = 189; LR 4.0% n = 115, HR 14.4% n = 74; p< 0.001). Figure 1 shows the prevalence of SPTB ≤ 34 weeks according to the TVCL groups in LR and HR patients. The prevalence of SPTB was significantly higher in HR patients at any TVCL group than for LR patients. Among LR women, the risk of SPTB did not significantly change when TVCL was ≥ 30 mm, yet LR patients with TVCL between 25.1 and 30.0 mm had a higher risk of SPTB ≤ 34 weeks as compared to those with TVCL > 30.1 mm (p = 0.02). In HR patients the prevalence of SPTB did not significantly change when TVLC was >25 mm.

**Conclusion**: In our population, the risk of SPTB ≤ 34 weeks does not substantively increase once TVCL ≥ 30 mm for LR women and once > 25 mm in HR women. HR women had almost 3 times higher rate of SPTB than LR patients at any TVCL cut‐off.

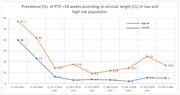

## 1021 | Are Standard Video Sweeps During Fetal Ultrasound Examination more Effective for Cardiac Screening?

Elena D'Alberti^1^; Olga Patey^2^; Lawrence Impey^2^; Christos Ioannou^2^; Angelo Cavallaro^2^; Aris T. Papageorghiou^2^

*^1^Università di Roma Sapienza, Roma, Lazio; ^2^University of Oxford, Oxford, England*

4:00 PM ‐ 6:00 PM

**Objective**: To compare workflow properties of standardized ultrasound video clips performed as sweeps from the four‐chamber [4C] view to the three‐vessel‐trachea [3VT] view, to the still images and videos acquired during the routine fetal cardiac screening (FCS).

**Study Design**: An observational study was conducted at the John Radcliffe Hospital, Oxford. Singleton fetuses with normal cardiac anatomy at the routine 18^+0^ to 23^+0^ weeks anomaly scan were included. The available stored grey‐scale images, videos and sweeps were extracted from scans performed by seven sonographers. We evaluated the number of records collected per fetus, time spent for data acquisition and the computer memory needed for storing these, comparing FCS and standardized sweeps. For the former, the order of view acquisition and the prevalence of cardiac workflow interrupted by extra‐cardiac planes was also assessed.

**Results**: A total of 100 studies were included. FCS included solely still images in 17% of cases, but in 83% videos were used to complete cardiac assessment, mostly cine‐4C alone or 4C and left ventricular outflow tract (LVOT) views. The mean number of images or videos in the FCS group were 10 ± 3, compared to 2 ± 1 in standardized sweeps. In 47% of cases, FCS data‐acquisition was interrupted by extra‐cardiac records, with a median amount of time spent in heart examination of 285 seconds (interquartile range (IQR) 94‐726), compared to 4 seconds (IQR 3‐8) needed to perform sweeps. Median memory required to store FCS data and sweeps were 167 MB (IQR 60‐257) and 123 MB (IQR 76‐165), respectively.

**Conclusion**: Standardized cardiac sweeps represented a more effective way to acquire and store data compared to the FCS; less time was spent on image acquisition, which was not interrupted by extra‐cardiac views, and lower storage memory was required. Further studies are needed to assess the two methods, especially in the presence of fetal congenital heart defects.

## 1022 | Optimizing Timing of Intravenous Iron Therapy for Improved Outcomes in Pregnant Women with Iron‐Deficiency Anemia

Ellen M. Murrin^1^; Olivia LeBeau^2^; Lillian Singer^2^; Mark Kassab^2^; Peyton Kalan^2^; McKenna Stidham^3^; Scott Sullivan^2^; G. Larry Maxwell^2^; George R. Saade^4^; Antonio F. Saad^5^

*^1^Inova Fairfax Medical Campus, Falls Church, VA; ^2^Inova Fairfax Medical Campus, Fairfax, VA; ^3^University of Virginia College of Medicine, Charlottesville, VA; ^4^Macon & Joan Brock Virginia Health Sciences Eastern Virginia Medical School at Old Dominion University, Norfolk, VA; ^5^Inova Health, Falls Church, VA*

4:00 PM ‐ 6:00 PM

**Objective**: To evaluate the efficacy and timing of intravenous (IV) iron therapy in improving hemoglobin (Hgb) levels and maternal and neonatal outcomes in pregnant women with iron‐deficiency anemia (IDA).

**Study Design**: A retrospective cohort study was conducted on pregnant women with IDA who received outpatient IV iron therapy from January 2017‐ June 2024. Patients were divided into those who received their last IV iron dose >10 days (N = 331) or < 10 days (N = 82) before delivery. Primary outcomes included changes in Hgb levels, maternal composite outcomes (including postpartum hemorrhage and blood transfusion), and neonatal outcomes (such as NICU admission and low birth weight).

**Results**: Demographics and characteristics did not differ between groups. The type of IV iron used was primarily iron sucrose (>86.31%). The >10 days group showed a greater increase in Hgb levels post‐infusion, with a median increase of 2.5 g/dL compared to 1.8 g/dL in the < 10 days group (p< 0.001). The maternal composite outcome rate was significantly lower in the >10 days group (7.14% vs. 14.63%, p = 0.03). Neonatal outcomes, including NICU admissions (7.46% vs. 8.54%, p = 0.7) and low birth weight (3.97% vs. 1.61%, p = 0.4), did not differ between the groups.

**Conclusion**: IV iron therapy improves hemoglobin levels in antenatal IDA. Administering the last dose of IV iron more than 10 days before delivery is associated with better maternal outcomes, suggesting that earlier treatment may reduce the risk of adverse pregnancy outcomes. Further studies are needed to refine treatment protocols and timing of administration to maximize benefits.

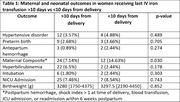

## 1023 | Ureaplasma Colonization in Preterm Premature Rupture of Membranes: Implications for Latency Period and Neonatal Outcomes

Ellen M. Murrin^1^; Sebastian Nasrallah^2^; Helen Havens Howell^3^; Amber Bowman^3^; Antonio F. Saad^4^; Scott Sullivan^2^

*^1^Inova Fairfax Medical Campus, Falls Church, VA; ^2^Inova Fairfax Medical Campus, Fairfax, VA; ^3^University of Virginia College of Medicine, Charlottesville, VA; ^4^Inova Health, Falls Church, VA*

4:00 PM ‐ 6:00 PM

**Objective**: Ascending microbial invasion is thought to be responsible for many cases of preterm premature rupture of membranes (PPROM). Ureaplasma colonization has been observed in up to 65% of cases and may result in poorer neonatal outcomes. We aimed to assess the prevalence of vaginal colonization with Ureaplasma species in pregnancies complicated by PPROM and determine its correlation with the latency period and pregnancy outcomes.

**Study Design**: Demographics, pregnancy complications, treatment regimens, and outcomes were analyzed from 165 patients with PPROM and vaginal Ureaplasma testing from 2017‐2024 at a tertiary care center. Chi‐square, t‐test, Wilcoxon rank sum tests, and logistic regression were used to assess Ureaplasma colonization effects.

**Results**: Ureaplasma was detected in 57.6% of PPROM patients. Baseline characteristics with significant variations between groups were adjusted for, including advanced maternal age, in vitro fertilization, history of cesarean section, cerclage, and mycoplasma positivity. There was no significant difference in latency periods between Ureaplasma‐positive and negative groups (6 [2‐12] vs. 5 [2‐16] days, p = 0.9).

After adjusting for significant differences in characteristics, Ureaplasma colonization was associated with reduced odds of intubation (aOR 0.32, 95% CI 0.15–0.71) and surfactant use (aOR 0.34, 95% CI 0.14–0.79), but did not significantly affect the composite neonatal morbidity (aOR 0.82, 95% CI 0.32–2.10; Table 1). Incidence of preterm birth at < 34 weeks and < 28 weeks (Table 1) and maternal outcomes (Table 2) did not differ between groups after adjusting for characteristics differences.

**Conclusion**: Ureaplasma colonization was common in PPROM patients, however its presence did not significantly affect the latency period. It was associated with specific neonatal outcomes, such as a decreased requirement for neonatal respiratory support. This association suggests the need for further research into the effects of Ureaplasma in pregnancy and the interaction with microbial therapy.

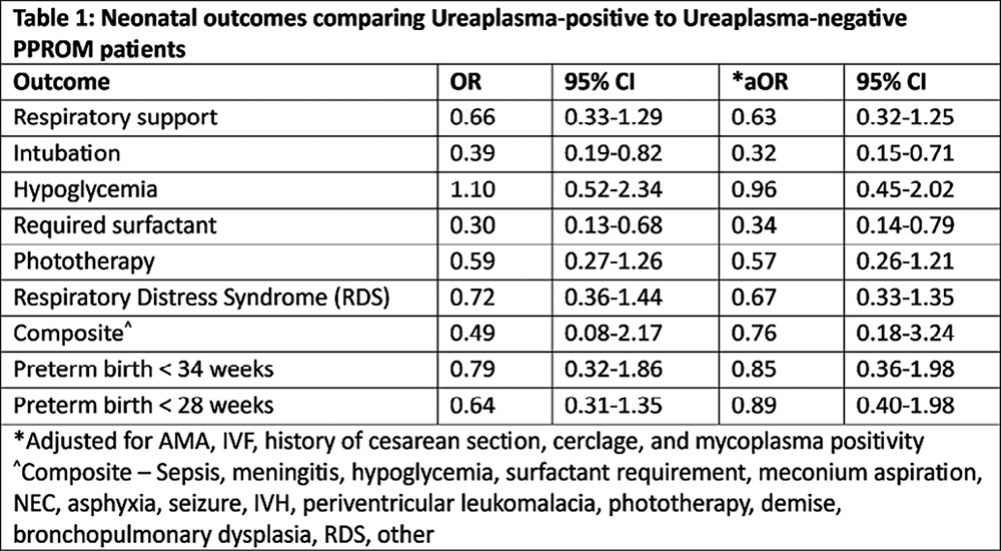

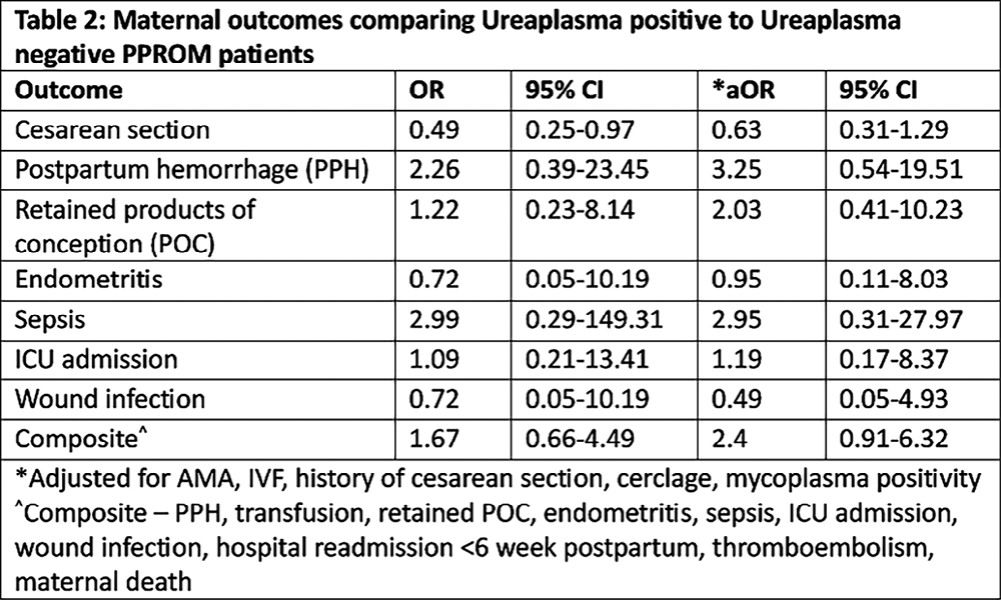

## 1024 | Comparing Inferior Vena Cava Collapsibility in Postpartum Patients with and without Severe Preeclampsia

Elsa Parra^1^; Kerri Brackney^2^; Lauren Piersall^2^; Michael VanDillen^2^; Paul J. Wendel^2^; Angela Nakahara^2^; Meyer L. Norman^2^; Patricia Goedecke^2^

*^1^University of Tennessee Health Science Center Memphis, Memphis, TN; ^2^University of Tennessee Health Science Center, Memphis, TN*

4:00 PM ‐ 6:00 PM

**Objective**: The aim of this study was to determine if there is a difference in the inferior vena cava collapsibility index (IVCCI) between postpartum patients with severe preeclampsia versus those without preeclampsia.

**Study Design**: This was a prospective cohort study conducted in an urban university teaching hospital from May 2024 to June 2024. Patients had singleton or twin gestations and were evaluated within 48 hours of delivery. Cases had severe features of preeclampsia and controls did not have preeclampsia. Patients who previously received furosemide, were hemodynamically unstable, or who had a postpartum hemorrhage were excluded. Inferior vena cava (IVC) diameter was measured at inspiration (IVCmin) and expiration (IVCmax) using transabdominal ultrasound with a curvilinear 1.5‐5 Hz probe. The IVCCI was calculated ((IVCmax‐IVCmin)/IVCmax). The primary outcome was the IVCCI in each group.

**Results**: Amongst cases, the median IVCCI was 0.27 (interquartile range = 0.14 to 0.37) while controls had a mean IVCCI of 0.28 (interquartile range = 0.19 to 0.37). There was no significant difference in the collapsibility index between those with severe preeclampsia and those without preeclampsia.

**Conclusion**: The IVCCI is an objective measure of intravascular volume. It was not shown to have a statistically significant difference between those with and without preeclampsia with severe features. A larger study cohort could establish a better understanding of IVC behavior in these two populations, and thus may provide a useful tool to guide fluid management and diuresis of postpartum patients with severe preeclampsia.

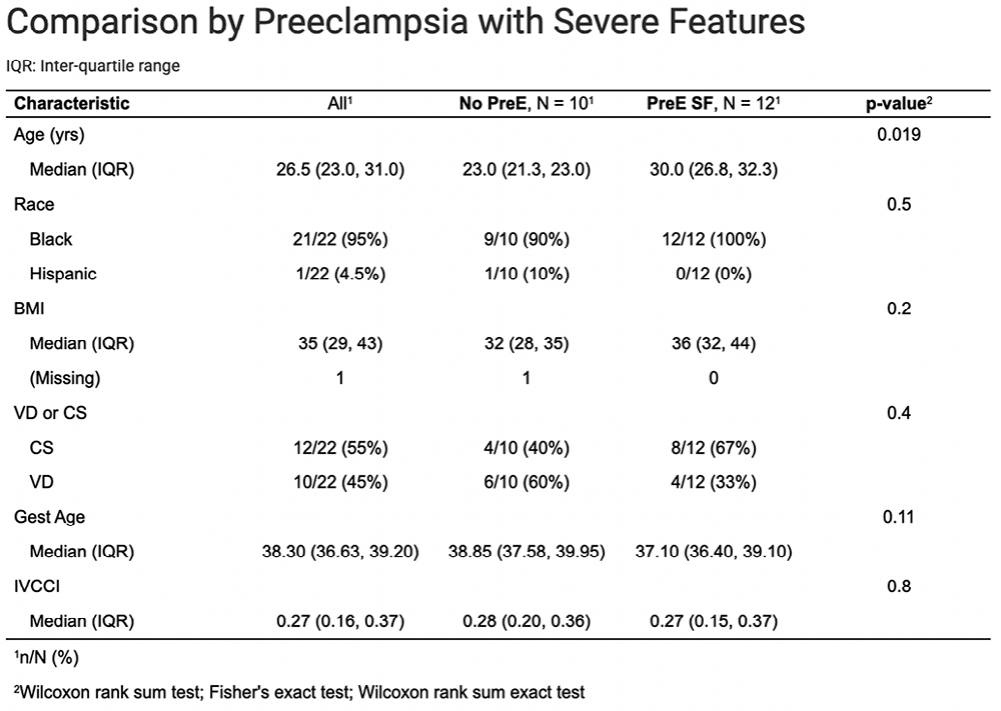

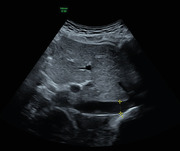

## 1027 | Should Fetuses with Growth Restriction at Term Undergo Trial of Labor?

Emily Schneider^1^; Dajana Alku^1^; Amberly Lao^1^; Delphina Maldonado^2^; Emma Walker^1^; Xiwei Yang^3^; Martin Chavez^4^; Hye Heo^2^

*^1^NYU Grossman Long Island School of Medicine, Mineola, NY; ^2^NYU Langone Hospital, Long Island, NY; ^3^NYU Langone Hospital ‐ Long Island, Mineola, NY; ^4^NYU Langone Hospital, New York, NY*

4:00 PM ‐ 6:00 PM

**Objective**: To determine if trial of labor in term pregnancies with fetal growth restriction (FGR) impacts neonatal morbidity compared to planned Cesarean delivery.

**Study Design**: This is a single‐site retrospective cohort study evaluating neonatal outcomes of singleton pregnancies with term FGR from 10/2019 to 02/2024 who underwent trial of labor (TOL) compared to planned Cesarean delivery (CD). Patients with fetal aneuploidy, major structural anomalies, or requiring emergent delivery prior to TOL were excluded. Patients were identified using a query within the obstetric ultrasound imaging program. FGR was defined as estimated fetal weight (EFW) or abdominal circumference (AC) < 10% for gestational age (GA). Maternal demographics, obstetric, and neonatal outcomes were obtained by chart review. Variables were compared via Chi‐square and Fisher's exact test, with significance of p < 0.05. Univariate logistic regression was used for the binary composite endpoint. The primary outcome was composite neonatal morbidity.

**Results**: There were 217 patients included for analysis; 178 (82.0%) underwent TOL and 39 (18.0%) planned CD. Of the TOL group, 140 (78.7%) had a vaginal delivery (VD) and 38 (21.3%) underwent CD. The TOL group was younger and had lower pre‐pregnancy BMI. Baseline characteristics were otherwise similar (Table 1). There was no difference in composite neonatal morbidity between groups. Subgroup analyses also did not demonstrate a difference in composite neonatal morbidity based on planned mode of delivery (Table 2). Delivery complication rates were similar between groups.

**Conclusion**: In pregnancies with term FGR, there was no difference in composite neonatal morbidity in those undergoing TOL versus planned CD. Subgroup analyses for severe FGR, FGR based on AC < 10%, oligohydramnios, and abnormal fetal Dopplers showed no difference in composite neonatal morbidity. Of those with term FGR undergoing TOL, 78.7% had a VD without significantly increased neonatal morbidity or delivery complications. These findings support a trial of labor for patients with FGR at term.

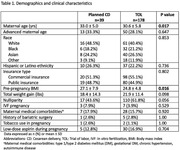

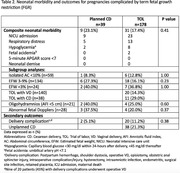

## 1028 | The Association between PSV MCA and Perinatal Outcomes in Pregnancies Complicated by Small‐for‐Gestational‐Age/Fetal‐Growth‐Restricted Fetuses

Misgav Rottenstreich^1^; Bryon DeFrance^2^; Jon F. Barrett^2^; Eran Ashwal^2^

*^1^McMaster University, Hamilton, ON; ^2^McMaster university, Hamilton, ON*

4:00 PM ‐ 6:00 PM

**Objective**: This study aimed to determine the relationship between middle cerebral artery (MCA) peak systolic velocity (PSV) and perinatal morbidity and mortality in SGA and FGR fetuses.

**Study Design**: A retrospective cohort study from a tertiary hospital including all singleton pregnancies without fetal genetic/structural anomalies and EFW and/or AC < 10^th^ centile for gestational age (2017‐2022). Only cases in which the umbilical artery (UA) and MCA were concurrently sampled within 14 days of birth were considered. The study compared cases with abnormal MCA PSV (> 95th centile for gestational age) to pregnancies with normal MCA PSV. The primary outcome was a composite adverse perinatal outcomes defined as either antenatal fetal death, cesarean birth due to fetal distress, 5‐minute Apgar < 7, arterial cord < 7.1, prolonged NICU admission (>7 days), need for intubation, sepsis, necrotizing enterocolitis, and neonatal death. Multi variable logistic regressions were conducted, adjusting for relevant covariates.

**Results**: The study included 983 patients, among whom 98 (10%) had abnormal MCA PSV and 885 (90%) had a normal PSV MCA. Maternal sociodemographic characteristics were similar between the group. Cases with abnormal MCA PSV presented worse Doppler studies. Cases with abnormal MCA PSV delivered earlier than those with normal MCA PSV (33.9±4.5 vs. 36.1±3.3 wks; p< 0.001); nevertheless, the composite adverse perinatal outcomes did not differ between those with abnormal and normal PSV MCA (56.1% vs. 46.8%, p = 0.09). Subgroup analysis limited to cases with ultrasound assessment ≥ 32 weeks (n = 796); or those with normal MCA PI (≥ 5^th^ centile; n = 708); or with normal CPR (≥ 5^th^ centile; n = 621) presented similar composite adverse outcomes irrespective of MCA PSV status (Figure). Multivariate logistic regression controlling for MCA PI and UA PI failed to show a significant association between abnormal MCA PSV to the composite adverse outcome aOR 1.07; 95% CI 0.68‐1.67.

**Conclusion**: In pregnancies complicated with SGA or FGR, abnormal MCA PSV, as a sole parameter, is not associated with adverse fetal/neonatal outcomes.

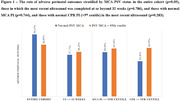

## 1029 | Unraveling Risk‐Factors for Intrapartum Cesarean Section in Pregnancies with Fetal‐Growth‐Restriction Undergoing Labor Induction at Term

Misgav Rottenstreich^1^; Bryon DeFrance^2^; Jon F. Barrett^2^; Eran Ashwal^2^

*^1^McMaster University, Hamilton, ON; ^2^McMaster university, Hamilton, ON*

4:00 PM ‐ 6:00 PM

**Objective**: This study aimed to identify risk factors for intrapartum cesarean section in pregnancies complicated by fetal growth restriction undergoing labor induction at term.

**Study Design**: This case‐control study included singleton pregnancies undergoing labor induction at term (≥37 weeks). All cases had estimated fetal weight (EFW) and/or abdominal circumference (AC) below the 10^th^ centile for gestational age on the last sonographic assessment within 14 days of delivery. Pregnancies with known fetal genetic/structural abnormalities, previous ≥2 cesarean sections, or non‐vertex presentation were excluded from the study. Perinatal and fetoplacental characteristics were compared between cases with successful vaginal delivery and those requiring intrapartum cesarean section (CS). Multivariable logistic regressions were conducted to identify independent variables associated with the need for intrapartum CS.

**Results**: A total of 405 eligible patients participated in the study, with 323 (79.8%) experiencing vaginal delivery, while 82 (20.2%) required intrapartum CS. Those undergoing CS exhibited higher rates of nulliparity and obesity. Gestational age at the most recent ultrasound assessment, mean EFW, and pulsatility index (PI) values of the umbilical artery and middle cerebral artery (MCA) were comparable between the groups. However, cases that underwent CS had a higher rate of fetal AC below the 3rd centile and abnormal cerebroplacental ratio PI. The gestational age at delivery did not differ significantly between the two groups. In the multivariate analysis (Table 2), maternal obesity, nulliparity, previous single CS, AC< 3^rd^ centile, and abnormal cerebroplacental PI were identified as significant and independent predictors of intrapartu m CS. The predictive model demonstrated an area under the curve (AUC) of 0.714 (95% CI: 0.651‐0.778); p< 0.001, indicating good discriminative ability.

**Conclusion**: Understanding maternal and fetoplacental characteristics can offer valuable insights into the success of vaginal delivery in pregnancies complicated by fetal growth impairment undergoing labor induction at term.

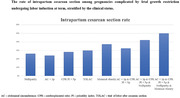

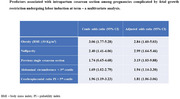

## 1030 | Prevalence of Cardiovascular‐Kidney‐Metabolic Syndrome in Reproductive‐aged Women in the United States: NHANES 2011‐2020

Eric K. BRONI^1^; Ebenezer Aryee^2^; Amber Lachaud^1^; Jennifer Lewey^1^; Lisa D. Levine^1^

*^1^University of Pennsylvania Perelman School of Medicine, Philadelphia, PA; ^2^Johns Hopkins School of Medicine, Baltimore, MD*

4:00 PM ‐ 6:00 PM

**Objective**: The AHA recently defined and staged cardiovascular kidney‐metabolic (CKM) syndrome to highlight the multisystem consequences of poor CKM health, harmonize guidelines and provide opportunities for actionable preventive interventions before overt CVD. In the US, the components that comprise CKM (e.g., obesity, diabetes, hypertension) are potent risk factors for poor maternal and neonatal outcomes with a disproportionate burden among racial/ethnic minorities. However, population‐level data on CKM syndrome in reproductive‐aged women is scarce and is a critical next step in evaluating the association between CKM stages and overall pregnancy outcomes.

**Study Design**: We analyzed 2,347 non‐pregnant, reproductive‐aged women (≥ 20‐49 years) from the National Health and Nutrition Examination Survey (NHANES) who had data on all CKM risk factors. We incorporated sampling weights into our analyses to account for the complex NHANES survey design. We reported prevalence estimates and standard errors of CKM syndrome stages (defined in Table) in the overall population, by race/ethnicity and across the study period (2011‐2020).

**Results**: The mean age was 34.6 ± 7.7 yrs., with 57% non‐Hispanic (NH) White, 19% Hispanic, 13% NH Black, 6% NH Asian, and 4% self‐reported other races. Within the study period, 74.7% of participants had CKM syndrome [Stage 1: 37.3%, Stage 2: 35.7%, Stage 3: 0.3% and Stage 4: 1.97%], with notable variations over time. **(Table A & Figure A)**. As noted in **Table B/Figure B**, NH Black women had the lowest prevalence of stage 0 CKM (13.2%) and the highest prevalence of stages ≥ 2 CKM (45.6%), followed by other races (40.8%) and Hispanic women (39.4%). Asian women had the lowest prevalence of stages ≥ 2 CKM (27.8%).

**Conclusion**: More than three‐quarters of reproductive‐aged women in the U.S. have at least stage 1 CKM with notable racial/ethnic disparities. Focusing health efforts on the prevention of these modifiable risk factors can undoubtedly improve health outcomes within this population. Studies evaluating the implications of these findings for overall pregnancy outcomes are urgently warranted.

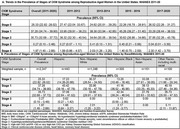

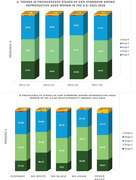

## 1031 | Incidence And Risk Factors For Postpartum Hypertension In LMICs: A Large Prospective Multi‐Country Cohort Study

M. Bridget Spelke^1^; Gillian Henderson^2^; Erin M. Oakley^3^; Victor Akelo^4^; Kwaku Poku Asante^5^; Irene Azindow^5^; Sasha Baumann^6^; Santosh Benjamin^7^; Anne G. Cherian^8^; Bethany L. Freeman^9^; Zahra Hoodbhoy^10^; Arun Singh Jadaun^11^; Margaret P. Kasaro^12^; Amna Khan^10^; Neeraj Sharma^11^; Jasmine Sugirtha^7^; Rupa Talukdar^11^; Charlotte Tawiah^5^; Joyce Akinyi Were^4^; Zacchaeus Abaja Were^4^; Blair Wylie^13^; Nida Yazdani^10^; Emily Smith^14^

*^1^University of North Carolina at Chapel Hill; UNC ‐Global Projects Zambia, Lusaka, Lusaka; ^2^University of North Carolina Global Projects–Zambia, Chapel Hill, NC; ^3^The George Washington, Washington, DC; ^4^Kenya Medical Research Institute (KEMRI); ^5^Kintampo Health Research Centre, Kintampo Health Research Centre, Brong‐Ahafo; ^6^The George Washington University, Washington, DC; ^7^Christian Medical College, Vellore, Tamil Nadu; ^8^Christian Medical College, Vellore, Christian Medical College, Tamil Nadu; ^9^University of North Carolina at Chapel Hill, Chapel Hill, NC; ^10^Aga Khan University, Karachi, Sindh; ^11^Society for Applied Studies, Delhi, Delhi; ^12^UNC Lusaka, UNC Zambia, Lusaka; ^13^Columbia University Medical Center, New York, NY; ^14^The George Washington University, The George Washington University, DC*

4:00 PM ‐ 6:00 PM

Objective: To identify the incidence and risk factors associated with elevated blood pressure at 6 weeks postpartum among women in Ghana, Kenya, India, Pakistan, and Zambia. Study Design: We report findings from the Pregnancy Risk, Infant Surveillance, and Measurement Alliance (PRISMA) prospective multi‐country pregnancy cohort in Kenya, Ghana, Zambia, Pakistan, and India. Hypertension (HTN) is defined as blood pressure (BP) greater than 140/90. We excluded participants with known chronic hypertension or elevated BP prior to 20 gestational weeks. We used descriptive statistics to report the incidence of postpartum hypertension overall and by region. We performed multivariable Poisson regression with robust variance to describe the relationship between risk factors and postpartum hypertension with relative risks and 95% confidence intervals. Results: A total of 3858/4358 (89%) participants had at least 1 BP measured at the 6‐week postpartum visit and were included in the analysis. The incidence of high BP postpartum differed by region, with 2.7% of participants in South Asia and 7.3% in Africa meeting criteria. Overall, 50/251 (19.9%) participants with hypertensive diseases of pregnancy had persistent elevated BP at 6 weeks postpartum, compared to 4% new onset high BP at 6 weeks postpartum among those who did not have any hypertensive disorders of pregnancy. Factors associated with high BP postpartum included maternal age (aRR 1.10, 95% CI 1.08, 1.13), early pregnancy BMI ≥ 30 (aRR 2.24, 95% CI 1.58, 3.18), and a diagnosis of gestational hypertension or preeclampsia during the current pregnancy (aRR 4.20, 95% CI 2.9, 5.9; aRR 4.49 95% CI 2.85, 7.07, respectively). Participants with HIV infection were less likely to have elevated blood pressure (aRR 0.46 95% CI 0.25, 0.83). Conclusion: This study demonstrates a high incidence of elevated blood pressure postpartum with notable differences by region. These findings underscore the need for robust follow‐up at and beyond 6 weeks after delivery and linkages to primary care for women with persistent hypertension.

## 1032 | Shoulder Dystocia Resolution Utilizing < vs. > 3 Maneuvers and Associated Adverse Outcomes

Fabrizio Zullo^1^; Teresa C. Logue^2^; Daniele Di Mascio^3^; Giuseppe Rizzo^3^; Antonella Giancotti^3^; Hector M. Mendez‐Figueroa^4^; Matthew K. Hoffman^2^; Anthony C. Sciscione^2^; Suneet P. Chauhan^5^

*^1^University of Rome La Sapienza, Rome, Lazio; ^2^Christiana Care Health System, Newark, DE; ^3^Sapienza Università di Roma (ROMA), Rome, Lazio; ^4^McGovern Medical School at UTHealth, Houston, TX; ^5^Delaware Center of Maternal‐Fetal Medicine at Christiana Care, Delaware, DE*

4:00 PM ‐ 6:00 PM

**Objective**: Our objective was to examine the association between the use of < 3 versus > 3 maneuvers to resolve SD and adverse outcomes.

**Study Design**: This was a secondary analysis of the Assessment of Perinatal EXcellence (APEX) study, a observational cohort of over 115,000 deliveries within 25 U.S. hospitals. We included women with singleton, vertex, non‐anomalous gestations at > 34 weeks who had SD, relieved with at least 1 maneuver. We excluded SD if no maneuvers were documented. We stratified the groups according to the number of maneuvers used to resolve the shoulder dystocia. The primary outcome was a neonatal composite outcome encompassing Apgar < 5 at 5’, fetal fractures, intracranial hemorrhage, brachial plexus injury, facial nerve palsy, hypotension and neonatal death. Statistical analysis included Chi‐square, Kruskal‐Wallis, logistic and Poisson regressions with robust error variance for adjusted Incidence Rate Ratios, adjusting for BMI and maternal age.

**Results**: The overall rate of SD in APEX was 1.9% (2,138/118,422). Of 2,138 SD, 96% met the inclusion criteria for analysis. Three or more maneuvers were utilized in 19% (391/2,062) of SD. The composite outcome was higher with > 3 maneuver (14.6%) vs < than 3 (5.7%; Fig 1). After adjustement the aIRR was 2.28 (95%CI 1.62, 3.20). Additionally, Apgar < 5 at 5` (aIRR 4.10 95% CI 1.18‐14.25), fetal fractures (aIRR 2.58 95% CI 1,66‐4.00), intracranial hemorrhage (aIRR 4.28 95%CI 1.06‐17.19), brachial plexus injury (aIRR 2.58 95%CI 1.45‐4.60) were significantly more likely when > 3 vs < 3 maneuvers were used. No statistically significant differences were noted for rates of facial nerve palsy, hypotension requiring treatment and neonatal death and postpartum hemorrhage (Table 1).

**Conclusion**: Shoulder dystocia relieved by 3 or more maneuvers, compared to fewer, was associated with a 2.5‐fold increased risk of composite neonatal adverse outcomes, albeit not postpartum hemorrhage.

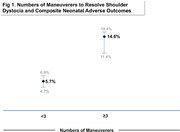

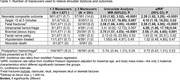

## 1033 | Variation in Fetal Heart Rate Tracing and Adverse Outcomes among Pregnancies Complicated by Hypertensive Disorder

Fabrizio Zullo^1^; Aaron W. Roberts^2^; Kristen A. Cagino^3^; Rachel L. Wiley^4^; Claudia J. Ibarra^5^; Shareen Patel^5^; Christina Cortes^5^; Natalie L. Neff^6^; Kimen S. Balhotra^5^; Tala Ghorayeb^5^; Khalil M. Chahine^5^; Holly Flores^7^; Hector M. Mendez‐Figueroa^5^; Suneet P. Chauhan^8^

*^1^University of Rome La Sapienza, Rome, Lazio; ^2^McGovern Medical School at UTHealth Houston, Houston, TX; ^3^UT Houston, Houston, TX; ^4^University of California, San Diego, San Diego, CA; ^5^McGovern Medical School at UTHealth, Houston, TX; ^6^McGovern Medical School at UT Health, Houston, TX; ^7^University of Texas Health Science Center, Houston, TX; ^8^Delaware Center of Maternal‐Fetal Medicine at Christiana Care, Delaware, DE*

4:00 PM ‐ 6:00 PM

**Objective**: To compare the fetal heart rate tracing (FHRT) and adverse outcomes among hypertensive and non‐hypertensive women who delivered at term (> 37 wks).

**Study Design**: The inclusion criteria of the retrospective study were consecutive individuals with non‐anomalous singletons who delivered at > 37 wks over 15 months. The groups were stratified by the presence or absence of hypertensive disorder of pregnancy (HDP), defined as gestational and chronic hypertension. Using the ACOG guidelines, clinicians—blinded to maternal characteristics, BP status, and outcomes—interpreted the FHRT for the last 60 mins of labor. Composite neonatal and maternal adverse outcomes (CNAO, CMAO) were compared between the groups. Chi‐square test was used to compare groups. Odds Ratios (OR) and adjusted OR were calculated using a multivariate binomial logistic regression model with permuted omnibus tests to identify confounding factors.

**Results**: Of the 5,160 deliveries during the study period, 3,166 (61%) met the inclusion criteria and among them 1,062 (33%) had HDP. Pregnancies complicated by HDP were significantly more likely to have minimal variability (p< 0.01). Decelerations occurred at similar rate. The rate of category I, II and III tracings were similar (Table 1). Cesarean delivery was  comparable among the two groups after adjustment. CNAO of newborns of individuals with HDP (2.5%) was significantly higher than newborn of normotensive individuals (1.0%; aOR 2.32 95%CI 1.21–4.49). CMAO was significantly higher in the HDP (9.9%) than normotensive group (6.8%; aOR 1.56 95% CI 1.17–2.07); Table 2). Apgar score < 7 at 5 min and neonatal seizure were the only components of the CNAO that differed significantly.

**Conclusion**: Minimal variability was the only fetal heart rate characteristic which differed significantly among hypertensive vs. normotensive individuals. Neonatal and maternal adverse outcomes were significantly higher in the hypertensive group. Intervention trials mitigating the increased likelihood of adverse outcomes among term individuals with hypertensive disorder are warranted.

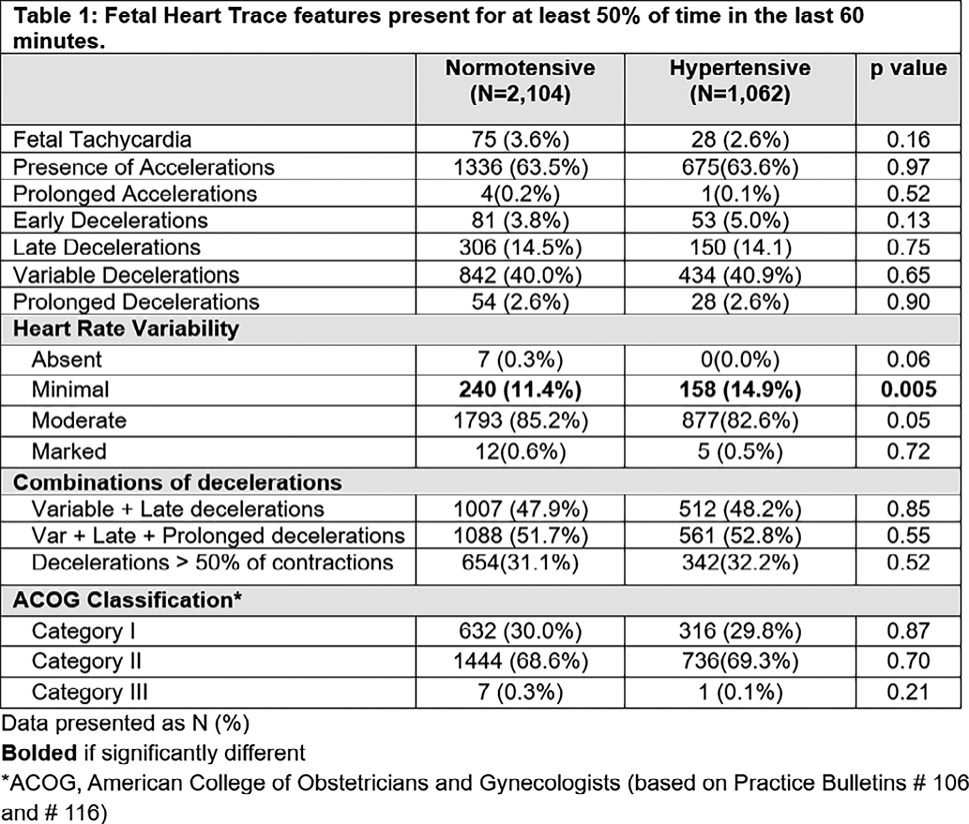

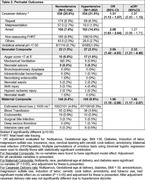

## 1034 | Impact of Timing of Hysteroscopy for Retained Products of Conception on Subsequent Pregnancy Outcomes

Gal Bachar^1^; Efrat Leibowitz^2^; Ron Beloosesky^2^; Naphtali Justman^1^; Dana Vitner^1^; Yaniv Zipori^1^; Zeev Weiner^2^; Nizar Khatib^2^

*^1^Rambam Medical Center, Haifa, Hefa; ^2^Rambam Medical Health Center, Haifa, Hefa*

4:00 PM ‐ 6:00 PM

**Objective**: Retained products of conception (RPOC) occur in ∼3% of deliveries and can have significant adverse effects on women's health. Hysteroscopy removes RPOC but has its own possible side effects. On the other hand, delayed treatment may damage the endometrium. Prolonged RPOC can lead to infection and inflammation, potentially affects future pregnancy outcomes. We studied outcomes for women with suspected RPOC, comparing hysteroscopy  performed within 8 weeks versus more than 8 weeks postpartum.

**Study Design**: Our 2010‐2023 retrospective dataset included information on women who had suspected RPOC and who underwent hysteroscopy at a tertiary medical health center. Women with a documented subsequent live birth following hysteroscopy were categorized based on timing: Group 1 (hysteroscopy within 8 weeks) and Group 2 (hysteroscopy beyond 8 weeks). We compared maternal characteristics, hysteroscopic findings, subsequent delivery details and neonatal outcomes between the two groups. The primary outcome was gestational age at delivery for the subsequent pregnancy. Secondary outcomes included various maternal and neonatal complications.

**Results**: Both groups had similar baseline characteristics. There were no significant differences in most aspects of the first delivery course, including mode of delivery. Indications for hysteroscopy were similar between groups, with retained placental tissue being the most common finding.

Regarding subsequent pregnancies following hysteroscopy, time to conception, gestational age at delivery, pregnancy complications including hypertensive disease, and most delivery outcomes were all similar between the two groups. Neonatal outcomes were also comparable.

**Conclusion**: The timing of hysteroscopy for RPOC following delivery ‐ whether within 8 weeks or more than 8 weeks postpartum ‐ appears to have minimal impact on subsequent pregnancy outcomes. Therefore, a wait‐and‐see approach might be recommended at least for two months after delivery. However, further investigation with a larger sample size is needed to confirm these findings.

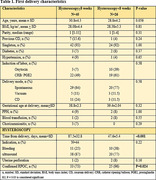

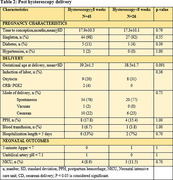

## 1035 | Vaginal Compared with Oral Misoprostol Induction for Individuals with Morbid Obesity

Gayathri D. Vadlamudi^1^; Donald D. McIntire^2^; Emily H. Adhikari^2^

*^1^UT Southwestern Medical Center, Dallas, TX; ^2^University of Texas Southwestern Medical Center, Dallas, TX*

4:00 PM ‐ 6:00 PM

**Objective**: To characterize labor progress in term individuals with morbid obesity undergoing induction using vaginal compared with oral misoprostol

**Study Design**: We conducted a secondary analysis of a cluster randomized controlled trial comparing oral or vaginal misoprostol regimens for term labor induction among individuals with intact membranes and cervical dilation ≤ 2cm. Pregnant persons with BMI ≥ 40kg/m2 at delivery were included. Weekly randomization was assigned to either oral (100 µg every 4 hours for up to two doses) or vaginal (25 µg every 3 hours for up to five doses) misoprostol, followed by a standardized oxytocin protocol. We recorded cervical exams at misoprostol administrations, oxytocin initiation, and prior to delivery. We compared labor progress and duration, need for oxytocin, and delivery and neonatal outcomes using generalized estimating equations to account for clustering. Outcomes were stratified by parity. Labor curves were constructed using a mixed effects repeated measures model.

**Results**: Of 491 patients, 264(53.8%) received vaginal and 227(46.2%) oral misoprostol. There were no significant demographic differences. Route of administration was not associated with a difference in time (hours) to delivery (22.9±0.74 vaginal vs 22.3±0.78 oral). Among 165(66.2%) vaginal and 137(62.8%) oral misoprostol recipients who reached the second stage of labor, length of first stage did not differ (20.6±0.81h vs 20.5±0.89h). Need for oxytocin was lower with vaginal misoprostol (188(71.2%) vs 194(85.5%). There were no significant differences in vaginal delivery or other maternal and neonatal outcomes including umbilical cord pH < 7.0 and neonatal intubation. Associations between route of misoprostol and outcomes were similar after stratification by parity. Rate of cervical dilation did not differ by route of administration.

**Conclusion**: In patients with morbid obesity undergoing term induction, route of misoprostol does not impact time to delivery or vaginal delivery rates.

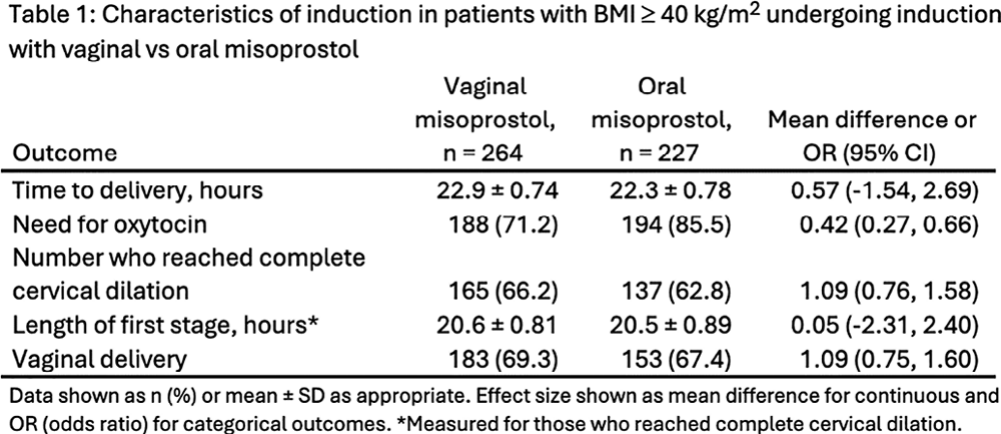

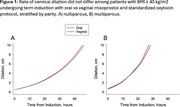

## 1036 | Characterizing Vaginal Misoprostol Induction in Individuals with Morbid Obesity

Gayathri D. Vadlamudi^1^; Donald D. McIntire^2^; Emily H. Adhikari^2^

*^1^UT Southwestern Medical Center, Dallas, TX; ^2^University of Texas Southwestern Medical Center, Dallas, TX*

4:00 PM ‐ 6:00 PM

**Objective**: To compare labor progression in individuals with body mass index < 40 vs ≥ 40 kg/m2 undergoing induction at term

**Study Design**: This was a secondary analysis of the vaginal misoprostol arm of a cluster randomized controlled trial comparing vaginal to oral misoprostol induction. This analysis included patients ≥ 37 weeks, with cervical dilation ≤ 2 cm who underwent induction using a standardized protocol. Vaginal misoprostol was given in 25 µg doses every three hours for up to five doses, followed by a standardized high‐dose oxytocin protocol. We compared characteristics of labor progression among individuals with BMI < 40 and ≥ 40kg/m2 including labor duration, number of misoprostol doses, and obstetric and neonatal outcomes. Data was analyzed using generalized estimating equations to account for clustering. Effect sizes were presented as odds ratios and mean differences in duration with 95% confidence intervals.

**Results**: From May 2021 to September 2022, 1322 patients received vaginal misoprostol, among whom 1058(80.0%) had BMI < 40 kg/m2 and 264(20.0%) had BMI ≥ 40kg/m2. Baseline differences between the two groups are shown in Table 1. Cervical dilation at the start of induction was lower in patients with BMI ≥ 40, and these individuals required more doses of misoprostol during induction (Table 2). Inductions in this group had longer durations of oxytocin use (13.8 ± 9.2 vs 11.1 ± 8.0 h), length of first stage of labor (20.6 ± 11.1 vs 18.0 ± 9.4 h), and time to delivery (22.9 ± 12.1 vs 19.0 ± 10.3), with lower rates of vaginal delivery (69.3 vs 80.2%). Patients with morbid obesity were more likely to have excess blood loss (22.3 vs 15.5%) and develop wound infection (1.5 vs 0.4%).

**Conclusion**: Following term labor induction with vaginal misoprostol, morbid obesity is associated with longer labor duration, lower vaginal delivery frequency, and increased obstetric morbidity. Whether alternative labor induction protocols in morbidly obese patients improve these outcomes requires additional study.

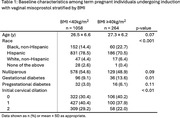

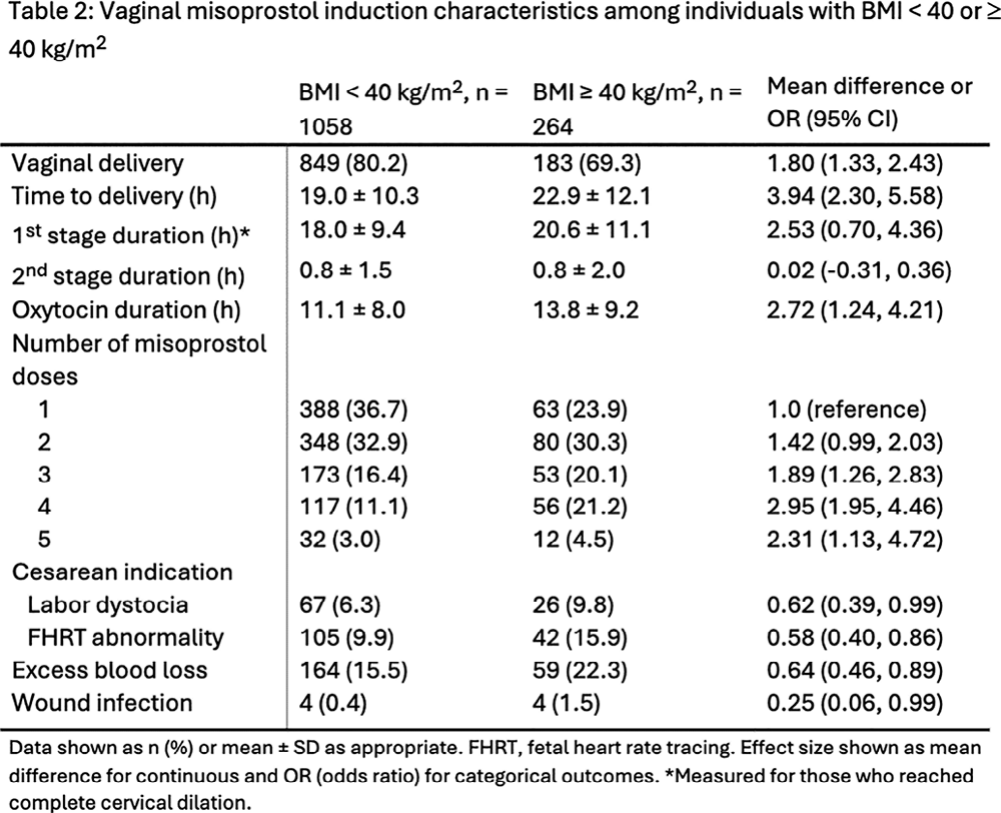

## 1037 | Recurrent Pregnancy Loss as an Independent Risk Factor for Celiac Disease of The Offspring

Gil Gutvirtz^1^; Gali Pariente^2^; Tamar Wainstock^2^; Eyal Sheiner^2^

*^1^Soroka University Medical Center, Metar, HaDarom; ^2^Department of Obstetrics and Gynecology, Soroka University Medical Center, Ben‐Gurion University of the Negev, Beer‐Sheva, Israel., Beer Sheva, HaDarom*

4:00 PM ‐ 6:00 PM

**Objective**: Celiac disease is a common immune‐mediated inflammatory disease that mainly develops in genetically predisposed individuals. However, it is still unclear what predisposes some to develop the disease but not others. Women with a history of recurrent pregnancy losses (RPL) may harbor a genetic disposition for inflammatory responses causing pregnancy losses and this genetic trait may pass to their offspring. We sought to investigate whether offspring of women with RPL are at an increased risk to develop celiac disease.

**Study Design**: A population‐based cohort study was conducted to evaluate the risk to develop celiac disease in offspring (up to the age of 18 years) born to mothers with and without a history of RPL. Only singleton deliveries were included. Data for the diagnosis of celiac disease of the offspring was extracted from community‐based clinics and/or hospitalization records. Kaplan‐Meier survival curve was used to compare the incidence of celiac disease between the study groups. A cox regression model was used to control for confounders.

**Results**: During the 30 years (1991‐2021) study period, 356,356 singleton deliveries occurred; 14,235 deliveries (4%) were in women with a history of RPL. Children in this group had higher rates of celiac disease as compared to children born to mothers without a history of RPL (**Figure**). The Kaplan‐Meier survival curve illustrated higher incidence of celiac disease in offspring with maternal history of RPL (**Figure**). Using a Cox regression model, adjusting for maternal celiac disease, maternal age and gestational age at birth, maternal history of RPL was independently associated with celiac disease of the offspring (adjusted HR = 1.22, 95% CI 1.1‐1.5; P = 0.04).

**Conclusion**: Maternal history of RPL is an independent risk factor for celiac disease in the offspring.

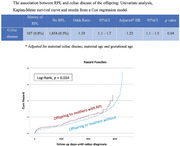

## 1038 | Maternal Vaginal Colonization with Group‐B Streptococcus During Labor and Long‐Term Cardiovascular Morbidity of the Offspring

Gil Gutvirtz^1^; Tamar Wainstock^2^; Eyal Sheiner^2^

*^1^Soroka University Medical Center, Metar, HaDarom; ^2^Department of Obstetrics and Gynecology, Soroka University Medical Center, Ben‐Gurion University of the Negev, Beer‐Sheva, Israel., Beer Sheva, HaDarom*

4:00 PM ‐ 6:00 PM

**Objective**: Group B Streptococcus (GBS) is a Gram‐positive bacteria that commonly colonizes the gastrointestinal and genital tracts of pregnant women. Vertical transmission to the neonate may occur during labor. While early onset GBS infection usually manifests with sepsis, pneumonia or meningitis, cardiac complications such as endocarditis, myocarditis and pericarditis have also been reported. This study was aimed to investigate whether an association exists between maternal vaginal GBS and offspring long‐term cardiovascular morbidity

**Study Design**: A retrospective population‐based cohort analysis of singleton vaginal deliveries between the years 2002‐2021 at a tertiary medical center was performed. Long‐term cardiovascular morbidity of children who were exposed to maternal positive GBS was compared with children to mothers with negative or unknown GBS status. Data for long‐term cardiovascular morbidity of the offspring was extracted from community‐based clinics and hospitalization records. A Kaplan‐Meier survival curve was constructed to compare long‐term cumulative cardiovascular morbidity, and a Cox regression model was used to adjust for possible confounders

**Results**: The analysis included 146,103 singleton vaginal deliveries that occurred during the study period. Of them, 2,225 (1.5%) mothers were identified with positive GBS status. The cumulative incidence of long‐term cardiovascular morbidity was comparable between maternal GBS exposed offspring and those with negative or unknown maternal GBS status (**Figure**). The Cox regression model, adjusting for various confounders including gestational and maternal age, hypertensive disorders, diabetes mellitus and ethnicity, found that maternal vaginal GBS colonization was not associated with long‐term cardiovascular morbidity of the offspring (adjusted HR = 0.88; 95% CI 0.55‐1.42, P = 0.608, **Table**)

**Conclusion**: Maternal vaginal colonization with GBS during vaginal delivery is not associated with long‐term cardiovascular morbidity of the offspring

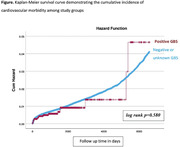

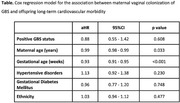

## 1039 | Pulse Pressure does not Predict Response to Treatment of Severe Hypertension in Preeclampsia

Gillian Piltch^1^; Rachel P. Gerber^2^; Chisom Chigozie‐Nwosu^3^; Burton Rochelson^2^; Matthew J. Blitz^2^; Evelina Grayver^2^; Alejandro D. Alvarez^2^; David Krantz^2^; Caroline Pessel^2^

*^1^Northwell, Astoria, NY; ^2^Northwell, New Hyde Park, NY; ^3^Zucker School of Medicine at Hofstra/Northwell, Hempstead, NY*

4:00 PM ‐ 6:00 PM

**Objective**: To evaluate pulse pressure as a predictor of response to treatment of severe hypertension in preeclampsia.

**Study Design**: Multicenter retrospective cohort study of pregnant or postpartum people who received treatment with immediate release nifedipine or intravenous labetalol or hydralazine for severe hypertension due to preeclampsia with severe features from 2017‐2022. Primary outcomes were time to resolution of severe hypertension and resolution of severe hypertension within 30 minutes. Secondary outcomes were number of fast‐acting antihypertensive doses and of distinct ACOG‐defined medication algorithms needed for resolution of severe hypertension. Log‐rank tests, likelihood ratio tests, and t‐tests were performed with p< 0.05 considered statistically significant.

**Results**: 1191 people met inclusion criteria. Range in pulse pressure of the severe blood pressure immediately prior to fast‐acting antihypertensive administration was 24‐132 mmHg and in time to resolution of severe hypertension was 10‐295 minutes. There was no relationship between pulse pressure of the severe blood pressure immediately prior to treatment (Figure 1) or change in pulse pressure between the two severe blood pressures prompting treatment (Figure 2) and time to resolution of severe hypertension. Pulse pressure was not predictive of resolution of severe hypertension within 30 minutes (p = 0.10). Subgroup analysis stratified by first fast‐acting antihypertensive administered demonstrated no relationship between pulse pressure and resolution within 30 minutes among those who received immediate release nifedipine (p = 0.23) or intravenous labetalol (p = 0.48) or hydralazine (p = 0.45). There was no association between pulse pressure and number of doses of fast‐acting antihypertensives (p = 0.74) needed for resolution. There was a wider range in pulse pressures that included narrower pulse pressures among people who required one medication compared to those who needed more than one (p< 0.01) for resolution.

**Conclusion**: Pulse pressure was not predictive of time to resolution of severe hypertension in preeclampsia with severe features.

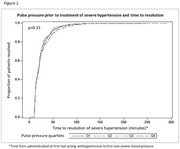

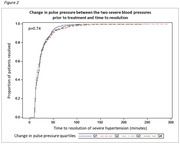

## 1040 | Safety and Efficacy of Maternal COVID‐19 Vaccination During Pregnancy: Umbrella Review & Meta‐Analyses

Giulia Bonanni^1^; Nikan Zargarzadeh^2^; Mohammad Haddadi^3^; Ali Javinani^2^; James Versalovic^4^; Kjersti M. Aagaard^5^; Stefania Papatheodorou^6^; Alireza A. Shamshirsaz^7^

*^1^Postdoctoral Research Fellow Fetal Care and Surgery Center Division of Fetal Medicine and Surgery Boston Children's Hospital, Harvard Medical School, Boston, MA; ^2^Boston Children's Hospital, Harvard Medical School, Boston, MA; ^3^Vali‐E‐Asr Reproductive Health Research Center, Family Health Research Institute, Tehran University of Medical Sciences, Tehran, Iran, Tehran, Tehran; ^4^Department of Pathology & Immunology, Baylor College of Medicine, Houston, TX, USA Department of Pathology, Texas Children's Hospital, Houston, TX; ^5^Boston Children's Hospital, Division of Fetal Medicine and Surgery, Boston, MA; HCA Healthcare and HCA Healthcare Research Institute, Nashville, TN; HCA Texas Maternal Fetal Medicine, Houston, TX; Baylor College of Medicine and Texas Children's Hospital, Houston, TX, Boston, MA; ^6^Department of Epidemiology, Harvard T.H. Chan School of Public Health, Harvard University, Boston, MA, USA Department of Biostatistics and Epidemiology, Rutgers School of Public Health, New Brunswick, NJ, USA, New Brunswick, NJ; ^7^Fetal Surgeon Chief, Division of Fetal Medicine and Surgery Director of the Maternal Fetal Care Center Director of the Perinatal Surgery Fellowship Professor of Surgery, Boston Children's Hospital Harvard Medical School, Boston, MA*

4:00 PM ‐ 6:00 PM

**Objective**: The safety & efficacy of vaccination against COVID‐19 during pregnancy is crucial for not only current maternal and fetal health, but public health policy and management with future pandemics. To date, many meta‐analyses on the safety and efficacy of COVID‐19 vaccine were published with conflicting results; we aimed to correct this crucial knowledge gap with the current study.

**Study Design**: We conducted a systematic search of PubMed, Scopus, Web of Science, and Embase from 01/01/2021 to 09/13/2023. We included 23 meta‐analyses inclusive of over 200 studies and 1,300,000 gravidae with or without documented evidence of COVID‐19 vaccination. Meta‐analyses were performed using R to aggregate effect sizes, utilizing random‐effects models. Heterogeneity was evaluated using the I^2^ statistic, and publication bias was assessed with Egger's test. PROSPERO registration: CRD42024519174.

**Results**: COVID vaccination during pregnancy reduced maternal COVID‐19 infection rates (Risk Ratio [RR] 0.41, 95% CI 0.30‐0.57), stillbirth (RR 0.75, 95% CI 0.58‐0.98), PTB ≤37 wks (RR 0.92, 95% CI 0.86‐0.99), congenital malformations (RR 0.83, 95% CI 0.70‐0.98), SGA neonates (RR 0.94, 95% CI 0.91‐0.98), and NICU admission (RR 0.91, 95% CI 0.85‐0.97). No significant risk differences were observed among other outcomes, with the exception of a marginal increased risk of Cesarean delivery (RR 1.07, 95% CI 1.01‐1.12; Table 1, Figure 1).

**Conclusion**: With the conduct of an umbrella review of comprehensive meta‐analyses and including over 1.3M patients, we demonstrate significant benefit towards vaccination at no increased risk. The quantifiable benefits may aid shared decision‐making and public health communications in this and future pandemics.

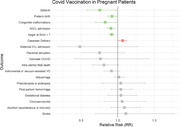

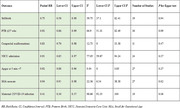

## 1041 | Prenatal Aortic Coarctation Sign Screening Using Automatic AI‐Assisted Vessel Diameter Measurement

Guillaume Corda^1^; Remi Besson^1^; Nikola Matevski^1^; Guy Vaksmann^2^; Julien Stirnemann^3^; Yves Ville^4^

*^1^Sonio, Paris, Ile‐de‐France; ^2^Cabinet Vendôme, Lille, Nord‐Pas‐de‐Calais; ^3^Hôpital Necker Enfants Malades, Paris, Ile‐de‐France; ^4^University and Necker‐Enfants Malades Hospital, Paris, Ile‐de‐France*

4:00 PM ‐ 6:00 PM

**Objective**: Aortic coarctation (AoC) is a congenital heart defect characterized by hypoplasia of the aortic arch visible on the 3‐vessel view (3VV). This study aims to (i) develop a pipeline using artificial intelligence (AI) for automatic vessel diameter measurement, (ii) assess whether vessel diameter is a reliable predictor of AoC prenatally.

**Study Design**: An AI software (under development, not yet FDA approved) trained on thousands of annotated images from US and European sites is used for the automatic detection and segmentation of the pulmonary artery (PA) and aorta (Ao) on 2D 3VV images. Post‐processing algorithms are applied to achieve automatic and standardized diameter measurements. The model's performance is assessed on a test set of images on which the models were not trained, consisting of 2548 non‐pathological images and 32 images from studies where AoC was diagnosed prenatally by a multidisciplinary team (Hôpital Necker, Paris).

**Results**: The models demonstrated high accuracy in vessel diameter measurement. The sign used for AoC screening is the diameter ratio of the PA to that of the Ao. With a ratio greater than 1.6, an AoC should be suspected. This ratio is calculated over the entire test set for the two populations, and a shift is observed (Figure 1). For healthy data, the mean ratio is 1.2±0.3 mm, while for the pathological data, the mean ratio is 1.8±0.3 mm. Our method achieves a sensitivity of 71.9% and a specificity of 87.8% for AoC sign screening.

**Conclusion**: This study validates AI's potential for automatic AoC sign screening. It offers a fast and reliable alternative to manual measurement methods. However, the AP/Ao ratio alone has limitations for screening CoA prenatally. Other indicators should be investigated. This study used cases detected prenatally, introducing a bias. Future work will look at cases that were diagnosed postnatally.

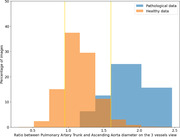

## 1042 | Direct Dispensation of Prenatal Supplements with Iron Among Pregnant People: a Cost‐Effectiveness Analysis

Helen Samuel^1^; Megha Arora^2^; Ashley E. Benson^2^; Aaron B. Caughey^2^

*^1^Oregon Health and Science University, Springfield, VA; ^2^Oregon Health & Science University, Portland, OR*

4:00 PM ‐ 6:00 PM

**Objective**: Postpartum transfusion is a leading cause of severe maternal morbidity in the United States, with pregnant individuals with iron deficiency and/or iron deficiency anemia (IDA) at increased risk. Despite early detection and treatment recommendations to reduce prenatal iron deficiency and IDA, effective strategies to overcome barriers to medication and supplement access are less clear, and iron supplement adherence remains suboptimal during pregnancy. This study compares the cost effectiveness of directly providing versus recommending prenatal iron.

**Study Design**: A decision‐analytic model was constructed in TreeAge to compare directly dispensing prenatal iron supplements versus the standard protocol of recommending prenatal iron supplements in a theoretical cohort of 1,514,784 pregnant individuals enrolled in Medicare annually. Probabilities, utilities, and costs were derived from the literature. Outcomes included costs, quality‐adjusted life years (QALY), preterm deliveries, neurodevelopmental disabilities, maternal postpartum anemia, and postpartum transfusion for acute blood loss. We defined our cost‐effective threshold as 100,000 USD per QALY.

**Results**: Directly dispensing prenatal iron supplements resulted in 62,600 fewer preterm deliveries, 52  fewer cases of neurodevelopmental disability, 75,683 fewer cases of maternal postpartum anemia, and 54,010 fewer postpartum blood transfusions (Table 1) within our theoretical cohort. This intervention resulted in an estimated 187,475 additional QALYs and cost savings of $62,187,202,377 annually. Given the increase in QALYs and cost savings, directly dispensing prenatal iron was a dominant strategy.

**Conclusion**: In this study, directly dispensing prenatal iron supplements to Medicaid‐enrolled pregnant individuals was a cost‐effective strategy associated with reduced rates of adverse perinatal outcomes. These findings support the implementation of iron supplement provision at point of care prenatally to improve adherence, maternal and child health outcomes, and reduce healthcare expenditures.

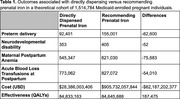

## 1043 | Increased Risk of Major Fetal Anomalies in Cases of Severe Placenta Accreta Spectrum

Helena C. Bartels^1^; Jennifer M Walsh^2^; Donal O'Brien^2^; Jennifer Donnelly^3^; Stephen Carroll^2^; Heather Hughes^2^; Barbara Cathcart^2^; Rhona Mahony^2^; Paul Downey^2^; Fionnuala M. M. McAuliffe^4^; Siobhan Corcoran^2^

*^1^University College Dublin, University College Dublin, Dublin; ^2^National Maternity Hospital, Dublin, Dublin; ^3^Rotunda Hospital, Dublin, Dublin; ^4^UCD Perinatal Research Centre, University College Dublin, Dublin 2, Dublin*

4:00 PM ‐ 6:00 PM

**Objective**: To evaluate the incidence of congenital fetal anomaly in pregnancies with placenta accreta spectrum (PAS).

**Study Design**: This is a prospective cohort study of PAS cases from January 2018–July 2024. Consecutive patients with clinically and histopathologically confirmed PAS were included. Ultrasound assessments were performed by fetal‐medicine specialists in a tertiary referral centre for PAS.

**Results**: 73 participants with a mean age of 36.0±3.6 met inclusion criteria. In seven cases (9.5%), a major fetal congenital anomaly in a euploid fetus was diagnosed, compared to a background rate of 3%. The anomalies diagnosed were as follows: two major cardiac anomalies (hypoplastic left heart, right heart), two tracheoesophageal fistula, one severe ventriculomegaly, one giant omphalocele with hydrops, and one congenital diaphragmatic hernia. No case was associated with an underlying genetic diagnosis. In each case, clinical PAS findings were on the severe end of spectrum, with low implantation in early pregnancy which evolved to intraoperative topography consistent with type 2L (low lateral parametrial involvement) and 3 (low anterior cervical).

**Conclusion**: Severe PAS may be associated with a higher rate of fetal congenital anomaly, which in our cohort were major single structural anomalies without genetic associations. PAS cases associated with congenital fetal anomalies were lateral and cervical defects on the severe end of the spectrum. These structural defects suggest fetal insult in early pregnancy from 5‐9 weeks gestation. It is possible early implantation within a previous uterine scar results in vascular disruption of embryonic development and the subsequent development of structural fetal anomaly. These cases present significant clinical challenges and warrant specialist multi‐disciplinary care to include neonatology and paediatric surgery to ensure safe delivery timing to optimise maternal health and fetal outcomes. PAS cases should have a detailed fetal medicine assessment in view of the higher incidence of fetal anomalies.

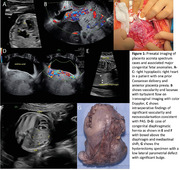

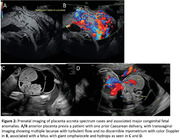

## 1044 | Assessing the Impact of Early Pregnancy Air Travel on First Trimester Miscarriage Rates

Hila Shalev‐Ram^1^; Shai Ram^2^; bitya Nehuf^3^; Netanella Miller^4^; Gal cohen^5^; Ron Sconman^6^; Gil Shechter Maor^7^; Tal Biron‐Shental^1^

*^1^Meir Medical Center, Meir Medical Center, HaMerkaz; ^2^ICHILOV, Tel Aviv, HaMerkaz; ^3^Jerusalem University, Jerusalem, Yerushalayim; ^4^Department of Obstetrics and Gynecology Meir Medical Center, Kfar Saba, HaMerkaz; ^5^Meir Hospital, Meir Hospital, HaMerkaz; ^6^Department of Obstetrics and Gynecology Meir Medical Center, Kfar Saba, Yerushalayim; ^7^Meir Medical Center, Kfar Saba, HaMerkaz*

4:00 PM ‐ 6:00 PM

**Objective**: Our study aimed to investigate the potential association between air travel and spontaneous abortions in the general population, addressing the limited and conflicting data currently available on this subject.

**Study Design**: We conducted an observational retrospective study using a large dataset comprising 469,976 live births and 76,556 spontaneous abortions from 2010 to 2019. We compared singleton pregnancies that resulted in live births with those that ended in spontaneous abortion in the first trimester, focusing on air travel during the first trimester (4w0d ‐ 13w6d) as the independent variable. The primary outcome was spontaneous abortion. A logistic regression model was used to analyze the association between the outcome and the exposure. Adjusted odds ratios (aOR) and 95% confidence intervals (CI) were reported.

**Results**: In this study, 427,727 live births (97.2%) and 12,263 (2.8%) spontaneous abortions were recorded. Women who experienced spontaneous abortions exhibited a higher incidence of air travel, with 0.11% having traveled compared to only 0.04% among those who delivered live births (P< 0.001). After adjusting for confounding factors (as detailed in Table 1), the odds ratio for spontaneous abortion was 5.20 (95% CI 4.38‐6.33) for those who traveled by air during early pregnancy.

**Conclusion**: Our study suggests that air travel during early pregnancy is associated with a higher risk of spontaneous abortion.

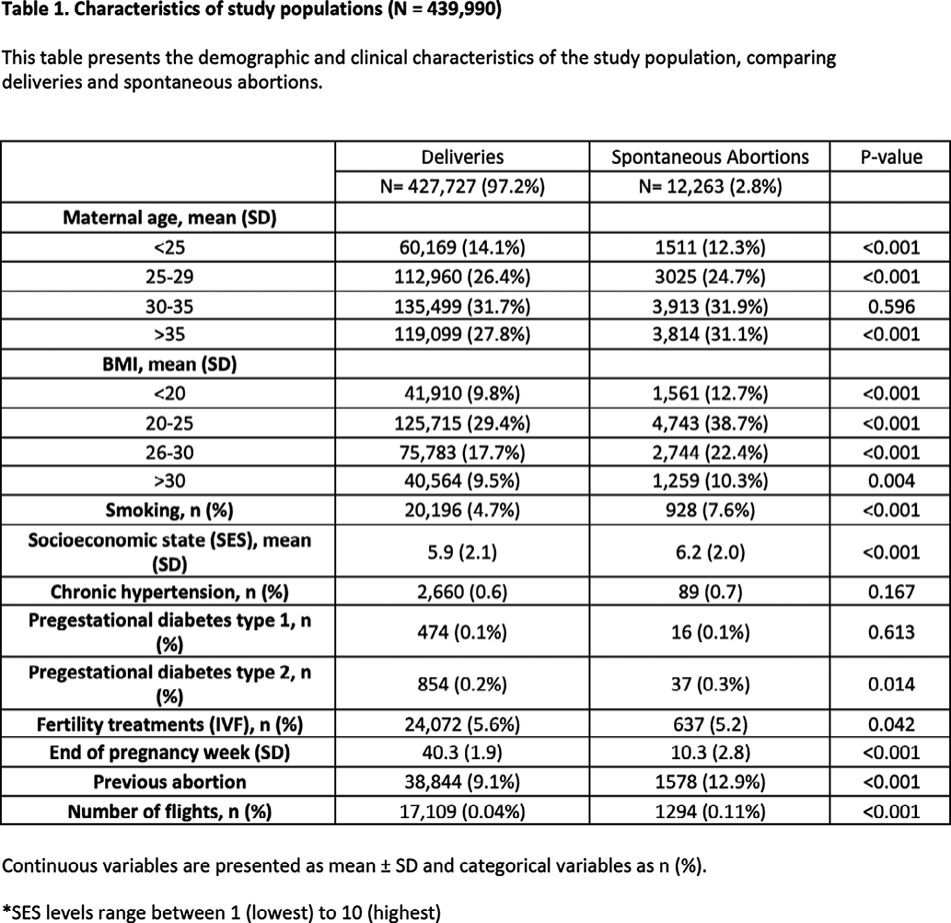

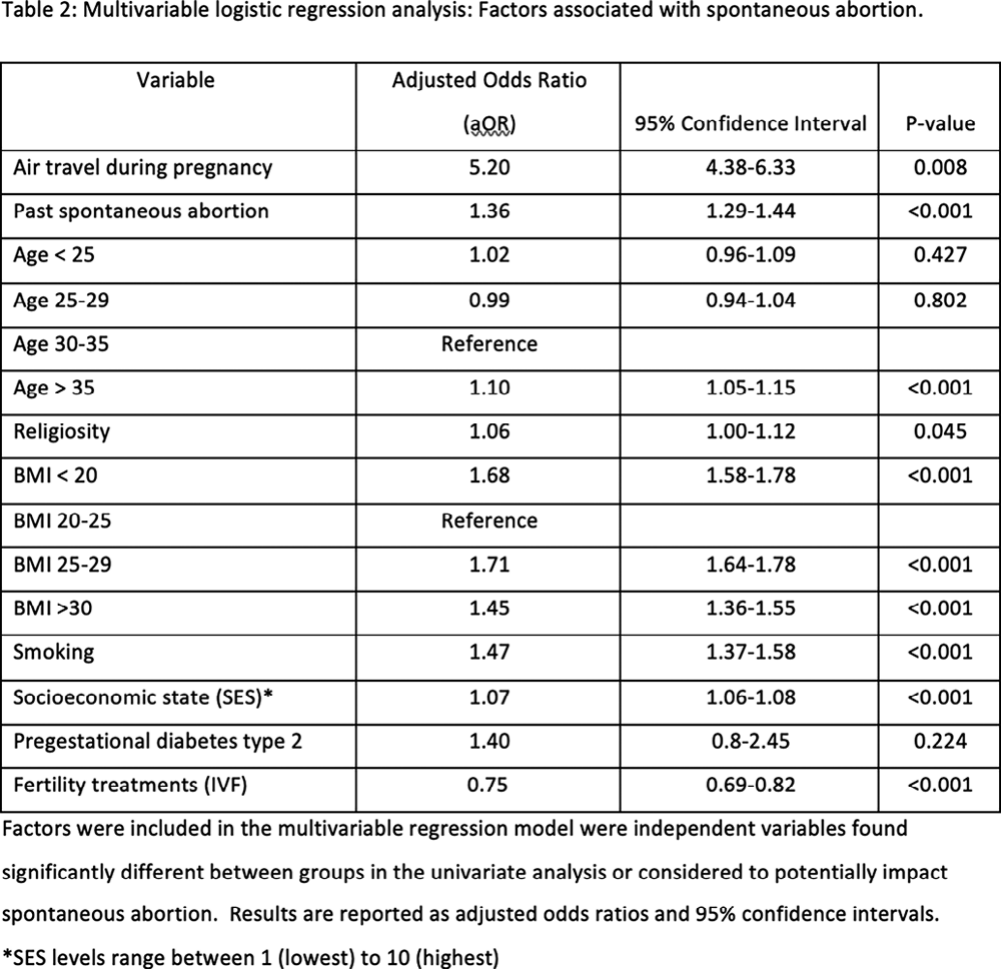

## 1045 | Utilizing the PEN‐FAST Scoring Tool in Pregnant Patients for Syphilis Management Complicated by Penicillin Allergy

Hope Brandon^1^; Noelle Leung^2^

*^1^University of Kentucky College of Pharmacy, Lexington, KY; ^2^University of Kentucky HealthCare, Lexington, KY*

4:00 PM ‐ 6:00 PM

**Objective**: Assess the PEN‐FAST scoring tool in conjunction with direct oral challenge to expedite appropriate syphilis treatment in pregnant patients reporting a penicillin allergy without increasing incidence of penicillin‐related adverse reactions.

**Study Design**: This was a single center, retrospective, case cohort study of pregnant patients with confirmed Syphilis requiring treatment and a reported penicillin allergy between August 2022 through August 2023.

**Results**: There were six patients included in analysis. Three patients had a PEN‐FAST score of zero, two of whom had direct de‐labeling of the penicillin allergy while one patient had an oral challenge followed by allergy de‐labeling. Three patients had a PEN‐FAST score of three. One of these patients had a successful oral challenge with subsequent penicillin allergy de‐labeling, one patient received an IV test dose followed by a continuous penicillin infusion and subsequent penicillin allergy de‐labeling, and one patient required admission to the intensive care unit for desensitization. All patients were successfully treated for syphilis with penicillin without adverse drug reactions, with only one requiring desensitization. Overall, four out of six patients had their penicillin allergy successfully de‐labeled as one patient had a true allergy and one declined de‐label. Patients with a documented penicillin allergy had a significant delay in time to treatment compared to patients without a documented penicillin allergy presenting with syphilis during the same time period.

**Conclusion**: Overall, the PEN‐FAST assessment was an efficient and safe tool for stratifying risk of penicillin allergies within this cohort of patients. The clinical impact of this assessment includes decreased utilization of healthcare resources and improved time to treatment for pregnant patients with syphilis. The PEN‐FAST scoring tool in combination with direct oral challenge when needed, resulted in the safe, effective, and timely treatment of six pregnant patients with syphilis.

## 1046 | Disposition Index as a Predictor of Diabetes Development in Women with Gestational Diabetes

Hiroshi Yamashita^1^; Harumi Kanzawa^1^; Ichiro Yasuhi^2^

*^1^NHO Nagasaki Medical Center, Omura City, Nagasaki; ^2^NHO Nagasaki Medical Center, Omura‐City, Nagasaki*

4:00 PM ‐ 6:00 PM

**Objective**: Gestational diabetes mellitus (GDM) increases the risk of developing type 2 diabetes mellitus (DM) postpartum. The disposition index (DI), reflecting pancreatic beta‐cell function in response to insulin resistance, is a potent predictor of diabetes development outside pregnancy. This study aimed to evaluate the utility of early postnatal DI in predicting DM development within three years postpartum in women with GDM.

**Study Design**: This prospective cohort study included singleton pregnant Japanese women diagnosed with GDM during pregnancy. An oral glucose tolerance test (OGTT) was conducted at 6 to 9 weeks postpartum and repeated every 6 to 12 months. The primary outcome was the development of DM within three years postpartum. Using early postpartum OGTT results, we calculated the Matsuda index (MI) and AUCins/AUCglu (area under the curve of insulin/glucose) over 120 minutes as indices of insulin sensitivity and secretion, respectively, and DI (MI x AUCins/AUCglu). We examined the association between DI and DM development, adjusting for potential confounders including age, family history of DM, insulin treatment during pregnancy, obesity, and breastfeeding.

**Results**: Among 363 women with GDM (mean age 34 ± 5 years, prepregnancy BMI 23.7 ± 5.0), 14 developed DM (3.9%). DI was significantly lower in the DM group compared to the non‐DM group (1.17 ± 0.30 vs. 1.83 ± 0.65, p < 0.01). A low DI was significantly associated with DM development (**Figure**). Women with a DI less than 1.58, derived from the receiver operating characteristic curve, had significantly higher odds of developing DM (crude odds ratio 20.6, adjusted odds ratio 12.5; 95% confidence intervals 4.0‐376.3 and 2.3‐234.6, respectively). Neither MI nor AUCins/AUCglu was significantly associated with DM development.

**Conclusion**: In Japanese women with GDM, early postnatal DI is a significant predictor of DM development within three years postpartum. Women who developed DM already had impaired beta‐cell response to insulin resistance during the early postpartum period.

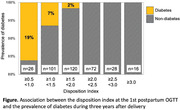

## 1047 | Association between Psychopharmacotherapy and Excessive Gestational Weight Gain

Insaf Kouba^1^; Frank I. Jackson^2^; Nathan A. Keller^2^; Luis A. Bracero^2^; Matthew J. Blitz^2^

*^1^Northwell, St. Petersburg, FL; ^2^Northwell, New Hyde Park, NY*

4:00 PM ‐ 6:00 PM

**Objective**: To determine the relationship between use of medications to treat mood disorders (psychopharmacotherapy, PPT) and excessive gestational weight gain (EGWG).

**Study Design**: This retrospective cohort study included all pregnant patients who delivered live, term, singleton newborns at seven hospitals within a large health system in New York from 2019‐2023. Patients with missing GWG data were excluded. The primary exposure was prenatal exposure to PPT (yes/no), which included selective serotonin reuptake inhibitors (SSRIs), serotonin‐norepinephrine reuptake inhibitors (SNRIs), dopamine‐norepinephrine reuptake inhibitors (DNRIs), benzodiazepines, and others. The primary outcome was EGWG (yes/no), defined as weight gain exceeding the upper limit recommended by the Institute of Medicine based on pre‐pregnancy BMI. Multiple logistic regression was performed to evaluate the association between PPT and EGWG, adjusting for covariate factors.

**Results**: A total of 87,385 pregnancies were included for analysis and EGWG occurred in 49.50% (n = 43,261). Patients with mental health conditions had EGWG more often than patients without such conditions (49.3% vs. 54.3%, respectively; aOR 1.26, 95% CI 1.10‐1.45). Among the individual PPT categories, only SSRI monotherapy was associated with increased odds of EGWG (aOR 1.21, 95% CI 1.09‐1.33) compared to patients without a mental health disorder. Patients taking two medications had higher odds of EGWG (aOR 1.62, 95% CI 1.23‐2.16) than those taking one medication (aOR 1.11, 95% CI 1.03‐1.20). Regression results are shown in Figure.

**Conclusion**: Pregnant patients with mental health disorders are at higher risk for EGWG, especially those treated with SSRIs and those receiving multiple PPT medications. Our findings suggest that the type of PPT affects GWG, underscoring the need for tailored management strategies to mitigate excessive weight gain in these patients

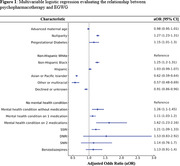

## 1048 | Patient‐reported Outcomes of Iron‐Deficiency Anemia Treatment in Pregnancy: a Mixed Methods Analysis

Irogue I. Igbinosa^1^; Ijeoma Iweakogwu^1^; Elizabeth B. Sherwin^1^; Sabrina Montgomery^2^; Caroline Berube^1^; Deirdre J. Lyell^1^

*^1^Stanford University, Palo Alto, CA; ^2^Charles R. Drew University of Medicine and Science, College of Medicine, Inglewood, CA*

4:00 PM ‐ 6:00 PM

**Objective**: Iron deficiency anemia (IDA) in pregnancy is common, yet patient‐reported outcomes on IDA treatment are limited. Our objective is to determine whether intravenous iron, compared with oral iron, improves patient‐reported outcomes in the treatment of iron‐deficiency anemia (IDA) in pregnancy.

**Study Design**: We conducted a randomized controlled trial of pregnant patients with laboratory‐confirmed IDA comparing IV ferumoxytol to oral ferrous sulfate. We assessed patient‐reported outcomes with a modified short‐form validated survey and free text feedback using a mixed methods approach. We compared the changes in survey domain scores (scale of 0‐100) in general health, physical functioning, role limitations due to physical health, and energy/fatigue from study initiation to 4 weeks between treatment groups. We analyzed free‐text responses with inductive thematic analysis and compared qualitative themes with quantitative results. Adverse side effects were monitored by survey and a Data Safety Monitoring Board.

**Results**: Among the 61/80 (76% of the total study) participants who responded to the 4‐week survey, participants in the IV ferumoxytol group noted improvements in energy/fatigue, with a median increase of 10 points over 4 weeks compared to no change in the oral ferrous sulfate group (p = 0.018) (Table 1). There were no changes in general health, physical functioning, or role limitations due to physical health at 4 weeks post‐treatment in either study group. Thematic analysis of free text responses revealed that patients generally favored IV iron (preferred IV treatment despite randomization, more energy and effectiveness, and fewer side effects) and desired anemia management (follow‐up of their hemoglobin levels) (Table 2). Analysis of quantitative and qualitative data reinforced patient preference for IV iron. There were no serious adverse events during the study period.

**Conclusion**: IV iron significantly improved patient‐reported outcomes in quality of life, specifically more energy and less fatigue, compared to oral ferrous sulfate.

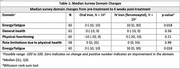

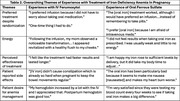

## 1049 | Relationship Between Patient Trust and Adverse Perinatal Outcomes Among Black and White Pregnant Patients

Jacklyn M. Locklear^1^; Madison Lanza, N/A^2^; Ateshi Bhatt^3^; Briasha Jones^3^; Emily Rebowe^3^; Annie Talbot^4^; Andrew G. Chapple^4^; Jill M. Maples^5^; Alicia Mastronardi^6^; Neelima Sukhavasi^4^; Elizabeth F. Sutton^3^; Kaitlyn Taylor^7^

*^1^University of Tennessee Graduate School of Medicine, Department of OB/GYN, Knoxville, TN; ^2^LSU Health Science Center, Baton rouge, LA; ^3^Woman's Hospital, Baton Rouge, LA; ^4^LSU Health Science Center, New Orleans, LA; ^5^University of Tennessee Health Science Center, College of Medicine, Knoxville, TN; ^6^University of Tennessee Graduate School of Medicine, Department of OB/Gyn, Knoxville, TN; ^7^UAMS, UAMS/Little Rock, AR*

4:00 PM ‐ 6:00 PM

**Objective**: To evaluate trust in physicians among different self‐reported patient races and evaluate if trust was a contributing factor to adverse perinatal outcomes by evaluating the interplay of trust, patient race and a severe maternal morbidity composite.

**Study Design**: The 10‐item Wake Forest Physician Trust Scale (WFPTS) was administered to pregnant individuals from July 2021‐ January 2022 (score 10‐50; higher scores indicating higher trust) in an academic, primarily Medicaid‐funded, OB/GYN practice in Baton Rouge, Louisiana. The survey also included demographic information including self‐reported race, household income, and education. Pregnancy and delivery data were abstracted from medical records and coded into a binary adverse perinatal outcome. The composite outcome included any of the following events: transfusion, hospital readmission, intensive care unit admission, non‐live birth, PPROM, and pre‐term birth. Categorical variables were compared using Fisher exact tests and continuous variables using Wilcoxon rank sum tests. Logistic and Quasi‐Poisson regressions were used to adjust for confounding effects.

**Results**: Average WFPTS score was 40.1±4.7, indicating moderate‐high trust. There was no difference in trust scores between Black patients compared to all other races (40.5±4.5 vs 39.4±4.9, p = 0.116), which held after confounding adjustment for income, age, and education. The adverse perinatal outcome composite occurred in 19% (n = 37) of the study cohort. There were no significant associations between the composite adverse outcome and patient race (aOR 1.65, 95% CI 0.64‐4.28, p = 0.301) or trust score (aOR 1.03, 95% CI 0.95‐1.12, p = 0.451).

**Conclusion**: This study showed high levels of trust in physicians across all racial groups. Neither trust score nor race was related to adverse perinatal outcomes in our cohort.

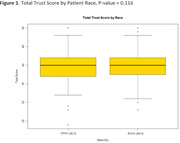

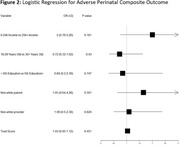

## 1050 | Obesity Remains Associated with Cesarean Delivery Despite Combined Cervical Ripening

Jacqueline J. Thompson^1^; Lisa D. Levine^2^; Celeste Durnwald^1^; Rebecca F. Hamm^2^

*^1^Hospital of University of Pennsylvania, Philadelphia, PA; ^2^University of Pennsylvania Perelman School of Medicine, Philadelphia, PA*

4:00 PM ‐ 6:00 PM

**Objective**: Prior studies demonstrate that patients with obesity have increased risk of cesarean delivery (CD) and longer inductions of labor (IOL), however few studies have evaluated this in the context of combination cervical ripening methods.

**Study Design**: This is a secondary analysis of prospective cohort study of term, singletons with intact membranes and unfavorable cervix undergoing standardized IOL at 2 sites from 2018‐2022. This analysis included only IOLs who utilized combined cervical ripening (Foley + either misoprostol or oxytocin concomitantly). Patients were stratified by BMI (< 35 vs. ≥35kg/m2). Primary outcomes included CD and labor length. Poisson regression with robust error variance was used to calculate adjusted relative risks (aRR) for CD. Time‐to‐event regression analyses for labor length, censored for CD, was modeled with a Cox proportional hazard model. Secondary outcomes included time Foley in, time utilizing misoprostol if applicable, dilation at and time to amniotomy, and maximum dose of oxytocin.

**Results**: 4,263 patients were included (BMI< 35 = 2,819, ≥35 = 1,444). BMI groups differed by hospital site, race, insurance, parity, diagnosis of diabetes and hypertension, modified Bishop score, maternal age, gestational age, and IOL indication. Even when controlling for differences between groups, patients with BMI ≥35 had a 64% higher risk of CD than those with BMI< 35 (24.0% v. 15.3%; aRR1.64[1.41‐1.90]. BMI ≥35 was not associated with differences in length of labor (HR 1.11[0.96‐1.29]). While patients with obesity had a similar time utilizing Foley and misoprostol, they had longer time to and were less dilated at amniotomy. Higher maximum oxytocin doses were also utilized in those with BMI > = 35.

**Conclusion**: Even in the setting of combined cervical ripening methods, patients with BMI ≥35 are at increased risk of CD, although there is no difference in labor length. As our data is indicative of lower success of ripening with BMI > = 35, innovative solutions for IOL are needed to improve CD rate for patients with obesity.

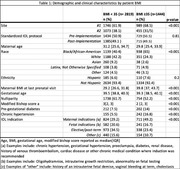

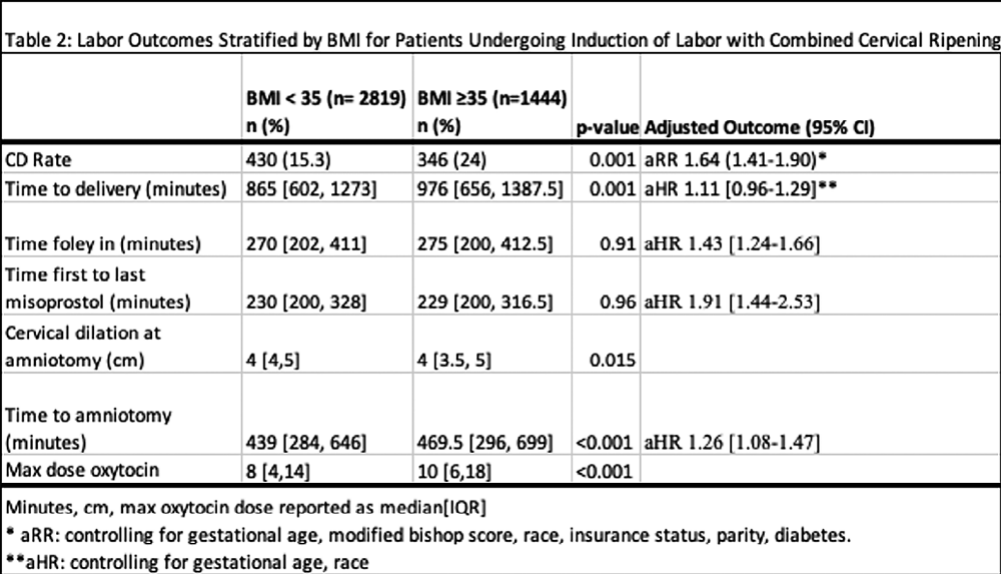

## 1051 | Maximum Oxytocin Dose in Labor Induction and Associated Pregnancy Outcomes in Nulliparous Individuals

Jae Baek^1^; Marissa J. Berry^1^; Patrick Schneider^2^; Maged M. Costantine^1^; William A. Grobman^1^; Heather A. Frey^1^

*^1^The Ohio State University, Columbus, OH; ^2^The Ohio State University, Wexner Medical Center, Columbus, OH*

4:00 PM ‐ 6:00 PM

**Objective**: Oxytocin is often used for induction of labor (IOL) with a lack of evidence‐based guidelines on its maximum dose. In some institutions, 20 milliunits/minute (mU/min) is used as a maximum dose due to concerns about adverse outcomes at higher rates. We sought to evaluate the relationship between the maximum dose of oxytocin with maternal and neonatal outcomes in low‐risk nulliparous individuals.

**Study Design**: This secondary analysis of the MFMU ARRIVE trial included nulliparous individuals who underwent IOL. The primary exposure was maximum oxytocin dose categorized as ≤ 20 or > 20 mU/min. Logistic regression was used to adjust for gestational age, body mass index, induction without medical indication, Bishop score on admission, and hypertensive disorders of pregnancy.

**Results**: Of the 3,565 participants included in this secondary analysis, 2,673 (75%) were exposed to a maximum dose of oxytocin of ≤ 20 mU/min and 892 (25%) were exposed to a maximum dose of > 20 mU/min. Those who were exposed to > 20 mU/min of oxytocin had a greater median BMI (p< 0.001) and a lower Bishop score on admission (p< 0.001) (Table 1). Duration from initiation of oxytocin to delivery was longer in the higher‐dose group compared to the lower‐dose group (median[IQR] ≤ 20 mU/min: 12.1[8.2‐17] vs. > 20 mU/min: 21.2 [15.9‐257] hours; p< 0.001). Individuals exposed to < 20 mU/min of oxytocin had higher rates of cesarean delivery (adjusted odds ratio [aOR] 1.23, 85%CI 1.01‐1.48), cesarean deliver for labor dystocia (aOR 1.63, 95%CI 1.28‐2.07), and postpartum hemorrhage (aOR 1.51, 95%CI 1.09‐2.09) (Table 2). There was no difference in rates of cesarean delivery for non‐reassuring fetal status (aOR 0.80, 95%CI 0.62‐1.03), NICU admissions (aOR 1.03 95%CI 0.82‐1.30), or neonatal adverse outcomes (aOR 0.97, 95%CI 0.68‐1.39).

**Conclusion**: This analysis showed no difference in rates of cesarean delivery for non‐reassuring fetal status or adverse neonatal outcomes at oxytocin rates > 20 mU/min, suggesting that 20 mU/min should not be used as a maximum dose during IOL of nulliparous individuals.

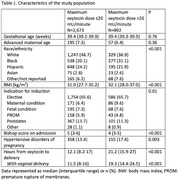

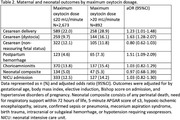

## 1052 | Assessment of Disparities in Cerclage Placement Among Individuals with a History of Preterm Birth

Jamie Green; Sarah H. Abelman; Frank I. Jackson; Nathan A. Keller; Julie Chen; Luis A. Bracero; Matthew J. Blitz

*Northwell, New Hyde Park, NY*

4:00 PM ‐ 6:00 PM

**Objective**: To evaluate the association between rates of cerclage placement and race and ethnicity group, primary language, and insurance type among patients with a history of preterm birth.

**Study Design**: This was a retrospective cohort study within a large healthcare system in New York. Patients with were included who had screening cervical length measurements between 16 weeks 0 days and 23 weeks 6 days of gestation and a history of preterm birth due to any indication. Multivariate logistic regression was used to compare rates of cerclage placement by race and ethnicity group, English as primary language, Medicaid insurance, and the presence of a very short cervix < 10mm. All statistical analyses were performed in R 4.3.1.

**Results**: A total of 3,988 pregnancies in patients with a history of preterm birth were included. A cerclage was placed during 1228 (30.8%) of these pregnancies, and not in the remaining 2760 (69.2%). There were no significant differences in the rates of cerclage placement based on race and ethnicity groups, primary language, or insurance type. Very short cervical length (< 10mm) was associated with a significantly higher rate of cerclage placement (aOR 4.04, 95% CI 2.97‐5.51).

**Conclusion**: Systemic disparities in rates of cerclage placement among patients with a history of preterm birth on the basis of race and ethnicity group, primary language, and insurance type were not identified in this cohort. Patients with a history of preterm birth and a cervical length < 10mm have increased odds of receiving a cerclage.

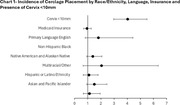

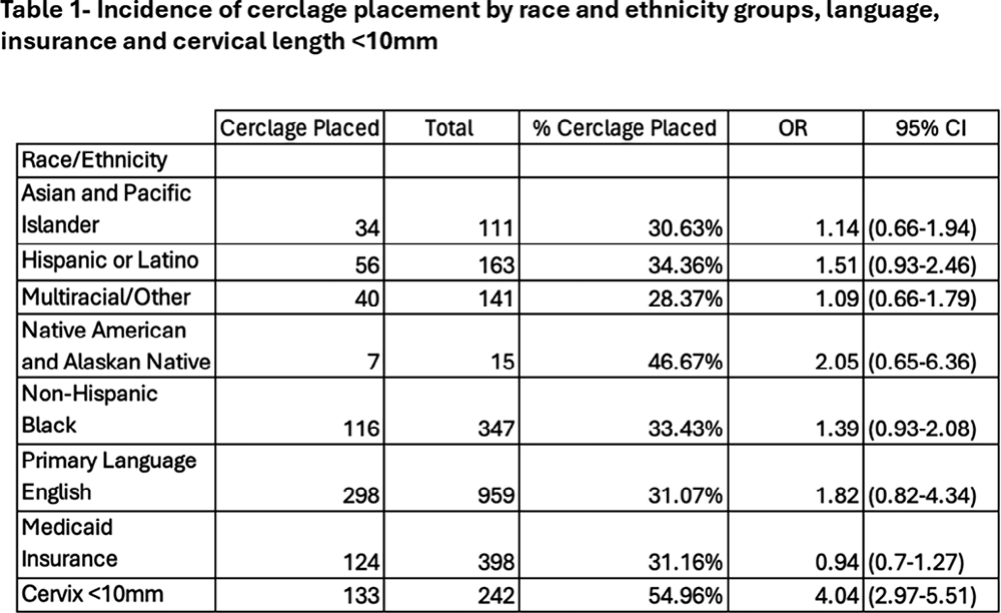

## 1053 | Migrating while Pregnant: A Qualitative Study of Immigration and Obstetric Health

Jecca R. Steinberg^1^; Hebron Kelecha^1^; Madeline F. Perry^1^; Allie Valenzuela^1^; Nivedita Potapragada^1^; Erica O'Neill^2^; Ashish Premkumar^3^; Lynn M. Yee^1^

*^1^Northwestern University Feinberg School of Medicine, Chicago, IL; ^2^Stroger Hospital Cook County, Chicago, IL; ^3^Pritzker School of Medicine, University of Chicago, Chicago, IL*

4:00 PM ‐ 6:00 PM

**Objective**: The recent influx of migrants to select regions of the United States (US) has reshaped the demographic landscape and need for social and health services. We aimed to understand the experiences of pregnant people during immigration and in the domestic migration system.

**Study Design**: This qualitative study applied inductive methods to explore the perspectives of pregnant and postpartum migrants in Chicago, Illinois, a sanctuary city and frequent destination for migrants transported from Texas. To design interviews, we engaged with patients, advocates, physicians, nurses, and social workers through community‐based participatory methods. Participants were recruited from a public hospital prenatal clinic and completed validated demographic surveys and semi‐structured interviews in Spanish or English. We applied iterative coding of transcripts to develop a codebook, evaluate interrater reliability (Cohen's kappa >0.85), and analyze themes.

**Results**: Of 12 participants (ages 19‐39), 11 identified as Latine and one as Black. Six were in the third trimester, 5 in the second and 1 postpartum. Half had attended or completed university prior to migration. Most were publicly insured (7/12) and the remainder were uninsured (5/12). Participants migrated from Venezuela, Colombia, and Sierra Leone. Interviews revealed that pregnant people face a number of health‐harming migration‐related circumstances, including: (1) unique obstetric health challenges during migration; (2) significant food insecurity; (3) hazardous conditions in shelters; (4) family separation; (5) limited access to income during pregnancy (largely due to the physically demanding nature of work in the informal sector); and (6) limited access to legal support (Table). When pregnant migrants faced hardship, scarce options were available to improve their circumstances and health.

**Conclusion**: Pregnant individuals migrating to the US face unique challenges that may negatively influence obstetric health. The current system for migrant support struggles to provide basic obstetric necessities, such as adequate safety, nutrition, or sanitation measures.

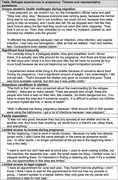

## 1054 | Postpartum Hospital use in the 90 days after Delivery

Jecca R. Steinberg; Mahika Gopalka; Samanvi Kanugula; Lynn M. Yee; Joe M. Feinglass

*Northwestern University Feinberg School of Medicine, Chicago, IL*

4:00 PM ‐ 6:00 PM

**Objective**: We aimed to perform a health system‐wide analysis of sociodemographic, clinical, and hospital‐level factors associated with 90‐day postpartum emergency department (ED) visits and inpatient hospital admissions.

**Study Design**: This cross‐sectional study of all 90‐day postpartum hospital ED visits and inpatient admissions (“90‐day readmission”) in a nine‐hospital Midwest health system included all births from 1/2018‐6/2023. We applied a multilevel eco‐social framework to examine associations between factors in three domains and 90‐day readmission. Exposure variables included sociodemographic (age, race/ethnicity, insurance, language, census zip code percent poor households), clinical (body mass index [BMI], severe maternal morbidity during delivery admission [SMM], and other comorbidities), and hospital‐level factors. We applied bivariate and adjusted Poisson regression analyses.

**Results**: Of 104,076 deliveries, 6,879 (6.6%) were followed by 90‐day readmission. In bivariate analysis, chronic hypertension, substance use disorder, BMI >40, and SMM demonstrated the strongest association with 90‐day readmission (Figure). After adjusting for all factors under investigation, of sociodemographic factors, Medicaid insurance (adjusted incidence rate ratio [aIRR] 1.43; 95% confidence interval [CI] 1.34‐1.45) and Black race (aIRR 1.35; CI 1.25‐1.46) were associated with the greatest 90‐day readmission risk. SMM remained most strongly associated with 90‐day readmission of any factor (aIRR 1.95; 95% CI 1.75‐2.18). Chronic hypertension (aIRR 1.59; CI 1.49‐1.70) and BMI >40 (aIRR 1.52; CI 1.36‐1.70) both had elevated 90‐day readmission risk. Primiparity and all other medical comorbidities had relatively weaker associations (Figure). At the hospital level, delivery at a suburban or community hospital was associated with greater 90‐day readmission risk than delivery at the academic medical center.

**Conclusion**: In this health system‐wide analysis, 90‐day readmission is driven by a combination of sociodemographic, clinical, and hospital‐level factors, underscoring the need for a comprehensive mitigation strategy.

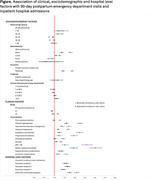

## 1055 | Frequency and Type of Chromosome Abnormalities in Stillbirths: Insights from More Than 4000 Cases

Vivienne Souter^1^; Katrina Merrion^1^; Madeleine Armer‐Cabral^1^; Katherine Howard^1^; Melda Balcioglu^1^; Kathryn J. Gray^2^; Peter Benn^3^

*^1^Natera, Inc., San Carlos, CA; ^2^University of Washington, Seattle, WA; ^3^UCONN Health, Farmington, CT*

4:00 PM ‐ 6:00 PM

**Objective**: Approximately 1 in 166 pregnancies in the US result in stillbirth (fetal demise >20 weeks’ gestation) and only a minority have a postmortem examination or genetic evaluation. Our objective was to evaluate chromosomal abnormalities detected by single nucleotide polymorphism (SNP)‐based chromosomal microarray (CMA) in products of conception (POC) from a large cohort of stillbirths.

**Study Design**: Retrospective review of 4,990 fresh POC specimens from singleton pregnancies at ≥ 20 weeks’ gestation evaluated 2010‐2022. Known pregnancy terminations and livebirths were excluded (278 cases). Illumina CytoSNP‐12b SNP‐based CMA was used to detect maternal cell contamination (MCC), aneuploidy, triploidy, copy number variants (CNVs), uniparental disomy (UPD), mosaicism, and parental origin of abnormalities.

**Results**: Of 4,712 samples, 244 (5.2%) had MCC and 37 (0.8%) had an inconclusive result leaving 4,431 samples for analysis. Of these, 517 (11.7%) were abnormal (Table). Median maternal age was 30 and 31 years for normal and abnormal cases, respectively, and median gestational age was 25 and 24 weeks. The percentage of abnormal results increased with maternal age (*p* < 0.001) but not gestational age (*p* = 0.34). Triploidy was not observed after 31 weeks’ gestation. Common autosomal trisomies (T21, T18, T13) were present in 3.9%; monosomy X, 2.6%; CNVs with one or more regions involved, 3.2%; triploidy, 1.0%; and single chromosome UPD, 0.2% (Table).

**Conclusion**: Here we report a large dataset of CMA analyses in stillbirth. CMA was successfully performed in approximately 95% of cases (MCC explained most of those without a result). More than 1 in 10 cases had an abnormal finding with a spectrum of abnormalities intermediate between those seen in livebirths and those present in early gestation miscarriages. Many of the findings had implications for future **r**eproductive risk. CMA was useful across all maternal ages and all gestational ages at the time of stillbirth.

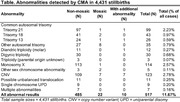

## 1056 | Perinatal Outcomes in Patients with Diagnosed and Undiagnosed Depression

Jessica Britt^1^; Moonseong Heo^2^; Lu Zhang^2^; Amy Crockett^3^

*^1^Prisma Health, Prisma Health, SC; ^2^Clemson University, Clemson, SC; ^3^Prisma Health and University of South Carolina School of Medicine, Greenville, SC*

4:00 PM ‐ 6:00 PM

**Objective**: Each year in the US, 20% of pregnant people suffer from perinatal mental health conditions; up to 75% never receive treatment. The adverse impact of undiagnosed depression is likely underestimated. We aimed to examine the association between diagnosed and undiagnosed depression and birth outcomes.

**Study Design**: This is a secondary analysis of the CRADLE randomized clinical trial which compared birth outcomes in group vs. individual prenatal care. Patients were included if they completed the Center for Epidemiological Studies‐Depression (CES‐D) tool and birth outcome data were available. A CES‐D score ≥23 was considered positive, and the clinical team was blinded to results. Clinically diagnosed depression (CD) was identified via chart review. Providers used patient history or the Edinburgh Postnatal Depression tool if CD was suspected; universal screening was not being utilized at the time. Patients were classified based on CD during pregnancy and CES‐D scores: 1) no depression (no CD and negative CES‐D; CON); 2) depression (CD with any CES‐D score; DIAG); 3) undiagnosed depression (no CD but positive CES‐D; CESD+). The primary outcome was preterm birth (< 37 weeks). Secondary outcomes were low birthweight (< 2,500g), NICU admission, and APGAR < 7 at 1 and 5 minutes. Multivariable logistic regression was performed with CON as the reference and adjustments for race, maternal age, nulliparity, and pregnancy intention.

**Results**: 1765 patients were included: 1380 (78%) in the CON group, 202 (12%) in DIAG, and 184 (10%) in CESD+. CESD+ patients were more likely to be younger, Black, nulliparous, and have an unintended pregnancy (Table 1). CESD+ patients had increased odds of preterm birth (OR 2.50, 95% CI 1.53‐4.10) compared to CON. CESD+ patients had increased odds of all secondary outcomes compared to CON while DIAG had increased odds of low birthweight and NICU admission (Table 2).

**Conclusion**: Diagnosed and undiagnosed depression is associated with adverse birth outcomes. Increased screening efforts using validated instruments during pregnancy are warranted and may help improve racial equity.

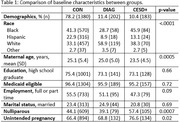

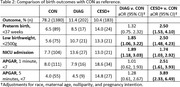

## 1057 | Healthy Placental or Umbilical Cord Stem Cell Extracellular Vesicles Restore Preeclamptic Cells to Normal Function

Jessica Greenberg^1^; Shauna F. Williams^2^; Lauren Sherman^3^; Pranela Rameshwar^3^; Bobak Shadpoor^3^

*^1^Rutgers New Jersey Medical School, South Orange, NJ; ^2^Rutgers New Jersey Medical School, Newark, NJ; ^3^Rutgers, Rutgers, NJ*

4:00 PM ‐ 6:00 PM

**Objective**: Preeclampsia (PE) contributes to pregnancy‐related morbidity and mortality, with inflammation a critical factor in PE pathophysiology. Healthy placenta mesenchymal stem cells (P‐MSC) mitigate inflammation. Our group reported on PE P‐MSC dysfunction, characterized by decreased anti‐inflammatory licensing, cell cycle dysregulation, and reduced immune suppressive cytokine production. Aspirin (ASA) treatment remedied these via epigenetic reprogramming. This study hypothesized extracellular vesicles (EV) from healthy P‐MSCs or umbilical cord (UC) blood could reset PE P‐MSCs to a healthy phenotype, improving cell cycle dysregulation and anti‐inflammatory capacity.

**Study Design**: P‐ and UC‐MSCs were isolated from healthy and PE pregnancies at delivery. EVs from healthy MSCs were isolated by differential ultracentrifugation and quantified. PE P‐MSCs were exposed to EVs or treated with 1mM ASA for 48h. P‐MSCs were added as third‐party cells in mixed lymphocyte reaction (MLR) to assess anti‐inflammatory capacity. Treated P‐MSCs were analyzed for the epigene regulator TDG and cell cycle linked CDK4, p21, and p53 proteins.

**Results**: EV‐ and ASA‐treated PE P‐MSC suppressed MLR to levels of healthy P‐MSCs. While ASA increased p21, p53, and TDG, and decreased CDK4, EV treatment decreased their expression. Of note, p53 and TDG remained elevated as compared to healthy P‐MSCs while p21 was decreased.

**Conclusion**: PE P‐MSC can be restored towards healthy anti‐inflammatory function. The healthy EVs reduced PE‐MSC cell cycle with evidence of changes in DNA methylation regulation. These findings are consistent with MSCs exhibiting tissue maintenance during insult. In total, healthy P‐MSC EVs may restore the function of PE P‐MSCs, as well as other insults during pregnancy. These findings suggest off‐the‐shelf availability of EVs to treat PE. Combined with prior data on ASA restoring MSC function, this study furthers our understanding of possible methods to reduce PE via anti‐inflammatory modality.

## 1058 | Development and Validation of Ensemble Model for Fetal Birthweight Prediction in Third‐Trimester Pregnant Women

Jing Gao^1^; Zhongzhou Xiao^2^; Jie Xu^2^; Weiwei Cheng^3^

*^1^International Peace Maternity and Child Health Hospital, Shanghai, Shanghai; ^2^Shanghai Artificial Intelligence Laboratory, Shanghai, Shanghai; ^3^International Peace Maternal and Child Health Hospital, Shanghai, Shanghai*

4:00 PM ‐ 6:00 PM

**Objective**: On the basis of clinical big data, we aim to develop a machine learning (ML) model for accurate prediction of birth weight in the third trimester of pregnancy, which can help reduce adverse maternal and fetal outcomes.

**Study Design**:  From 1 January 2018 to 31 December 2019, a retrospective cohort study involving 16655 singleton live births without congenital anomalies (> 28 weeks of gestation) was conducted in a tertiary first‐class hospital in Shanghai. The initial set of data was divided into a train set for algorithm development and a test set on which the algorithm was divided in a ratio of 4:1. We extracted maternal and neonatal delivery outcomes, as well as parental demographics, obstetric clinical data, and sonographic fetal biometry, from electronic medical records. Five basic machine learning algorithms, including Ridge, SVM, Random Forest, XGBoost, and Multi‐Layer Perceptron, were used to develop the prediction model, which was then averaged into an ensemble learning model. The models were compared using accuracy, mean squared error, root mean squared error, and mean absolute error.

**Results**: Train and test sets contained a total of 13324 and 3331 cases, respectively. From a total of 59 variables, we selected 17 variables that were readily available for the “few feature model” which achieve high predictive power with an accuracy of 81.84% and significantly exceeds ultrasound formula methods. In addition, our model maintained superior performance for low birth weight and macrosomic fetal populations.

**Conclusion**:  Our research investigated an innovative artificial intelligence model for predicting fetal birthweight and maximizing healthcare resource utilization. In the era of big data, our model improves maternal and fetal outcomes and promotes precision medicine.

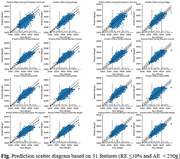

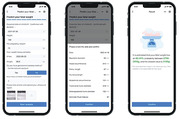

## 1059 | Increased BMI Associated with Decreased Breastfeeding Initiation in Million Veteran Program Participants

Joanna Lankester^1^; Rodrigo Guarischi‐Sousa^2^; Austin T. Hilliard^2^; Labiba Shere^2^; Marya Husary^2^; Susan Crowe^3^; Philip S. Tsao^2^; David H. Rehkopf^3^; Themistocles L. Assimes^2^

*^1^Palo Alto Veterans Affairs/Stanford University, Palo Alto, CA; ^2^Palo Alto Veterans Affairs, Palo Alto, CA; ^3^Stanford University, Stanford, CA*

4:00 PM ‐ 6:00 PM

**Objective**: Breastfeeding has been associated with maternal and infant health benefits but has been inversely associated with body mass index (BMI) prepartum. Breastfeeding and BMI are both linked to socioeconomic factors. The objective of this study was to disentangle the underlying biological association between BMI and breastfeeding initiation using a genetic predictor of BMI.

**Study Design**: Data from parous female participants with available breastfeeding information from the Million Veteran Program cohort was included (n = 20,375). BMI at enrollment and earliest BMI available were extracted, and polygenic scores (PGS) for BMI were calculated. We modeled breastfeeding for one month or more as a function of BMI at enrollment; earliest BMI where available pre‐pregnancy; and PGS for BMI. We conducted Mendelian randomization for breastfeeding initiation using PGS as an instrumental variable.

**Results**: A higher BMI predicted a lower likelihood of breastfeeding for one month or more in all analyses. A +5 kg/m^2^ BMI pre‐pregnancy was associated with a 24% reduced odds of breastfeeding, and a +5 kg/m^2^ genetically predicted BMI was associated with a 17% reduced odds of breastfeeding.

**Conclusion**: BMI predicts a lower likelihood of breastfeeding for one month or longer. The impact of breastfeeding on later BMI is an important future direction of research. Given the high success of breastfeeding initiation regardless of BMI in supportive environments as well as potential health benefits, patients with elevated BMI may benefit from additional postpartum breastfeeding support.

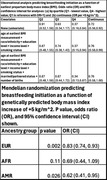

## 1060 | Concordance of Maternal GBS DNA‐PCR Rectovaginal test at 36 weeks and Maternal/Neonatal Testing at Delivery

Amanda Peña^1^; Meredith Rochon^1^; Guillermo De La Vega^1^; Kara Madey^2^; Kyle Shaak^3^; Joanne N. Quiñones^1^

*^1^Lehigh Valley Health Network, Allentown, PA; ^2^University of South Florida Morsani College of Medicine, Tampa, FL; ^3^Network Office of Research and Innovation, Lehigh Valley Health Network, Allentown, PA*

4:00 PM ‐ 6:00 PM

**Objective**: GBS is an important cause of neonatal infectious morbidity. Universal GBS screening via rectovaginal test in the late third trimester, with intrapartum antibiotic prophylaxis if GBS positive, decreases the risk of neonatal GBS infection. The effectiveness of this strategy depends on the predictive nature of this result, and GBS point‐of‐care (POC) testing at the time of delivery admission has been proposed as a possible strategy to further decrease neonatal risk. The goal of this study was to determine discordance between a negative GBS test at 36 weeks and at the time of delivery using GBS DNA‐PCR.

**Study Design**: Prospective pilot study of pregnant persons with a negative GBS DNA‐PCR rectovaginal test on routine screen at 36 weeks intending to deliver vaginally at the study institution from 3/2023‐4/2024. On admission for delivery, repeat maternal rectovaginal GBS DNA‐PCR was performed; immediately after delivery, GBS DNA‐PCR testing was performed on the placenta and neonatal ear. Results were not disclosed to the clinical team and intrapartum/neonatal care was otherwise routine. Primary outcome was GBS positive rate. Planned sample size was 60. Study supported by an institutional grant.

**Results**: 39 patients were enrolled; 9 did not complete the study (tests not performed as planned, subject withdrew or delivered elsewhere) for 30 completed subjects. Recruitment was terminated early due to slow enrollment. Most subjects were White (80.0%), non‐Hispanic (86.7%), and multiparous (60.0%), Table. Mean gestational age at delivery was 40.3±0.68 wks and 93.3% delivered vaginally. Of the 90 GBS DNA‐PCR study tests performed, 2/90 (2.2%) were positive in 2 different patients–1 maternal rectovaginal and 1 neonatal ear–for a GBS positive rate of any result in 2/30 (6.7%) patients. There were no cases of neonatal GBS sepsis.

**Conclusion**: Maternal and neonatal GBS DNA‐PCR testing results done at 36 weeks and at the time of delivery are highly concordant. Our findings suggest that the addition of GBS POC testing at the time of delivery admission is likely low yield and would add additional cost.

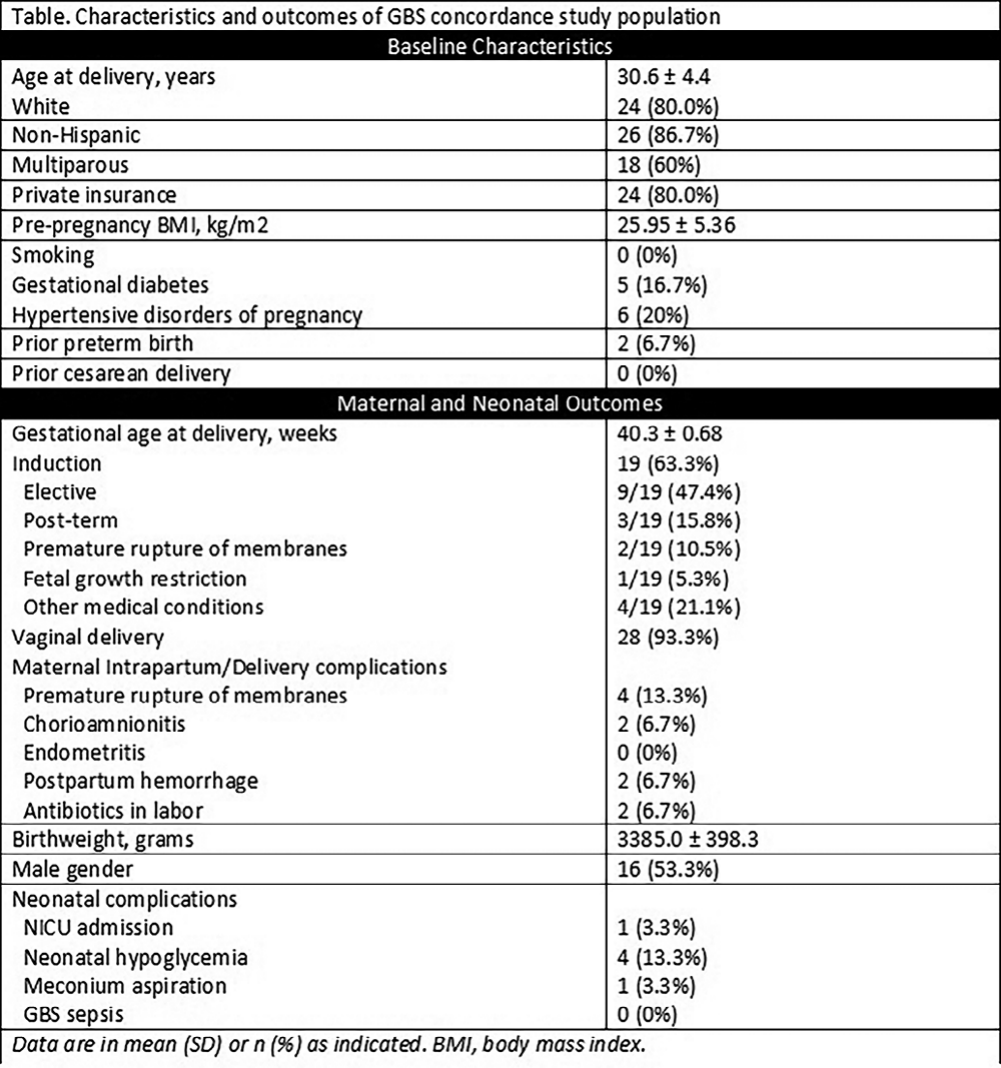

## 1061 | How does Needing an Interpreter Affect Outcomes among Primarily Non‐English Speaking Patients?

Jocelyn Reckford^1^; Nandini Raghuraman^1^; Antonina I. Frolova^2^; Amanda C. Zofkie^1^; Sherri Jackson^1^; Katherine H. Bligard^1^; Jeannie C. Kelly^1^

*^1^Washington University School of Medicine in St. Louis, St. Louis, MO; ^2^Washington University School of Medicine, St. Louis, MO*

4:00 PM ‐ 6:00 PM

**Objective**: The need for interpreter services has been linked to poor health outcomes but remains understudied in the obstetric population. We sought to compare outcomes among patients whose primary language was not English between those who used an interpreter and those who did not.

**Study Design**: We performed a retrospective cohort study of all patients delivering at an urban tertiary care center between 2021‐2023 who self‐reported their primary language as anything other than English. Patients were dichotomized between those who used an interpreter versus those who did not; all patients with non‐English as a primary language are offered interpreter services. Our primary outcomes were mode of delivery, gestational age at delivery, and low birth weight (LBW, < 2500g); secondary outcomes included postpartum hemorrhage (PPH), shoulder dystocia, APGAR at 5 minutes, and perineal lacerations. Mann‐Whitney U, chi‐square, fisher exact, and multivariable logistic regression were performed appropriately.

**Results**: 767 patients reported a primary language other than English; 396 (51.6%) did not use an interpreter and 371 (48.4%) did. There were lower rates of Hispanic ethnicity (28% vs 43%, p< 0.001) and gestational hypertension (11% vs 16%, p = 0.010), and higher rates of pre‐existing diabetes (4.3% vs 1.3%, p = 0.035) among patients who used interpreters, as well as differences in self‐reported race between groups (Table 1). There were no differences in age, parity, BMI, or rates of chronic hypertension. Gestational age at delivery and LBW did not differ between cohorts (Table 2); most patients delivered at term with birthweights >2500g. However, there were increased odds of cesarean (aOR 1.08, 95% CI 1.02‐1.14) and PPH (aOR 1.61, 95% 1.43‐1.82) in the cohort who used an interpreter.

**Conclusion**: For delivering patients whose primary language was not English, needing an institutional interpreter was associated with a higher risk of cesarean delivery and PPH. These results suggest that language barriers in the labor unit are an important target to improve outcomes, especially for those who require an interpreter.

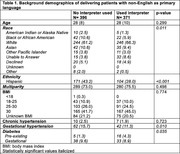

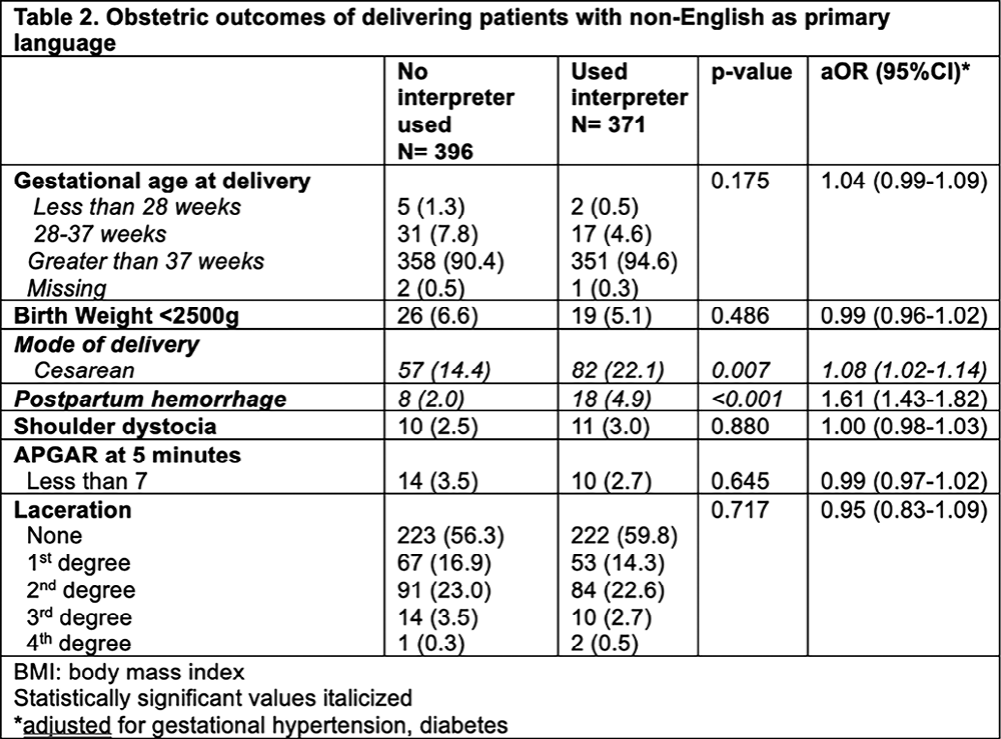

## 1062 | Routine Genetic Carrier Screening Practices During Prenatal Care and Perceptions Regarding Expanded Carrier Screening

Joseph Levy^1^; Carolyn Chatterton^2^; Melissa Hicks^3^; Devika Maulik^1^

*^1^University of Missouri‐Kansas City, Kansas City, MO; ^2^Detroit Medical Center/Wayne State University, Detroit, MI; ^3^Detroit Medical Center / Wayne State University, Detroit, MI*

4:00 PM ‐ 6:00 PM

**Objective**: The American College of Obstetricians and Gynecologists (ACOG) and the American College of Medical Genetics (ACMG) disagree on prenatal genetic carrier screening (GCS) guidelines. ACOG recommends routine screening for 2 genes plus risk based screening, while ACMG recommends screening for genes with a ≥1/200 carrier frequency. We sought to understand current GCS practices, and perceptions of expanded genetic carrier screening (EGCS).

**Study Design**: An electronic survey was distributed to obstetrical providers, including Ob/Gyns, midwives, and medical students. For statistical analysis, logistic regression was used.

**Results**: 210 responses were recorded from 37 states. The majority of responses were from providers in Michigan (20%, n = 42), in an urban setting (49.5%, n = 104), and practicing as Ob/Gyn physicians (81.9%, n = 172) or Maternal Fetal Medicine physicians (14.8%, n = 31). For a low risk prenatal patient, providers reported screening for a wide range of genes (1‐445 genes, Fig. 1). Most reported screening for 14 genes (n = 49, 23.3%), followed by 4 genes (n = 33, 15.7%). A minority of providers deviate from this practice, with some (4.6%) basing the gene number on patient preferences, while others (5.7%) routinely screen for >100 genes; furthermore, 12.9% of providers were unsure of the number screened (Fig. 1). Overall, 62.4% (n = 131) of respondents support expanding ACOG recommendations for routine GCS for low risk PNC. Increased provider comfort level for counseling patients on GCS results was significantly associated with increased support for EGCS (p = 0.02) (Fig. 2). Perceived barriers to integrating EGCS include concerns about patient cost, a lack of appropriate pre and post‐test counseling, and clinic logistics.

**Conclusion**: There is significant heterogeneity in current GCS practices among obstetrical providers. The majority of respondents support an updated ACOG practice guideline for EGCS. Given the rapidly changing landscape of genetic technology and reproductive choice, increased provider education is paramount to overcome potential barriers to implementing EGCS and achieving equitable PNC.

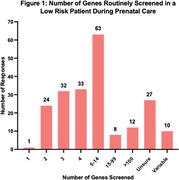

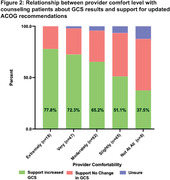

## 1063 | Risk Of Severe Maternal Morbidity and Mortality Among Pregnant Patients with Chronic Kidney Disease

Joyce H. Xu^1^; Isabella Toledo^2^; Emily A. DeFranco^3^; Carri Warshak^1^; Heather N. Czarny^1^; Robert M. Rossi^1^

*^1^University of Cincinnati College of Medicine, Cincinnati, OH; ^2^Indiana University School of Medicine, Indianapolis, IN; ^3^Department of Obstetrics and Gynecology, University of Kentucky, Lexington, KY*

4:00 PM ‐ 6:00 PM

**Objective**: Chronic kidney disease (CKD) is a significant cause of adverse obstetric outcomes, yet few studies assess the risk of severe maternal morbidity (SMM) among those with CKD. We evaluated the prevalence and association of CKD with SMM.

**Study Design**: This was a population‐based, retrospective cohort study of U.S. delivery hospitalizations from 2010‐2020 utilizing the National Inpatient Sample database. Patients were identified as having a delivery hospitalization, CKD, and SMM using International Classification Diagnoses codes (9th and 10th edition). The primary outcome of SMM was defined by the Centers for Disease Control and Prevention criteria. Multivariate logistic regression analyses were used to estimate adjusted relative risks (aRR) and 95% confidence intervals (CI). Subgroup analyses were performed by CKD subtype, stage, race and ethnicity, and individual SMM indicators.

**Results**: Among 38,374,326 parturients, 77,210 (0.2%) had CKD. CKD was associated with an increased risk of SMM (aRR 7.2, 95% CI 6.7‐7.6) and maternal death (aRR 4.6, 95% CI 3.2‐6.4). Increased risk of SMM was associated with all CKD subtypes, stages, and a history of renal transplant. Those with CKD were at the highest risk for acute renal failure (aRR 24.4, 95% CI 22.2‐26.8) and sepsis (aRR 9.3, 95% CI 7.9‐11.0) (Table 1). Black individuals had a higher adjusted population attributable fraction between SMM and CKD (3.8%, 95% CI 3.5‐4.1). Maternal death was significantly associated with diabetic nephropathy, renovascular, and obstructive or unspecified CKD (aRR 7.3‐14.1), as well as CKD stages 3‐5 (aRR 15.5‐32.6). The risk of SMM and maternal death was similar in those with a history of renal transplant and those with stage 1 CKD (Figure 1). The number needed to treat with renal transplant to prevent one SMM event or maternal death in those with stages 3‐5 CKD was 2.6 (95% CI 2.4‐2.9) and 45.0 (95% CI 31.0‐82.0), respectively.

**Conclusion**: CKD in pregnancy was significantly associated with SMM, mortality, and other adverse perinatal outcomes, warranting close surveillance and multidisciplinary management throughout pregnancy.

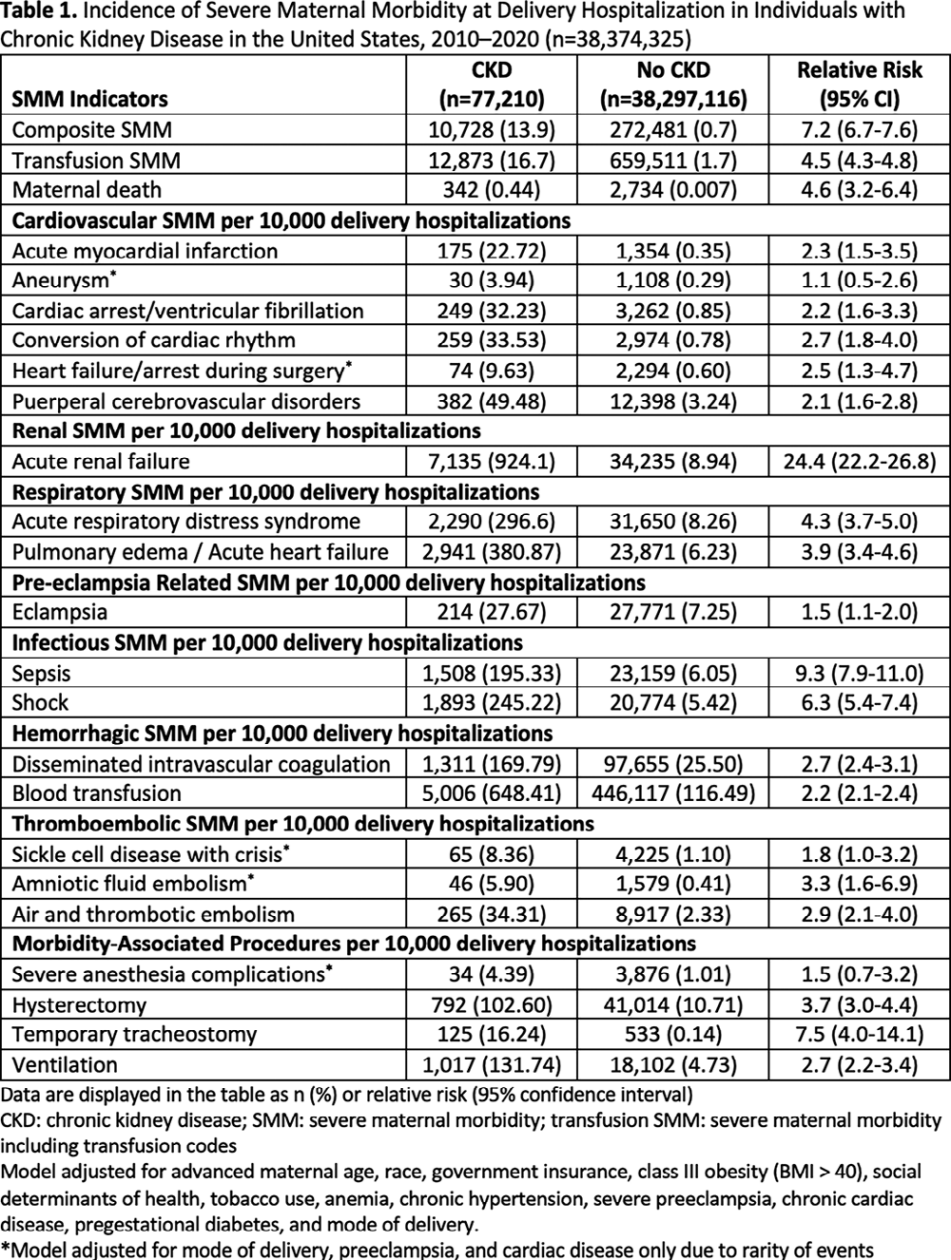

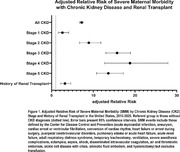

## 1064 | Effect of Intraoperative Sublingual Nitroglycerin on Active Segment Uterine Incision Rate in Preterm Cesarean Delivery

Judy Jiang^1^; Alexandra L. Berra^2^; Megan Breen^3^; Brittany Butler^4^; Anna Rybinska‐Campbell^5^; Angela Ranzini^6^

*^1^Case Western Reserve University/Metrohealth, Cleveland, OH; ^2^Case Western Reserve University Metrohealth Medical Center, Cleveland, OH; ^3^Ohio State University, OH; ^4^Saint Peter's University Hospital, Saint Peter's University Hospital, NJ; ^5^Metrohealth, Cleveland, OH; ^6^The MetroHealth System, Cleveland, OH*

4:00 PM ‐ 6:00 PM

**Objective**: Active segment uterine incisions, including classical, T‐shaped and high transverse incisions are often performed during preterm cesarean deliveries (CD). These uterine incisions increase the risk of maternal morbidity with longer operative time, increased blood loss, and rate of uterine dehiscence in future pregnancies compared to lower segment uterine incisions. Patients with uterine incisions in the active segment are also not candidates for future trials of labor. Nitroglycerin is known to cause uterine relaxation and has been used in cases of uterine inversion and Bandl's rings. Our primary aim was to determine if intraoperative sublingual nitroglycerin is associated with decreased active segment uterine incision rate in patients undergoing CD prior to 30 weeks of gestation. Secondary outcomes included maternal blood loss, umbilical artery pH, and five minute APGAR scores.

**Study Design**: In this case control study, patients with singleton pregnancy between 22 weeks 0 days and 29 weeks 6 days of gestation undergoing CD at two community‐based hospitals were compared between the years of 2017 and 2023. Maternal and neonatal variables were collected and analyzed using equality of means, Mann‐Whitney U tests as appropriate.

**Results**: Patients who received nitroglycerin (n = 26) were compared with patients at the same week of gestation who did not receive nitroglycerin (n = 26) during cesarean delivery. The rate of active segment uterine incision was lower in the group that received nitroglycerin intraoperatively (26.9%) compared to the group that did not (61.5% p = 0.012). Quantitative blood loss (QBL) was significantly decreased in patients who received nitroglycerin with a mean QBL of 583mL compared to 794mL in patients who did not (p = 0.002). Neonatal outcomes including umbilical artery pH and APGAR scores were not found to be significantly different between the two groups.

**Conclusion**: Intraoperative nitroglycerin administration was associated with a lower rate of active segment uterine incision in patients undergoing CD and decreased maternal blood loss. Neonatal outcomes were similar between groups.

## 1065 | Factors Associated with Exclusive Formula Feeding in Individuals with History of Diabetes in Pregnancy

Julia Sroda Agudogo^1^; Krysten North^2^; Chloe Zera^1^; Stephen Wagner^1^; Anna M. Modest^1^

*^1^Beth Israel Deaconess Medical Center, Boston, MA; ^2^Brigham and Women's Hospital, Boston, MA*

4:00 PM ‐ 6:00 PM

**Objective**: Infants of those with diabetes are less likely to be breastfed despite unique benefits in this population. Knowledge of factors associated with exclusive formula feeding (EFF) would enable identification of patients at high‐risk of lactation difficulties. Our objective was to investigate factors associated with EFF in those with pregestational diabetes (PGDM) or gestational diabetes (GDM).

**Study Design**: The United States Vital Statistics Birth Certificate data was utilized in this retrospective cohort study. Singleton pregnancies with PGDM or GDM from 2016‐2022 were included. Pregnancies with missing infant feeding and diabetes data were excluded. We evaluated EFF risk at hospital discharge across multiple maternal and neonatal characteristics and morbidity within both groups. Composite maternal morbidity included admission to ICU, blood transfusion, clinical chorioamnionitis/fever, uterine rupture, unplanned hysterectomy, and perineal laceration. Composite neonatal morbidity included admission to NICU, APGAR score < 5 at 5 min, assisted ventilation > 6 hours and neonatal seizure.

**Results**: Of the 1,796,752 births included, 227,948 (12.7%) had PGDM and 1,568,804 (87.3%) had GDM. Demographic factors associated with EFF included racial minorities especially non‐Hispanic Black, education less than college level, single marital status, lack of prenatal care, smoking in pregnancy, underweight, overweight and obesity within both groups (Table 1). EFF was more likely after caesarean delivery and operative vaginal delivery compared to vaginal delivery within both groups (Table 2). Composite maternal morbidity, ICU admission and transfusion were associated with increased EFF, while clinical chorioamnionitis/fever was not associated with increased EFF within both groups. EFF was more likely within the setting of composite neonatal morbidity or preterm delivery within both groups (Table 2).

**Conclusion**: This study demonstrates that several maternal and neonatal risk factors are associated with EFF among those with PGDM or GDM. There is a need for enhanced breastfeeding support in this vulnerable population.

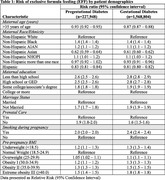

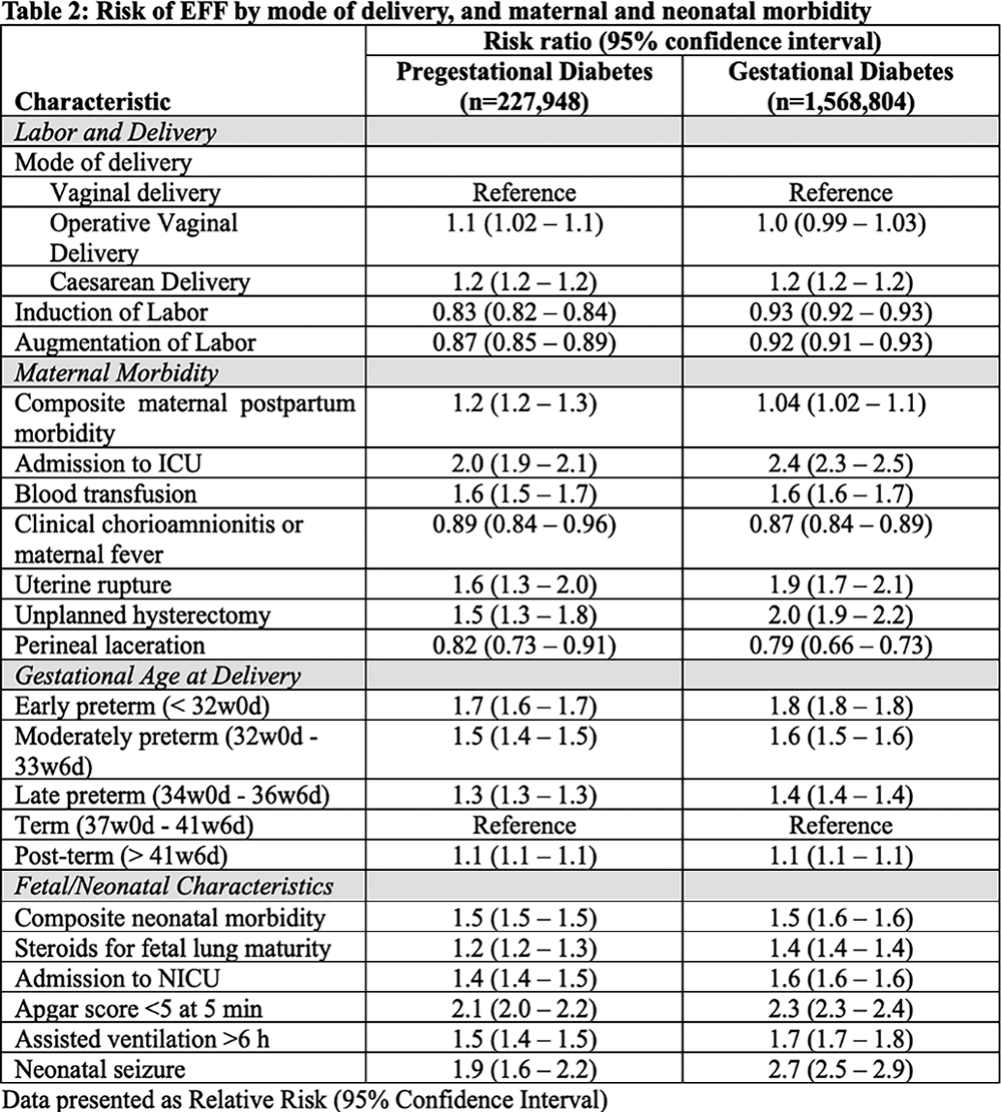

## 1066 | First Trimester Combined Screening for Preterm Preeclampsia using the Fetal Medicine Foundation Competing Risks Model

Juliana Martins^1^; Elizabeth Miller^2^; Michael Bittner^2^; Daniel L. Rolnik^3^; Tetsuya Kawakita^4^

*^1^Macon & Joan Brock Virginia Health Sciences Eastern Virginia Medial School at Old Dominion University, Virginia Beach, VA; ^2^Macon & Joan Brock Virginia Health Sciences Eastern Virginia Medial School at Old Dominion University, Norfolk, VA; ^3^Monash University, Melbourne, South Australia; ^4^Macon & Joan Brock Virginia Health Sciences Eastern Virginia Medical School at Old Dominion University, Norfolk, VA*

4:00 PM ‐ 6:00 PM

**Objective**: To assess the performance of first trimester combined screening using the Fetal Medicine Foundation (FMF) model for the prediction of preterm preeclampsia in a U.S. population.

**Study Design**: This was a secondary analysis of the Nulliparous Pregnancy Outcomes Study: Monitoring Mothers‐to‐be database. Diagnoses of chromosomal or structural fetal abnormality, miscarriage, or fetal death before 24 weeks of gestation, and those missing information regarding gestational age at delivery or preeclampsia were excluded. Variables in the FMF risk calculation included maternal factors, mean arterial pressure, biochemical markers, and uterine artery pulsatility index. We assessed the model performance using the area under the curve (AUC) of the receiver operating characteristic (ROC) curve using a bootstrap method. The optimal cutoff point was determined using Liu's method and calculated sensitivity, specificity, positive and negative predictive values (PPV, NPV), positive and negative likelihood ratio (LHR), and odds ratio (OR). Goodness of fit was evaluated by calibration plot.

**Results**: The study analyzed 8,675 nulliparous women, among whom 2.18% had preterm preeclampsia. The ROC curve (Figure 1) revealed an AUC of 0.75, with a 95% confidence interval (CI) of 0.71 to 0.78 at the optimal cutoff point of 0.7%. Sensitivity of the model was 64.0% (95% CI 56.7–70.9), and specificity was 72.5% (95% CI 71.5–73.4). The PPV was 4.9% (95% CI 4.1–5.9), whereas NPV was 98.9% (95% CI 98.6–99.1). The positive LHR was 2.3 (95% CI 2.1–2.6), and the negative LHR was 0.5 (95% CI 0.4–0.6). OR for predicting preterm preeclampsia was 4.7 (95% CI 3.5–6.3). Overall, the FMF model underestimated the risk of preterm preeclampsia as shown by calibration plot (Figure 2).

**Conclusion**: The FMF combined screening algorithm demonstrates a moderate ability to predict preterm preeclampsia in a U.S. nulliparous population, indicating a fair discriminative power. Ongoing prospective studies aim to validate this model across diverse populations, potentially leading to more tailored and effective pre‐eclampsia prediction.

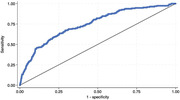

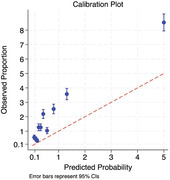

## 1067 | Association of CDC Social Vulnerability Index and Adequate Prenatal Care

Julie Robin Dean^1^; Anna P. Staniczenko^1^; Kristin M. Voegtline^2^; Charlene Thomas^2^; Steven Yen^2^; Evan Sholle^2^; Lauren M. Osborne^2^; Heather S. Lipkind^2^; Moeun Son^2^

*^1^Weill Cornell Medical College, New York, NY; ^2^Weill Cornell Medicine, New York, NY*

4:00 PM ‐ 6:00 PM

**Objective**: Disparities in prenatal care (PNC) access are a major public health issue. We aimed to evaluate the association between the CDC Social Vulnerability Index (SVI) and receipt of adequate prenatal care.

**Study Design**: This was a retrospective case control study of patients delivered at 3 hospitals within a New York City health system from 1/1/22 to 5/31/24 who had at least 1 completed PNC visit at < 20 weeks’ gestation at an affiliated clinic. Data were extracted from the electronic medical record. Outcomes were based on the distribution of the number of outpatient PNC visit encounters among the cohort: inadequate PNC was defined as <8 visits (<25^th^ percentile) and adequate PNC was defined as >8 visits. The exposure of interest was CDC‐defined SVI, which was calculated using a validated technique for geocoding patient addresses: FIPS codes were generated for the census tract and block group of each patient's home address to generate overall SVI and SVI theme scores. SVI scores were converted into categorical variables by quartiles signifying low to high social vulnerability. Other sociodemographic, medical, and obstetric factors were examined. Univariate and multivariate logistic regression models were performed.

**Results**: Compared to those with adequate PNC, those with inadequate PNC were more often Black, Hispanic, non‐married, and < 21 years old. Among the SVI quartiles, high vulnerability SVI had the greatest number of patients with inadequate PNC (40.0%). Compared to those with low SVI, patients with mid‐high and high SVI had the lowest odds of adequate PNC (respectively: OR = 0.84, 95% CI 0.73, 0.96; OR = 0.67, 95% CI 0.58, 0.77). Of the SVI themes, socioeconomic status and household composition had the strongest associations with inadequate PNC.

**Conclusion**: Social vulnerability is associated with adequacy of prenatal care. The CDC's SVI may be a useful tool to identify patients at risk of receiving inadequate prenatal care and linking them to appropriate resources.

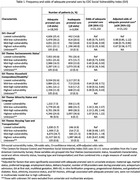

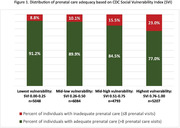

## 1068 | Maternal Serum Leptin Level in Early‐and mid‐Gestation as a Possible Marker of Fetal Adiposity

Junko Tamai^1^; Satoru Ikenoue^1^; Keisuke Akita^1^; Naotsugu Ishikawa^1^; Yasuhiko Ogata^1^; Kaoru Kajikawa^1^; Yuka Fukuma^1^; Yuya Tanaka^1^; Toshimitsu Otani^2^; Yoshifumi Kasuga^1^; Mamoru Tanaka^1^

*^1^Keio University School of Medicine, Shinjuku‐ku, Tokyo; ^2^Keio University School of Medicine, Nakano‐ku, Tokyo*

4:00 PM ‐ 6:00 PM

**Objective**: Newborns exhibit substantial variation in fat mass accretion, which extend into infancy and metabolic dysregulation in later life. Leptin is one of the adipokines related with lipid metabolism, which affects birth weight and infant adiposity. However, the association between maternal serum leptin and fetal fat deposition remains unclear. This study aimed to investigate the association of maternal serum leptin in early‐ and mid‐gestation with fetal adiposity across gestation.

**Study Design**: A prospective cohort study was conducted in 95 singleton uncomplicated pregnancies. Maternal blood sample was obtained at 10 and 24 weeks’ gestation. Serum leptin level was log transformed to be normally distributed. Fetal ultrasonography was performed at 24, 30 and 36 weeks’ gestation. Estimated fetal adiposity (EFA) was calculated as the average of standardized z scores of arm percentage fat area, thigh percentage fat area and anterior abdominal wall thickness as previously reported. The association between maternal serum leptin and EFA was examined using Pearson product moment correlation. Multiple linear regression was conducted to quantify the association between maternal serum leptin and EFA with adjustment for potential confounding factors including maternal age, parity, pre‐pregnancy body mass index, gestational weight gain, and infant sex.

**Results**: Maternal serum leptin was 20.0 ± 11.9 (mean ± S.D.) and 22.8 ± 14.6 ng/ml at 10 and 24 weeks, respectively. Maternal leptin levels were not associated with EFA at 24 and 30 weeks. Maternal leptin at 10 weeks (r = 0.298, p = 0.005) and 24 weeks (r = 0.361, p < 0.001) were associated with EFA at 36 weeks. After controlling for the covariates, leptin at 10 weeks (standardized B = 0.317, p = 0.028) and 24 weeks (standardized B = 0.413, p = 0.004) significantly correlated with EFA at 36 weeks.

**Conclusion**: Maternal serum leptin level in early‐ and mid‐gestation is associated with fetal fat deposition in late gestation. Maternal serum leptin could be a marker for identifying increased adiposity during intrauterine life.

## 1069 | Association of Cord Blood Cytokine Levels with Fetal Adiposity Across Gestation

Junko Tamai^1^; Satoru Ikenoue^1^; Keisuke Akita^1^; Naotsugu Ishikawa^1^; Yasuhiko Ogata^1^; Kaoru Kajikawa^1^; Yuka Fukuma^1^; Yuya Tanaka^1^; Toshimitsu Otani^2^; Yoshifumi Kasuga^1^; Mamoru Tanaka^1^

*^1^Keio University School of Medicine, Shinjuku‐ku, Tokyo; ^2^Keio University School of Medicine, Nakano‐ku, Tokyo*

4:00 PM ‐ 6:00 PM

**Objective**: Recent clinical and experimental evidence have shown that the origins of obesity (i.e., increased adiposity) can be, in part, traced back to intrauterine life. However, the determinants of fetal fat deposition have yet to be elucidated. This study aimed to investigate the association between cord blood cytokines related with lipid metabolism (leptin, adiponectin, insulin‐like growth factor‐1 [IGF‐1]) and fetal adiposity across gestation.

**Study Design**: A prospective study was conducted in a cohort of 91 singleton pregnancies. Fetal 3D ultrasonography was performed at 24, 30, and 36 weeks’ gestation. Estimated fetal adiposity (EFA) was calculated by integrating measurements of cross‐sectional arm and thigh percentage fat area and anterior abdominal wall thickness as previously reported. Serum cytokine levels and C‐peptide immunoreactivity (CPR) of the umbilical cord (as a proxy for fetal insulin resistance) were evaluated in the cord blood sample obtained at delivery. The associations between cord blood cytokine levels and EFA at 24, 30 and 36 weeks were determined by multiple linear regression adjusted for potential covariates including maternal age, parity, pre‐pregnancy BMI, gestational weight gain, fetal sex, and gestational age at assessments.

**Results**: Serum leptin, adiponectin and IGF‐1 of the umbilical cord was 8.5 ± 6.4 ng/ml (mean ± S.D.), 23.0 ± 6.7 µg/ml and 52.1 ± 19.0 ng/ml, respectively. After adjusting for the covariates, leptin was not associated with EFA at 24 and 30 weeks, but significantly correlated with EFA at 36 weeks (standardized B = 0.299, p = 0.008). Leptin was also positively correlated with CPR in the umbilical cord (p = 0.024). Cord adiponectin and IGF‐1 were not associated with EFA across gestation.

**Conclusion**: Cord blood leptin level was associated with fetal adiposity in late gestation. Considering the effect of leptin on fetal insulin resistance and lipid metabolism, increased leptin level potentially be one of the serum biomarkers of increased fetal adiposity, which leads to infant obesity and metabolic dysfunction in later life.

## 1070 | Non‐Mother Birthing Identities and Risk of Hospitalization for Psychiatric Diagnoses Before and After Delivery Hospitalizations

Justin S. Brandt^1^; Rebecca J. Baer^2^; Scott P. Oltman^2^; Diana S. Abbas^3^; Marra Ackerman^1^; Allison Deutch^1^; Dana R. Gossett^1^; Laura L. Jelliffe‐Pawlowski^1^

*^1^NYU Langone Health, New York, NY; ^2^University of California San Francisco School of Medicine, San Francisco, CA; ^3^NYU Grossman School of Medicine, New York, NY*

4:00 PM ‐ 6:00 PM

**Objective**: To understand the impact of pregnancy on depression and other psychiatric illnesses for transgender and gender diverse people, we evaluated the risk of hospitalization for psychiatric diagnoses among non‐mother birthing people in the year before and after delivery hospitalizations.

**Study Design**: We performed a cross‐sectional study of singleton live births in California (2019‐2021). Birth certificates were linked to hospital discharge records for birthing people for the year before and after delivery hospitalizations. Birthing parent identity was based on birth certificates, in which birthing people self‐identify as mother, father, or parent. The primary outcome was hospital admission with psychiatric diagnoses, as determined by ICD‐10 codes. Risk of hospitalization with psychiatric diagnoses was calculated for non‐mother versus mother birthing people in the year before or after delivery hospitalization using Poisson regression models.

**Results**: There were 1,298,307 singleton live births in California from 2019‐2021, including 1,065,714 hospital records linked with birthing person identities. 898 (0.08%) people had non‐mother birthing identities (n = 515 fathers, n = 383 parents), of whom 143 (15.9%) had psychiatric diagnoses compared to 127,545 (12.0%) birthing mothers. In the year prior to delivery hospitalization, 41 (4.6%) non‐mother birthing people were hospitalized with psychiatric diagnoses compared to 30 (3.3%) non‐mother birthing people in the year postpartum. Compared to mother birthing people, non‐mother birthing people were at increased risk for these hospitalizations in the year before (RR 1.22, 95% CI 0.90, 1.66) and after (RR 1.52, 95% CI 1.06, 2.17) delivery hospitalizations. Characterization of medical engagement is described in the Table.

**Conclusion**: In this study, non‐mother birthing people were at increased risk for hospitalization with psychiatric diagnoses in the year after delivery hospitalizations, but the absolute number of postpartum hospitalizations with psychiatric diagnoses was lower. Further study is needed to understand the impact of pregnancy on this risk.

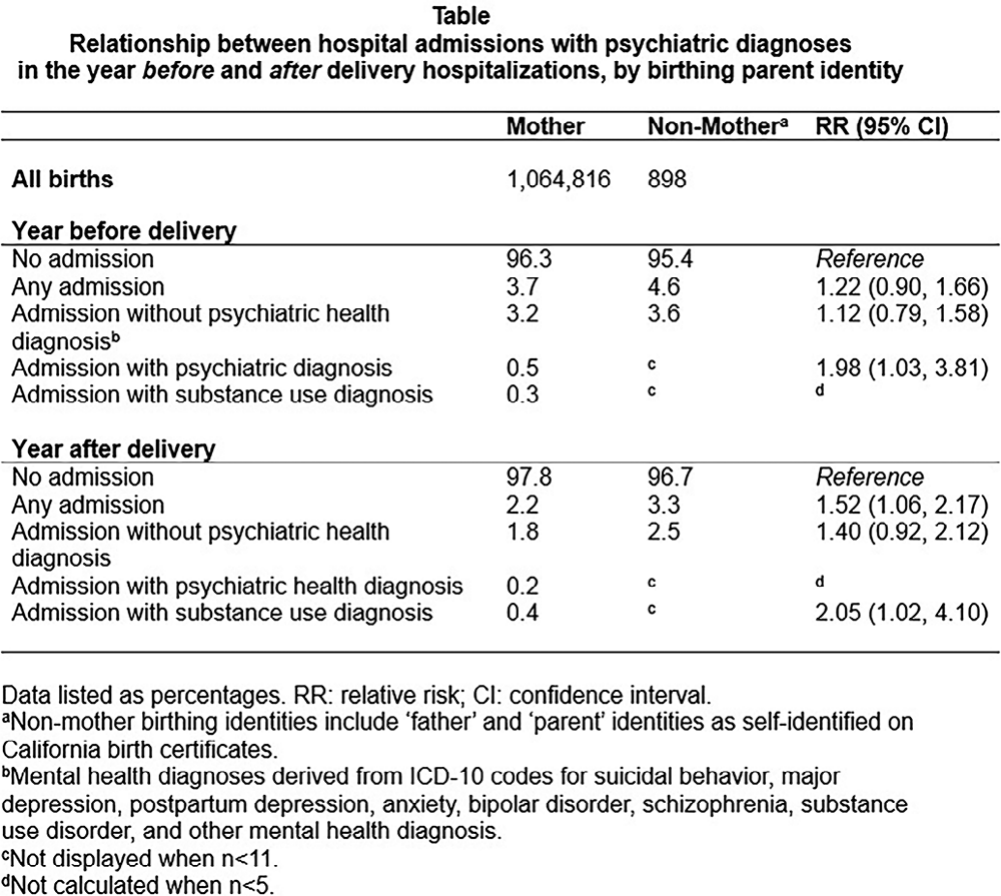

## 1071 | Non‐Mother Birthing People and the Risk of Ischemic Placental Disease, Severe Morbidity, and Preterm Birth

Justin S. Brandt^1^; Rebecca J. Baer^2^; Scott P. Oltman^2^; Diana S. Abbas^3^; Marra Ackerman^1^; Allison Deutch^1^; Dana R. Gossett^1^; Laura L. Jelliffe‐Pawlowski^1^

*^1^NYU Langone Health, New York, NY; ^2^University of California San Francisco School of Medicine, San Francisco, CA; ^3^NYU Grossman School of Medicine, New York, NY*

4:00 PM ‐ 6:00 PM

**Objective**: To evaluate the risk of ischemic placental disease (IPD), severe morbidity (SM), and preterm delivery (PTD) among individuals who identify as non‐mother birthing people, presumably transgender men and gender diverse people assigned female sex at birth.

**Study Design**: We performed a cross‐sectional study of singleton live births in California (2019‐2021). Birth certificates were linked to hospital discharge and neonatal records. Birthing parent identity was based on birth certificates, in which birthing people self‐identify as mother, father, or parent. The primary outcomes were IPD (hypertensive disorders of pregnancy, placental abruption, and small for gestational age birth), composite SM based on the CDC definition, and PTD < 37 weeks gestation. Outcomes were determined by ICD‐10 codes. The risk of outcomes was calculated for non‐mother versus mother birthing people using Poisson regression models.

**Results**: There were 1,298,307 singleton live births in California from 2019‐2021, including 1,065,714 linked hospital and neonatal records with birthing person identities. 898 (0.08%) people had non‐mother birthing identities, of whom 9.2% were age >40 years, 24.9% had BMI >30 kg/m^2^, 13.1% had pregestational diabetes, and 2.2% had chronic hypertension. Compared to mother birthing people, non‐mother birthing people had similar rates of adequate prenatal care as defined by Kotelchuck (71.9% vs. 70.1%), but higher rates of cesarean delivery (30.9% vs. 24.9%) and pregnancies conceived with assisted reproduction (17.3% vs. 1.7%). The risk of IPD was similar between the groups, but the risks of SM (RR 1.69, 95% CI 1.20, 2.38) and preterm birth (RR 1.30, 95% CI 1.07, 1.58) were increased among non‐mother birthing people. The risks are further described in the Table.

**Conclusion**: In this study of singleton live births in California, non‐mother birthing people had similar rates of adequate prenatal care, but were at increased risk for SM and PTD, though not at increased risk for IPD, compared to mother birthing people. These disparate risks may reflect the impact of minority stress and warrant further evaluation.

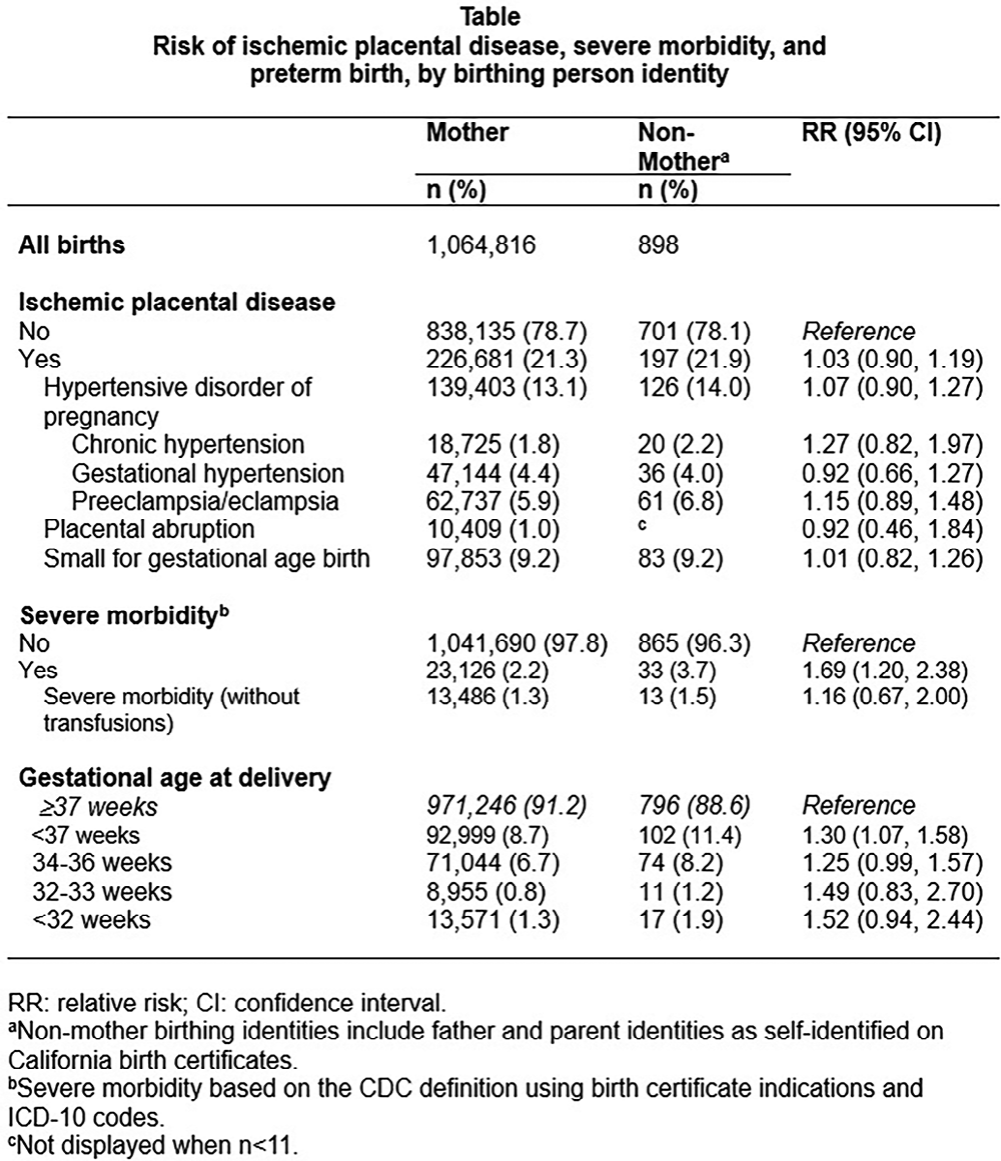

## 1072 | Respectful Maternity Care in Ukraine During a Time of War

Volodymyr V. Artyomenko^1^; Annekathryn Goodman^2^; Igor Shpak^3^; Mary Greenwald^4^; Marcella Regina Cardoso^4^; Katherine D. Fachon^4^; Christina M. Duzyj^2^

*^1^Odesa National Medical University, Odesa, Odes'ka Oblast'; ^2^Massachusetts General Hospital, Boston, MA; ^3^Maternity Hospital No 5, Odesa, Odes'ka Oblast'; ^4^Strength and Serenity Initiative Against Gender‐Based Violence, Massachusetts General Hospital, Boston, MA*

4:00 PM ‐ 6:00 PM

**Objective**: Respectful maternity care (RMC) is a fundamental right of birthing people. Our objectives were: to measure the proportion of obstetric providers at an Odesa Maternity Hospital reporting disrespect and abuse (D&A) during childbirth; to identify challenges to RMC; and to assess perceptions of the impact of war on RMC.

**Study Design**: This is a cross‐sectional study consisting of a quantitative survey distributed to physicians, nurses, midwives, and other personnel at Odesa City Maternity Hospital № 5. The survey included 90 questions assessing prevalence and types of disrespect. Topics covered included: RMC practices performed; disrespectful behaviors witnessed; the impact of war on the provision of RMC; Perceived Stress Scale and Post‐Traumatic Stress Scale; perspectives on patient autonomy and decision‐making. Results were compared with a survey administered in a Boston maternity unit.

**Results**: 202 Providers responded: 89 nurses and junior nurses, 28 midwives, 84 physicians, and 1 unknown provider. 49.5% were familiar with the term “disrespect and abuse,” compared with 82.6% in Boston; and 50.0% believed D&A exists in the obstetrics field, compared with 84.8% in Boston. The most reported types of disrespect were scolding or blaming (58.4% compared with 63% in Boston), asking private questions in the presence of others (42.1% vs 58.7%) and uncomfortable vaginal examinations (39.6% vs. 65.2%). The most common types of discriminatory care witnessed were discrimination based on social status (38.6% vs. 28.3%) and ability to pay “pocket money” (37.1%).

**Conclusion**: This study provides insights into the provision of maternity care under conditions of duress, and highlights challenges faced under the strains of conflict. These efforts aim to identify gaps in education and training around RMC in order to enhance maternal healthcare delivery in conflict‐affected regions, fostering a more supportive and respectful environment for providers and patients.

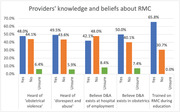

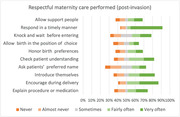

## 1073 | Short‐Interval Births Among Adolescents who Received Immediate Postpartum Long‐Acting Reversible Contraception

Katherine B. Kaak^1^; Alicia Mastronardi^2^; Jill M. Maples^3^; Kimberly B. Fortner^4^; Lilyan Starkey^1^; William L. Riley^4^; Rebecca Purvis^5^; Megan L. Young^6^; Nikki B. Zite^4^

*^1^University of Tennessee at Knoxville, Knoxville, TN; ^2^University of Tennessee Graduate School of Medicine, Department of OB/Gyn, Knoxville, TN; ^3^University of Tennessee Health Science Center, College of Medicine, Knoxville, TN; ^4^University of Tennessee Graduate School of Medicine, Knoxville, TN; ^5^University of Tennessee Medical Center Knoxville, Knoxville, TN; ^6^University of Tennessee Medical Center, Center for Women and Infants, Knoxville, TN*

4:00 PM ‐ 6:00 PM

**Objective**: Adolescent pregnancy is linked to adverse maternal and neonatal outcomes and is associated with short interval birth, further exacerbating these risks. Contraceptive counseling and access can decrease rates of short‐interval birth, especially in this high‐risk population. Tennessee faces high rates of both adolescent pregnancies and SIB, with 32.5% of pregnancies following a prior live birth having <18 months of birth spacing (2018‐2020 average). In 2018, a statewide quality improvement project introduced immediate postpartum long‐acting reversible contraception (IPP LARC) for publicly insured recipients. This study aims to assess SIB rates among adolescents who received IPP LARC.

**Study Design**: This retrospective study utilized billing data for publicly insured adolescents (≤20 years old) giving birth from January 2018‐December 2019. These data were matched by SSN, date of birth, and mother's name with statewide birth certificate records from January 2015‐December 2021. SIB was defined as two births ≤24 months apart. Among multiparous patients, SIB prior to and after receiving IPP LARC was assessed.

**Results**: We identified 267 adolescent deliveries. IPP arm implants were inserted more often than intrauterine devices (IUDs) (65.5% vs 34.5%) (Figure1) in these adolescents. Of the 267, 62.0% (n = 165) were primiparous (Table 1). Among multiparous patients (n = 101), 56.4% (n = 57) had a SIB prior to IPP LARC placement. The median number of previous live births among multiparous was 1.0 (IQR:1,2). Among the multiparous patients with a prior SIB, 90.7% (49) did not have another SIB post LARC. Among the entire cohort, 8.2% (n = 22) of deliveries had a SIB after IPP LARC placement.

**Conclusion**: Adolescents electing IPP LARC had low subsequent SIB rates. Addressing adolescent pregnancy is a public health priority, and, although secondary prevention, access to desired IPP LARC is a strategy to decrease repeat adolescent pregnancies and short interval births.

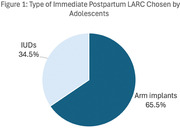

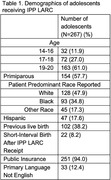

## 1074 | Impact of Socioeconomic Disadvantage on Adolescent Pregnancy

Katherine B. Kaak^1^; Alicia Mastronardi^2^; Jill M. Maples^3^; Destiny Pearson^1^; Zachary J. Shelton^2^; Kimberly B. Fortner^4^; William L. Riley^4^; Rebecca Purvis^5^; William S. Havron, IV^4^; Nikki B. Zite^4^

*^1^University of Tennessee at Knoxville, Knoxville, TN; ^2^University of Tennessee Graduate School of Medicine, Department of OB/Gyn, Knoxville, TN; ^3^University of Tennessee Health Science Center, College of Medicine, Knoxville, TN; ^4^University of Tennessee Graduate School of Medicine, Knoxville, TN; ^5^University of Tennessee Medical Center Knoxville, Knoxville, TN*

4:00 PM ‐ 6:00 PM

**Objective**: Adolescent pregnancy is associated with adverse maternal and neonatal outcomes, less prenatal care, and lower school attendance. In Tennessee, the adolescent birth rate is higher than the national average (23.7/1,000 vs 16.7/1,000). The Area Deprivation Index (ADI), ranging from 1‐100, assesses neighborhood‐level disadvantages, with higher values indicating greater deprivation. This study explores the relationship between adolescent pregnancy, ADI scores, and pregnancy outcomes.

**Study Design**: This retrospective cohort study analyzed adolescent patients (age ≤20) delivering at a regional academic medical center from March 2018‐June 2023. Data was extracted from electronic medical records. Each patient's address was used to determine their Area Deprivation Index (ADI) score, categorized into quintiles representing increasing deprivation (1 to 100), with scores of 61 or higher considered high risk. Chi‐square tests were used to evaluate associations within the dataset.

**Results**: Of 22,086 deliveries, 1,281 (5.8%) were to patients ≤20 years of age. The mean ADI score in this adolescent cohort was 73.4 and 78.8% had ADI 61 or greater (Figure 1). No statistically significant differences were found in patients with higher ADI scores in adequacy of prenatal care obtained, low birth weight full‐term infants, illicit drug use, or hypertensive disorders. Reported marijuana use was significantly lower (p = .007) in patients with higher ADI scores (Table 1). During this time, 60 adolescents had an additional birth while under the age of 20 and 85.0% of those had a high‐risk ADI. Nearly 1 in 4 (23.3%) of the births to adolescent patients were pre‐term.

**Conclusion**: Although nearly 4 of 5 adolescents giving birth at our center were from high‐risk zip codes, risk factors and outcomes did not statistically differ based on ADI score. Further research should look more into these at‐risk teens, focusing on patients with the highest burden of disadvantages.

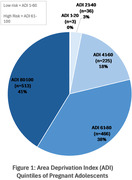

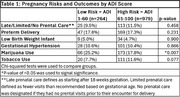

## 1075 | Remote 3D LiDAR Imaging to Estimate Fundal Height: A Feasibility Study

Katherine J. Kissler^1^; Shalom Darmanjian^2^; Quentin Noirhomme^2^

*^1^University of Colorado Anschutz Medical Campus, Aurora, CO; ^2^Bloomlife, San Francisco, CA*

4:00 PM ‐ 6:00 PM

**Objective**: Prenatal care is increasingly offered virtually to expand access to care. However, estimation of fetal growth is limited during virtual appointments jeopardizing safety and quality of care. The purpose of this feasibility study was to evaluate accuracy of term fundal height estimation using 3D LiDAR (Light Detection and Ranging) technology to create accurate 3D images of the gravid abdomen using an Apple iPad. Development of this technology would allow patients to remotely share a 3D image during a virtual visit to allow for accurate physical exam including fundal height estimate.

**Study Design**: In this retrospective feasibility study, we compared fundal height measurements calculated remotely from 3D LiDAR images to those measured in‐person by research nurses using a measuring tape at term in (N = 20) low risk pregnant patients. The 3D LiDAR image was captured on the same day as the physical measurement by the nurse using an Apple iPad with the Polycam app. Participants with incomplete data or inadequate imaging of the fundus and pubis were excluded. Fundal heights were calculated by first mapping fiducial markers along the midline from pubis to the fundus then summing the distances calculated between the 3D surface markers. Fundal heights measured using the 3D LiDAR images were detrended with linear regression and a Bland‐Altman Plot was created to evaluate agreement.

**Results**: Fundal heights measured via 3D LiDAR imaging ranged from 36.68 to 40.21 cm (mean = 38.53) and by the nurses from 35.50 to 44.00 cm (mean = 38.53). The mean absolute error (MAE) before detrending was 1.97±2.44cm. Post detrending MAE was 1.01±1.57cm with the limits of agreement (95% CI, ±3.09cm).

**Conclusion**: 3D LiDAR imaging shows promising preliminary results for accurately measuring fundal heights at term and may provide a clinically useful alternative for estimating fetal growth for patients receiving care remotely. Follow‐up studies are needed to extend these findings to earlier gestations, explore usability of the app during remote visits, and identify additional obstetric applications of 3D abdominal imaging.

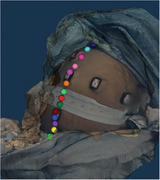

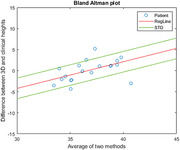

## 1077 | Impact of Prenatal Care on Perinatal Outcomes in Individuals with Opioid Use

Kristin C. Prewitt; Alyssa R. Hersh; Bharti Garg; Ximena A. Levander; Bradley M. Buchheit; Aaron B. Caughey

*Oregon Health & Science University, Portland, OR*

4:00 PM ‐ 6:00 PM

**Objective**: Prenatal opioid use is associated with maternal and neonatal morbidity and mortality, yet only half of individuals are connected to treatment due to barriers accessing care. The objective of this study is to assess how the number of prenatal care attendance affects perinatal outcomes in individuals with opioid use.

**Study Design**: We conducted a retrospective cohort of singleton, non‐anomalous births between 23‐42 weeks gestation using California linked vital statistics and hospital discharge data (2008‐2020). Opioid‐related diagnosis was identified using ICD‐9/ICD‐10 codes. Prenatal visit number grouping (≥5 visits and < 5 visits) was determined based on WHO recommendations for minimum visits to mitigate adverse perinatal outcomes. Chi‐squared and multivariable logistic regression were utilized for statistical analyses.

**Results**: There were 8,799 individuals with opioid use in pregnancy that met inclusion criteria, with the majority attending ≥5 prenatal care visits (80.34%). Attendance at < 5 prenatal care visits was associated with adverse maternal outcomes, including a higher rate of placental abruption (6.2% vs 2.7%; aOR 1.87, 95% CI: 1.42‐2.47), preterm premature rupture of membranes (8.0% vs 3.8%; aOR 1.91, 95% CI: 1.50‐2.43), preterm delivery < 37 weeks (33.0% vs 14.2%; aOR 2.69, 95% CI: 2.34‐3.08), and severe maternal morbidity (6.5% vs 2.8%; aOR 2.31, 95% CI: 1.77‐3.02). Neonatal outcomes were also worse in those with fewer prenatal visits, including higher rates of respiratory distress (4.6% vs 2.6%; aOR 1.78, 95% CI: 1.31‐2.42), neonatal withdrawal (37.6% vs. 29.4%, aOR 1.42, 95% CI: 1.26‐1.61), and NICU admission (58.4% vs 41.9%; aOR 1.76, 95% CI: 1.57‐1.99).

**Conclusion**: We found that lower prenatal care attendance among individuals with an opioid‐related diagnosis is associated with worse maternal and neonatal outcomes. Although our study is limited by the ability to control for pharmacotherapy, addressing barriers to prenatal care among those with opioid use may impact maternal and neonatal morbidity and mortality.

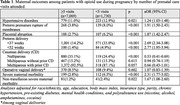

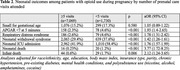

## 1078 | Impact of Changing Medicaid Coverage for Methadone on Opioid Use Disorder Treatment in Pregnancy

Kristin C. Prewitt^1^; Megan Fuerst^1^; Ximena A. Levander^1^; Maria I. Rodriguez^2^

*^1^Oregon Health & Science University, Portland, OR; ^2^Oregon Health & Science Unviersity, Portland, OR*

4:00 PM ‐ 6:00 PM

**Objective**: Driven by synthetic opioids, overdose is the leading cause of maternal mortality. Pregnancy increases difficulty in accessing treatment. The purpose of this study was to assess how Medicaid coverage for methadone affected treatment for opioid use disorder in pregnancy.

**Study Design**: This is a cross‐sectional study using linked South Carolina claims and birth certificate data (2015 to 2022) of live births with a diagnosis of opioid use disorder (OUD) in pregnancy. We divided our population into 2 cohorts: 1) before and 2) after the state's Medicaid approval for methadone coverage on January 1st 2019. Our primary outcome was receipt of medication for OUD (MOUD) during pregnancy or within the first 60 days postpartum. Using standard bi‐variate tests, we compared the pre‐ and post‐policy rates of Methadone of MOUD as well as demographic factors between each group.

**Results**: In total, 2,633 patients with OUD were included in our study. Of these, 1181 delivered prior to Medicaid approval of methadone and 1,452 were in the post‐approval cohort. Following Medicaid approval for methadone, there was a statistically significant increase in the number of methadone prescriptions within the Medicaid population (p< 0.001) and a near significant increase of individuals receiving methadone as MOUD (5.1% vs 9.1%, p = 0.06). There was a statistically significant increase in those receiving any MOUD post‐coverage (34.8% vs 47.2%, p < 0.001). Those who did not receive Methadone or any MOUD were more likely to be under the age of 25 (57.2%) and live in a rural county (59.9%).

**Conclusion**: Our results show that changing Medicaid coverage for methadone increases overall treatment for OUD in pregnancy. However, there continue to be gaps in treatment connections for individuals < 25 years of age and from rural counties. As the opioid epidemic increases, improving access to medication coverage for OUD and focusing efforts on younger, more rural areas may impact OUD treatment in pregnancy, and subsequently, maternal mortality.

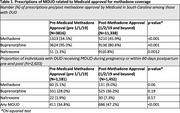

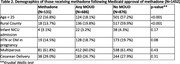

## 1079 | Inequities in Breastfeeding Patterns Among Patients with HDP at a Southwestern Safety net Hospital

Kristin Hinsen^1^; Fiona LaRoc^1^; Christina Yarrington^2^

*^1^School of Medicine Health Sciences Center, University of New Mexico, Albuquerque, NM; ^2^University of New Mexico, Albuquerque, NM*

4:00 PM ‐ 6:00 PM

**Objective**: Breastfeeding reduces lifelong cardiovascular (CV) risk, particularly in those with a hypertensive disorder of pregnancy (HDP). American Indian (AI) patients experience inequities in both preeclampsia and chronic CV disease. This study investigated newborn feeding differences among women with HDP in a cohort with a representative AI population.

**Study Design**: This retrospective cohort study analyzed patients with chronic hypertension or HDP using a quality improvement data set. Cases with uncertain diagnoses underwent direct chart review. The primary outcome was exclusive formula feeding (FF). Demographics included race, ethnicity, insurance type, cesarean delivery (CD), and selected comorbidities. Univariate and multivariable analysis used t‐tests, chi‐square, and logistic regression as appropriate, with a significance level of p< 0.05.

**Results**: Data were analyzed from 975 patients: 198 non‐Hispanic white (NHW), 494 Hispanic (H), 178 AI, and 37 non‐Hispanic Black (NHB). FF was higher (44%) and exclusive breastfeeding (EB) lower (16%) among AI patients compared to other groups (p< 0.001). Univariate analysis revealed increased odds of FF among patients with CD (OR 2.1 [1.6‐2.9]), antihypertensive use (3.5 [2.5‐4.8]), transfusion (2.2 [1.4‐3.5]), and preterm birth (PTB) (13.7 [9.2‐20.4]). AI patients were more likely to have diabetes (2.9 [1.8‐4.7]), PTB (3.3 [2.0‐5.5]), and require antihypertensives (1.8 [1.1‐2.9]). FF rates were high (>60%) in PTB cases across all groups. Multivariable analysis showed 80% increased odds of FF among AI patients compared to NHW, controlling for DM, CD, and HTN medications. Among term deliveries and controlling for the above, AI patients had higher odds of FF (aOR 1.9 [1.2‐3.0]) as well as lower odds of EB (aOR 0.35 [0.21‐0.59]) compared to NHW.

**Conclusion**: These findings demonstrate a higher rate of FF among AI patients compared to NHW, indicating a potential contributor of inequities in subsequent CV disease. Future educational and quality improvement measures should seek to optimize rates of breastfeeding in this high‐risk population.

## 1080 | Racial Disparities in Maternal Mortality before, during, and after the COVID‐19 Pandemic in the US

Kyosuke Kamijo^1^; Lindsay S. Robbins^2^; George R. Saade^2^; Tetsuya Kawakita^2^

*^1^Department of Obstetrics and Gynecology Nagano Prefectural Shinshu Medical Center, Suzaka, Nagano; ^2^Macon & Joan Brock Virginia Health Sciences Eastern Virginia Medical School at Old Dominion University, Norfolk, VA*

4:00 PM ‐ 6:00 PM

**Objective**: To evaluate racial disparities in maternal mortality before, during, and after the Coronavirus Disease 2019 (COVID‐19) pandemic.

**Study Design**: We conducted difference‐in‐differences (DID) analyses using the Centers for Disease Control and Prevention's WONDER database. Data on the number of births and maternal mortality were aggregated into monthly intervals. We compared changes in maternal mortality during pregnancy and up to 1 year after delivery per 100,000 births between White and Black or African Americans before (Jan 2018–Mar 2020), during (Apr 2020–Mar 2022), and after (Apr 2022–Jun 2024) the COVID‐19 pandemic. Predicted incidence of maternal mortality and DID with 95% confidence intervals (95% CI) were calculated using negative binomial regression models with interactions between race and periods.

**Results**: There were 3,694,282 Black individuals and 17,284,929 White individuals who gave birth during that period, with 2,513 (68 per 100,000) and 4,547 (26 per 100,000) death, respectively (**Figure 1**). Mortality per 100,000 from before COVID‐19 to during COVID‐19 increased by 29.4 (95% CI: 19.8‐39.1) for Black and 11.8 (95% CI: 8.2‐15.4) for White individuals, with a DID of 17.6 (95% CI: 7.3‐28.0) in favor of White (Table 1). Mortality per 100,000 from before COVID‐19 to after COVID‐19 increased by 9.5 (95% CI: 3.2‐15.9) for Black and 1.6 (95% CI: ‐0.4‐3.7) for White with a DID of 7.9 (95% CI: 1.2‐14.6), suggesting that Black‐White disparity worsened after COVID‐19 pandemic.

**Conclusion**: The findings highlight that racial disparity in maternal mortality, which disproportionately affects Black individuals, was further exacerbated by the COVID‐19 pandemic. The gap in maternal mortality between Black and White individuals increased post‐pandemic, underscoring the need for targeted interventions to address systemic inequities in maternal healthcare.

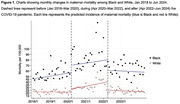

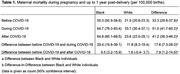

## 1081 | An Increased Postpartum Visit Model vs. the Usual Postpartum Visit Model: A Cost‐Effectiveness Analysis

Lamia K. Hauter^1^; James Cahill^1^; Sarah K. Dzubay^2^; Megha Arora^2^; Ava D. Mandelbaum^2^; Aaron B. Caughey^2^

*^1^Oregon Health & Science University School of Medicine, Portland, OR; ^2^Oregon Health & Science University, Portland, OR*

4:00 PM ‐ 6:00 PM

**Objective**: The postpartum (PP) period is crucial for the health of both mothers and infants, necessitating comprehensive care. Traditional obstetrics care includes a routine postpartum follow‐up visit at perhaps 2 to 6 weeks postpartum, but there is growing interest in exploring alternatives. A recent model which utilized Certified Nurse Midwives (CNMs) to provide more frequent PP visits was found to reduce both visits to the Emergency Department as well as hospital readmissions. The current study models the outcomes, costs, and cost‐effectiveness of more frequent postpartum care versus a traditional obstetric care approach.

**Study Design**: A decision‐analytic model was created using TreeAgePro software to compare outcomes between using the frequent PP visit model versus the standard approach for postpartum follow‐up visits. Our theoretical cohort was 2,356,883, the approximate number of postpartum individuals following low‐risk vaginal deliveries annually in the US. Outcomes were emergency department visits, hospital readmissions, costs, and quality‐adjusted life years (QALYs). All probabilities were derived from literature. The willingness to pay threshold was $100,000/QALY.

**Results**: In our cohort, the higher frequency care model was associated with 68,350 fewer emergency department visits (129,629 vs 197,978) and 683 fewer hospital readmissions (1296 vs 1980) (Table 1). There were $1,487,263,566 of increased costs with the higher frequency of visits model and it increased QALYs by 1217 (by 63,878,707 vs 63,877,490). The increased frequency model was not cost‐effective with an ICER of $1,222,097/QALY. However, the model becomes cost effective when the costs were varied down to 50% or less of baseline assumed cost of 6 postpartum visits.

**Conclusion**: The model of more frequent postpartum visits led to fewer ED visits and hospitalizations, but ultimately was not cost‐effective. Whether the benefits from this high‐frequency visit model would be realized from a virtual visit approach which would reduce costs should be investigated.

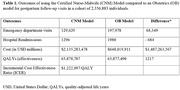

## 1082 | Impact of Patient‐Directed Financial Penalties on Appointment Non‐Adherence Rates in an Academic Maternal‐Fetal Medicine Clinic

Lauren A. Forbes^1^; Matvey Karpov^1^; Peter Takacs^1^; Tetsuya Kawakita^2^

*^1^Eastern Virginia Medical School, Norfolk, VA; ^2^Macon & Joan Brock Virginia Health Sciences Eastern Virginia Medical School at Old Dominion University, Norfolk, VA*

4:00 PM ‐ 6:00 PM

**Objective**: To examine the impact of patient‐directed financial penalties on appointment non‐adherence rates within a single academic institution's maternal‐fetal medicine (MFM) outpatient clinic.

**Study Design**: This retrospective policy effectiveness study included administrative data from all appointments at Eastern Virginia Medical School's MFM outpatient clinics from May 1, 2018 to April 30, 2022. The institution‐wide patient‐directed financial penalty for appointment non‐adherence was implemented on May 1, 2020. Institutional Review Board approval was waived for this study. An interrupted time series analysis (ITS) stratified by patient insurance type was performed after adjusting the model for the effect of COVID‐19.

**Results**: There were 272,335 appointments; 29,452 (10.8%) were not attended. Pre‐implementation mean (standard deviation) per month was 5,586 (466) appointments with11.1% (0.99%) non‐adherence rate. Post‐implementation was 5,762 (429) appointments with 10.6% (1.36%) non‐adherence rate. Publically insured patients had a higher non‐adherence rate (pre‐: 15.60% (1.56%); post‐: 15.46% (2.13%)).

Adjusted ITS models observed a statistically significant immediate (1.47% fewer appointments; p = 0.01) and sustained (0.09% additional appointments; p = 0.02) effect on number of non‐adherent appointments per month.

Stratified by insurance, non‐publically insured patients observed a significant immeidate effect (1.66% fewer appointments; p = 0.02). Publicly insured patients observed a significant sustained effect (0.18% additional appointments; p< 0.01).

**Conclusion**: A clinically significant reduction in appointment non‐adherence was not observed in a single academic center's MFM outpatient clinic after institutional implementation of patient‐directed financial penalties for appointment non‐adherence. Patient‐directed financial penalties for appointment non‐adherence may not be an effective strategy for decreasing appointment non‐adherence rates. The impact may differ based on patient insurance type.

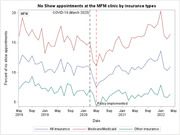

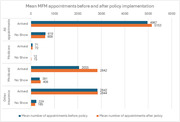

## 1084 | Childhood Sexual Abuse Impacts 2nd Trimester Blood Pressure Nadir

Lauren Holt^1^; Mariana Rocha^1^; Natalie Poliektov^2^; Kaitlyn Stanhope^3^; Vasiliki Michopoulos^1^; Suchitra Chandrasekaran^4^

*^1^Emory University School of Medicine, Atlanta, GA; ^2^Emory University School of Medicine, Department of Gynecology and Obstetrics, Atlanta, GA; ^3^Rollins School of Public Health, Atlanta, GA; ^4^Emory University, Atlanta, GA*

4:00 PM ‐ 6:00 PM

**Objective**: Childhood trauma (CT) impacts health in adulthood with increased risks of hypertensive disease. Relationships between CT & maternal blood pressure (BP) during pregnancy are less clear. Using the Childhood Trauma Questionnaire (CTQ), we sought to assess relationships between CT & BP in each trimester (TRI) & BP nadir in the 2nd TRI.

**Study Design**: We performed a prospective longitudinal cohort study investigating associations between trauma & pregnancy outcomes at an urban safety net hospital. Participants who self‐ identified as Black completed the CTQ, a validated measure of 5 trauma domains in 1st TRI. CT was determined by CTQ total score: ≥35 with CT, < 35 no CT. Systolic (SBP) and diastolic (DBP) pressures were recorded in each TRI. SBP/DBP nadir was calculated (2nd TRI SBP/DBP–1st TRI SBP/DBP) & compared between subjects with & without CT. Continuous variables were compared using Student T‐tests or Rank Sum Tests. Categorical variables were compared using Chi‐Square tests. Multivariable linear regression was performed controlling for age, race, 1st TRI body mass index, & chronic hypertension.

**Results**: Of N = 335, 34.3% & 65.6% had no CT & CT exposure respectively (Table 1). There were no differences in 1st, 2nd or 3rd TRI SBP/DBP or 2nd TRI SBP/DBP nadir between groups (Table 2). CT scores were further subtyped into 3 groups: sexual abuse (SA), physical abuse (PA) and emotional abuse (EA). SA scores were associated with lower 1st TRI SBP (β = ‐0.06, 95% CI ‐0.14‐0.01, p = 0.09). No significant differences in 1st TRI SBP were seen in those with PA or EA. Those with SA had a lower 2nd trimester SBP nadir compared to those without SA (‐1.7±10.5 vs ‐6.3±11.2, p = 0.04).

**Conclusion**: CT is a concerning problem affecting many reproductive aged individuals. Using the detailed CTQ, our data innovatively demonstrates SA in childhood may mitigate vascular relaxation between 1st & 2nd TRI, a time where vascular relaxation is physiologically necessary. Future studies need to evaluate mechanisms driving the relationship between CT subtypes & gestational vascular dysregulation.

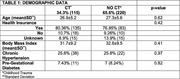

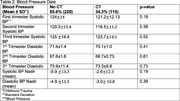

## 1085 | Neighborhood Deprivation as a Predictor of Congenital Syphilis

Le'Nisha Williams^1^; Mariella Gastanaduy^2^; James D. Toppin^3^; Frank B. Williams^1^

*^1^Ochsner Clinic Foundation, New Orleans, LA; ^2^Ochsner Clinic, New Orleans, LA; ^3^Ochsner Health, New Orleans, LA*

4:00 PM ‐ 6:00 PM

**Objective**: In the US, congenital syphilis (CS) has increased 464% since 2001, a trend that has been even more pronounced in Louisiana, a place with both high socioeconomic deprivation at the individual and neighborhood levels. Area Deprivation Index (ADI) is a geographic measure of socioeconomic disadvantage that quantifies neighborhood‐level limitations to material resources. High ADI is associated with a range of adverse health outcomes. We hypothesize that high ADI is a predictor of CS.

**Study Design**: We performed a retrospective cohort study of patients between 18‐45 years old with at least one prenatal visit and delivery within a single large health system in Louisiana between 2015‐2023. Incomplete charts and those without any prenatal syphilis testing were excluded. Patients were stratified as high ADI if their home address census block ranked above the 75^th^ percentile; controls were those below the highest quartile. CS was defined by Centers for Disease Control case criteria. Baseline and pregnancy characteristics were compared using chi square or Wilcoxon Ranked‐Sum test where appropriate. Outcomes were compared by regression analysis controlling for individual level socioeconomic deprivation (indicated by public insurance) generating odds ratio and 95% confidence intervals.

**Results**: Among 56,654 pregnancies meeting inclusion criteria, 17,335 patients (30.5%) resided in high deprivation neighborhoods. High ADI residence was associated with younger age, Black race & public insurance (Table). Among 49 positive CS cases, 37 (75.5%, Table) occurred among patients in high ADI census blocks, corresponding with a congenital syphilis rate of 214 per 100,000 live births compared to 30 per 100,000 live births among non‐high ADI pregnancies (aOR 5.1, 95% CI 2.7–9.8). When analyzed in a continuous fashion, there is a positive correlation between CS and high ADI, with each increasing decile representing an 80% increased risk for CS.

**Conclusion**: High neighborhood deprivation was highly associated with congenital syphilis even when controlling for individual‐level socioeconomic deprivation.

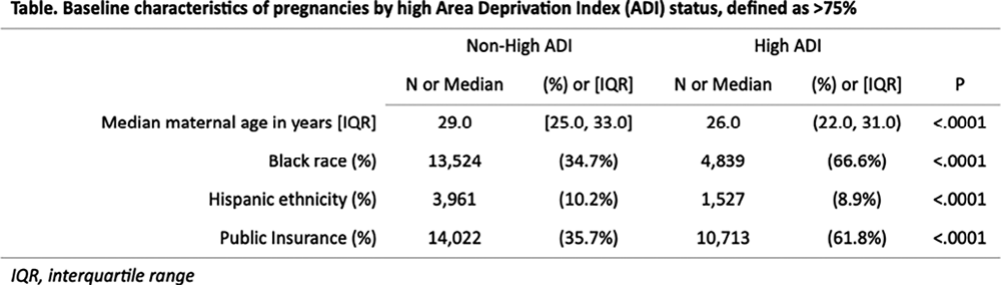

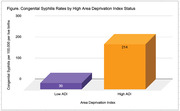

## 1086 | Displaying 1‐year Versus Lifetime Pay‐offs In the Perinatal Decision Analyses

Lihong Mo^1^; Nithya Sivakumar^1^; Herman L. Hedriana^1^; Jeffrey Hoch^2^

*^1^UC Davis Health, Sacramento, CA; ^2^UC Davis, Sacramento, CA*

4:00 PM ‐ 6:00 PM

**Objective**: When the interests of maternal and fetal patients do not align, pregnancy presents a unique challenge to health economic evaluations. The common practice on perinatal topics has been combining all health pay‐offs during the lifetime for both mothers and babies in the equation. However, using lifetime evaluation may not truly reflect the complicated decision‐making considerations in real‐life practice. The objective of this study is to construct a decision tree to compare immediate delivery and expectant management in a hypothetical clinical scenario to compare the preferred decision when 1‐year versus lifetime pay‐offs are used.

**Study Design**: This is a decision analysis in a hypothetical clinical situation when choosing immediate delivery or expectant management is an equipoise. Key assumptions are 1) only one maternal complication is included, its probability is 10% when seeking immediate delivery, while increases to 20% if pregnancy is prolonged for a week, and 25% if prolonged for two weeks; 2) neonatal demise, a short‐term complication (i.e. respiratory distress syndrome), and a long‐term complication (i.e. developmental delay) are considered for neonatal outcomes. The probabilities of neonatal complications are derived from literature. Quality Adjusted Life Week (QALW) was calculated as a product of utility and duration.

**Results**: When maternal complication lasts lifetime, preferred strategies between the 1‐year or lifetime evaluation differ at or before 31 weeks. At and after 32 weeks, both evaluation methods favor immediate delivery due to maternal benefits (Table 1). However, when maternal complication is presumed as short‐term only, preferred strategies between the 1‐year and lifetime evaluation differ almost throughout the span of gestational age.

**Conclusion**: Choosing between immediate delivery versus expectant management is a complicated decision that requires balancing the maternal and neonatal risks and benefits. Our study supports individualized consideration when the risk of maternal complication is high, and the impact is long lasting.

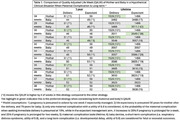

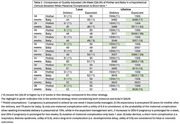

## 1087 | Electrocardiogram Changes Associated with Pregnancy and Pregnancy Trimesters

Lihong Mo^1^; Sonul Gupta^1^; Vivian Pae^1^; Ijeoma Uche^1^; Matthew Ponzini^2^; Machelle Wilson^2^; Philip Strong^1^; Uma Srivasta^1^; Imo Ebong^1^; Herman L. Hedriana^1^; Elaine Waetjen^1^

*^1^UC Davis Health, Sacramento, CA; ^2^University of California, Davis, Sacramento, CA*

4:00 PM ‐ 6:00 PM

**Objective**: Pregnancy is a physiologic high‐volume state. Physiologic electrocardiogram (ECG) changes in pregnancy have been reported but not well characterized. We hypothesize that certain ECG features are more likely to be present in pregnant compared to non‐pregnant individuals, reflecting physiologic cardiac volume and compliance changes. The objective of this study is to evaluate ECG changes in normal pregnancies by trimester compared to women who are not pregnant.

**Study Design**: This is an analysis of a database that includes 22,034 ECGs on 7,298 individual patients from 18‐50 years of age who were pregnant at least once between 2011 to 2024 in a single tertiary medical center. Univariate and multiple generalized linear mixed effect models were performed to evaluate associations between four ECG features (QRS interval, QTc interval, PR interval, and heart rate) in pregnancy and by trimester compared to those in women who were not pregnant or within 6 months postpartum.

**Results**: After excluding patients with pre‐existing cardiac morbidity, or affected hypertensive disorders of pregnancy, 5,574 ECGs from 2,988 patients were included: 3,423 of ECGs (61%) were obtained outside of pregnancy or the 6 months postpartum period, 2,151 (39%) of ECGs during pregnancy or postpartum. Shorter QRS duration, QTc interval, PR interval, and higher heart rate were associated the pregnancy or 2 weeks postpartum state (Table 1). The shorter QRS duration, PR interval, and higher heart rate were associated with the second, third trimester, and two weeks postpartum, and these changes resolved within 6 months postpartum (Table 2).

**Conclusion**: Four ECG features were found to be associate with pregnancy. The association is present particularly in the 2nd and 3rd trimester when cardiovascular changes are most pronounced. Future research includes extracting subtle patterns beyond well characterized ECG features to further investigate associations with normal pregnancy and to evaluate how they can be used to predict pathologic cardiovascular changes associated with pregnancy and postpartum.

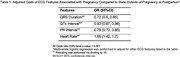

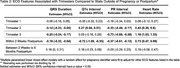

## 1088 | Trends in Uterine Rupture and Perinatal Outcome Among Individuals Undertaking Trial of Labor After Cesarean

Lillian H. Goodman^1^; Amanda A. Allshouse^1^; Torri D. Metz^1^; Ann M. Bruno^2^

*^1^University of Utah, Salt Lake City, UT; ^2^University of Utah Health, Salt Lake City, UT*

4:00 PM ‐ 6:00 PM

**Objective**: ACOG endorses trial of labor after cesarean (TOLAC) as a reasonable alternative to repeat cesarean delivery (CD). Concordantly, national rates of TOLAC have increased but the risk of uterine rupture remains a serious complication. We aimed to evaluate U.S. trends in uterine rupture and perinatal outcomes among individuals undertaking TOLAC from 2010 to 2022.

**Study Design**: This was a population‐level retrospective cohort study using natality files in the National Vital Statistics System (NVSS) from 2010‐2022. We included individuals with singleton, cephalic, term pregnancies undertaking TOLAC with 1 or 2 prior CD. The primary outcome was uterine rupture. Secondary outcomes included unplanned hysterectomy, blood transfusion, maternal or neonatal ICU admission, and neonatal death. Baseline characteristics were compared among those with and without uterine rupture. Temporal trends in outcomes were characterized using JoinPoint regression with average annual percentage change (AAPC) and 95% CI reported. Log binomial modeling estimated changes in maternal outcomes by 1‐year increments adjusting for confounding.

**Results**: Of 1,016,073 deliveries included, uterine rupture occurred in 2,888 (0.28%). Those with uterine rupture were more likely to be older, have gestational diabetes, and be diagnosed with a hypertensive disorder of pregnancy. Rates of uterine rupture, transfusion, and maternal and neonatal ICU admission increased over time, while rates of hysterectomy were not significantly different over time and rates of neonatal death declined (Figure). In adjusted modeling, each 1‐year increment remained significantly associated with higher rates of uterine rupture, transfusion, and admission to ICU but absolute rates remained low (Table).

**Conclusion**: While national rates of uterine rupture increased over time, uterine rupture continues to be rare. Counseling regarding mode of delivery among those with a prior CD should continue to weigh maternal and neonatal outcomes.

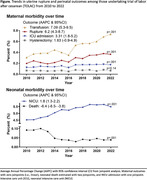

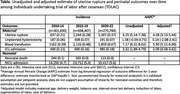

## 1089 | Maternal Cytokine Expression in Mice with Gestational Breast Cancer

Lizelle Comfort^1^; Mackenzie Callaway^2^; Clarissa Bonanno^1^; Christine Metz^3^; Camila dos Santos^2^; Burton Rochelson^1^

*^1^Northwell, New Hyde Park, NY; ^2^Cold Spring Harbor Laboratory, Cold Spring Harbor, NY; ^3^Feinstein Institutes for Medical Research, Manhasset, NY*

4:00 PM ‐ 6:00 PM

**Objective**: Immunosuppressive cytokines are implicated in both cancer progression and prenatal health outcomes. This study aims to examine cytokine levels in the plasma of tumor‐bearing pregnant mice using a murine model of gestational breast cancer.

**Study Design**: On estrous day 11, nulliparous female BALB/c mice were either injected with 20K 4T1 MCherry tumor cells in PBS (20uL) a model for triple‐negative breast cancer, or PBS (20uL) injected into the right 4th mammary duct (5 per group). These nulliparous BALB/c female mice were bred with BALB/c males (N = 10). Mice were euthanized on gestational day 19: pup numbers and fetal weights were recorded and maternal blood was collected. Isolated serum was frozen and analyzed using a cytokine array (Proteome Profiler Mouse Array Kit).

**Results**: Three of 5 mice injected with tumor cells and 1 of 5 mice injected with PBS were pregnant at time of specimen collection. Mice injected with tumor cells developed small but grossly visible mammary tumors. The mouse injected with PBS had 5 pups weighing ∼1.3g/pup, whereas mice injected with tumor cells had 1‐6 pups weighing ∼1.2g/pup. Cytokine array demonstrated absence of pro‐inflammatory cytokines in both serum samples and similar expression of CXCL13 and GM‐CSF. However, tumor‐injected serum demonstrated decreased IL‐10, an anti‐inflammatory cytokine implicated in tumorigenesis and pregnancy outcomes (4.27 units control to tumor‐injected serum expression), IL‐3, and IL‐4 (3.95 and 2.09 units control to tumor‐injected, respectively), regulators of adaptive immunity.

**Conclusion**: There does not appear to be a difference in pro‐inflammatory cytokine expression in serum of pregnant tumor‐affected mice; however, there is decreased anti‐inflammatory cytokine expression among individual markers. This was a pilot analysis on a small sample size; we plan to perform cytokine analysis on a larger murine sample size, to validate findings in the pregnant human population, and to examine murine cytokine expression of amniotic fluid and pups in this gestational breast cancer model.

## 1090 | Prevalence of and Factors Associated with Iron Deficiency at the time of Delivery

Logan Mauney^1^; Ashley Parker^2^; Kathleen J. Koenigs^2^; Pichliya Liang^1^; Sarah N. Bernstein^3^; Mark A. Clapp^1^

*^1^Massachusetts General Hospital, Boston, MA; ^2^Brigham and Women's Hospital, Boston, MA; ^3^Massachusetts General Hospital, Division of Maternal Fetal Medicine, Boston, MA*

4:00 PM ‐ 6:00 PM

**Objective**: Iron deficiency (ID) and iron deficiency anemia (IDA), the end‐stage manifestation of ID are associated with maternal and fetal morbidity in pregnancy and postpartum. While pregnant individuals are screened for anemia, there is no consensus on the role of routine ID screening. Our study sought to assess the prevalence of ID and IDA at the time of delivery.

**Study Design**: This pilot, single‐site, prospective cohort study recruited patients with singleton pregnancies at ≥ 36 weeks of gestation during routine prenatal care visits. Patients who had received IV iron during the pregnancy were excluded. Participants completed surveys about their use of iron supplementation during pregnancy and had iron studies and hemoglobin measured on admission for delivery. We report the prevalence of ID and IDA by varying definitions: ferritin < 30 ng/mL, transferrin saturation (TSAT) < 20%, or either criterion; anemia was defined as Hgb < 11 g/dL. Patient characteristics and the rates of anemia were compared among those with and without ID by ferritin criteria.

**Results**: Of 92 patients included, 45 (49%) had ferritin < 30 ng/mL, 52 (57%) had TSAT < 20%, and 60 (65%) had either (Figure 1). 10 patients (11%) had anemia, of which 8 (80%) had ferritin < 30 ng/mL. Patients with ID were more likely to be multiparous (60% vs. 36%, p = 0.04) and have higher BMI at delivery (median (IQR) 32.0 (28.5‐34.3) vs. 29.2 (27.3‐31.7), p = 0.02; Table 1). 13% of the subjects self‐identified as Asian; none had ID. Although the rates of anemia in the 3^rd^ trimester were similar, 19 patients without ID (40%) vs. 5 with ID (11%) reported taking an iron (p = 0.001). On admission, 8 (18%) with ID and 2 (4%) without ID were anemic (p = 0.048).

**Conclusion**: ID was highly prevalent at the time of delivery, exceeding previously published estimates, and associated with anemia. Further research is needed to evaluate the impact of ID at delivery as well as the treatment of ID with and without anemia on perinatal outcomes.

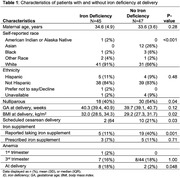

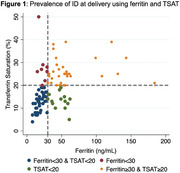

## 1091 | Patient‐Reported unmet Health‐Related Social Needs in Patients with and without Preterm Birth

Logan Mauney^1^; Jonathan Y. Siden^2^; Kaitlyn E. James^1^; Chengbo Zeng^3^; Sarah N. Bernstein^4^

*^1^Massachusetts General Hospital, Boston, MA; ^2^Massachusetts General Hospital, Harvard Medical School, Boston, MA; ^3^Brigham & Women's Hospital, Boston, MA; ^4^Massachusetts General Hospital, Division of Maternal Fetal Medicine, Boston, MA*

4:00 PM ‐ 6:00 PM

**Objective**: Preterm birth (PTB) is the leading cause of infant mortality and has been linked to social determinants of health (SDOH) such as economic stability, social and community context, and limited healthcare access. There is little evidence on the impact of unmet health‐related social needs (HRSNs), the patient‐level manifestations of these SDOH, on birth outcomes. We sought to characterize the impact of patient‐reported HRSNs on PTB.

**Study Design**: Retrospective cohort study of pregnant patients who completed a screen at least once in pregnancy for HRSNs in a regional health‐care system from 5/2020 to 3/2024. HRSNs in the domains of education, employment, family care, food, housing, medication affordability, transportation, and household utilities affordability were identified through a validated screening tool. The primary outcome was birth before 37 weeks’ gestation. Generalized estimating equations were generated to determine adjusted odds ratios (aOR) for the association between preterm birth and HRSN domains. Models were adjusted for advanced maternal age, nulliparity, hypertensive disorders of pregnancy, pre‐existing diabetes mellitus, and pregravid body mass index.

**Results**: There were 2,055 pregnancies screened for HRSNs that met inclusion criteria, of whom 156 (7.6%) had a PTB. Patients with a PTB were significantly more likely to report food insecurity (14.1% vs 8.2%; aOR 2.09 95% CI 1.22‐3.59) and unemployment (19.2% vs 11.2%; aOR 1.74 95%CI 1.07‐2.81). The presence of ≥ 2 or ≥ 3 HRSNs was associated with increased odds of PTB: ≥ 2 HRSNs aOR 1.68 (95% CI 1.12‐2.52); ≥ 3 HRSNs aOR 1.81 (95% CI 1.11‐ 2.95). Other HRSNs represented in the cohort were not significantly associated with risk of PTB.

**Conclusion**: Patient‐reported food insecurity and unemployment are independently associated with PTB. Further, HRSNs in 2 or more of any domains have nearly the same impact. Future research is needed to understand the mechanisms of these associations to inform critically needed patient and policy level interventions to reduce PTB.

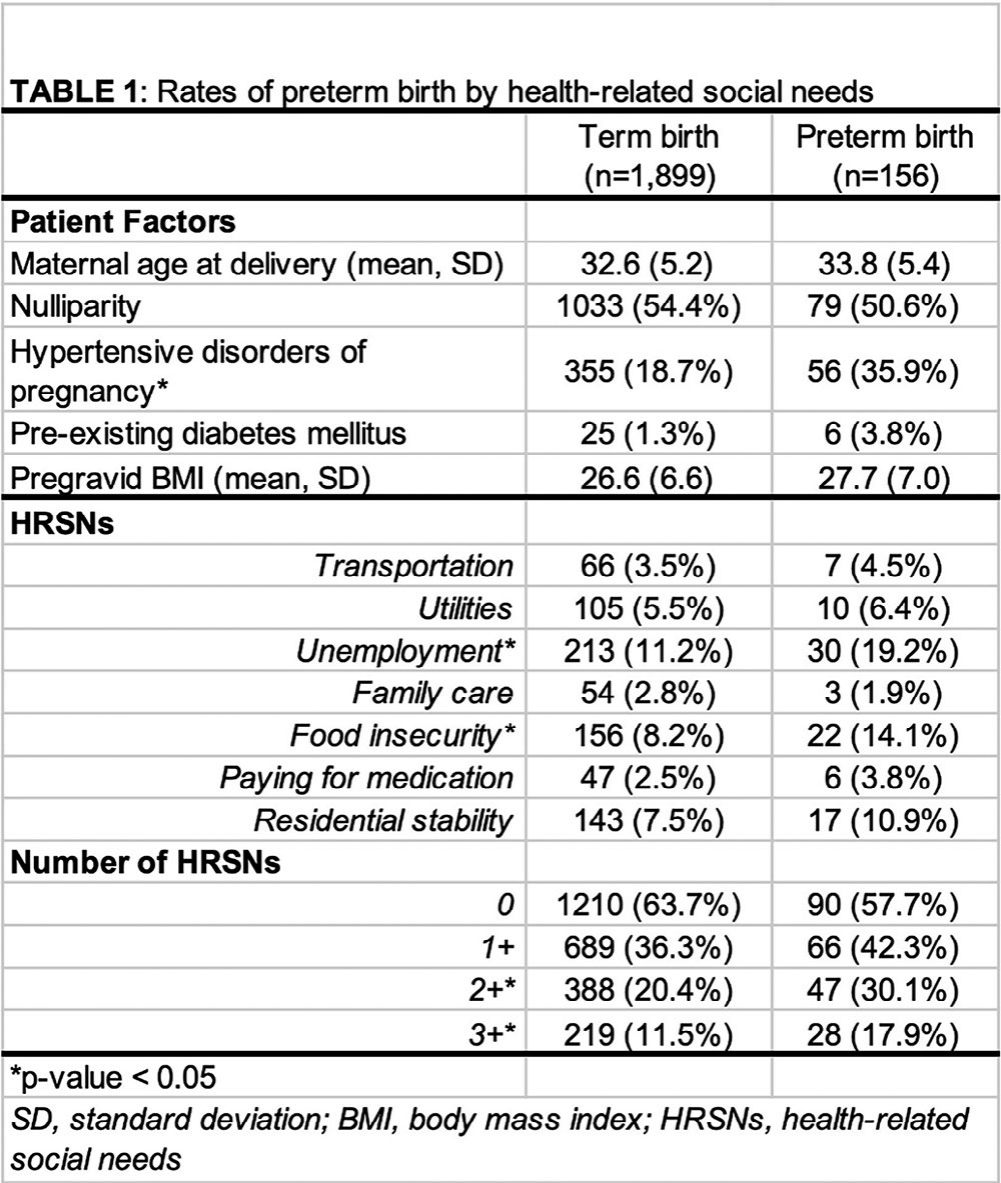

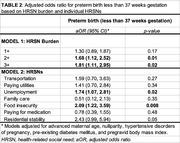

## 1092 | State Partisan Divide and Intimate Partner Violence before and during Pregnancy

Madeline F. Perry; Joe M. Feinglass; Lynn M. Yee

*Northwestern University Feinberg School of Medicine, Chicago, IL*

4:00 PM ‐ 6:00 PM

**Objective**: Pregnancy is a high‐risk period for intimate partner violence (IPV), with greater risk for adverse pregnancy outcomes among people who experience IPV. Our objective was to evaluate the association between a state's percent vote for Donald Trump in the 2020 presidential election and statewide IPV rates before and during pregnancy reported by birthing people.

**Study Design**: Data from 2016‐2020 from 44 states using the Pregnancy Risk Assessment Monitoring System (PRAMS) were linked to the state percent vote for Trump in the 2020 election. PRAMS surveys postpartum people two to four months after delivery. The survey asks participants if their current or past husband/partner “push, hit, slap[ped], kick[ed], choke[d], or physically hurt [them] in any way” in the 12 months before pregnancy and/or during pregnancy. Respondents who answered “yes” to either question were defined as reporting IPV. Our main outcome was the mean state‐reported prevalence of IPV in the 12 months before or during pregnancy. Multivariable Poisson regression estimated the association between the 2020 state percent vote for Trump and the mean IPV rate reported, controlling for individual‐level sociodemographic variables. Sensitivity analysis with 2016 election data was performed.

**Results**: Among 190,800 respondents representing an estimated population of 9,762,956, 3.1% of respondents reported experiencing IPV before or during pregnancy. There was a significant positive association (r = 0.62, p< 0.001) between mean state IPV rate and state percent vote for Trump in 2020 (Figure). After adjusting for respondent sociodemographic factors, each 10‐point increase in the state percent vote for Trump was associated with a nine percent greater likelihood of reporting IPV before or during pregnancy (aIRR 1.09, 95% CI 1.05, 1.13; Table). Findings were similar for 2016 election data (aIRR 1.08, 95% CI 1.04, 1.13).

**Conclusion**: Political and cultural environments are potential contributing factors to IPV prevalence before and during pregnancy. The state percent vote for Trump may be a signal of a greater prevalence or toleration of abusive behavior.

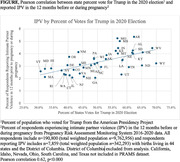

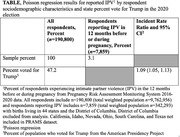

## 1093 | Exposed Fetal Membranes Prior to Physical Examination‐Indicated Cervical Cerclage and Time to Delivery

Mallory Vial^1^; Baha M. Sibai^2^; Emily Hyde^3^; Khalil M. Chahine^4^; Nahla Daye^1^; Sami Backley^5^; Sean C. Blackwell^2^; Ahmed Zaki Moustafa^6^

*^1^University of Texas Health Science Center at Houston, Houston, TX; ^2^McGovern Medical School at UTHealth Houston, Houston, TX; ^3^UT Houston, Houston, TX; ^4^McGovern Medical School at UTHealth, Houston, TX; ^5^Division of Fetal Intervention, Department of Obstetrics, Gynecology and Reproductive Sciences, McGovern Medical School at UTHealth Houston, Houston, TX; ^6^Division of Maternal‐Fetal Medicine, Department of Obstetrics, Gynecology and Reproductive Sciences, McGovern Medical School at UTHealth Houston, University of Texas ‐ Houston, TX*

4:00 PM ‐ 6:00 PM

**Objective**: Physical examination‐indicated cerclage (PEIC) is considered in the setting of painless cervical dilation with or without exposed fetal membranes. We hypothesize that exposed fetal membranes is a poor prognostic sign and associated with shortened latency from cerclage to delivery.

**Study Design**: This is a retrospective cohort study of all singleton pregnancies that underwent PEIC at a Level IV center between January 2015 and March 2024. Patients with no delivery records available for review were excluded. We compared demographic and clinical characteristics, latency from cerclage to delivery, and perinatal outcomes among patients with and without exposed fetal membranes at the time of surgery.

**Results**: Ninety‐three cases of PEIC were analyzed: 56 (60%) had exposed fetal membranes and 37 (40%) did not. There were no significant differences in the distribution of age, race, parity, or medical comorbidities between groups. Latency period was significantly shortened in patients with exposed membranes compared to patients without (median 5 vs 16 weeks, P = 0.008) (Figure 1). Exposed membranes was associated with higher rates of preterm birth at < 24, < 28, < 32, and < 37 weeks gestation respectively (32 vs 11%, P = 0.02; 55 vs 22%, P = 0.001; 57 vs 27%, P = 0.004; and 73 vs 46%, P = 0.01). Exposed membranes was also associated with higher rates of prelabor premature rupture of membranes (PPROM) and intraamniotic infection (IAI) (27 vs 8%, P = 0.008; 12 vs 2%, P = 0.04) (Table 1).

**Conclusion**: Sixty percent of patients with PEIC had exposed fetal membranes at the time of cerclage placement. Patients with exposed membranes had shortened latency periods and higher rates of preterm delivery < 24, < 28, < 32, and < 37 weeks. These data will inform provider counseling and patient expectations when considering PEIC.

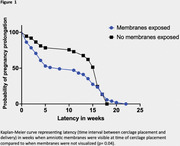

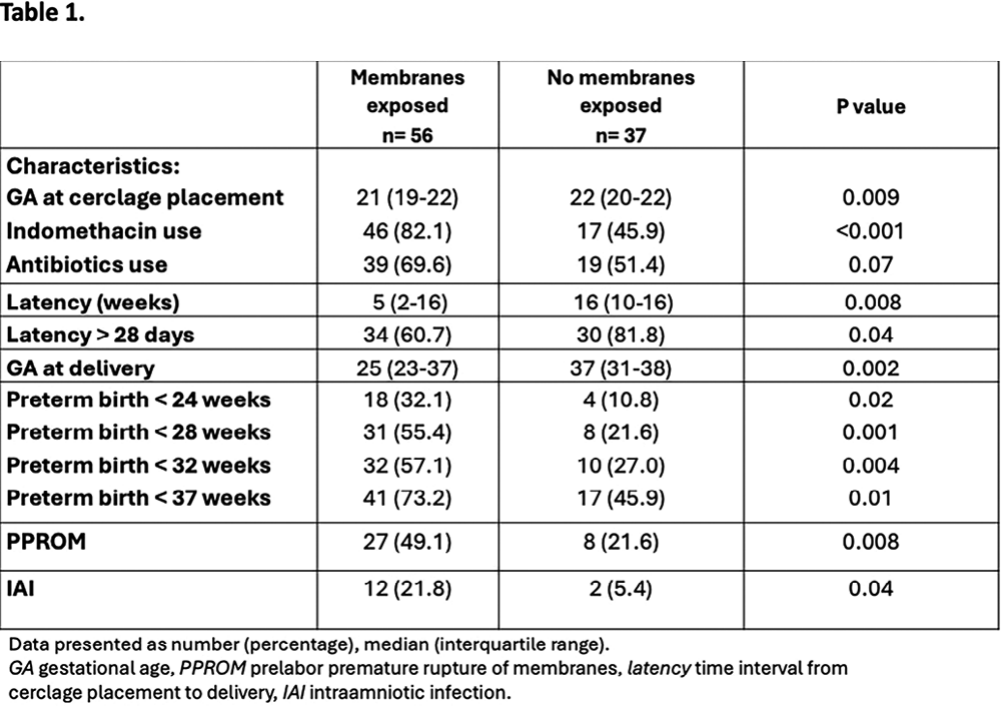

## 1094 | The Accuracy of Crown‐Rump Length Measurements: Comparison of 5‐7 with 8‐10 Gestational Weeks

Manal Massalha^1^; Maria Abbas^2^; Etty Daniel‐Spiegel^2^; Enav Yefet^3^; Zohar Nachum^1^

*^1^Emak Medical Center, Afula, HaZafon; ^2^Emek Medical Center, Emek Medical Center, Afula, HaZafon; ^3^Tzafon Medical Center, Poriya, HaZafon*

4:00 PM ‐ 6:00 PM

**Objective**: The International Society of Ultrasound in Obstetrics and Gynecology recommends for dating to use ultrasound (US) measurements at 8‐13gestational weeks (GW), and to change the dating if the difference from the last menstrual period (LMP) is of 6d or more. We aimed to evaluate the accuracy of the crown‐rump length (CRL) at an earlier stage of 5‐7GW compared with 8‐10GWand to estimate if 5d difference justifies dating correction.

**Study Design**: A retrospective cohort study was conducted. Data was retrieved from the medical records of women treated in the assisted reproductive technology (ART) clinic between 2016‐2023 at Emek Medical Center, Afula, Israel. The LMP was calculated from embryo age and the date of transfer. The study and control groups included women who conceived by ART a singleton pregnancy and performed an US exam at 5+5‐7+6 or/and 8‐10+6GW, respectively. Transvaginal US was performed using the Voluson E10 BT20 device by experienced technicians at the US unit. Women with severe chronic diseases (e.g. chronic hypertension, pre‐gestational diabetes, autoimmune diseases and heart or renal diseases) were excluded. The primary outcome was the accuracy of the CRL values at a range of ±4d according to Hadlock at 5+5(CRL = 2mm)‐7+6 compared with 8‐10+6(CRL = 42mm)GW. Assuming that 20% of the US CRL measurements at 5+7‐7+6GW will differ at least 5d from the calculated GW compared with 10% of the measurements at 8‐10+6GW, a sample size of 400 pregnancies was required (α = 0.05, power = 80%).

**Results**: Included in the study 443 women, who performed 1045 US exams (mean 2.4/woman). The study and control groups performed 575 and 470 exams, respectively, and the accuracy of CRL measurements of ±4d within the LMP was 96% and 99%, respectively (P = 0.02). High rate of accuracy of 95‐100% was found throughout all GWs (Table and Figure).

**Conclusion**: Although the control group performed slightly better, the early 5+5‐7+6GW group has a high accuracy of CRL measurements and can be used for dating. We recommend dating correction if a 5d difference or more is found using transvaginal US exam during 5+5‐10+6GW.

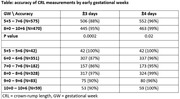

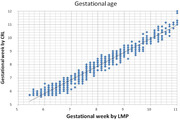

## 1095 | Intellectual Disability as a risk Factor for Adverse Outcomes Among Pregnant People with Diabetes

Manasa G. Rao^1^; Timothy Wen^2^; Mary E. D'Alton^1^; Alexander M. Friedman^3^; Noelia Zork^3^

*^1^Columbia University Medical Center, New York, NY; ^2^University of California, San Diego, Irvine, CA; ^3^Columbia University Irving Medical Center, New York, NY*

4:00 PM ‐ 6:00 PM

**Objective**: Intellectual and developmental disabilities (IDD) are associated with increased risk of adverse pregnancy outcomes and the proportion of pregnant patients with a IDD diagnosis has increased over time. Diabetes mellitus (DM) requires more intensive antenatal care including frequent glucose monitoring, medication adherence and titration, and frequent visits. It is unclear whether IDD is an independent risk factor for adverse outcomes among pregnancies with DM. The purpose of this analysis was to evaluate whether IDD was associated with adverse outcomes among deliveries complicated by DM.

**Study Design**: Delivery hospitalizations to patients with pregestational and gestational DM aged 15‐54 were analyzed using the 2000‐2021 Nationwide Inpatient Sample. The primary exposure of interest was IDD defined using ICD9/ICD10 codes. Temporal trends in proportion of deliveries with IDD and DM were analyzed with joint point regression to determine average annual percent change (AAPC) with 95% confidence intervals. The association between hospital, demographic, and a range of adverse outcomes were analyzed with logistic regression models.

**Results**: 2389 (0.04%) deliveries of 6,012,324 with DM diagnosis had an associated IDD diagnosis. Deliveries to patients with both DM and IDD increased from 2 per 10,000 in 2000 to 7 per 10,000 in 2021 (AAPC 7.8, 6.4‐10.3, p< 0.01). More patients with IDD had pregestational DM (30.2% vs 13.5%). In adjusted analyses, patients with IDD had increased odds of diabetic ketoacidosis (aOR 2.66, 1.06‐6.69), preterm birth < 37 weeks (aOR 1.46, 1.10‐1.92), cesarean delivery (aOR 1.30, 1.05‐1.60), non‐transfusion severe maternal morbidity (aOR 2.06, 1.26‐3.32), hypertensive disorders of pregnancy (aOR 1.29, 1.03‐1.63), and stillbirth (aOR 1.94, 1.07‐3.5). Odds of shoulder dystocia did not differ between the groups.

**Conclusion**: Among deliveries with DM, the proportion of IDD increased over time. IDD is associated with increased risk of adverse outcomes among deliveries with DM. Additional resources and surveillance may be warranted for patients with IDD and DM to optimize outcomes.

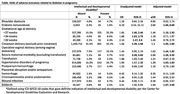

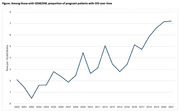

## 1096 | Race and Temporal Temperature Measurement in Intraamniotic Infection

Maria A. Phillis^1^; Peyton Johnston^1^; Esha Ghosalkar^2^; David N. Hackney^1^

*^1^University Hospitals Cleveland Medical Center, Cleveland, OH; ^2^Case Western Reserve University, Cleveland, OH*

4:00 PM ‐ 6:00 PM

**Objective**: Temperature is underestimated in Black versus White inpatients when temporal thermometers are used. There is no data on racial differences in temporal temperature measurements in obstetrical patients in assessment of Intraamniotic infection (IAI). Our aim was to analyze intrapartum temporal temperature readings in self‐identified Black versus White patients with IAI. We hypothesized that Black patients would have significantly lower max temperature (Tmax) when measured temporally and this may result in delay of diagnosis or antibiotics, higher stage or grade of chorioamnionitis on pathology or neonatal and maternal morbidity.

**Study Design**: We conducted a single institution retrospective cohort study of self‐identified Black and White patients at term with clinical IAI or histopathological diagnosis of chorioamnionitis from 2021‐2022. Primary outcomes were differences in Tmax in Black versus White patients with clinically diagnosed IAI and Tmax differences in Black versus White patients with histopathological chorioamnionitis. All temperatures were measured with temporal thermometers. Secondary outcomes included differences in stage and grade of chorioamnionitis, timing of diagnosis and antibiotics, and neonatal and maternal complications.

**Results**: 459 patients were identified that met the inclusion and exclusion criteria, 285 (62.1%) Black patients and 174 (37.9%) White patients, with Black patients more likely to be diagnosed with IAI. Black patients were younger, more often multiparous, with higher BMIs and a higher likelihood of hypertensive diseases. There was no difference in primary outcome of Tmax by race. Black patients with a clinical diagnosis of IAI were more likely to undergo cesarean delivery. Infants of White patients with a histopathological diagnosis of chorioamnionitis were more likely to be admitted to the NICU.

**Conclusion**: Temporal thermometer measurements were not statistically significant by race. However there were disparities in cesarean delivery rates and NICU admissions.

## 1098 | Risk Factors for Unplanned Cesarean Delivery Among Pregnancies with Preexisting Diabetes

Marie‐Julie Trahan^1^; Qi Yan^2^; Noelia Zork^2^; On behalf of the MOMPOD Consortium

*^1^Columbia University, New York, NY; ^2^Columbia University Irving Medical Center, New York, NY*

4:00 PM ‐ 6:00 PM

**Objective**: Diabetes in pregnancy is associated with an increased risk of cesarean delivery (CD). While CD risk factors among pregnancies with gestational diabetes (GDM) are well defined, such as nulliparity, body mass index, gestational weight gain, and insulin use, it is unclear whether these same risk factors apply to pregnancies with preexisting diabetes. The objective of this study was to identify risk factors for unplanned CD among pregnancies with Type 2 diabetes (T2D) or early GDM.

**Study Design**: This was a secondary analysis of the Metformin Plus Insulin for Preexisting Diabetes or Gestational Diabetes in Early Pregnancy (MOMPOD) randomized controlled trial. Participants with T2D or early GDM were included if they had a trial of labor. Baseline and pregnancy characteristics were compared between those with a vaginal delivery (VD) and those with an unplanned CD using descriptive and logistic regression analyses, controlling for potential confounders.

**Results**: Of 405 participants, 144 (36%) had an unplanned CD and 261 (64%) had a VD. There were no differences in diabetes type, metformin use, or neonatal birthweight between groups. Compared to those with a VD, participants with an unplanned CD had greater median gestational weight gain [11.6 pounds (interquartile range (IQR) 6.8‐15.4) vs. 10 pounds (IQR 4.4‐14.9), p = 0.019], enrollment HbA1c ≥ 6.5% (69% vs. 56%, p = 0.0239), third trimester HbA1c ≥ 6.5% (39% vs. 25%, p = 0.0459), and median glucose levels in labor [130.0 mg/dL (IQR 112.0‐161.5) vs. 121.0 mg/dL (IQR 100.0‐144.0), p = 0.0025]. There was no difference in median third trimester HbA1c. In multivariate adjusted analysis, only median enrollment HbA1c (mean gestational age 11.2 weeks) was associated with CD [7.3% for CD vs. 6.85% for VD, p = 0.0107; aOR 1.21 (95% confidence interval 1.03, 1.42)].

**Conclusion**: Higher first trimester HbA1c is associated with CD in patients with T2D or early GDM attempting VD. Preconception counseling should focus on improved glycemic control prior to and in early pregnancy as a potential strategy to reduce risk of unplanned CD for pregnant individuals with preexisting diabetes.

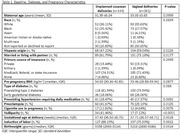

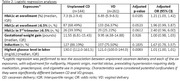

## 1099 | Anal Sphincter Injury After Vacuum‐Assisted Delivery‐ is Avoiding Episiotomy Still Safe?

Matan Anteby^1^; Tally Pinchas‐Cohen^1^; Asnat Groutz^1^; Ronen Gold^1^; Anat Lavie^2^; Yariv Yogev^3^; Yoav Baruch^1^

*^1^Lis Maternity Hospital, Sourasky Medical Center, Tel Aviv University, Israel, Tel Aviv, Tel Aviv; ^2^Tel Aviv Sourasky Medical Center, Tel Aviv Sourasky Medical Center, Tel Aviv; ^3^Lis Maternity Hospital, Sourasky Medical Center, Tel Aviv University, Tel Aviv Sourasky Medical Center, Tel Aviv*

4:00 PM ‐ 6:00 PM

**Objective**: To assess whether avoiding episiotomy during vacuum‐assisted deliveries (VAD) affects the incidence of anal sphincter injury (OASI) in primiparous women.

**Study Design**: A retrospective cohort study of all primiparous patients who had a VAD at a university‐affiliated tertiary center (2011‐2022). In our institution, during VAD, a mediolateral episiotomy is performed at the attending physician's discretion. We compared outcomes between VADs with and without episiotomy. Baseline characteristics were evaluated along with various VAD parameters. The primary outcome was OASI, with secondary outcomes including other maternal adverse events.

**Results**: Out of 157,282 deliveries, 10,400 (6.6%) were VADs, and 7,951 met the inclusion criteria. Among these, 7,201 (90.6%) had episiotomies, and 750 (9.4%) did not. Baseline maternal characteristics were similar between the groups. Those who had episiotomy had a higher rate of prolonged second stage of labor (PSS) (32% vs. 25.9%, p = 0.001), higher rates of occipitoposterior position (OP) (18.6% vs. 14.3%, p = 0.016) and more advanced head station (+3 under ischial spine in 5.3% vs. 9.3%, p< 0.001), as compared to no episiotomy. Newborns weighing >3500 grams were more common in the episiotomy group (25.4% vs. 21.7%, p = 0.027).

Despite these known risk factors for OASI, the incidence of OASI was higher in the no‐episiotomy group (2.8% vs. 1.4%, p = 0.004). Multivariable analysis confirmed that episiotomy had a protective effect against OASI [aOR 0.433 (0.258‐0.728), p = 0.002]. Prolonged second stage [aOR 1.98 (1.32‐2.94), p< 0.001] and higher birthweight [aOR 1.86 (1.09‐3.17), p = 0.022] also correlated with a higher risk of OASI. The number needed to treat (NNT) to prevent one OASI with episiotomy was 71, reduced to 42 for PSS, 33 for OP, and 22 for newborns >3500 g. Other maternal adverse outcomes were similar, except for a higher postpartum blood transfusion rate in the episiotomy group (3.9% vs. 1.5%, p = 0.008).

**Conclusion**: Avoiding routine episiotomy during VAD in primiparous women is associated with a higher OASI rate, particularly in those with PSS, OP, or larger newborns.

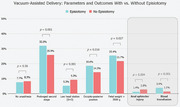

## 1100 | Normotensive vs. Hypertensive HELLP Syndrome: Should We Expect Different Outcomes?

Matan Anteby^1^; Keren Or Wertheimer^1^; Liran Hiersch^2^; Yariv Yogev^3^

*^1^Lis Maternity Hospital, Sourasky Medical Center, Tel Aviv University, Israel, Tel Aviv, Tel Aviv; ^2^Lis Maternity Hospital, Sourasky Medical Center, Tel Aviv University, Israel, Lis Hospital for Women's Health, Tel Aviv Sourasky Medical Center, Tel Aviv; ^3^Lis Maternity Hospital, Sourasky Medical Center, Tel Aviv University, Tel Aviv Sourasky Medical Center, Tel Aviv*

4:00 PM ‐ 6:00 PM

**Objective**: To compare maternal and neonatal outcomes between women with hemolysis, elevated liver enzymes, and low platelet (HELLP) syndrome with and without associated hypertension, focusing on differences in presentation, disease severity, and associated complications.

**Study Design**: A retrospective cohort study of singleton deliveries diagnosed with HELLP syndrome at a tertiary university‐affiliated hospital (1.2011‐3.2023). Cases were categorized into normotensive HELLP and hypertensive HELLP. We collected and compared data on maternal demographics, clinical presentation, laboratory findings, delivery outcomes, and neonatal complications.

**Results**: Among 157,282 deliveries, 118 met the full criteria for HELLP syndrome: 74 (68.5%) hypertensive and 34 (31.5%) normotensive. Normotensive HELLP cases were diagnosed at a significantly later gestational age (37.5 ± 3.0 vs. 34.5 ± 4.4 weeks, p< 0.001). Only 2 women (5.9%) with normotensive HELLP delivered < 34 weeks, compared to 33 (44.6%) with hypertensive HELLP (p< 0.001) (figure). Proteinuria was less common in normotensive cases (58.8% vs. 87.8%, p< 0.001). Laboratory parameters, including hemoglobin, platelet count, creatinine, liver function tests, and hemolysis parameters, did not differ significantly between the groups. Neonates in the normotensive group had significantly higher mean birth weights (2574.6 ± 746.2 vs. 1947 ± 816.6 grams, p< 0.001), fewer NICU admissions (29.4% vs. 56.8%, p = 0.008), and lower rates of respiratory distress syndrome (2.9% vs. 24.3%, p = 0.007) (figure). Maternal complications related to HELLP syndrome did not differ significantly between normotensive and hypertensive cases (figure).

**Conclusion**: Normotensive HELLP syndrome appears to be a less severe form of HELLP syndrome, presenting at a later gestational age. Despite differences in neonatal outcomes, maternal adverse outcomes remain comparable to those in hypertensive HELLP syndrome. This suggests that normotensive HELLP should be managed with the same caution as hypertensive HELLP to ensure optimal maternal and neonatal outcomes.

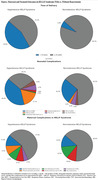

## 1101 | Optimizing Induction: Shortened Balloon Protocols for Better Outcomes

Matthew D. Mitts^1^; Kamran Hessami^1^; Meija Caldwell^1^; Christina M. Ackerman‐Banks^1^; Karin A. Fox^2^

*^1^Baylor College of Medicine, Houston, TX; ^2^University of Texas Medical Branch, Galveston, TX*

4:00 PM ‐ 6:00 PM

**Objective**: The optimal timing of mechanical balloon induction remains controversial. This meta‐analysis compares the effectiveness of 6‐hour versus 12‐hour mechanical balloon induction protocols in reducing delivery time in term pregnancies.

**Study Design**: A comprehensive literature search was conducted using terms related to mechanical balloon induction. Randomized controlled trials (RCTs) comparing 6‐hour and 12‐hour induction protocols with various types of balloon catheters (single and double balloon) were included. The primary outcome was the interval time to delivery, defined as the time from the start of the induction process to delivery. The secondary outcomes were cesarean delivery rate (CD), operative vaginal delivery rate, time on oxytocin, and delta bishop score. The random‐effects model was used to pool the odds ratios (ORs) and the corresponding 95% confidence intervals (CIs).

**Results**: Four RCTs were included in the meta‐analysis, encompassing a total of 972 participants. Overall, patients with a 6‐hour balloon induction delivered 4.5 hours sooner compared to those with a 12‐hour balloon (mean difference ‐4.56, CI [‐5.75,‐3.37], P< 0.001). The risk of cesarean delivery was 1.5 times higher for patients with a 12‐hour balloon compared to those with a 6‐hour balloon (OR 0.67, 95% CI [0.47, 0.96], P = 0.028). Additionally, the risk of cesarean delivery due to non‐reassuring fetal heart tones (NRFHTs) was 1.7 times higher in the 12‐hour group (OR 0.59, 95% CI [0.36, 0.96], P = 0.030). No significant differences were found in the rate of operative vaginal delivery, time on oxytocin, change in Bishop score, or starting and post‐intervention Bishop scores between the two groups.

**Conclusion**: A 6‐hour mechanical balloon induction protocol significantly reduces the interval to delivery and the risk of cesarean delivery, particularly due to NRFHTs, compared to a 12‐hour protocol. These findings support the use of a shorter induction interval for improved maternal and fetal outcomes in term pregnancies.

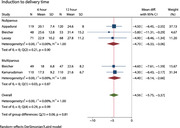

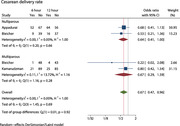

## 1102 | Neonatal Outcomes of Gastroschisis Births by mode of Delivery in the United States

Matthew H. Mossayebi^1^; Jennifer E. Powel^2^; Rodney A. McLaren, Jr., Jr.^3^; Zubair H. Aghai^4^; Virali Patel^4^; Mariella F. Toro^4^; Huda B. Al‐Kouatly^3^

*^1^Brigham and Women's Hospital, Boston, MA; ^2^Hackensack Meridian Jersey Shore University Medical Center, Neptune, NJ; ^3^Thomas Jefferson University Hospital, Philadelphia, PA; ^4^Sidney Kimmel Medical College at Thomas Jefferson University, Philadelphia, PA*

4:00 PM ‐ 6:00 PM

**Objective**: Although trial of labor (TOL) versus planned caesarean delivery (CD) has been hypothesized to influence clinical outcomes in neonates with gastroschisis, this clinical decision remains controversial. We assessed neonatal outcomes in newborns with gastroschisis based on delivery mode in a contemporary United States cohort.

**Study Design**: We conducted a retrospective cohort study using population‐based data from the US Natality Database on live singleton births complicated by gastroschisis from 2017 to 2022. Data was stratified by TOL versus CD. A sub‐group analysis for term versus preterm neonates was performed. Cases with CD after failed TOL were included in the TOL group. Our primary outcome was composite adverse neonatal outcome, including chorioamnionitis, 5‐minute Apgar score < 3, assisted ventilation use > 6 hours, surfactant use, neonatal seizures, and neonatal death. Patient characteristics were compared and logistic regression was performed to estimate adjusted odds ratios (aORs) for adverse outcomes accounting for gestational age (GA) at birth and birthweight.

**Results**: Of 17,104,917 births, 3,543 (0.021%) were complicated by gastroschisis. TOL was attempted in 2,433 (68.7%), while 1,110 (31.3%) reported planned CD. Compared to the planned CD group, those undergoing TOL had lower rates of preterm birth, delivered at later GA, and had higher birthweights (Table 1). TOL was associated with lower odds of composite adverse neonatal outcome (25.5% vs 31.4%, aOR 0.79). TOL was also associated with lower odds of NICU admission (aOR 0.53) and immediate ventilation use (aOR 0.73), and higher odds of chorioamnionitis (OR 2.12) and infant transfer to a higher‐level care facility (OR 1.23) (Table 2). Similar results were observed after stratification for term versus preterm neonates.

**Conclusion**: TOL was associated with lower composite adverse neonatal outcome compared to planned CD among pregnancies complicated by gastroschisis. This study is limited by variables of antenatal morbidity, such as fetal growth restriction and size of gastroschisis.

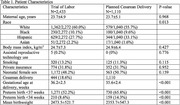

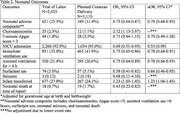

## 1103 | Antenatal Ultrasound Predictors of Genitourinary Injury in Cesarean Hysterectomy for Placenta Accreta Spectrum

Matthew D. Mitts^1^; Kamran Hessami^1^; Yamely H. Mendez^1^; Christina C. Reed^2^; Arthur Ladron De Guevara^1^; Keneshia Lane^1^; Kayla E. Ireland^3^; Georgia A. McCann^3^; Claire Hoppenot^1^; Amir A. Shamshirsaz^1^; Hendrik A. Lombaard^4^; Michael A. Belfort^5^; Jessian L. Munoz^1^

*^1^Baylor College of Medicine, Houston, TX; ^2^Baylor College of Medicine at Texas Children's Pavilion for Women, Houston, TX; ^3^University of Texas Health Science Center at San Antonio, San Antonio, TX; ^4^Texas Children's Hospital, Houston, TX; ^5^Texas Children's Hospital and Baylor College of Medicine, Houston, TX*

4:00 PM ‐ 6:00 PM

**Objective**: Placenta accreta spectrum (PAS) is a complex obstetric condition associated with maternal morbidity. Urologic injuries complicate 20‐30% of PAS deliveries during cesarean hysterectomy. This study aimed to evaluate ultrasonographic predictors of genitourinary (GU) injury during Cesarean Hysterectomy for PAS from multiple referral centers.

**Study Design**: We conducted a multicenter, retrospective analysis of singleton pregnancies with pathology‐confirmed PAS (accreta, increta, or percreta). Ultrasonographic variables assessed included placental location, presence of previa or low‐lying placenta, presence of lacunae, loss of retroplacental clear zone, lower segment hypervascularity, bladder and uterine bulge, myometrial thinning, abnormal uterine‐bladder interface, and suspected depth of invasion. The primary outcome was the occurrence of any GU injury (ureteral or bladder) during cesarean hysterectomy. Comparisons were made using Fisher's Exact test, and odd ratios were established using logistic regression.

**Results**: Among 358 patients included in the study, 59 (16%) experienced GU injury during surgery. Patients with GU injury were significantly more likely to have had antenatal suspicion of percreta based on ultrasound findings (OR 2.89, 95% CI: 1.56–5.35) and to have complete placenta previa (OR 3.26, 95% CI: 1.71–6.21), as seen in Table 1. Conversely, posterior placental location was associated with a lower risk of GU injury (OR 0.31, 95% CI: 0.11–0.89). None of the six ultrasonographic markers independently predicted GU injury.

**Conclusion**: Antenatal suspicion of percreta and complete placenta previa significantly increase the risk of GU injury during Cesarean Hysterectomy for PAS. Conversely, posterior placental location appears to be associated with a lower risk of such injuries. Individual ultrasonographic markers alone do not reliably predict GU injury at the time of cesarean hysterectomy; thus, multimodal imaging may be important for patient counseling and surgical preparation.

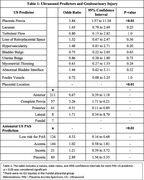

## 1104 | Azithromycin Utilization and Maternal Outcomes in Patients with Intrapartum Cesarean Birth, 2016‐2020

Maura Jones Pullins^1^; Marie‐Louise Meng^2^; Jerome J. Federspiel^2^; Kim Boggess^3^; Matthew Fuller^4^; Johanna Quist‐Nelson^1^

*^1^University of North Carolina, Chapel Hill, NC; ^2^Duke University School of Medicine, Durham, NC; ^3^University of North Carolina at Chapel HIll, Chapel Hill, NC; ^4^Duke University, Durham, NC*

4:00 PM ‐ 6:00 PM

**Objective**: In 2016, the Cesarean Section Optimal Antibiotic Prophylaxis (C/SOAP) study demonstrated perioperative azithromycin at time of labored cesarean delivery reduced infection by 50% (12% to 6%). We examined national trends in perioperative azithromycin use following C/SOAP. We also evaluated association between azithyromicin use and maternal sepsis and death.

**Study Design**: This retrospective cohort study used the Premier Healthcare Database to identify births from 2016‐2020 in patients aged 12 to 55, at > 24 weeks’ gestation, with a singleton pregnancy and intrapartum cesarean or cesarean after rupture of membranes. Those with chorioamnionitis prior to delivery were excluded. The primary outcome was azithromycin use. Secondary outcomes were a composite endpoint of infectious and surgical wound complications, endometritis, necrotizing fasciitis, abscess, septic pelvic thrombophlebitis, sepsis, pyelonephritis, pneumonia, meningitis and death during delivery hospitalization or the following 30 days.

**Results**: From 2016 to 2020, there were 1,528,602 cesarean deliveries, of which 531,709 met inclusion criteria. Azithromycin recipients were more likely than Azithyromicin non‐recipients to have diabetes, hypertension, and tobacco use disorder. Azithromycin use increased from < 1% to nearly 40% from 2016‐2020 (Figure). Utilization was highest in hospitals with > 500 beds compared to hospitals with < 100 beds (41.4% vs 4.7% p < 0.001). Despite increasing azithromycin use, the composite outcome was higher among patients receiving Azithyromicin, as was endometritis, length of stay, and home health use (Table).

**Conclusion**: After C/SOAP, the uptake of azithromycin was rapid and faster in larger hospitals. Further implementation should focus on smaller rural hospitals where uptake remains low, which may reflect limited financial resources, or capacity for implementation. Given outcomes assessment was limited to inpatient encounters and this cohort was not randomized, we encourage caution when evaluating associations between azithromycin exposure and outcomes.

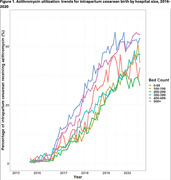

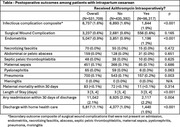

## 1105 | Trial of Labor After Cesarean in Twin Pregnancies with Two Prior Cesarean Deliveries

May Abiad^1^; William A. Grobman^2^; Kelly Mosesso^3^; Joanne Daggy^3^; Ali Javinani^4^; Asma Khalil^5^; Vincenzo Berghella^6^; Mark B. Landon^2^; Alireza A. Shamshirsaz^7^; Hiba J. Mustafa^8^

*^1^Maternal Fetal Care Center, Boston Children's Hospital, Boston, MA; ^2^The Ohio State University, Columbus, OH; ^3^Department of Biostatistics and Health Data Science, Indiana University School of Medicine, Department of Biostatistics and Health Data Science, Indiana University School of Medicine, IN; ^4^Boston Children's Hospital, Harvard Medical School, Boston, MA; ^5^Fetal Medicine Unit, St George's Hospital, St George's University of London, London, England; ^6^Sidney Kimmel Medical College of Thomas Jefferson University, Philadelphia, PA; ^7^Fetal Surgeon Chief, Division of Fetal Medicine and Surgery Director of the Maternal Fetal Care Center Director of the Perinatal Surgery Fellowship Professor of Surgery, Boston Children's Hospital Harvard Medical School, Boston, MA; ^8^Indiana University and Riley Children's Hospital, Indianapolis, IN*

4:00 PM ‐ 6:00 PM

**Objective**: To investigate short‐term maternal and neonatal outcomes in individuals with twin pregnancies and two prior CDs undergoing TOLAC.

**Study Design**: A cross‐sectional study of live birth data was conducted between 2014‐2021 in the United States. Individuals with twins and two prior CDs undergoing TOLAC were included. Comparison groups included those with: (1) twins and two prior CDs undergoing elective CD, (2) twins and one prior CD undergoing TOLAC, and (3) singletons and two prior CDs undergoing TOLAC. The primary outcomes were composite measures of maternal and neonatal morbidity. Maternal outcomes included chorioamnionitis, transfusion, hysterectomy, uterine rupture, ICU admission, and vaginal birth after cesarean (VBAC). Neonatal outcomes included 5‐min Apgar ≤ 3, assisted ventilation, NICU admission, surfactant or antibiotic use, and seizures.Univariable and multivariable analyses were conducted with P value < 0.05 considered significant after Bonferroni adjustment.

**Results**: A total of 92,665 pregnant individuals and 106,361 neonates were isolated. VBAC was achieved in 37.8% (239/632) of individuals with twins and two prior CDs attempting TOLAC compared to 61.5% (2,271/3,693) in twins and one prior CD and 58% (45,834/78,969) in singletons and two prior CDs undergoing TOLAC (*P*< 0.001). Both composite maternal and neonatal morbidity were not significantly different between individuals with twin pregnancies and two prior CDs attempting TOLAC and other twin groups. None of the 632 individuals with twins and two prior CDs attempting TOLAC had a uterine rupture. Following adjustments with covariates, the odds of VBAC were more than twice as great in those with twins with one prior CD (aOR: 2.41; 95% CI: 2.01‐2.90) and those with singletons with two prior CDs (aOR: 2.23; 95% CI: 1.88‐2.65) compared to individuals with twins and two prior CDs.

**Conclusion**: In twin pregnancies with two prior CDs, although the chance of VBAC was 37.8%, there was no significant difference in adverse maternal or neonatal outcomes.

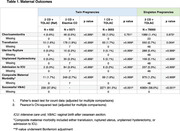

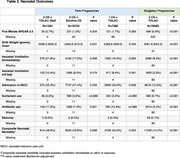

## 1106 | Role of Prenatal Care in Modifying the Risk of NAS Among Mothers with OUD

Megha Arora; Bharti Garg; Aaron B. Caughey

*Oregon Health & Science University, Portland, OR*

4:00 PM ‐ 6:00 PM

**Objective**: To examine the risk of neonatal abstinence syndrome (NAS) among mothers with opioid use disorder with low and high engagement with prenatal care.

**Study Design**: We conducted a retrospective cohort study of births in the state of California from 2008‐2020 among individuals with opioid use disorder to compare rates of NAS between those with < 5 prenatal visits and ≥5 prenatal visits. NOWs rates were assessed in the overall cohort then stratified by urban/rural residence. We performed multivariable logistic regression controlling for age, race/ethnicity, education, pre‐pregnancy BMI, parity, and insurance status to estimate adjusted odds ratios (aOR) and 95% confidence intervals.

**Results**: In the cohort of 10,654 individuals with opioid use disorder, 2,147 (20.15%) attended < 5 prenatal visits and 8,507 (79.85%) attended ≥5 prenatal visits. There was lower prenatal care attendance among individuals aged 20‐34, those with public insurance, and those with high school education or less. In the overall cohort, the adjusted odds of NAS was significantly higher among individuals with < 5 prenatal visits (aOR = 1.29; 95% CI 1.15‐1.44). Similar significant results were found in rural residents; however, the results were not statistically significant in urban residents.

**Conclusion**: Our study found that among pregnant individuals with opioid use disorder, those with lower prenatal care had a higher risk of NAS in the infant. Close follow up of pregnant individuals with opioid use disorder through prenatal visits may reduce the burden of NAS among neonates.

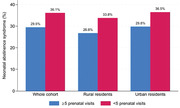

## 1107 | What to Expect when you're Expecting: Impact of Physician and Patient factors on Predicted Delivery

Melissa S. Wong^1^; So Yung Choi^1^; Karla Gonzalez^1^; Samira Torna^1^; Matthew Wells^1^; Alex A. T. Bui^2^; Kimberly D. Gregory^3^

*^1^Cedars‐Sinai Medical Center, Los Angeles, CA; ^2^Medical & Imaging Informatics Group, University of California, Los Angeles, Los Angeles, CA; ^3^Cedars Sinai Medical Center, Los Angeles, CA*

4:00 PM ‐ 6:00 PM

**Objective**: The cesarean delivery rate continues to rise, and it is critical to understand how physician perceptions of likely mode of delivery influence the delivery outcome. The aim of this study was to perform an early, intrapartum assessment of physician's predictions of their patient's likelihood of vaginal delivery (VD) and to identify which physician and patient factors affect these predictions.

**Study Design**: We prospectively assessed physician prediction of anticipated mode of delivery for patients presenting to labor and delivery for planned vaginal delivery. At 4 hours, physicians were sent a single‐item REDCap survey, “On a scale of 1‐10 (where 10 = the highest), what is your patient's likelihood of a vaginal delivery?” and the value was converted to a probability (0‐1.0).

We chart abstracted patient demographics and outcomes and obtained physician demographics through publicly available sources. We used linear mixed models to assess differences in predictions by patient and physician characteristics, and performed subgroup analysis for nulliparous, term, singleton vertex (NTSV) patients.

**Results**: We received 203 physician responses from 46 physicians. Table 1 lists physician and patient demographics. The median prediction was 0.9. Physicians predicted a lower likelihood of VD for patients with obesity and a higher likelihood of VD for multiparas. (Table 2)

The NTSV subgroup was 111 patients delivered by 39 physicians. Physician and patient characteristics were similar to the overall population. The median prediction was 0.8. Physicians predicted a lower likelihood of VD for patients age > 35 and for BMI >30 and a higher likelihood of VD for Asian patients. There were no differences in predictions by physician demographics (age, gender, language, practice duration).

**Conclusion**: Numerous patient factors influence physician's perception of the probability of a VD. Given that we anticipate the physician's early perception impacts the eventual outcome (anchoring bias), future research will focus on the importance of early recognition to determine strategies to mitigate those without causal association.

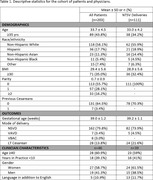

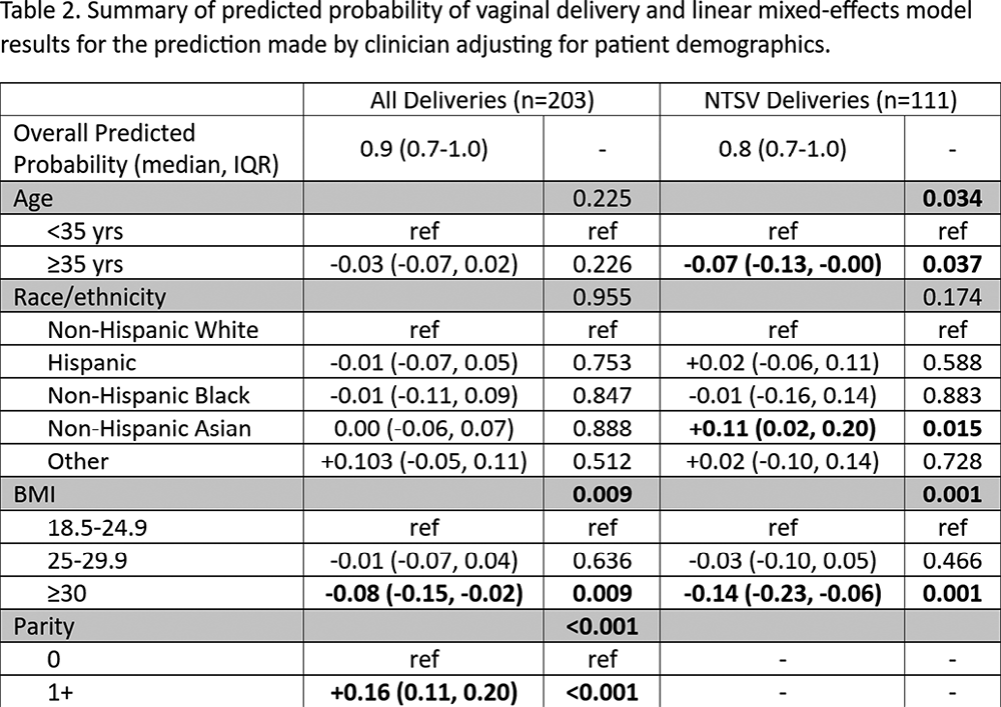

## 1108 | Physician vs. Partometer: a Machine Learning Model Predicts mode of Delivery as well as Physicians

Melissa S. Wong^1^; So Yung Choi^1^; Karla Gonzalez^1^; Samira Torna^1^; Matthew Wells^1^; Alex A. T. Bui^2^; Kimberly D. Gregory^3^

*^1^Cedars‐Sinai Medical Center, Los Angeles, CA; ^2^Medical & Imaging Informatics Group, University of California, Los Angeles, Los Angeles, CA; ^3^Cedars Sinai Medical Center, Los Angeles, CA*

4:00 PM ‐ 6:00 PM

**Objective**: Performance of predictive machine learning (ML) models has rarely been assessed against clinician intuition. The Partometer is a validated ML model which uses data from the electronic health record to generate ongoing predictions of a patient's likelihood of vaginal delivery (VD). The aim of this study is to compare calibration and accuracy of the Partometer in predicting VD compared to the physician.

**Study Design**: This was a prospective study of all patients presenting for a planned VD. At 4 hours, we conducted two concurrent assessments: 1) to the patient's physician “on a scale of 1‐10 (ordinal; 10 = highest), what is your patient's likelihood of a vaginal delivery?”; 2) we queried the Partometer on the same continuous scale.

Patient information was chart abstracted; physician characteristics were obtained from public sources. We assessed calibration by fitting a linear mixed model for difference in Brier scores with random intercept to account for the predictions made by the same physician. For accuracy, we used >6 as predicting a VD (i.e., if the prediction was >6 and a VD occurred, this was a true positive). We stratified by patient characteristics. Subgroup analysis was performed among patients who were nulliparous, term, singleton vertex (NTSV).

**Results**: There were 203 predictions from 46 clinicians. The Partometer was equally well calibrated (Partometer 0.11 +/‐ 0.18, physician 0.12 +/‐ 0.22) and accurate (85.7% [80.1‐90.2] vs. 80.8% [74.7‐86.0]) as physicians in predicting VD. The findings persisted after adjustment for patient and clinician characteristics. On demographic‐adjusted analysis, both the Partometer and physicians were less well calibrated for patients who were non‐Hispanic Black, with obesity, or nulliparous. For the NTSV subgroup, predictions on subjects age ≥35 were less well calibrated by both Partometer and physicians. Differences in calibration were found in the NTSV subgroup among the clinicians < 40 years old and/or < 10 years of practice.

**Conclusion**: ML model to predict mode of delivery was as well calibrated and accurate as physicians’ own predictions of their patients.

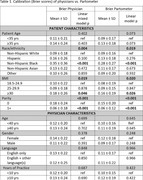

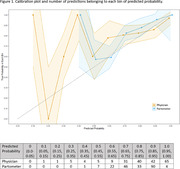

## 1109 | Proinflammatory Omega‐6 and Arachidonic Acid are Increased in Breast Milk of Mothers with Overweight/Obesity

Michael G. Ross; MacKenzie Cervantes; Guang Han; Lihiri Bora; Mina Desai

*The Lundquist Institute at Harbor‐UCLA Medical Center, Torrance, CA*

4:00 PM ‐ 6:00 PM

**Objective**: Human milk composition varies depending on maternal diet, duration of nursing, time of day and maternal body habitus. Among the critical milk components, essential polyunsaturated fatty acids (PUFA omega‐6 and omega‐3) must be supplied to the newborn for brain growth and development. Western diets have led to an increase in maternal consumption of omega‐6 linoleic acid (LA) and a decrease in omega‐3 α‐linolenic acid (ALA) PUFA. Through the elongation and desaturation process, LA is converted to arachidonic acid (AA) and ALA to docosahexaenoic acid (DHA). While AA and DHA are important for brain development, an excessive amount of total omega‐6 (ω6), AA and a higher AA/DHA ratio are associated with accelerated weight gain in infants, increased oxidative stress, inflammation and cognitive impairment. As women with OW/OB have increased total milk fat content, we sought to determine the influence of maternal body mass index (BMI) on specific PUFA levels.

**Study Design**: We studied women who delivered singleton, term neonates and were exclusive breastfeeding at 7‐9 weeks postpartum. Among both normal weight (BMI 18.5‐24.9 kg/m^2^) and women with overweight and obesity (OW/OB; BMI≥25 kg/m^2^), continuous milk samples (10ml) from foremilk to hindmilk were obtained via pump until the breast was emptied. First and last pumped milk samples were analyzed. Milk and maternal plasma lipidomics were undertaken (Lipidyzer Platform).

**Results**: In women with OW/OB, breast milk total ω6, LA and AA levels were significantly increased in both the first (foremilk) and last (hindmilk) samples as compared to normal weight (Fig 1). Importantly, the AA/DHA ratio was significantly increased in foremilk and hindmilk of women with OW/OB (Fig 2).

**Conclusion**: The increased ω6, LA and AA levels including an increased AA/DHA ratio in the breast milk of women with OW/OB has a potential risk for infant obesity, inflammation and adverse cognitive/behavioral outcomes. Novel strategies to optimize milk AA/DHA are urgently needed during pregnancy and lactation, particularly in women with obesity.

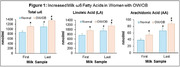

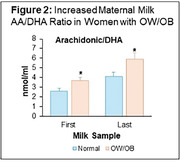

## 1110 | Metformin Inhibits Insulin‐induced Mammary Lipid Synthesis: Development of Personalized Human Milk to Prevent Childhood Obesity

Mina Desai^1^; Guang Han^1^; Krishna Rao^2^; Michael G. Ross^1^

*^1^The Lundquist Institute at Harbor‐UCLA Medical Center, Torrance, CA; ^2^Southern Illinois University School of Medicine, Springfield, IL*

4:00 PM ‐ 6:00 PM

**Objective**: Infants born to obese mothers are at increased risk of childhood obesity due to in‐utero programming and accelerated infant weight gain. The increased fat/calorie content of milk of mothers with obesity suggests that the maternal metabolic milieu, including increased serum insulin,  alters breast lipid synthesis. We utilized a 3‐D immortalized human mammary epithelial cell (MEC) culture to examine the effects of exogenous insulin on lipid synthesis. We further hypothesized that Metformin would prevent insulin‐induced milk lipid synthesis.

**Study Design**: MECs were cultured on cell inserts which separate the basolateral chamber containing “serum” from the apical chamber of milk production. Insulin (5 ng/ml), with and without Metformin (5 µmol/L), was added to the lactogenic medium within the basolateral chamber. After 48h, MECs were analyzed for protein expression (Western Blot) of lipogenic transcription factor (sterol regulatory element‐binding protein, SREBP), and enzymes involved in triglyceride uptake (lipoprotein lipase, LPL) and fatty acid synthesis (fatty acid synthase, FAS). Treated vs. untreated MECs were compared by ANOVA with Dunnett's post‐hoc test. Values are fold change (mean ± SEM) of N = 4 independent cultures.

**Results**: In response to insulin, cellular MECs demonstrated activation of the lipid synthesis pathway as evident by increased protein expression of SREBP1 including its downstream target enzymes LPL and FAS. Metformin treatment normalized the insulin‐induced upregulation of MEC lipid synthesis (Fig 1).

**Conclusion**: The increased fat/caloric content of milk of obese mothers is a result, in part, of insulin‐enhanced mammary lipid synthesis. Consistent with hepatic effects, Metformin reduces insulin‐mediated lipogenesis. These findings suggest that excess human milk lipid synthesis may be normalized by maternal diet or pharmacologic interventions which reduce serum insulin. The extension of personalized human milk from the low birth weight to macrosomic infants may prevent excess infant weight gain resulting from high caloric breast milk.

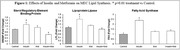

## 1111 | Effect of the COVID‐19 Pandemic on Sterilization Rates based on Location in Wisconsin

Michelle P. Lin^1^; Jacqueline M. Powell^1^; Megan Knutson Sinaise^1^; Janine S. Rhoades^1^; Ronald E. Gangnon^2^; Kara K. Hoppe^1^

*^1^University of Wisconsin School of Medicine and Public Health, Madison, WI; ^2^University of Wisconsin Department of Population Health Sciences, Madison, WI*

4:00 PM ‐ 6:00 PM

**Objective**: The COVID‐19 pandemic placed significant demands on healthcare infrastructure, and medical resources were preferentially allocated to emergent or urgent services. This study aims to examine the effect of the COVID‐19 pandemic on the rate of immediate and 3‐month postpartum (PP) sterilization procedures in Wisconsin (WI) based on geographic location.

**Study Design**: A retrospective cohort of all births from 3/1/2017 to 3/31/2023 in WI was constructed using WI Hospital Association coding data. Pre‐COVID was defined as 3/1/2017 ‐ 2/29/2020 and post‐COVID was defined as 3/1/2020 ‐ 3/31/2023. Generalized additive logistic regression models were constructed for the rate of sterilization at birth and at 3 months PP as a function of geographic location (ZIP code). Statistical analyses were performed using the mgcv package in R.

**Results**: There were 334,408 births – 172,752 pre pandemic and 161,656 during/post pandemic. There were 15,534 sterilization events at the time of birth (4.6%)–8,263 (4.8%) pre pandemic and 7,271 (4.5%) during/post pandemic (RR 0.94, 95% CI 0.91‐0.97, p = 0.0002). There was very strong evidence (p< 0.0001) of geographic variation in the impact of the pandemic on the rate of immediate sterilization. There were nominally significant (p< 0.05) elevations in the rate of immediate sterilization for zip codes in northern and western WI and nominally significant reductions in the rate of immediate sterilization for zip codes in northwest, west central, and south central WI. Similar relationships were seen at 3 months PP.

**Conclusion**: The COVID‐19 pandemic demonstrated a significant decrease in rates of immediate to 3‐month PP sterilization procedures in WI. There was strong evidence of geographic variation in the pandemic's impact on the sterilization rate statewide. The findings of this study indicate a shift in rate of sterilization during a pandemic, which demonstrates the need for development of provisions or alternatives to permanent sterilization to allow for optimal family planning and contraceptive choice for PP patients during times of healthcare crises or when access to care is limited.

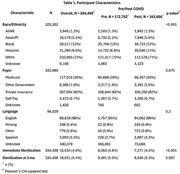

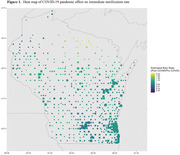

## 1112 | Impact of a Statewide Quality Collaborative on Screening and Referral for Perinatal Substance Use Disorders

Michelle R. Petrich^1^; Alison Williams^2^; Josh Grotzinger^3^; Katie Brassfield^3^; Niraj R. Chavan^4^

*^1^Saint Louis University School of Medicine, Saint Louis University / St. Louis, MO; ^2^Missouri Hospital Association, Missouri Hopsital Association / Jefferson City, MO; ^3^Missouri Hospital Association, Missouri Hospital Association / Jefferson City, MO; ^4^Saint Louis University School of Medicine, St. Louis, MO*

4:00 PM ‐ 6:00 PM

**Objective**: To evaluate the effectiveness of a statewide quality improvement (QI) collaborative for increasing the adoption of Screening, Brief Intervention, and Referral to Treatment (SBIRT) in pregnant persons with substance use disorder (SUD).

**Study Design**: We evaluated the impact of a statewide perinatal collaborative aimed at increasing the adoption of SBIRT through implementation of the Alliance for Innovation in Maternal Health (AIM) patient safety bundle on caring for pregnant and postpartum people with substance use disorder (SUD). We evaluated changes in average rates of SBIRT and bundle implementation through hospital‐level chart reviews, aggregate data submission, structure measure surveys, and engagement metrics over a 2‐year period from January 2022 to December 2023. QI metrics for the collaborative, including process, outcome and structure measures were defined in alignment with the AIM bundle. Hospitals with data from the initial (Jan 1 ‐ Mar 31, 2022) and final (Oct 1–Dec 31, 2023) phase of the project were included. All study variables were coded as nominal data and analyzed using Chi‐square tests; statistical significance was set at p≤.05.

**Results**: Ten hospitals met criteria for inclusion in this QI initiative review and were evaluated for SBIRT adoption before and after implementation of the patient safety bundle. There were no significant differences among sites with regards to geographic location or obstetric bed size. Evaluation of screening patterns demonstrated a significant increase in rates of SUD screening (59.4% to 90.1%, p< .001) after bundle implementation (Figures 1 and 2). Rates of referral for SUD treatment (77.8% to 83.8%, p = .21) and counseling about medications for opioid use disorder (MOUD) (84.5% to 89%, p = .31) were also noted to increase over the 2‐year implementation period, however–this did not reach statistical significance (Figure 1).

**Conclusion**: Adoption of a structured patient safety bundle implemented through a statewide quality collaborative has the potential to increase screening and timely referrals for enhancing care delivery in patients with perinatal SUD.

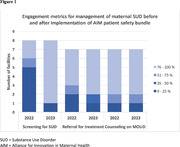

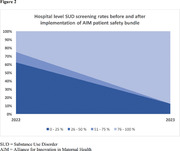

## 1113 | Magnesium Sulfate Infusion During Delivery Hospitalization is not Independently Associated with Postpartum Hemorrhage

Molly McAdow^1^; Jennifer F. Culhane^1^; Kathryn M. McKenney^2^; Moeun Son^3^; Heather Burris^4^; Sara Handley^5^; Jay Greenspan^6^; Kevin Dysart^6^

*^1^Yale School of Medicine, New Haven, CT; ^2^University of Colorado, Denver, CO; ^3^Weill Cornell Medicine, New York, NY; ^4^Children's Hospital of Philadelphia, Philadelphia, PA; ^5^Children's Hospital of Pennsylvania, Philadelphia, PA; ^6^Nemours Children's Hospital, Wilmington, DE*

4:00 PM ‐ 6:00 PM

**Objective**: To determine whether intravenous (IV) magnesium sulfate (Mg) use is associated with postpartum hemorrhage (PPH) and peripartum hysterectomy (hyst).

**Study Design**: This retrospective cross‐sectional study utilizes Epic Systems’ Cosmos research platform, a US‐based electronic health record database with de‐identified patient‐level data. All deliveries from 2017‐2023 without International Classification of Diseases (ICD)‐10 codes for placenta accreta spectrum were included. The exposure was IV Mg use based on the medication administration record. The outcome of PPH was defined by ICD‐10 code plus red blood cell transfusion, procedure code for hemorrhage treatment, or receipt of methergine or carboprost. The outcome of hyst was based on Current Procedural Terminology codes. The associations of IV Mg and the outcomes of (1) PPH and (2) hyst were examined using bivariate and multivariate analyses. In addition, these associations were examined stratified by gestational age at delivery (< 32 vs >32 weeks’ gestation).

**Results**: PPH was significantly associated with all listed covariates in bivariate tests (Table 1). Patients receiving IV Mg were significantly more likely to have PPH [OR 2.55 (95% CI: 2.48‐2.61)], which was slightly attenuated after adjustment [aOR 2.01 (95% CI 1.95‐2.08)]. Among patients with PPH, IV Mg was associated with higher odds of hyst in both models [OR 4.22 (95% CI 3.64‐4.88); aOR 7.72 (95% CI 6.38‐9.30)]. In stratified analyses, after adjustment, patients who delivered >32 weeks and received IV Mg had even higher odds of PPH [aOR 8.40 (95% CI 6.90‐10.21)] (Table 2). There was no significant association among those who delivered < 32 weeks after adjusting for potential confounders.

**Conclusion**: IV Mg use was significantly associated with increased risk of PPH but only among those patients who delivered >32 weeks’ gestation. Since IV Mg is administered primarily for eclampsia risk reduction and not for fetal neuroprotection after this gestational age threshold, our findings suggest that severe preeclampsia may drive the association between Mg and hemorrhage. Further exploration is needed.

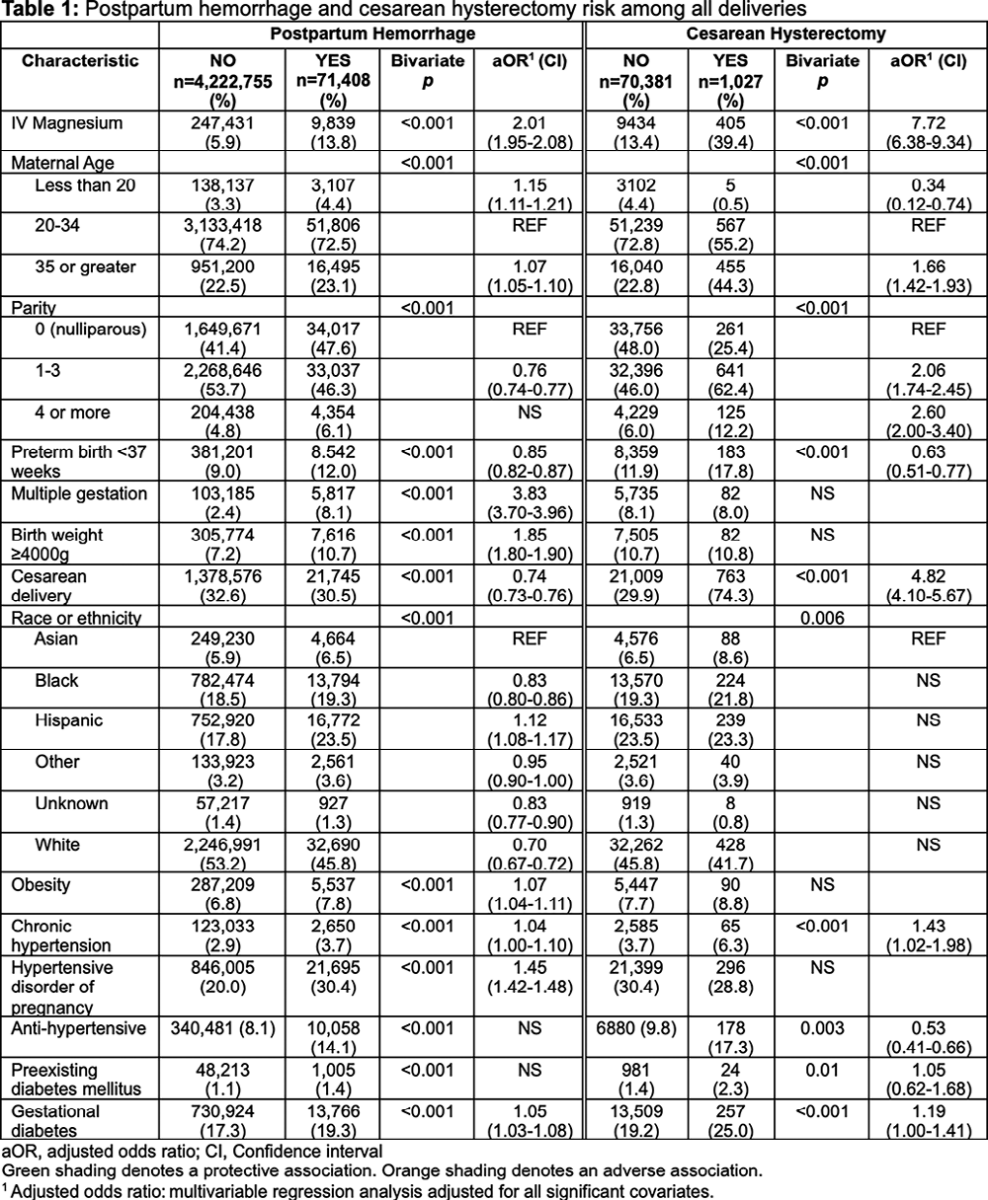

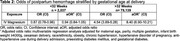

## 1114 | Perinatal Healthcare Inequities: a Causal Network Analysis

Monica A. Lutgendorf^1^; Teri R. Ryals^2^; Erin M. Blevins^3^; Gayle Haischer‐Rollo^1^; Caitlin Drumm^1^; Torie C. Plowden^1^; Carmen N. Spalding^4^; Rasheda J. Vereen^5^; Brelahn J. Wyatt‐Nash^1^; Shara M. Fuller^3^; Megan G. Musilli^3^; Abigail Konopasky^6^

*^1^Uniformed Services University of the Health Sciences, Bethesda, MD; ^2^Naval Hospital Bremerton, Bremerton, WA; ^3^Naval Medical Center San Diego, San Diego, CA; ^4^Sharp Healthcare System, San Diego, CA; ^5^Carl R. Darnell Medical Center, Fort Cavazos, TX; ^6^Geisel School of Medicine at Dartmouth, Hanover, NH*

4:00 PM ‐ 6:00 PM

**Objective**: Persistent healthcare disparities exist within the Military Health System (MHS). The objective of this study was to use qualitative interviews to assess the intersection of operational and cultural experiences of military service with the lived experience of individuals on their use and experience of health care.

**Study Design**: This is an IRB approved qualitative study of the perinatal birthing experience of servicemembers and TRICARE beneficiaries. Thirty semi‐structured interviews were conducted with individuals who delivered an infant within the last five years. Causation coding and deductive methods were used to generate a variable list of antecedent variables, mediating variables and outcomes. Themes were organized into causal chains based on participant stories. A causal network was developed using cross‐case mapping to generate a thematic narrative from a systematic comparison of within‐case causal networks.

**Results**: A complex detailed causal network was developed, depicting structural and social factors affecting birth experiences. Such causal relationships impact individual experiences to varying degrees. Antecedent variables included experiences of dismissal, lack of support, concerns about the pregnancy, knowledge of poor outcomes, family experiences, and systemic issues. Mediating variables included delays in care, lack of command support, fragmented care, microaggressions and fear of medications and interventions. Outcomes included mistrust of the medical system, fear and anxiety, early cessation of human milk feeding and decreased military retention.

**Conclusion**: Comprehensive and logical pathways illustrate challenges faced by birthing individuals in the MHS. Outcomes including mistrust, fear and anxiety and early cessation of human milk feeding are mediated by healthcare experiences and concerns.

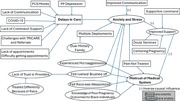

## 1115 | Fertility Treatments Resulting in Twin Pregnancy and the Risk for Childhood inFectious Disease

Moran Shahar^1^; Gil Gutvirtz^2^; Tamar Wainstock^1^; Eyal Sheiner^1^

*^1^Department of Obstetrics and Gynecology, Soroka University Medical Center, Ben‐Gurion University of the Negev, Beer‐Sheva, Israel., Beer Sheva, HaDarom; ^2^Soroka University Medical Center, Metar, HaDarom*

4:00 PM ‐ 6:00 PM

**Objective**: Recent research suggests that singleton pregnancies achieved using fertility treatment are at an increased risk for long‐term infectious morbidity of the offspring as compared with spontaneous singleton pregnancies. Scarce data exists regarding twins. We investigated the risk of long‐term infectious morbidity among twins born following fertility treatments.

**Study Design**: A population‐based retrospective cohort study of all twin deliveries born between the years 1991‐2021 at a tertiary center. Twins born using fertility treatments including ovulation induction (OI) and in‐*vitro* fertilization (IVF) were compared with twins born following spontaneous pregnancies. The long‐term infectious morbidity was compared based on data from community clinics visits and hospitalizations of offspring up to the age of 18 involving an infectious morbidity. The diagnoses of infectious morbidities were based on ICD‐9 codes used in the clinics and hospital wards. A Kaplan–Meier survival curve was used to compare the cumulative incidence of infectious morbidities and a Cox proportional hazards model was constructed to control for confounders.

**Results**: A total of 7790 twins met the inclusion criteria: 2076 twins (26.6%) were born following fertility treatments, among them 696 (8.9%) were born using OI and 1380 (17.7%) following IVF. The total infectious morbidity rate was significantly higher in twins born following fertility treatments as compared with twins from spontaneous pregnancies (73.3% for OI, 71.1% for IVF and 64.1% for spontaneous twins, *p*< 0.001). Likewise, the cumulative incidence of infectious morbidity over time was higher for twins born following fertility treatments (**Figure**). In the Cox regression model, controlling for maternal and gestational age, hypertensive disorders, diabetes mellitus and ethnicity, fertility treatments resulting in twin pregnancy were found to be an independent risk factor for long‐term infectious morbidity of the offspring (**Table**).

**Conclusion**: In our cohort, twins born following fertility treatments were found to be at higher risk for long‐term infectious morbidity.

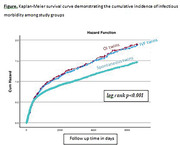

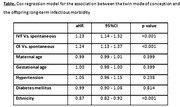

## 1116 | Does Maternal Age at First Pregnancy Affect Long‐Term Endocrine Morbidity of the Mother?

Moran Shahar^1^; Eyal Sheiner^1^; Ruslan Sergienko^2^; Naama Steiner^1^; Tamar Wainstock^1^; Gil Gutvirtz^3^; Roy Kessous^4^

*^1^Department of Obstetrics and Gynecology, Soroka University Medical Center, Ben‐Gurion University of the Negev, Beer‐Sheva, Israel., Beer Sheva, HaDarom; ^2^Ben‐Gurion University of the Negev, Beer Sheva, HaDarom; ^3^Soroka University Medical Center, Metar, HaDarom; ^4^Soroka, Beer‐Sheva, HaDarom*

4:00 PM ‐ 6:00 PM

**Objective**: Pregnancies of women of advanced maternal age are associated with various pregnancy and perinatal complications for the mother and the offspring. However, the long‐term outcomes of the mothers were scarcely investigated. Our aim was to investigate the association between maternal age at first pregnancy and long‐term endocrine morbidity of the mother

**Study Design**: In a population‐based cohort study, long‐term endocrine morbidity of mothers who delivered between 1991‐2021 in a regional tertiary medical center was analyzed. Mothers were stratified by 4 age groups (< 30,30‐35,35‐40, >40). Endocrine morbidity was assessed according to a predefined set of ICD‐9 codes and extracted from endocrine‐related hospitalization files of the mothers. A Kaplan‐Meier survival curve was used to assess cumulative incidence of endocrine morbidity and a Cox proportional hazards model was constructed to control for confounders, comparing the groups of maternal age over 30 to a reference group of women under 30 in their first pregnancy

**Results**: During the study period 73258 women were included, of which 67365 (92.0%) women had their first delivery prior to 30 years of age, 4571 (6.2%) were between the ages 30‐35, 1069 (1.5%) between the ages 35‐40 and 253 (0.3%) were older than 40 years. The total endocrine‐related hospitalization rate increased as maternal age increased among the 4 study groups (15.1%, 15.2%, 17.8% and 18.6%, respectively, *p* = 0.004). Likewise, the cumulative incidence of endocrine morbidity over time was higher for women as they were older in their first pregnancy (**Figure**). The Cox regression model, controlling for fertility treatments and ethnicity, found a ‘dose dependent’ effect as maternal age in the first pregnancy was an independent risk factor for long‐term endocrine morbidity of the mother as compared with the reference group of women under 30 years old (**Table**)

**Conclusion**: As women delay their first pregnancy to an older age, they have higher long‐term endocrine morbidity later in life. The risk tends to increase in a dose‐dependent manner for women over the age of 30 and is highest for women over 40.

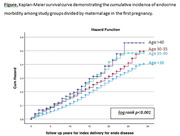

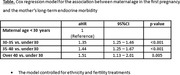

## 1117 | Lactated Ringer's versus Normal Saline for Initial Fluid Resuscitation in Pregnant Patients with Diabetic Ketoacidosis

Morgan A. Scaglione^1^; Melissa J. Cazzell^1^; Margaret Mlynarczyk^2^; Jerri A. Waller^2^; Rebecca Horgan^2^; Alfred Z. Abuhamad^2^; George R. Saade^2^; Marwan Ma'Ayeh^2^

*^1^Macon & Joan Brock Virginia Health Sciences, Eastern Virginia Medical School at Old Dominion University, Norfolk, VA; ^2^Macon & Joan Brock Virginia Health Sciences Eastern Virginia Medical School at Old Dominion University, Norfolk, VA*

4:00 PM ‐ 6:00 PM

**Objective**: Prior studies examining diabetic ketoacidosis (DKA) outside of pregnancy show a shorter time to resolution of the anion gap when utilizing Lactated Ringer's (LR) compared to normal saline (NS) for fluid resuscitation. Our objective was to evaluate this evidence.

**Study Design**: This was a retrospective cohort study of pregnant individuals who present with DKA. The primary outcome was time to anion gap closure. Secondary outcomes included intensive care unit admission, total insulin infusion received, and total crystalloid volume. Continuous variables were tested for normality using the Shapiro‐Wilk test, and compared using Student t‐test or Mann‐Whitney‐U test as appropriate. Categorical variables were compared using Fisher's exact or Chi Squared test.

**Results**: 156 patients were included, 43 (28%) treated with LR and 113 with NS. Baseline characteristics were similar between groups. Median time to anion gap closure was shorter in the LR group (8.8 hours vs 13.2 hours, p = 0.003). Those who received LR also required less insulin from admission to anion gap closure (median 25.4 units vs 41 units, p = 0.005). Volume of crystalloid required to close the anion gap did not differ between groups.

**Conclusion**: The use of lactated ringers as the crystalloid solution with volume resuscitation in DKA is associated with a shorter time to anion gap closure.

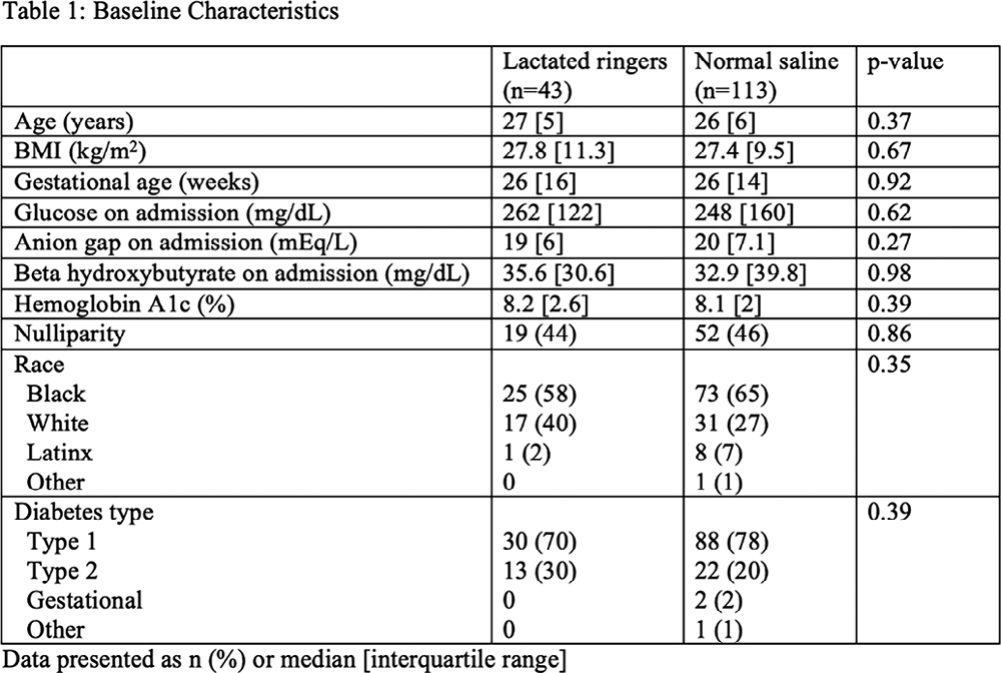

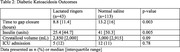

## 1118 | Outcomes of Patients with Euglycemic Versus Hyperglycemic Diabetic Ketoacidosis During Pregnancy

Morgan A. Scaglione^1^; Melissa J. Cazzell^1^; Margaret Mlynarczyk^2^; Jerri A. Waller^2^; Rebecca Horgan^2^; Alfred Z. Abuhamad^2^; George R. Saade^2^; Marwan Ma'Ayeh^2^

*^1^Macon & Joan Brock Virginia Health Sciences, Eastern Virginia Medical School at Old Dominion University, Norfolk, VA; ^2^Macon & Joan Brock Virginia Health Sciences Eastern Virginia Medical School at Old Dominion University, Norfolk, VA*

4:00 PM ‐ 6:00 PM

**Objective**: Euglycemic diabetic ketoacidosis (DKA) is uncommon in the adult non‐pregnant diabetic population, but is more common in pregnancy. Our objective was to evaluate the outcomes of DKA admissions in pregnant patients with euglycemic versus hyperglycemic DKA.

**Study Design**: This was a retrospective cohort study of individuals with diabetes admitted with DKA during pregnancy. They were classified as euglycemic if their admission blood glucose was < 250 mg/dL. The primary outcome was the duration of time between diagnosis of anion gap metabolic acidosis and anion gap closure. Secondary outcomes included admission to the intensive care unit, total insulin received between diagnosis of DKA and anion gap closure, and total crystalloid volume. For continuous variables, normality testing was done using the Shapiro‐Wilk test and data compared using Student t‐test or Mann‐Whitney‐U test as appropriate. Categorical variables were compared using Fisher's exact or Chi Squared test.

**Results**: Euglycemic DKA was present in 78 (50%) of 156 included individuals with DKA. Those with euglycemic DKA had a lower blood glucose, beta‐hydroxybutyrate, hemoglobin A1c, and anion gap compared to those with hyperglycemic DKA. Time between diagnosis of DKA and anion gap closure was longer in those with euglycemic DKA (median 14.8 hours vs 11.6 hours), however volume of crystalloid and amount of insulin did not differ between the groups.

**Conclusion**: Euglycemic DKA is associated with a longer time to anion gap closure despite a lower admission anion gap and beta‐hydroxybutyrate level.

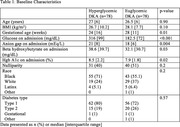

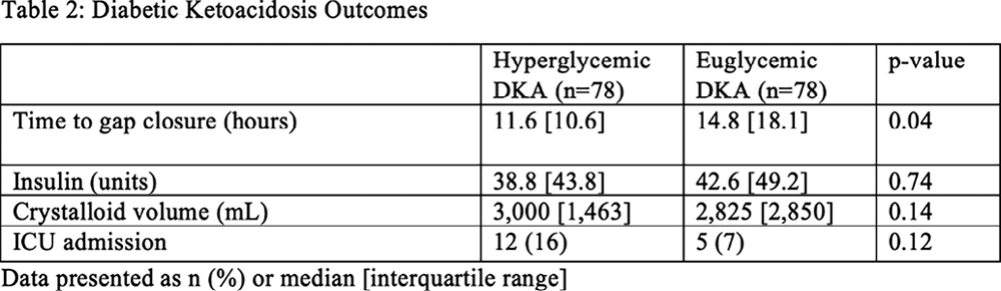

## 1119 | Prospective Validation of USPSTF Guidelines for Preeclampsia Prediction and Clinician Compliance with Aspirin Recommendations

Morten Rasmussen^1^; Arun Jeyabalan^2^; Michal A. Elovitz^3^; Carrie Haverty^4^; Alison Moe^1^; Arkady Khodursky^1^; Maneesh Jain^1^; Manfred Lee^1^; Thomas F. McElrath^5^; Elizabeth F. Sutton^6^; George R. Saade^7^; Antonio F. Saad^8^; Luis D. Pacheco^9^; Joseph R. Biggio, Jr^10^; Ebony B. Carter^11^; Kara M. Rood^12^; Antonina I. Frolova^13^; Esther Park‐Hwang^14^; Cynthia Gyamfi‐Bannerman^15^; Ai‐ris Y. Collier^16^; William A. Grobman^12^; Vincenzo Berghella^17^

*^1^Mirvie Inc., South San Francisco, CA; ^2^University of Pittsburgh School of Medicine, Pittsburgh, PA; ^3^Icahn School of Medicine at Mount Sinai, New York, NY; ^4^Mirvie Inc., South San Fransisco, CA; ^5^Brigham Women's Hospital, Boston, MA; ^6^Woman's Hospital, Baton Rouge, LA; ^7^Macon & Joan Brock Virginia Health Sciences Eastern Virginia Medical School at Old Dominion University, Norfolk, VA; ^8^Inova Health, Falls Church, VA; ^9^University of Texas Medical Branch, Galveston, TX; ^10^Ochsner Health, New Orleans, LA; ^11^University of North Carolina, Chapel Hill, NC; ^12^The Ohio State University, Columbus, OH; ^13^Washington University School of Medicine, St. Louis, MO; ^14^Multicare, Orlando, FL; ^15^University of California, San Diego, San Diego, CA; ^16^Beth Israel Deaconess Medical Center, Boston, MA; ^17^Sidney Kimmel Medical College of Thomas Jefferson University, Philadelphia, PA*

4:00 PM ‐ 6:00 PM

**Objective**: United States Preventive Services Task Force (TF) guidelines recommend using risk‐factors for preeclampsia (PE) in order to offer low‐dose aspirin (LDA) to reduce risk. However, the predictive value is unclear. We sought to prospectively evaluate the performance and utilization of TF in predicting PE and to determine if LDA was recommended based on TF in a diverse population.

**Study Design**: People with a singleton pregnancy enrolled in an IRB‐approved prospective, observational, cohort study < 22 weeks gestation between July 2020 ‐ June 2023. Enrollment occurred at one of 12 clinical centers or via direct‐to‐participant‐recruitment across the United States. Data was obtained from medical records review including TF risk‐factors and if LDA had been recommended (Table 1). All PE cases were adjudicated by trained MFMs.

**Results**: The study enrolled 5,652 diverse participants (Table 1). Using TF recommendations for prediction of PE the sensitivity is 74% and the specificity is 48%. When including one or more moderate risk‐factors the sensitivity increases to 97% and the specificity drops to 12%. 2,440 (43%) received a LDA recommendation. Of those with at least one high‐risk factor, 82% had LDA recommended while in those with one or more moderate risk‐factors, 37.4% received LDA recommendation (Fig 1a). 3,974 (70.3%) were in the TF's moderate risk‐factors only category. For those with any risk factor, LDA was more likely to have been recommended in those who later developed PE vs those who didn't (Fig 1b).

**Conclusion**: TF had poor performance as a predictive screening tool for PE in the group with moderate risk factors. There was also low compliance with LDA recommendations suggesting non‐adherence to TF, practice variation, or incomplete documentation. The higher rate of PE in those recommended LDA may reflect provider identification of a higher risk subgroup, non‐adherence with taking aspirin, or failure of LDA to reduce PE in certain subgroups. These data highlight the opportunity and urgent need to develop better risk assessment tools based on objective biology rather than based on clinical risk factors.

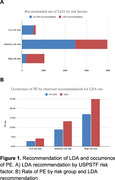

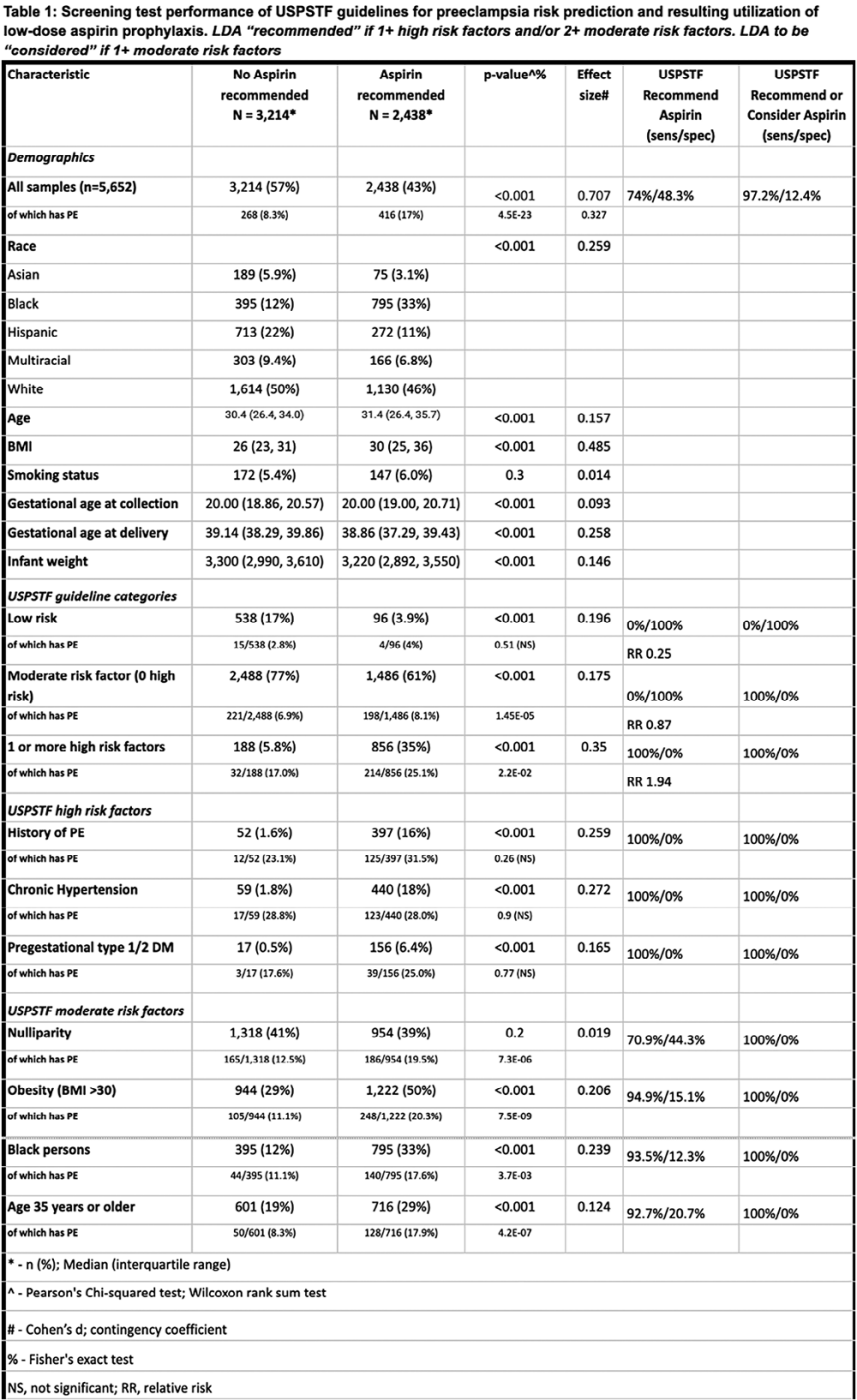

## 1120 | Time Interval from Antenatal Corticosteroid Administration to Delivery and Severe Neonatal Morbidity

Moti Gulersen^1^; Frank I. Jackson^2^; Ashley N. Battarbee^3^; Vincenzo Berghella^1^; Matthew J. Blitz^2^

*^1^Sidney Kimmel Medical College of Thomas Jefferson University, Philadelphia, PA; ^2^Northwell, New Hyde Park, NY; ^3^Center for Women's Reproductive Health, University of Alabama at Birmingham, Birmingham, AL*

4:00 PM ‐ 6:00 PM

**Objective**: To evaluate the association between time interval from ACS administration to delivery and severe neonatal morbidity (SNM) among periviable, early, and late preterm births

**Study Design**: Retrospective cohort of singleton preterm births (PTBs) between 22 0/7‐36 6/7 weeks’ gestational age (GA) at 2 tertiary academic medical centers from 2019‐2022. All patients exposed to ACS prior to delivery were eligible for inclusion. Patients who had neonates that were not actively resuscitated, intrauterine fetal demise, and missing data were excluded. The primary outcome was SNM, a standardized composite neonatal adverse outcome indicator which includes diagnoses and procedures from the neonatal intensive care unit indicative of severe morbidity. Baseline characteristics and outcomes were compared by time interval from the first dose of ACS administration to delivery in 3 groups: < 2, 2‐7, and >7 days. Multivariate logistic regression was performed to evaluate the association between the ACS timing and SNM within planned stratification by GA if there was evidence of interaction. Models were adjusted for potential confounders, and delivery within 2‐7 days was as the reference group. Data were presented as adjusted odds ratios (aOR) with 95% confidence intervals (CI).

**Results**: Of the 2,367 patients included, 518 (21.9%) delivered after 2‐7 days of ACS administration, 1,215 (51.3%) at < 2 days after ACS and 634 (26.8%) at >7 days after ACS. An interaction was noted between GA and time interval from ACS to delivery, where time interval from ACS to delivery was associated with more benefit in reducing SNM at earlier GA (*P* < 0.001). There was no association between ACS timing and SNM in PTBs occurring between 22 0/7‐33 6/7 weeks (Table). Among late PTBs, ACS exposure < 2 days before delivery was associated with an increased risk of SNM (Table).

**Conclusion**: The likelihood of ACS administration occurring within 2‐7 days of PTBs is low. ACS exposure < 2 days before delivery in the late preterm period was associated with an increased risk of SNM. Optimizing timing of ACS in the late preterm period requires further study.

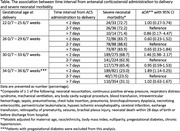

## 1121 | Trends in Previable Antenatal Corticosteroid Administration Among Singletons After Implementation of a Practice Advisory

Moti Gulersen^1^; Erez Lenchner^2^; Marlee Hirsch^3^; Rodney A. McLaren, Jr., Jr.^4^; Huda B. Al‐Kouatly^4^; Vincenzo Berghella^1^; Eran Bornstein^5^

*^1^Sidney Kimmel Medical College of Thomas Jefferson University, Philadelphia, PA; ^2^New York University Rory Meyers College of Nursing, New York, NY; ^3^Northwell Lenox Hill Hospital, Lenox Hill Hospital, NY; ^4^Thomas Jefferson University Hospital, Philadelphia, PA; ^5^Northwell Lenox Hill Hospital, New Hyde Park, NY*

4:00 PM ‐ 6:00 PM

**Objective**: We aimed to evaluate national trends in antenatal corticosteroid administration among previable singletons after implementation of a practice advisory by the American College of Obstetricians and Gynecologists (ACOG) recommending consideration of ACS use as early as 22 0/7 weeks of gestation.

**Study Design**: Retrospective analysis of the United States Centers for Disease Control and Prevention Natality Live Birth Database (2016‐2022). All patients with in‐hospital, non‐anomalous, singleton previable live births (20 0/7‐23 6/7 weeks’ gestation) were eligible for inclusion. Cases where ACS exposure was unknown were excluded. Rates of ACS exposure among all births were compared before and after ACOG's practice advisory published online in September, 2021 using Pearson's chi‐squared test. September to December 2021 was considered an implementation period and therefore excluded from the analysis. Statistical significance was set at *P* < 0.05.

**Results**: A total of 33,570 previable live births comprised the study cohort. There was a significant increase in rates of ACS administration among singletons after ACOG's practice advisory compared to before (1,304/4,827 [27.0%] vs. 6,014/28,743 [20.9%], *P* < 0.001). Rates of ACS exposure stratified by gestational age before and after ACOG's practice advisory are displayed in the Table. Trends in ACS exposure over time among births occurring before and after ACOG's practice advisory is illustrated in the Figure.

**Conclusion**: Data from this large cohort demonstrates the immediate impact ACOG's practice advisory had on administering previable ACS in singleton gestations. A significant increase in ACS exposure occurred at all previable gestational ages. Future research evaluating the impact of this practice change on neonatal outcomes during this time period is needed.

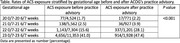

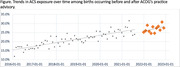

## 1123 | Survey of Individuals with HDP on Cardiovascular Disease Risk Awareness and Prevention Perspectives

Nadine Sunji^1^; Natalia Tovar^1^; Kathryn E. Flynn^1^; Alisse Hauspurg^2^; Anna Palatnik^1^

*^1^Medical College of Wisconsin, Milwaukee, WI; ^2^Alpert Medical School of Brown University, Providence, RI*

4:00 PM ‐ 6:00 PM

**Objective**: To survey individuals with hypertensive disorders of pregnancy (HDP) on their knowledge of cardiovascular disease (CVD) risk and their views on prevention and treatment.

**Study Design**: A 22‐question prospective survey on postpartum HDP management preferences and future cardiovascular risk was administered to postpartum individuals with HDP (gestational hypertension or preeclampsia) during postpartum days 1‐4 before hospital discharge. The survey was validated through six cognitive interviews with HDP individuals.

**Results**: Of 185 individuals with HDP that were approached, 158 (85.4%) completed the survey with their characteristics described in Table 1. Regarding short‐term risks, 57% thought that the risk of developing severe‐range blood pressures (BPs) is the highest during labor admission with 29% estimating the risk to be highest during the first week postpartum. Many individuals were aware of the risk of stroke, severe hypertension, kidney problems, and other CVD during the first 6 weeks postpartum. When asked about treatment for high BPs in the first 6 weeks after delivery, 29% prefer to start with daily exercise, 52% prefer to start with eating healthier with limited salt intake, and only 19% prefer to start with an antihypertensive medication. If exercise and diet were unsuccessful, 61% stated they will consider medications. Table 2 describes participants’ perspectives on facilitators and barriers to starting antihypertensive medications postpartum. 92% reported that doing physical activity regularly and eating healthy may prevent hypertension. 74% thought that eating healthy and engaging in physical activity are easy to do after delivery, and 72% were planning to pursue these activities to lower the risk of hypertension. When asked about future risk of CVD, 40% reported they are well or very well informed on future risk of CVD; however, 74% underestimated the future risk to be ≤10%.

**Conclusion**: Most individuals with HDP recognized short‐term risks and preferred lifestyle changes over medication initially. However, despite 40% feeling well‐informed, many underestimated their long‐term CVD risk.

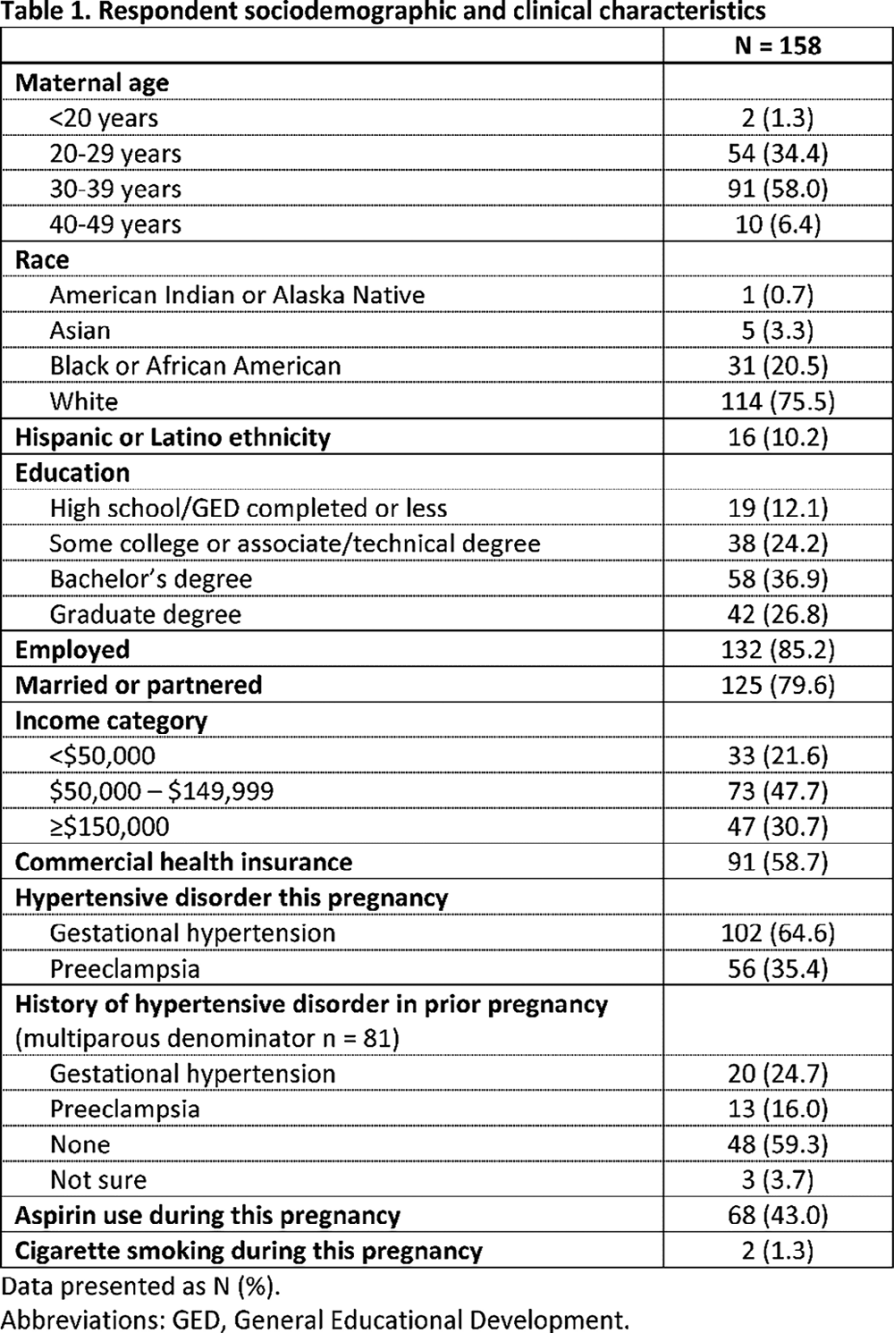

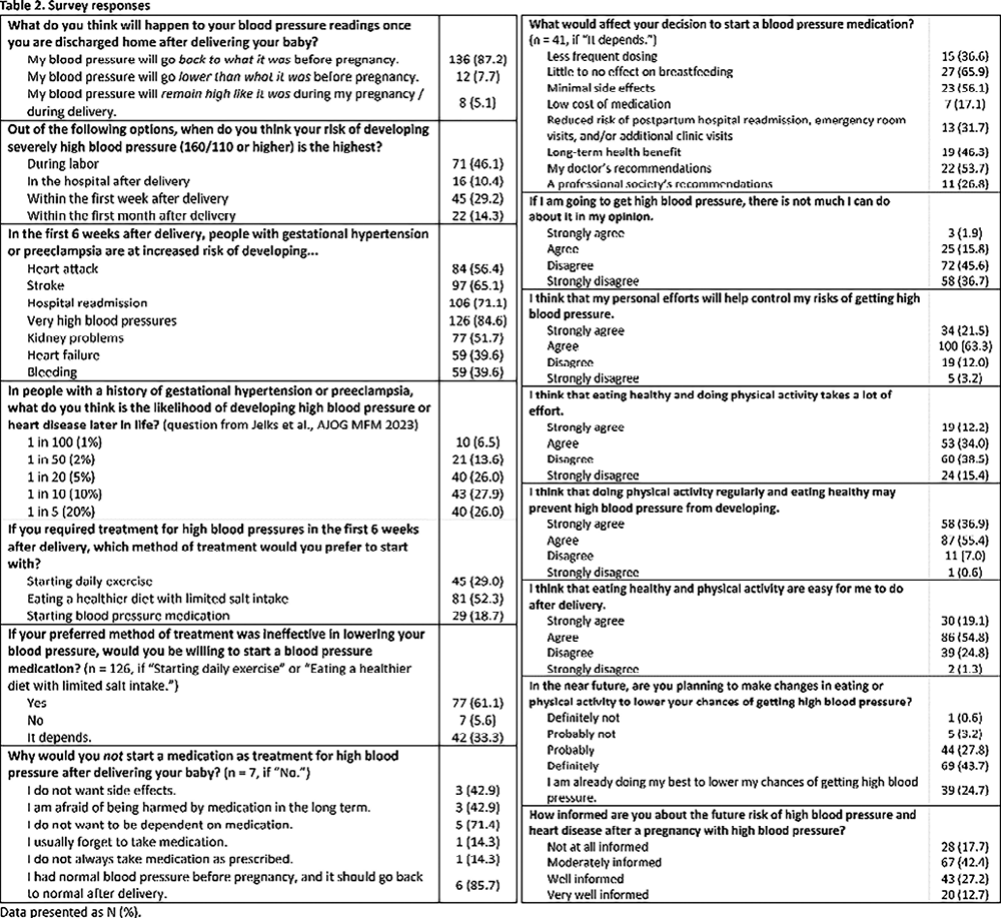

## 1124 | Risk Factors for Dual Demise in Monochorionic Pregnancies Undergoing Radiofrequency Ablation for Discordant Anomalies

Nahla Khalek^1^; Shelly Soni^1^; Juliana S. Gebb^2^; Christina Paidas Teefey^1^; Desiree Fiorentino^1^; Alekhya Jampa^1^; Violetta Bakunina^2^; Julie S. Moldenhauer^1^

*^1^Richard D. Wood Jr. Center for Fetal Diagnosis and Treatment at CHOP, Philadelphia, PA; ^2^Richard D. Wood, Jr Center for Fetal Diagnosis and Treatment, Children's Hospital of Philadelphia, Philadelphia, PA*

4:00 PM ‐ 6:00 PM

**Objective**: To determine the rate and risk factors for dual demise in monochorionic pregnancies with discordant malformation (DM) managed with selective cord occlusion (SCO) via radiofrequency ablation (RFA) of the affected fetus.

**Study Design**: Single center retrospective analysis of a prospective registry of complex monochorionic pregnancies managed with SCO via RFA for DM between 2010 and 2023. DM as indication for procedure was stratified by type of anomaly. Outcomes were analyzed based on the intended outcome of single survivor versus dual demise. Risk factors as defined by DM were compared between groups as well to the cohort of all MC pregnancies undergoing SCO via RFA during the same time period. Mann‐Whitney U test was used for continuous variables and Fisher's exact test was used for categorical variables.

**Results**: A total of 213 pregnancies underwent RFA in the study period; 38 performed for the indication of DM. For the total registry cohort, the incidence of postprocedural dual demise regardless of indication was 23/213 (10.8%). The risk increased more than twofold when analyzed specifically for the indication of DM, 9/38 versus 14/175 when compared to other indications **(23.7% vs 8%; p = 0.009)**. Limb body wall complex (LBWC) yielded the highest risk for dual demise; 3 of 4 cases and 33.3% of all DM with dual demise. The remaining variables analyzed were not significant (Table)

**Conclusion**: Selective cord occlusion via RFA remains a safe and reasonable option in complex MC pregnancies. In the cohort where RFA was indicated for DM, the significantly increased risk of dual demise in cases of LBWC should be reviewed when counseling patients.

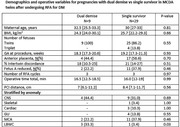

## 1125 | Is the Etiology of Fetal Death Associated with Adverse Health Outcomes During Second‐Trimester Medication Abortion?

Neha Reddy^1^; Vanya Manthena^2^; Jocelyn Wascher^1^; Eryn Wanyonyi^1^; Camille Johnson^1^; Lahari Vuppaladhadiam^3^; Julie Chor^1^; Beth Plunkett^4^; Isa Ryan^5^; Olivert Mbah^5^; Jungeun Lee^6^; Emily Barker^7^; Laura Laursen^8^; Leanne McCloskey^9^; Sloane York^8^; Ashish Premkumar^2^

*^1^University of Chicago Medicine, Chicago, IL; ^2^Pritzker School of Medicine, University of Chicago, Chicago, IL; ^3^University of Chicago, Chicago, IL; ^4^Northshore Endeavor Health, Chicago, IL; ^5^NorthShore Health, Chicago, IL; ^6^Endeavor Health, Evanston, IL; ^7^Washington University in St Louis, St. Louis, MO; ^8^Rush University, Chicago, IL; ^9^Northwestern Medicine, Chicago, IL*

4:00 PM ‐ 6:00 PM

**Objective**: Induction of fetal asystole (IFA) is commonly used before second‐trimester medication abortion (MAB). Data suggest a higher risk for adverse health outcomes when compared to MAB without IFA. Yet there is a poor understanding of whether the etiology of fetal death (IFA or intrauterine fetal demise [IUFD]) is associated with adverse health outcomes.

**Study Design**: We analyzed a retrospective cohort of people undergoing second‐trimester MAB of a singleton at 4 centers from 2009‐2019. Eligible individuals underwent IFA or had IUFD and did not have rupture of membranes, preterm labor, or cervical insufficiency. The primary exposure was etiology of fetal death. Fetal asystole was induced with potassium chloride. The primary outcome was duration of labor. The secondary outcomes were composite morbidity (i.e. uterine rupture, need for blood transfusion, clinical chorioamnionitis, intensive care unit admission, or need or readmission) and its components. Bivariate and multivariate analyses were performed. For the primary outcome, a survival curve was generated, censoring at the time of delivery, and a hazard ratio (HR) was computed. A sensitivity analysis was performed, matching participants based on gestational age and history of uterine scarring.

**Results**: 399 participants were analyzed. 83 (20.8%) underwent IFA and 316 (79.2%) had IUFD (Table). For the primary outcome, IFA was associated with a longer duration of labor, which was nonsignificant after adjusting for race/ethnicity and receipt of mifepristone (Figure). For the secondary outcomes, there was no difference in composite morbidity between the groups. However, IFA was associated with a higher frequency of uterine rupture and need for blood transfusion on bivariate analyses (Table). On sensitivity analysis, there was only a significant difference in frequency of blood transfusion.

**Conclusion**: The etiology of fetal death is not consistently associated with an increased length of labor or increased risk of most adverse health outcomes. IFA may be associated with the need for blood transfusion when compared to IUFD, which requires further investigation.

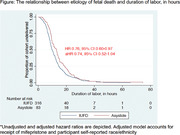

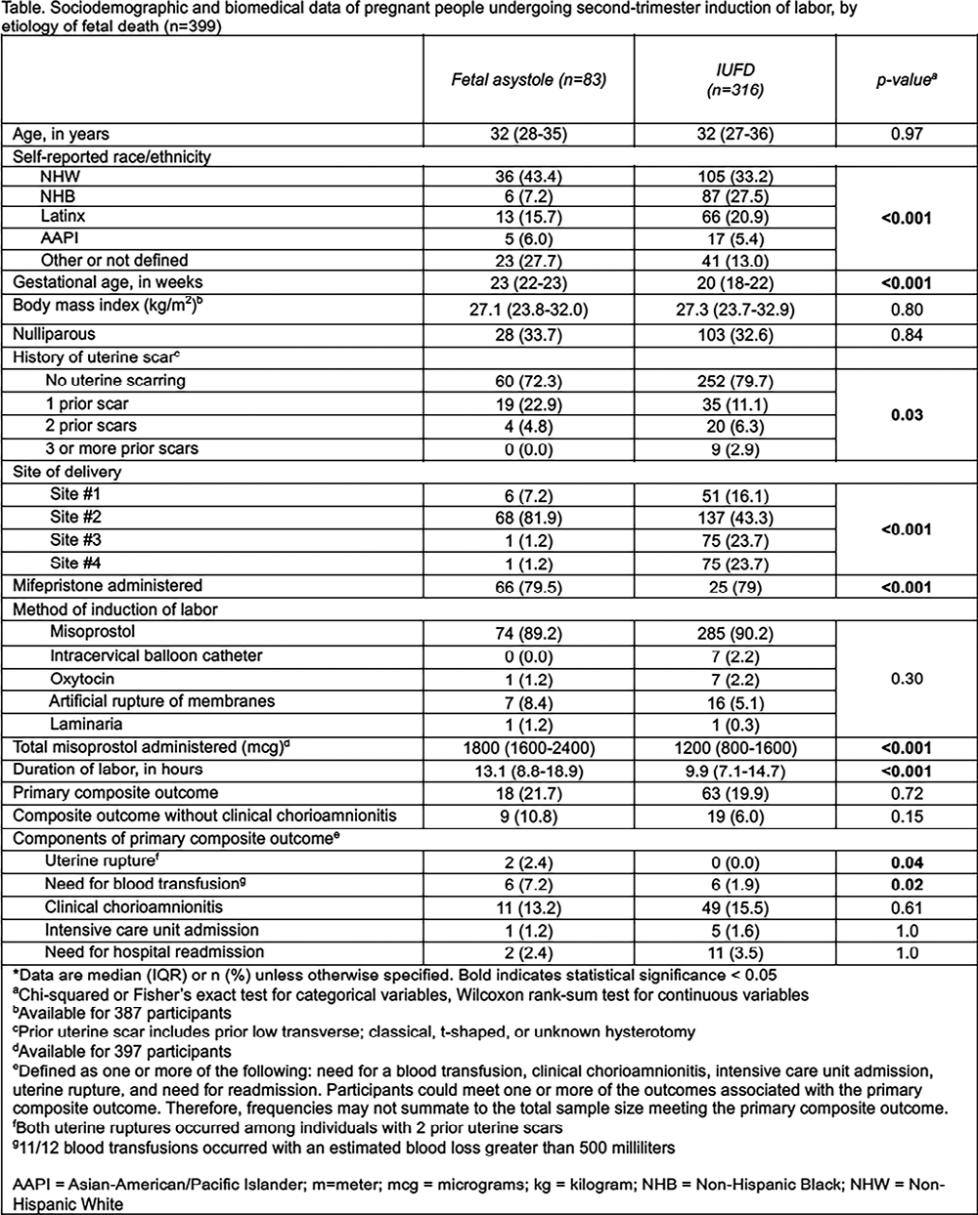

## 1126 | Prescribing less than or greater than 150 MME after Cesarean Delivery: A Cost Effectiveness Analysis

Neha Mishra^1^; Megha Arora^2^; Aaron B. Caughey^2^

*^1^Oregon Health and Science University, Portland, OR; ^2^Oregon Health & Science University, Portland, OR*

4:00 PM ‐ 6:00 PM

**Objective**: Cesarean delivery is the most common major surgery performed in the United States, with an estimated 1,178,066 cesareans performed in 2022 alone. Current standards for postoperative pain management of cesarean deliveries include the use of nonsteroidal anti‐inflammatory drugs (NSAIDs) combined with opioids for breakthrough pain. Despite increased caution around the over‐prescription of opioids, more than 75% of post‐cesarean patients fill an opioid prescription with wide variances in dosing. Previous studies indicate higher rates of adverse outcomes for opioid prescriptions exceeding 150 morphine milligram equivalents(MME). In this study, we evaluated cost‐effectiveness of prescribing greater than or less than 150 MME(e.g. 20 oxycodone tablets, 5 mg each) on developing an opioid use disorder (OUD), overdose, and opioid‐related death.

**Study Design**: We constructed a decision‐analytic model to compare outcomes between receiving greater than 150 MME and less than 150 MME. Our theoretical cohort included 1,178,066 individuals, the number of cesareans performed in 2022. Outcomes were development of an OUD, overdose, opioid use‐related death, costs, and quality adjusted life years (QALYs). We used a willingness‐to‐pay for the incremental cost‐effectiveness ratio of $100,000/QALY. Model inputs were derived from the literature and assessed with sensitivity analyses.

**Results**: In our cohort, prescribing less than 150 MME was associated with 1587 fewer cases of developing an OUD. Prescribing less than 150 MME was cost‐effective with an ICER of $8.94/QALY. In one way sensitivity analysis, receiving greater than 150 MME would only be cost effective if the probability of developing an OUD were below 0.005, above this probability, prescribing less than 150 MME is the only cost effective strategy.

**Conclusion**: In our study, prescribing less than 150 MME was a cost‐effective strategy to improve outcomes and minimize the adverse events associated with opioid prescription. Adopting a standard of prescribing less than 150 MME post‐cesarean delivery would be advantageous for recovery of patients and the health system.

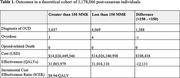

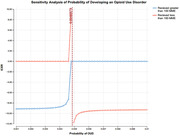

## 1127 | Improved AI Prediction of Preterm Birth by Expanded Training and Retraining

Neil B. Patel^1^; John O'Brien^2^; Robert Bunn^3^; Brandon Schanbacher^2^; John A. Bauer^2^; Garrett K. Lam^2^

*^1^Ascension Sacred Heart ‐ Pensacola, Pensacola, FL; ^2^University of Kentucky, Lexington, KY; ^3^Ultrasound AI, Ultrasound AI, CO*

4:00 PM ‐ 6:00 PM

**Objective**: To assess improvements in Artificial Intelligence (AI) for predicting preterm birth (PTB) after the addition of 1,165,618 images to the training dataset and an 8‐month retraining period.

**Study Design**: Proprietary AI software (V1) was initially trained on 877,141 de‐identified ultrasound images from 19,940 unique ultrasound exams from a cohort of women who delivered at the University of Kentucky from 2017 to 2021. The AI trained on 79% of exams, and predictive performance was validated on a separate 21% of unique exams (n = 4306). Only de‐identified ultrasound images were used to predict the days until delivery. Statistical analyses utilized R^2^ values and mean absolute error (MAE).

An additional 1,165,618 ultrasound images with corresponding patient data from 2021 to 2023 were added to the training set (V2). The AI underwent 8 months of retraining (V3). The original validation set remained blinded and was utilized to evaluate the AI's performance using the original algorithm (V1) and dataset versus its performance in V2 (expanded images dataset) and V3 (expanded images dataset with retraining).

**Results**: After introduction of additional data and retraining, the AI's predictive performance improved across all outcomes (Table 1). The correlation coefficient for spontaneous PTB increased from 0.48 (V1) to 0.64 (V2) and 0.72 (V3). Correlation coefficients for prediction of Term+ all PTB increased from 0.85 (V1) to 0.88 (V2) to 0.92 (V3).

MAE was reduced (Table 2) in all categories from V2 and V3. For Term + all PTB, the MAE reduced from 14.30 days (V2) to 12.90 days (V3). Notable reductions were observed in the MAE for spontaneous PTB (22.08 days (V2) to 19.99 days (V3)), and for iatrogenic PTB (2.44 days (V2) to 19.33 days (V3)).

**Conclusion**: AI's ability to learn and improve its performance in PTB prediction is demonstrated. Expansion of the dataset and retraining sequentially improved its predictive ability and notably reduced the MAE.

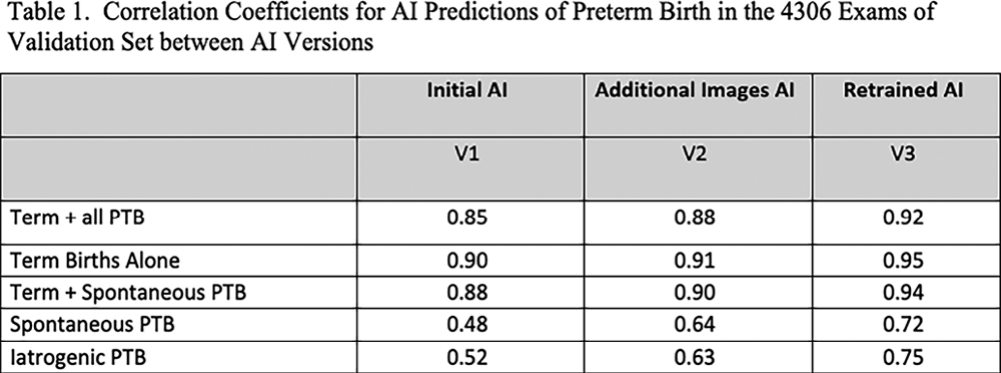

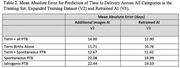

## 1128 | Mid‐Trimester Concentrations of Total Cell‐Free DNA in Amniotic Fluid–A Pilot Study

Dan Tirosh^1^; Amos Douvdevani^2^; Neta Benshalom‐Tirosh^3^; Offer Erez^1^; Joel Baron^1^

*^1^Department of Obstetrics and Gynecology, Soroka University Medical Center, Faculty of Health Sciences, Ben‐Gurion University of the Negev, Beer Sheva, HaDarom; ^2^Department of Clinical Biochemistry and Pharmacology, Faculty of Health Sciences, Soroka University Medical Center, Ben‐Gurion University of the Negev, Beer‐Sheva, HaDarom; ^3^Department of Obstetrics and Gynecology, Samson Assuta Ashdod University Hospital; Faculty of Health Sciences, Ben‐Gurion University of the Negev, Ashdod, HaDarom*

4:00 PM ‐ 6:00 PM

**Objective**: 1) To test the feasibility of measuring total cell‐free DNA (cfDNA) concentration in amniotic fluid, using a unique direct fluorometric assay; and 2) to determine the association between amniotic fluid total cfDNA concentration and gestational age at amniocentesis, establishing reference values for further studies.

**Study Design**: A prospective cohort pilot study was conducted, including women undergoing elective mid‐trimester amniocentesis for genetic or other evaluation, between 16‐23 weeks of gestation. Following written consent, an additional 1‐2 ml of amniotic fluid were collected during amniocentesis. Total cfDNA was quantified by a validated rapid fluorometric assay, using the fluorochrome SYBR Gold without requiring prior DNA extraction and amplification.

**Results**: A total of 82 women were enrolled. Seventy‐seven women were included in the analysis, as five were excluded due to preterm delivery (n = 4) or a genetic disorder (x‐linked ichthyosis, n = 1). All samples had measurable total cfDNA concentrations. Maternal characteristics including indications for amniocentesis are presented in Table 1. Total cfDNA concentrations correlated significantly with gestational age at sample collection (rho = 0.41, p value< 0.001), as presented in Figure 1.

**Conclusion**: 1) Total cfDNA amniotic fluid concentrations in the mid‐trimester, correlate with gestational age at sampling; 2) This simple assay enables rapid quantitative measurements, and thus should be further evaluated as an amniotic fluid biomarker for obstetric complications.

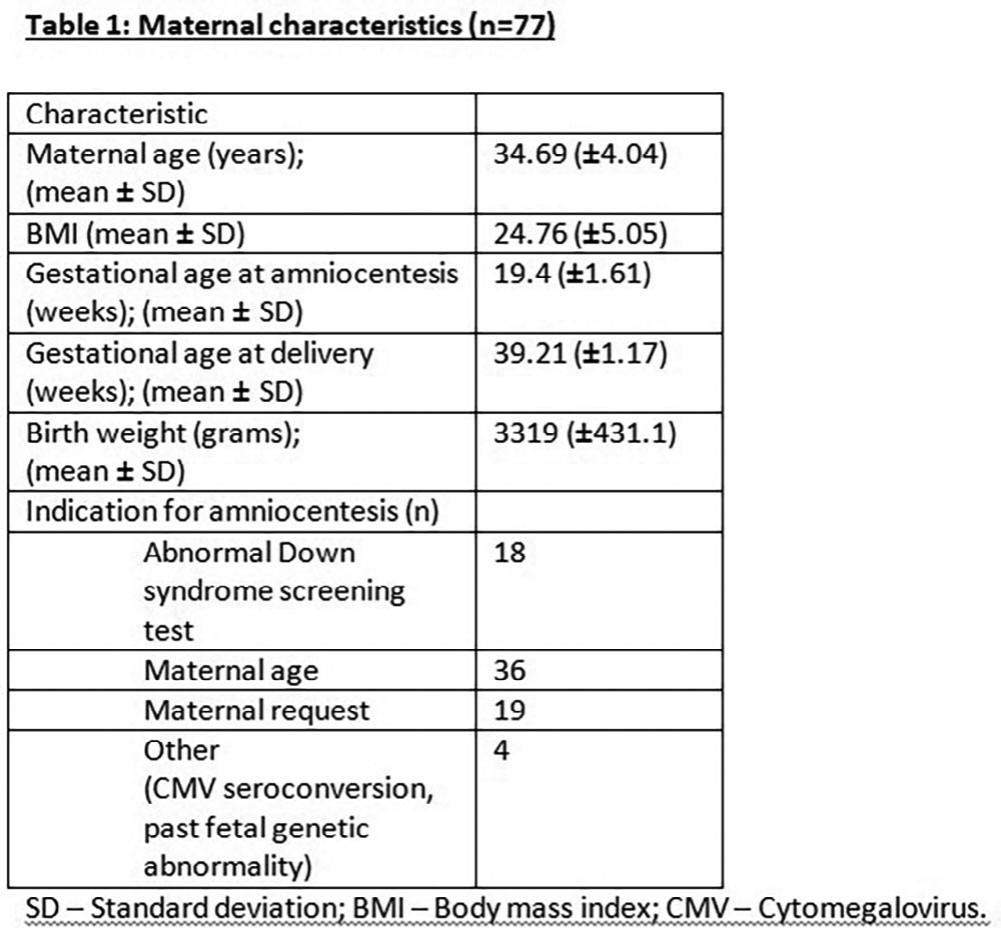

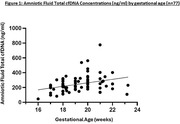

## 1129 | Work‐Related Musculoskeletal Disorders (WRMSD) among Ultrasound Sonographers

Nhu Q. Vu^1^; Ibrahim Tsolakian^2^; Hind N. Moussa^3^

*^1^University of Toledo, Toledo, OH; ^2^Susquehanna OB/GYN and Nurse Midwifery, Bel Air, MD; ^3^ProMedica Toledo Hospital, University of Toledo, Toledo, OH*

4:00 PM ‐ 6:00 PM

**Objective**: Sonographers are at high risk for serious work‐related musculoskeletal injuries. Despite ergonomic solutions, nearly 90% of sonographers experience chronic pain for more than half of their career, and one in five sustain a career‐ending injury. Work‐related musculoskeletal disorders (WRMSD) among sonographers have been an understudied topic. We aim to 1) assess the prevalence of WRMSD among sonographers in our local health system, 2) correlate workplace environment with the development of WRMSD, 3) assess the perception of WRMSD among sonographers, and 4) evaluate the impact of WRMSD on the career of affected sonographers.

**Study Design**: A cross‐sectional study using a 40‐question anonymous survey was electronically distributed to sonographers in our local health system. The survey was developed from previous research and had four main sections 1) demographic information, 2) work schedule and tasks, 3) pain and discomfort, 4) work environment.

**Results**: 91 surveys were distributed with a 45% response rate (n = 41). The majority were age < 29 (34.2%) with a BMI between 18.5‐24.9 (39%). 60.9% scan patients with obesity most of the time. 17.1% gets no breaks during a typical shift and 31.6% disagreed that they can take scheduled breaks. 46.2% reports that pain is most pronounced during work hours, and 81.6% reports that scanning patients with obesity aggravates their pain the most. Of those who sought treatment for their injuries, only 17% reported that the interventions were successful and 59% continues to scan with pain. 100% of respondents are concerned about developing work‐related injury. 84.6% know of other sonographers who have left the field due to their work‐related injury or pain. Most respondents (29.3%) would modify the current equipment design and maneuverability to improve their workplace.

**Conclusion**: Our study shows that sonographers are at an increased risk for WRMSD. These injuries can potentially impact quality of life and can ultimately be career‐ending. More research and advocacy are needed to improve ergonomics and working conditions to help reduce the rate of WRSMD among sonographers.

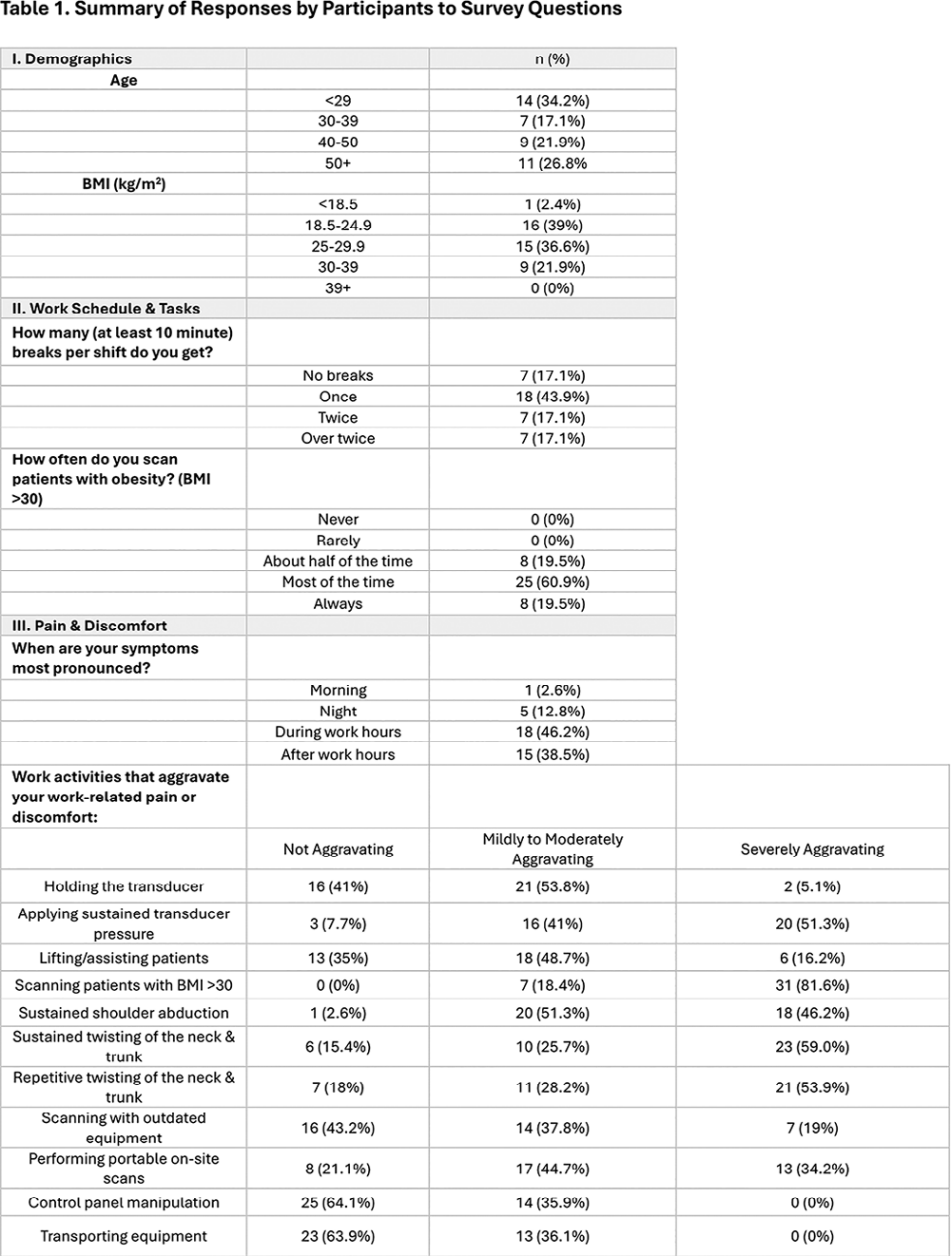

## 1130 | Cesarean Delivery on Maternal Request Among Nulliparous Women: Rates, Timing and 10‐Year Trends

Niall McGowan^1^; Clare Greaney^2^; John J. Morrison^3^

*^1^University of Galway, Galway, Galway; ^2^Galway University Hospital ‐ Ireland, Galway University Hospital ‐ Ireland, Galway; ^3^Dept. of Obstetrics and Gynecology University of Galway ‐ Ireland, University of Galway ‐ Ireland, Galway*

4:00 PM ‐ 6:00 PM

**Objective**: Cesarean delivery on maternal request (CDMR) is defined as caesarean delivery (CD) performed without a clinical reason, i.e. no fetal or maternal indication. It is a controversial area of practice with huge variation in general CDMR rates globally (1,2). However it is difficult to ascertain nulliparous rates resulting in the primary CD. The aims of this study were to determine the following: 1. CDMR rate for nullipara in Irish obstetric practice; 2. Contribution of CMDR to overall CD rates; 3. Associated demographic features and models of obstetric care; 4. Trends in CDMR rates throughout 2014‐2023.

**Study Design**: The data were obtained from EuroKing Obstetric Database at Galway University Hospital, a tertiary referral center in the West of Ireland. The information had been entered prospectively during 2014‐2023. Descriptive statistics were calculated for each year, and compared using Chi2 for proportions and trend, using the program GraphPad Prism v10.2.3. Ethical Committee approval for the study was obtained.

**Results**: There were 28,264 deliveries of which 10,259(36.3%) were nullipara with singleton pregnancies ^3^ 36 weeks. There were n = 134 CDs performed solely for maternal request which represented 1.3% of all nullipara. CDMR contributed to 3.8% of all nulliparous CDs, and constituted 1.4% of all hospital CDs. The maternal age, BMI, and birthweight characteristics of these pregnancies are shown in Table 1. The prevalence of CDMR among nullipara attending a private obstetrician for care was 5.9% and was 0.7% (P < 0.0001) for women attending for public hospital care. The CDMR rate among nullipara increased significantly over the duration of the study from 0.9% in 2014 to 2.7% in 2023 (P < 0.0001), Figure 1.

**Conclusion**: These findings describe the prevalence of CDMR among nulliparous women and the subsequent contribution to overall CD rates. A significant increase in rates was observed in the last decade. The rates of CDMR were higher among women attending a private obstetric service.

References

1. Jenabi E et al. J Mat‐Fetal & Neo Med 2020; 33: No.22, 3867‐3872

2. Begum T et al. BJOG 2020; 128:798‐806

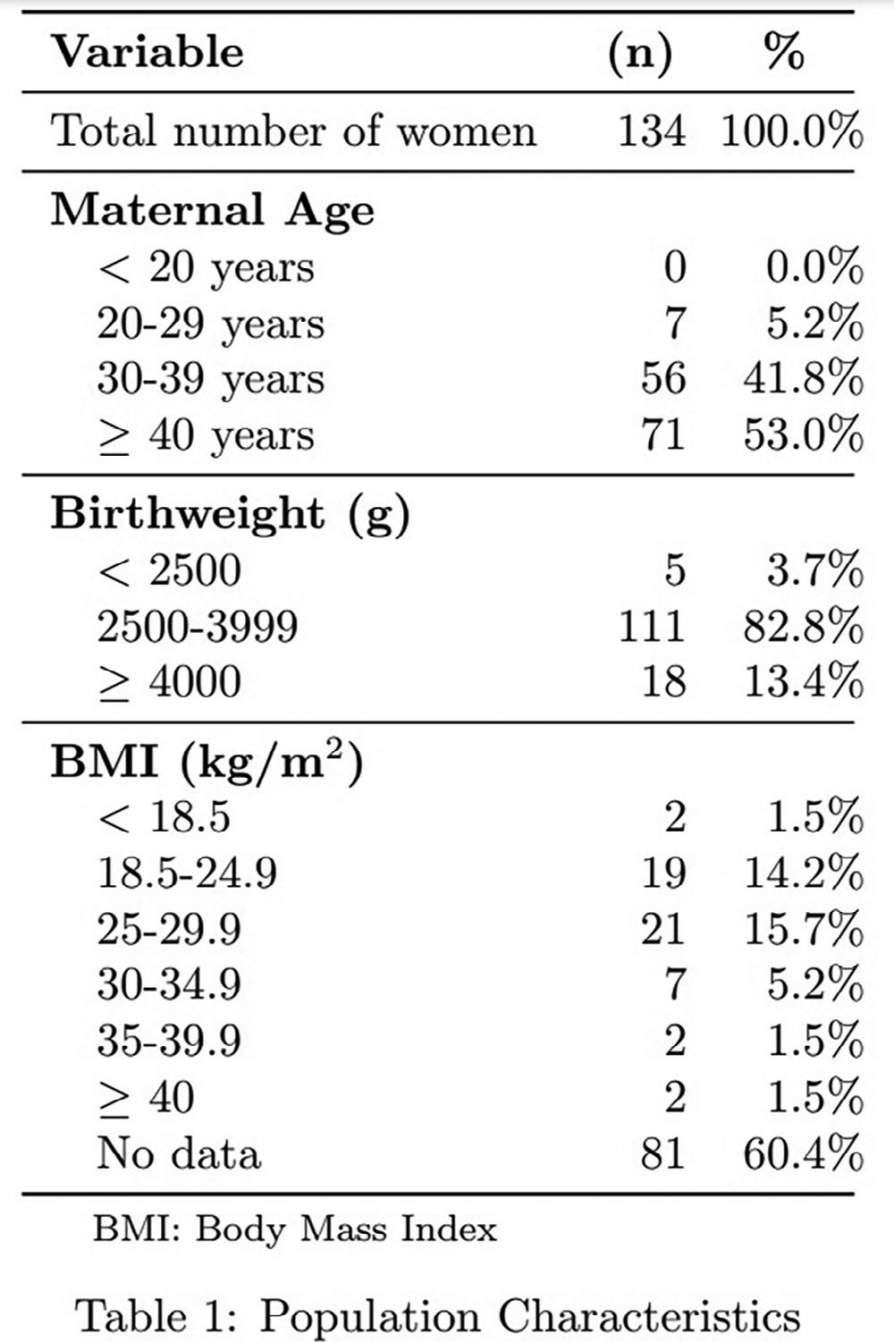

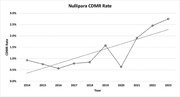

## 1131 | Categorizing the cause of Maternal Instability with MEOWS

Nicholas W. Racchi^1^; Kourosh Kiyani^2^; William Schnettler^3^

*^1^Trihealth, Cincinnati, OH; ^2^TriHealth, Cincinnati, OH; ^3^TriHealth, Cincinatti, OH*

4:00 PM ‐ 6:00 PM

**Objective**: To investigate the ability of a customized MEOWS (Modified Early Obstetric Warning System) calculator to categorize the cause of maternal critical illness.

**Study Design**: This retrospective self‐controlled cohort study evaluated all pregnant people admitted to an intensive care unit at two separate delivering hospitals in Cincinnati, Ohio, between January 1, 2018, and August 1, 2023. Inclusion required study participants to be at least 20 weeks’ gestation through 6 weeks postpartum. Vital signs obtained within the 4 hours prior to the decision for ICU admission were compared to participants’ prior vitals from earlier triage visits. These values were then put into a customized MEOWS calculator (Figure 1) that incorporated additional history‐based questions to categorize the patient into a MEOWS subset (stable, hypertensive emergency, hemorrhage, cardiac/possible peripartum cardiomyopathy, sepsis, trauma, or not otherwise classified). The calculated MEOWS category was then compared to the final discharge diagnosis to determine accuracy using Exact Methods.

**Results**: Vital signs from 87 patients were obtained and used in the modified MEOWS calculator. Our modified MEOWS calculator could predict maternal instability with a sensitivity of 93.3% and a specificity of 83.3%. The calculator was able to correctly categorize the cause of maternal morbidity with a sensitivity of 89.8% and a specificity of 67.8%. The individual sensitivities and specificities for the individual categories of the cause of maternal morbidity can be seen in table 1.

**Conclusion**: The use of a modified MEOWS calculator can better help determine the cause of maternal instability. This type of modified MEOWS calculator could better help categorize the cause maternal instability and thus allow for the effective use of an appropriate clinical protocol.

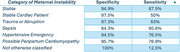

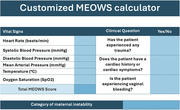

## 1132 | Validating the Use of Epigenetic Clocks in a Preconception Population

Nicola C. Perlman^1^; Jiaqi Zhang^1^; Andrea Cipriano^2^; Stephanie A. Leonard^1^; Xixi D. Plummer^1^; Samantha L. Kruger^1^; Arian Korshid^2^; Janet Hurtado^1^; Vittorio Sebastiano^2^; Danielle M. Panelli^1^; Ruth B. Lathi^1^; Katherine Bianco^1^

*^1^Stanford University, Palo Alto, CA; ^2^Stanford University, Stanford, CA*

4:00 PM ‐ 6:00 PM

**Objective**: Prior studies have attempted to use epigenetic clocks to correlate biologic age with pregnancy outcomes. Despite this, no epigenetic clock has been validated for use in pregnancy, and biologic age outputs can vary in different tissues and populations. This study assessed the validity of 9 epigenetic clocks in a preconception cohort.

**Study Design**: This study utilized biobanked samples from preconception patients between 2020‐2023. Peripheral blood mononuclear cell DNA was extracted from buffy coat, and DNA methylation was profiled using Illumina Infinium MethylationEPIC BeadChip array, allowing prediction of biologic age via 9 epigenetic clocks. Pearson correlation coefficients were calculated for biologic age and chronologic age (defined as age by birthdate) for each clock. An epigenetic clock was defined as valid if the correlation coefficient (r) was >0.800, in accordance with prior literature.

**Results**: 68 blood samples from people assigned female at birth were analyzed. The median chronologic age was 38.0 years [Q1‐Q3 33.0‐40.3]. No sample had the same biologic age in all 9 clocks. Of the epigenetic clocks evaluated, the correlation coefficient ranged from 0.673 to 0.916 (Table 1). Three clocks exceeded the preset validity threshold of r >0.800; the SkinBlood Clock (r = 0.916), the Zhang Clock (r = 0.915), and the Hannum Clock (r = 0.822). Each of the top 3 performing clocks had a different range of biologic age outputs (Figure 2). Although the Zhang and the SkinBlood Clock had similar correlation coefficients, the SkinBlood Clock produced biologic ages more akin to chronologic ages (median 35.8 years [32.6‐39.2]) compared to the Zhang Clock (56.02 years [54.39‐56.81]).

**Conclusion**: In this validation study we considered 9 different clocks for use in a preconception cohort. The SkinBlood Clock performed best with high correlation and chronologic age congruency. Epigenetic clocks are an exciting new technology for studying aging. Research using epigenetic clocks in pregnancy and fertility should assess validity prior to testing for association with perinatal outcomes.

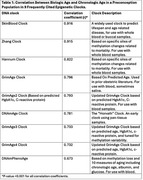

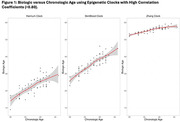

## 1133 | The Association of Preconception Epigenetic Changes of Aging with Live Birth and Pregnancy Outcomes

Nicola C. Perlman^1^; Jiaqi Zhang^1^; Andrea Cipriano^2^; Stephanie A. Leonard^1^; Xixi D. Plummer^1^; Samantha L. Kruger^1^; Arian Korshid^2^; Janet Hurtado^1^; Vittorio Sebastiano^2^; Danielle M. Panelli^1^; Katherine Bianco^1^

*^1^Stanford University, Palo Alto, CA; ^2^Stanford University, Stanford, CA*

4:00 PM ‐ 6:00 PM

**Objective**: Biologic age from epigenetic clocks has been used to predict diseases of aging such as cardiac disease, however its association with fertility and pregnancy complications is not known. We examined the relationship between biologic age and both live birth and pregnancy complications.

**Study Design**: This study analyzed biobanked serum samples of people desiring pregnancy from 2020‐2023. Pregnancy outcomes were followed for 1‐4 years after sample collection. Peripheral blood mononuclear cell DNA was extracted and >850,000 CpG methylation loci were interrogated. This allowed prediction of biologic age (bio‐age) using the SkinBlood Clock, which had the highest validity in the cohort. The difference between chronological age (chrono‐age, measured from birthdate) and bio‐age was calculated (chrono‐bio age difference); decelerated aging was defined if bio‐age was less than chrono‐age. A multivariable logistic regression was performed to test the association between the chrono‐bio age difference and (1) live birth in the entire cohort, and (2) a composite of pregnancy complications related to aging (miscarriage/fetal demise, preterm labor/delivery, hypertensive disorders, gestational diabetes, or placental insufficiency) in a sub‐cohort which achieved pregnancy.

**Results**: Among 64 patients, the median chrono‐age was 38.0 years (Q1‐Q3 34.8‐41.0), and bio‐age was 35.8 (32.6‐39.2). 46 patients achieved pregnancy; 38 (83%) had a live birth, and 23 (50%) had at least one pregnancy complication. Bio‐ages differed between people with versus without a live birth (Table 1). When controlled for confounders, for every year that bio‐age was less than chrono‐age, there was a two‐fold increased odds of live birth (aOR: 2.02; 1.20‐4.11, Table2). There was no significant association between the chrono‐bio age difference and the composite outcome of pregnancy complications.

**Conclusion**: In this study, decelerated biologic aging was associated with higher rate of live birth. Robust studies are needed to understand ways to reverse biologic aging and use it to predict and optimize perinatal outcomes.

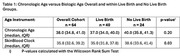

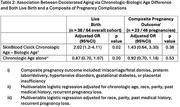

## 1134 | Safety and Efficacy of Influenza Vaccination in Pregnancy: Umbrella Review and Meta‐Analyses

Nikan Zargarzadeh^1^; Giulia Bonanni^2^; Mohammad Haddadi^3^; Ali Javinani^1^; James Versalovic^4^; Kjersti M. Aagaard^5^; Stefania Papatheodorou^6^; Alireza A. Shamshirsaz^7^

*^1^Boston Children's Hospital, Harvard Medical School, Boston, MA; ^2^Postdoctoral Research Fellow Fetal Care and Surgery Center Division of Fetal Medicine and Surgery Boston Children's Hospital, Harvard Medical School, Boston, MA; ^3^Vali‐E‐Asr Reproductive Health Research Center, Family Health Research Institute, Tehran University of Medical Sciences, Tehran, Iran, Tehran, Tehran; ^4^Department of Pathology & Immunology, Baylor College of Medicine, Houston, TX, USA Department of Pathology, Texas Children's Hospital, Houston, TX; ^5^Boston Children's Hospital, Division of Fetal Medicine and Surgery, Boston, MA; HCA Healthcare and HCA Healthcare Research Institute, Nashville, TN; HCA Texas Maternal Fetal Medicine, Houston, TX; Baylor College of Medicine and Texas Children's Hospital, Houston, TX, Boston, MA; ^6^Department of Epidemiology, Harvard T.H. Chan School of Public Health, Harvard University, Boston, MA, USA Department of Biostatistics and Epidemiology, Rutgers School of Public Health, New Brunswick, NJ, USA, New Brunswick, NJ; ^7^Fetal Surgeon Chief, Division of Fetal Medicine and Surgery Director of the Maternal Fetal Care Center Director of the Perinatal Surgery Fellowship Professor of Surgery, Boston Children's Hospital Harvard Medical School, Boston, MA*

4:00 PM ‐ 6:00 PM

**Objective**: Although influenza vaccination during pregnancy is known to prevent seasonal flu outbreaks, reliable and contemporary quantitation of benefits and safety has not been performed. The aim of the current umbrella review to synthesize evidence from multiple meta‐analyses was to reliably and robustly provide quantitative measures of the effectiveness and safety of influenza vaccines among gravidae.

**Study Design**: We conducted a systematic search of PubMed, Scopus, Web of Science, and Embase from inception to 09/13/2023, including over 100 individual studies retaining 38 million pregnancies. Data were extracted from multiple meta‐analyses, and included studies were filtered to ensure no duplication within outcomes. Risk ratios (RRs) and pooled proportions were calculated using R, with aggregate effect sizes and random‐effects models applied. Egger's test was used to assess publication bias. PROSPERO registration: CRD42024519189.

**Results**: As shown in Figure & Table, influenza vaccination during pregnancy resulted in a significant risk reduction of fetal death or stillbirth (RR 0.83 & 0.77, 95% CI 0.78–0.89 and 0.69–0.87), PTB (RR 0.92, 95% CI 0.88–0.96), and LBW neonates (RR 0.88, 95% CI 0.84‐0.93). Reflective of changes in co‐morbidities and other respiratory illness pandemics over the study interval, there was heterogeneity in some outcomes, such as PTB (I^2^: 63.06%), while other outcomes showed low to moderate heterogeneity. Egger's test for publication bias indicated no significant bias across the studies.

**Conclusion**: Influenza vaccination during pregnancy is associated with significant and quantifiable reductions in the risk ratio of multiple adverse perinatal outcomes. Providing these quantitative estimates may aid in public health messaging and shared decision‐making with patients and their families.

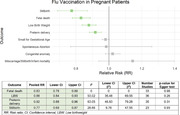

## 1135 | Risk of Hypertensive Disorders of Pregnancy in GLP‐1 Agonist Exposed and Unexposed Patients

Nishita Pondugula^1^; Jennifer F. Culhane^1^; Lisbet S. Lundsberg^1^; Audrey A. Merriam^2^; Caitlin Partridge^1^

*^1^Yale School of Medicine, New Haven, CT; ^2^Yale New Haven Hospital, New Haven, CT*

4:00 PM ‐ 6:00 PM

**Objective**: To understand the effect of GLP‐1 agonist use in the year prior to conception on risk of developing hypertensive disorders of pregnancy (HDP).

**Study Design**: All patients delivering between 2014‐2024 with evidence of GLP‐1 exposure in the year prior to conception were identified through electronic medical record (EMR) query with hand review to confirm exposure. GLP‐1 exposed patients were classified according to GLP‐1 indication for use: 1) pregestational diabetes mellitus (PGDM) or 2) weight management (WM) for increased body mass index (BMI). Control groups for each indication cohort were identified from 2021‐2022 delivery EMR data. PGDM controls were patients with pregestational DM1 or DM2 managed with medication other than GLP‐1 agonist in the year prior to conception. WM controls were a stratified random sample of patients without PGDM not on antihyperglycemic medication in the year prior to conception with BMIs between 30 < 40 kg/m^2^ (n = 100) and ≥40 kg/m^2^ (n = 100). Demographic and clinical characteristics and obstetrical outcomes were compared. Logistic regression was employed to assess the crude (OR) and adjusted odds ratios (aOR) for HDP controlling for covariates significant in bivariate tests at < 0.05.

**Results**: 246 patients had GLP‐1 exposure in the year prior to conception. Of these, 104 were exposed for PGDM and 142 for WM. For the PGDM cohort, 175 controls were identified from the two years of EMR data interrogated. For the WM cohort, random sampling yielded 200 controls.

Bivariate tests for race/ethnicity, pre‐pregnancy BMI, and chronic hypertension identified significant differences between exposed and unexposed patients in the PGDM cohort. For the WM cohort, exposed and unexposed patients had significant differences in PCOS diagnosis, gestational weight gain, and HDP. For both the PGDM and WM cohort, exposed patients were half as likely to develop HDP compared to unexposed counterparts (PGDM aOR = 0.47 (0.27‐0.83); WM aOR = 0.48 (0.29‐0.80)).

**Conclusion**: GLP‐1 use in the year prior to conception significantly reduced the likelihood of developing HDP for patients with PGDM and undergoing WM.

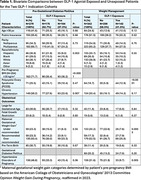

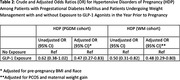

## 1136 | Early Pregnancy Cortisol Levels in Women with and without a History of Preterm Deliveries

Noam Tomasis Damri^1^; Eyal Sheiner^2^; Ifat Baram Goldberg^3^; Maayan Hagbi Bal^1^; Doron Bergman^1^; Ron Rosenbaum^1^; Ayal Haimov^4^; Tamar Wainstock^2^

*^1^Ben Gurion University of the Negev, Beer Sheva, HaDarom; ^2^Department of Obstetrics and Gynecology, Soroka University Medical Center, Ben‐Gurion University of the Negev, Beer‐Sheva, Israel., Beer Sheva, HaDarom; ^3^Ben Gurion University Of The Negev, beer sheva, HaDarom; ^4^Ben Gurion University of the Negev, Ramat Gan, HaMerkaz*

4:00 PM ‐ 6:00 PM

**Objective**: Elevated cortisol levels during pregnancy have been associated with an increased risk for preterm delivery (PTD). However, the precise nature of this relationship and its potential mechanisms remain poorly understood, particularly concerning cortisol levels in early pregnancy. The present prospective study was designed to investigate the association between early pregnancy cortisol levels and perceived stress, and adverse pregnancy outcomes in women with and without a history of PTD.

**Study Design**: In this prospective study, women with or without a history of PTD were recruited during 11‐13 weeks of gestation at a tertiary medical center. All participants provided a saliva sample and answered a health and stress questionnaire. Pregnancy outcomes were compared between women with high and low salivary cortisol levels. Mean salivary cortisol levels were also compared between women with and without a history of PTD and between women with and without selected pregnancy complications.

**Results**: The study included 375 women, out of them 194 with, and 181 without previous PTD. The levels of cortisol were higher among women with vs. without PTD (0.1440 ug/dL ± 0.095 and 0.297 ug/dL ± 0.23, respectively, p‐value< 0.001) in total, and when comparing the groups at each gestational week (Figure 1). Women who had high cortisol levels were not at increased risk for adverse pregnancy outcomes including PTD (Table 1). No association was found between the perceived stress based on the questionnaire and the measured cortisol levels.

**Conclusion**: Although cortisol levels were higher among women with previous PTD, cortisol levels were not associated with the risk for PTD and other adverse pregnancy outcomes, possibly due to the early gestational age at which the cortisol was measured. Further study on early pregnancy markers for potential complications is needed to facilitate effective intervention and prevention.

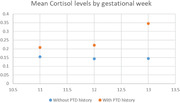

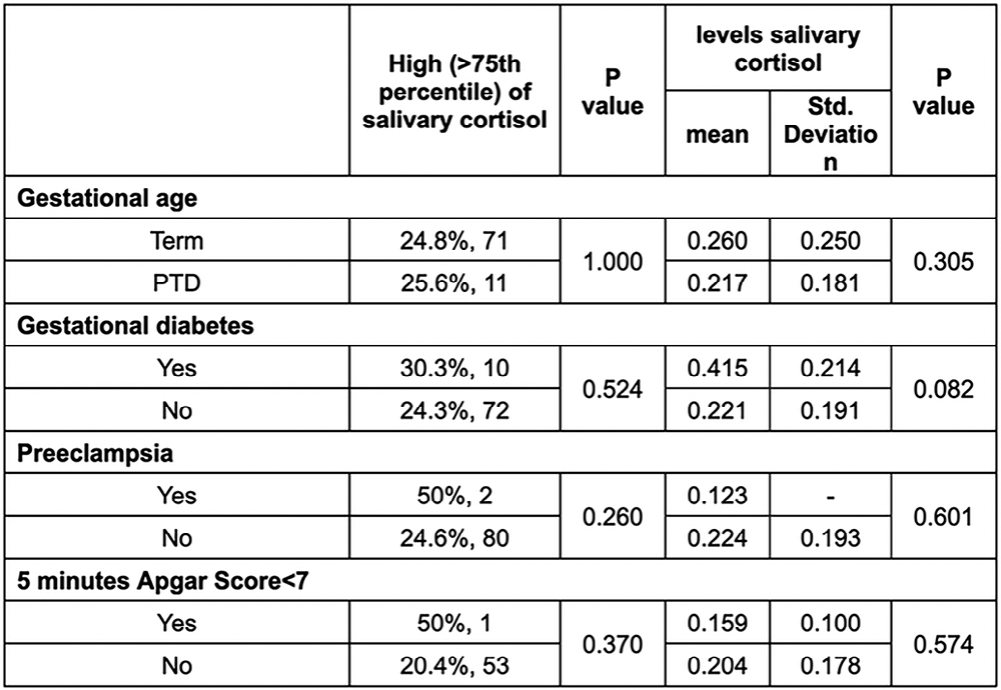

## 1137 | Brain Imaging for Evaluation of Severe Headache in Pregnancy

Noam Pardo^1^; Zborovsky Ilanit^2^; Yahel Ben Shimol^2^; Anat Pardo^3^; Keren Zloto^4^; Noa Gonen^5^; Halit Kantor^6^; Rakefet Yoeli‐Ullman^4^; Shali Mazaki‐Tovi^7^; Michal Fishel Bartal^8^

*^1^Maternal Fetal Medicine Division, Sheba Medical Center, Tel Hashomer, Sheba Medical Center/Ramat Gan, HaMerkaz; ^2^Sheba Medical Center, Tel Hashomer, Sheba Medical Center/Ramat Gan, HaMerkaz; ^3^The Helen Schneider Hospital for Women at Rabin Medical Center, Petach Tikwa, HaMerkaz; ^4^Sheba Medical Center, Ramat Gan, HaMerkaz; ^5^Sheba Medical Center, Ramat gan, HaMerkaz; ^6^University Of Haifa, University Of Haifa/Haifa, HaZafon; ^7^Department of Obstetrics and Gynecology, Sheba Medical Center, Tel HaShomer, Ramat Gan, HaMerkaz; ^8^UTH Houston & Sheba Medical Center Israel, Houston, TX*

4:00 PM ‐ 6:00 PM

**Objective**: Headache is the most common neurologic symptom in pregnancy. There is a debate on the necessity and imaging indications for headache evaluation. We aimed to assess the effectiveness of brain imaging for persistent severe headaches in pregnant individuals.

**Study Design**: This retrospective study included pregnant individuals with headaches requiring brain imaging at a tertiary center from 2011 to 2024. Those with “red flags” (neurological signs, severe headaches, or lack of response to analgesics) were evaluated by a neurologist for imaging needs. Abnormal imaging was defined as new brain findings explaining the headache. The study details patient characteristics, etiologies, clinical features, immediate treatments, and impacts on pregnancy management.

**Results**: During the study period, 458 pregnant individuals had brain imaging due severe headache. Of them only 34 (7.4%) had positive imaging. Abnormal Imaging results included stroke (n = 10,29.4%), space‐occupying lesion  (n = 7,20.6%), Idiopathic Increased Intracranial Pressure (n = 6,6%.71), Cavernoma (n = 5, 14.7%), and other less frequent causes such as Multiple Sclerosis, Posterior Reversible Encephalopathy Syndrome (PRES) (n = 2, 5.9%) Acute Disseminated Encephalomyelitis (ADEM), Optic Neuritis, Subarachnoid Hemorrhage, Reversible cerebral vasoconstriction syndrome (RCVS), Internal carotid thrombus (n = 1, 2.9% each).

There were no differences in maternal and pregnancy baseline characteristics (Table 1).

Among individuals with abnormal imaging 19 (55.9%)  needed medical or surgical treatment including 4 (11.7%) brain surgeries, 1 (2.9%) angiographic intervention,  16 individuals (47.0%) received medical treatment such as aspirin, low molecular weight heparin, nimodipine, steroids, and acetazolamide. Five (14.7%) patients underwent an early delivery between 29‐36 weeks (Table 2).

**Conclusion**: At a tertiary care center, the yield of brain imaging to diagnose significant etiologies in pregnant individuals is 7.4% with CVA being the most common etiology. Medical or surgical treatment was required in 55.9% of the positive imaging group.

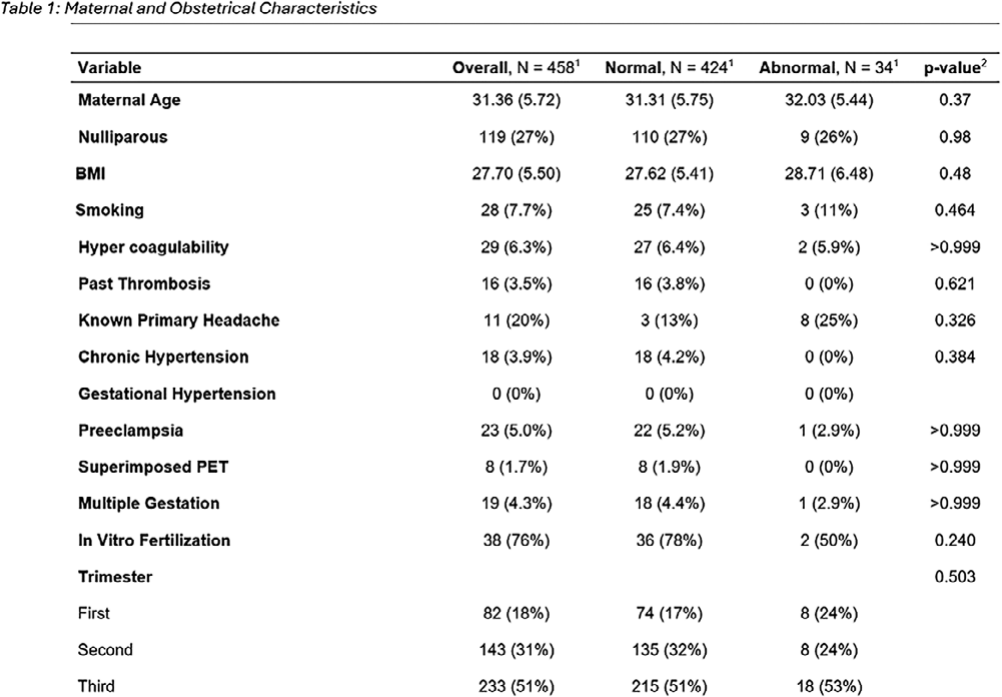

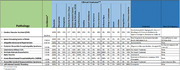

## 1138 | Impact of Residence in a Food Desert on Prenatal Metabolic Health and Diet Indices

Noor K. Al‐Shibli^1^; Anne L. L. Dunlop^2^; Suchitra Chandrasekaran^3^

*^1^Emory University, School of Medicine, Atlanta, GA; ^2^Emory University School of Medicine, Atlanta, GA; ^3^Emory University, Atlanta, GA*

4:00 PM ‐ 6:00 PM

**Objective**: With increasing rates of metabolic syndrome and associated adverse pregnancy outcomes, there is a need to better understand risks for poor nutritional status. Little is known about the impact of the lived food environment on prenatal diet quality. We aimed to explore impact of living in a food desert (FD) on prenatal dietary scores & metabolic health indices.

**Study Design**: We performed a single‐center prospective pregnancy cohort study from 2014‐2024. Participants were a subset of those enrolled in the Atlanta African American Maternal‐Child Cohort, which included pregnant people ages 18‐40 with a singleton 8‐14 weeks’ gestation who identified as Black/African American. Block‐Bodnar food frequency questionnaires (FFQ) were administered at 8‐14 or 24‐30 weeks’ gestation. Dietary scores and indices were calculated from FFQ responses. FD status was assigned to participants residing in low‐income/low‐access census tracts according to U.S. Department of Agriculture (USDA) definitions. Descriptive and bivariate statistics were utilized.

**Results**: Of n = 543 pregnancies, n = 242 (45%) lived in a FD. Median cohort age was 25 and BMI 29.8 kg/m2. There were no significant differences in glycemic/lipid indices (Table 1) or diet quality scores (Table 2) by FD status. Notably, median Alternate Healthy Eating Index‐Pregnancy (AHEI‐P) scores showed low‐moderate adherence to dietary guidelines for those residing in FD and non‐FDs (41.5 & 40.2, respectively). Both groups demonstrated pro‐inflammatory Dietary Inflammatory Index (DII) scores.

**Conclusion**: Prenatal glycemic/lipid intake and diet quality did not significantly differ by FD status. Rather, those residing in FD & non‐FD demonstrated high intake of total sugar, mono/poly‐unsaturated fat, low‐moderate adherence to pregnancy dietary guidelines, and pro‐inflammatory diets. This suggests that metabolic dietary risks in pregnancy are common, irrespective of FD status. Future directions should focus on nutrition education and behavioral approaches for all pregnant people regardless of their geographic food environment.

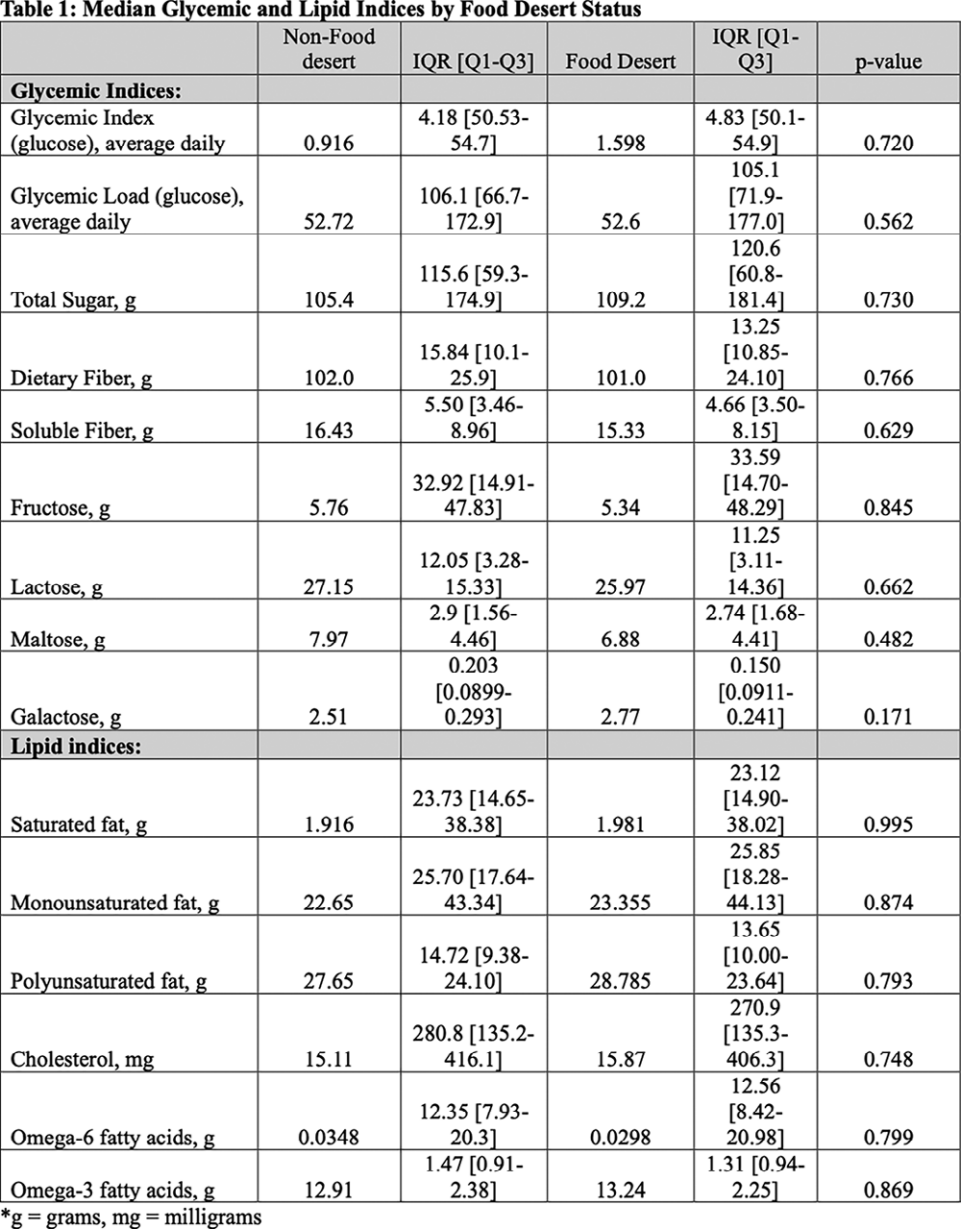

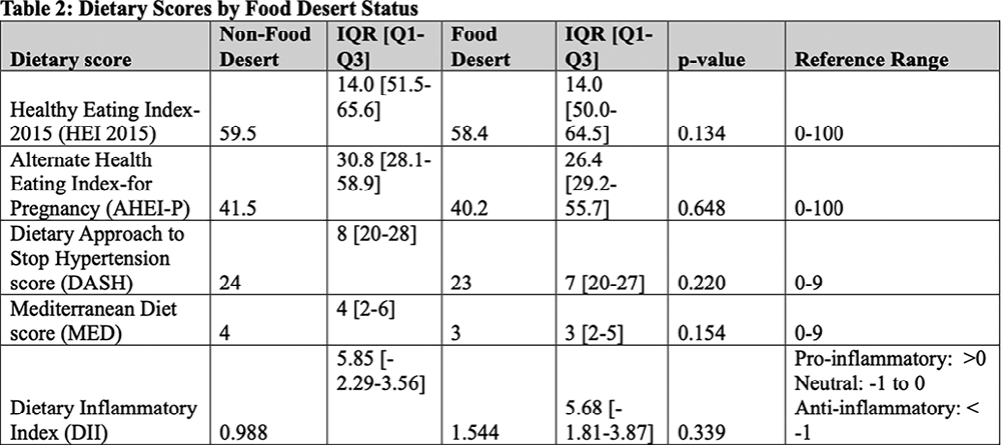

## 1139 | Hypertensive Disorders and Postpartum Follow up: Care in and Outside the Hospital

Oladunni Ogundipe^1^; Michelle Carfagno^2^; Adeola F. Adeyeye^3^; Cassandra Heiselman^3^; Kimberly Herrera^4^

*^1^Stony Brook University, Stonybrook, NY; ^2^Stony Brook University Hospital, Stonybrook, NY; ^3^Stony Brook University Hospital, Stony Brook, NY; ^4^Stony Brook University, Stony Brook, NY*

4:00 PM ‐ 6:00 PM

**Objective**: We sought to evaluate the role of social determinants of health (SDH) in postpartum care delivery and utilization among patients with hypertensive disorders of pregnancy (HDP) by examining rates of 6 week follow up and unscheduled healthcare utilization.

**Study Design**: This was a retrospective cohort study of patients who delivered at a single hospital between 12/2020 and 2/2023 including those diagnosed with preeclampsia with/without severe features during pregnancy or in the postpartum period. Exclusion criteria: < 18 years of age, delivered < 20 weeks GA, or delivered with community practices. SDH included age, self‐identified race and ethnicity, marital status, number of living children, health insurance, and area deprivation index (a mapping tool from 1 [most advantaged] to 10 [least advantaged] obtained from census data displaying relative socioeconomic conditions of regions). Statistical analysis included cross‐tabulation, chi‐square tests, independent samples t‐tests, bivariate analyses, and multivariable logistic regression analyses. Statistical significance was determined using a threshold of p < 0.05.

**Results**: 257 patients were included (Table 1). Being single, having prior living children, and belonging to a racial or ethnic group other than White non‐Hispanic was significantly associated with poor attendance at the six‐week postpartum visit (Table 2). Unscheduled hospital admissions were also significantly associated with being black, higher ADI scores, and having living children (p< 0.05) (Table 3).

**Conclusion**: SDH were significantly associated with poor postpartum follow up and higher rates of unscheduled health care utilization.

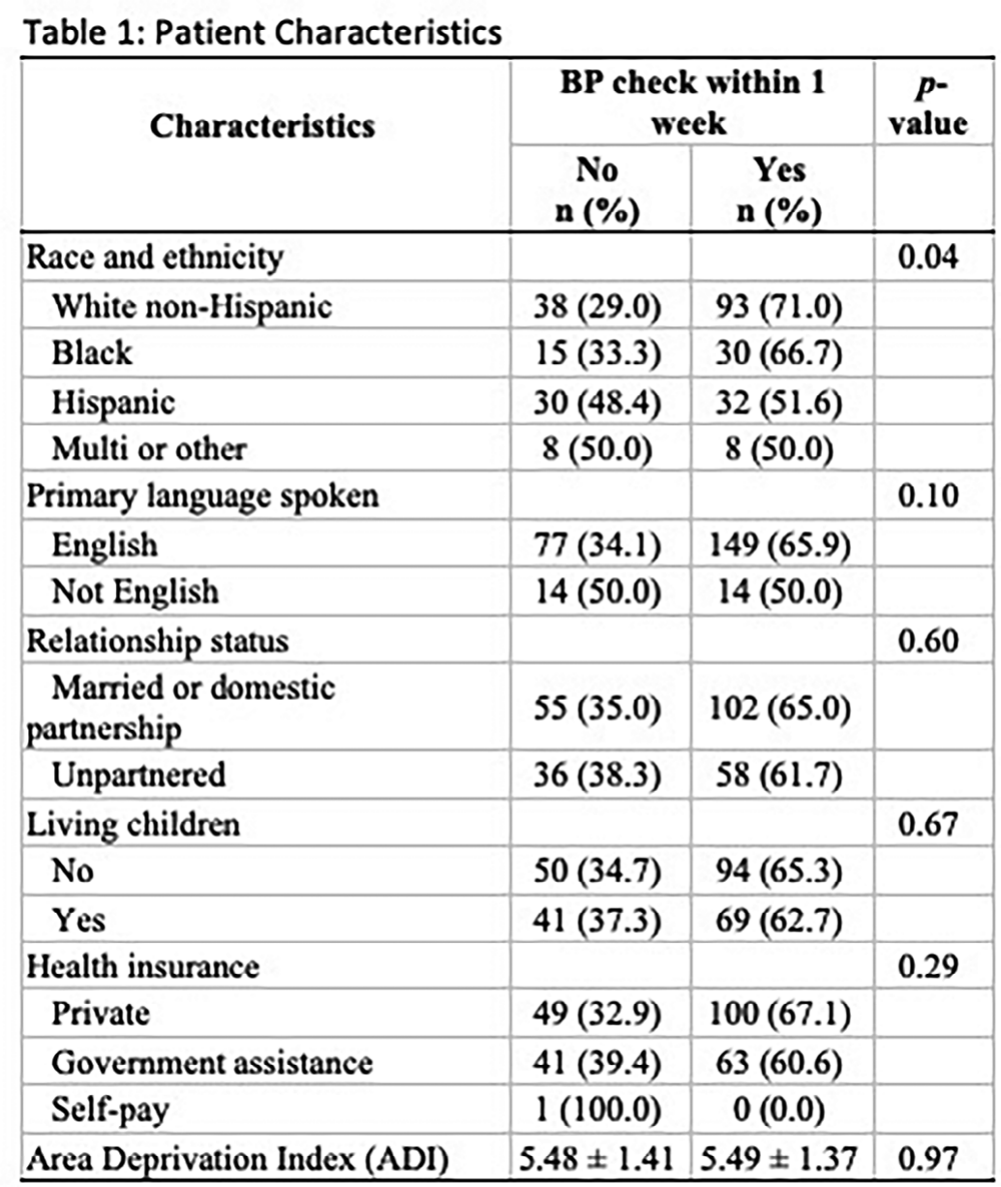

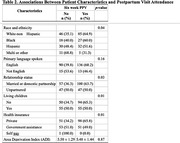

## 1140 | Characterization of a 96‐Cytokine Panel in Blood of Women with Healthy Pregnancies

Viktoriia Kolesnyk^1^; Colleen Sinnott^2^; Tobias Hartwich^2^; Yang Yang‐Hartwich^1^; Olga Grechukhina^1^

*^1^Yale School of Medicine, New Haven, CT; ^2^Yale University, New Haven, CT*

4:00 PM ‐ 6:00 PM

**Objective**: Serological cytokines are potential biomarkers of pregnancy pathology. While this is a convenient non‐invasive technique, establishing normal *versus* abnormal levels in pregnancy has been challenging, limiting its diagnostic value. Some cytokines vary between individuals or due to physiological factors. By evaluating a panel of 96 cytokines in serum from patients with normal pregnancies, our objective is to devise a comprehensive nomogram that integrates a complex cytokine profile and will allow determination of the normal versus abnormal cytokine levels.

**Study Design**: Serum samples collected at four timepoints (∼12 weeks, 18‐22 weeks, 26‐30 weeks, 34‐38 weeks) from 15 healthy pregnant subjects who had no pregnancy complications and delivered full‐term were obtained from an institutional biobank. Human Cytokine 96‐Plex Discovery Assay was performed. One‐way ANOVA was used to analyze trends in each cytokine for each pregnancy. Cytokines with both low variability between timepoints and consistent trends across individuals were identified for further testing in clinical setting.

**Results**: Among the 96 cytokines (growth factors, chemokines, interleukins and others) that were assessed, levels of 54 significantly increased or decreased during pregnancy while 42 remained stable. Of those that showed change, 7 with low variability were manually picked: GROa, IL‐27 and VEGF‐A increased while BCA‐1, IL‐24, Lymphotactin, MCP‐4 decreased during the course of pregnancy. Among those that showed no change during pregnancy IL‐22, 6CKine (aka CCL21), Exotaxin 3, HMGB1, IL‐11, IL‐28A, MIP‐3b were identified to have low variability.

**Conclusion**: Out of 96 tested cytokines, 54 change significantly during healthy pregnancy, which should be accounted for when studying maternal serum cytokines in various pregnancy pathologies. The remaining cytokines do not show level change over the course of pregnancy. Specific cytokines that are better suited for clinical setting were identified, setting up a foundation for studying cytokine levels and identifying cytokine footprint in specific pregnancy pathologies.

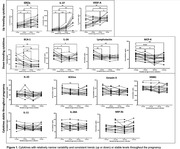

## 1141 | Impact of Fetal Surgery Type and Complications on Maternal Perinatal Mental Health

Olivia Liseth^1^; Jessica Weng^1^; Mauro Schenone^2^; Katherine Moore^2^; Hannah Betcher^2^; Yan Li^2^; Megan E. Branda^2^; Enid Rivera‐Chiauzzi^2^; Alyssa Larish^2^

*^1^Mayo Clinic MSTP, Rochester, MN; ^2^Mayo Clinic, Rochester, MN*

4:00 PM ‐ 6:00 PM

**Objective**: Previous work has identified high rates of depression and anxiety in fetal surgery patients, therefore, we sought to determine the effect of fetal surgery approach type and outcomes on perinatal mood and anxiety disorders (PMAD).

**Study Design**: A retrospective chart review was conducted of fetal surgery patients from 2017‐2024 at a tertiary care center. Demographics, surgical, obstetric, and psychiatric diagnoses were recorded. Complications included surgical site infection, chorioamnionitis, NICU admission > 45 days, PPROM, preterm labor, or delivery < 34 weeks gestation. Severe complications included delivery < 30 weeks gestation, unplanned delivery at the time of the procedure, maternal ICU admission, and fetal or neonatal death. Minimally invasive surgery (MIS) included both fetoscopic and ultrasound guided.

**Results**: Our cohort had an average age of 30.5 yrs and a mean gravidity of 2.7 (n = 141). Of 141 surgeries, 32 (22.7%) were open and 109 (77.3%) were MIS. Pregnancy complications occurred in 115, with severe complications in 64 patients (Table 1). The rates of any or severe complications were 75.0%, 21.9%, respectively, for open procedures and 83.5%, 52.3%, respectively, for MIS. MIS patients did not have a lower rate of exacerbation or de novo PMAD (25/109, 22.9%) compared to open surgery (8/32, 25.0%, p = 0.8084; Χ^2^ test). Patients with any complication did not have a higher risk of exacerbation or de novo PMAD (30/115, 26.1%), versus patients without any complications (3/26, 11.5%, p = 0.1315; Fisher's exact test). After adjusting for multiple testing, no individual complication was predictive of subsequent PMAD. Among patients with baseline mood disorders, complications did not result in increased exacerbation compared to no complications (2/5, 40% without vs 25/48, 52.1% with, p = 0.6687; Fisher's exact test).

**Conclusion**: Both complications and surgical approach are not predictive of PMAD; universal follow‐up and screening are needed. Further research is necessary to determine the most effective timepoints and methods of screening in this population with a uniquely challenging life event.

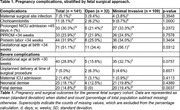

## 1142 | Intrapartum Maternal Bacteremia: an Important Predictor of Puerperal Adverse Outcomes

Omer Nir^1^; Tal Israeli^2^; Michal Axelrod^2^; Sharon Amit^3^; Shali Mazaki‐Tovi^2^; Michal Fishel Bartal^4^

*^1^Department of Obstetrics and Gynecology, Sheba Medical Center, Tel Hashomer, Ramat Gan, HaMerkaz; ^2^Department of Obstetrics and Gynecology, Sheba Medical Center, Tel HaShomer, Ramat Gan, HaMerkaz; ^3^Clinical Microbiology, Sheba Medical Center, Tel HaShomer, Ramat Gan, HaMerkaz; ^4^UTH Houston & Sheba Medical Center Israel, Houston, TX*

4:00 PM ‐ 6:00 PM

**Objective**: We aimed to assess the risk factors, pathogens, and outcomes in Individuals with intrapartum fever with and without proven bacteremia bacteremia.

**Study Design**: We conducted a retrospective study at a single tertiary center (October 2011 to February 2024). Included in the study were individuals with singleton pregnancies in labor beyond 24 weeks who experienced intrapartum fever (defined as > 38 degrees Celsius). We excluded those with unavailable blood cultures for review. Baseline demographic and delivery characteristics were compared between those with positive cultures and those with negative or non‐pathogenic blood cultures (contaminants). The primary outcome was a composite adverse maternal outcome (CAMO), which included any of the following: length of stay > 4 days, ICU admission, CT‐guided drainage, fever‐related surgery, and death. A multivariable regression analysis was performed for the primary outcome, with adjusted odds ratios (aOR) and 95% confidence intervals (CI) reported.

**Results**: Out of 86,904 deliveries, 3,603 (4.1%) had intrapartum fever, and 3,047 (85%) met inclusion criteria. Among these, 117 (3.8%) had positive blood cultures, with the most common blood‐borne pathogens being non‐hemolytic Streptococci, Enterobacterales, and GBS (Figure). Those with bacteremia differed from those without in terms of age, rate of nulliparity, rate of spontaneous labor onset, preterm delivery, and neuraxial analgesia. Furthermore, patients with bacteremia had a higher maximal temperature and WBC count, had a higher rate of cesarean delivery, and required additional broad‐spectrum second‐line antibiotics. Following multi‐variable regression, those with bacteremia had a higher rate of CAMO (85.5% vs. 38.6%, aOR 16.0, CI 7.2‐35.8, Table) compared to those without bacteremia.

**Conclusion**: Intrapartum fever accompanied by bacteremia is associated with an increased need for second‐line antibiotic use and poorer maternal outcomes. This study underscores the importance of blood culture among individuals at risk in guiding treatment and identifying individuals at higher risk of adverse maternal complications.

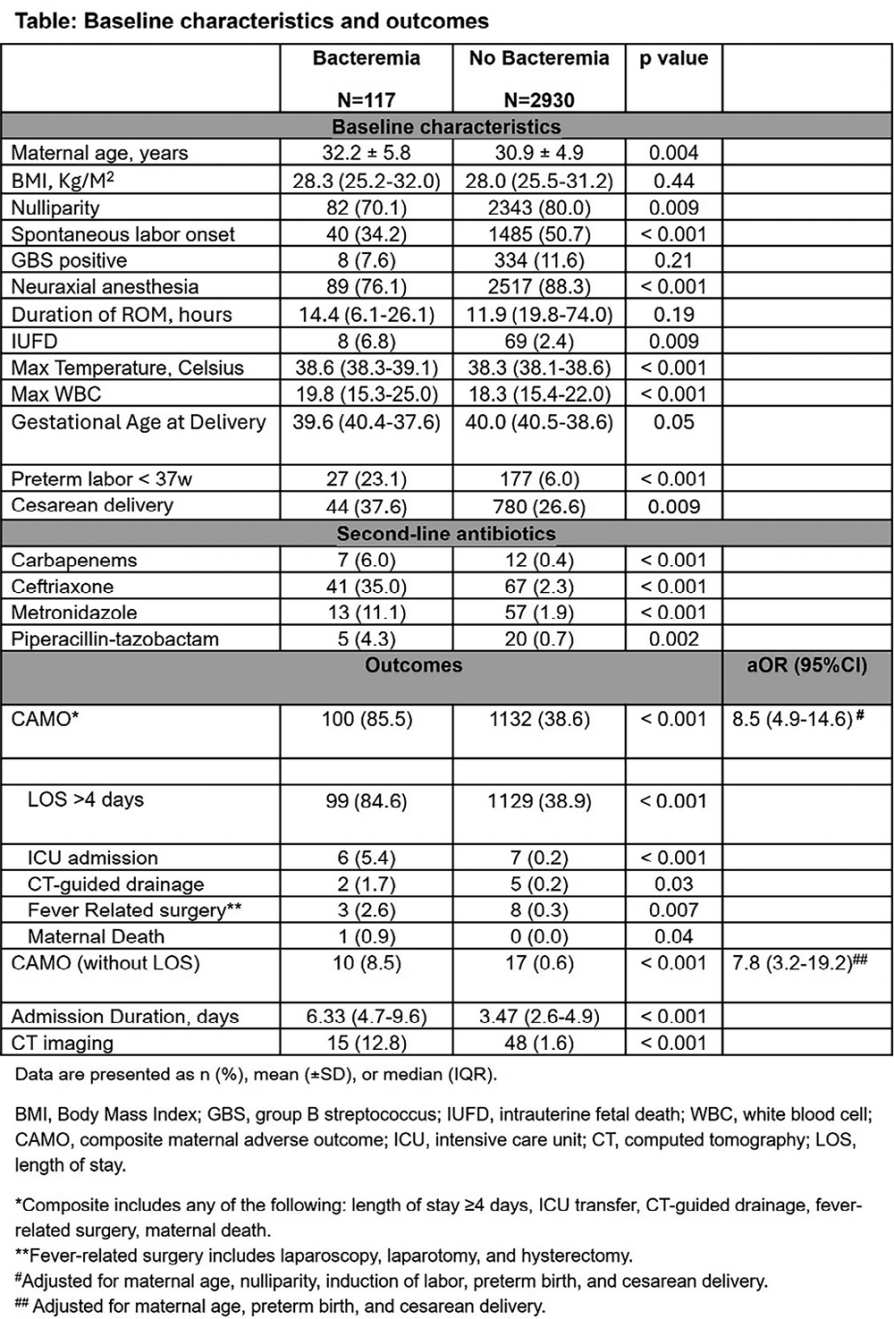

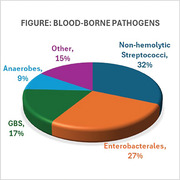

## 1143 | Maternal Lipidomics for the Accurate Prediction of Fetal Cyanotic Congenital Heart Defects

Onur Turkoglu^1^; Nadia Ashrafi^2^; Ali Yilmaz^2^; Romana Ashrafi^2^; Abdullah Khan^2^; Perry Friedman^3^; Ciara Talbot^4^; Stewart graham^2^; Ray Bahado‐Singh^5^

*^1^Baylor College of Medicine, Houston, TX; ^2^William Beaumont University Hospital, Research Institute, Metabolomics Division, Royal Oak, William Beaumont University Hospital, Research Institute, Metabolomics Division, Royal Oak, MI; ^3^Memorial Healthcare System, Memorial Healthcare System, FL; ^4^Kaiser Permanente, Kaiser Permanente Brookwood at Peachtree Medical Office, GA; ^5^Corewell Health William Beaumont University Hospital, Oakland University William Beaumont School of Medicine, Royal Oak, MI*

4:00 PM ‐ 6:00 PM

**Objective**: Late diagnosis of cyanotic congenital heart defects (CCHD) significantly increases newborn morbidity and mortality. However, a significant percentage of CCHD cases remain undetected prenatally. Our objectives were therefore to evaluate the accuracy of maternal serum lipidomic markers for the prenatal detection of CCHD and also to elucidate the pathogenesis of these disorders.

**Study Design**: This prospective case‐control study included 40 cases of CHD (22 cyanotic, 18 acyanotic) and 39 unaffected controls. Maternal serum samples were profiled via a combination of Ultra‐High‐Performance Liquid Chromatography coupled with Mass Spectrometry and Nuclear Magnetic Resonance. The mean of each lipid specie was compared between CCHD vs all others. Logistic regression models were developed for the detection of CCHD. Areas under the receiving operating curve (AUC), sensitivity, and specificity values were calculated. Metabolite Set Enrichment Analysis (MSEA) was also performed to discover the top dysregulated biochemical pathways in CCHD.

**Results**: In total, 803 lipids and 97 metabolites were profiled in the maternal serum. In CCHD cases, concentrations of ceramides, phosphatidic acids, phosphatidylcholines, and phosphatidylethanolamines were decreased while fatty acids and phosphatidylserines were elevated (p< 0.05) (Fig1). The lipidomics model (Fig2) achieved outstanding predictive accuracy: AUC (95%) = 0.959 (0.946‐0.972). This outperformed the accuracy of routinely used standard clinical risk predictors for CHD (diabetes + obesity + hypertension + in vitro fertilization + family history of CHD + alcohol or tobacco exposure). MSEA analysis revealed profound alterations of mitochondrial beta‐oxidation of short and long‐chain fatty acids involved in lipid metabolism, in CCHD.

**Conclusion**: Fetal CCHD was associated with significant disturbance of lipid metabolism. Maternal lipid biomarkers were highly accurate for the detection of fetal CCHD. This has the potential for the development of non‐invasive pregnancy population screening which could help to reduce mortality and morbidity in CCHD.

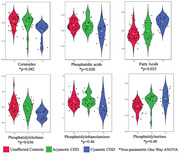

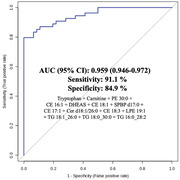

## 1144 | Association Between Health Literacy and Cardiovascular Health in Early Pregnancy

Onyinye Ohamadike^1^; Xiaoning Huang^2^; Sadiya S. Khan^1^; William A. Grobman^3^; Philip Greenland^4^; David M. Haas^5^; Uma M. Reddy^6^; Lynn M. Yee^1^

*^1^Northwestern University Feinberg School of Medicine, Chicago, IL; ^2^Northwestern University, Chicago, IL; ^3^The Ohio State University, Columbus, OH; ^4^Northwestern, Northwestern/Chicago, IL; ^5^Indiana University School of Medicine, Indianapolis, IN; ^6^Columbia University, New York, NY*

4:00 PM ‐ 6:00 PM

**Objective**: Inadequate health literacy (HL) has been associated with adverse pregnancy outcomes, yet it is not well understood to what extent HL is associated with early pregnancy cardiovascular health (CVH). We aimed to understand the association between inadequate HL and CVH in early pregnancy.

**Study Design**: In this secondary analysis of cross‐sectional data from a large, multi‐site cohort of nulliparous people enrolled in a prospective observational study conducted at 8 US medical centers, we included participants who completed an assessment of HL at 6 to 13 weeks of gestation. HL, assessed using the Rapid Estimate of Adult Literacy in Medicine‐Short Form (REALM‐SF), was categorized as inadequate if the REALM‐SF score was < 7 (8^th^ grade level or lower) and adequate if score 7 or greater. The primary outcome was CVH at 6‐13 weeks of gestation, quantified by a modified (i.e., non‐fasting) version of the American Heart Association's Life's Essential 8 (LE8) score, a validated marker of CVH in which lower scores indicate worse CVH. Multivariable linear regression was performed to evaluate the association of inadequate HL with early pregnancy modified LE8 score.

**Results**: Of the 4,424 participants who completed the REALM‐SF, 825 (18.6%) had REALM‐SF scores representative of inadequate HL. People with adequate HL were older, less likely to identify as non‐Hispanic Black or Hispanic, had higher incomes and education, and were less likely to have public insurance. Participants with adequate HL had higher early pregnancy LE8 scores [79.0 ± 13.3 vs. 73.0 ± 14.2, p< 0.001; mean difference 7.1 (95% CI 5.2‐8.8)] compared to participants with inadequate HL. This association persisted after adjustment for age, insurance type, and family income (aβ 3.8, 95% CI 2.2‐5.5). The overall association between adequate HL and better CVH was driven by the glucose, diet, physical activity, and sleep subcomponents of the LE8 (Table).

**Conclusion**: Adequate HL was associated with better CVH scores in early pregnancy. These findings highlight the importance of optimizing HL prior to pregnancy which might promote CVH in the peripartum period.

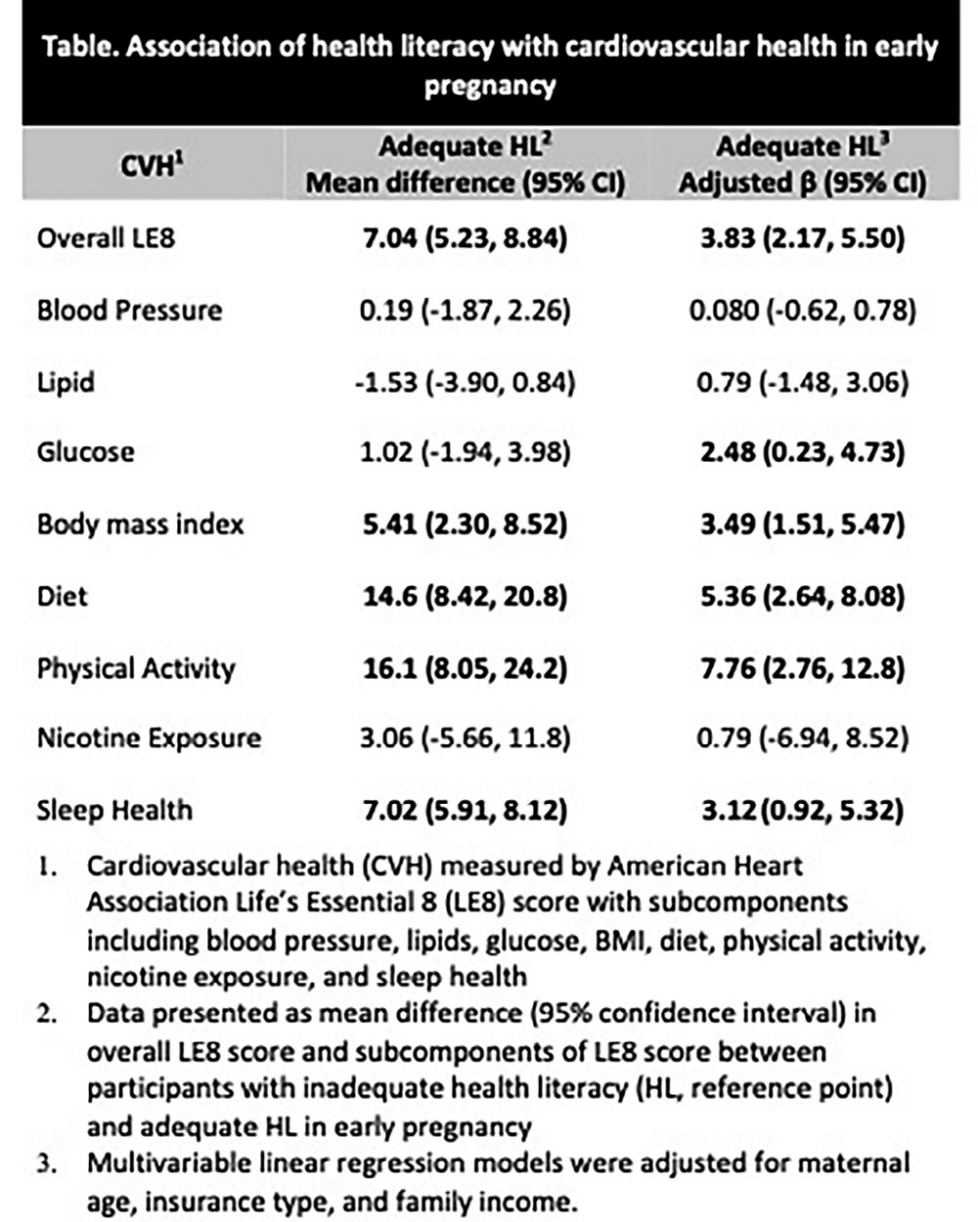

## 1145 | Association Between Health Literacy and Cardiovascular Health at 2‐7 Years After First Birth

Onyinye Ohamadike^1^; Xiaoning Huang^2^; Sadiya S. Khan^1^; William A. Grobman^3^; Philip Greenland^4^; David M. Haas^5^; Uma M. Reddy^6^; Lynn M. Yee^1^

*^1^Northwestern University Feinberg School of Medicine, Chicago, IL; ^2^Northwestern University, Chicago, IL; ^3^The Ohio State University, Columbus, OH; ^4^Northwestern, Northwestern/Chicago, IL; ^5^Indiana University School of Medicine, Indianapolis, IN; ^6^Columbia University, New York, NY*

4:00 PM ‐ 6:00 PM

**Objective**: Inadequate health literacy (HL) is associated with worse cardiovascular health (CVH) in non‐pregnant individuals and, among pregnant individuals, adverse pregnancy outcomes (APO). We aimed to study the association between HL in early pregnancy and CVH at 2‐7 years after first birth.

**Study Design**: In this secondary analysis of longitudinal data from a large, multi‐site cohort of nulliparous people enrolled in a prospective observational study at 8 US medical centers, we included participants who completed an assessment of HL. HL, assessed using the Rapid Estimate of Adult Literacy in Medicine‐Short Form (REALM‐SF), was categorized as inadequate if the REALM‐SF score was < 7 and adequate if score 7 or greater. The primary outcome was CVH, quantified by the American Heart Association's Life's Essential 8 (LE8) score, a validated marker of CVH in which lower scores indicate worse CVH. Multivariable linear regression models were performed to evaluate the association of inadequate HL in early pregnancy (6‐13 weeks of gestation) with LE8 score at 2‐7 years after first birth.

**Results**: Of the 4,424 participants who completed the REALM‐SF in early pregnancy and had 2‐7 year follow‐up data, 825 (18.6%) had REALM‐SF scores representative of inadequate HL. People with adequate HL were older, less likely to identify as non‐Hispanic Black or Hispanic, had higher incomes and education, and were less likely to have public insurance. Participants with adequate HL in early pregnancy had higher postpartum LE8 scores [80.5 ± 13.3 vs. 73.2 ± 13.8, p< 0.001; mean difference 7.3 (95% CI, 3.5‐11.1)] compared to those with inadequate HL. When adjusted for age, insurance type, and family income, this relationship persisted (aβ 3.3, 95% CI 0.4‐6.2). Findings were driven by higher scores for the body mass index, diet, physical activity, nicotine exposure, and sleep subcomponents of the LE8 (Table).

**Conclusion**: Adequate HL in early pregnancy is associated with better CVH scores at 2‐7 years after first birth, suggesting interventions to optimize maternal HL should be evaluated as a strategy for long‐term CVH promotion.

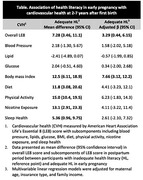

## 1146 | Association of Aspirin Use and Preeclampsia in Pregnancy after Kidney Transplant

Ophelia Yin^1^; Lisa Coscia^2^; Serban Constantinescu^3^; Michael Moritz^2^; Roxanna Irani^1^

*^1^Division of Maternal Fetal Medicine and Reproductive Genetics, Department of Obstetrics, Gynecology, and Reproductive Sciences, University of California San Francisco, San Francisco, CA; ^2^Transplant Pregnancy Registry International, Gift of Life Institute, Philadelphia, PA; ^3^Section of Nephrology, Hypertension, and Kidney Transplantation, Department of Medicine, Lewis Katz School of Medicine, Temple University, Philadelphia, PA*

4:00 PM ‐ 6:00 PM

**Objective**: We aim to characterize trends in aspirin use in the kidney transplant population and the associations of aspirin use with preeclampsia, obstetric outcomes, and graft function.

**Study Design**: We conducted a retrospective cohort study using the Transplant Pregnancy Registry International of pregnancies after kidney transplant reaching 20 weeks gestation with year of conception from 2000 onwards. The primary outcome was preeclampsia with delivery at < 37 weeks. Univariate and multivariable logistic regression was performed in R.

**Results**: Our analysis included 708 pregnancies after kidney transplant resulting in 723 livebirths. Of the 708 pregnancies, 74 (10.5%) reported aspirin use and 634 (89.5%) reported no aspirin use in pregnancy. There was a significant increase in aspirin use over time, from 3.1% in 2000 to 25.0% in 2019, p< 0.001 (Figure 1). Aspirin use was associated with higher body mass index (BMI) (27 vs 25 kg/m^2^), later year of conception (2014 vs 2009), and living unrelated kidney donor (32.4% vs 15.7%), all p< 0.01. After adjustment for chronic hypertension, year, diabetes, pre‐pregnancy creatinine, multiple gestation, donor type, and BMI, preeclampsia with delivery < 37 weeks occurred in 20 (27.0%) of aspirin use and 135 (21.3%) of no aspirin use pregnancies, (aOR 1.22, 95% CI 0.64 to 2.24, p = 0.5). Aspirin use was associated with postpartum hemorrhage (6.8% vs 2.2%, p = 0.04) but not blood transfusion (5.4% vs 1.7%, p = 0.06) and aspirin use was associated with reduced rates of small for gestational age neonates (9.1% vs 19.8%, p = 0.02). Aspirin use was not associated with any adverse graft outcomes including creatinine changes in pregnancy, acute graft rejection, or graft loss 2 years from time of delivery.

**Conclusion**: In kidney transplant recipients who reported aspirin use in pregnancy, there was no difference in rates of preeclampsia with preterm delivery. The lack of association could be due to variation in dosage, timing of use, and indication. The increased rates of postpartum hemorrhage and reduction in rates of small for gestational age babies warrants future prospective investigation.

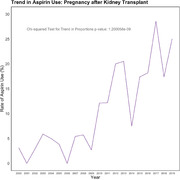

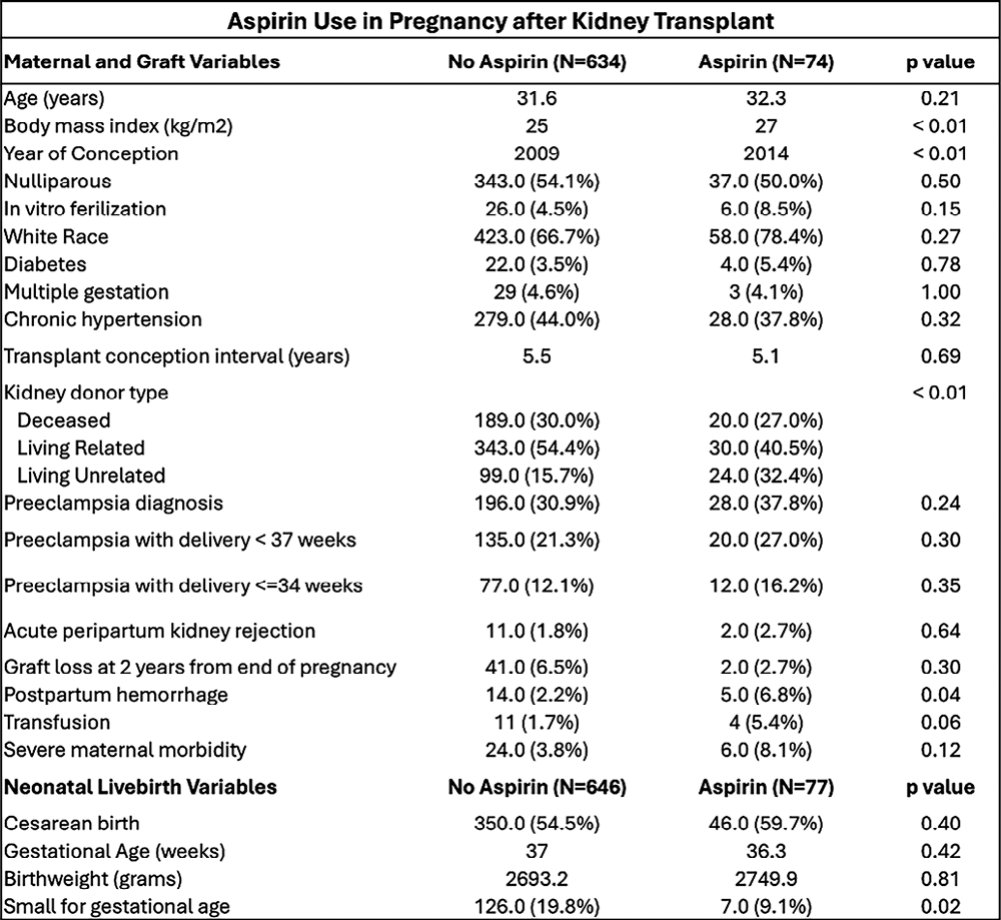

## 1147 | Transcriptional Meta‐Analysis of Preeclampsia Compared to Placenta Accreta: Implications for Placentation

Ophelia Yin^1^; Laura Almonte^2^; Romina Appierdo^3^; Monica Yang^4^; Tomiko Oskotsky^5^; Yalda Afshar^6^; Marina Sirota^5^

*^1^Division of Maternal Fetal Medicine and Reproductive Genetics, Department of Obstetrics, Gynecology, and Reproductive Sciences, University of California San Francisco, San Francisco, CA; ^2^Bakar Computational Health Sciences Institute, University of California (UCSF), San Francisco, San Francisco, CA; ^3^Program in Cellular and Molecular Biology, Department of Biology, University of Rome “Tor Vergata”, San Francisco, CA; ^4^Division of Rheumatology, Department of Medicine, University of California (UCSF), San Francisco, CA; ^5^Bakar Computational Health Sciences Institute, University of California (UCSF), San Francisco, CA; ^6^University of California, Los Angeles, Los Angeles, CA*

4:00 PM ‐ 6:00 PM

**Objective**: To compare the transcriptomic signature of early onset preeclampsia with placenta accreta to gain insight into genes and pathways relevant to placentation at the maternal‐fetal interface.

**Study Design**: The Gene Expression Omnibus (GEO) was queried for preeclampsia and placenta accreta transcriptional datasets derived from human placental tissue from 2010 onwards. Microarray analysis was conducted in R and performed with *crossmeta* or *oligo* and batch corrected with *ComBat*. Differential gene expression using *Limma* was adjusted for cell type. Processing of single cell data was performed using *Seurat* with *MAST*. Gene hyper enrichment, correlation testing, and gene set enrichment analysis with *clusterprofiler* was performed for pathway analysis.

**Results**: We performed a gene expression meta‐analysis of placental samples from 86 patients with early onset preeclampsia and 65 preterm controls from 4 publicly available microarray datasets. This preeclampsia gene signature was compared to 1 placenta accreta microarray with 6 accreta and 3 preterm controls and 1 placenta accreta single‐cell dataset with 3 accreta and 3 controls. Comparing the 4,903 differentially expressed genes in the early onset preeclampsia meta‐analysis to 416 differentially expressed genes in placenta accreta microarray, we found 120 overlapping genes with significant negative correlation in expression (*r* = ‐0.39, p = 8.06e‐6). Key genes included *ANKRD37, AOX1, CP, GBP3, IFIT1, IGFBP6, NEK11, OPRK1*, and *SERPINA3* (Figure 1). Comparing to single‐cell placenta accreta, we found that preeclampsia genes were enriched in all cell types and negatively correlated for decidua type, endothelial, and extravillous trophoblasts (Table 1). Shared pathways included hypoxia, glycolysis, oxidative phosphorylation, and early estrogen response.

**Conclusion**: We identified early onset preeclampsia genes that are oppositely regulated in placenta accreta, both in bulk tissue and in specific cell types at the maternal‐fetal interface. We hypothesize that these candidate genes underly the biology of placental attachment and spiral artery remodeling for both disorders.

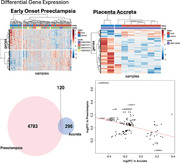

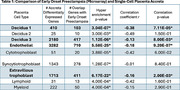

## 1148 | Nomogram for Predicting Obstetric Anal Sphincter Injuries in Nulliparous Women

Or Bercovich^1^; Daniela Chen^2^; Ohad Houri^1^

*^1^Rabin Medical Center, Petach Tikva, HaMerkaz; ^2^Helen Schneider Hospital for Women, Rabin Medical Center, Petach‐Tikva, Tel Aviv, HaMerkaz*

4:00 PM ‐ 6:00 PM

**Objective**: To develop a predictive model for obstetric anal sphincter injuries (OASIS) in nulliparous women.

**Study Design**: This retrospective observational study analyzed data from a tertiary care center. Inclusion criteria encompassed all singleton deliveries at ≥ 37 gestational weeks from January 2007 to December 2019, involving nulliparous women. The primary outcome was the occurrence of OASIS. The full multivariate logistic regression included variables such as age, body mass index (BMI), mode of delivery (MOD) (vaginal or vacuum‐assisted), use of oxytocin, spontaneous or induced labor, birth weight, neuraxial analgesia, and episiotomy. The stepwise model, selected based on the Akaike Information Criterion, retained the variables BMI, MOD, induction, and birth weight. A nomogram was then developed using the best‐fitting model to visually represent the predicted probability of OASIS. The nomogram was calibrated and validated internally to ensure accuracy and reliability.

**Results**: The study cohort comprised 22,738 deliveries: 17,518 vaginal deliveries (77%) and 5,220 vacuum‐assisted deliveries (23%). Overall, 221 cases (1%) were diagnosed with OASIS. The variables significantly associated with the primary outcome in the multivariate model were BMI (p = 0.034), MOD (p < 0.001), induction of labor (p = 0.010), and birth weight (p < 0.001). Their respective predicted probabilities are represented in the nomogram (Figure 1).

**Conclusion**: The developed nomogram allows for the calculation of individual risk scores for OASIS, identifying patients with a very high risk for OASIS (above 10%) versus those with low risk (less than 1%).

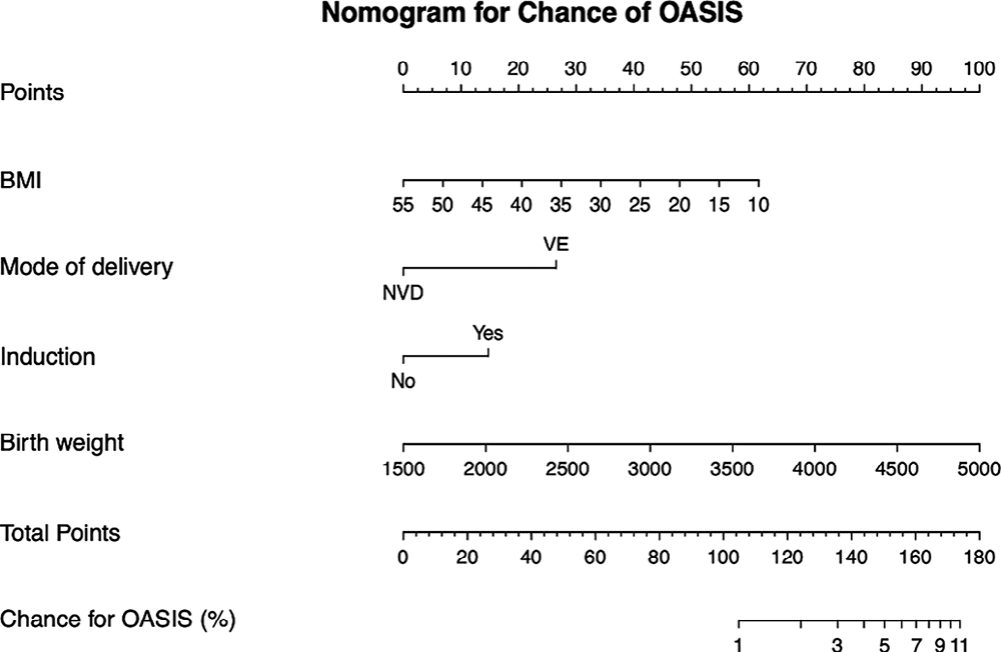

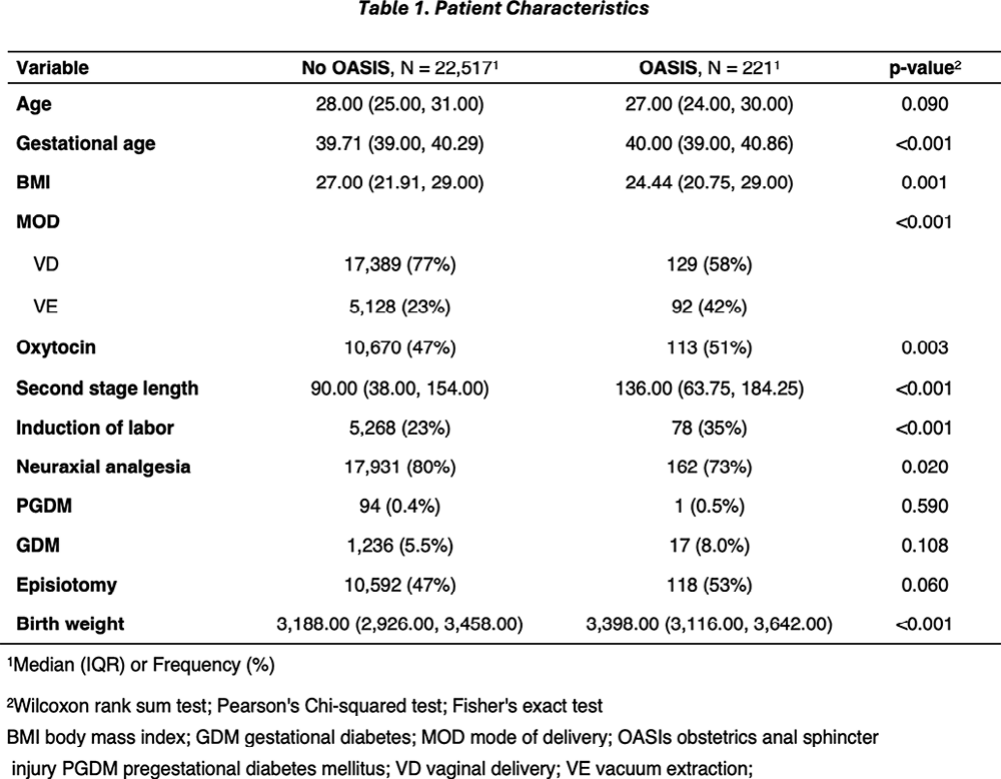

## 1149 | Intra‐Operative Clinical 2019 FIGO Classification has High Correlation with Histologic PASD in a High‐Risk Population

Oxana M. Zarudskaya^1^; Caitlin E. Martin^1^; Georgia A. McCann^1^; Kayla E. Ireland^1^; Alixandria F. Pfeiffer^2^; Patrick S. Ramsey^3^

*^1^University of Texas Health Science Center at San Antonio, San Antonio, TX; ^2^University of Texas Health Science Center at San Antonio, San Antonio, TX; ^3^University of Texas Health Science Center San Antonio, San Antonio, TX*

4:00 PM ‐ 6:00 PM

**Objective**: Accurate confirmation of intra‐operative diagnosis of placenta accreta spectrum disorder (PASD) is essential for proper use of resources, use or withhold uterine artery embolization, transfusion strategy, anesthesia care and post operative management. To date there is limited data to suggest how clinical findings correlate to microscopic or histologic diagnosis, when universal clinical FIGO staging is used intra‐operatively.

**Study Design**: We conducted a retrospective review of all patients evaluated and delivered in our center with suspected PASD between January 2022 and July 2024. At our institution we implemented a universal reporting protocol for macroscopic PASD findings during cases with suspected PASD.

**Results**: During the study interval, 56 cases of PASD were delivered at our center. Of these cases, 50 had FIGO staging documentation of macroscopic PASD findings intra‐operatively and were included in analysis. Clinical staging was done by the operative members of our PASD team. Histologic findings were uniformly documented by a designated pathologist. Coefficients of correlation were assessed between the clinical grade and the histologic grade. There was a very strong coefficient of correlation between clinical and histologic FIGO grading in cases with suspected PASD–0.96. This correlation was even stronger when analyzed grouped cased with accreta/increta/percreta 3A versus PASD cases with severe invasion (percreta 3B and 3C)–0.99.

**Conclusion**: Our data demonstrated a very strong correlation between clinical and histologic/microscopic FIGO grading when used in cases with suspected PASD.

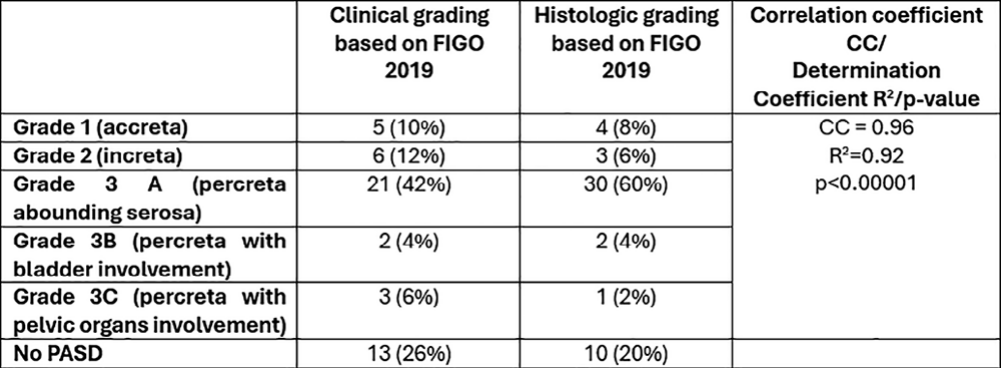

## 1150 | What type of Anesthesia Causes Worse Maternal Outcomes in Patients with Hypertensive Disorders of Pregnancy?

Patricia Rojas Mendez^1^; Rachael Sampson^1^; Jordan A. Gillenwater^1^; Marcia Des Jardin^2^; Nicholas Baranco^1^; Robert Silverman^1^

*^1^SUNY Upstate Medical University, Syracuse, NY; ^2^University of Alabama at Birmingham, Birmingham, AL*

4:00 PM ‐ 6:00 PM

**Objective**: Hypertensive disorders of pregnancy (HDP) are a leading cause of adverse obstetrical and neonatal outcomes. No optimal anesthesia strategy is established for pregnancies affected by HDP. The study objective is to compute the association between type of anesthesia and maternal and short‐term neonatal adverse outcomes among patients with HDP undergoing cesarean delivery (CD).

**Study Design**: Secondary analysis was performed on a cohort of 736 patients who underwent CD at our institution from 2022‐2023. HDP was stratified into five groups: chronic hypertension (HTN), gestational HTN, preeclampsia without severe features (SF), preeclampsia with SF (including HELLP and eclampsia), and superimposed preeclampsia. The primary outcome was a composite of maternal morbidity (CMM) (postoperative (PO) fever, PO antibiotics, blood transfusion, intensive care unit (ICU) admission, reoperation, readmission and death) in patients undergoing general vs. neuraxial anesthesia for CD. Secondary outcomes were Apgars < 7 and neonatal ICU admission. Multivariate logistic regression was performed. Adjusted odds ratios (aOR) with 95% confidence intervals (CI) for primary and secondary outcomes were calculated after controlling for potential confounders.

**Results**: In the cohort, 546 patients (74%) had HDP and were included in the analyses. Of these, 14% received general anesthesia and 86% received neuraxial anesthesia. Demographic characteristics and other preoperative risk factors were similar in both groups (Table 1). After adjusted analysis, general anesthesia was associated with higher rates of CMM when compared to neuraxial anesthesia among all groups (aOR 3.96, 95% CI 1.54, 10.22). The association was maintained after controlling for urgency, gestational age and delivery indication. We found no association between the type of anesthesia and 5‐minute Apgar < 7 or neonatal ICU admission (Table 2).

**Conclusion**: Among patients with HDP undergoing CD, general anesthesia is associated with higher rates of adverse maternal outcomes even after controlling for urgency and delivery indication.

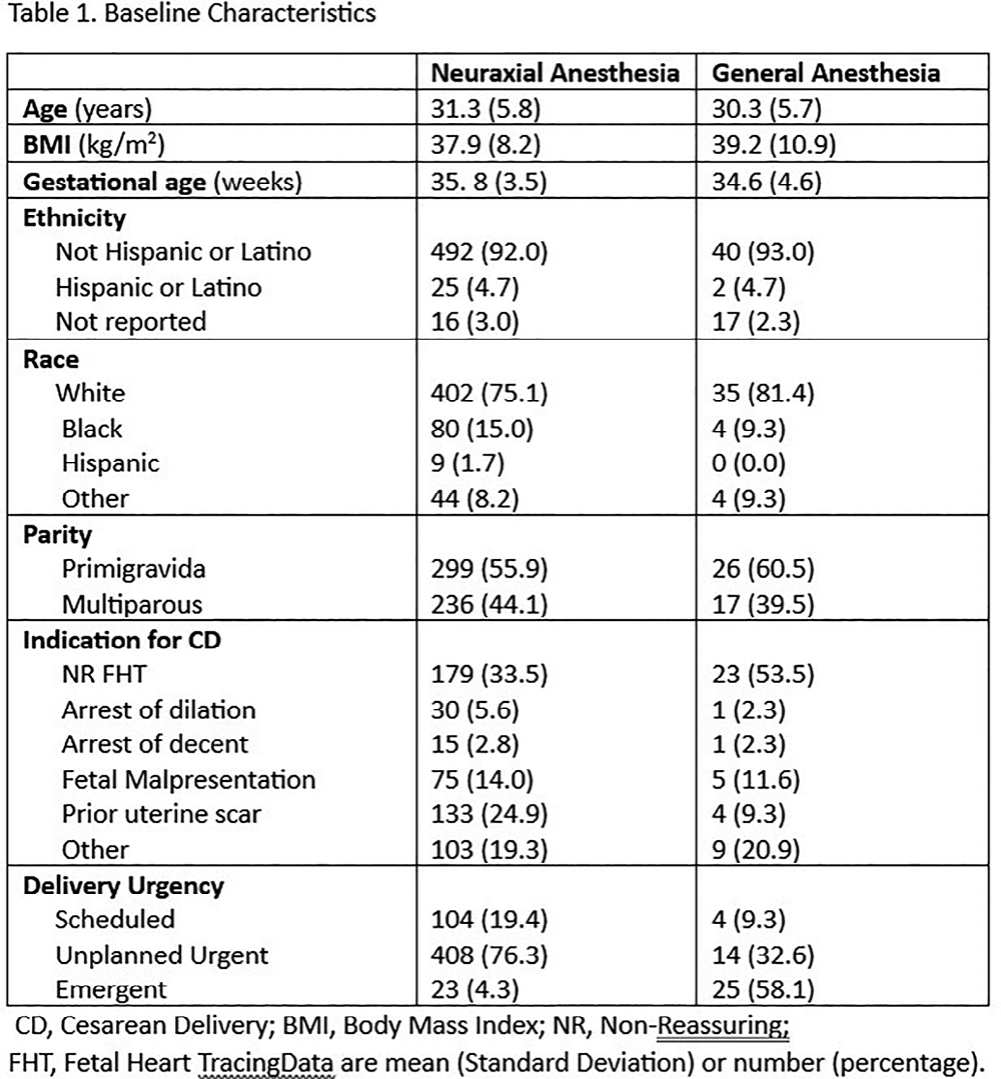

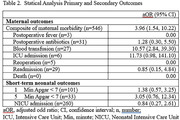

## 1151 | Does an Early Isolated Increase in Fetal Abdominal Circumference Heighten the Risk of Macrosomia?

Patrick S. Kim^1^; David J. Rivera Vazquez^1^; Tiffany A. Lowtan^1^; Kelsey M. Pozerski^1^; Krista Howard^2^; Bradley H. Sipe^3^; Rachelle Schwartz^3^

*^1^Orlando Health Bayfront Hospital, St. Petersburg, FL; ^2^Texas State University, San Marcos, TX; ^3^Johns Hopkins All Children's Hospital, St. Petersburg, FL*

4:00 PM ‐ 6:00 PM

**Objective**: To determine if an isolated second trimester fetal abdominal circumference (AC) >90th %tile is an independent risk factor for large for gestational age (LGA) neonates and macrosomia.

**Study Design**: This multi‐center retrospective cohort study analyzed patients who delivered term singletons between 1/1/2019‐1/1/2024. The exposed group involved normally grown fetuses with isolated AC's >90th %tile, measured between 18 and 24 weeks gestation. Control subjects were matched in a 1:1 ratio from the same period. Congenital anomalies were excluded. Statistical analyses included independent t‐tests for continuous variables, chi‐squared tests for categorical variables, and binary logistic regression models for LGA and macrosomia.

**Results**: Of the 1,188 patients screened, 752 met inclusion criteria (n = 376 per cohort). Mean AC in the AC >90 group was 94%tile compared to 51%tile in controls. Birthweights were higher in the AC >90 group (3,629g vs. 3,257g, p< 0.001). Rates of LGA (29.5% vs. 6.6%, p< 0.001) and macrosomia (15.2% vs. 2.9%, p< 0.001) were higher in the AC >90 group. AC >90%tile was an independent risk factor for both LGA (AOR 7.05 [3.43, 14.52]) and macrosomia (AOR 4.51 [2.78, 7.32]). Additional logistic regressions are shown in Table 1. Higher maternal BMI increased the risk of LGA and macrosomia. There were no differences in parity, pregestational or gestational diabetes, or mode of delivery between cohorts. Gestational hypertension was more prevalent in the AC >90 group (13.8% vs. 9.0%, p = 0.039), whereas chronic hypertension was more prevalent among controls (13.3% vs. 7.2%, p = 0.006). A sub‐analysis of macrosomic infants is shown in Table 2. Macrosomic infants in the AC >90 group trended toward higher rates of lacerations (72.7% vs. 33.3%) and shoulder dystocia (9.1% vs. 0%), although overall incidence was low.

**Conclusion**: An isolated second trimester AC >90th %tile in an otherwise normally grown fetus is an independent risk factor for both LGA and macrosomia. An isolated increase in AC may also be a risk factor for lacerations and shoulder dystocia at birth, but further research is required.

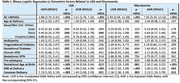

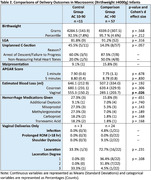

## 1152 | Mode of Delivery and Neonatal Survival Among Periviable Vertex Singleton Pregnancies

Peeraya S. Sawangkum^1^; Jason L. Salemi^1^; Jean Paul P. Tanner^1^; Alexia Eiges^2^; Anjali J. Kaimal^3^

*^1^Department of Obstetrics and Gynecology, University of South Florida, Tampa, FL; ^2^Department of Obstetrics and Gynecology, University of South Florida, Nashville, TN; ^3^University of South Florida, Tampa, FL*

4:00 PM ‐ 6:00 PM

**Objective**: To determine the association between mode of delivery and neonatal survival for periviable, vertex, singleton gestations.

**Study Design**: Retrospective cohort study using a maternal‐infant linked database of live births in Florida from 2006 to 2019. Vertex, singleton deliveries of infants born at 22w0d to 25w6d with attempted neonatal resuscitation were included. We calculated risk ratios (RRs) for binary outcomes and hazard ratios (HRs) for time‐to‐event outcomes that were adjusted for confounders. Outcomes were examined overall and stratified by week of gestation. A planned sensitivity analysis was performed examining outcomes in neonates who survived beyond 24 hours of life.

**Results**: A total of 4,820 periviable deliveries were identified. Of those, 2,472 (51.3%) were cesarean deliveries. People who underwent cesarean delivery were more likely to have hypertension and diabetes. In the overall model, vaginal delivery was associated with a higher risk of mortality at 24 hours (aRR 1.20, 95% CI 1.01‐1.44), lower risk of mortality at 7 days (aRR 0.88, 95% CI 0.79‐0.99), but no difference in mortality at 28 days (aRR 0.94, 95% CI 0.86‐1.02) or 1 year (aRR 0.95, 95% CI 0.89‐1.03). After excluding neonates who died within the first 24 hours, vaginal delivery was associated with lower risk of mortality, with aRR of 0.70 (95% CI 0.59‐0.83) at 7 days, aRR of 0.87 (95% CI 0.77‐0.97) at 28 days, and aRR of 0.91 (95% CI 0.83‐1.00) at 1 year. Stratified analyses by gestational age demonstrated increased mortality with vaginal delivery among 22w‐ and 23w‐neonates (aHR 1.48, 95% CI 1.06‐2.06 and aHR 1.19, 95% CI 1.01‐1.43 respectively), but decreased mortality with vaginal delivery among 24w‐ and 25w‐neonates (aHR 0.75, 95% CI 0.63‐0.89 and aHR of 0.72, 95% CI 0.58‐0.90 respectively).

**Conclusion**: Our study demonstrates the effect of mode of delivery on neonatal survival in the periviable period is dependent upon gestational age. Further study is needed to understand the clinical situations in which cesarean is most likely to improve neonatal outcome in order to balance this against potential maternal complications.

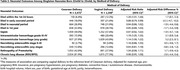

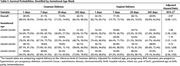

## 1153 | Fetal Fraction in Patients with Autoimmune Diseases of the Gastrointestinal Tract

Chi Nguyen^1^; Nicole Legro^1^; Sara N. Iqbal^1^; Taylor Martin^2^; Bailey Liter^2^; Allison Willett^2^; Victoria R. Greenberg^3^

*^1^Medstar Washington Hospital Center / Georgetown, Washington, DC; ^2^Georgetown School of Medicine, Washington, DC; ^3^Medstar Washington Hospital Center /Georgetown, Washington, DC*

4:00 PM ‐ 6:00 PM

**Objective**: We sought to evaluate differences in fetal fraction (FF) in individuals with autoimmune diseases of the gastrointestinal (GI) tract compared to those without autoimmune disease. Additionally, we assessed whether use of immunomodulating medications (IM) at time of cell‐free DNA (cfDNA) screening has any effect on FF for patients with autoimmune diseases of the GI tract

**Study Design**: We performed a retrospective cohort study of singleton pregnancies who had cfDNA testing 2017‐2024. Patients without autoimmune disease served as controls and were matched to patients with GI autoimmune diseases (ulcerative colitis and Crohn's) during pregnancy in a 3:1 fashion, controlling for gestational age and BMI. Exclusion criteria included those on anticoagulants, history of organ transplant, and/or high‐risk cfDNA results concerning for aneuploidy. IM therapy included mesalamine, infliximab, vedolizumab, and adalimumab. We used the student t‐test for normally distributed variables, chi‐square and Fisher's exact tests for categorical variables and ANOVA to compare three groups. Multivariate regression analysis was performed to evaluate the effect of immunomodulator use on fetal fraction, after adjusting for BMI and gestational age at the time of cfDNA draw.

**Results**: We included 300 patients in the study (75 with GI autoimmune disease and 225 without disease). Baseline demographics between the two groups were similar with respect to BMI, race, and smoking status (Table 1) with a statistically significant difference in age. Mean fetal fractions were similar in those with and without disease (Table 1). In the subset of individuals with GI autoimmune disease, use of IM medication did not result in higher fetal fraction compared to individuals with GI autoimmune disease not on IM medication, even after adjusting for gestational age and BMI (aβ .072, 95%CI ‐1.2, 2.3)

**Conclusion**: Contrary to other reports, this study found no effect of GI autoimmune disease on FF, even when adjusting for IM use. Further research is needed to assess the impact of various autoimmune diseases on FF.

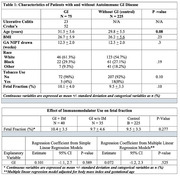

## 1154 | Novel uEMG Technology Significantly Improves Preterm Birth Prediction: The Labor Status Monitor

Ponnila S. Marinescu^1^; Neil S. Seligman^1^; Jennifer B. Gilner^2^; Sarah Caveglia^1^; Win Phyo^1^; Hayden Rigsbee^2^; Roger C. Young^3^

*^1^University of Rochester Medical Center, Rochester, NY; ^2^Duke University Hospital, Durham, NC; ^3^PreTeL, Inc., Portland, OR*

4:00 PM ‐ 6:00 PM

**Objective**: Using current methods, sensitivity for predicting preterm birth (PTB) in patients presenting with contractions before 37 weeks GA is < 50%. While not all contractions contribute towards labor, legacy toco cannot distinguish those who will deliver preterm. We developed a novel Labor Status Monitor that uses uterine bioelectrical synchronization and between‐contraction, tissue‐level stimulation to predict PTB < 7 days.

**Study Design**: Multicenter, prospective cohort study of pregnant individuals between 26‐36 weeks GA, with a cervix £ 5 cm and self‐reporting ^3^ 1 contraction in 10 minutes (min). 6‐channel uEMG recordings were obtained using PreTeL's directional uEMG sensors applied to the abdomen for up to 4 hours (hr). uEMG data were classified on a channel‐by‐channel basis as rest, contraction‐associated signals (CAS), or fasciculation‐like signals (FLS, tissue‐level stimulation between contractions). Contraction Synchronization Index (CSI) was calculated as CAS in > 5 channels observed over the previous 10‐min, averaged over 60 min. FLS index (FLSi) was calculated as the fraction of time FLS occurred in each channel over the previous 10‐min, averaged over all 6 channels, and then averaged over 1 hr. Primary outcome was delivery < 7 days of uEMG recording. Sensitivity and specificity were analyzed from receiver operator characteristic curves.

**Results**: 43 subjects were evaluated; 63% (27/43) delivered < 37 weeks GA, 34% (15/43) delivered < 7 days. AUC was 0.74 for CSI and 0.66 for FLSi for delivery < 7 days. Based on AUC, optimal sensitivities and specificities were 74% and 70% for CSI and 56% and 70% for FLSi. Assuming test independence, by applying CSI first, then applying FLSi to indeterminate results, sensitivity and specificity were 89% and 91% for delivery < 7 days.

**Conclusion**: Our analysis of the Labor Status Monitor suggests that uEMG significantly improves PTB prediction compared to current methods. Ability to predict PTB < 7 days has the potential to meaningfully impact management, particularly administration of antenatal corticosteroids. Further studies are needed to validate these findings.

## 1155 | Identifying Markers for Gestational Diabetes Using Continuous Glucose Monitoring

Pranaya Chilukuri; Lindsay Beechem; Aric Schadler; Abigail Leonhard; Josie Llanora; Kristina Naseman; Karen Playforth

*University of Kentucky, Lexington, KY*

4:00 PM ‐ 6:00 PM

**Objective**: Per ACOG, all pregnant women should be screened for gestational diabetes (GDM) with an oral glucose tolerance test (OGTT). As continuous glucose monitor (CGM) use increases, CGM goals “target range” and “time in range” have been set for diabetes in pregnancy. This study aims to identify CGM metrics that may be linked to GDM diagnosis in patients considered at high risk.

**Study Design**: This single‐institution pilot prospective observational study includes pregnant individuals at 24‐28 weeks’ with singleton gestation and 1‐hour OGTT of 140‐200mg/dl, without previous Type 1 or 2 Diabetes, PCOS, or recent metformin, insulin, or corticosteroid administration. Upon IRB approval, participants were screened through electronic medical records and recruited to wear Dexcom G6 CGM in blinded mode for 7‐10 days. Patients completed a 3‐hour OGTT, determining GDM diagnosis. Pearson's chi‐square, independent samples t‐test, and Mann‐Whitney U tests were used as appropriate for analysis.

**Results**: CGM was worn by 22 participants, with 7 subsequently diagnosed with GDM. Metrics of CGM average glucose (124 vs. 111, p = 0.011), time in range (82% vs. 90%, p = 0.047), time above range (17% vs. 7%, p = 0.021), and glucose management indicator (GMI), analogous to HbA1c, (6.3 vs. 6.0, p = 0.009) significantly differed for those diagnosed with and without GDM, respectively (Table 1). Significant differences have not resulted in birth outcomes yet, with recruitment ongoing.

**Conclusion**: This study highlights CGM metrics that may be used as potential markers for patients at risk for GDM.

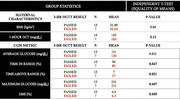

## 1156 | Interpregnancy Development of Chronic Hypertension after a Hypertensive Disorder of Pregnancy and Subsequent Pregnancy Outcomes

Praveen Ramesh^1^; Malamo Countouris^2^; Arun Jeyabalan^3^; Alisse Hauspurg^4^

*^1^Magee‐Women's Hospital of UPMC, Pittsburgh, PA; ^2^University of Pittsburgh Medical Center, Pittsburgh, PA; ^3^University of Pittsburgh School of Medicine, Pittsburgh, PA; ^4^Alpert Medical School of Brown University, Providence, RI*

4:00 PM ‐ 6:00 PM

**Objective**: Hypertensive disorders of pregnancy (HDP) are associated with future cardiovascular (CV) risk, likely mediated through development of chronic hypertension (CHTN). We sought to assess if development of interpregnancy CHTN following a pregnancy complicated by an HDP was associated with adverse outcomes in the subsequent pregnancy.

**Study Design**: This is a cohort study of individuals with consecutive pregnancies with an index pregnancy complicated by an HDP. We compared demographics and outcomes in the subsequent pregnancy between those who developed interpregnancy CHTN with those who had blood pressure (BP) normalization. We included patients with a known diagnosis of CHTN and those who had 2 or more BPs ≥ 140/90 mmHg prior to 20 weeks. The primary outcome was preterm birth < 37 weeks (PTB). Secondary outcomes included a small‐for‐gestational age (SGA) newborn and recurrent pre‐eclampsia. The association of all outcomes with interpregnancy CHTN was adjusted for age, BMI, and maternal self‐identified race (as a social construct) via multivariable logistic regression.

**Results**: We included 4,437 individuals with an HDP during the index pregnancy. 523 (11.8%) developed interpregnancy CHTN. Individuals who developed interpregnancy CHTN were older, more likely to identify as Black race, had a higher index pregnancy BMI, and were more likely to have had preeclampsia with severe features in the index pregnancy compared to those with BP normalization (Table 1). Those who developed interpregnancy HTN were more likely to have a preterm birth, (19.5% vs.11%; aOR 2.25; 95% CI 1.66‐3.04, p< 0.001), an SGA newborn (14.2% vs. 8.3%; aOR 2.48; 95% CI 1.76‐3.48, p< 0.001) and develop recurrent pre‐eclampsia (22.2% vs.12.6; aOR 2.27; 95% CI 1.70‐3.04, p< 0.001) in the subsequent pregnancy compared to those with BP normalization.

**Conclusion**: Individuals with interpregnancy development of CHTN after an HDP have an increased odds of adverse pregnancy outcomes compared to those with BP normalization. Postpartum interventions to reduce the risk of interpregnancy CHTN may improve long‐term CV health and future pregnancy outcomes.

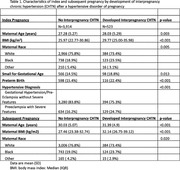

## 1157 | Prenatal Detection of Structural Heart Defects in Pre‐Gestational Diabetes with Expanded Cardiac Screening

Rachel L. Wiley^1^; Maya Pai^2^; Tracy Anton^3^; Maryam Tarsa^4^

*^1^University of California, San Diego, San Diego, CA; ^2^UC San Diego School of Medicine, UC San Diego School of Medicine, CA; ^3^UC San Diego, UC San Diego, CA; ^4^University of California San Diego, San Diego, CA*

4:00 PM ‐ 6:00 PM

**Objective**: Pregnancies affected by pre‐gestational diabetes mellitus (PDM) are recommended to have a fetal echocardiogram (ECHO). In an institution with expanded cardiac screening on level II ultrasounds, we aimed to examine detection of congenital heart disease (CHD) in pregnancies affected by PDM with selective referrals for fetal ECHOs.

**Study Design**: This was a retrospective cohort of pregnancies who received level II ultrasounds in a two‐year period. Ultrasounds were performed by experienced MFM sonographers competent in image optimization techniques to visualize the fetal heart, including utilizing system settings that maximize frame rate and applying color Doppler. Standard and extended views of the heart were obtained and recorded using still frame and cine clips (Table 1). Patients were included if they had a diagnosis of prediabetes, type I, or type diabetes mellitus. Demographics were collected, and neonatal charts were reviewed for CHD. Groups were analyzed using one‐way ANOVA, student's t‐test, chi‐squared or fisher exact test.

**Results**: 306 pregnancies complicated by PDM were included; 66% with type 2 diabetes mellitus, 19% with type I diabetes mellitus and 15% with prediabetes (Table 1). Of these, 64 (21%) had a fetal ECHO, with the indications of suboptimal cardiac views on level II ultrasound (n = 22, 34%), suspected anomaly (n = 20, 31%), other maternal indication (n = 13, 20%) and high initial A1C (n = 9, 14%). In total, 28 (7.8%) neonates had confirmed postnatal diagnosis of CHD, and 16 (57%) of the defects were diagnosed prenatally. CHD detected prenatally had higher A1C (8.1% vs 6.5%, P = 0.016), but no significant difference in maternal BMI (33.2 kg/m^2^ vs 32.5 kg/m^2^, p = 0.74). Of the 12 CHD that were diagnosed only postnatally, there were minor defects (small septal defects, bicuspid aortic valve), but no major cardiac defects.

**Conclusion**: In a tertiary center with expanded cardiac level II ultrasound protocols and selective ECHOs, all major CHD in PDM was detected prenatally. Protocols for selective fetal ECHOs in pregnancies affected by PDM may be considered in experienced tertiary centers.

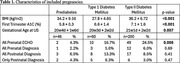

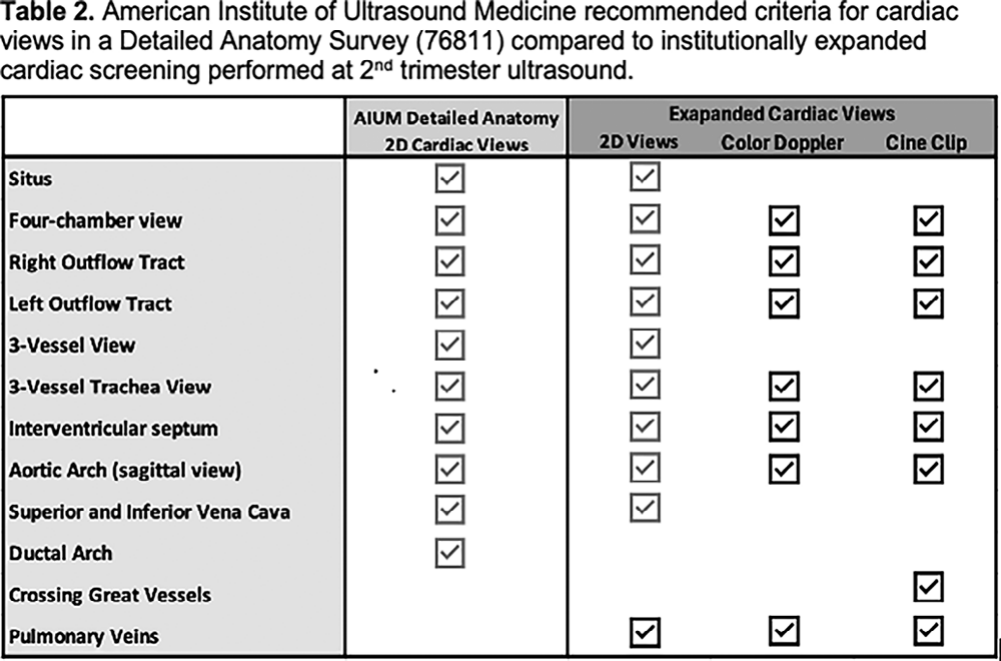

## 1158 | Methods of Estimating Blood Loss and Clinical Outcomes

Rachel L. Wiley^1^; Ipsita Ghose^2^; Hector M. Mendez‐Figueroa^3^; Suneet P. Chauhan^4^

*^1^University of California, San Diego, San Diego, CA; ^2^Baylor College of Medicine, Houston, TX; ^3^McGovern Medical School at UTHealth, Houston, TX; ^4^Delaware Center of Maternal‐Fetal Medicine at Christiana Care, Delaware, DE*

4:00 PM ‐ 6:00 PM

**Objective**: ACOG released a Committee Opinion stating quantitative blood loss (QBL) was more accurate than visually estimated blood loss (EBL), however the clinical impact of QBL remains uncertain.

**Study Design**: This was a secondary analysis of a retrospective cohort study of all singletons delivered at > 20 weeks at a Level IV center during 24 months. This time period included an institutional transition from EBL to QBL, and patients with both methods of estimation recorded were included. Composite maternal adverse outcome included additional surgical techniques, transfusion of > 4 units, balloon tamponade, intensive care unit (ICU) admission, venous thromboembolism (VTE), hysterectomy or death. The diagnostic accuracy of CMAO for EBL and QBL were assessed by using the area under the curve (AUC) of receiver‐operating characteristics (ROC) curves, and diagnostic test statistics were calculated and compared using overlapping confidence intervals.

**Results**: Of 8,623 deliveries, 1,530 (18%) had both EBL and QBL recorded; the demographics are included in Table 1. QBL identified more postpartum hemorrhages (PPH; blood loss > 1000 mL) than EBL (9.0% vs. 6.0%; p< 0.01). CAMO occurred in 42 (3.0%) of patients, and Figure 1 shows the ROC curve for detecting CAMO with a nonsignificant difference in AUC of 0.91 for EBL and 0.86 for QBL (p = 0.07). Using the threshold for PPH of > 1000 mL, EBL and QBL were equally sensitive, but EBL was more specific (95.8%; 95% CI 94.6% ‐ 96.7% vs 92.6%; 95% CI 91.1% ‐ 93.9%, Figure 1). While positive and negative predictive power and likelihood ratio did not differ significantly, negative predictive power and likelihood ratio performed better with EBL.

**Conclusion**: While QBL and EBL are both predictive for maternal morbidity, estimated blood loss is less likely to meet the threshold for PPH, EBL is more specific and may predict adverse outcomes better. The benefits of QBL over EBL and clinical outcomes need to be evaluated in prospective trials.

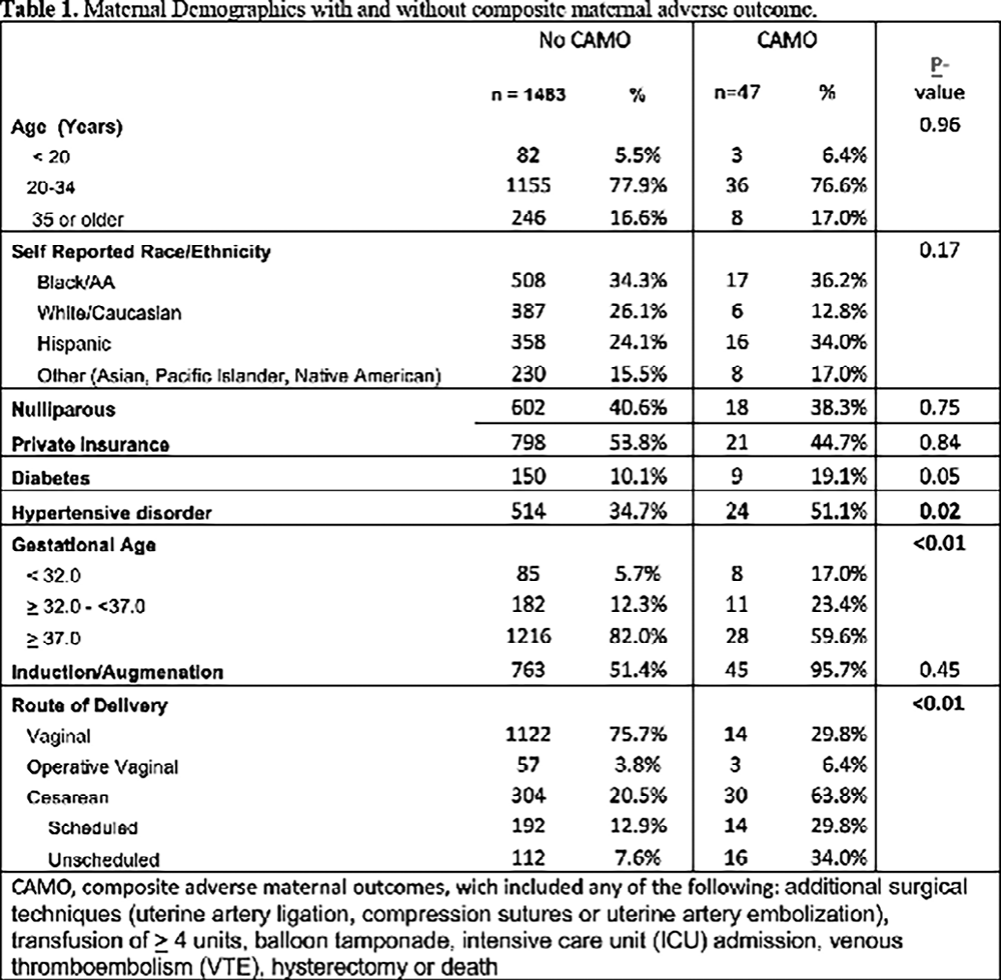

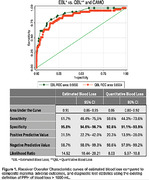

## 1159 | Concordance of Early Pregnancy Smoking Disclosure Rates with DNA Methylation among Pregnant Patients

Rachel S. Ruderman^1^; Lauren S. Keenan‐Devlin^2^; Renee Odom‐Konja^3^; Amy Inkster^4^; Lavisha Singh^5^; Mahitha Chaturvedula^3^; Linda M. Ernst^3^; Alexa A. Freedman^6^; Wendy P. Robinson^7^; Greg E. Miller^6^; Ann EB Borders^5^

*^1^University of Chicago, CHICAGO, IL; ^2^Endeavor Health/ University of Chicago Pritzker School of Medicine, Endeavor Health, IL; ^3^Endeavor Health, Evanston, IL; ^4^The University of British Columbia, The University of British Columbia, BC; ^5^Endeavor Health, Evanston Hospital, Evanston, IL; ^6^Northwestern University, Chicago, IL; ^7^University of British Columbia, University of British Columbia, BC*

4:00 PM ‐ 6:00 PM

**Objective**: Prenatal substance use is an increasing cause of morbidity and mortality. Changes in gene function that do not affect nucleotide sequence, or epigenetics, has emerged as a screening mechanism for substance use. We evaluated the application of serum‐validated epigenetic DNA methylation markers in human placental tissues to assess the correlation between reported smoking and placental epigenetic markers.

**Study Design**: This is a secondary analysis of the Stress, Pregnancy, and Health Study, a prospective study of 605 patients at an urban hospital from 2018‐2022. Patients completed the Health Practices Survey (HPS) during mid‐trimester and reported cigarette or marijuana use during pregnancy (current use) or in the 3 months prior to pregnancy (former use). We evaluated the degree of methylation in placental DNA using CPG sites previously associated with smoking. Differences in site cg05575921 have been reported from placental tissue, while sites cg23079012 and cg22112841 have been associated in serum. Methylation percentages were determined using absolute values; p‐values were calculated from log‐transformed data.

**Results**: Among 488 patients who completed HPS, 440 (90.2%) reported never using cigarettes or marijuana, while 27 (6%) reported current smoking and 21 (5%) former smoking. Current smoking was not associated with placental DNA methylation of cg05575921 (51% vs 53% methylation in never users, p = 0.13). This finding was similar for the other sites (Table 1). A sensitivity analysis comparing current and former users to never users also yielded non‐significant results.

**Conclusion**: In this novel exploration of placental DNA methylation using validated sites, we did not find significant DNA methylation changes with smoking status. However, methylation was lower in current than never smokers, which is similar to the change observed in serum. This study is amongst the first to explore the application of validated DNA methylation markers of smoking on placental tissues, and will inform larger explorations of the association between placental DNA methylation and reported substance use during pregnancy.

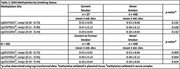

## 1160 | Psychopharmacotherapy Use in Pregnancies Conceived with IVF and Risk of Postpartum Hemorrhage

Rachel Solmonovich; Frank I. Jackson; Jamie Green; Brenda Lin; Randi Goldman; Matthew J. Blitz

*Northwell, New Hyde Park, NY*

4:00 PM ‐ 6:00 PM

**Objective**: To determine differences in the frequency of psychopharmacotherapy (PPT) use during pregnancies conceived with and without in vitro fertilization (IVF). Secondary objectives were to evaluate rates of PPT drug classes among the cohorts and their associations with postpartum hemorrhage (PPH) requiring transfusion of packed red blood cells (pRBC) among IVF pregnancies.

**Study Design**: Retrospective cohort study of all deliveries that occurred at ≥ 23 weeks gestational age in a New York academic health system from January 2019 to December 2022. Adjusted odds ratios for non‐Hispanic White ethnicity, public insurance, and obesity were calculated using a logistic regression in R version 4.3.1.

**Results**: A total of 3,383 IVF and 104,297 non‐IVF pregnancies were included. Patients who conceived via IVF were more likely to self‐identify as non‐Hispanic white (54.9% vs. 43.1%), be nulliparous (59.0% vs. 48.7%), and have private insurance (92.3% vs. 64.4%), p< 0.001. Prenatal PPT exposure was more common in pregnancies conceived with IVF, 6.4% vs. 3.5%; aOR 1.62 (95% CI, 1.39 ‐ 1.88; Table 1). IVF pregnancies exposed to PPT had nearly double the odds of experiencing a PPH requiring pRBC (aOR 1.90; 95% CI, 1.18‐2.95). Selective Serotonin Reuptake Inhibitors (SSRIs) were associated with a two‐fold increase in the odds of PPH requiring pRBC, aOR 2.11 (95% CI, 1.10 ‐ 3.51), and benzodiazepines with three‐fold increased odds, aOR 3.22 (95% CI, 1.56 ‐ 6.12).

**Conclusion**: Prenatal PPT use is higher during IVF pregnancies, specifically SSRIs and benzodiazepines. Their use is associated with increased odds of clinically significant postpartum hemorrhage. While these findings suggest potential risks, they should not discourage mental health treatment, as untreated conditions are also associated with adverse outcomes. These results contribute to comprehensive counseling and informed decision‐making when selecting PPT class during pregnancy.

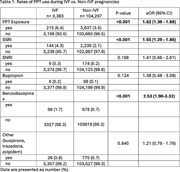

## 1161 | Using Neighborhood‐Level Indices to Discriminate Small for Gestational Age Birthweight Among Racial and Ethnic Groups

Raina Kishan^1^; Daniel L. Kuhr^2^; Nicola F. Tavella^1^; Zahava P. Michaelson^2^; Nicole Parkas^2^; Helen Feltovich^2^; Kimberly B. Glazer^3^

*^1^Icahn School of Medicine at Mount Sinai, New York, NY; ^2^Division of Maternal‐Fetal Medicine, Icahn School of Medicine at Mount Sinai, New York, NY; ^3^Department of Population Health Science and Policy; Department of Obstetrics, Gynecology and Reproductive Science, Blavatnik Family Women's Health Research Institute, Icahn School of Medicine at Mount Sinai, New York, NY*

4:00 PM ‐ 6:00 PM

**Objective**: To examine the performance of publicly available neighborhood‐level indices of the built and social environment in the prediction of small for gestational age (SGA) in different racial/ethnic groups.

**Study Design**: This was an a priori secondary analysis of a retrospective cohort study examining risk of sPTB of 1000 patients who delivered at a tertiary, urban academic hospital between January 2013 and August 2023 with ≥ 1 transvaginal cervical length during pregnancy. The primary outcome was SGA, defined by birth weight ≤ 10th percentile for gestational age (GA), using Intergrowth‐21st growth curves. Medical history, race, delivery GA, and birth weight were abstracted from electronic medical records. The Childhood Opportunity Index (COI), Social Vulnerability Index (SVI) and Index of Concentrations at the Extremes (ICE) were determined through patients’ listed 2020 census tracts. We used logistic regression models to predict the probability of SGA, incorporating neighborhood‐level indices and clinically relevant individual‐level covariates in the whole group and stratified by patient race. We compared model discrimination using the area under the receiver operating characteristics curve (AUC). All analyses were performed in R version 4.4 (24 April 2024).

**Results**: 1038 pregnancies among the 1000 patients met inclusion criteria, 105 of which resulted in SGA infants. 45% of patients were Hispanic, 25% were Black and 18% were White (Table 1). Models with all three neighborhood indices had an AUC of 0.49 in Black, 0.52 in Hispanic, and 0.68 in White patients (Table 2). The addition of socioeconomic and obstetric covariates improved discrimination in each model, particularly in the Black (AUC 0.74) and White (AUC 0.73) patients.

**Conclusion**: Individual neighborhood level indices were poorly predictive of SGA in each racial group. Including all indices improved model ability to discriminate SGA in White patients. The addition of sociodemographic and obstetric characteristics led to improved predictive ability in all racial groups, particularly among Black and White births.

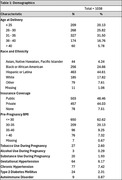

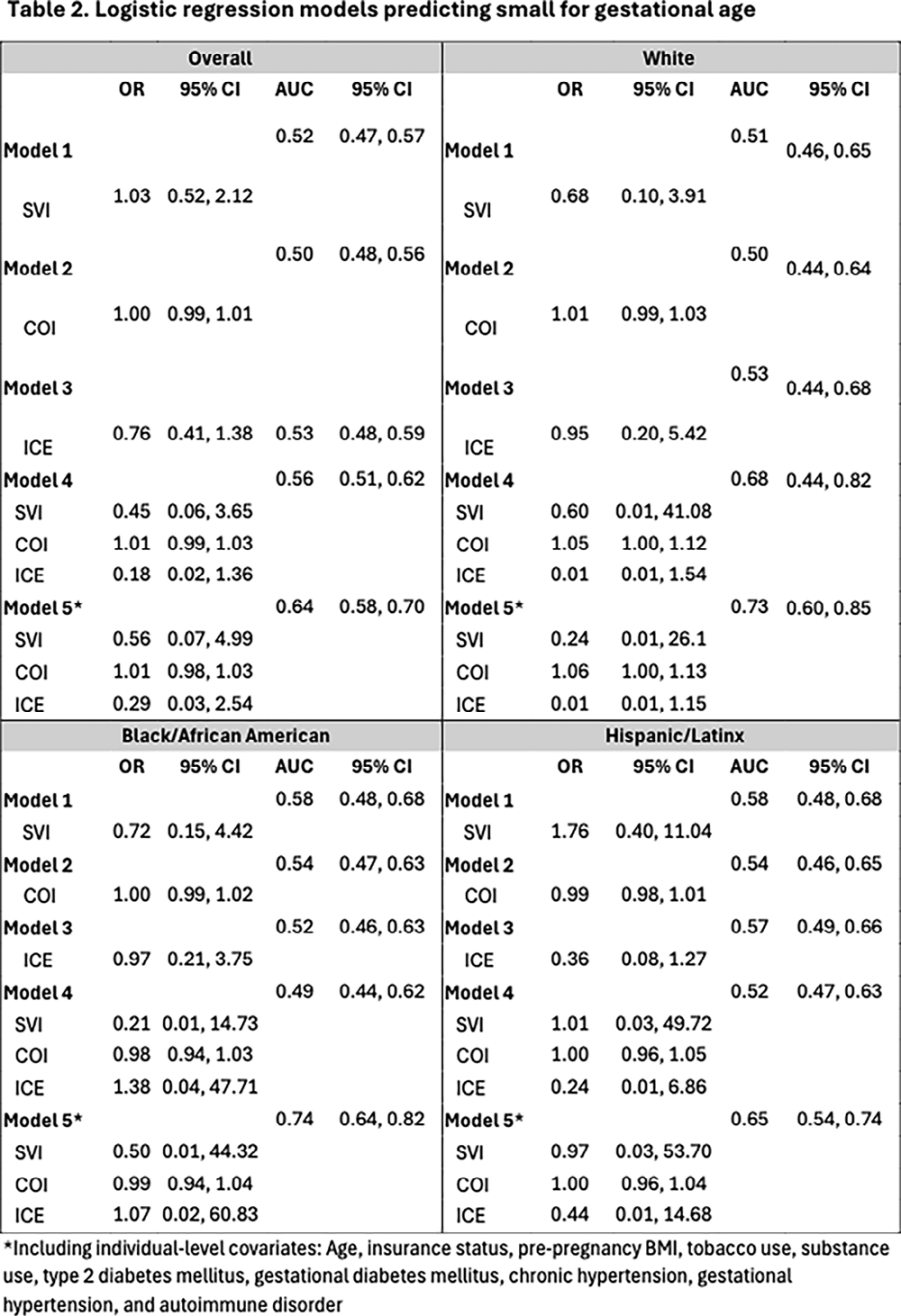

## 1162 | Increased Adverse Perinatal Outcomes (APO) for Twin Pregnancies Complicated by Congenital Heart Disease (CHD)

Rainney Chiu^1^; Lihong Mo^2^; Herman L. Hedriana^2^; Shinjiro Hirose^3^; Sherzana Sunderji^4^; Amelia Mclennan^4^

*^1^University Of California, Davis, University of California, Davis, CA; ^2^UC Davis Health, Sacramento, CA; ^3^UC Davis Health, UC Davis, CA; ^4^UC Davis Health, UC Davis Health, CA*

4:00 PM ‐ 6:00 PM

**Objective**: APO, namely preterm birth (PTB), hypertensive disorders of pregnancy (HDP), and placental abruption have been associated with CHD in singleton pregnancies. This association has not been investigated in twin pregnancies. The aim of this study is to 1) evaluate the prevalence of APO in twin pregnancies affected by CHD using a large national database; 2) identify pregnancy characteristics associated with the APOs using a local database.

**Study Design**: A retrospective case‐control study of twin pregnancies with CHD was conducted on Center of Disease Control and Prevention Natality Database from 1995‐2000 (403,018 births) and 2016‐2022 (842,878 births). Maternal demographics and perinatal outcomes were compared. Subsequently a local twin case series with CHD was reviewed for characteristics leading to APO.

**Results**: Demographically, there was no significant difference in twin pregnancies with or without CHD in both periods The CHD group showed significantly increased PTB: 1995‐2000: < 37 weeks: Odds Ratio [OR], 2.37; (2.04‐2.77 95% Confidence Interval [95%CI]); < 28 weeks: OR, 3.07; (2.54‐3.71 95%CI); 2016‐2022: < 37 weeks: OR, 1.91; (1.64‐2.24 95%CI); < 28 weeks: OR, 2.18; (1.70‐2.80 95%CI). Eclampsia was higher with CHD: 1995‐2000: OR, 3.35; (2.30‐4.89 95%CI); 2016‐2022: OR, 5.32; (3.60‐7.90 95%CI). Other HDP were not significantly increased in either cohort. Local center case series revealed that PTB was due to 6.7% spontaneous and 93.3% iatrogenic (18.2% for maternal indications, 81.8% for fetal indications).

**Conclusion**: CHD is associated with increased odds of PTB and eclampsia in twin pregnancies in CDC database collected in 1995‐2000 and 2016‐2022. Our single center study shed light on the common reasons for PTB in this CHD population. Further characterization with a larger sample size is needed to elucidate the incidence of APOs in twin pregnancies complicated by CHD.

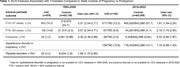

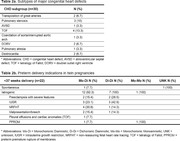

## 1163 | Rate of Tethered Spinal Cord Release After Fetoscopic Repair of Open Spina Bifida

Ramen H. Chmait^1^; Jason K. Chu^2^; Alexander L. Van Speybroeck^3^; HaiThuy N. Nguyen^3^; Arlyn Llanes^1^; Grace Hamadeh^1^; Adam E. Masri^1^; Lisa M. Korst^4^; Eftichia V. Kontopoulos^5^; Ruben A. Quintero^5^; Denise A. Lapa^6^

*^1^Keck School of Medicine, University of Southern California, Los Angeles, CA; ^2^Riley Hospital for Children, Indianapolis, Indianapolis, IN; ^3^Children's Hospital Los Angeles, Los Angeles, CA; ^4^Childbirth Research Associates, North Hollywood, CA; ^5^The Fetal Institute, USFETUS Research Consortium, Miami, FL; ^6^Hospital Israelita Albert Einstein, Hospital Infantil Sabara, Sao Paulo, Sao Paulo*

4:00 PM ‐ 6:00 PM

**Objective**: A recent meta‐analysis reported approximately 22% rate of symptomatic tethered spinal cord (TSC) at 4.1 years median age of follow‐up after prenatal closure of open spina bifida (OSB). TSC was defined as either neurologic deterioration, urological deterioration, or need for a surgical TSC release. The aim of this study was to assess the rate of surgical TSC release in a cohort of patients who underwent fetoscopic repair of OSB, followed between 2.5 and 5 years of life.

**Study Design**: This was a prospective study of patients who underwent percutaneous or percutaneous mini‐laparotomy fetoscopic OSB repair between 24 and 28 gestational weeks and who were followed for 2.5 to 5 years of life. The postnatal evaluation and treatment were at the discretion of the referring team and based on clinical findings and symptoms.

**Results**: Of 105 consecutive patients who received the fetoscopic OSB repair and achieved 30 months of age, 6 (5.7%) underwent a surgical TSC release procedure. Table 1 shows specific details of these 6 cases. Four of these 6 patients developed symptomatic hydrocephalus and were treated with a shunt or endoscopic third ventriculostomy, two at the same time of the TSC release. Because of the small number of affected patients, risk factors for receiving a detethering procedure could not be assessed.

**Conclusion**: Of patients who underwent fetoscopic OSB repair and were between 2.5 to 5 years of age, 5.7% underwent a surgical TSC release procedure. As TSC symptomology may not arise until the later childhood or adolescent period, further long term follow up is necessary. Understanding the risk factors for development of symptomatic TSC will be important to optimize the prenatal surgical approach.

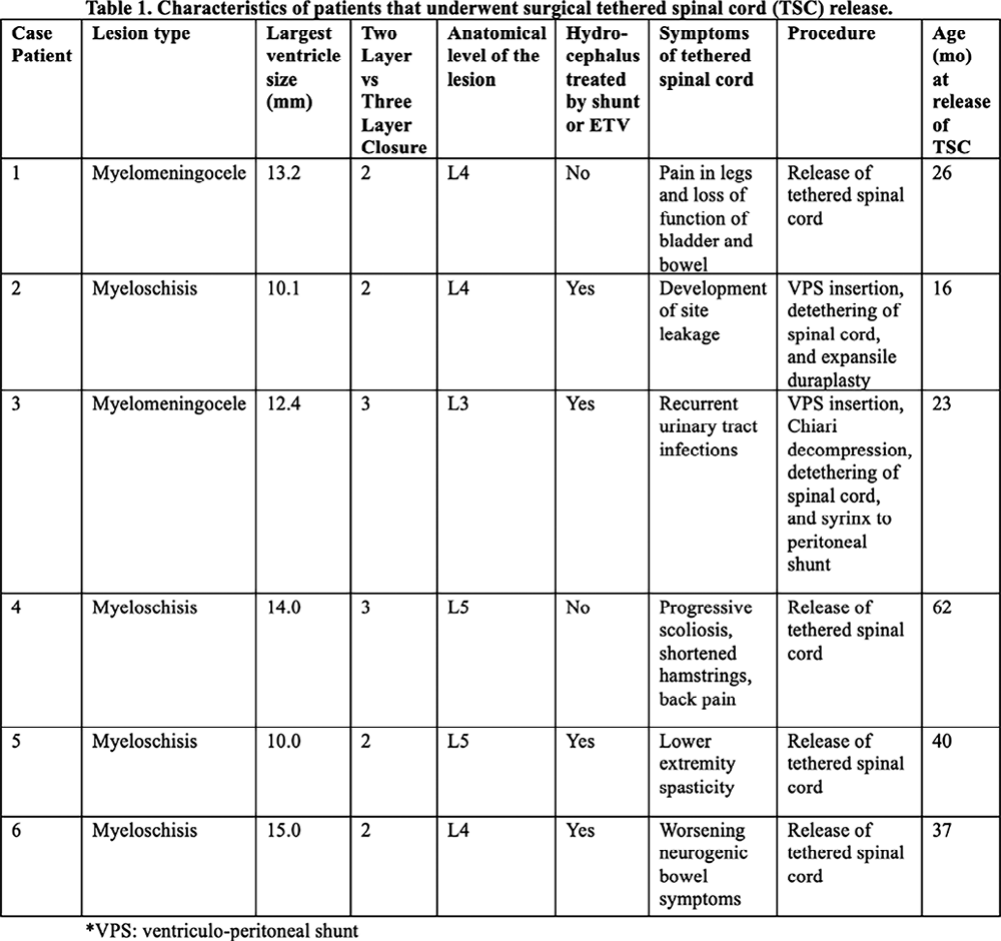

## 1164 | Maternal Outcomes of First‐ Versus Second‐Line Intrapartum Antibiotic Prophylaxis for Group B Streptococcus: A Retrospective‐Study

Raneen Abu Shqara^1^; Shany Or^2^; Lior Lowenstein^3^; Maya Frank Wolf^4^

*^1^Galilee Medical Center, Nahariya, HaZafon; ^2^Bar‐Ilan univeristy, Bar‐Ilan university, HaZafon; ^3^Galilee Medical center, Nahariya, HaZafon; ^4^Galilee Medical Center, Naharyia, HaZafon*

4:00 PM ‐ 6:00 PM

**Objective**: The incidence of maternal infectious morbidity according to the intrapartum antibiotic prophylaxis (IAP) regimen used for Group B streptococcus (GBS) prophylaxis is poorly described. We aimed to compare maternal peripartum infections and microbiological outcomes among GBS carriers according to IAP regimen.

**Study Design**: This retrospective study included women hospitalized in a tertiary university‐affiliated hospital between March 2021 and March 2024, who were GBS positive at term and received first‐line ampicillin (n = 1833), or second‐line clindamycin (n = 78) due to penicillin allergy. The co‐primary outcomes were clinical chorioamnionitis and neonatal intensive care admission rates. Univariate and multivariate analyses were performed. Chorioamniotic swabs were obtained after delivery, and microbiological outcomes were compared according to IAP regimen.

**Results**: Among GBS carriers who received clindamycin vs. ampicillin, the rates were higher of chorioamnionitis (p< 0.001), intrapartum fever (p< 0.001), postpartum fever (p = 0.001) and antibiotics use (p< 0.001) (Figure). The cesarean delivery rates were similar (p = 0.086). The rate of neonatal intensive care admission and other neonatal complications were similar. In multivariate analysis, the factors that were independently associated with clinical chorioamnionitis were: clindamycin‐IAP, odds ratio (OR) 20.1, 95% confidence interval (CI) (6.6‐61.4), p< 0.001; cervical ripening by catheter balloon, OR 4.1, 95%CI (1.2‐13.6), p = 0.023; and prolonged rupture of membranes, OR 4.9, 95%CI (1.6‐15.7), p = 0.006 (Table). In chorioamniotic swabs, among women who received clindamycin vs. ampicillin, rates were higher of positive GBS isolates, 28% vs. 11%, p< 0.001 and of overall positive cultures, 32.1% vs. 16.4%, p< 0.001.

**Conclusion**: Clindamycin compared with ampicillin was associated with a higher rate of maternal peripartum infectious morbidity.

This underscores the importance of minimizing clindamycin use for GBS prophylaxis. Further research is warranted to evaluate alternative second‐line IAP options and their effectiveness in preventing GBS‐related infections.

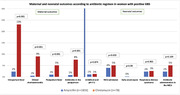

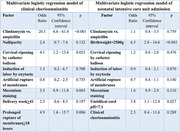

## 1165 | Contraceptive Counseling and use Among Pregnant Individuals who Underwent Spontaneous Versus Medically Indicated Preterm Birth

Rebecca Purvis^1^; Alicia Mastronardi^2^; Megan L. Young^3^; William S. Havron, IV^4^; Jill M. Maples^5^; Nikki B. Zite^4^

*^1^University of Tennessee Medical Center Knoxville, Knoxville, TN; ^2^University of Tennessee Graduate School of Medicine, Department of OB/Gyn, Knoxville, TN; ^3^University of Tennessee Medical Center, Center for Women and Infants, Knoxville, TN; ^4^University of Tennessee Graduate School of Medicine, Knoxville, TN; ^5^University of Tennessee Health Science Center, College of Medicine, Knoxville, TN*

4:00 PM ‐ 6:00 PM

**Objective**: Preterm birth (PTB) contributes to maternal and neonatal morbidity. Adequate contraceptive uptake can help reduce rates of PTB, however, individuals who undergo PTB are less likely to obtain contraception than those who undergo term delivery. We sought to describe rates of contraceptive uptake and patient reported recollection of contraceptive counseling in our population who underwent PTB, as stratified by etiology.

**Study Design**: This retrospective cohort study examined all liveborn PTBs within a single regional academic medical center between January 2023‐April 2024. Demographic and contraceptive data was collected via EMR and confirmed through billing data. Etiology of preterm birth was abstracted from the medical record and categorized by a single physician. Chi‐square, t‐test, and Mann‐Whitney U were used to compare populations and outcomes, as appropriate.

**Results**: Of the 6,058 total births at our institution during the study, 934 (15.4%) delivered in the preterm period (< 37 weeks) and were analyzed. Of these, 426 (45.6%) were considered spontaneous (SPTB) and 508 (54.4%) were considered medically indicated (IPTB). Demographic information did not significantly differ between SPTB vs IPTB, other than median gestational age at delivery (34.6 weeks vs 35.3 weeks, p = .002) and limited/no prenatal care (11.3% vs 5%, p< .001). Those who underwent IPTB were more likely to recall contraceptive counseling (41.7% vs 30%, p< .001) and to identify their contraceptive desires at discharge (84.5% vs 77.9%, p = 0.24). Similar trends are present when stratifying each type of preterm birth by above and below 32 weeks at time of delivery (Table 2).

**Conclusion**: Pregnant individuals who undergo IPTB are more likely to recall contraceptive counseling and to identify their contraceptive desires at discharge than those who undergo SPTB. Future research will focus on identifying reasons why those who undergo IPTB vs SPTB recall differences in contraceptive counseling, given the importance of contraception in helping to reduce the risk of recurrent PTB.

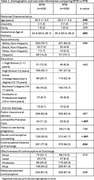

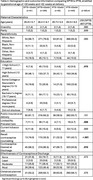

## 1166 | First Trimester Machine Learning to Predict Preeclampsia in Normotensive Pregnancies by American Heart Association Guidelines

Rebecca Horgan^1^; Erkan Kalafat^2^; Elena Sinkovskaya^1^; Alfred Z. Abuhamad^1^; George R. Saade^1^

*^1^Macon & Joan Brock Virginia Health Sciences Eastern Virginia Medical School at Old Dominion University, Norfolk, VA; ^2^Koc University Hospital, Istanbul, Istanbul*

4:00 PM ‐ 6:00 PM

**Objective**: The threshold to define normal blood pressure (BP) outside pregnancy have been lowered. We assessed if machine learning can identify patients at high risk of hypertensive disorders of pregnancy (HDP) amongst normotensive first‐trimester patients per American Heart Association (AHA) guidelines.

**Study Design**: This was a prospective cohort study which enrolled patients at ≤ 13+6 weeks’ gestation without fetal, umbilical cord or placental abnormalities. For this analysis, patients with chronic hypertension were excluded. Baseline maternal BP was classified according to the AHA guidelines (normal, elevated, stage I hypertension, stage II hypertension). The primary outcome was the incidence of HDP diagnosed per ACOG criteria. First trimester BP values were clustered using unsupervised machine learning models and patients were categorized as; AHA normal BP, low risk clustering; AHA normal BP, high risk clustering and AHA abnormal BP (elevated or above). Association with outcomes were assessed with regression analyses, which were adjusted for race, body‐mass index, and risk factors for preeclampsia (PE).

**Results**: 570 patients were included in this analysis, with 43 developing PE and 73 any HDP. After clustering, 350 patients fell within the AHA normal BP low‐risk cluster, 28 AHA normal BP high‐risk cluster and 192 patients AHA abnormal BP (elevated or above). The PE rates in these categories were 3.1%, 25.0% and 13.0%, respectively (P< 0.001). The rate of small for gestational age (SGA) were 6.6%, 21.4% and 6.8% respectively (P = 0.032). The hazard of PE was higher in patients with AHA normal BP, high risk clustering (adjusted HR: 8.51, 95% CI: 3.30‐22.0, P< 0.001) compared to AHA abnormal BP (adjusted HR: 4.33, 95% CI: 2.12‐8.84, P< 0.001) (Figure). Similar findings were observed for any HDP (adjusted HR:4.15 vs 5.77, P< 0.001 both, Figure).

**Conclusion**: A cohort of patients with AHA normal BP, identified by unsupervised machine learning models, had significantly higher risk of preeclampsia and neonatal SGA than those with AHA abnormal BP or AHA normal BP clustered in the low‐risk group.

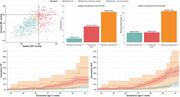

## 1167 | Preeclampsia Associated Morbidity Requires a Double hit from First‐Trimester Maternal Characteristics and Placental Dysfunction

Rebecca Horgan^1^; Erkan Kalafat^2^; Elena Sinkovskaya^1^; Alfred Z. Abuhamad^1^; George R. Saade^1^

*^1^Macon & Joan Brock Virginia Health Sciences Eastern Virginia Medical School at Old Dominion University, Norfolk, VA; ^2^Koc University Hospital, Istanbul, Istanbul*

4:00 PM ‐ 6:00 PM

**Objective**: To evaluate whether unsupervised machine learning clustering of first‐trimester data can identify pregnancies at high risk for preeclampsia associated morbidity.

**Study Design**: This is a prospective cohort study which enrolled patients at ≤ 13+6 weeks’ gestation without fetal, umbilical cord or placental abnormalities. Baseline maternal characteristics, blood pressure, and first trimester ultrasound including uterine and placental artery (spiral artery and intervillous arteriole) Dopplers were obtained. The primary outcome was the prevalence of preeclampsia with associated morbidity defined as delivery at < 34 weeks, eclampsia, HELLP syndrome or placental abruption. Patients were followed throughout pregnancy and outcomes collected by trained research coordinators. Maternal characteristics (BMI and blood pressure) and uterine & placental Dopplers (uterine, spiral, and intervillous artery pulsatility index) were used to create their distinct risk groups using unsupervised machine learning models. Outcomes of patients who were labeled as high risk in both (maternal characteristics and Doppler) or just one (Maternal characteristics or Doppler) were compared using cox‐proportional hazard models.

**Results**: Analysis included 617 patients. Machine learning identified 164 patients as high risk of preeclampsia by both maternal characteristics and Dopplers, 126 by maternal characteristics alone, 161 by Dopplers alone, and 166 as low risk. Preeclampsia rates were highest in the dual high‐risk group (17.1%), compared to maternal characteristics alone (13.5%) or Dopplers alone (7.5%) (HR: 1.80, 95% CI: 1.07‐3.03, P = 0.026). The low‐risk group had a 2.4% preeclampsia rate. 70% of preeclampsia cases with associated morbidity were in the dual high‐risk group, significantly higher than those identified by only one criterion (HR: 4.15, 95% CI: 1.07‐16.0, P = 0.039).

**Conclusion**: Preeclampsia with associated morbidity mostly occurs in patients with abnormalities in both first trimester blood pressure values and placental Doppler values.

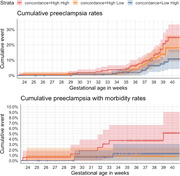

## 1168 | Clinical Factors Associated with Survival of Periviable Rupture of Membranes

Rebecca Crowe^1^; Eliza R. McElwee^2^; Jessica Pittman^1^; Devin Hatchell^2^

*^1^Medical University of South Carolina, Charleston, SC; ^2^Medical University of South Carolina, Columbia, SC*

4:00 PM ‐ 6:00 PM

**Objective**: The aim of this study is to assess neonatal survival to discharge and factors that may influence survival in pregnancies affected by rupture of membranes (ROM) less than 25 weeks gestation (w).

**Study Design**: This is a retrospective cohort study of non‐anomalous, singleton pregnancies with ROM prior to 25w delivered at a tertiary care center from 2005‐2024. Patients included in the study elected for expectant management at the diagnosis of ROM and delivered a liveborn neonate that was a candidate for resuscitation. Patients who elected comfort care were excluded. Patients were stratified by neonatal survival to discharge. Maternal, obstetric, and neonatal variables were compared between survival groups. Chi‐square and student's t‐test were used to compare categorical and continuous variables.

**Results**: Of 90 patients with ROM less than 25 w, 54 (60%) neonates survived to discharge, and 36 (40%) had a neonatal demise. Gestational age (GA) at ROM (23.2w IQR 22.6‐24 v 23.1w IQR 22.5 ‐ 24, p = 0.318), GA at delivery (24.4w IQR 23.7‐24.9 v 24.2w IQR 23.6‐24.9, p = 0.197), and latency from ROM to delivery (4.6d (IQR 2‐16) v 4 (IQR 2‐16), p = 0.623) were not statistically different between survivors and non‐survivors. Six percent of patients delivered prior to 23w0d. Residual amniotic fluid values were not statistically different between survivors and non‐survivors (p = 0.42). Antenatal interventions, including antenatal corticosteroid (100% v 94.4%, p = 0.157) and antibiotic administration (90.7% v 83.3%, p = 0.337) were not different between groups. Mode and indication for delivery did not impact neonatal survival to discharge.

**Conclusion**: In our cohort, overall survival of expectantly managed pregnancies affected by ROM less than 25 weeks gestation is 60%. Survival to discharge is multi‐factorial, and is challenging to predict in the periviable period.

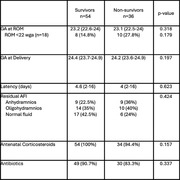

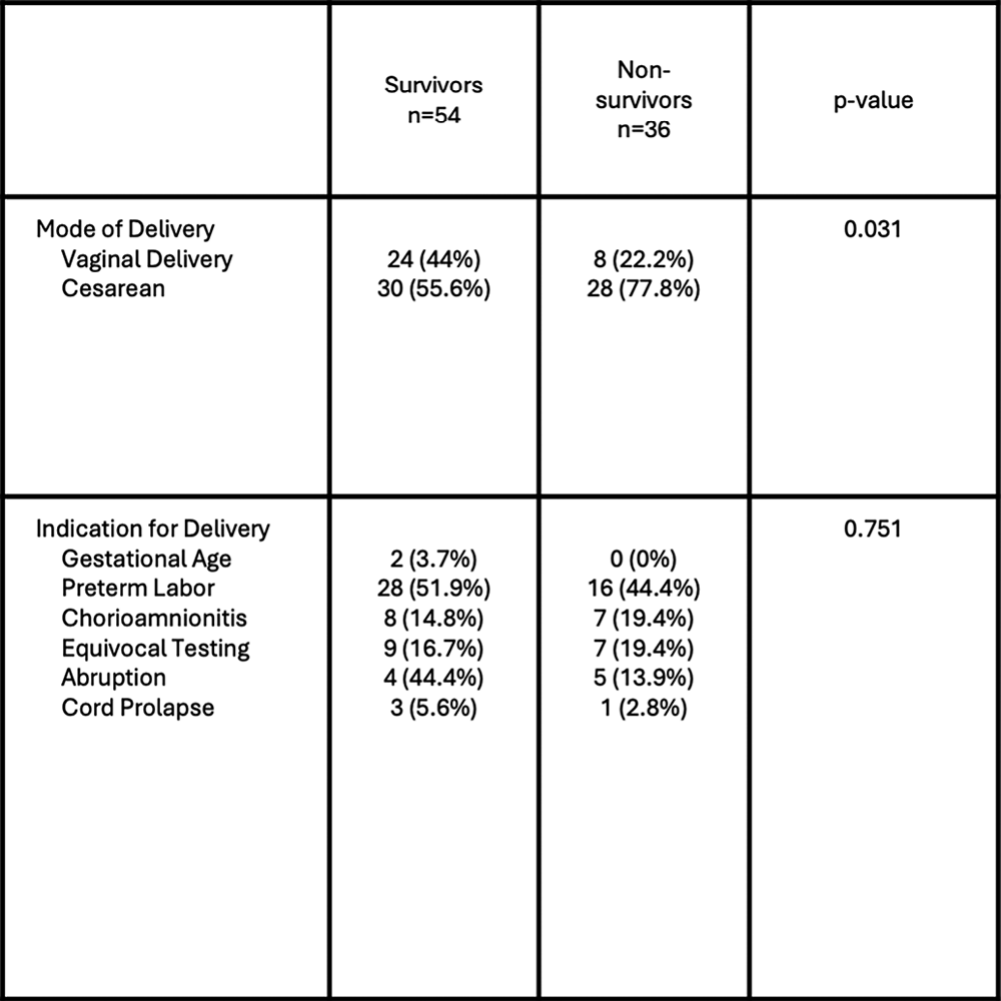

## 1169 | Impact of Gestational Age on Outcomes in Periviable Rupture of Membranes

Rebecca Crowe^1^; Eliza R. McElwee^2^; Jessica Pittman^1^; Devin Hatchell^2^; Kelli M. McFarling^2^

*^1^Medical University of South Carolina, Charleston, SC; ^2^Medical University of South Carolina, Columbia, SC*

4:00 PM ‐ 6:00 PM

**Objective**: The aim of this study is to evaluate neonatal survival to discharge and neonatal outcomes based on gestational age of rupture of membranes (ROM) in pregnancies affected by ROM less than 25 weeks gestation (w).

**Study Design**: This is a retrospective cohort study of non‐anomalous, singleton pregnancies with ROM prior to 25w delivered at a tertiary care center from 2005‐2024. Patients included in the study elected for expectant management at the diagnosis of ROM and delivered a liveborn neonate that was a candidate for resuscitation. Patients who elected comfort care were excluded. Patients were stratified by gestational age at ROM (early ROM less than 22w, late ROM greater than 22w). The primary outcome was neonatal survival to discharge; secondary outcomes included neonatal variables amongst survivors. Chi‐square and student's t‐test were used to compare categorical and continuous variables.

**Results**: Of 90 patients with ROM less than 25w, 18 (20%) had early rupture and 72 (80%) had late ROM. Overall survival to discharge in the study group was 60% (n = 54). There was no statistical difference in survival to discharge when comparing early versus late ROM (44.4% v 63.9%, p = 0.132). Gestational age at delivery was not significantly different between early and late ROM groups (24w3d IQR 23w0d‐27w5d v 24w2d IQR 23w5d‐24w5d); however, the early ROM group did have greater latency to delivery (6.14 days v 0.4 days, p< 0.01). Six percent of patients delivered before 23 w. All neonates were affected by respiratory distress syndrome; remaining neonatal outcomes including bronchopulmonary dysplasia, intraventricular hemorrhage, retinopathy of prematurity, necrotizing enterocolitis, and sepsis were similar between groups.

**Conclusion**: In our cohort, gestational age at ROM did not affect neonatal survival to discharge. Patients with ROM less than 22w have greater latency to delivery, but no difference in gestational age at delivery or survival.

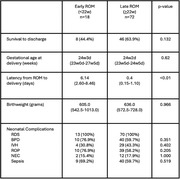

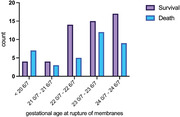

## 1170 | Machine Learning Algorithm for the Point of Care Prediction of Preterm Labor

Reetam Ganguli^1^; Stephen Wagner^2^

*^1^Elythea, San Jose, CA; ^2^Beth Israel Deaconess Medical Center, Boston, MA*

4:00 PM ‐ 6:00 PM

**Objective**: Preterm birth is the leading cause of neonatal mortality and is 25‐fold more expensive than term deliveries to payors. Relevant literature has demonstrated that clinical intervention indicated by early risk screening tests can reduce preterm birth occurrence by 20% and can save over $800 per patient. Current screening methods to detect patients at risk of preterm labor are limited with some studies suggesting established protocols such as cervical length screening have a sensitivity as low as 11%. Our goal was to develop a machine learning model to non‐invasively predict risk of preterm birth at the point of care.

**Study Design**: We conducted a cohort study utilizing sociodemographic and clinical data obtainable in the 1st trimester from the CDC Vital Statistics System. Deliveries with missing preterm labor data and deliveries in hospitals not reporting preterm labor were excluded. The primary outcome was spontaneous preterm birth < 37 weeks.

Models were trained on years 2018‐2020 of the CDC Vital Statistics System and were tested on a hold‐out set of year 2021.

**Results**: 40 clinical variables for 12,476,992 obstetric patients were included in the training data. 2,685,095 (21.5%) patients had a preterm birth.

The model was tested on 3,666,801 patients, of which 450,318 (12.3%) patients went into preterm labor. The developed model had an area under the receiver operating characteristic curve of 0.73, sensitivity of 0.65, and F1 score of 0.68.

The highest weighted factors in the model were: number of prenatal visits, trimester that prenatal care was initiated in, and interval since last live birth.

**Conclusion**: Our developed ML model leveraging data available at early stages of pregnancy harbors the potential to identify women at increased risk for spontaneous preterm birth at nearly 6 times the sensitivity than existing cervical length measurement tests as soon as the point of care and may allow for targeted interventions to reduce spontaneous preterm labor incidence/lower healthcare expenditures related to preterm birth.

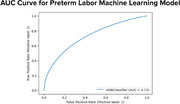

## 1171 | Maternal Hypertension Care Bundle Implementation at an Academic Tertiary Hospital: System Redesign using Improvement Science

Isabella Toledo^1^; Joyce H. Xu^2^; Lori Fannin^2^; Mary Giles^2^; Heather N. Czarny^2^; Stephen Afflito^3^; Muhammad Zafar^2^; Robert M. Rossi^2^; On behalf of the Maternal Safety Quality Improvement Initiative of the Ohio Department of Health

*^1^Indiana University School of Medicine, Indianapolis, IN; ^2^University of Cincinnati College of Medicine, Cincinnati, OH; ^3^Ohio Colleges of Medicine, Government Resource Center, Columbus, OH*

4:00 PM ‐ 6:00 PM

**Objective**: Uncontrolled hypertension in pregnancy is a major cause of maternal morbidity and mortality. Application of best‐practice maternal hypertension care bundle reduces severe maternal morbidity. Our objective was to implement an in‐patient, maternal hypertension bundle (HTN‐bundle) with reliable adherence of >80% in an academic tertiary care hospital.

**Study Design**: University of Cincinnati Medical Center is a level IV maternal care academic center, safety net hospital. We participated in the AIM Hypertension Quality Improvement Project (AIM‐QIP) by the Ohio Colleges of Medicine Government Resource Center.

The HTN‐bundle consisted of 1) treatment of systolic blood pressure (BP) or diastolic BP ≥ 160/110 in < 60 min, 2) appropriate medication use for acute HTN treatment, 3) patient education, 4) timely discharge follow‐up, 5) blood pressure cuff provision. We aimed to improve the adherence to bundle components from 62% (baseline) to >80% within 6‐months.

Staff education was led by the medical director and facilitated by AIM‐QIP. We performed detailed process maps, Pareto analyses, empathy mapping, and Go‐see activities to identify improvement opportunities. Interventions for redesign were testing using iterative Plan‐Do‐Study‐Act (PDSA) cycles (Fig 1). Bundle adherence was tracked by chart reviews and plotted on run‐chart.

**Results**: The baseline HTN‐bundle adherence was 62%. We performed 33 PDSA cycles, testing 16 interventions. By 6‐months, HTN‐Bundle adherence reached 84% with a sustainable system shift on the run‐chart (Fig 2). The highest increases were noted in provision of BP cuffs and 3‐day follow‐up BP check appointments. The HTN‐bundle delivery now relies on different team members performing inter‐dependent task, making it a system property rather than an individual's responsibility.

**Conclusion**: Using improvement science methods, adherence to HTN‐bundle can be made a part of reliable, standard‐of‐care for all affected patients. Key success factors were setting a clear aim, team engagement, QI expertise, data‐guided change efforts, and availability of resources.

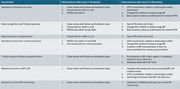

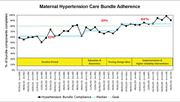

## 1172 | Xenobiotic Transfer of Tranexamic Acid Through an ex vivo Placental Perfusion Model

Roshini Zachariah^1^; Helen Zhao^2^; Andrea D. Shields^3^; Richard Wagner^1^

*^1^University of Connecticut Health Center, Farmington, CT; ^2^University of Connecticut, West Hartford, CT; ^3^University of Connecticut Health, Avon, CT*

4:00 PM ‐ 6:00 PM

**Objective**: In obstetrics, intravenous tranexamic acid (TXA) is used to treat postpartum hemorrhage via inhibition of fibrinolysis and is effective in reducing mortality from postpartum hemorrhage. TXA is not currently approved for prophylactic use of postpartum blood loss but existing meta‐analyses suggest it may reduce the incidence of postpartum hemorrhage and overall blood loss. However, data on use of TXA for antepartum bleeding is limited.

**Study Design**: We used an *ex vivo* placental perfusate model to study the rate of transfer of TXA across the placental interface. Placentas from term uncomplicated singleton pregnancies were obtained immediately after scheduled cesarean deliveries. A fetal vein‐artery pair supplying a cotyledon was isolated, cannulated and perfused. Maternal circulation was also established through placental storm. After addition of TXA and antipyrine (positive control) to the system, fetal samples were collected every 15 minutes for 3 hours with two parallel closed circulations (maternal and fetal). Fetal artery pressure and pH of the maternal and fetal reservoir were monitored every 15 minutes to continuously assess the integrity of the model. Fetal vein samples were analyzed via HPLC to quantify TXA and antipyrine concentrations.

**Results**: TXA rapidly crosses the placental interface within 15 minutes of administration. TXA levels in the fetal vein rose over 3 hours and was modeled with a logarithmic curve. The predicted final fetal vein concentration was 250 µM. The *in vivo* fetal concentration would likely be lower due to increased maternal circulating volume and renal clearance. Based on transfer seen in this *ex vivo* model, we would predict an *in vivo* fetal vein concentration of 7 µg/mL (clinical effects are seen at 10‐15 mg/mL).

**Conclusion**: Tranexamic acid rapidly crosses the placental interface in an ex vivo model. However, the fetal vein concentration is reduced compared to maternal concentration. This suggests that in vivo use may be ethically safe to test as expected fetal concentrations would be below the clinically effective threshold.

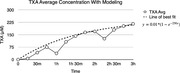

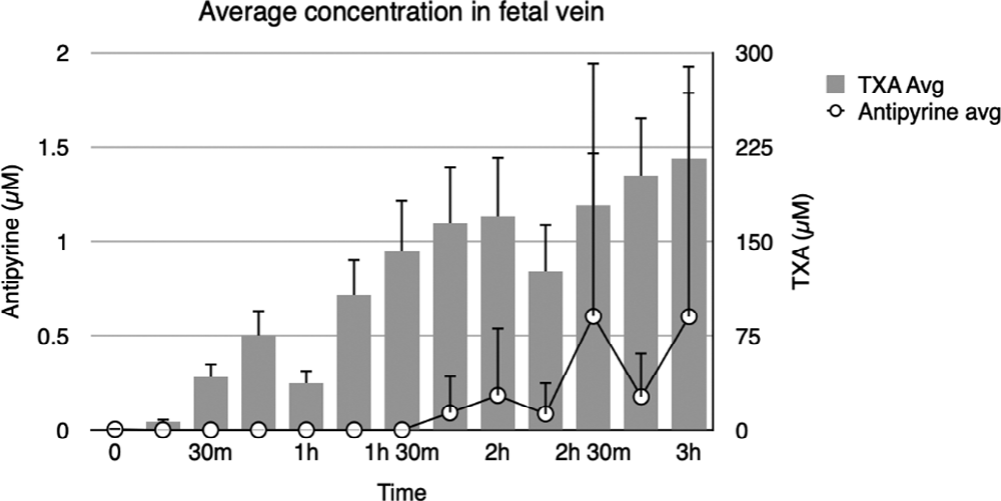

## 1173 | Amlodipine Versus Nifedipine ER for Managing Postpartum Hypertension: Medication Continuation Beyond 6 weeks Postpartum

Ross F. Lordo^1^; Alondra DeSantiago^2^; Zachary C. Travis^2^; Himashreya Katti^2^; Stella Self^3^; Laura Carlson^4^; Katelyn Pratt^4^

*^1^Prisma Health Upstate, Greenville, SC; ^2^University of South Carolina School of Medicine Greenville, Greenville, SC; ^3^University of South Carolina Arnold School of Public Health, Columbia, SC; ^4^Prisma Health Upstate/University of South Carolina School of Medicine Greenville, Greenville, SC*

4:00 PM ‐ 6:00 PM

**Objective**: Despite being a leading cause of maternal morbidity, data on optimal management of postpartum (PP) hypertension (HTN) is limited. Our parent study demonstrated noninferiority of amlodipine for treatment of PP HTN in the immediate PP period when compared to nifedipine ER. In this secondary analysis, we sought to assess use of antihypertensives beyond 6 weeks PP. We hypothesized that the majority of patients using either medication would discontinue treatment prior to 6 weeks PP and that of those who continued treatment, participants assigned to amlodipine would be more likely to continue the assigned study drug than those assigned to nifedipine ER, as use of amlodipine is more common in the non‐obstetric setting.

**Study Design**: This was a secondary analysis of a randomized controlled noninferiority trial. 175 patients were enrolled in the parent study, with 120 patients started on either amlodipine or nifedipine ER in the per protocol cohort (n = 51 amlodipine, 69 nifedipine ER). Charts of the per protocol cohort were abstracted to assess continuation of the initially assigned antihypertensive at 6 weeks, 12 weeks, and 12 months PP. Data was analyzed utilizing chi‐squared and Fishers exact test.

**Results**: Overall, forty percent of patients remained on antihypertensives at 6 weeks PP. No significant differences were found in rates of continuation of any antihypertensive at 6 weeks, 12 weeks, or 12 months PP. Those assigned to amlodipine were significantly more likely to be continued on their assigned study medication at 6 weeks and 12 weeks, but not 12 months, PP (Table 1).

**Conclusion**: While the majority of patients discontinue antihypertensives by 6 weeks PP, some patients with hypertensive disorders of pregnancy will require prolonged treatment. In patients requiring prolonged treatment, patients assigned to amlodipine were significantly less likely to be changed to an alternative antihypertensive. Although this was not statistically significant at 12 months PP, this may be due to sample size. Our findings support that amlodipine should be considered for treatment of postpartum hypertension.

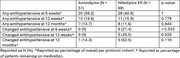

## 1174 | Impact of Chorioamnionitis on Maternal and Neonatal Outcomes Categorized by Presence or Absence of Fever

Rula Atwani; George R. Saade; Tetsuya Kawakita

*Macon & Joan Brock Virginia Health Sciences Eastern Virginia Medical School at Old Dominion University, Norfolk, VA*

4:00 PM ‐ 6:00 PM

**Objective**: To examine the impact of a chorioamnionitis diagnosis on maternal and neonatal outcomes categorized by the presence or absence of documented fever.

**Study Design**: We conducted a secondary analysis using data from the Nulliparous Pregnancy Outcomes Study: Monitoring Mothers‐to‐Be. We excluded individuals who delivered before 20 weeks' gestation and those with missing chorioamnionitis data, then categorized participants based on the diagnosis of chorioamnionitis with versus without fever. Data was obtained prospectively by trained research coordinators. Our primary outcome was the neonatal composite outcome including suspected or confirmed neonatal sepsis, necrotizing enterocolitis, respiratory distress/transient tachypnea of the newborn (RDS/TTN), seizure, hypoglycemia, and hyperbilirubinemia. We also examined maternal outcomes (cesarean delivery, endometritis, and postpartum hemorrhage). We calculated adjusted relative risks (aRRs) with 95% confidence intervals (95% CIs) using modified Poisson regression.

**Results**: Of 9,432 individuals, 574 (6.1%) had chorioamnionitis with fever and 46 (0.5%) had chorioamnionitis without fever. Proportions and relative risks of outcomes are present in Tables 1 and 2. Compared to individuals with no chorioamnionitis, chorioamnionitis without fever was associated with higher risks of neonatal composite outcomes, neonatal sepsis, RDS/TTN cesarean delivery and postpartum hemorrhage. Compared to individuals with no chorioamnionitis, chorioamnionitis with fever was associated with higher risks of neonatal composite outcomes, neonatal sepsis, RDS/TTN, hypoglycemia, cesarean delivery, endometritis; and lower risk of hyperbilirubinemia.

**Conclusion**: Chorioamnionitis is associated with adverse maternal and neonatal outcomes regardless of fever, supporting ACOG's recommendations for diagnosing intraamniotic infections without maternal fever.

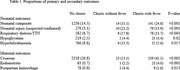

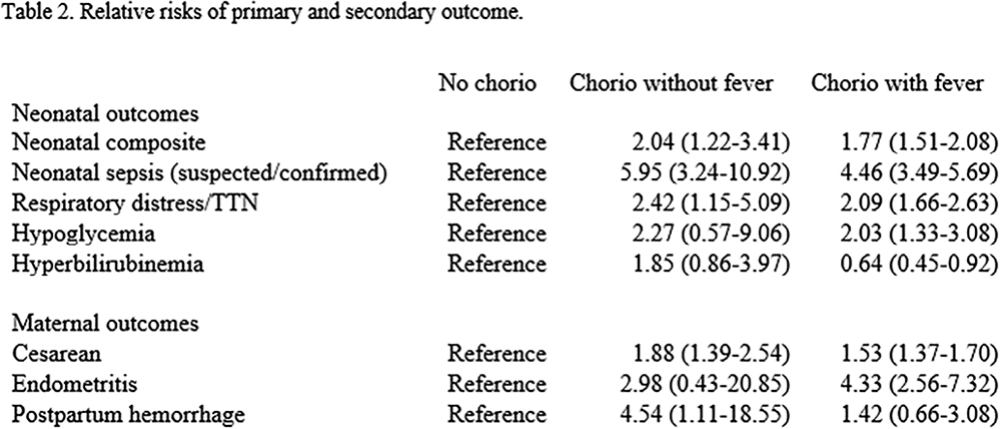

## 1175 | Predicting Gestational Latency Using Serial Monitoring Among Nulliparous, Singleton Pregnancies with low‐Normal Mid‐Trimester Cervical Lengths

Sandhya Chandrasekaran^1^; Vanya Manthena^2^; Andrew Rausch^2^; Ryan E. Longman^2^; Ashish Premkumar^2^

*^1^University of Chicago School of Medicine, Chicago, IL; ^2^Pritzker School of Medicine, University of Chicago, Chicago, IL*

4:00 PM ‐ 6:00 PM

**Objective**: Preterm births account for approximately 70% of neonatal deaths and 36% of infant deaths. In this setting, cervical length screening is of utmost importance as a predictor of preterm birth. Nulliparous patients pose a particularly difficult challenge with respect to counseling reading cervical insufficiency given their lack of pregnancy history. We sought to explore predictive trends in serial CLs associated with preterm birth in this otherwise low‐risk population.

**Study Design**: We performed a retrospective case control analysis of all nulliparous, singleton pregnancies from 2021 to 2023 who underwent an anatomy scan with an initial low‐normal cervical length (CL) >25mm and < 30mm followed by at least one serial CL with available gestational age at the time of delivery. The primary outcome was gestational latency (GL), defined as time of diagnosis of low‐normal cervical length to delivery. Trajectory analysis was performed to gauge CL changes as a function of time from delivery between the two groups, using linear regression models. Bivariate analyses were performed.

**Results**: 30 nulliparous, singleton pregnancies with initial low‐normal CL were evaluated, with 23 resulting in term deliveries (“tb”, > = 37 weeks) and 7 resulting in preterm deliveries (“ptb”, < 37 weeks, Table). When plotting serial CLs as a function of time, CL in the “tb” controls generally increased with GL (slope: 0.05), while CLs in the “ptb” controls demonstrated a modest decrease with GL (slope: ‐0.12). Importantly, there was a high degree of individual variability in CL trajectories among those whose pregnancies resulted in preterm deliveries (Figure).

**Conclusion**: 23% of individuals with a low‐normal cervix will deliver preterm. However, high individual variability exists across groups. These data point to the use of serial monitoring in the setting of low‐normal cervix and the need for further work into deciphering individuals factors contributing to these differences to drive novel interventions aimed at stabilizing the cervix with the goal of minimizing risk of preterm birth.

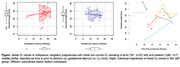

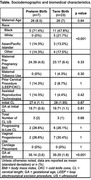

## 1176 | COVID‐19 Vaccine Uptake Amongst Adult Women of Reproductive Age in the United States

Sandra O. Anazor^1^; Samuel Tundealao^2^; Valentine C. Nriagu^3^; Ezinne J. Ugwoke^4^; Hezborn Magacha^5^; Chisom Nwaneki^6^; Nnamdi J. Omenuko^7^; Francis T. Okeke^8^

*^1^Corewell Health West/Michigan State University, Grand Rapids, Michigan., Grand Rapids, MI; ^2^Rutgers University, New Jersey, Rutgers University, NJ; ^3^Maimonides Medical Center, Brooklyn, NY; ^4^Montefiore St. Luke's Cornwall, Newburgh, NY; ^5^East Tennessee State University, Johnson City, TN; ^6^Saint Peter's Healthcare System, New Jersey., Jersey City, NJ; ^7^University of Chicago, Chicago, IL; ^8^Oklahoma University Health Science Center, Tulsa, OK*

4:00 PM ‐ 6:00 PM

**Objective**: To evaluate COVID‐19 vaccine uptake rate and the association between COVID‐19 vaccine uptake and race/ethnicity and, education in women of reproductive age in the United States.

**Study Design**: A secondary data analysis was conducted using United States COVID‐19 Vaccine Hesitancy Survey dataset from the OpenICPSR database. The final sample size was 681 for females aged 18 to 49 years. Descriptive statistics (unweighted and weighted) frequencies and percentages, multivariable and adjusted bivariate logistic regression analyses were obtained for COVID‐19 vaccine uptake, race/ethnicity, age, educational achievement, urbanicity, and chronic disease status.

**Results**: The COVID‐19 vaccine uptake rate was found to be 32.2%. The population consisted of individuals from different racial backgrounds, with the majority identifying as non‐Hispanic White (49.7 %), followed by Hispanic (25.4%), non‐Hispanic Black (14.4%), non‐Hispanic Asian (7.8%), and others (2.9%). The education level varied similarly: Bachelor's degree (36%), associate's or trade school (33%) and high school or less (32%). After adjusting for all predictor variables, only educational achievement of bachelor's degree or higher (AOR: 3.60, C.I: (2.20,5.91), p‐value: < .0001) and urbanicity‐not living in rural area (AOR: 0.55, C.I: (0.37,0.82), p‐value: 0.0031) retained statistical significance in predicting uptake of any COVID‐19 vaccine. Those who had a bachelor's degree or higher were 3.6 times more likely to have received any COVID‐19 vaccine compared to those who had high school education or less (AOR: 3.60, C.I: (2.20,5.91), p‐value: < .0001). There was no statistically significant association between COVID‐19 vaccine uptake and race/ethnicity and age (table 2).

**Conclusion**: This study found statistically significant association between COVID‐19 vaccine uptake and having a bachelor's degree or higher amongst adult women of reproductive age in the United States. Interventions to increase bachelor education can increase COVID‐19 vaccine uptake and other vaccine uptake amongst women of reproductive age in the United States.

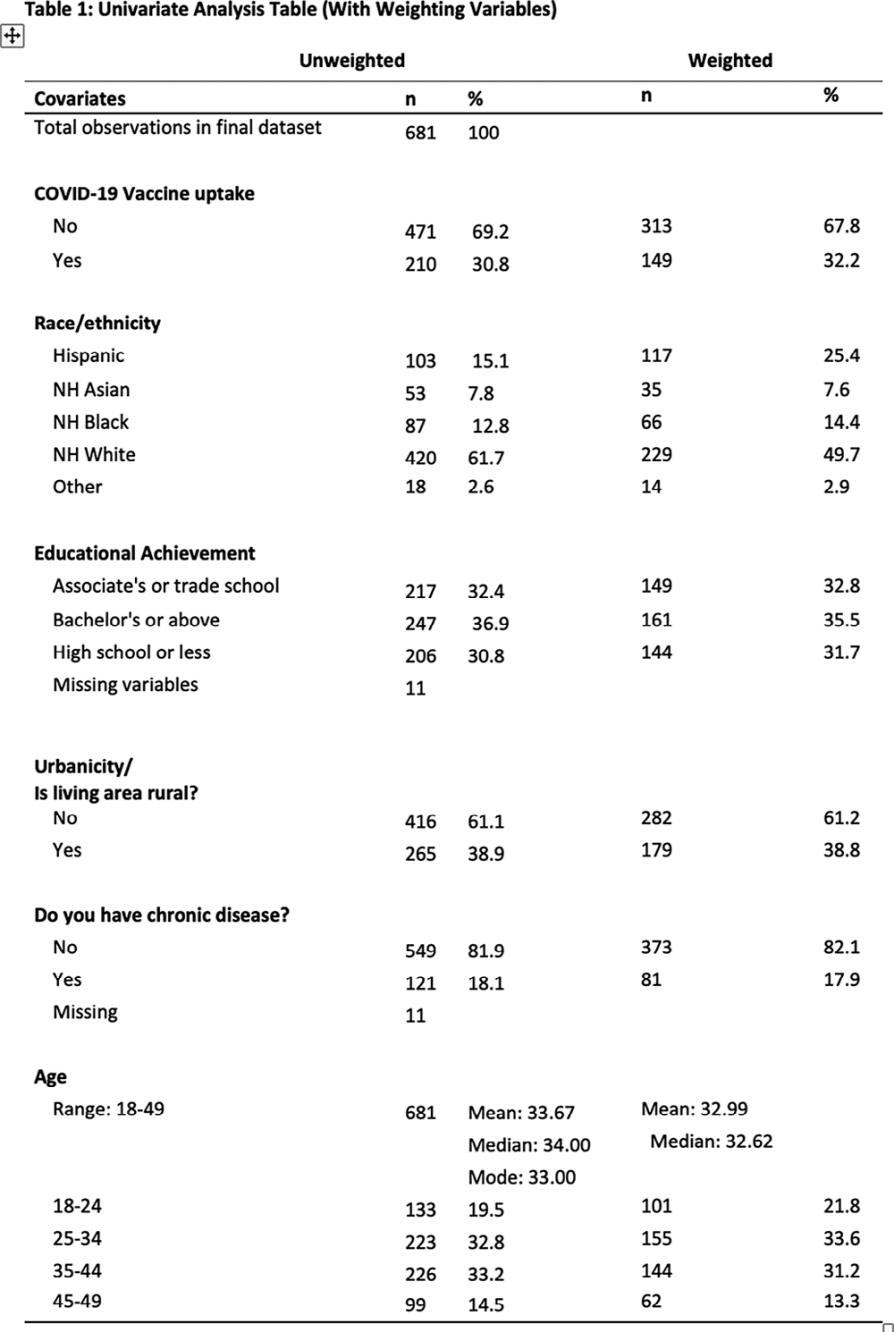

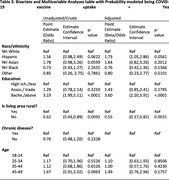

## 1177 | Collaborative Care Model Reduces Perinatal Mood and Anxiety Symptoms

Sarah J. Weingarten^1^; Semra Etyemez^2^; Spencer Darveau^2^; Xiaoyue Ma^2^; Lorena Rincones Rojas^2^; Emily Tutino^2^; Kristin M. Voegtline^2^; Steven Yen^2^; Lauren M. Osborne^2^

*^1^Weill Cornell Medicine at New York Presbyterian, New York, NY; ^2^Weill Cornell Medicine, New York, NY*

4:00 PM ‐ 6:00 PM

**Objective**: Collaborative care (CC) models, which embed mental health care into the outpatient setting, have been shown to improve mental health outcomes in primary care settings, but evidence is still scarce for effectiveness in perinatal settings. We aimed to determine if our CC intervention model would decrease depressive and anxiety symptoms in our perinatal population.

**Study Design**: Patients from three prenatal clinics at Weill Cornell Medicine were systematically screened and those with an Edinburgh Postnatal Depression Scale (EPDS) ≥9 were eligible for referral. EPDS, Perinatal Anxiety Screening Scale (PASS), Generalized Anxiety Disorder Screen (GAD‐7), Adverse Childhood Experiences Questionnaire (ACE) and Client Satisfaction Questionnaire (CSQ‐8) were measured at the intake and discharge visits. Treatment plans included psychotherapy, medication management, or referral to psychiatry for long‐term management. Differences in depression and anxiety scales were compared between the intake and discharge visits.

**Results**: During 1/23/23‐07/24/24, 219 patients completed the CC program. Demographics and patient treatment plans are shown in Table 1. 158 (72.5%) of enrolled patients had a past mental health history. Most patients had current anxiety or obsessive‐compulsive disorder, adjustment disorder, or a depressive disorder at the intake visit. 42 (19.4%) patients were taking psychiatric medications at the intake visit and 33 of those patients (78.6%) were on an SSRI. 72 (33%) were on a medication at time of discharge, with 83.3% being an SSRI. There were significant changes in EPDS, PASS and GAD‐7 scores from intake to discharge visit (p< 0.001) (Table 2). Patients showed high scores on the CSQ‐8, indicating high satisfaction with the CC.

**Conclusion**: A CC program within the OB setting is an effective intervention to reduce perinatal symptoms of anxiety and depression.

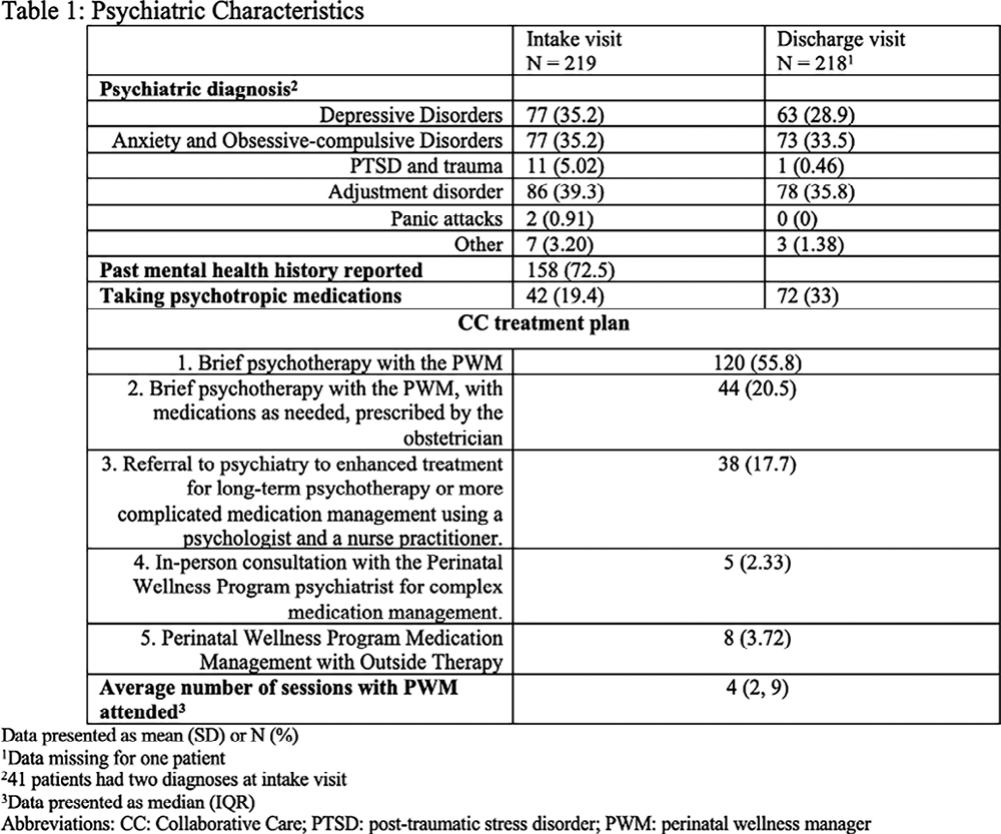

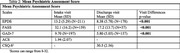

## 1178 | Low‐Fetal Fraction on NIPT: The Role of the 11‐14 week Ultrasound

Sarah J. Weingarten^1^; Alex Raghunandan^2^; Stephen T. Chasen^3^

*^1^Weill Cornell Medicine at New York Presbyterian, New York, NY; ^2^Weill Cornell Medical College, New York, NY; ^3^Weill‐Cornell Medical College, New York, NY*

4:00 PM ‐ 6:00 PM

**Objective**: Low fetal fraction (LFF) on non‐invasive prenatal testing (NIPT) is associated with a higher risk of chromosomal abnormalities. Our goal was to determine the negative predictive value (NPV) of a normal 11‐14 week ultrasound (US) in the setting of LFF on NIPT.

**Study Design**: This was a retrospective review of patients with LFF on first‐trimester NIPT who had an 11‐14 week US from 2021‐2024. Patients with multiple gestations were excluded. Body mass index (BMI), age and estimated gestational age (EGA) at time of NIPT were assessed. Normal genetic outcomes were defined as normal repeat NIPT, normal invasive testing or normal postnatal evaluation. The primary outcome was the negative predictive value of the 11‐14 week US in those with LFF. Fisher's exact test was used for statistical comparison.

**Results**: 179 patients with LFF were included. Five (2.8%) had chromosomal abnormalities including Trisomy 18 (n = 3), Trisomy 21 (n = 1) and Monosomy X (n = 1). Eight patients (4.5%) had an abnormal US: all eight had abnormal nuchal translucency, and two had additional structural abnormalities. 167 patients (93.3%) had a normal repeat NIPT or underwent invasive testing. Of those who underwent repeat NIPT, LFF was found again in 18.3%. Of these patients, one patient had an abnormal genetic result of Monosomy X. There was a strong correlation between abnormal US and chromosomal abnormalities (50% vs. 0.6%; p<  0.001), with four of five affected pregnancies having an abnormal US. The NPV of normal US after LFF result was 99.4% and the sensitivity of the 11‐14 week US was 80%. There was no significant difference in BMI or gestational age at NIPT between those with normal and abnormal outcomes, and rates of obesity were similar in those with normal vs abnormal outcomes (36.1% vs 40%; p = 1.0).

**Conclusion**: In patients with LFF choosing between repeat NIPT vs invasive testing, the 11‐14 week US has high sensitivity and NPV in screening for chromosomal abnormalities. As with all screening tests, patients must be counseled about the potential for false‐negative results.

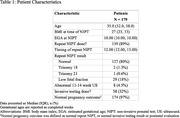

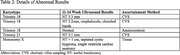

## 1179 | Utero‐Cervical Angle in Standing Position Improves Prediction of Spontaneous Preterm Birth: A Multicenter Cohort Study

Jean‐Charles Pasquier^1^; Isabelle Boucoiran^2^; Marie‐Ève Roy‐Lacroix^3^; Begona Martinez de Tejada Weber^4^; Mi‐Suk Kang Dufour^5^; Sarah Bilodeau^6^; Emmanuel Bujold^7^

*^1^Research Centre of Centre hospitalier de l'Université de Montréal, Montreal, PQ; ^2^University of Montreal, Montreal, PQ; ^3^Université de Sherbrooke, Sherbrooke, PQ; ^4^Hopitaux Universitaires Genève (HUG), Geneve, Geneve; ^5^Ste‐Justine Hospital, Montreal, PQ; ^6^Research Center Of Centre Hospitalier De L'Université De Montréal, Montreal, PQ; ^7^Université Laval, PQ*

4:00 PM ‐ 6:00 PM

**Objective**: Measuring cervical length (CL) is a standard practice of stratifying the likelihood of preterm birth (PTB). Dynamic changes in the cervix can also be explored by placing the patient in the upright position, i.e. in the usual position of activity and measuring the shortening of the cervix and changes in the utero‐cervical angle (UCA). The aim of this study is to determine whether dynamic measurement of CL and UCA improves prediction of PTB compared additionally to the CL measurement performed in a gynecological position.

**Study Design**: This is a prospective cohort study of women who had vaginal ultrasound exam for CL measurement at 4 institutions in Canada and Switzerland. The ultrasound exam was performed endovaginally in the supine position, then in the standing position with measurements taken at 1 minute, 3 minutes and during standing Valsalva (SV). UCA were measured after the exam on the images according to a standardized measurement procedure and the differences in UCA (DUCA) between the supine and standing positions were calculated.

**Results**: 253 participants were included, with a mean age at ultrasound of 27.4 weeks of gestation (WG), a median CL of 26 mm, and a funneling present in 29% of cases in the gynecological position. Among those women, 68 (26.8%) experienced PTB. LC mean was 28.5mm (standard deviation (SD) 10) at rest and 27.3mm (11.5) in SV in the term group, compared with 23.5mm (10.2) and 21.4mm (10) in the PB group respectively. The UCA mean was 113° (25) at rest and 115° (28) in SV in the term group compared with 88.6° (25) and 99° (27) in the PTB group respectively. From the ROC curves, we chose a threshold for UCA of 104° and 5° for DUCA (figure 1). Among participants with LC<31mm, the use of UCA and DUCA significantly improved the prediction of PB but not the LC.

**Conclusion**: Using endovaginal screening to select a population at risk of PB (LC<31mm), the use of UCA measurement and its variation between the supine and upright positions (DUCA) improves prediction of PB.

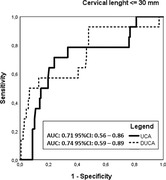

## 1180 | McDonald versus Shirodkar Cervical Cerclage for the Prevention of Preterm Birth in Patients with Obesity

Sarah H. Abelman; Frank I. Jackson; Nathan A. Keller; Julie Chen; Luis A. Bracero; Matthew J. Blitz

*Northwell, New Hyde Park, NY*

4:00 PM ‐ 6:00 PM

**Objective**: Differences in outcomes after Shirodkar versus McDonald cerclage techniques remain unclear. Some evidence suggests a potential benefit of Shirodkar cerclage in obese patients. This study aims to compare gestational age at delivery of patients with obesity who received either McDonald or Shirodkar cerclages.

**Study Design**: This was a retrospective cohort study of all patients with obesity (BMI ≥ 30 kg/m^2^) who received cerclage during pregnancy between January 2018 and December 2023 within a large health system in New York. The primary exposure was cerclage type. The primary outcome evaluated was gestational age at delivery. Outcomes were compared using a linear mixed model regression analysis, and were then adjusted for maternal age, BMI, nulliparity, history of prior preterm birth, cerclage indication, and gestational age at cerclage placement. A subgroup analysis was performed based on cerclage indication, additionally adjusting for very short cervix less than 1 cm in the ultrasound‐indicated cerclage subgroup.

**Results**: 517 pregnant patients with obesity who received cerclages were included in this study. 238 (46%) were ultrasound‐indicated, 118 (23%) were exam‐indicated, and 158 (31%) were history‐indicated cerclages. 385 (74%) received a McDonald cerclage and 132 (26%) received a Shirodkar cerclage. The mean gestational age at placement was 17.0 weeks (SD 3.5) for the McDonald group and was 17.3 (SD 3.4) for the Shirodkar group. Unadjusted and adjusted analyses showed no significant difference in pregnancy duration between the two groups, with the mean gestational age at delivery being 36.2 weeks (SD 4.4) for the McDonald group and 35.8 (SD 4.5) for the Shirodkar group (*P* = 0.48). Subgroup analyses by cerclage indication also showed no difference in gestational age at delivery based on Shirodkar versus McDonald technique (figure 1).

**Conclusion**: In this large cohort of patients with obesity undergoing cerclage placement during pregnancy, there was no difference in delivery timing based on cerclage technique. Both techniques appear equally effective in prolonging pregnancy.

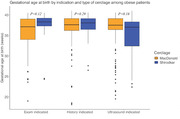

## 1181 | Screening and Diagnosis of Gestational Diabetes, and Perinatal Outcomes in Patients with Bariatric Surgery History

Silpa Annavarapu^1^; Charlette Williams^2^; Rodney A. McLaren, Jr., Jr.^3^; Sarah Boudova^4^

*^1^Thomas Jefferson University Sidney Kimmel Medical College, Philadelphia, PA; ^2^Sidney Kimmel Medical College, Thomas Jefferson University, Philadelphia, PA; ^3^Thomas Jefferson University Hospital, Philadelphia, PA; ^4^Thomas Jefferson University, Philadelphia, PA*

4:00 PM ‐ 6:00 PM

**Objective**: History of bariatric surgery is increasingly common in pregnancy. The glucose tolerance test (GTT) is the gold standard for diagnosing gestational diabetes (GDM), however individuals with a history of bariatric surgery may not tolerate the sugar load and there is concern that the test may be inaccurate in this population. There are no current ACOG guidelines regarding screening for GDM in patients with a history of bariatric surgery. We aimed to characterize how individuals with a history of bariatric surgery were screened and diagnosed with GDM and describe pregnancy outcomes by screening method.

**Study Design**: This was a retrospective cohort study of pregnancies with a history of bariatric surgery delivered at a major urban referral hospital from 1/17‐12/23. Demographics, bariatric surgery history, GDM screening method, pregnancy outcomes and neonatal outcomes were abstracted. The protocol was IRB approved. Data were analyzed with descriptive statistics.

**Results**: Of the 192 identified pregnancies with a history of bariatric surgery, 17 (8.9%) had pregestational diabetes and 11(5.7%) had no data on screening and were excluded from analysis. Among the remaining 164 pregnancies, 29 (17.7%) were diagnosed with GDM, and 59 (36.0%) did not complete screening. The most common screening method was exclusive GTT (47, 28.7%), followed by glucose fingersticks (34, 20.7%), followed by mixed screening methods (18, 11.0%) (Table 1). GTT was most commonly used for those with sleeve gastrectomy, while fingersticks were most commonly used for Roux‐en‐Y. Cesarean delivery occurred in 68, 41.5% pregnancies. Hypertensive disorder of pregnancy occurred in 28, 17.1% pregnancies. The composite adverse neonatal outcome occurred in 40 (24.4%) deliveries and was more common among those diagnosed with GDM (Table 2).

**Conclusion**: Among pregnancies with a history of bariatric surgery, GDM was common, however, there was a low rate of completed GDM screening. GTT was the most common screening method. Further research is needed to determine the reason for low rates of GDM screening.

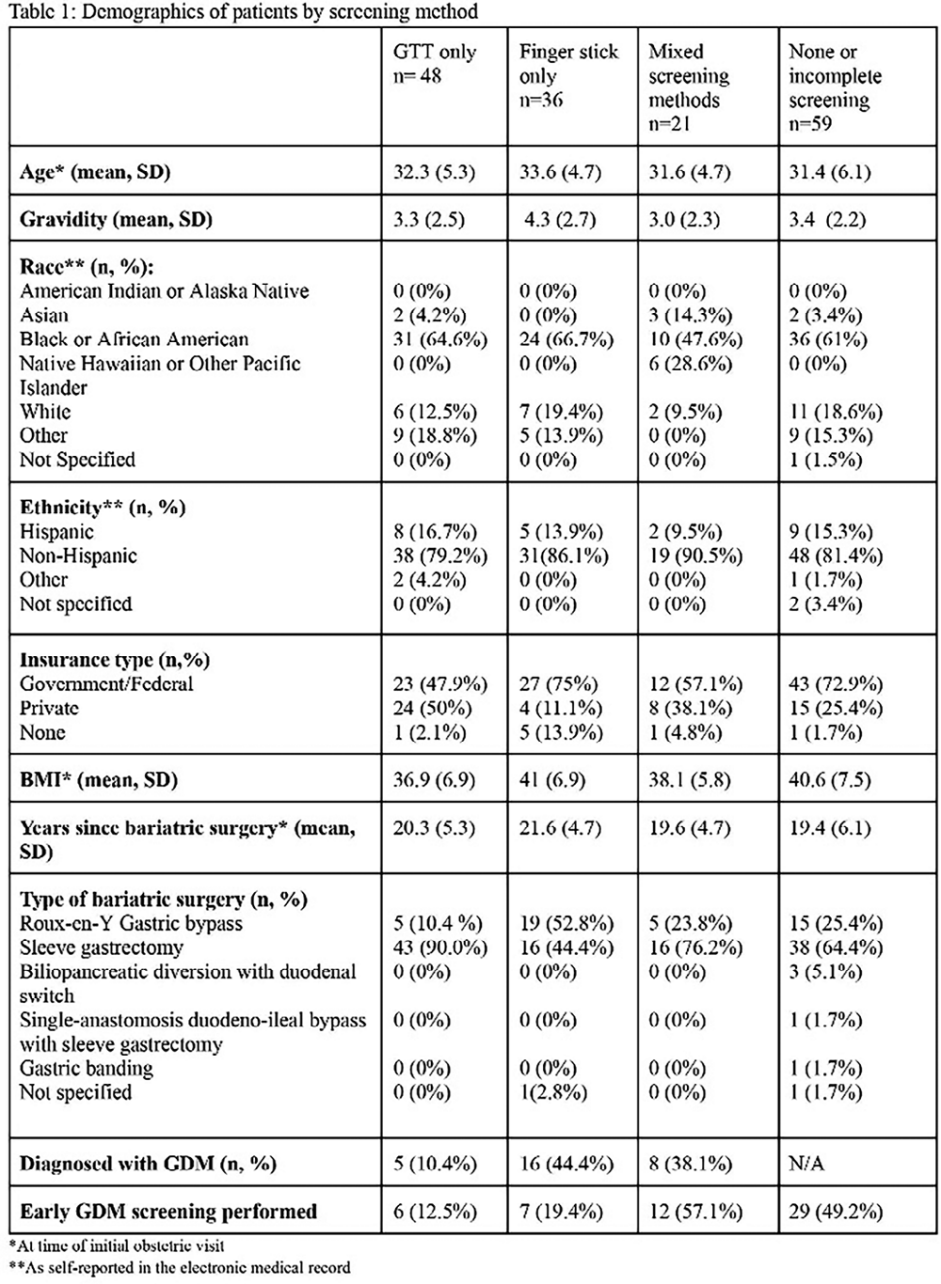

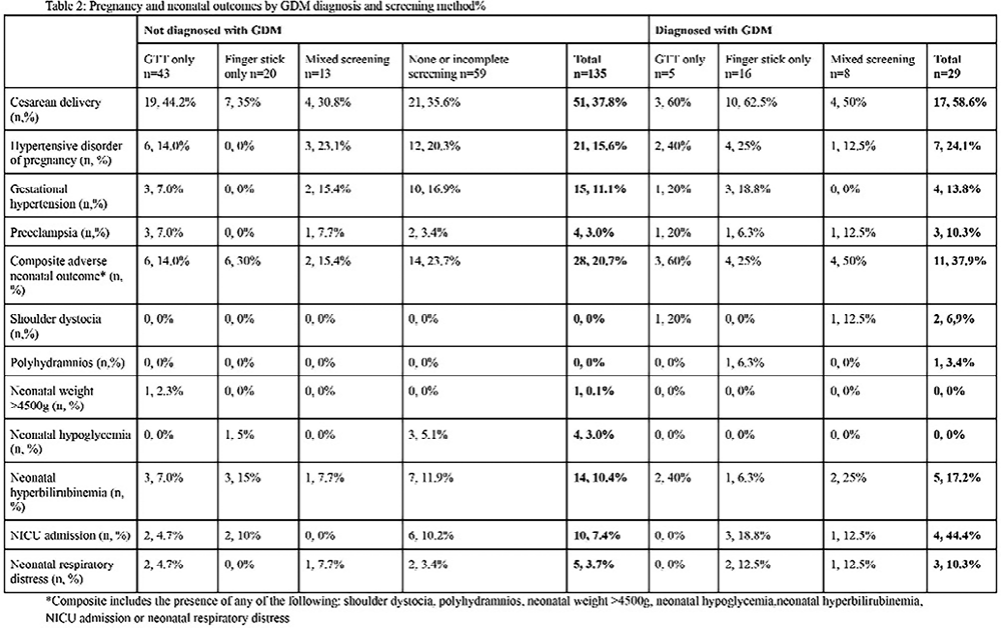

## 1182 | The Effect of Gestational Weight Gain on Successful Operative Vaginal Delivery

Shannon R.O Moran; James A. Shelton; Dawei David Wang

*University at Buffalo, Buffalo, NY*

4:00 PM ‐ 6:00 PM

**Objective**: Successful operative vaginal delivery (OVD) has been associated with reduced maternal and neonatal morbidity. However, appropriate patient selection is necessary to reduce the risks of failed OVD. Previous studies have shown mixed results regarding the effect of body mass index (BMI) on rates of OVD success. However, no studies have examined the effect of gestational weight gain (GWG) on OVD success. Therefore, we sought to investigate the effect of excessive GWG on OVD success.

**Study Design**: This was an IRB‐approved retrospective cohort study of birth certificate data from our institution from 2014 to 2023. We included singleton, term, vertex livebirths with no major fetal anomalies who attempted OVD. Those with excessive GWG based on the Institute of Medicine guidelines were compared to those with adequate GWG. The primary outcome was successful OVD. Secondary outcomes included composite maternal and neonatal morbidity among those who achieved successful OVD. Logistical regression models estimated the odds ratios (OR) of successful OVD by GWG adjusted for maternal age and race, infant birthweight, gestational diabetes, gestational hypertension, previous cesarean section, prolonged labor, prior vaginal delivery, and BMI.

**Results**: 2,433 patients were included. 1,055 had adequate GWG (43%). 1,378 had excessive GWG (57%). 2,271 had a successful OVD (93%) and 162 had a failed OVD (7%). OVD was not affected by GWG (aOR 0.99, CI 0.64‐1.55). Subgroup analyses based on pre‐pregnancy BMI revealed no effect of weight gain on the success rate of OVD across all BMI groups. Finally, there was no difference in both maternal and neonatal outcomes in those with excessive GWG who had a successful OVD (14%, p‐value 0.6 versus 14% for those with adequate GWG and 14%, p‐value 0.095 versus 11% for those with adequate GWG, respectively).

**Conclusion**: Excessive GWG is not associated with a reduction in the rate of successful OVD or with adverse maternal or neonatal outcomes in those who do achieve successful OVD. Excessive GWG should not be considered a relative contraindication to OVD.

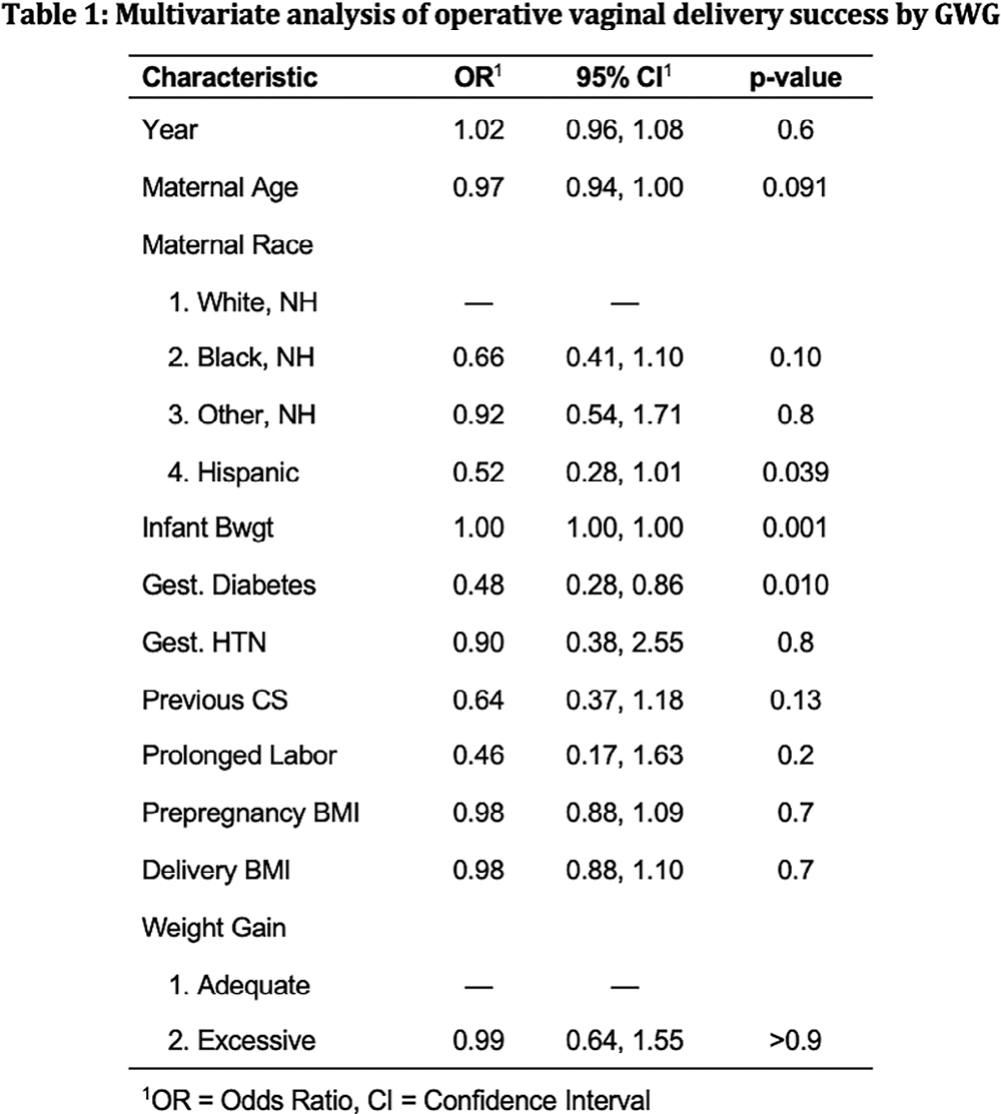

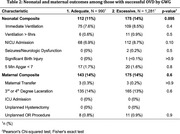

## 1183 | Monogeneic Disorder (MD) Screening using Cell‐Free DNA (cfDNA) In Routine Practice

Xiteng Yan^1^; Samantha R. Ratner^2^; Andrei Rebarber^3^; Tamar H. Goldwaser^4^; Shari Gelber^5^

*^1^Mount Sinai West, New York, NY; ^2^Perelman School of Medicine, Philadelphia, PA; ^3^Icahn School of Medicine at Mount Sinai Hospital, New York, NY; ^4^Maternal Fetal Medicine Associates, New York, NY; ^5^Icahn School of Medicine at Mount Sinai, New York, NY*

4:00 PM ‐ 6:00 PM

**Objective**: More pregnancies are affected by MDs than by trisomy 21. MDs are often not identified on ultrasound, standard invasive testing, or microarray. Clinical studies of cfDNA detection of MD report >99% sensitivity and specificity, with a 1:600 incidence of positive results in a high‐risk population. We sought to determine (1) the feasibility of offering routine cfDNA for MD, (2) false positive rate of MD diagnosed at the time of cfDNA for aneuploidy, (3) accuracy of cfDNA for MD when routinely offered to low‐risk patients.

**Study Design**: From 10/11/2021‐7/15/2024 all patients in a single obstetrical practice were offered MD screening (25 conditions in 47 genes) at the time of cfDNA screening for aneuploidy. Patients with egg donors, multiple gestation, and vanishing twins were excluded, as were those who had testing for ultrasound findings or other risk factors. Genetic counseling, diagnostic CVS/amniocentesis, and parental testing were recommended for patients with positive results. Repeat testing was recommended for patients who did not receive a result.

**Results**: 1831 pregnancies in 1746 women were included. Results were positive in 5 pregnancies (0.28%) (**Table**), negative in 1730 (94%), and 96 (5.2%) received no initial result. 75/96 patients repeated testing: 63 were negative and 12 did not result (0.66% final no‐call rate). Of the positive tests, 4 were true positive on confirmatory testing with CVS or amniocentesis and one was negative for both fetus and parents. MD testing was positive in 1:366 patients; incidence of the disorders was 1:458. False positive results occurred in < 1% of tests.

**Conclusion**: MDs affected >1:500 pregnancies. Screening for MDs is not recommended by professional societies or offered as part of routine care. Screening for MD in low‐risk pregnancies is feasible. MD screening in the general population should be considered, as it is unlikely to significantly increase unnecessary invasive testing and may prenatally diagnose conditions and afford women reproductive choice in the current pregnancy and the future.

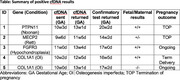

## 1184 | Tighter Blood Pressure Control Targets to Reduce Adverse Pregnancy Outcomes for Patients with Chronic Hypertension

Sharon W. Shu; Meena Mishra; Frank B. Williams

*Ochsner Clinic Foundation, New Orleans, LA*

4:00 PM ‐ 6:00 PM

**Objective**: The Chronic Hypertension and Pregnancy (CHAP) Trial demonstrated improved outcomes for pregnant women with mild chronic hypertension (HTN) assigned strict blood pressure (BP) target of ≤ 140/90 compared to controls with target of ≤ 160/110. CHAP‐based treatment guidelines were implemented at our health system in 2022. We hypothesize that implementation of CHAP guidelines in a large, socio‐demographically diverse population reduced HTN‐related morbidity.

**Study Design**: This is a retrospective cohort study of singleton pregnancies with a known or new diagnosis of mild HTN receiving prenatal and delivery care at a large regional health system in 2021 and 2023. In April 2022, BP targets of ≤ 140/90 were implemented. We compared 2021 with 2023 deliveries, with 2022 a washout period. Multiple gestations, major fetal anomalies, > 1 baseline antihypertensive, phospholipid syndrome or HTN‐related cardiac or kidney disease were excluded. Primary outcome per CHAP was a composite of medically indicated preterm birth before 35 weeks, superimposed preeclampsia with severe features, placental abruption & perinatal death. Secondary outcomes included small‐for‐gestational‐age and preterm birth before 37. Outcome adjusted odds ratios were generated via regression analyses controlling for maternal age, body mass index, public insurance, history of preeclampsia, and pre‐existing diabetes.

**Results**: Of 1626 pregnant women with HTN, 134 were excluded for twins, congenital anomalies, >1 baseline antihypertensive or HTN‐related comorbidities. Of the 1492 remaining, 710 were pre‐, while 782 were post‐implementation. The post‐intervention group had higher BMI and higher public payor insurance status (Table 1). The primary composite outcome occurred in 30.9% of pre‐implementation pregnancies compared to 29.2% of post‐implementation pregnancies (aOR 0.84, 95% CI 0.66‐1.09). Medically indicated delivery before 35 weeks was lower in the post‐intervention group (10.4% vs 12.1%, aOR 0.7, 95% CI 0.47‐0.99, Table 2).

**Conclusion**: Tighter BP control targets did not reduce the CHAP‐defined outcome.

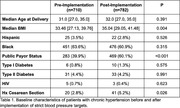

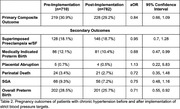

## 1185 | Intertwin EFW Discordance and Perinatal Loss in Monochorionic Diamniotic Twins with Selective Fetal Growth Restriction

Shelly Soni^1^; Juliana S. Gebb^2^; Christina Paidas Teefey^1^; Beverly G. Coleman^3^; Julie S. Moldenhauer^1^; Nahla Khalek^1^

*^1^Richard D. Wood Jr. Center for Fetal Diagnosis and Treatment at CHOP, Philadelphia, PA; ^2^Richard D. Wood, Jr Center for Fetal Diagnosis and Treatment, Children's Hospital of Philadelphia, Philadelphia, PA; ^3^Richard D. Wood, Jr Center for Fetal Diagnosis and Treatment, The Children's Hospital of Philadelphia, Philadelphia, PA*

4:00 PM ‐ 6:00 PM

**Objective**: To identify the association between the degree of intertwin estimated fetal weight (EFW) discordance and perinatal loss in monochorionic diamniotic (MCDA) twin pregnancies complicated by selective fetal growth restriction (sFGR).

**Study Design**: Single center retrospective review of MCDA twins diagnosed with sFGR that opted for expectant management between 2010‐2021. The relationship between intertwin EFW discordance and perinatal loss was evaluated using receiver–operating characteristics (ROC) and survival analysis to identify the optimal cut‐off for EFW discordance. The 2 groups of different intertwin discordance were compared to evaluate and identify other risk factors.

**Results**: A total of 212 MCDA twin pregnancies with sFGR underwent expectant management in the study period. 18 pregnancies (8.5%) were dual demise and 11 (5.2%) had demise of one fetus. Of those born alive, dual neonatal demise was seen in 5 (2.4%) pregnancies whereas neonatal demise of one twin was seen in 11 pregnancies (5.2%). Further, of the 11 pregnancies with one fetal demise, 5 (2.4%) had neonatal demise. Total perinatal loss rate was 73 fetuses/neonates (17.2%) of 424 (212 twins). The area under curve (AUC) ROC curve for intertwin EFW discordance and perinatal loss was 0.68 [95% CI 0.59‐0.78] with a p‐value of 0.0001 (Graph 1). A discordance of ≥ 33% was 56.82% sensitive and 68.45% specific in predicting perinatal loss. The gestational age at delivery and two survivors to discharge were significantly lower in pregnancies with a discordance of ≥ 33% (Table). Kaplan–Meier analysis showed that pregnancies with a discordance of ≥ 33% had a significantly lower survival trend with a p‐value of 0.0001 and hazards ratio for risk of perinatal loss of 3.83 [95% CI 1.85‐7.95] (Graph 2).

**Conclusion**: Intertwin EFW discordance of ≥ 33% can be used as the optimal cut‐off for prediction of perinatal loss in cases of MCDA twin with sFGR.

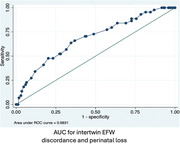

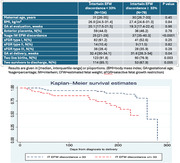

## 1186 | A Scoring System for Estimating Risk of Perinatal Loss in Monochorionic Diamniotic Twins with sFGR

Shelly Soni^1^; Juliana S. Gebb^2^; Christina Paidas Teefey^1^; Edward R. Oliver^2^; Julie S. Moldenhauer^1^; Nahla Khalek^1^

*^1^Richard D. Wood Jr. Center for Fetal Diagnosis and Treatment at CHOP, Philadelphia, PA; ^2^Richard D. Wood, Jr Center for Fetal Diagnosis and Treatment, Children's Hospital of Philadelphia, Philadelphia, PA*

4:00 PM ‐ 6:00 PM

**Objective**: To evaluate a novel scoring system based on markers of placental insufficiency and fetal physiology that can assist in predicting perinatal loss in monochorionic diamniotic (MCDA) twins complicated by selective fetal growth restriction (sFGR).

**Study Design**: Single center retrospective review of MCDA twins diagnosed with sFGR that opted for expectant management between 2010‐2021. Various demographic and outcome variables were compared in pregnancies with and without perinatal loss. Significant variables were analyzed in a multiple logistic regression model with backward elimination method to identify factors predictive of perinatal loss. The identified significant factors were re‐run in multiple logistic regression to assess individual effect on outcomes. A score was then assigned based on the strength of the effect. The relationship between the assigned score and perinatal loss was evaluated using receiver–operating characteristics (ROC) and survival analysis to identify optimal cut‐off.

**Results**: A total of 212 MCDA twin pregnancies with sFGR underwent expectant management in the study period. Perinatal loss occurred in 44 pregnancies (20.8%). Of the significant variables from univariate analysis (Table 1); intertwin EFW discordance, and abnormal findings in growth restricted twin including low amniotic fluid, abnormal UA Dopplers and abnormal MCA Dopplers were identified as significant in the stepwise multiple logistic regression. These variables were re‐entered into a logistic regression model and a score was assigned according to the relative contribution of each variable in this model (Table 2). The area under ROC curve(AUC) for the score and perinatal loss was 0.78 [95% CI 0.7‐0.85] with a p‐value of 0.0001 (Graph 1). A score of ≥ 3 was 70.5% sensitive and 76.2% specific in predicting perinatal loss. Kaplan–Meier analysis showed that pregnancies with a high score (≥ 3) had a significantly lower survival trend with a p‐value of < 0.0001 (Graph 2).

**Conclusion**: This novel scoring system in MCDA twins with sFGR can be used to assess risk of perinatal loss that can be used to assist in counseling.

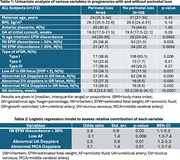

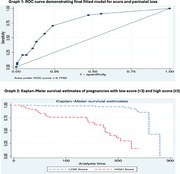

## 1187 | Association Between Being Born Small for Gestational Age and the Risk for Childhood Celiac Disease

Shir Luvaton^1^; Tamar Wainstock^2^; Eyal Sheiner^2^; Gali Pariente^2^

*^1^Department of Obstetrics and Gynecology, Soroka University Medical Center, Ben‐Gurion University of the Negev, Beer‐Sheva, Israel., Be'er Sheva, HaDarom; ^2^Department of Obstetrics and Gynecology, Soroka University Medical Center, Ben‐Gurion University of the Negev, Beer‐Sheva, Israel., Beer Sheva, HaDarom*

4:00 PM ‐ 6:00 PM

**Objective**: Celiac disease is a common immune disorder, frequently associated with other autoimmune conditions. Autoimmunity is recognized as a potential factor contributing to placental insufficiency, by affecting placental development and structure, which may lead to fetal growth restriction. Based on the possible correlation between autoimmunity, celiac disease and placental insufficiency, we sought to investigate whether small for gestational age (SGA) newborns are at an increased risk of developing celiac disease during childhood.

**Study Design**: A population‐based cohort study was conducted to evaluate the risk of celiac disease during childhood (up to the age of 18 years) among individuals who were born SGA between the years 1991‐2021, in a single tertiary medical center. Data for the diagnosis of celiac disease was extracted from community‐based clinics and hospitalization records. Kaplan‐Meier survival curve was used to compare the cumulative incidence of celiac disease between the study groups (SGA vs. no SGA). A cox proportional hazards model was used to control for confounders.

**Results**: During the study period, 356,356 newborns were included in the analysis; of which 16,210 (4.5%) were born SGA. Being born SGA was associated with higher risk for celiac disease as compared to children who were not born SGA (0.9% vs 0.5%, P< 0.001, Table). However, the Kaplan‐Meier survival curve did not demonstrate higher cumulative incidence rates of celiac disease in infants born SGA (Log Rank p = 0.287, Figure). Likewise, using a Cox proportional hazards model, adjusted for maternal celiac disease, maternal age and gestational age at birth, the finding was no longer statistically significant (adjusted HR 1.10, 95% CI 0.92‐1.30, p = 0.274, Table).

**Conclusion**: According to our results, being born SGA is not an independent risk factor for long‐term childhood celiac disease.

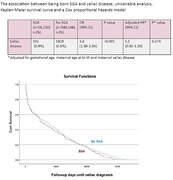

## 1188 | Risk of Severe Maternal Morbidity in Pregnant People with Malignant Neoplasm Using a Population Database

Shriddha Nayak^1^; Kristin C. Darwin^2^; Arthur Jason Vaught^2^; Marika Toscano^1^

*^1^Johns Hopkins University, School of Medicine, Baltimore, MD; ^2^Johns Hopkins University, Baltimore, MD*

4:00 PM ‐ 6:00 PM

**Objective**: The objective of this study was to determine the difference in odds of severe maternal morbidity (SMM) in people with and without malignant neoplasm in pregnancy using a real‐world sample derived from TriNetX, a research network containing de‐identified electronic health record data from 93 health care organizations and > 131 million patients.

**Study Design**: This population‐based, retrospective cohort study queried TriNetX from inception to 8/2024 for subjects 12‐55 years with a diagnosis of pregnancy. Two cohorts were derived: subjects with and without active diagnosis of any malignant neoplasm within one year prior to, or up to one month after, first instance of pregnancy. Cohorts were propensity score matched 1:1 based on demographic characteristics (age, race, and ethnicity) and comorbidities (diabetes, hypertension, obesity, cardiovascular disease, asthma, and tobacco use) by TriNetX analysis tools. SMM was defined as CDC's 21 indicators and corresponding ICD‐10 codes. The primary outcome was difference in odds ratio (OR) between cohorts of composite SMM occurring up to one year after first instance of pregnancy. Secondary outcomes were differences in odds of individual indicators of SMM. All statistical analyses were conducted 8/2024 using the TriNetX platform.

**Results**: Of 5567972 pregnant subjects, 5373233 had no malignancy history. After propensity score matching, subjects with and without (n = 51484 for both) malignant neoplasm in pregnancy were analyzed (Table). Subjects with malignant neoplasm in pregnancy had significantly higher odds of composite SMM (OR 3.43 (95% CI 3.24‐3.64), p< 0.001) compared to those without malignant neoplasm in pregnancy. Cases of malignant neoplasm in pregnancy had significantly higher odds of all individual indicators of SMM compared to controls, except for eclampsia (Table).

**Conclusion**: Subjects with malignant neoplasm in pregnancy had over threefold increased odds of composite SMM, as well as significantly increased odds of 16 of the 21 individual indicators of SMM, compared to those without malignancy in pregnancy.

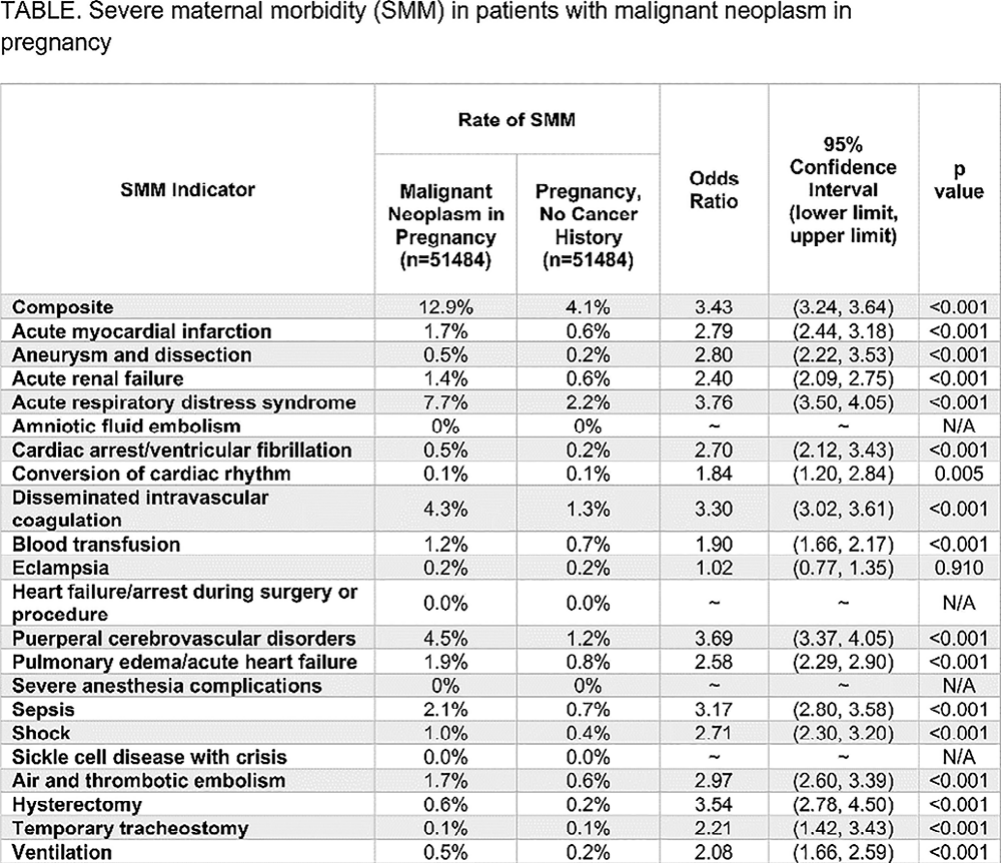

## 1189 | Prevalence of Severe Maternal Morbidity Associated with Different Cancer Types in Pregnancy

Shriddha Nayak^1^; Kristin C. Darwin^2^; Arthur Jason Vaught^2^; Marika Toscano^1^

*^1^Johns Hopkins University, School of Medicine, Baltimore, MD; ^2^Johns Hopkins University, Baltimore, MD*

4:00 PM ‐ 6:00 PM

**Objective**: To describe prevalence of composite severe maternal morbidity (SMM) in the top ten most common types of female cancers in the United States using a real‐world sample derived from TriNetX, a research network containing de‐identified electronic health record data from 93 health care organizations and > 131 million patients.

**Study Design**: This descriptive cohort study queried TriNetX from inception to 8/2024 for subjects 12‐55 years with a diagnosis of malignant neoplasm within one year prior to, or up to one month after, first instance of pregnancy. A cohort of all malignant neoplasm types, further subdivided by individual types, were analyzed. Included malignant neoplasm types were identified from CDC's most recently reported female cancer types associated with new cancer diagnoses, or cancer deaths, in the United States. SMM was defined as CDC's 21 indicators and corresponding ICD‐10 codes. The primary outcome was prevalence of composite SMM occurring up to one year after first instance of pregnancy. Secondary outcomes included prevalence of individual indicators of SMM. All descriptive analyses were conducted 8/2024 using the TriNetX platform.

**Results**: There were 51484 individuals with malignant neoplasm in pregnancy. Mean age was 32.4 years and comorbid conditions included hypertension (12.3%), diabetes mellitus (11.1%), obesity (13.6%), asthma (11.3%) and tobacco use (13.0%). Breast cancer and skin cancer/melanoma were the most common malignancies co‐occurring with pregnancy (Table). The prevalence of composite SMM for all cancer type in pregnancy was 12.9% with morbidity most often acute respiratory distress syndrome (7.7%) (Table). Composite SMM prevalence was highest for liver/bile duct cancer (17.4%). Thyroid cancer in pregnancy had the lowest composite SMM rate of 6.5%.

**Conclusion**: Close to one in eight patients with a malignant neoplasm in pregnancy will experience SMM, with prevalence of composite SMM differing by malignancy type. Further research is needed to evaluate the short‐ and long‐term outcomes of patients with cancers during pregnancy.

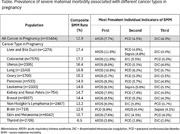

## 1190 | Risk of Severe Maternal Morbidity Among Pregnant People with Breast Cancer Using a Population Database

Shriddha Nayak^1^; Kristin C. Darwin^2^; Arthur Jason Vaught^2^; Marika Toscano^1^

*^1^Johns Hopkins University, School of Medicine, Baltimore, MD; ^2^Johns Hopkins University, Baltimore, MD*

4:00 PM ‐ 6:00 PM

**Objective**: A new breast cancer diagnosis is more common than all other cancer types in females 15‐39 years in the United States and is the second leading cause of female cancer deaths. Our objective was to determine the odds of severe maternal morbidity (SMM) in breast cancer in pregnancy using TriNetX, a research network containing real‐world data from 93 health care organizations and > 131 million patients.

**Study Design**: This retrospective cohort study queried TriNetX from inception to 8/2024 for subjects 12‐55 years with a pregnancy diagnosis. Two cohorts were derived: (1) individuals with an active diagnosis of breast cancer 1 year prior to, or up to 1 month after, first instance of pregnancy and (2) those without a history of any cancer type in pregnancy. Cohorts were propensity score matched 1:1 by demographics and comorbidities by TriNetX built‐in analysis tools. SMM was defined as CDC's 21 indicators and corresponding ICD‐10 codes. The primary outcome was odds ratio (OR) between cohorts of composite SMM occurring up to 1 year after first instance of pregnancy. Secondary outcomes were odds of individual indicators of SMM. All statistical analyses were conducted in the TriNetX platform.

**Results**: Propensity score matching yielded 9937 cases of breast cancer in pregnancy and 9937 cases of pregnancy without cancer history (Table). Subjects with breast cancer in pregnancy had significantly higher odds of composite SMM (OR 3.61 (95% confidence interval (CI) 3.17‐4.12), p< 0.001) compared to those without malignancy in pregnancy. The odds of acute respiratory distress syndrome (ARDS) were highest (OR 3.89 (95% CI 3.31‐4.56), p< 0.001), followed by cerebrovascular disorders (OR 3.30 (95% CI 2.73‐3.98), p< 0.001) and disseminated intravascular coagulation (DIC) (OR 3.01 (95% CI 2.47‐3.67), p< 0.001).

**Conclusion**: Individuals with breast cancer in pregnancy have more than three‐fold increased odds of composite SMM compared to those without cancer history, with considerably higher risk of ARDS, cerebrovascular disorders, and DIC.

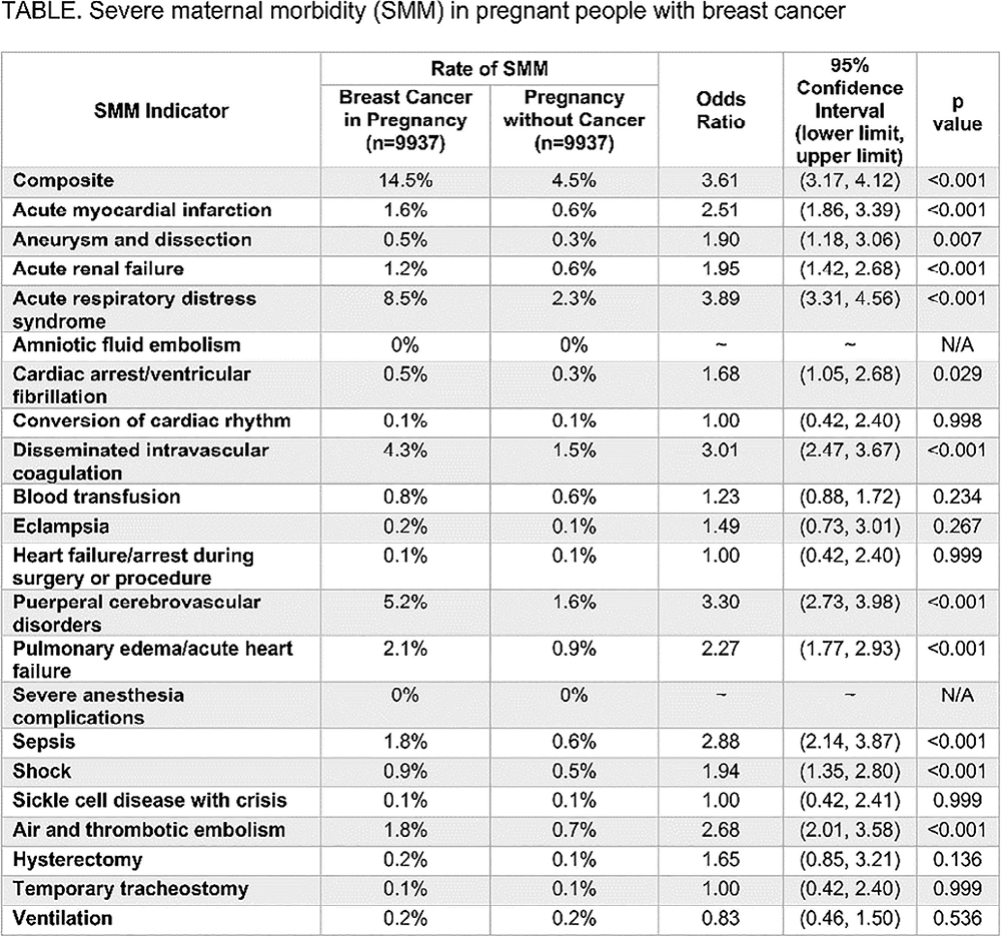

## 1191 | Timing of Alpha‐Feto Protein (AFP) Screening: Unveiling the Best Predictor for Adverse Pregnancy Outcomes

Siddharth Hariharan^1^; Hameeda A. Naimi^2^; Eric K. BRONI^3^; Shifa Turan^4^; Ozhan M. Turan^5^

*^1^University of Rochester, Rochester, NY; ^2^Virginia Women's Center, Inc., Richmond, VA; ^3^University of Pennsylvania Perelman School of Medicine, Philadelphia, PA; ^4^University of Maryland Medical System, Baltimore, MD; ^5^University of Maryland Medical Center, Baltimore, MD*

4:00 PM ‐ 6:00 PM

**Objective**: AFP at second trimester is used for prediction of neural tube defects and adverse pregnancy outcomes (APO). We hypothesized that first trimester is a better time for screening APOs than second trimester.

**Study Design**: This secondary analysis of a prospective cohort study included 2,245 patients. Pregnancies with known structural or chromosomal abnormalities, multifetal gestation, or incomplete data were excluded. Primary outcomes were preterm premature rupture of membranes (PPROM), preterm delivery (PD), and composite outcomes (CO: any occurrence of PPROM, PD, placental abruption, or preeclampsia). Alpha‐fetoprotein (AFP) levels were measured in the first and second trimesters. A cutoff of 1.33 multiples of the median (MoM) for first trimester AFP, determined via receiver operating curve analysis, was used to predict adverse pregnancy outcomes (APOs) in the 1,713 first‐trimester cases. The routine 2.5 MoM threshold was applied for second trimester AFP. A statistical model, adjusted for common risk factors (maternal age, sepsis, chronic hypertension, and preeclampsia), was used to assess associations with APOs, and the predictive values of first and second trimester AFP levels were compared in the statistical model.

**Results**: In the final analysis, 1,224 patients were included (Figure 1). Those with elevated first trimester AFP were more likely to be nulliparous (53.4% vs. 36.0%, p< 0.001) and have elevated second trimester AFP (5.54% vs. 0.65%, p< 0.001). Adjusted odds ratios for elevated first trimester AFP were 2.28 (p = 0.004) for PPROM, 3.42 (p< 0.001) for PD, and 1.66 (p = 0.009) for CO. A high negative predictive value (92.4‐96.6%) was observed (Table 1). More patients with APOs were detected using first trimester AFP compared to second trimester AFP (p< 0.001 for all, Table 2).

**Conclusion**: First trimester AFP is a reliable biomarker with a high negative predictive value for excluding pregnancies at risk for APOs. In our study, it effectively replaced second trimester AFP, potentially eliminating the need for second trimester blood draws in care centers with advanced ultrasound capabilities.

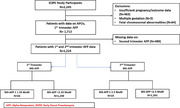

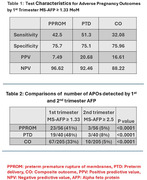

## 1192 | The Diagnostic Accuracy of the sFlt‐1/PlGF Ratio vs. PlGF alone for Preeclampsia

Siobhan Wilson^1^; Elaine Carson^2^; Lei Fu^3^; Nir Melamed^4^

*^1^University of Toronto, Toronto, ON; ^2^Department of Maternal Fetal Medicine, Leeds Hospital Trust, Leeds, England; ^3^Sunnybrook Health Sciences Centre, Toronto, ON; ^4^Division of Maternal Fetal Medicine, Department of Obstetrics and Gynecology, Sunnybrook Health Sciences Centre, Toronto, ON*

4:00 PM ‐ 6:00 PM

**Objective**: Preeclampsia is a leading cause of maternal and fetal morbidity and mortality. Abnormal levels of the angiogenic proteins soluble fms‐like tyrosine kinase (sFLT‐1) and placental growth factor (PlGF) have been shown to be predictive of preeclampsia. However, it remains unclear whether the combination of these proteins in the form of the sFlt1/PlGF ratio performs better than PlGF alone. The objective of the current study was to compare the diagnostic accuracy of the sFlt1/PlGF ratio for preeclampsia with that achieved with PlGF alone under real‐life settings.

**Study Design**: A retrospective cohort study of patients with a singleton pregnancy evaluated in a single center for suspected preeclampsia using the Roche Elecsys sFlt1/PlGF Ratio Assay between 2020‐2023. The sFlt1/PlGF ratio was interpreted as low‐risk (≤ 38), moderate‐risk (38‐85), or high‐risk (> 85), while PlGF was defined as low‐risk (≥ 100 pg/mL) or high‐risk (< 100 pg/mL).

**Results**: Of the 509 patients who met the study criteria, 206 (40.5%) had a final diagnosis of preeclampsia. When compared to  PlGF<  100 ng/pL, a sFLT‐1/PlGF ratio > 85 was associated with a greater risk of delivery within < 14 (RR = 2.0 [1.8‐2.4] vs 1.4 [1.3‐1.6]), < 7 (RR = 2.7 [2.1‐3.5] vs 1.5 [1.3‐1.8]), and < 3 days (RR = 2.7 [2.0‐3.8] vs 1.4 [1.2‐1.8]). In addition, the area under the ROC curve was higher for the sFLT‐1/PlGF ratio to PlGF for delivery within < 7 days (0.86 [0.83‐0.89] vs. 0.64 [0.58‐0.71]) and preeclampsia diagnosis within < 7 days (0.91 [0.91‐0.95] vs. 0.72 [0.65‐0.78]). Finally, in a time‐to‐event analysis, the cumulative risk of delivery was significantly higher among patients with sFLT‐1/PlGF ratio > 85 compared to those with PlGF < 100 ng/pL (p< .001) (Figure 1).

**Conclusion**: The diagnostic accuracy and prognostic value of the sFLT‐1/PlGF ratio appears to be greater than that achieved with PlGF alone.

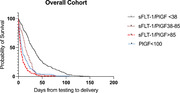

## 1193 | Small‐For‐Gestational‐Age Predictors in Gestational Diabetes Mellitus: The Impact of Metformin use in GDMA2 Pregnancies

Sivan Farladansky Gershnabel; Dina Lidsky‐Sachs; Nur Abd El Qadir; Ronny Ram Biton; Tal biron shental; Michal Kovo; Dorit Ravid

*Meir Medical Center, Kfar Saba, HaMerkaz*

4:00 PM ‐ 6:00 PM

**Objective**: Gestational diabetes mellitus (GDM) affects 3% ‐ 25% of pregnancies worldwide, posing risks to maternal, fetal, and neonatal health. GDM is often associated with macrosomia and large‐for‐gestational‐age (LGA) infants. However, the relationship between GDM and small‐for‐gestational‐age (SGA) infants is less understood. This study aims to identify predictors of SGA in women with GDM.

**Study Design**: This retrospective study included GDM patients (GDM A1 and A2) admitted to the high‐risk unit between 2014 and 2023. The study population was divided into those who delivered an appropriate for gestational age (AGA) neonate (AGA group) and those who delivered an SGA neonate (SGA group), defined as birthweight < 10th percentile. Women with pregestational diabetes mellitus were excluded. Obstetric and neonatal outcomes were compared between the groups. A subgroup analysis focused on GDMA2 patients, comparing maternal and neonatal outcomes and treatment regimens (insulin and metformin use).

**Results**: The study included 894 GDM patients. Compared to the AGA group (n = 712), the SGA group (n = 182) had lower maternal BMI (p = 0.02). Maternal age (p = 0.04), rates of GDMA2 (30.2% vs. 23.45%, p = 0.07), and hypertensive disorders (7.2% vs. 5.1%, p = 0.21) did not differ significantly between the groups. The SGA group had lower neonatal birthweight (2375 ± 432 g vs. 3021 ± 165 g, p = 0.005) and a higher rate of CD due to NRFHR (51% vs. 27.2%, p< 0.01). Among GDMA2 patients (n = 222), more women in the SGA group (n = 55) were treated with metformin compared to the AGA group (n = 167) (72.7% vs. 28.7%, p< 0.001). Multivariate regression analysis revealed that metformin treatment was independently associated with the risk of SGA among GDMA2 patients (OR 1.7, CI 1.18‐1.35, p< 0.01).

**Conclusion**: Metformin use in GDMA2 pregnancies may be linked to SGA neonates. The impact of metformin on fetal growth highlights the need for careful monitoring and individualized treatment strategies in managing GDMA2.

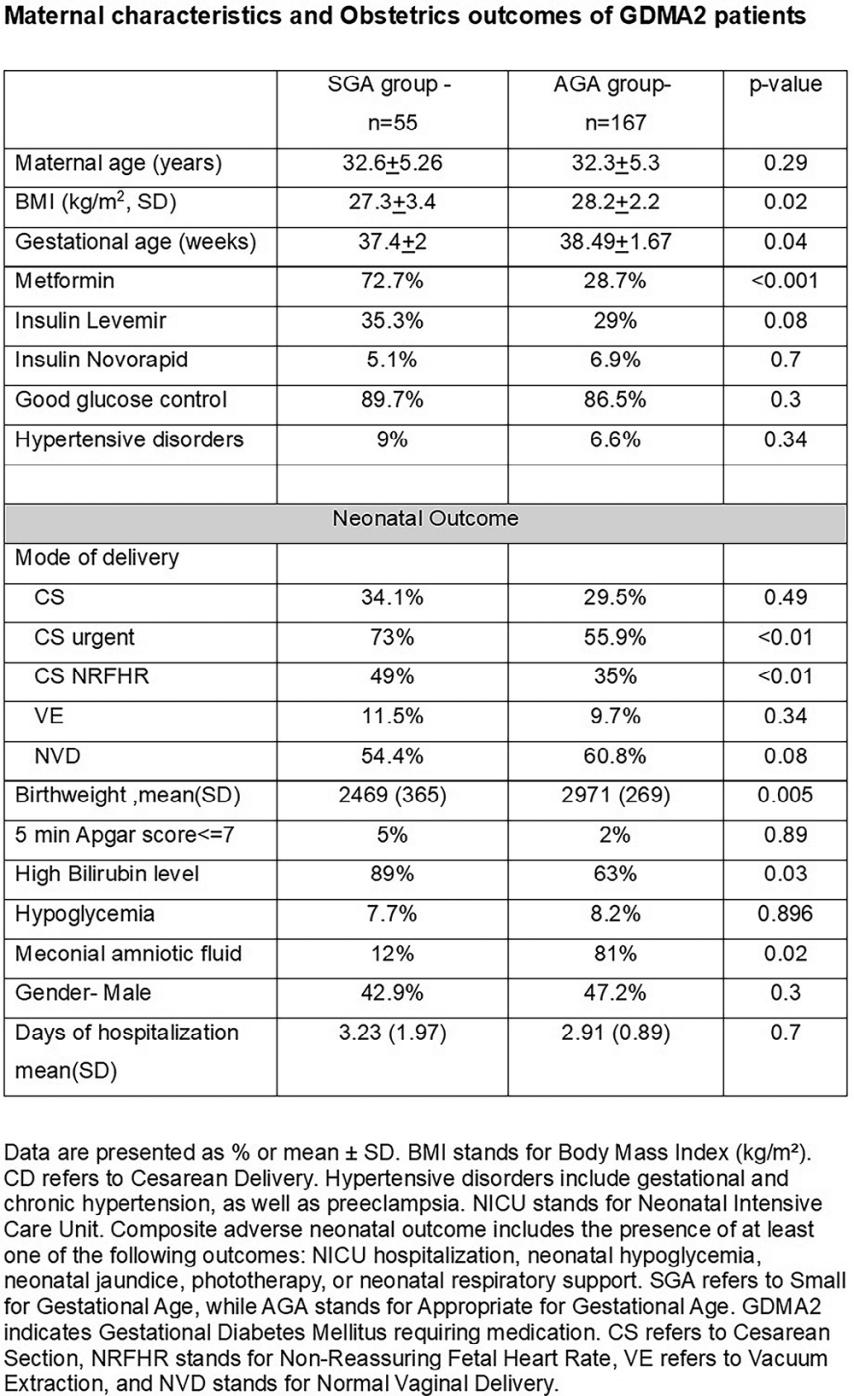

## 1194 | MRI‐Based Prediction of the Need for Surgical Intervention in Fetuses with Micrognathia

Stephanie Marie Cruz; Oluseyi Ogunleye; Jason Xia; Mitchell Rees; Mai‐Lan Ho; Jonathan Grischkan; Meredith Lind; Adolfo Etchegaray; Oluyinka Olutoye

*Nationwide Children's Hospital, Columbus, OH*

4:00 PM ‐ 6:00 PM

**Objective**: Diagnosis of fetal micrognathia, characterized by a small fetal mandible causing upper airway obstruction and immediate postnatal respiratory distress, has historically relied on subjective ultrasound assessments. More recent objective measurements including the inferior facial angle (IFA), jaw index (JI), mandibular width/maxillary ratio (MD/MX ratio), and oropharyngeal area (OP) aim to enhance diagnostic accuracy. This study aimed to evaluate the ability of fetal MRI measurements to predict the need for immediate postnatal surgical intervention.

**Study Design**: We conducted a retrospective query of our Radiology database for ‘micrognathia’ cases from 2007 to 2022. Radiologists, blinded to clinical details, independently assessed JI, IFA, MD/MX ratio, and OP area. Baseline demographics, comorbidities and clinical outcome metrics including the need for any Otolaryngologic surgical intervention were collected. Logistic regression models were fitted to examine the association between each parameter and the need for surgery, using median radiologist‐assessed values.

**Results**: Forty fetal MRI scans of 37 patients were analyzed at a median gestational age of 27 weeks. Median values of JI, IFA, MD/MX ratio, and OP area were lower in cases requiring surgical intervention compared to those not needing surgery, but these differences were not statistically significant (p > 0.05). Receiver operating characteristic (ROC) analysis revealed optimal cutoffs for predicting surgery. The more reliable predictors demonstrated modest performance: JI (sensitivity 65.4%, specificity 63%) and IFA (sensitivity 52.5%, specificity 52.8).

**Conclusion**: While not statistically significant, both JI and IFA showed modest performance as predictors of the need for the need for surgical intervention compared to MD/MX ratios and OP area when assessed via ROC curves. This suggests their potential utility in delivery planning and selection of surgical management strategies.

## 1195 | Effect of Intrapartum Antibiotic Prophylaxis on Systemic Maternal Inflammation During Labor Induction

Stephanie L. Pierce^1^; Jennifer Peck^1^; Erin Schone^1^; Dean Myers^1^; Rodney Edwards^2^

*^1^University of Oklahoma Health Sciences Center, Oklahoma City, OK; ^2^University of Florida College of Medicine, Gainesville, FL*

4:00 PM ‐ 6:00 PM

**Objective**: Labor is an inflammatory process, but excessive inflammation may contribute to abnormal labor by causing myometrial dysfunction, leading to cesarean delivery. We explore whether serum pro‐inflammatory cytokines during labor differ in women who receive intrapartum antibiotic prophylaxis (IAP) for group B streptococcus compared with those who do not.

**Study Design**: This prospective cohort study enrolled women age 18‐45 years, term, and undergoing labor induction (IOL) with cervical Foley and oxytocin. Exclusion criteria were known fetal demise and multiple gestation. We drew blood samples prior to IOL and 6‐12 and 18‐24 hours (h) after IOL start and compared pro‐inflammatory cytokine levels (IL‐1α, IL‐1β, IL‐6, IL‐8, GM‐CSF, IFN‐g, MCP‐1, and TNF‐α) in those who received IAP versus those who did not. We used linear mixed models to evaluate differences in labor cytokines between IAP groups over time, controlling for obesity, parity, race/ethnicity, chronic hypertension, preeclampsia and diabetes.

**Results**: 113 women (n = 59 did not receive IAP and n = 54 received IAP) were recruited. Cytokine concentrations significantly increased between pre‐labor and 12‐24h post‐IOL measures with the exception of IFN‐g, IL‐1α and TNF‐α (Figure). Overall, patients who received IAP had higher mean concentrations of IFN‐g (unadjusted geometric mean ratio [GMR] 1.27 [95% CI 1.05, 1.55]) and lower levels of TNF‐ α (unadjusted GMR 0.87 [95% CI 0.76, 0.99]; Table). IL‐1α levels did not differ by IAP status pre‐labor but were 80% greater for those with IAP at 6‐12h post‐IOL. TNF‐α concentrations at 6‐12h were significantly lower in those who received IAP compared with those who did not (GMR 0.84 [95% CI 0.71, 0.99]).

**Conclusion**: Intrapartum TNF‐α concentration was lower in those who received IAP for GBS compared with those who did not, while IFN‐g, IL‐1α concentrations were higher. Given the known effects of these cytokines on uterine contractility and labor, this could suggest a mechanism by which prophylactic antibiotics during labor induction may influence uterine activity and the risk of abnormal labor.

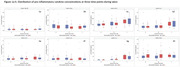

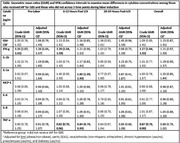

## 1196 | No Association Between Biological Therapeutics and Infections in Pregnant Patients with Autoimmune Disease

Beverley Cruz Alfonso; Mindy Pike; Christine Nolde; Manya Puri; Trent Bronnenberg; Alisa B. Kachikis; Stephen A. McCartney

*University of Washington, Seattle, WA*

4:00 PM ‐ 6:00 PM

**Objective**: The incidence of autoimmune disease (AID) peaks during reproductive years. Active AID increases pregnancy risks, making disease control during pregnancy crucial for improving maternal and fetal outcomes. Biologic therapeutics (biologics) targeting the immune system have significantly improved AID management. Current clinical guidelines generally recommend continuation of biologics during pregnancy to maintain clinical remission, however, there is conflicting evidence regarding risk of infections. This study evaluates the association between the use of biologics during pregnancy and peripartum infections.

**Study Design**: We performed a retrospective cohort study including pregnant patients with autoimmune disease from a single institution during 2013‐2023. Data on demographics, medical conditions, pregnancy, and therapeutics was abstracted from the medical record. The primary outcomes were rates of antepartum and postpartum infections between patients exposed to biologics during pregnancy versus unexposed. We hypothesized that the rate of antepartum and postpartum infections is higher in patients exposed to biologics during pregnancy compared to unexposed.

**Results**: We identified a total of 202 pregnancies in 131 individuals. 94 pregnancies (46.5%) were exposed to biologics, while 108 (53.5%) pregnancies were unexposed. Logistic regression adjusted for non‐independent data was used to assess the association between biologic exposure and antepartum and postpartum infections. There was no significant difference in the rates of overall antepartum (p = 0.66) or postpartum (p = 0.39) infections between the biologics exposed and unexposed pregnancies as well as no difference in the rates of individual infections.

**Conclusion**: There was no significant association between the use of biologics during pregnancy and the incidence of peripartum infections. Studies in larger populations are needed to confirm safety of biologics during pregnancy as they may be useful for treatment of pregnancy complications related to inflammation.

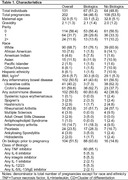

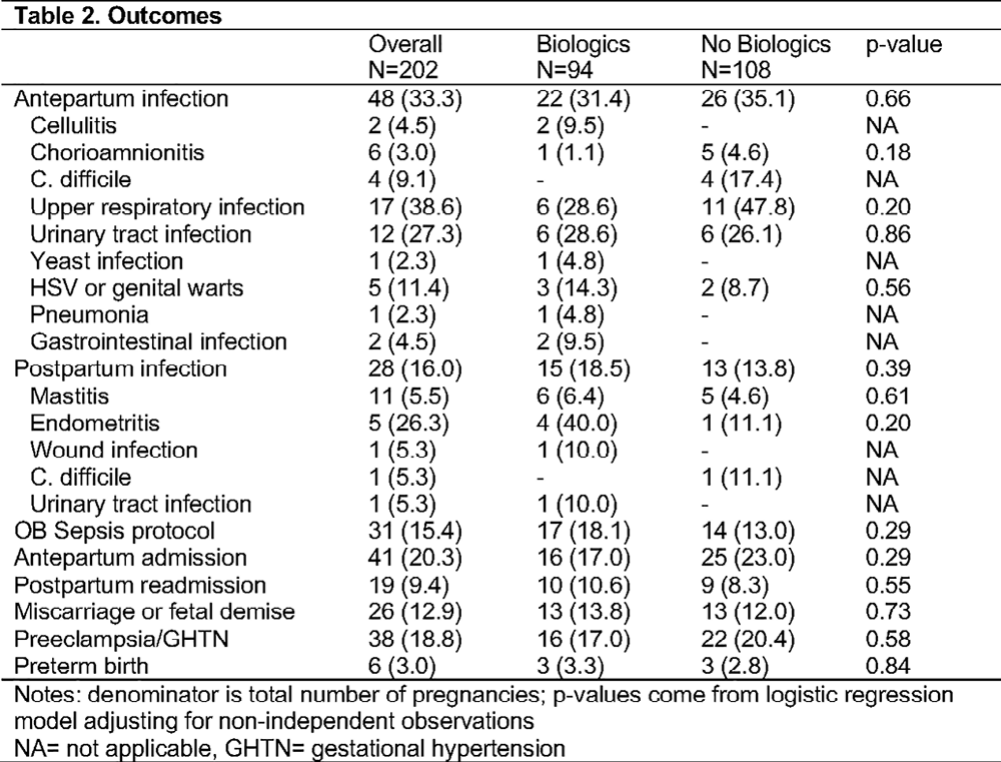

## 1197 | Vasa Previa: Factors Associated with Inpatient vs. Outpatient Antepartum Management

Sarah Heaps^1^; Stephen T. Chasen^2^

*^1^Weill Cornell Medicine, New York, NY; ^2^Weill‐Cornell Medical College, New York, NY*

4:00 PM ‐ 6:00 PM

**Objective**: When vasa previa is diagnosed, guidelines support recommendations about timing and route of delivery, as well as steroid administration. While elective admission to ensure proximity to care is common, evidence does not support a clear recommendation. Our objective was to compare patients with vasa previa managed as inpatients vs outpatients.

**Study Design**: This is a single institution cohort study of patients with a prenatal diagnosis of vasa previa from 2013‐2023. Cases resolved in the third trimester were excluded. Decisions about inpatient vs. outpatient management and timing of planned delivery were made by physicians and patients. Demographic factors and outcomes were obtained through chart review. Cohorts managed with elective admission for the indication of vasa previa were compared with those managed as outpatients. Mann‐Whitney U and Fisher's Exact test were used for statistical comparison.

**Results**: 89 patients were included, including 72 (80.9%) electively admitted vs. 17 (19.1%) managed as outpatients. The cohorts are compared in Table 1. The groups were of similar age and parity. A higher proportion of patients managed as outpatients had public insurance. There were no differences in the rate of a short cervical length or antepartum vaginal bleeding between the cohorts, and the rates of non‐scheduled Cesarean Delivery were similar. Betamethasone was administered at a median gestational age of 32‐33 weeks in both groups. Elective admission was associated with earlier gestational age at delivery overall, as well as earlier scheduled delivery. There were no stillbirths or neonatal deaths, and the rates of NICU admission were not significantly different between the groups.

**Conclusion**: Patients electively admitted for vasa previa do not appear to have been at higher risk for emergent delivery, though admission was associated with earlier delivery, including scheduled deliveries. The lower rate of admission in those with Medicaid could indicate a disparity in management, though further study is necessary. While our data do not rule out a benefit to routine admission, the benefits remain unproven.

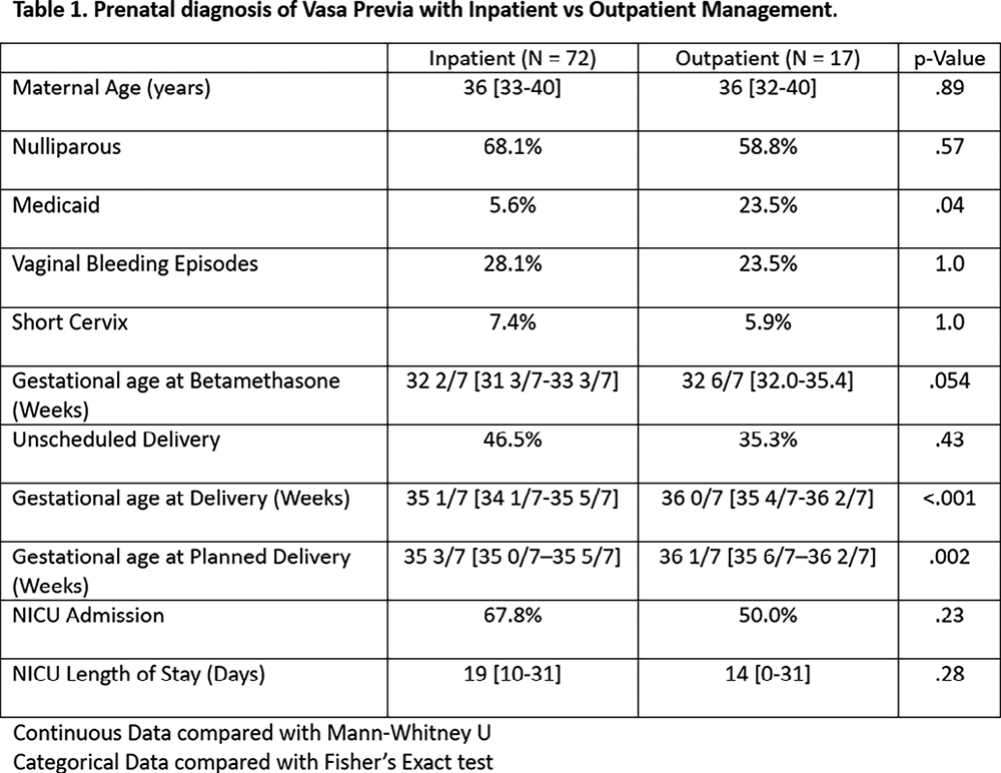

## 1198 | A Comparison Between Provider Density and Severe Maternal Morbidity Rates: A Georgia State Review

Suchitra Chandrasekaran; Ran Zhang; Jane Ellis; Sheree L. Boulet

*Emory University, Atlanta, GA*

4:00 PM ‐ 6:00 PM

**Objective**: Georgia's (GA) maternal morbidity & mortality rate is among the highest in the country. GA also has one of the highest rates of maternity deserts, defined as counties without obstetric care providers. Increasing the rates of non‐physician providers, including certified nurse midwives (CNM), nurse practitioners (NP), advanced practice nurses (APRN), & physician assistants (PA) has been proposed to address the physician provider shortage. We analyzed the change in non‐physician & physician provider density in relation to severe maternal morbidity (SMM) rates in GA from 2011 to 2020.

**Study Design**: We conducted a population‐based cohort study utilizing GA Birth Certificate Data from 2011‐2020 (n = 1,278,299). SMM included admission to intensive care, unplanned hysterectomy, eclampsia, & maternal blood transfusion. Changes in provider density for non‐physician & physician providers were calculated & compared with changes in SMM rates overall & stratified by rural residence.

**Results**: Between 2011‐2020, non‐physician provider density increased significantly *[CNM (60.3%), NP (164.4%), APRN (151.1%), & PA (62.3%)] (Table 1)*. This increase of >50% in non‐physician providers was seen in both rural and non‐rural settings. While overall physician density increased by 12.8%, physician density in rural settings declined by 5% (Table 1). The SMM rate per 10,000 births increased from 42.2 in 2011 to 65.2 in 2020, indicating a 54.5% increase. There was no significant difference between changes in non‐physician provider density & SMM rate *(p = 0.91)* or changes in physician provider density & SMM rate *(p = 0.61)*.

**Conclusion**: Our data demonstrate significant increases in CNM, NP, APRN, & PA rates across GA, including rural settings. While physician density in rural settings decreased, further efforts to provide directive education regarding maternal health in pregnancy to existing non‐physician providers could help optimize care quality and access. Leveraging the opportunity to train non‐physician providers in rural settings could help stratify care needs & facilitate transfer to higher level facilities when necessary.

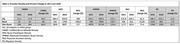

## 1199 | Second Trimester Placental Proteins are Associated with Maternal Vascular Malperfusion Among Patients with Adverse Outcomes

Sunitha Suresh^1^; Alexa A. Freedman^2^; Beth Plunkett^3^; Linda M. Ernst^1^

*^1^Endeavor Health, Evanston, IL; ^2^Northwestern University, Chicago, IL; ^3^Northshore Endeavor Health, Chicago, IL*

4:00 PM ‐ 6:00 PM

**Objective**: Adverse pregnancy outcomes (APO) are known to be heterogenous in etiology; many are linked to maternal vascular malperfusion (MVM) of the placenta. Second trimester placental proteins have poor prediction of APO. Placental proteins may have a greater association with APO related to a specific underlying mechanism such as MVM. The objective of this study is to evaluate levels of second trimester placental proteins and placental MVM among patients with an APO.

**Study Design**: This is a secondary analysis of a prospective cohort study of 9,289 low risk nulliparous patients with singleton gestation (nuMom2b). For this analysis, participants who met the following criteria were included: 1. APO defined as preterm birth (< 37 weeks), preeclampsia/eclampsia, small for gestational age infant, or stillbirth; 2. Maternal serum placental proteins (sFlt‐1, Adam‐1, VEGF, endoglin, bHcg, AFP, PlGF, Inhibin A, Papp‐A, sFlt/PlGF) collected between 16 and 21 weeks gestational age; 3. Placental pathology data. A single perinatal pathologist (LE) manually reviewed all placental data (lesions and additional comments included in the publicly available data set) to categorize participants as MVM absent or present. Placental protein levels were compared by MVM category using Wilcoxon rank sum.

**Results**: Of the 9,289 participants in the parent study, 2,091 (24%) had APO. Of these, 510 patients were eligible for inclusion, of which 216 (42%) had any presence of MVM. There was a significantly higher level of inhibin‐A and sFlt‐1/PlGF ratio and lower levels of VEGF and PlGF among those with MVM compared to those without (Table). A trend towards increase in sFlt‐1, ADAM‐12, and endoglin was additionally seen in MVM related APO.

**Conclusion**: Among individuals with APO, MVM is associated with significant differences in second trimester maternal serum placental proteins, especially those related to angiogenesis. Over half of APO were unrelated to MVM in this cohort. Classifying APO by placental pathology may lead to improvement in use and development of predictive biomarkers, given the underlying heterogeneity of APO.

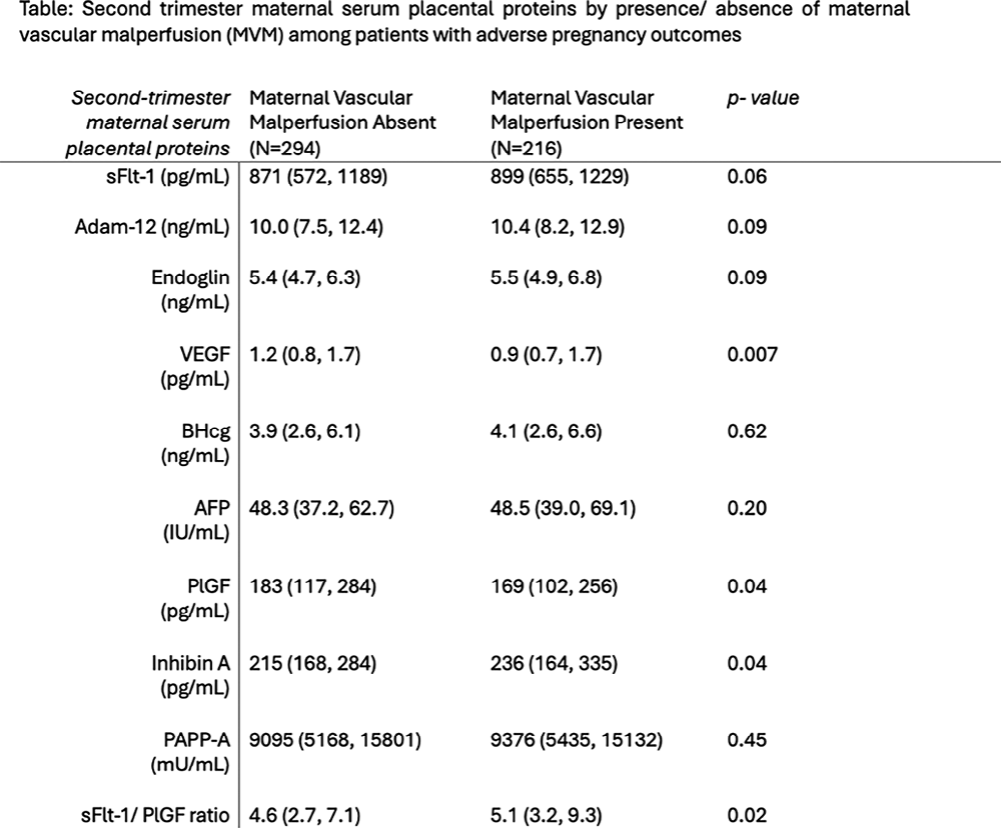

## 1200 | Neighborhood Socioeconomic Disadvantage and Antenatal Depressive Symptoms

Sydney Lammers^1^; Courtney Denning‐Johnson Lynch^2^; Jiqiang Wu^3^; Lynn M. Yee^4^; Michelle Miller^5^; Rebecca B. McNeil^6^; Brian M. Mercer^7^; Hyagriv Simhan^8^; Uma M. Reddy^9^; Robert M. Silver^10^; Samuel Parry^11^; George R. Saade^12^; Judith H. Chung^13^; William A. Grobman^3^; Kartik K. Venkatesh^3^

*^1^The Ohio State University, College of Medicine, Columbus, OH; ^2^The Ohio State University College of Medicine, Columbus, OH; ^3^The Ohio State University, Columbus, OH; ^4^Northwestern University Feinberg School of Medicine, Chicago, IL; ^5^Indiana University School of Medicine, Indianapolis, IN; ^6^Research Triangle International, Durham, NC; ^7^Case Western Reserve University, Cleveland, OH; ^8^University of Pittsburgh Medical Center, Pittsburgh, PA; ^9^Columbia University, New York, NY; ^10^University of Utah, Salt Lake City, UT; ^11^University of Pennsylvania, Philadelphia, PA; ^12^Macon & Joan Brock Virginia Health Sciences Eastern Virginia Medical School at Old Dominion University, Norfolk, VA; ^13^University of California‐ Irvine, Irvine, CA*

4:00 PM ‐ 6:00 PM

**Objective**: To evaluate whether neighborhood‐level socioeconomic disadvantage is associated with an increased risk of antenatal depressive symptoms.

**Study Design**: We conducted a secondary analysis of data from the Nulliparous Pregnancy Outcomes Study: Monitoring Mothers‐To‐Be, a prospective cohort of nulliparous pregnant individuals. Data were obtained throughout pregnancy by patient interviews and health record abstraction. Participant home addresses in the first trimester were geocoded at the census‐tract level. The exposure was socioeconomic disadvantage (categorized by tertile, with the least deprived [T1] as the reference) by the Area Deprivation Index (ADI). The outcome was depressive symptoms in the early third trimester (median gestational age: 26.9 weeks) by the Edinburgh Postnatal Depression Scale (EPDS), assessed as a score > 10, a cutoff which is commonly used for screening, and secondarily, > 13, a higher more specific cutoff. Modified Poisson regression was used and adjusted for maternal age, individual‐level social determinants (insurance, household income), and EPDS score in the first trimester.

**Results**: Of 8,678 assessed nulliparous individuals, the mean ADI was 44.4 (SD: 30.2), 16.1% had an EPDS score > 10, and 6.5% had an EPDS score > 13. In adjusted analyses, individuals who lived in a community with the highest tertile of ADI were nearly 40% more likely to screen positive for depressive symptoms in the third trimester compared with those in the lowest tertile (21.6% vs. 11.7%; aRR: 1.38; 95% CI: 1.16, 1.63). The association was similar at the higher EPDS screening threshold > 13 (9.9% vs. 3.8%; aRR: 1.75; 95% CI: 1.29, 2.38).

**Conclusion**: Neighborhood‐level socioeconomic disadvantage early in pregnancy was associated with an increased risk of screening positive for depressive symptoms in the third trimester, even after accounting for depressive symptoms in early pregnancy. These findings highlight the need for multilevel and structural interventions to address perinatal mental health.

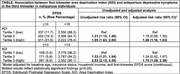

## 1201 | The Impact of Denying Abortion Access to Patients with Heart Failure: A Cost‐Effectiveness Analysis

Sydney McCarthy^1^; Emily Modlin^1^; Stacia C. Hickey^1^; Ava D. Mandelbaum^2^; Aaron B. Caughey^2^

*^1^Oregon Health and Science University, Portland, OR; ^2^Oregon Health & Science University, Portland, OR*

4:00 PM ‐ 6:00 PM

**Objective**: In June 2022, the *Dobbs v. Jackson Women's Health* Supreme Court decision eliminated constitutional protections for abortion access. This ruling has far‐reaching impacts on long‐term morbidity, mortality, and health‐related expenses for individuals capable of pregnancy. This study investigates the influence of abortion access on outcomes for pregnant individuals with stage 1 or 2 heart failure (HF) seeking abortion care based on the presence of a statewide abortion ban.

**Study Design**: A decision‐analytic model was developed to evaluate the outcomes and cost‐effectiveness associated with providing in‐state abortion services versus those incurred under a statewide abortion ban for individuals with HF seeking an abortion. Clinical outcomes included preeclampsia, preterm birth, pregnancy‐related major adverse cardiac events (MACE), disease progression, and mortality. The likelihood and financial burden of traveling out of state for abortion care was considered. The cost‐effectiveness threshold was set at $100,000/QALY. Model inputs were derived from the literature.

**Results**: In our theoretical cohort of 1,515 pregnant people with HF, access to abortion services led to 348 fewer cases of preeclampsia, 315 fewer preterm births, 218 fewer cases of pregnancy‐related MACE, 233 fewer cases of HF stage progression, and 112 fewer deaths annually relative to no access to abortion services (Table 1). In‐state abortion access was the dominant strategy, resulting in a $57,229,928 reduction in the costs and an increase in 3,182 QALYs.

**Conclusion**: Protecting in‐state abortion access for pregnant individuals with HF is a cost‐effective strategy, reducing adverse perinatal outcomes, enhancing resource allocation, and improving quality of life. These findings may inform policies to improves individual and societal outcomes for this vulnerable population in the context of restrictive abortion laws.

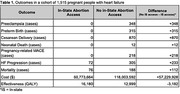

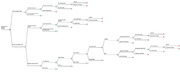

## 1202 | Assessing the Reliability of ChatGPT for Evaluation of Risk‐of‐Bias in Randomized Clinical Trials

Takeshi Nagao^1^; George R. Saade^2^; Tetsuya Kawakita^2^

*^1^The Jikei University School of Medicine, Minatoku, Tokyo; ^2^Macon & Joan Brock Virginia Health Sciences Eastern Virginia Medical School at Old Dominion University, Norfolk, VA*

4:00 PM ‐ 6:00 PM

**Objective**: To examine whether ChatGPT can be useful in the assessment of the risk‐of‐bias in Randomized Clinical Trials (RCTs) traditionally performed by reviewers performing systematic reviews.

**Study Design**: The study analyzed systematic reviews and meta‐analyses published in the American Journal of Obstetrics and Gynecology from 2021 to 2023, focusing on the risk‐of‐bias assessments in RCTs using Version 2 of the Cochrane risk‐of‐bias tool for randomized trials (RoB 2). ChatGPT‐4 was trained on RoB 2 guidelines and then tasked with evaluating each RCT across five domains: Randomization process, Deviations from intended interventions, Missing outcome data, Measurement of the outcome, and Selection of the reported result. Each domain, along with the overall bias, was rated as Low risk of bias, Some concerns, or High risk of bias. ChatGPT's evaluations were compared to those of human reviewers, as described in published papers, using Cohen's Kappa to determine agreement. Additionally, for each RCT, ChatGPT conducted three separate evaluations to calculate the intra‐ChatGPT agreement rate using Fleiss' Kappa.

**Results**: The study included 161 RCTs. The Cohen's Kappa scores for agreement between ChatGPT's evaluations and those of the reviewers were as follows: Domain 1 (Randomization) 0.485, Domain 2 (Deviations) 0.489, Domain 3 (Missing) 0.596, Domain 4 (Measurement) 0.446, Domain 5 (Selection) 0.484, and Overall bias 0.262. For the three separate evaluations conducted by ChatGPT on the same RCTs, the Fleiss' Kappa scores were Domain 1 (Randomization) 0.245, Domain 2 (Deviations) 0.359, Domain 3 (Missing) 0.555, Domain 4 (Measurement) 0.493, Domain 5 (Selection) 0.351, and Overall bias 0.760.

**Conclusion**: ChatGPT's risk‐of‐bias assessments moderately agreed with human reviewers across five domains but showed only fair agreement in overall bias assessment. Intra‐ChatGPT evaluations were inconsistent for certain domains (Randomization, Deviations, Selection), though the overall bias assessment showed substantial agreement.

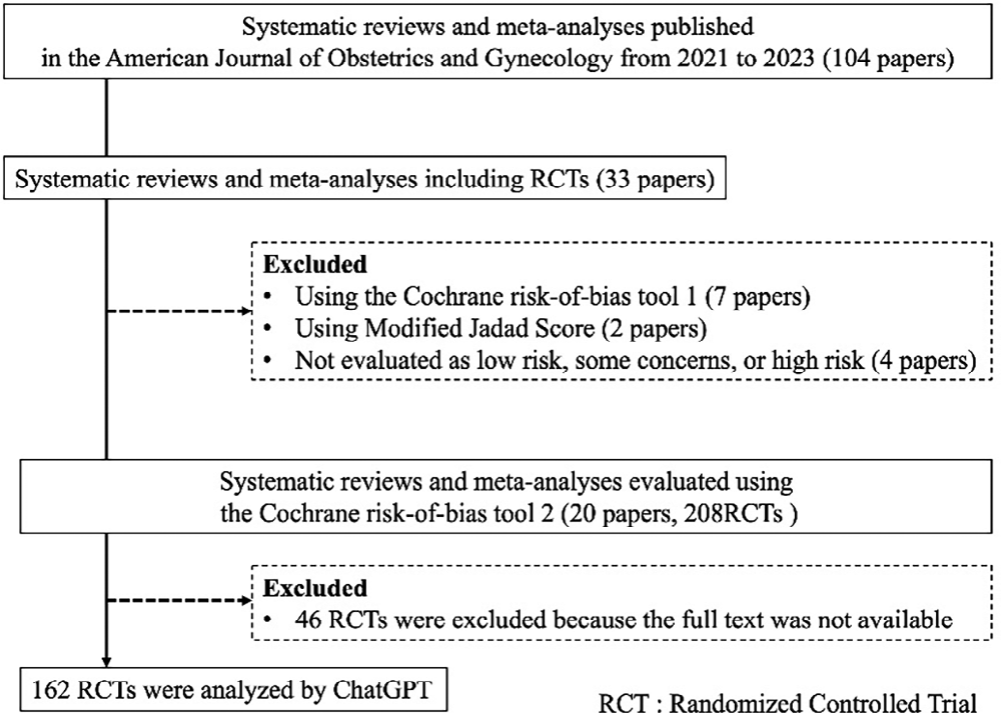

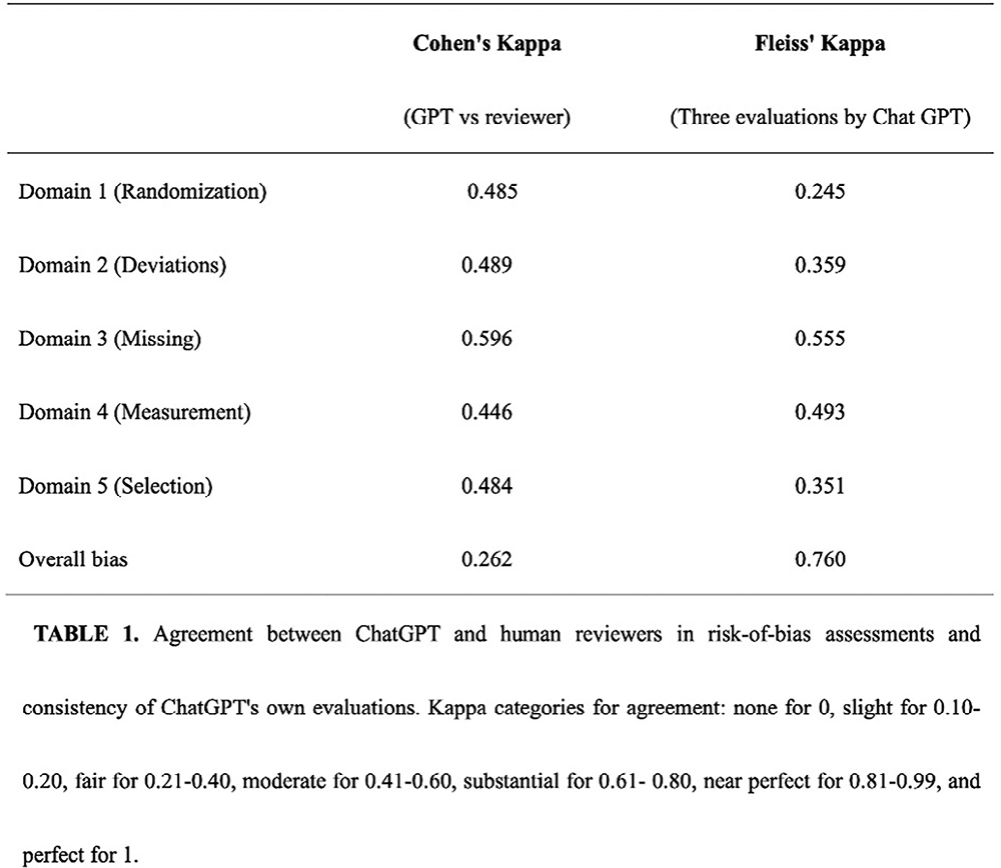

## 1203 | The effect of One‐Abnormal Glucose‐Value of the 100‐gram Oral‐Glucose Tolerance‐Test According to Carpenter‐and‐Coustan on Obstetrical‐Outcomes

Tal zilberman kimhi^1^; Yuri Perlitz^2^; Zohar Nachum^3^; Enav Yefet^2^

*^1^Tzafon Medical Center, tzafon medical center, HaZafon; ^2^Tzafon Medical Center, Poriya, HaZafon; ^3^Emak Medical Center, Afula, HaZafon*

4:00 PM ‐ 6:00 PM

**Objective**: To examine the effect of one abnormal glucose value only according to C&C criteria in 100 g OGTT on pregnancy outcomes.

**Study Design**: A population‐based retrospective cohort study was conducted involving women who delivered at Tzafon Medical Center in Israel between March 2012 and December 2022. The women were categorized into three groups: (1) those without GDM (either normal GCT or OGTT; N = 18,422), (2) those with one abnormal OGTT value according to C&C criteria (fasting glucose 95‐104, one hour 180‐189, two hours 155‐164, and three hours 140‐144 mg/dL; N = 371), and (3) those with GDM, defined as at least two abnormal OGTT values according to C&C (N = 431). Only the last group was officially diagnosed with GDM. The primary outcome measured was the rate of large for gestational age (LGA) neonates.

**Results**: Univariable and multivariable analyses are detailed in Tables 1 and 2. After adjusting for background characteristics, having one abnormal glucose value significantly increased the risk, compared to the control group, and similar to the values in the two pathologic values group, for the following outcomes: large for gestational age (LGA) neonates (adjusted OR 1.5, 95% CI [1.2‐2.0]), macrosomia (2.0, 95% CI [1.3‐2.8]), cesarean delivery (1.5, 95% CI [1.2‐1.9]), and hypertensive disease of pregnancy (2.0, 95% CI [1.3‐3.3]).

Univariable and multivariable analyses are detailed in Tables 1 and 2. After adjusting for background characteristics, having one abnormal glucose value significantly increased the risk, compared to the control group, and similar to the values in the two pathologic values group, for the following outcomes: large for gestational age (LGA) neonates (adjusted OR 1.5, 95% CI [1.2‐2.0]), macrosomia (2.0, 95% CI [1.3‐2.8]), cesarean delivery (1.5, 95% CI [1.2‐1.9]), and hypertensive disease of pregnancy (2.0, 95% CI [1.3‐3.3]).

**Conclusion**: Women with one abnormal OGTT value, according to the C&C criteria, had worse outcomes compared to normoglycemic women, and similar to those with GDM. Therefore, these women should be considered as having GDM.

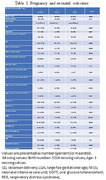

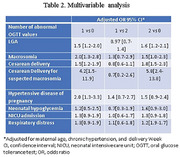

## 1204 | Risk of HDP in Nulliparas with Treated Versus Untreated Mood and Anxiety Disorders

Taylor M. Lewis^1^; Alyssa M. Hernandez^2^; Nadine Sunji^2^; Liyun Zhang^2^; Amy Y. Pan^2^; Anna Palatnik^2^

*^1^Medical College of Wisconsin, Oconomowoc, WI; ^2^Medical College of Wisconsin, Milwaukee, WI*

4:00 PM ‐ 6:00 PM

**Objective**: The impact of mood and anxiety disorders (MAD) on the risk of developing hypertensive disorders of pregnancy (HDP) remains unclear. This study aimed to explore the risk of HDP in nulliparas with either treated or untreated MAD.

**Study Design**: This was a secondary analysis of the Nulliparous Pregnancy Outcomes Study: Monitoring Mothers‐to‐Be (nuMoM2b). Nulliparas with singleton gestations ≥20 weeks without chronic hypertension were grouped as follows: no MAD, untreated MAD, and treated MAD. The diagnoses included in the MAD groups were depression, bipolar disorder, other mood disorders (dysthymia, seasonal affective disorder), and anxiety disorders (generalized anxiety disorder, panic disorder, obsessive‐compulsive disorder). Treatment was defined as the use of a psychotropic medication for a psychiatric indication. The risk of HDP was compared between groups using univariate and multivariate analyses.

**Results**: Of the 8,492 patients included in the analysis, 6,877 (81.0%) had no MAD diagnosis, 1,014 (11.9%) had an untreated MAD, and 601 (7.1%) had a treated MAD. Patients with a treated MAD were more likely to be older (p< 0.001), white (p< 0.001), non‐Hispanic (p< 0.001), college‐educated (p = 0.01), privately insured (p< 0.001), have a higher pre‐pregnancy BMI (p = 0.001), and score higher on the Edinburgh Postnatal Depression Scale (EPDS) in early pregnancy (p< 0.001). Compared to patients with no MAD, the risk of any HDP was similar across groups in both unadjusted and adjusted models (untreated MAD, aRR 0.96 [CI 0.85‐1.08]; treated MAD, aRR 0.92 [CI 0.79‐1.08]) (Table 1). The risk of preeclampsia was also not significantly different by MAD diagnosis or treatment exposure. To account for patients who may have undiagnosed or undertreated MAD, a multivariate model was created to examine the risk of any HDP or preeclampsia alone by EPDS score, which also showed no significant difference in risk (aRR 0.93 [CI 0.80‐1.07]).

**Conclusion**: This large cohort analysis found no significant statistical difference in the risk of HDP by MAD diagnosis, psychotropic treatment, or worst mood symptoms during pregnancy.

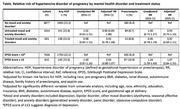

## 1205 | More Frequent Emergency care Utilization, Prior to 20w0d, is Associated with Higher Preterm Birth Rates

Tenisha D. Wilson^1^; Lauren Kucirka^2^; Catherine Vladutiu^3^; M. Kathryn Menard^2^

*^1^University of North Carolina Chapel Hill, Chapel Hill, NC; ^2^University of North Carolina, Chapel Hill, Chapel Hill, NC; ^3^University of North Carolina, Chapel HIll, Chapel Hill, NC*

4:00 PM ‐ 6:00 PM

**Objective**: Frequent emergency care (EC) utilization throughout pregnancy has been associated with adverse pregnancy outcomes, including preterm birth (PTB). Given that it has previously been shown that care coordination of at‐risk populations in North Carolina (NC) is associated with a reduced PTB rate, identifying early EC utilization as a risk factor for PTB affords an opportunity for intervention with the potential for introduction of a care coordination program in EC settings. To that end, the objective of this study was to assess whether the frequency of early EC utilization, prior to 20w0d, is associated with higher rates of PTB.

**Study Design**: Retrospective cohort study of pregnant Medicaid recipients who had a live birth in NC between 01/01/2014–12/31/2019, using linked birth records and NC Medicaid hospital claims. Early pregnancy EC use was defined as an encounter in OB triage or the emergency department prior to 20w0d. We categorized frequency of EC visits before 20w0d as follows: 0, 1, 2, or 3 visits. We built a multivariable modified Poisson regression model to analyze the association between early EC visits and PTB, adjusted for potential confounders.

**Results**: With increasing EC utilization prior to 20w0d (Table 1), the % of patients with PTB increased. Among those with zero EC visits prior to 20w0d, 0.53% delivered < 28w0d, compared to 0.86%, 1.02%, and 1.43%, in those with 1, 2, and 3 ED visits, respectively. A similar trend was observed among those who delivered between 28w0d‐33w6d and between 34w0d‐36w6d. In a multivariable model adjusted for confounders, a higher number of early ED visit was associated with an increased risk of PTB < 28 weeks (adjusted relative risk (aRR) = 1.35, 95% CI: 1.28‐1.42), 28w‐33w6d (aRR = 1.18, 95% CI: 1.15‐1.20), and >34 weeks (aRR = 1.13, 95% CI: 1.11‐1.15).

**Conclusion**: More frequent, early EC utilization is associated with a higher rate of PTB in every gestational age category assessed. Clinically, the study reveals an area of opportunity to initiate a prenatal care coordination plan as an intervention in the EC setting, which may potentially reduce the rate of PTB.

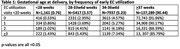

## 1206 | Is First Trimester Hemorrhage with Concurrent Anemia Associated with Placenta Accreta Spectrum?

Tess E.K Cersonsky^1^; Henri M. Rosenberg^2^; Eugenia Alleva^1^; Isabelle C. Band^1^; Calvin E. Lambert, Jr.^1^; Angela T. Bianco^1^

*^1^Icahn School of Medicine at Mount Sinai, New York, NY; ^2^Icahn School of Medicine at Mount Sinai, Division of Maternal‐Fetal Medicine, Icahn School of Medicine at Mount Sinai, NY*

4:00 PM ‐ 6:00 PM

**Objective**: First trimester hemorrhage may contribute to abnormal placentation. These abnormalities can have devastating consequences in the form of placenta accreta spectrum (PAS), which is associated with significant maternal morbidity and mortality. Subchorionic hematoma, a leading cause of first trimester hemorrhage, and anemia may contribute to placental hypoxia and aberrant placentation. We sought to establish if an association exists between anemia, hemorrhage, and PAS.

**Study Design**: his is a secondary analysis of a retrospective case‐control study using patient data from 2007 to 2022 at a large tertiary care center. Demographics and clinical characteristics of patients with and without PAS (diagnosed by histopathology or high index of clinical suspicion) were collected as well as complete blood count in early pregnancy. Bivariate analyses compared baseline case and control characteristics. Logistic regression was performed to assess risk of PAS according to the presence of first trimester hemorrhage with concurrent anemia (hemoglobin < 11 g/dL) while adjusting for relevant covariates.

**Results**: The final cohort included 88 patients with confirmed PAS and 27,126 controls. Rates of first trimester hemorrhage alone were not significantly different between groups (12.3% of those without PAS versus 17.0% of those with PAS, p = 0.2); however, those with PAS were more likely to have first trimester hemorrhage and concurrent anemia (0.4% of those without PAS versus 5.7% of those with PAS, p < 0.001). In multivariate logistic regression, first trimester hemorrhage and concurrent anemia was associated with higher odds of PAS than any other covariate, including prior cesarean or placenta previa (adjusted odds ratio = 16.6, 95% confidence interval 5.58 ‐ 39.7).

**Conclusion**: Patients with a first trimester hemorrhage and concurrent anemia have 16‐fold higher odds of PAS. This suggests that early pregnancy placental disruptions may significantly increase the risk for PAS. Further research into the pathophysiology of this process is warranted to fully understand this phenomenon.

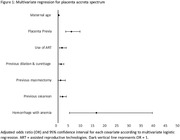

## 1207 | Use of Mean Arterial Blood Pressure to Predict Superimposed Preeclampsia with Severe Features

Tetsuya Kawakita^1^; Justin Leach^2^; On behalf of the CHAP Consortium

*^1^Macon & Joan Brock Virginia Health Sciences Eastern Virginia Medical School at Old Dominion University, Norfolk, VA; ^2^University of Alabama at Birmingham, Birmingham, AL*

4:00 PM ‐ 6:00 PM

**Objective**: To examine whether longitudinal assessment of mean arterial pressure (MAP) could predict the development of superimposed preeclampsia (SIPE) with severe features.

**Study Design**: This was a secondary analysis of the Chronic Hypertension and Pregnancy (CHAP) trial. We excluded individuals with missing or invalid information regarding SIPE diagnosis. Our primary outcome was SIPE with severe features. We fitted Bayesian joint longitudinal and time‐to‐event models (linear and non‐linear natural spline‐based) using MAP from baseline and throughout pregnancy prior to the diagnosis of SIPE or censoring as repeated measures. A hazard ratio (HR) with a 95% credible interval was estimated. The fitted models were evaluated using the area under the receiver‐operating curves (AUC) and Brier scores, estimated by 5‐fold cross‐validation 5 times.

**Results**: Of 2316 individuals with chronic hypertension, 600 (25.9%) developed SIPE with severe features. Compared to individuals without SIPE with severe features, those with SIPE with severe features had higher MAP, were more likely to be Black or smokers, and were more likely to have government insurance and diabetes (Table 1). Higher MAP was associated with an increased risk of SIPE with severe features (HR 1.15; 95% CI 1.12‐1.19), indicating that each mmHg increase in MAP was associated with a 15% increase in the risk of SIPE with severe features. AUCs  (Table 2) were low‐to‐moderate, with better performance for intervals later in pregnancy and shorter in duration (e.g., 0.60 at 20‐40 weeks to 0.71 at 34‐37 weeks). Similarly, Brier scores were low‐to‐moderate indicating poor performance. Estimated HRs and prediction performance were nearly indistinguishable between linear and spline‐based models.

**Conclusion**: While repeated measures of MAP are associated with developing SIPE with severe features, the predictive ability of longitudinal MAP alone was fair to moderate. Future studies should consider longitudinal MAP in modeling the risk of SIPE with severe features, but additional predictors are necessary to improve predictive performances.

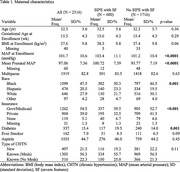

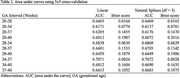

## 1208 | Association between Levels of Perceived Stress and Severity of Nausea and Vomiting in Pregnancy

Ummayhany F. Bharmal^1^; George R. Saade^2^; Tetsuya Kawakita^2^

*^1^Medical College Baroda, Medical College Baroda, Gujarat; ^2^Macon & Joan Brock Virginia Health Sciences Eastern Virginia Medical School at Old Dominion University, Norfolk, VA*

4:00 PM ‐ 6:00 PM

**Objective**: The role of psychosocial factors such as perceived stress in the severity of Nausea and Vomiting of Pregnancy (NVP) is unclear. Our objective was to determine the association between perceived stress levels and NVP severity.

**Study Design**: This was a secondary analysis of data from the Nulliparous Outcomes Study: Mothers to be (nuMoM2b). Perceived stress was assessed in the first trimester using the Perceived Stress Scale, categorized into mild (0‐14), moderate (15‐27), and severe (28‐40). NVP severity over the past 24 hours was assessed at 6‐13 (visit 1), 16‐21 (visit 2), and 22‐29 weeks (visit 3) using the Pregnancy Unique Quantification of Emesis and Nausea tool, categorizing symptoms as no symptoms (0‐3), mild (4‐6), moderate (7‐12), and severe (13‐15). The primary outcome was the moderate or severe NVP in each trimester. Modified Poisson regression with robust variance was used to calculate adjusted relative risks (aRR) and 95% confidence intervals (CI), controlling for potential confounders (mild stress as the referent).

**Results**: Of 8,867 participants, 5,279 (59.5%) had mild stress, 3,298 (37.2%) had moderate stress, and 290 (3.3%) had severe stress. Maternal characteristics and socioeconomic factors varied across the groups (Table 1). The risk of moderate or severe NVP is presented in Table 2. Individuals with severe stress had a significantly increased risk of having moderate or severe NVP in visit 1 (aRR 1.65, 95% CI 1.25‐2.13) and visit 2 (aRR 2.08, 95% CI 1.15‐3.77) when compared to those with mild stress. Individuals with moderate stress had a significantly increased risk of having moderate or severe NVP in all visits when compared to those with mild stress (visit 1 aRR 1.24, 95% CI 1.09‐1.40; visit 2 aRR 1.49, 95% CI 1.10‐2.02: visit 3 aRR 1.59, 95% CI 1.06‐2.41).

**Conclusion**: Elevated perceived stress levels show a significant association with increased severity of NVP throughout all trimesters, warranting the need for specific interventions in the management of NVP in high‐stress pregnancies.

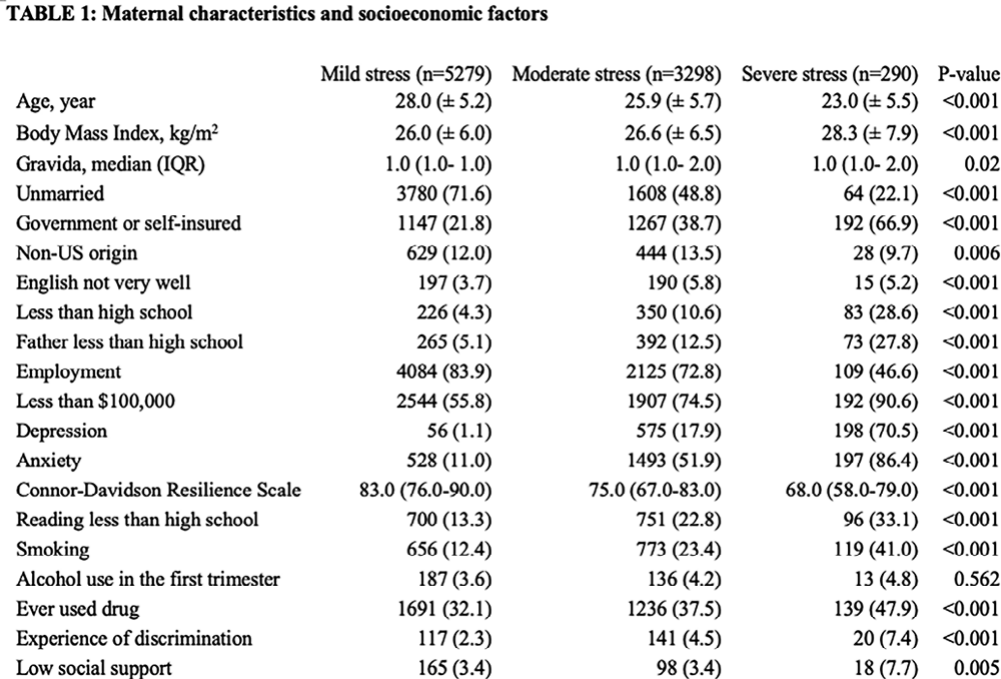

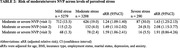

## 1209 | Hypertensive Control and Delivery Outcomes with Use of Oral Antihypertensives in Pregnancy for Non‐severe Hypertension

Tiffany Yang^1^; Hannah K. Agoglia^2^; David Garry^1^; Cassandra Heiselman^1^

*^1^Stony Brook University Hospital, Stony Brook, NY; ^2^Renaissance School of Medicine at Stony Brook University, Stonybrook, NY*

4:00 PM ‐ 6:00 PM

**Objective**: This study aims to determine if there are differences in pregnancy outcomes and hypertension control for patients on single versus multiple antihypertensive maintenance drug regimens.

**Study Design**: Retrospective chart review of adult pregnant patients who received prenatal care and delivered at a single academic institution with a diagnosed hypertensive disorder requiring antenatal antihypertensive maintenance medications started by 34 weeks GA or for a minimum of 2 weeks. Exclusion criteria were pregnancies of known anomalous fetus and multifetal gestations. Patients were then stratified by type and number of medications prescribed. Chi‐squared, Fischer's exact, and student's t‐test analysis were performed.

**Results**: 112 patients met inclusion criteria. 96 (85.7%) were prescribed a single drug regimen: labetalol 71(63.4%), metoprolol 2(1.8%), nifedipine 11 (9.8%), other 12 (10.7%); 16 (14.3%) were taking a combination of medications. There were no differences in age, obesity, race/ethnicity, insurance status, hypertensive diagnosis, maternal co‐morbidities, aspirin or tobacco use. More women requiring polytherapy had more history of preeclampsia versus those on monotherapy (89% vs 28%, p< 0.001), along with higher diastolic BP and MAP at first prenatal visit. Patients on multidrug regimens had higher rates of change in hypertensive diagnosis (i.e. chronic to preeclampsia) (p = 0.01), inpatient admission for blood pressure control (p = 0.002), higher BP and MAP at delivery (p< 0.001), earlier gestational age at delivery (p< 0.001), rates of medically indicated PTD (p< 0.001), cesarean delivery (p< 0.001), and longer maternal hospitalization (p = 0.02), as compared to the monotherapy group (Table 1).

**Conclusion**: Requiring multidrug antihypertensive regimens may be a proxy for pregnancies with poor hypertensive control, and furthermore a marker for poor maternal outcomes. Close supervision and attention to these patient populations may be warranted to optimize their pregnancy outcomes.

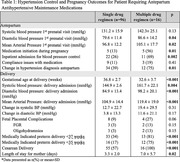

## 1210 | Maternal Delivery Outcomes at 35‐37 Versus 34 weeks after PPROM <34 weeks in the US

Timothy Wen^1^; Brittany Arditi^2^; Nasim C. Sobhani^3^; Cynthia Gyamfi‐Bannerman^4^; Adina R. Kern‐Goldberger^5^; Teresa C. Logue^6^; Maria Andrikopoulou^2^; Lanbo Yang^7^; Milli Desai^8^; Alexander M. Friedman^2^; Kartik K. Venkatesh^9^

*^1^University of California, San Diego, Irvine, CA; ^2^Columbia University Irving Medical Center, New York, NY; ^3^UCSF, San Francisco, CA; ^4^University of California, San Diego, San Diego, CA; ^5^Cleveland Clinic Lerner College of Medicine, Cleveland, OH; ^6^Christiana Care Health System, Newark, DE; ^7^Tulane University School of Medicine, Tulane University School of Medicine, LA; ^8^University of California, San Francisco, University of California, San Diego, CA; ^9^The Ohio State University, Columbus, OH*

4:00 PM ‐ 6:00 PM

**Objective**: International data suggest that expectant management of preterm premature rupture of membranes (PPROM) up to 37 weeks is safe compared with 34‐week delivery. U.S. data on this evolving practice is lacking. We examined maternal outcomes and healthcare utilization between delivery at 35‐37 versus 34 weeks after a PPROM diagnosis < 34 weeks.

**Study Design**: This study included livebirth delivery hospitalizations in the Nationwide Inpatient Sample from 2016‐2021 that had a diagnosis of PPROM < 34 weeks with consequent delivery between 34‐37 weeks. The exposure was timing of delivery defined as 34+0 to 34+6 versus 35+0 to 37+6 weeks per best obstetrical estimate of gestational age in completed weeks at delivery per National Center for Health Statistics recommendations. Outcomes at delivery included a composite of maternal infection (chorioamnionitis, endometritis, sepsis, shock), severe maternal morbidity (SMM) with and without transfusion, and cesarean delivery. Secondary outcomes included postpartum length of stay (ppLOS) and total hospitalization costs. Logistic regression models adjusted for payer type, self‐reported race as a social construct, obstetric comorbidity index, hospital characteristics, and birth year.

**Results**: Among 73,365 deliveries between 34‐37 weeks with a PPROM diagnosis < 34 weeks, 920 (1.3%) delivered between 35‐37 weeks. Delivery between 35‐37 weeks was associated with a higher risk of maternal infection (3.3% vs. 12.5%, aOR: 3.74, 95% CI: 2.37, 5.91) and cesarean delivery (28.4% vs. 44.7%, aOR 1.69, 95% CI: 1.22, 2.34) versus delivery at 34 weeks. There were no differences in SMM by timing of delivery (Figure 1). Hospitalization costs were three‐fold higher with delivery between 35‐37 weeks ($22,487 vs. $6,669), but median ppLOS were similar between the two groups (Figure 2).

**Conclusion**: Later delivery between 35+0 to 37+6 weeks following PPROM < 34 weeks was associated with higher likelihood of infectious morbidity and cesarean delivery compared with delivery between 34+0 to 34+6 weeks. These national U.S. observational data contrast with recent international trials.

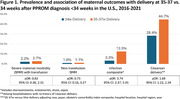

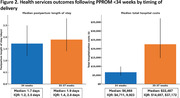

## 1211 | Firearm‐Related Injuries and Severe Maternal Morbidity in the United States, 2017‐2021

Timothy Wen^1^; Brittany Arditi^2^; Cynthia Gyamfi‐Bannerman^3^; Adina R. Kern‐Goldberger^4^; Nasim C. Sobhani^5^; Teresa C. Logue^6^; Maria Andrikopoulou^2^; Chia‐Ling Nhan‐Chang^3^; Alexander M. Friedman^2^; Kartik K. Venkatesh^7^

*^1^University of California, San Diego, Irvine, CA; ^2^Columbia University Irving Medical Center, New York, NY; ^3^University of California, San Diego, San Diego, CA; ^4^Cleveland Clinic Lerner College of Medicine, Cleveland, OH; ^5^UCSF, San Francisco, CA; ^6^Christiana Care Health System, Newark, DE; ^7^The Ohio State University, Columbus, OH*

4:00 PM ‐ 6:00 PM

**Objective**: Firearm violence is a preventable public health crisis in the United States and a primary cause of maternal mortality, but its national impact on perinatal outcomes remains to be fully examined. We examined trends in firearm‐related injuries (FRI) during pregnancy, and their association with severe maternal morbidity (SMM) and adverse pregnancy outcomes (APOs).

**Study Design**: We utilized all delivery hospitalizations in the Nationwide Inpatient Sample from 2017‐2021. The exposure was any firearm‐related injuries at delivery hospitalization. We analyzed trends of FRI overall. The primary outcome was non‐transfusion severe maternal morbidity (ntSMM) and secondarily SMM with transfusion, inpatient mortality, stillbirth, critical care procedures, and preterm birth < 37 weeks. We evaluated the association between FRI and perinatal outcomes using survey‐adjusted logistic regression models, adjusting for demographic factors, hospital factors, delivery year, and obstetric comorbidity score.

**Results**: Among 17.5 million deliveries, 6,365 were complicated by FRI at a rate of 3.6 per 10,000 delivery hospitalizations. Deliveries with FRI significantly increased from 2.7 to 3.6 per 10,000 delivery hospitalizations from 2017 to 2021 (p< 0.01), with a peak of 5.0 per 10,000 delivery hospitalizations in 2020 [Figure 1]. In adjusted analyses, FRI was associated with over a five‐fold increased likelihood of ntSMM (5.1% vs 0.8%; aOR 5.21, 95% CI: 3.91, 6.95). In addition, FRI was associated with higher likelihood of adverse of all APOs, with the highest likelihood of needing critical care procedures (aOR 8.47, 95% CI: 4.64, 15.44) and inpatient mortality (aOR 19.19, 95% CI: 4.66, 79.07) [Figure 2].

**Conclusion**: Firearm‐related injury at delivery hospitalization has increased across the US from 2017 to 2021 and is associated with an increased risk of SMM as well as APOs. Preventing gun violence has the potential reduce perinatal morbidity and mortality and should be an urgent public health priority.

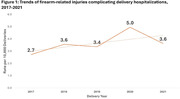

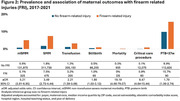

## 1212 | Determining time Course of Complex Gastroschisis Development in Fetal Lambs with Standardized Abdominal Wall Defect

Tomohiro Arai^1^; Jan A. Deprest^1^; Marianna Scuglia^2^; David Basurto^2^; Wasinee Tianthong^1^; Watananirum Kanokwaroon^2^; Emma Van Den Eede^1^; Simen Vergote^2^; Tom Bleeser^2^; Francesca M. Russo^2^; Keswani Sundeep^3^; Michael A. Belfort^4^; Luc Joyeux^3^

*^1^My FetUZ Fetal Research Center, Universitary Hospital Leuven, Leuven, Vlaams‐Brabant; ^2^My FetUZ Fetal Research Center, Department of Development and Regeneration, Cluster Woman and Child, Biomedical Sciences, KU Leuven, Leuven, Belgium, Leuven, Vlaams‐Brabant; ^3^Department of Surgery, Division of Pediatric Surgery, Baylor College of Medicine and Texas Children's Hospital, Houston, TX; ^4^Texas Children's Hospital and Baylor College of Medicine, Houston, TX*

4:00 PM ‐ 6:00 PM

**Objective**: Complex gastroschisis is the severe type of gastroschisis, characterized by intestinal atresia, stenosis, volvulus, perforation, or necrosis. A systematic review (Arai, *Prenatal Diagnosis* 2024) demonstrated complex gastroschisis can be reliably surgically induced in fetal lambs using a 1.5 cm left lateral abdominal wall defect stabilized by a 1.5 cm silicone ring. It results in a 60% complex gastroschisis and a 40% fetal death rate *by term*. We aimed to identify from what time point after induction gastroschisis becomes complex.

**Study Design**: Gastroschisis was induced in 75‐day (term = 145d) fetal lambs, which were harvested between 13 and 21d post‐induction. Primary outcome was the occurrence of *complex* gastroschisis; secondary outcomes included *in‐vivo* ultrasound characteristics of the bowel, *ex‐vivo* bowel contractility, and histology (Fig. 1). We first assessed 6 animals at 13‐16d and 6 at 17‐21d post‐induction to sufficiently power the study.

**Results**: In the first 12 lambs, 0/6 developed *complex* gastroschisis at 13‐16d, whereas this was present in 4/6 at 17‐21d. Hypothesizing a complex gastroschisis rate of 10% at 13‐16d and 65% at 17‐21d, we added 6 lambs (total:18). Finally, 17‐21d post‐induction, *complex* gastroschisis was present in the vast (5/8 = 63%) majority, whereas it was simple at 13‐16d (9/10 = 90%, p = 0.0265, 2‐sided Fisher exact). Bowels from lambs with complex gastroschisis display inappropriate bowel relaxation on contractility testing and a thicker muscular layer on histology. Remarkably, lambs with complex gastroschisis presented with intra‐abdominal bowel dilation on prenatal ultrasound at obduction; a 2.58mm cut‐off could identify complex with 100% sensitivity and 60% specificity.

**Conclusion**: Gastroschisis can be reliably induced in fetal lambs. This leads to simple gastroschisis early on (13‐16d), whereafter (17‐21d) complex gastroschisis becomes very common. Complex gastroschisis coincided with intra‐abdominal bowel dilation on ultrasound, which may be used as a non‐invasive biomarker. This defines a time window of opportunity and signs to guide experiments for prenatal intervention.

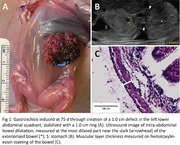

## 1213 | Neighborhood Socioeconomic Disadvantage and Severe Maternal Morbidity

Tracy Caroline Bank^1^; Janet Catov^2^; Jiqiang Wu^3^; Lynn M. Yee^4^; David M. Haas^5^; Rebecca B. McNeil^6^; Brian M. Mercer^7^; Hyagriv Simhan^8^; Uma M. Reddy^9^; Robert M. Silver^10^; Samuel Parry^11^; George R. Saade^12^; Judith H. Chung^13^; Courtney Denning‐Johnson Lynch^14^; William A. Grobman^3^; Kartik K. Venkatesh^3^

*^1^The Ohio State University Wexner Medical Center, Columbus, OH; ^2^Magee‐Womens Hospital, Pittsburgh, PA; ^3^The Ohio State University, Columbus, OH; ^4^Northwestern University Feinberg School of Medicine, Chicago, IL; ^5^Indiana University School of Medicine, Indianapolis, IN; ^6^Research Triangle International, Durham, NC; ^7^Case Western Reserve University, Cleveland, OH; ^8^University of Pittsburgh Medical Center, Pittsburgh, PA; ^9^Columbia University, New York, NY; ^10^University of Utah, Salt Lake City, UT; ^11^University of Pennsylvania, Philadelphia, PA; ^12^Macon & Joan Brock Virginia Health Sciences Eastern Virginia Medical School at Old Dominion University, Norfolk, VA; ^13^University of California‐ Irvine, Irvine, CA; ^14^The Ohio State University College of Medicine, Columbus, OH*

4:00 PM ‐ 6:00 PM

**Objective**: To determine whether neighborhood socioeconomic disadvantage in early pregnancy was associated with severe maternal morbidity (SMM) at delivery hospitalization.

**Study Design**: This was a secondary analysis from the prospective cohort Nulliparous Pregnancy Outcomes Study: Monitoring Mothers‐To‐Be study. Participant residential addresses in the first trimester were geocoded at the census‐tract level. The exposure was neighborhood socioeconomic disadvantage, measured by the Area Deprivation Index (ADI) and modeled in quartiles from the least (quartile 1, Q1, reference) to most (Q4) disadvantage. The outcome was SMM, based on the Centers for Disease Control and Prevention definition. Secondarily, SMM without transfusion was assessed. Modified Poisson regression with robust error variance was used and adjusted for individual‐level covariates: age, pre‐pregnancy body mass index, chronic hypertension, and diabetes in pregnancy. We also evaluated whether any observed associations between ADI and SMM differed by race/ethnicity, understood as a social construct (effect modification).

**Results**: Among 9,588 nulliparous individuals, 2.3% (n = 221) experienced any SMM and 0.5% (n = 48) experienced non‐transfusion SMM. Individuals who self‐identified as non‐Hispanic Black were more likely to experience SMM than non‐Hispanic White individuals (3.9% vs. 2.1% p < 0.001). Individuals living in the most disadvantaged neighborhoods (Q4) were more likely to experience SMM compared with those in the least disadvantaged neighborhoods (Q1) (3.4% vs 2.1%; aRR 1.73; 95% CI 1.17, 2.58) (Table). In secondary analyses, the association was also significant for non‐transfusion SMM (1.0% vs 0.3%; aRR 2.82; 95% CI 1.15, 6.93). There was no evidence of effect modification by self‐reported race and ethnicity (interaction p >0.05).

**Conclusion**: Nulliparous individuals living in the most disadvantaged US neighborhoods were at increased risk of experiencing SMM. Known racial and ethnic disparities in SMM may be related to differential neighborhood‐level social determinants.

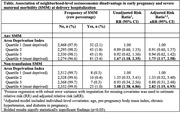

## 1214 | Antepartum Depressive Symptoms and Severe Maternal Morbidity

Tracy Caroline Bank^1^; Janet Catov^2^; Jiqiang Wu^3^; Lynn M. Yee^4^; David M. Haas^5^; Rebecca B. McNeil^6^; Brian M. Mercer^7^; Hyagriv Simhan^8^; Uma M. Reddy^9^; Robert M. Silver^10^; Samuel Parry^11^; George R. Saade^12^; Judith H. Chung^13^; Courtney Denning‐Johnson Lynch^14^; William A. Grobman^3^; Kartik K. Venkatesh^3^

*^1^The Ohio State University Wexner Medical Center, Columbus, OH; ^2^Magee‐Womens Hospital, Pittsburgh, PA; ^3^The Ohio State University, Columbus, OH; ^4^Northwestern University Feinberg School of Medicine, Chicago, IL; ^5^Indiana University School of Medicine, Indianapolis, IN; ^6^Research Triangle International, Durham, NC; ^7^Case Western Reserve University, Cleveland, OH; ^8^University of Pittsburgh Medical Center, Pittsburgh, PA; ^9^Columbia University, New York, NY; ^10^University of Utah, Salt Lake City, UT; ^11^University of Pennsylvania, Philadelphia, PA; ^12^Macon & Joan Brock Virginia Health Sciences Eastern Virginia Medical School at Old Dominion University, Norfolk, VA; ^13^University of California‐ Irvine, Irvine, CA; ^14^The Ohio State University College of Medicine, Columbus, OH*

4:00 PM ‐ 6:00 PM

**Objective**: The relationship between maternal mental health during pregnancy and consequent severe maternal morbidity (SMM) remains to be investigated. Therefore, we examined whether antepartum depressive symptoms in early pregnancy were associated with SMM at delivery hospitalization.

**Study Design**: This was a secondary analysis from the prospective cohort Nulliparous Pregnancy Outcomes Study: Monitoring Mothers‐To‐Be study. Nulliparous pregnant individuals were followed from the first trimester through delivery at 8 centers in the United States. The Edinburgh Postnatal Depression Scales (EPDS) was administered at 11‐13 weeks’ gestation. The EPDS was assessed categorically at ≥10 and ≥13, which are thresholds commonly used in clinical practice. The outcomes were SMM (including transfusion) and SMM (without transfusion) at delivery hospitalization. SMM was identified based on the Centers for Disease Control and Prevention definition. Modified Poisson regression with robust error variance was used and adjusted for age, insurance status, tobacco use, and Area Deprivation Index.

**Results**: Among 8,784 nulliparous individuals, 2.3% experienced any SMM and 0.5% had non‐transfusion SMM. In early pregnancy (median gestational age: 12.0 weeks; IQR: 11.0,13.0), 17.2% of individuals had EPDS scores ≥10 and 7.1% ≥13. In univariable analysis, having an EPDS ≥10 was associated with a greater frequency of SMM (3.0% vs 2.1%; RR: 1.42; 95% CI: 1.02, 1.96), although this did not remain statistically significant after adjustment (Table). Individuals who met the higher EPDS threshold of ≥13 had a statistically significant increase in risk of SMM without transfusion in both univariable (1.1% vs. 0.4%, RR 2.53, 95% CI: 1.13, 5.67) and multivariable analyses (aRR: 3.12; 95% CI: 1.11, 8.81).

**Conclusion**: In a prospective cohort of nulliparous individuals from across the US, depressive symptoms in early pregnancy, defined by an EPDS score ≥13 was associated with an increased risk of SMM without transfusion.

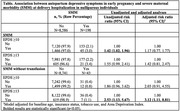

## 1215 | Association between Antenatal low‐dose Aspirin use and de Novo Postpartum Hypertension Incidence

Ukachi N. Emeruwa^1^; Chaukim Peters^2^; Kysha Mercader^3^; Minhazur R. Sarker^1^; Elizabeth Nicole Teal^1^; Gregory W. Poorman^4^; Barbara Levy^4^; Patrick Edmundson^4^; Maryam Tarsa^5^; Cynthia Gyamfi‐Bannerman^1^

*^1^University of California, San Diego, San Diego, CA; ^2^St. George's University School of Medicine, St. George's, Virgin Islands, U.S.; ^3^University of California, San Diego School of Medicine, La Jolla, CA; ^4^Dorsata, Inc., Arlington, VA; ^5^University of California San Diego, San Diego, CA*

4:00 PM ‐ 6:00 PM

**Objective**: Low dose aspirin (LDA) mitigates antenatal preeclampsia risk. Data is scarce regarding LDA effectiveness in preventing de novo postpartum hypertension (dnPPHTN), a growing contributor to maternal morbidity and mortality. We investigated the association between antenatal LDA prescription and dnPPHTN in a diverse multicenter cohort.

**Study Design**: We conducted a retrospective cohort study of patient data extracted from a multicenter electronic medical record system, including patients delivering from 2017 to 2024 from 90 locations and ∼320 providers. Patients who met ACOG criteria for LDA 81 mg once daily throughout pregnancy for preeclampsia risk reduction were included in the analysis. Exclusion criteria were: preexisting HTN, congenital heart disease, or pregnancy‐related HTN in the index pregnancy. Patients were grouped by LDA exposure (based on provider completion of prescription/recommendation). The primary outcome was dnPPHTN incidence, defined as “postpartum HTN” recorded at the postpartum encounter or on the delivery outcome form. We fit logistic regression models for dnPPHTN with unadjusted and adjusted odds ratios (OR, aOR) presented as measures of association.

**Results**: A total of 15,899 patients met criteria for LDA for preeclampsia risk reduction and did not develop hypertension antenatally; of those 10209 (64%) had been appropriately prescribed LDA. The overall incidence of dnPPHTN was 2.9% (n = 466). (Table 1) LDA‐exposed patients had an increased odds of dnPPHTN compared to LDA‐unexposed patients (OR 1.4, 95% CI 1.2,1.7). After controlling for baseline differences in clinically‐relevant risk factors and other clinical characteristics (Table 2), this association persisted (aOR 1.4, 95% CI 1.1,1.8).

**Conclusion**: Patients at increased risk of preeclampsia by ACOG criteria who are prescribed LDA remain at elevated risk for HTN in the postpartum period. Given limitations in assessing adherence and unmeasured confounders, prospective trials may help elucidate whether aspirin serves any preventative role beyond delivery.

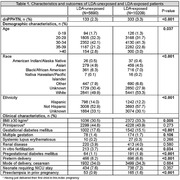

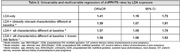

## 1216 | Comparing Perinatal Outcomes between Women with Subcapsular Liver Hematoma versus Preeclampsia ‐ A Population‐Based Study

Uri Amikam^1^; Ahmad badeghiesh^2^; Haitham Baghlaf^3^; Michael H. Dahan^4^; Richard Brown^4^

*^1^Lis Maternity & Women's Hospital, Tel‐Aviv, Tel Aviv; ^2^Department of Obstetrics and Gynecology, King Abdulaziz University, Rabigh Branch, Rabigh, Makkah; ^3^Department of Obstetrics and Gynecology, University of Tabuk, Tabuk, Saudi Arabia, Tabuk; ^4^McGill University, Montreal, PQ*

4:00 PM ‐ 6:00 PM

**Objective**: Subcapsular Liver Hematoma (SLH) is a rare life‐threatening complication of preeclampsia and the data regarding it is scarce. We aimed to compare pregnancy and perinatal outcomes in women with preeclampsia with and without an SLH.

**Study Design**: We conducted a retrospective cohort study using the Healthcare Cost and Utilization Project, Nationwide Inpatient Sample (HCUP‐NIS). Included in the study were all pregnant women who delivered or had a maternal death in the US between 2004‐2014 with an ICD‐9 diagnosis of preeclampsia. We divided the cohort into women with a diagnosis of SLH (study group) and women without an SLH (control group). Pregnancy and perinatal outcomes were compared between the two groups using multivariate logistic regression models adjusting for potential confounders.

**Results**: Overall, 327,485 women met the inclusion criteria. Amongst them, 119 women had a diagnosis of SLH. Women with an SLH, compared to those without, were more likely to be older (p = 0.002); had a higher BMI (BMI >30kg/m^2^) (p = 0.004); and suffered from chronic hypertension (p = 0.007). Patients in the SLH group, compared to those without, had a higher rate of eclampsia (aOR 15.23, 95% CI 4.82‐48.16, p< 0.001); and placental abruption (aOR 8.87, 95% CI 5.62‐14.01, p< 0.001). Additionally, they had a higher rate of cesarean delivery (aOR 3.02, 95% CI 1.95‐4.68, p< 0.001), postpartum hemorrhage (aOR 3.5, 95% CI 2.24‐5.48, p< 0.001), packed red blood cell transfusion (aOR 23.58, 95% CI 16.41‐33.89), disseminated intravascular coagulation (DIC) (aOR 23.11, 95% CI 14.67‐36.41, p< 0.001), and maternal death (aOR 98.48, 95% CI 36.4‐266.45, p< 0.001). Regarding neonatal outcomes, patients with an SLH, compared to those without, had a higher rate of intrauterine fetal demise (IUFD) (aOR 30.69, 95% CI 19.36‐48.67, p< 0.001).

**Conclusion**: Women diagnosed with an SLH had a higher incidence of myriad pregnancy and perinatal complications, including DIC, maternal death, and IUFD, as compared to women with preeclampsia alone, highlighting the severity of this condition.

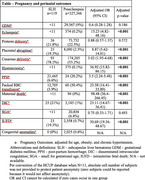

## 1217 | Rare Inflammatory Arthritis and Obstetric and Neonatal Outcomes ‐ A Population‐Based Study

Uri Amikam^1^; Ahmad badeghiesh^2^; Haitham Baghlaf^3^; Richard Brown^4^; Michael H. Dahan^4^

*^1^Lis Maternity & Women's Hospital, Tel‐Aviv, Tel Aviv; ^2^Department of Obstetrics and Gynecology, King Abdulaziz University, Rabigh Branch, Rabigh, Makkah; ^3^Department of Obstetrics and Gynecology, University of Tabuk, Tabuk, Saudi Arabia, Tabuk; ^4^McGill University, Montreal, PQ*

4:00 PM ‐ 6:00 PM

**Objective**: Inflammatory Arthritis (IA) is a common pathology among reproductive‐aged women. While abundant data exists regarding pregnancy outcomes in the more common IA subtypes, data is scarce regarding these outcomes in rare IA subtypes. We aimed to compare pregnancy and perinatal outcomes between women who suffered from rare types of IA and those who did not.

**Study Design**: A retrospective cohort study using the Healthcare Cost and Utilization Project, Nationwide Inpatient Sample (HCUP‐NIS). Included in the study were all pregnant women who delivered or had a maternal death in the US (2004‐2014) with an ICD‐9 diagnosis of any of the following rare IA subtypes: Arthritis Nodosa, Acute Febrile Mucocutaneous Lymph node, Hypersensitivity Angiitis, Wegner Granulomatosis, Giant Cell Arteritis, and Takayasu Arteritis. We divided the cohort into women with rare IA (study group) and women without (control group). Obstetric and perinatal outcomes were compared between the two groups using multivariate logistic regression adjusting for potential confounders.

**Results**: A total of 9,096,788 women met the inclusion criteria. Amongst them, 335 women (3.7/100,000) had a diagnosis of rare IA. Women with rare IA, compared to those without, were more likely to be older; Caucasian; in the highest income quartile; be insured by private insurance; and suffer from obesity, pregestational diabetes mellitus, thyroid disorders, and chronic hypertension (p< 0.05, all). Patients in the rare IA group, compared to those without, had a higher rate of hypertensive disorders of pregnancy (HDP) (aOR 1.89, 95% CI 1.4‐2.56, p< 0.001); preterm delivery (aOR 1.76, 95% CI 1.28‐2.42, p< 0.001); and blood products transfusion (aOR 3.68, 95% CI 2.14‐6.34, p< 0.001); with lower rates of spontaneous vaginal delivery (SVD) (aOR 0.76, 95% CI 0.61‐0.94, p = 0.012); and a higher rate of congenital anomalies (aOR 4.1, 95% CI 2.03‐8.31, p< 0.001).

**Conclusion**: Women with rare IA had a higher incidence of maternal complications, including HDP and preterm delivery, as well as an increased risk of congenital anomalies.

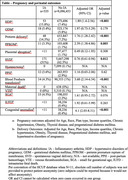

## 1218 | The social Vulnerability (SV) Index is Related to Toxicology Testing During Pregnancy

Virginia Lijewski^1^; Jeanelle Sheeder^2^; Heather Straub^2^

*^1^University of Colorado, School of Medicine, Aurora, CO; ^2^University of Colorado School of Medicine, Aurora, CO*

4:00 PM ‐ 6:00 PM

**Objective**: To compare the demographic profiles and SV of pregnant patients who did and did not receive urine toxicology (uTox) testing. We also wished to determine if the declaration of the opioid epidemic as a National Public Health Emergency (PHE) on 10/27/17 led to increased testing rates. We hypothesized that patients receiving uTox testing were more likely to be SV than those who did not and that testing rates would increase post‐PHE declaration.

**Study Design**: Patients receiving prenatal care and delivered within a multihospital academic healthcare system with urban, suburban, and rural hospitals from 8/23/2016‐12/31/2018 were included. Patient addresses were matched to the CDC Social Vulnerability Index to obtain overall and domain‐specific (Household Composition, Socioeconomic Status, Racial/Ethnic Minority Status, and Housing/Transportation) SV scores. Scores ≥0.75 indicate high SV. Chi‐squared or Fisher's exact tests were used to compare categorical variables, and Mann‐Whitney U tests were used to compare continuous variables.

**Results**: 3,938 were included; 3,405 (86%) did not receive uTox testing, and 533 (14%) did. Testing rates did not differ pre‐ and post‐PHE (14% vs 13%; p = 0.25). Patients with uTox testing were more likely to be non‐Hispanic Black or Hispanic, older, unmarried, without private insurance (Table 1). Overall elevated SV scores were significantly higher among those who received uTox testing and individually in all domains except for racial/ethnic minority status (Table 1). Those who received testing were more likely to have inadequate prenatal care, fetal or neonatal demise, abruption, PPROM and poor pregnancy outcomes (Table 2).

**Conclusion**: The PHE declaration did not impact testing rates. Demographic and other clinical differences in uTox testing may highlight provider biases. A high SV score may be a helpful tool for investigating other ways to make substance use testing in pregnancy more reliable.

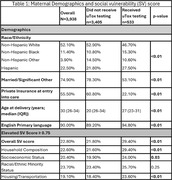

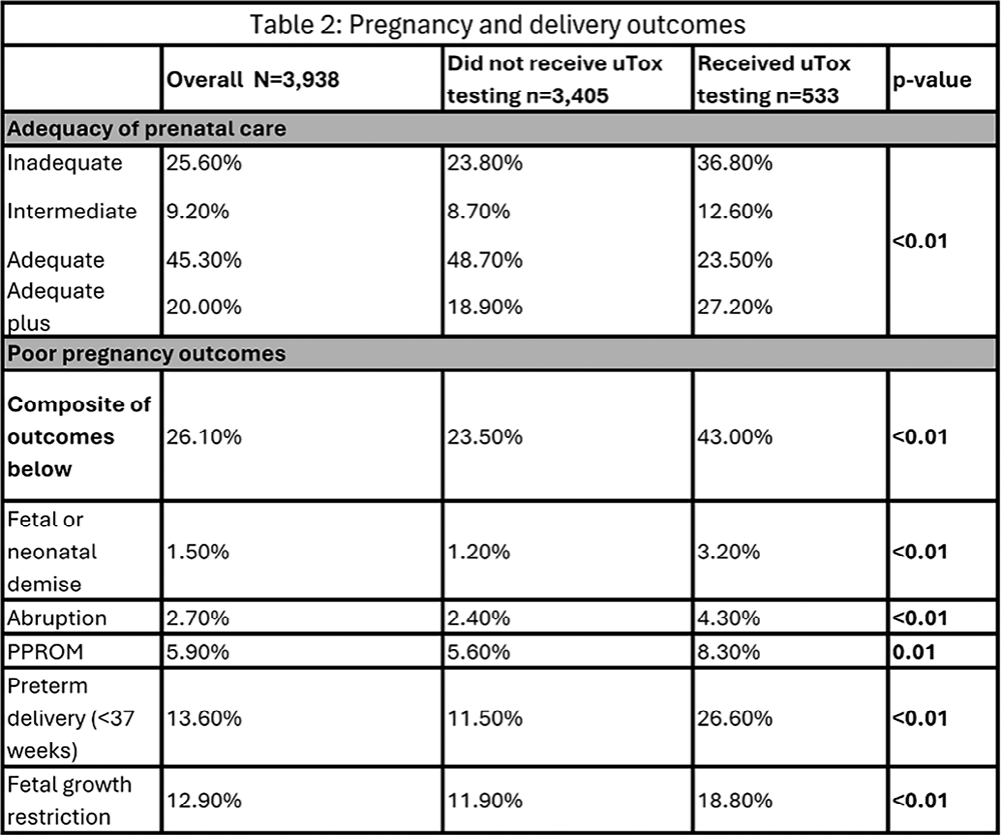

## 1219 | Factors Associated with Subsequent Diagnosis of T2DM Following an Initial Pregnancy with GDM

Wendy Tian; Bharti Garg; Alyssa R. Hersh; Aaron B. Caughey

*Oregon Health & Science University, Portland, OR*

4:00 PM ‐ 6:00 PM

**Objective**: Gestational diabetes (GDM) affects more than 200,000 people annually in the US, with prevalence continuing to rise. GDM is associated with development of type 2 diabetes mellitus (T2DM) in the future, which can affect subsequent pregnancies. The purpose of this study was to identify factors that are associated with the development of T2DM in a subsequent pregnancy in patients who had GDM in their initial pregnancy.

**Study Design**: This was a retrospective cohort study of pregnant persons in California between 2008‐2020 with two births that were both singleton, non‐anomalous, gestational ages 23‐42 weeks. Included persons had GDM in their index pregnancy. We assessed factors associated with development of T2DM in a subsequent pregnancy compared to those who did not develop T2DM. Statistical analyses were performed utilizing chi squared and multivariable logistic regression with a p‐value of 0.05.

**Results**: There were 65,160 (6.9%) of patients who had GDM in their initial pregnancy, of which 3,904 (6.0%) developed T2DM in their second pregnancy. Factors associated with development of T2DM at second delivery included non‐Hispanic black race (aOR 1.98, CI 95% 1.67‐2.35), Hispanic ethnicity (aOR1.88, CI 95% 1.70‐2.08), overweight or obese body mass index (BMI) (aOR 1.81, CI 95% 1.62‐2.02; aOR 3.61, CI 95% 3.27‐3.99), public insurance (aOR 1.26, CI 95% 1.17‐1.36), and diagnosis of chronic hypertension (CHTN) (aOR 1.93, CI 95% 1.66‐2.25). Patients that were less than 20 years of age were less likely to develop T2DM in their subsequent pregnancy (aOR 0.82, CI 95% 0.68‐0.98).

**Conclusion**: We found that a new diagnosis of T2DM in a subsequent pregnancy after an initial pregnancy with GDM is associated with several factors, including non‐Hispanic black race, Hispanic ethnicity, overweight or obese BMI, public insurance and diagnosis of CHTN during initial pregnancy. These findings can be used when counseling patients with a history of GDM to discuss the risk of development of T2DM in subsequent pregnancies. Future studies should assess structural and sociocultural factors that may contribute to these findings.

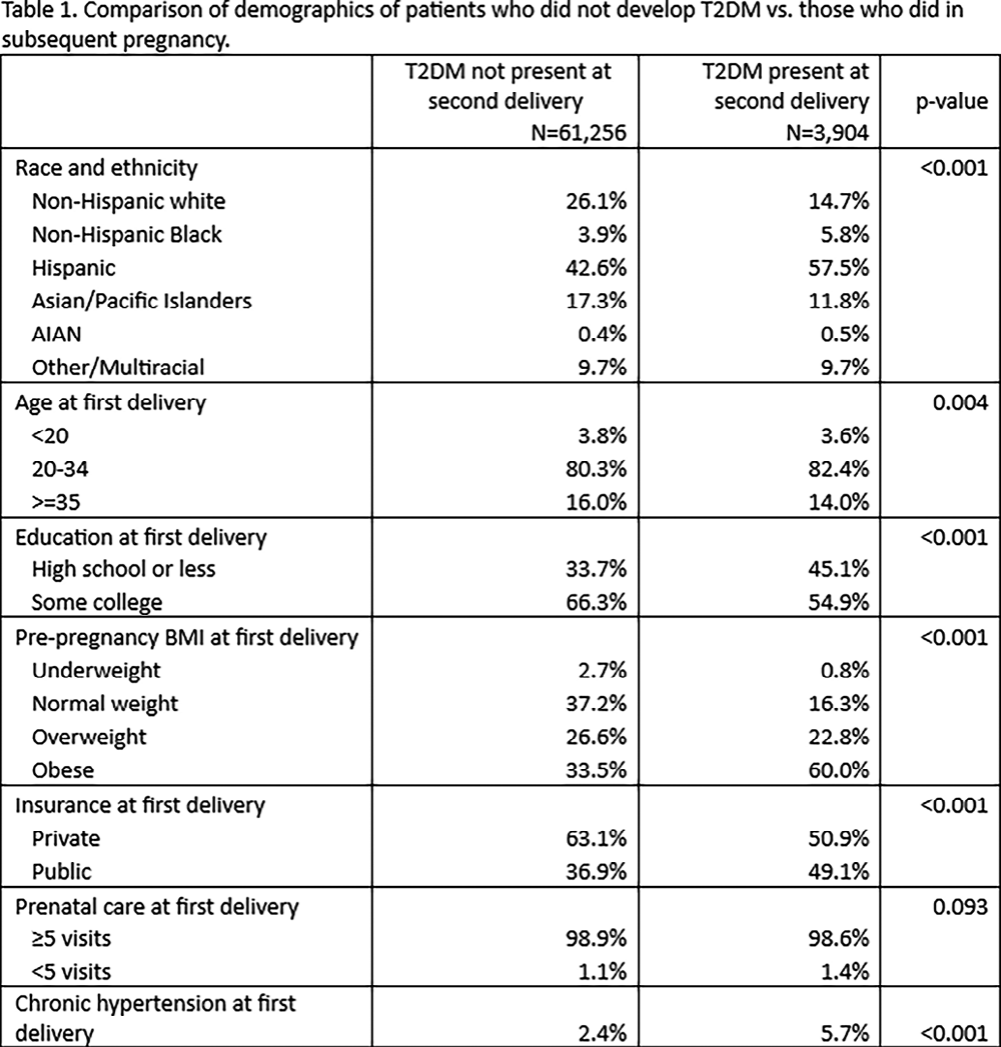

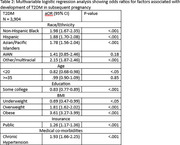

## 1220 | Association of Active Antenatal Management before Periviable Birth and Birthing Parent race in the US

Niurka L. Monteserin^1^; Whitney Bender^2^

*^1^Virginia Women's Center, Richmond, VA; ^2^Thomas Jefferson University Hospital, Philadelphia, PA*

4:00 PM ‐ 6:00 PM

**Objective**: sMFM provides guidance on the use of antenatal corticosteroids, antibiotics, and cesarean delivery in the periviable period. Although differences exist in postnatal active management of periviable neonates by parent race, less is known about the receipt of antenatal interventions. The objective of this study is to describe differences in antenatal interventions before a periviable delivery by birthing parent race.

**Study Design**: This is a population‐based cohort study of the National Center for Health Statistics database of periviable deliveries (20w0d‐25w6d) from 2016‐2021. Subjects were included if they delivered a non‐anomalous live singleton gestation in a hospital. To minimize inaccurate dating, subjects were excluded for birthweight >97% for gestational age. The exposure was self‐reported race; those of Black race were compared to those of non‐Black race. Outcomes included receipt of antenatal steroids or antibiotics, and cesarean delivery. The relationship of race to antenatal interventions was assessed using multivariable logistic regression.

**Results**: Of 45339472 live births during the study period, 93686 met inclusion criteria for this study. 37626 (40.2%) periviable births occurred in Black subjects. Black subjects differed from non‐Black subjects in baseline characteristics (Table 1). As seen in Table 2, Black subjects were less likely to receive antenatal steroids (33.5% v. 34.3%, aOR 0.93, 95% CI 0.90‐0.96), receive antibiotics (36.8% v. 38.1%, aOR 0.96, 95% 0.93‐0.99), or receive antibiotics in the presence of intraamniotic infection(64.7% v. 67.4%, aOR 0.87, 95% CI 0.78‐0.97). There were no differences in mode of delivery in adjusted analyses.

**Conclusion**: Black birthing parents are less likely to receive antenatal steroids or antibiotics prior to a periviable delivery. Given the complexity of counseling and clinical‐decision making required in those at risk of periviable birth, additional research is warranted to understand the root of these disparities with the goal of equitable, patient‐centered care in the periviable period.

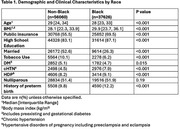

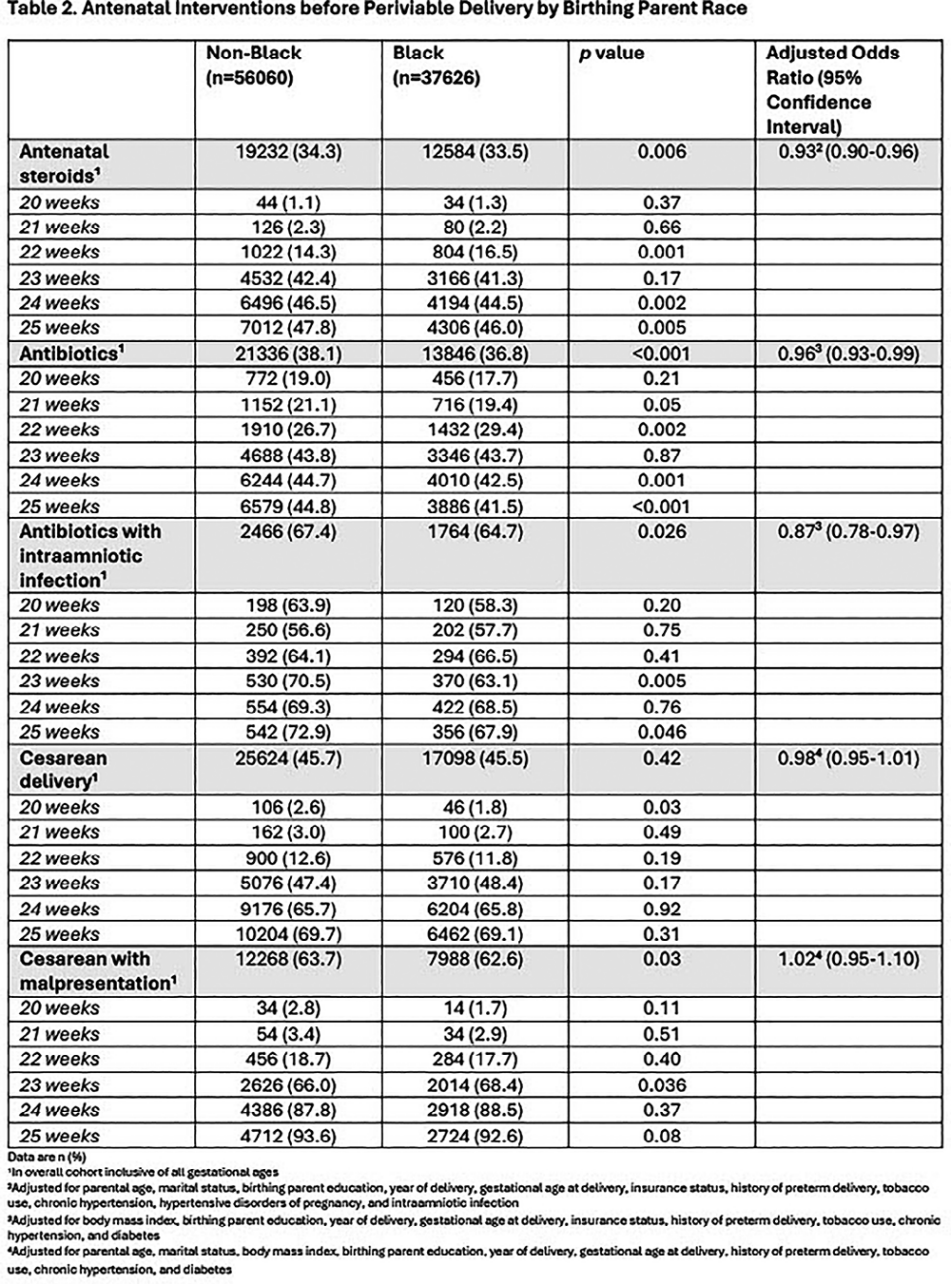

## 1221 | Is Detectable Deleterious? Comparing Maternal and Neonatal Factors Stratified by Hepatitis C Viral Load Detection

William L. Riley^1^; Rebecca Purvis^2^; Alicia Mastronardi^3^; Jill M. Maples^4^; Molly Rigell Peek^1^; Elizabeth Kirby^5^; Zuha Khan^5^; Mary Herndon^1^; Emily Katz^6^; Lynlee M. Wolfe^1^; Kimberly B. Fortner^1^; Callie Reeder^1^

*^1^University of Tennessee Graduate School of Medicine, Knoxville, TN; ^2^University of Tennessee Medical Center Knoxville, Knoxville, TN; ^3^University of Tennessee Graduate School of Medicine, Department of OB/Gyn, Knoxville, TN; ^4^University of Tennessee Health Science Center, College of Medicine, Knoxville, TN; ^5^University of Tennessee, Knoxville, TN; ^6^High Risk Obstetrical Consultants of Knoxville, Knoxville, TN*

4:00 PM ‐ 6:00 PM

**Objective**: To investigate maternal and neonatal risk factors and outcomes among pregnant individuals with positive hepatitis C virus (HCV) screening results stratified by undetectable or detectable HCV RNA or viral load (VL).

**Study Design**: This retrospective cohort study included all deliveries between January 2022 and June 2023 at our institution with positive HCV antibody screening results and quantitative HCV RNA testing. Data on demographics and maternal and neonatal outcomes were collected. Comparisons were performed on factors and outcomes stratified by undetectable or detectable VL. Pearson chi‐square tests were used to compare populations and outcomes as appropriate and can be found in the table.

**Results**: Of the 6,337 births with HCV screening results, the prevalence of positive HCV screening serology was 3.6% (n = 228). HCV VL results were available for 206 patients. When stratified by undetectable or detectable VL, there were no statistically significant differences in preterm delivery, hypertensive disorders of pregnancy, late or limited prenatal care, delivery route, NICU admission, or neonatal abstinence syndrome diagnosis. There were no differences in the rate of self‐reported current substance misuse or a history of IV substance misuse. A history of substance misuse was significantly higher in patients with a detectable viral load (92.4% vs. 83.2%, p = .043). Full outcome data is shown in the table.

**Conclusion**: Although obstetric complications have been reported among women with HCV, the frequency of these outcomes remained unchanged when stratified by detectable VL. The only factor associated with detectable VL was a self‐reported history of substance misuse. HCV genotyping remains an important factor in determining treatment type and duration. Future research will focus on understanding factors related to HCV infection that drive delivery and neonatal outcomes, including the temporal association of last substance use, IV misuse, and VL in pregnancy.

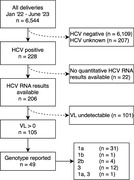

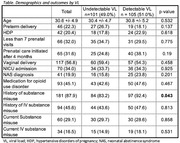

## 1222 | Collagen's Critical Role in Uterine Structure and Function and Implications in Placenta Accreta Spectrum

Sohum Shah^1^; Lior Kashani Ligumsky^1^; Alex Kot^1^; Liwen Xu^1^; Anhyo Jeong^1^; Guadalupe Martinez^1^; Scott A. Shainker^2^; Deborah Krakow^1^; Yalda Afshar^1^

*^1^University of California, Los Angeles, Los Angeles, CA; ^2^Maternal Fetal Care Center Associate Professor of Surgery Associate Professor of Obstetrics, Gynecology and Reproductive Biology Harvard Medical School, Boston, MA*

4:00 PM ‐ 6:00 PM

**Objective**: Properly synthesized and cross‐linked collagen fibrils are the principal source of tensile strength in tissues and altered during pregnancy. We previously described changes in collagen at the decidual‐stromal interface in placenta accreta spectrum (PAS). We aim to describe histoarchitectural variables of collagen organization in the uterus of wild type and mutant type I collagen mice to understand the role of collagen in pregnancy and repair as it related to PAS.

**Study Design**: We utilized two mutant type I collagen mice: *Col1a1^Aga2/+^* (*Aga2/+*) mice which express modified *Col1a1, *C‐terminal frameshift mutation, and *Col1a1^+/‐^* which express reduced levels of type I collagen. These mice, historically used in the study of skeletal dysplasias, allow us to interrogate the role of type I collagen. Uteri and placenta from wildtype (WT)*, Col1a1^+/‐^*, and *Aga2/+* mice were harvested. Additionally, we utilized a surgical mouse model of PAS. We employed quantitative histomorphometry with standard histochemistry as well as label‐free 3D spectroscopy to understand collagen fibril orientation and distribution.

**Results**: Type I (Col1a1) collagen was expressed in WT non‐pregnant mice and at postpartum day (PP). In the WT, collagens were organized around smooth muscle and in the basement membranes of luminal and glandular epithelium (Fig 1). PP type I collagen staining was prominent within the endometrial stroma. *Aga2/+* mice demonstrated decreased type I collagen with a compensatory increase in type III collagen but no difference in type I and III collagen PP. *Aga2/+* stromal thickness was decreased. Utilizing the surgical PAS mouse, RNA and protein expression of COL1A1 was altered and there was an increase deposition of type III collagen at the decidual‐placental interface in PAS (Fig 2).

**Conclusion**: Collagens are a main component of the dynamic architecture of the uterus. Type I collagen remodeling is a physiological component of pregnancy and birth and altered in PAS. The increased abundance and disorganization of collagen fibers at the site of adherence in PAS provide spatiotemporal clues to the mechanisms of PAS.

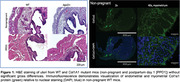

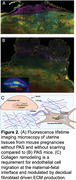

## 1223 | Cervicovaginal Microbiome in Pre vs Post Cerclage Placement

Yasaman C. Yaghoubian^1^; Dzhamala Gilmandyar^2^; Burton Rochelson^3^; Jane Cerise^4^; Anette Lee^4^

*^1^Zucker School of Medicine at Hofstra/ Northwell, Queens, NY; ^2^Zucker School of Medicine, Queens, NY; ^3^Northwell, New Hyde Park, NY; ^4^Feinstein Institute for Research, Manhasset, NY*

4:00 PM ‐ 6:00 PM

**Objective**: Though an association between changes in the cervicovaginal microbiome (CVM) and preterm birth has previously been demonstrated, the impact of cerclage placement on the CVM is poorly understood. Our objective was to assess significant changes in CVM due to cerclage placement, and association of CVM makeup with preterm delivery in patients with cervical insufficiency requiring cerclage placement.

**Study Design**: This was a prospective cohort study of singleton, non‐anomalous pregnancies from 2022‐2023 at two tertiary care centers. We enrolled 22 subjects receiving ultrasound or physical exam indicated cerclage, and 20 subjects with no prior preterm delivery and a normal cervical length as controls. Cervicovaginal fluid samples were collected prior to, and 1‐2 wks post cerclage placement for the study group, and during routine anatomical surveys for the control group. Samples were analyzed using 16S rRNA gene sequencing.

**Results**: We detected no significant difference in Shannon diversity between study and control groups. However, a significant difference in distribution was observed in Shannon diversity between preterm and term deliveries (p = 0.01, Graph1). There were significant differences in differential abundance of 14 taxa in study vs control groups, and 4 taxa in preterm vs term deliveries (Table 1).

When comparing samples pre vs post cerclage placement, no significant difference was observed in Shannon diversity. However, a significant difference in distributions was observed in beta diversity between these samples (p = .04). There were no differences in specific taxon abundance between pre and post cerclage samples.

**Conclusion**: In a diverse patient population, differences in CVM distribution are associated with preterm delivery. The abundance of certain taxa may be associated with cervical insufficiency and preterm birth. Though cerclage placement did not implicate a difference in specific taxa present, it does appear to alter the distribution of the CMV.

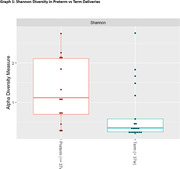

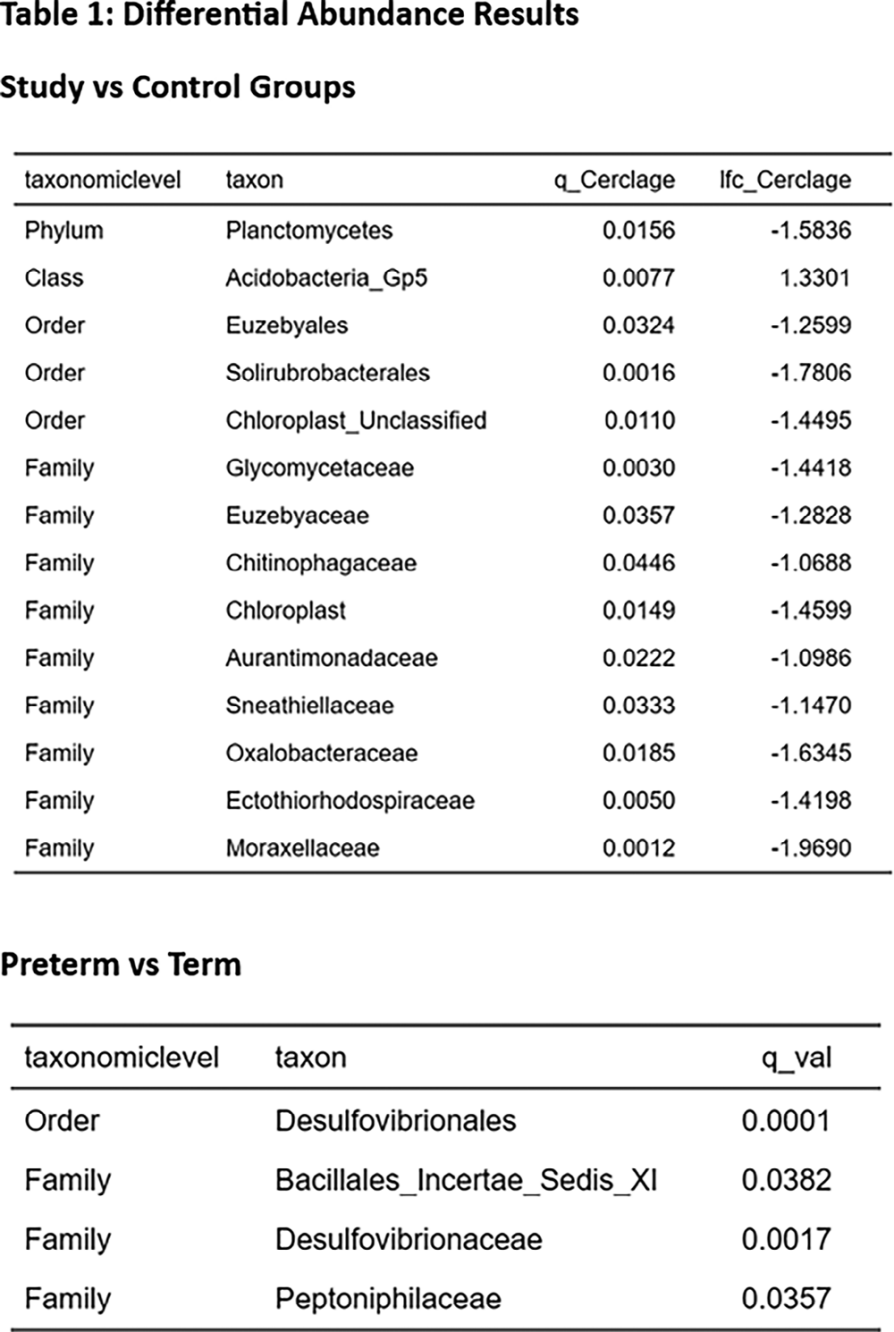

## 1224 | Do Maternal Fetal Medicine Fellowship Websites Adequately Depict Training Opportunities?

Zenobia Gonsalves^1^; Rachel Lee^2^; Lama R. Noureddine^3^; Kimberly Herrera^4^; Cassandra Heiselman^1^

*^1^Stony Brook University Hospital, Stony Brook, NY; ^2^Stony Brook University Hospital, Lake Grove, NY; ^3^Rutgers New Jersey Medical School, Newark, NJ; ^4^Stony Brook University, Stony Brook, NY*

4:00 PM ‐ 6:00 PM

**Objective**: After the COVID‐19 pandemic and with current virtual fellowship interviews, applicants rely heavily on programs’ websites early in the application process to assess a program's unique training attributes and fit with their professional goals. We aimed to evaluate and compare the availability of information on education and training on MFM fellowship program websites between 2021 and 2024.

**Study Design**: This was a cross‐sectional analysis of MFM fellowship websites for the 109 ACGME‐accredited programs. Fellowship program websites had previously been reviewed in July 2021 and then again in July/August 2024 for the following information: delivery and antepartum volumes; level of neonatal intensive care units (NICU); regional perinatal center designation; sample curriculum with didactic schedule, simulation sessions, and call responsibilities; and training in placenta accreta spectrum (PAS) disorders, prenatal diagnostic procedures, and dilatation and evacuation (D&E). The percentage of websites that included each of these components was calculated and compared across time points. Chi‐square and Fisher's exact analyses were performed with a p‐value < 0.05.

**Results**: 107 (98%) of the 109 ACGME‐accredited MFM fellowship programs had an official website as of August 2024. Less websites were “up to date” for the current academic year in July 2024 (50%) compared to July 2021 (65%) [p = 0.04]. A larger proportion of programs in 2024 included information about the didactic schedule (p = 0.029), procedure training (p = 0.014), simulation training (p = 0.01), NICU level (p = 0.014), and perinatal center designation (p = 0.002) [Figure 1]. However, a smaller percentage of websites in 2024 included the rotation schedule as compared to 2021 (56% vs 71%, p = 0.03).

**Conclusion**: This study demonstrates continued gaps in the availability of desired information about education and training on MFM fellowship program websites. Programs should continue to improve information availability to enhance the application process, particularly as fellowship programs look to adopt signaling as AGCME residency applications have implemented.

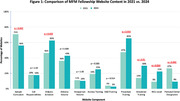

## 1225 | Uterine Electrical Activity in Labor: EMMI Insights on Contraction “Pacemakers”

Zichao Wen^1^; Hui Wang^2^; Hansong Gao^2^; Yuelin Li^2^; Yuan Nan^2^; Josephine Lau^2^; Yong Wang^2^

*^1^Washington University School of medicine in St. Louis, St. Louis, MO; ^2^Washington University School of Medicine in St. Louis, St. Louis, MO*

4:00 PM ‐ 6:00 PM

**Objective**: This study aims to analyze the correlation between initiation and duration of Electromyometrial Imaging (EMMI)‐reconstructed electrical activity across the whole 3D uterine surface, which could provide new insights into electrophysiological “pacemakers” in human labor contractions.

**Study Design**: The Washington Institutional Review Board approved this prospective cohort study. Following informed consent, 38 subjects underwent MRI at ∼37 weeks of gestation to capture subject‐specific body‐uterus geometry. Upon admission to Labor & Delivery Unit and during active labor (≥4 cm dilation with regular contractions), ≤192 electrodes were applied to the abdomen and back. Electrical potentials on the body were recorded for ∼1 hour. EMMI generated uterine potential maps, electrogram bursts, and isochrone maps of burst initiation and duration (Fig. 1). Four representative uterine EMG (A‐D) are shown.

**Results**: A strong negative correlation (Fig. 2) was observed between burst initiation time and duration, suggesting uterine regions with earlier activation had longer burst durations. Uterine locations B‐D in Fig. 1 are depicted in the scatter plot. Statistics of R2, P‐values, and slopes for 482 contractions from 38 subjects are also shown in Fig. 2. This finding aligns with the concept that the uterus does not have a single, dedicated, anatomical pacemaker, but instead shows variable initiation sites for each contraction, supporting the idea of multiple, widely dispersed, functional “pacemakers”.

**Conclusion**: The inverse relationship between burst initiation time and duration highlights the importance of early‐activating regions in maintaining normal uterine contractions. These findings challenge the traditional notion of an anatomically fixed uterine pacemaker like the heart's sinoatrial node. Instead, the data support the hypothesis that the uterus operates with multiple functional “pacemakers,” likely coordinated through intrauterine pressure. Future studies should address potential bias from varying detected contractions per subject to refine these findings and explore uterine contraction coordination mechanisms.

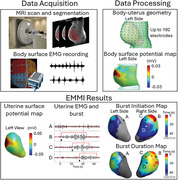

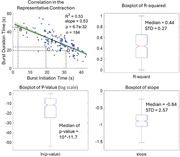

## 1226 | Determining Potentially Avoidable Blood Transfusion after Cesarean Delivery: A Retrospective Cohort

Zoya Pervaiz Butt; Rebecca F. Hamm

*University of Pennsylvania Perelman School of Medicine, Philadelphia, PA*

4:00 PM ‐ 6:00 PM

**Objective**: Prior work shows that actual blood loss is similar between vaginal (VD) and cesarean deliveries (CD). Yet, 12‐hour complete blood counts (12hCBCs) are only routinely performed after CD, possibly contributing to a higher blood transfusion rate after CD as compared to VD. We sought to determine the proportion of blood transfusions after CD performed secondary to the routine post‐CD 12hCBC alone, thus potentially avoidable.

**Study Design**: This retrospective cohort evaluated indications for packed red blood cell (pRBC) transfusion after CD in the postpartum (PP) period at a single institution from 2017‐2022. Patients were evaluated for: pRBC transfusion before or after 12hCBC, high risk clinical criteria for PP anemia warranting a CBC (defined as ≥1 of the following: EBL≥1L, ≥3 CD, placental abnormalities, severe preeclampsia, multiple gestation, bleeding/clotting disorder, antepartum pRBC transfusion, admission Hb< 9.5g/dL, and surgical injury), and documented symptomatology (lightheadedness and/or fatigue) or abnormal vital signs (VS, defined as ≥1 of the following HR≥100; BP< 90/50; O2 sat < 95%) prior to transfusion. Those transfusions that occurred post‐12hCBC, without high‐risk clinical criteria, symptoms, or abnormal VS, were deemed potentially avoidable without a routine 12hCBC. Descriptive statistics were utilized.

**Results**: 421 patients were included; demographics are shown in the Table. Of those 421, 244 (58.0%) of post‐CD transfusions occurred after the 12hCBC. Among those 244, 216 had at least one of the high‐risk clinical criteria. Of the remaining 28 (6.6% of the initial cohort), 17 had either documented symptoms or abnormal VS. Thus, of 421 patients receiving pRBC transfusions post‐CD after the 12hCBC, only 11 (2.6%), encompassing 19 pRBC units out of 984 transfused in the cohort (1.9%), were deemed potentially avoidable (Figure).

**Conclusion**: This study highlights that the majority (>97%) of patients who received pRBCs post CD would not have avoided the transfusion even in the absence of a routine 12hCBC. Alternative interventions to reduce unnecessary post‐CD transfusion should be pursued.

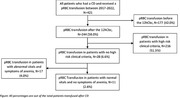

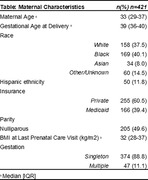

## 1227 | Severe Early Post‐Partum Hemorrhage: An Individualized Risk Assessment by Machine Learning Models

Ido Bachar^1^; Zvi Ehrlich^2^; Ari Weiss^3^; Rivka Farkash^4^; Tzuria Peled^3^; Sorina Grisaru Granovsky^5^

*^1^Bnei Zion, Bnei Zion, Yerushalayim; ^2^Shaare Zedek Medical Center, Jerusalem, Yerushalayim; ^3^Shaare Zedek Medical Center, Shaare Zedek Medical Center, Yerushalayim; ^4^SZMC, SZMC, Yerushalayim; ^5^Hebrew University, Jerusalem, Yerushalayim*

4:00 PM ‐ 6:00 PM

**Objective**: Severe early post‐partum hemorrhage (SEPPH) is a common, life‐threatening obstetric challenge. The risk factors of early postpartum hemorrhage had been researched, however, early, upon admission for labor, recognition of high‐risk parturients by machine learning models (ML) has not been attempted.

**Study Design**: Analysis of a multi‐parameter dataset of 227,424 births at a tertiary medical center. SEPPH was defined as ‐ Hemoglobin loss more than 4 g/dl or post‐partum hemoglobin level less the 8 g/dl or treatment of 2 or more pRBC post‐partum. For the ML analyses, the data set was divided into two groups: SEPPH and no SEPPH; analysis was conducted using a gradient boosting model (CatBoost) algorithm, to create a model predicting the risk score of SEPPH. Furthermore, this type of analysis categorized the study population high‐risk and low‐risk groups for SEPPH by using the Youden's Index.

**Results**: Study population is depicted in Figure 1. Using traditional statistical methods dissected into the individual risk factors for SEPPH. Notably, we revealed that the primiparity is a major risk factor for extreme postpartum hemorrhage (OR 4.5 [4.0‐5.0] CI, p< 0.001). In addition, Multiple gestation (OR 2.5 [2.0‐3.1] CI p< 0.001), previous caesarean section (OR 3.0 [2.6‐3.5] CI, p< 0.001), placentation anomaly (OR 4.0 [3.424‐4.814] CI, p< 0.001), were significant risk factors.

The high‐risk group identified by the ML model showed a significant higher risk for adverse maternal and neonatal outcomes including among the low and high risk for SEPPH: uterine rupture (OR‐3.5), re‐admission 42 and 90 days after delivery (OR ‐2.2, 2.2 respectively) and perinatal fever (OR‐2.2), uterine scar dehiscence (OR‐1.9). Newborn infection (OR‐2.5), postnatal intensive care unit (NICU) stay >3 days (OR 2.4, 2.37 respectively) and low Apgar (1‐7) at 1^st^ and 5^th^ min (OR 2.0, 1.26 respectively). Figure ‐2.

**Conclusion**: We built a machine learning model with defined measurable predictable factors to identify high risk for SEPPH. Prediction models for early PPH may allow appropriate risk reducing preventive care during and after delivery.